# Catalytic asymmetric total syntheses of myrtucommuacetalone, myrtucommuacetalone B, and callistrilones A, C, D and E†
†Electronic supplementary information (ESI) available. CCDC 1526145, 1526146, 1582656, 1582657 and 1582658. For ESI and crystallographic data in CIF or other electronic format see DOI: 10.1039/c7sc04672c


**DOI:** 10.1039/c7sc04672c

**Published:** 2017-11-27

**Authors:** Min-Jing Cheng, Jia-Qing Cao, Xin-Yi Yang, Li-Ping Zhong, Li-Jun Hu, Xi Lu, Bao-Long Hou, Ya-Jian Hu, Ying Wang, Xue-Fu You, Lei Wang, Wen-Cai Ye, Chuang-Chuang Li

**Affiliations:** a College of Pharmacy , Jinan University , Guangzhou 510632 , China . Email: cpuwanglei@126.com ; Email: chyewc@gmail.com; b Department of Chemistry , Southern University of Science and Technology , Shenzhen 518055 , China . Email: ccli@sustc.edu.cn; c Institute of Medicinal Biotechnology , Chinese Academy of Medical Sciences , Peking Union Medical College , Beijing 100050 , China

## Abstract

Herein, we describe a concise catalytic approach to the first asymmetric total syntheses of myrtucommuacetalone, myrtucommuacetalone B, and callistrilones A, C, D and E. The syntheses proceed in only 5–7 steps from the readily available compound **11**, without the need for protecting groups. Key features of the syntheses include a unique organocatalytic asymmetric Friedel–Crafts-type Michael addition with high enantioselectivity and a broad substrate scope, a novel Michael-ketalization-annulation cascade reaction, and an oxidative [3 + 2] cycloaddition. Furthermore, the new compound **7** exhibited potent antibacterial activities against several multidrug-resistant strains (MRSA, VISA and VRE), and showed greater potency than vancomycin.

## Abstract 2

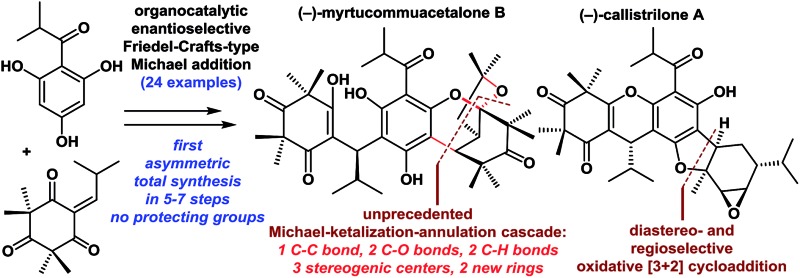
A highly concise catalytic approach for the first asymmetric total syntheses of myrtucommuacetalones and callistrilones is reported.

## Introduction

**Fig. 1:**
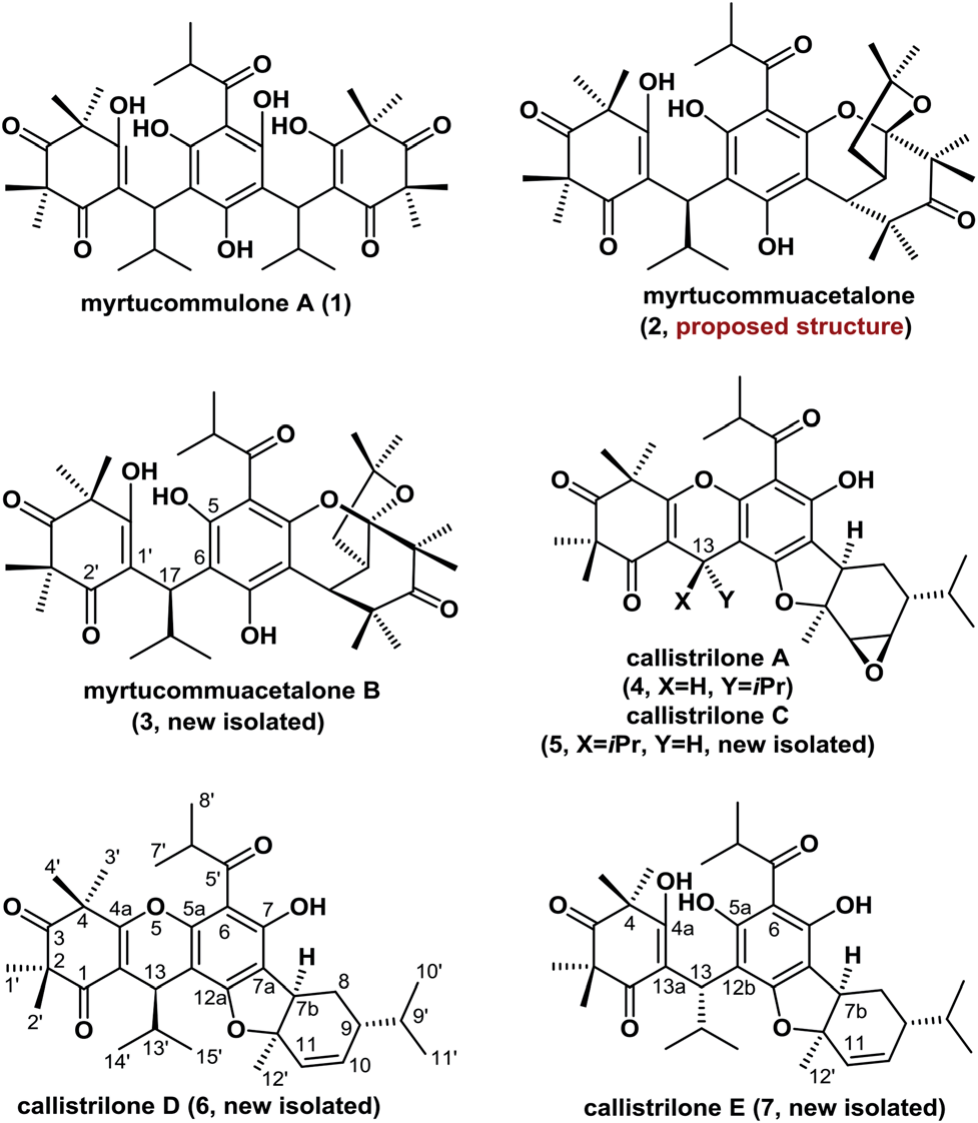
Representative polycyclic polymethylated phloroglucinols.

Polycyclic polymethylated phloroglucinols (PPPs) are a class of natural product isolated mainly from the plants of the *Myrtaceae* and *Guttiferae* families. A diverse range of more than 70 PPPs have been isolated. These natural products have become attractive targets for chemists because of their complex structural features, and some PPPs have been reported to exhibit biological activities.^1^,^2^ For example, myrtucommulone A (**1**), which was first isolated in 1974, is highly active against Gram-positive bacteria and cancer cells.1a,b Myrtucommuacetalone (**2**) exhibits inhibitory activity towards the production of nitric oxide (NO˙), as well as pronounced antiproliferative activity against T-cells.^3^ Structurally, the naturally occurring PPPs **1–7** are based on a diverse range of complex scaffolds (Fig. 1). Myrtucommuacetalone (**2**) and myrtucommuacetalone B (**3**) consist of a synthetically challenging and unprecedented bridged furochromene moiety with a fascinating polycyclic ketal skeleton and a 2-oxabicyclo[3.3.1]nonane scaffold. Callistrilones (**4–7**) are based on a previously unknown carbon skeleton consisting of a unique [1]benzofuro[2,3-*a*]xanthene ring system.^4^ Furthermore, compounds **4** and **5** consist of a sterically compact 6/6/6/5/6/3-fused hexacyclic skeleton, containing six stereocenters with one tetrasubstituted center. Based on their structural complexity, the construction of compounds belonging to this family represents a synthetic challenge.

The synthetically challenging structural motifs of these PPPs together with their promising pharmacological properties have attracted considerable interest from the synthetic community.^5^,^6^ In 2010, Jauch *et al.* reported the first total synthesis of myrtucommulone A (**1**).5*a* However, the asymmetric total syntheses of compounds **2–7** are yet to be reported and the catalytic asymmetric and divergent syntheses of PPPs have not been achieved. In our continuing efforts towards the synthesis of biologically active natural products,^7^ herein we describe the isolation and identification of four novel PPPs (**3** and **5–7**) from the plants *Callistemon rigidus* and *Myrtus communis*. Furthermore, we report the first catalytic asymmetric total syntheses of myrtucommuacetalone, myrtucommuacetalone B and callistrilones A, C, D and E in only 5–7 steps. Notably, the new compound **7** was found to exhibit antibacterial activity against multidrug-resistant strains.

## Results and discussion

### Isolation and structural elucidation of compounds **3** and **5–7**

The new compounds **3** and **5–7** were isolated from the plants *C. rigidus* and *M. communis* (Myrtaceae) by column chromatography and preparative high-performance liquid chromatography [see ESI†].

The molecular formula of compound **3** was established to be C_38_H_52_O_9_ based on its HRESIMS data (*m*/*z* 653.3688 [M + H]^+^, calcd for C_38_H_53_O_9_: 653.3684). The IR spectrum suggested the presence of aromatic (1592 and 1470 cm^–1^), hydroxyl (3202 cm^–1^), and carbonyl groups (1707 cm^–1^). Comparison of the ^1^H and ^13^C NMR data of **3** with those of myrtucommuacetalone (**2**) revealed that their chemical shifts were similar,^3^ except for differences in the C-17, C-5 and C-2′ signals, indicating that **3** was a C-17 epimer of **2**. This conclusion was further confirmed by X-ray diffraction analysis (see ESI†) and by our total synthesis.

The HRESIMS of **7** showed a quasimolecular ion peak at *m*/*z* 567.3328 [M + H]^+^ (calcd for C_34_H_47_O_7_: 567.3316), consistent with the molecular formula C_34_H_46_O_7_. The ^1^H and ^13^C NMR spectra of **7** displayed two sets of signals in a ratio of approximately 5 : 4, which suggested that this compound exists as a pair of rotamers owing to the intramolecular hydrogen-bonding. Comprehensive analysis of the NMR data of **7** indicated that it shared the same framework as callistrilone A (**4**),^4^ however, the signals for the epoxy carbons in **4** were replaced by signals for olefinic carbons and two additional hydroxyl signals were present in **7**. The HMBCs between OH-4a and C-4/C-13a, between OH-5a and C-6/C-12b, and between H-12′ and C-7b/C-11 confirmed its planar structure (Fig. S1–S7†). The unambiguous structural assignments and stereochemistry of **7** could be elucidated by X-ray diffraction (see ESI†) and the successful total synthesis.

Comparison of the NMR data of **5** and **6** with those of the known compound callistrilone A (**4**) suggested that they possessed a similar framework.^4^ A comprehensive analysis of their ^1^H–^1^H COSY, HSQC, HMBC, and NOESY spectra led us to conclude that **5** is the C-13 epimer of **4**, and the epoxy group in **5** is replaced by olefinic carbons in **6** (see ESI†), as confirmed by our asymmetric total synthesis.

### Retrosynthetic analysis of PPPs **2–7**

**Fig. 2:**
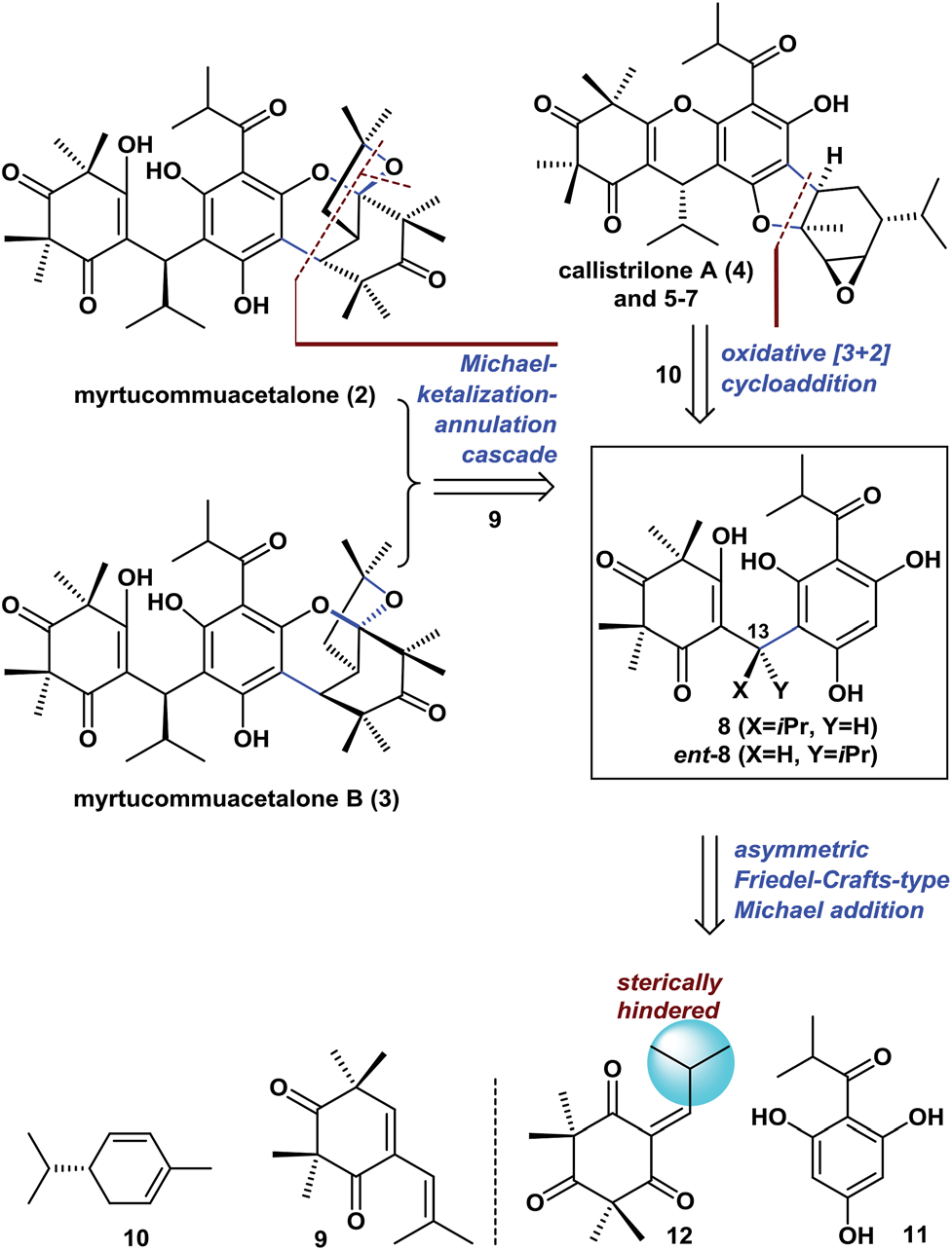
Retrosynthetic analysis of PPPs **2–7**.

**Scheme 1:**
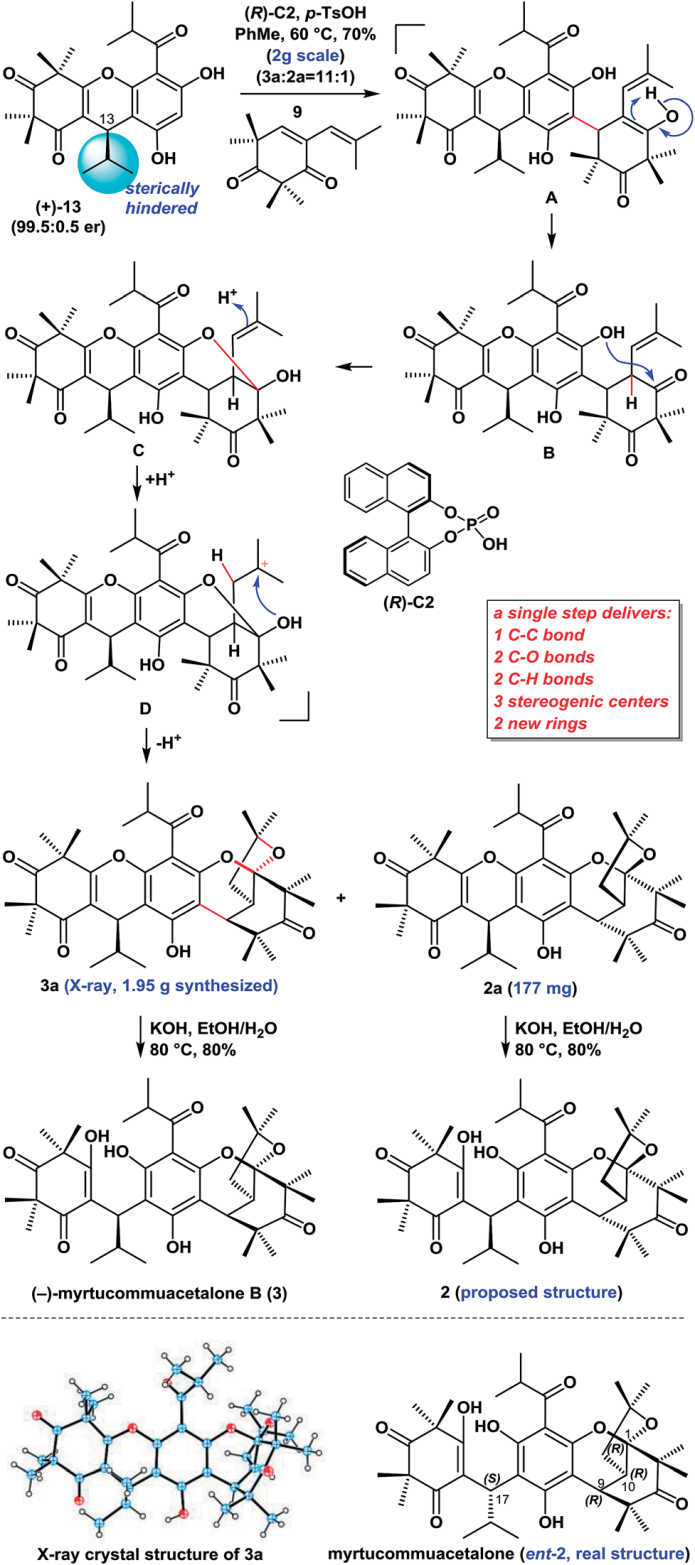
Asymmetric syntheses of **2** and **3**.

Retrosynthetically (Fig. 2), the bridged polycyclic ketal skeletons in **2** and **3** could be synthesized from **8**, by an unreported Michael-ketalization-annulation cascade reaction^8^ with compound **9** (see the proposed pathway in Scheme 1). Callistrilone A (**4**) could be synthesized from ***ent*-8** by a biomimetic oxidative [3 + 2] cycloaddition^9^ with commercially available (–)-α-phellandrene (**10**), followed by cyclization and epoxidation. In addition, several other natural PPPs isolated from *Myrtaceae* plants (such as **5–7**) could be constructed in a similar manner through a few simple functional-group transformations.

To achieve the enantioselective synthesis of **2–7**, it is essential to prepare **8** or ***ent*-8** with high enantioselectivity from compounds **11** and **12**, by Friedel–Crafts-type Michael (FCM) additions (Fig. 2). In recent years, enantioselective FCM additions with active aromatics have been intensively investigated.^10^ However, current approaches10g–n give only poor yields and/or poor enantioselectivities if the FCM acceptor is sterically hindered or alkyl substituted, as is the case for **12**. Moreover, there have been few literature reports concerning phloroglucinol derivatives as FCM donors. In particular, the three unprotected hydroxyl groups in **11**, which can undergo a competing oxa-Michael addition to give unexpected products, make this FCM addition more difficult. Recently, Jauch *et al.* reported the enantioselective synthesis of **8** with an 81 : 19 enantiomeric ratio (*er*), through the use of an excess (3 equiv.) of a chiral Al–Li–BINOL (1,1′-bi-2-naphthol) complex. These conditions resulted in an inseparable mixture of chiral (+)-**1** with an 85 : 15 *er* and *meso*-**1** in a ratio of 59 : 41.5*b* Thus, the catalytic and highly enantioselective synthesis of **8** and **2–7** remains a challenge to be addressed and is in demand.

### Organocatalytic enantioselective FCM additions

With compounds **11** and **12** 11 in hand, we proceeded to investigate the enantioselective FCM addition. Inspired by previous elegant work by Luo and co-workers,^12^ we envisioned that chiral phosphoric acids (CPAs),^13^ which have been used as powerful organocatalysts for numerous reactions over the past 12 years, might be able to facilitate this transformation enantioselectively. We initially conducted the FCM addition reaction of **11** and **12** in PhMe at –40 °C in the presence of 10 mol% of phosphoric acid (*S*)-C1 (entry 1, Table 1). Encouragingly, despite the high steric hindrance of **12**, the reaction proceeded smoothly to give **8**, followed by *p*-TsOH-mediated cyclization to give (+)-myrtucommulone B (**13**)1*m* in 35% yield with an 82.5 : 17.5 *er* (see ESI† for details). This proof of principle outcome showed that control of the C13 chirality of **8** (or **13**) was possible through the use of a chiral phosphoric acid-catalyzed asymmetric FCM addition. Next, we turned our attention to the effects of the substituents and axial chiral backbone of the catalysts to improve the enantioselectivity. As shown in Table 1, the electron-donating/withdrawing properties and steric bulk of the aromatic-ring substituents, as well as the nature of the backbone, had a considerable influence on the enantioselectivity. We further optimized the reaction conditions by changing the solvents and adding Lewis acids.^14^ After extensive experimentation, we identified the following protocol to be optimal (entry 31): when **11** was treated with **12** in the presence of catalytic (*S*)-C15 (10 mol%) and AlF_3_ (100 mol%) with 3 Å MS in PhMe at –70 °C for 6 days, followed by TsOH-mediated cyclization, (+)-(13*R*)-**13** 1m was obtained in a 75% isolated yield with a 95 : 5 *er* (2.0 g scale). After the recrystallization of **13**, its *er* value was improved to 99.5 : 0.5. Moreover, the catalyst (*S*)-C15 was easily recoverable and could be reused several times without a considerable loss of activity. We also achieved the highly enantioselective synthesis of (–)-(13*S*)-***ent*-13**, using (*R*)-C16 as the catalyst, according to the same procedure as above (entry 32). Notably, the route described above allowed for the facile synthesis of 10 g of both (+)-**13** and (–)-***ent*-13** (see ESI† for details), thereby highlighting the robust nature of this chemistry.

**Table 1:** The optimization of enantioselective FCM additions^*a*^

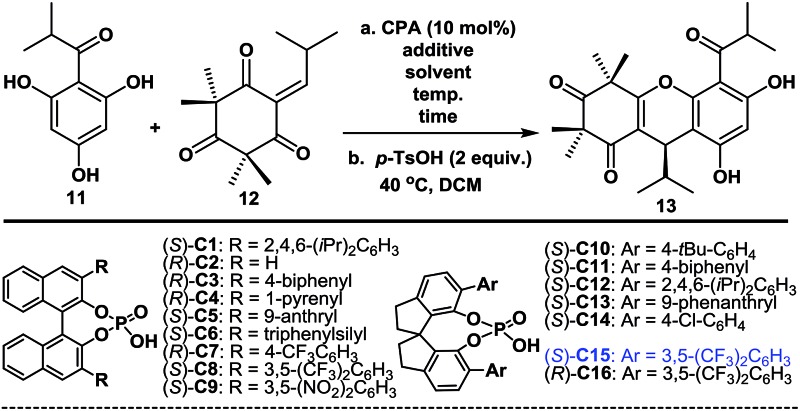							
Entry	Catalyst	Additive	*T* (°C)	Solvent	Time	Yield^*b*^ (%)	*er* ^*c*^
1	(*S*)-C1		–40	Toluene	24 h	35	82.5 : 17.5
2	(*S*)-C2		–40–26	Toluene	24 h	73	50 : 50
3	(*S*)-C3		–40	Toluene	24 h	Trace	—
4	(*S*)-C4		–40	Toluene	24 h	Trace	—
5	(*S*)-C5		–40	Toluene	24 h	18	52.5 : 47.5
6	(*S*)-C6		–40	Toluene	24 h	24	55 : 45
7	(*R*)-C7		–40	Toluene	24 h	30	40 : 60
8	(*S*)-C8		–40	Toluene	24 h	36	60 : 40
9	(*S*)-C9		–40	Toluene	24 h	Trace	—
10	(*S*)-C10		–40	Toluene	24 h	23	62 : 38
11	(*S*)-C11		–40	Toluene	24 h	21	0
12	(*S*)-C12		–40	Toluene	24 h	Trace	—
13	(*S*)-C13		–40	Toluene	24 h	25	64 : 36
14	(*S*)-C14		–40	Toluene	24 h	18	50 : 50
15	(*S*)-C15		–40	Toluene	24 h	73	88 : 12
16	(*S*)-C15		–60	Toluene	108 h	76	91 : 09
17	(*S*)-C15		–60	DCM	108 h	70	89.5 : 10.5
18	(*S*)-C15		–60	CHCl_3_	108 h	30	91 : 09
19	(*S*)-C15^*d*^		–60	Toluene	108 h	74	92 : 08
20	(*S*)-C15^*d*^	MgF_2_ (10%)	–60	Toluene	108 h	76	93 : 07
22	(*S*)-C15^*d*^	Fe(OTf)_3_ (10%)	–60	Toluene	108 h	74	91 : 09
23	(*S*)-C15^*d*^	Zn(OTf)_2_ (10%)	–60	Toluene	108 h	72	92.5 : 7.5
24	(*S*)-C15^*d*^	AIF_3_ (10%)	–60	Toluene	72 h	71	93.5 : 6.5
25	(*S*)-C15^*d*^	CaF_2_ (10%)	–60	Toluene	72 h	74	92.5 : 7.5
26	(*S*)-C15^*d*^	AlF_3_ (10%)	–76	Toluene	7 d	55	95.5 : 4.5
27	(*S*)-C15^*e*^	AlF_3_ (10%)	–76	Toluene	6 d	25	96.5 : 3.5
28	(*S*)-C15^*f*^	AlF_3_ (10%)	–76	Toluene	6 d	28	96 : 04
29	(*S*)-C15^*e*^	AIF_3_ (100%)	–70	Toluene	6 d	77	95 : 05
30	(*R*)-C16^*e*^	AIF_3_ (100%)	–70	Toluene	6 d	76	5 : 95
31	(*S*)-C15^*e*^ ^,^^*g*^	AlF_3_ (100%)	–70	Toluene	6 d	75	95 : 5 (99.5 : 0.5)^*h*^
32	(*R*)-C16^*e*^ ^,^^*g*^	AlF_3_ (100%)	–70	Toluene	6 d	75	5 : 95 (0.5 : 99.5)^*h*^

^*a*^Unless otherwise stated, the reactions of entries 1–30 were performed with **11** (0.1 mmol), **12** (0.2 mmol) and CPA (0.01 mmol) in toluene (2 mL) at –76 °C to 26 °C.

^*b*^Isolated yield.

^*c*^Determined by chiral HPLC analysis with a ChiralCel OD-H column (i-PrOH/*n*-hexane = 5 : 95, 0.8 mL min^–1^).

^*d*^4 Å MS (35 mg) was added.

^*e*^3 Å MS (35 mg) was added.

^*f*^5 Å MS (35 mg) was added.

^*g*^The reactions of entries 31 and 32 were performed with **11** (2.0 g, 10.2 mmol), **12** (20.4 mmol) and (*S*)-C15 or (*R*)-C16 (1.02 mmol) in toluene (204 mL) at –70 °C.

^*h*^The *er* could be easily improved by recrystallization.

With the optimized conditions in hand, we next examined the substrate scope of various substituted acylphloroglucinols. As shown in Table 2, different substituents of the substrates (**11** or **11a–11j**, see ESI† for details) were tolerated in the FCM addition with the Michael reaction acceptor **12**, to give the corresponding products **13a–13g** and **13o–13q** in good yields with *er* values of 91 : 9 to 95 : 5. Hence, our method has broad generality for the synthesis of polycyclic polymethylated phloroglucinol derivatives. Notably, when the *R*_1_ group was CHX_2_ (X = Et or Ph), the reactions were accelerated (**13a** and **13b**); however, the *er* values were slightly lower. Interestingly, when the *R*_1_ group was a 5-, 6- or 7-membered ring, there were no notable effects on the enantioselectivity of the FCM additions (**13o**, **13p** and **13q**).

**Table 2:** Substrate scope of the enantioselective FCM additions

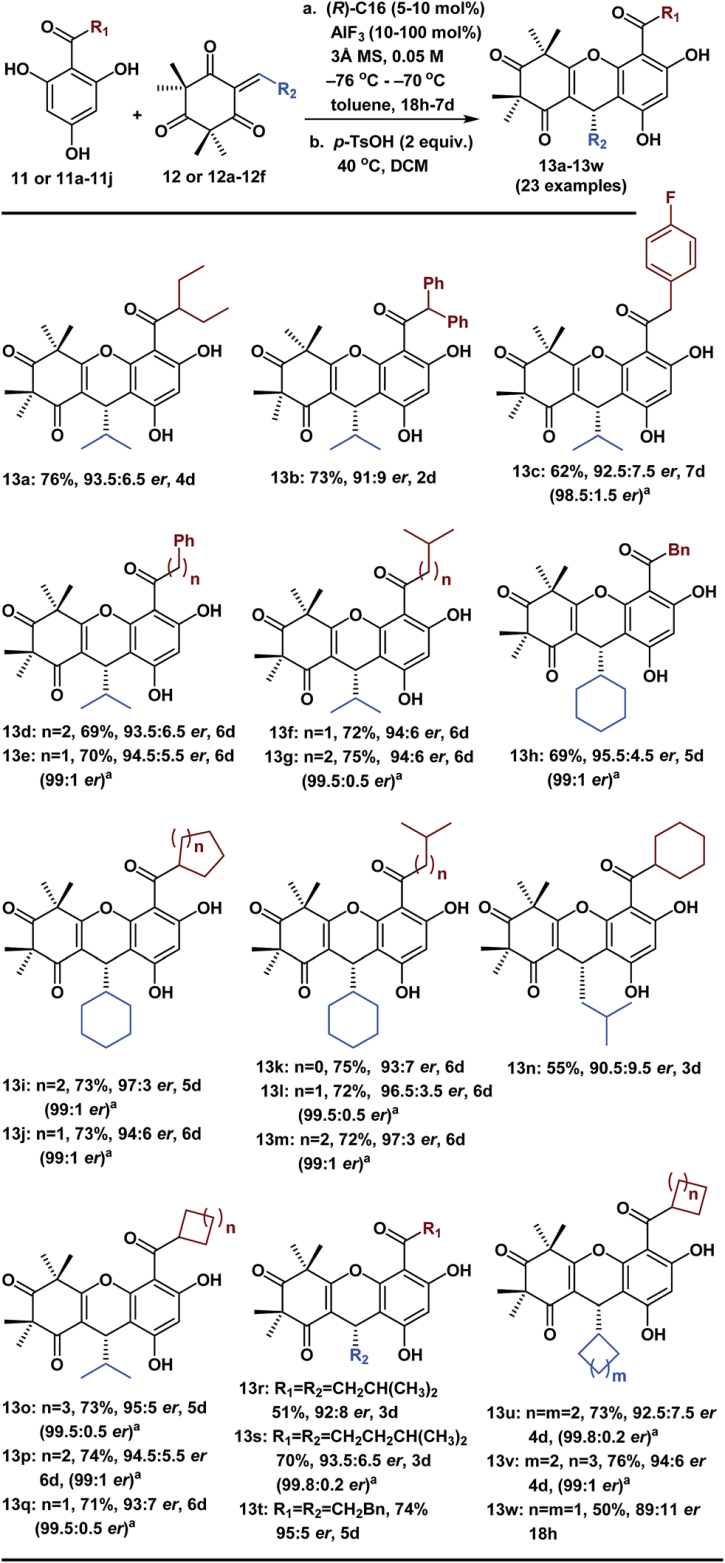

^*a*^The *er* could be easily improved by recrystallization.

Moreover, to validate the generality of this transformation, we evaluated the use of various sterically hindered Michael reaction acceptors (**12** or **12a–12f**, see ESI† for details) with the various Michael reaction donors (**11** and **11a–11j**). Most of the reactions reached completion within 6 days and gave the desired products **13h–13m** and **13s–13v** in good yields with good or excellent enantioselectivities. Notably, the products (**13n**, **13r** and **13w**) were isolated in lower yields because the byproducts of the double-FCM additions increased as the reaction time was extended. When the *R*_2_ group was a 6-, 5- or 4-membered ring with a different cyclic tension, there were considerable effects on the reaction rates (**13j** took 6 days, **13u** took 4 days and **13w** took 18 h). Furthermore, in many cases, after the recrystallization of the corresponding phloroglucinol products, the *er* improved to 99 : 1–99.8 : 0.2. These polycyclic polymethylated phloroglucinol derivatives will be beneficial for the asymmetric synthesis of other diverse PPPs.^1^

### Asymmetric syntheses of myrtucommuacetalone (**2**) and myrtucommuacetalone B (**3**)

With compounds **9** 11 and (+)-**13** in hand, we proceeded to investigate our proposed syntheses of the natural myrtucommuacetalone (**2**) and myrtucommuacetalone B (**3**) (Scheme 1). We subsequently evaluated the proposed Michael-ketalization-annulation cascade sequence using (+)-**13** and **9** as substrates with a variety of catalysts (*i.e.*, TFA, *p*-TsOH, CSA, AcOH and some chiral phosphoric acids, as shown in Table 1) and solvents (*i.e.*, DCM, DCE, THF, DME, CHCl_3_ and PhMe). Rewardingly, we found that the treatment of a mixture of **13** and **9** with (*R*)-**C2** and TsOH (1 : 1.5) in PhMe at 60 °C resulted in the expected Michael-ketalization-annulation cascade reaction to give the desired diastereoisomers **3a** and **2a** with sterically compact hexacyclic skeletons in a combined yield of 70% (2.0 g) and a ratio of 11 : 1. However, it is worth mentioning that without the chiral acid (*R*)-**C2**, the treatment of **13** and **9** with *p*-TsOH (1.5 equiv.) in similar conditions resulted in the formation of **3a** and **2a** in a combined yield of 60% and a ratio of 4 : 1. These compounds were readily separated by recrystallization. The structure of **3a** was unambiguously confirmed by X-ray crystallography.

We envisaged that (+)-**13** might undergo a Michael (or FCM) addition to **9** in the presence of (*R*)-**C2** and *p*-TsOH to generate intermediate **B** (presumably from **A**). The subsequent intramolecular attack of the less hindered free phenolic hydroxyl group on the less hindered carbonyl group in **B** would then yield the hemiacetal **C**, whose trisubstituted alkene would be protonated to give intermediate **D**. The hydroxyl group of the hemiacetal in **D** would undergo the annulation to yield the ketalization products **3a** and **2a**. We reasoned that the sterically hindered isopropyl group at C13 in (+)-**13** was critical for this diastereo- and regioselective outcome. This route therefore provided facile access to a total of 2.13 g of **3a** and **2a** (see the ESI† for details), thereby highlighting the robust nature of this chemistry. Notably this new cascade reaction constructed five new chemical bonds, two new rings, and three stereogenic centers with high diastereoselectivity6*f* and regioselectivity in a single step.

The treatment of compounds **3a** and **2a** with KOH in EtOH/H_2_O at 80 °C gave (–)-myrtucommuacetalone B (**3**) and myrtucommuacetalone (**2**, proposed structure), respectively, in good yields. The ^1^H and ^13^C NMR spectra of **2** were identical to those of the natural product, however the sign of its optical rotation was the opposite of that reported in the literature {synthetic: [*α*]25D = +24 (*c* = 0.1, CHCl_3_); natural: [*α*]30D = –33 (*c* = 1.0, CHCl_3_)}.^3^ Notably, the absolute configuration of naturally occurring **2** has not been reported previously.^3^,6f The absolute configuration of naturally occurring myrtucommuacetalone was therefore determined to be 1*R*, 9*R*, 10*R*, 17*S* based on our total synthesis.

### Asymmetric syntheses of callistrilones A, C, D and E

**Scheme 2:**
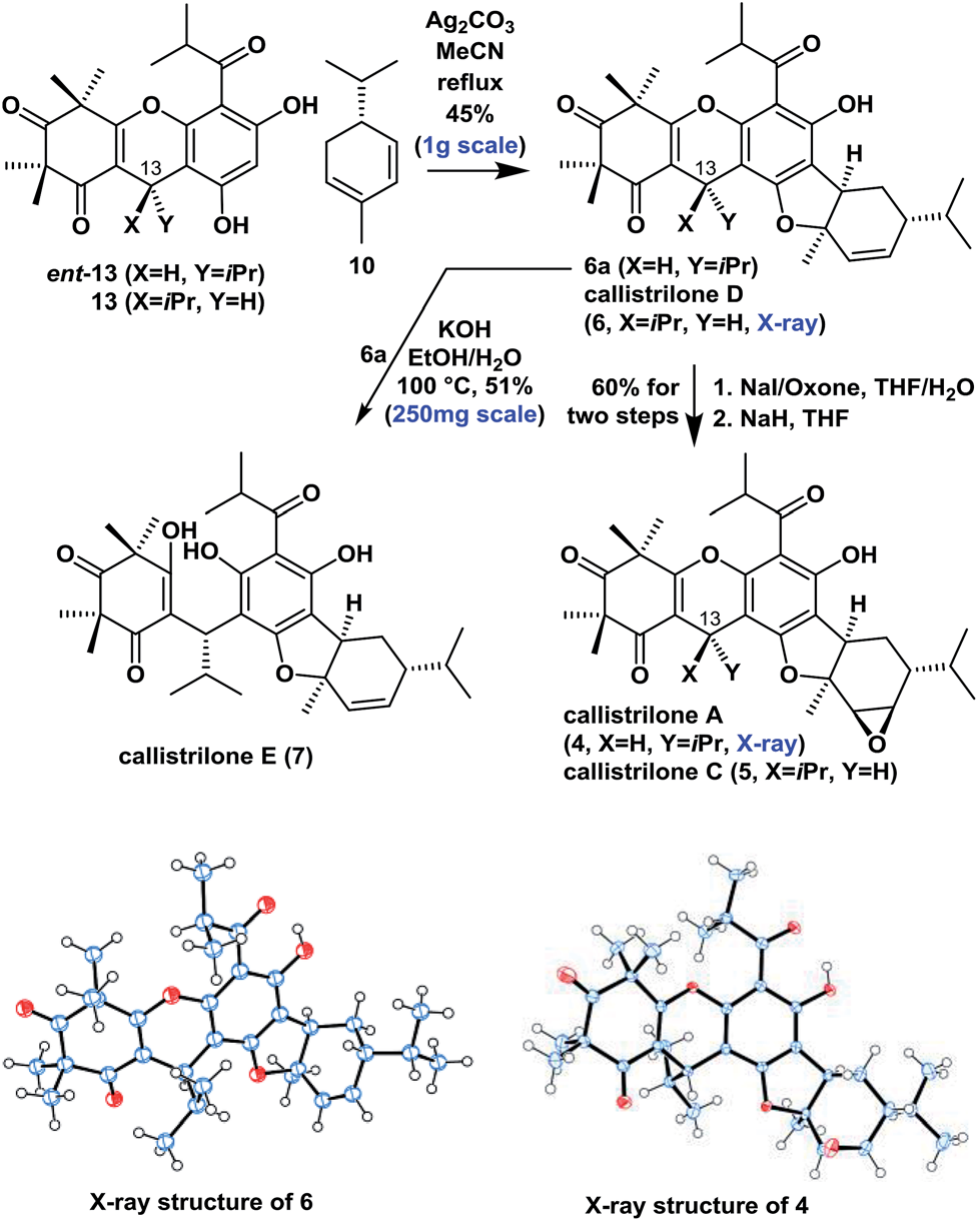
Asymmetric synthesis of PPPs **4–7**.

Moving forward, we proceeded with our proposed syntheses of the remaining natural PPPs **4–7** (Scheme 2). We initially investigated the intermolecular oxidative [3 + 2] cycloaddition of ***ent*-13** or **13** with **10** using various conditions from the literature that have previously been applied to achieve the construction of several related systems.^9^,7b Disappointingly, these conditions failed to afford the desired product in this particular case. Eventually however, the treatment of ***ent*-13** or **13** and **10** with Ag_2_CO_3_ 15 in refluxing MeCN afforded the angular product **6a** or callistrilone D (**6**) diastereo- and regioselectively as a single product in a 45% yield (1.0 g scale). Furthermore, compounds **6a** and **6** were treated with NaI/oxone for iodohydroxylation of the disubstituted double bond, followed by the diastereoselective cyclization with NaH as a base, to give both the desired callistrilone A (**4**) and callistrilone C (**5**) in a 60% overall yield. Notably, the expected direct epoxidation of compound **6a** or **6** with *meta*-chloroperoxybenzoic acid (*m*CPBA) or dimethyldioxirane (DMDO) failed to afford the desired product **4** or **5**. Pleasingly, the treatment of **6a** with KOH in EtOH/H_2_O gave callistrilone E (**7**). The structures of synthetic **6** and **4** were also confirmed by X-ray crystallography.

### Antibacterial activity assay

The emergence of multidrug-resistant bacteria has become a major threat to public health. It is therefore important to develop a better understanding of chemicals that show activity against drug-resistant bacterial strains such as methicillin-resistant *Staphylococcus aureus* (MRSA), vancomycin-intermediate *S. aureus* (VISA), and vancomycin-resistant *Enterococcus faecium* (VRE).^16^ A major focus in current antibiotic development is based on the screening of natural products.^17^ Therefore, the antibacterial activities of the compounds prepared in the current study were evaluated against six Gram-positive and five Gram-negative bacteria (Table 3). Among the compounds, **7** exhibited pronounced antibacterial activities against all Gram-positive bacteria, including three multidrug-resistant strains, with MIC values in the range of 0.25 to 2 μg mL^–1^. Notably, compound **7** exhibited greater antibacterial activity against multidrug-resistant strains (MRSA, VISA and VRE) than vancomycin, which is currently considered to be the last resort for treatment of Gram-positive bacterial infections. This compound therefore represents a promising lead compound for the development of antibacterial agents.

**Table 3:** Antibacterial activities of **2–7** and vancomycin against six Gram-positive bacterial strains (MIC, μg mL^–1^)

Compound	*S*. *aureus* ATCC 33591 (MRSA)	*S*. *aureus* ATCC 700699 (VISA)	*E*. *faecium* ATCC 700221 (VRE)	*S*. *aureus* ATCC 29213 (MSSA)	*E*. *faecalis* ATCC 29212	*S*. *epidermidis*
**2**	8	8	64	8	64	16
**3**	16	16	>128	16	>128	>128
**4**	32	32	64	32	64	>128
**5**	>128	>128	>128	>128	>128	>128
**6**	>128	>128	>128	>128	>128	>128
**7**	0.25	0.25	0.5	0.25	2	2
Vancomycin	2	8	>128	1	4	2

## Conclusions

We have developed a new approach for the highly concise, catalytic and first total asymmetric synthesis of myrtucommuacetalone, myrtucommuacetalone B and callistrilones A, C, D and E, in only 5–7 steps. Our route shows good step, redox and atom economy from simple building blocks (**9–12**) and avoids the need for protecting groups.^18^ This synthetic strategy was enabled by a unique organocatalytic asymmetric Friedel–Crafts-type Michael addition to synthesize **8** (95 : 5 *er*, after recrystallization of **13**: 99.5 : 0.5 *er*), a versatile biomimetic synthetic precursor for the construction of some other PPPs. Notably, a Michael-ketalization-annulation cascade reaction was established as the key step in the efficient formation of the difficult to construct bridged furochromene moiety, together with the polycyclic ketal skeleton of myrtucommuacetalone B, with high diastereoselectivity and regioselectivity. A diastereo- and regioselective oxidative [3 + 2] cycloaddition allowed for the facile construction of the unusual and sterically compact 6/6/6/5/6-fused pentacyclic skeleton of the callistrilones. Based on our total synthesis, the absolute configuration of myrtucommuacetalone (**2**) was determined. Notably, the new compound **7** exhibited considerable antibacterial activity against Gram-positive bacteria and showed greater antibacterial activity against multidrug-resistant strains (*i.e.*, MRSA, VISA and VRE) than that of vancomycin. This work will serve as a platform for the catalytic asymmetric synthesis of a diverse range of PPPs^1^ and for further systematic evaluation of their biological activities. These investigations are underway and will be reported in due course.

## Conflicts of interest

There are no conflicts to declare.

## Supplementary Material

Supplementary information

Crystal structure data
