# Supplementary material for: Catalytic asymmetric total syntheses of myrtucommuacetalone, myrtucommuacetalone B, and callistrilones A, C, D and E
Source: Chem Sci. 2017 Nov 27;9(6):1488–95. doi: 10.1039/c7sc04672c (PMC5875087; doi:10.1039/c7sc04672c)
Supplement: Supplementary file 1 [file SC-009-C7SC04672C-s001.pdf]

Electronic Supplementary Material (ESI) for Chemical Science.
This journal is © The Royal Society of Chemistry 2017

# Supporting Information

## Catalytic Asymmetric Total Syntheses of Myrtucommuacetalone, Myrtucommuacetalone B and Callistrilones A, C, D, E

Min-Jing Cheng,[†,‡] Jia-Qing Cao,[†] Xin-Yi Yang,[∥] Li-Ping Zhong,[‡] Li-Jun Hu,[†] Xi Lu,[∥] Bao-Long Hou,[‡] Ya-Jian Hu,[‡] Ying Wang,[†] Xue-Fu You,[∥] Lei Wang,*[,†] Wen-Cai Ye*[,†] and Chuang-Chuang Li*[,‡]

[†] College of Pharmacy, Jinan University, Guangzhou 510632, China.
E-mail: cpuwanglei@126.com; chyewc@gmail.com

[‡] Department of Chemistry, Southern University of Science and Technology, Shenzhen 518055, China. E-mail: ccli@sustc.edu.cn

[∥] Institute of Medicinal Biotechnology, Chinese Academy of Medical Sciences/Peking Union Medical College, Beijing 100050, China

**Table of Contents**

1. Experimental detail for isolation and structural elucidation of natural products........................S3

1.1 General experimental procedure................................................................................S3
1.2 Isolation of natural products.......................................................................................S4
1.3 Structural determination.............................................................................................S5
1.4 Physico-chemical data of natural products...............................................................S14
1.5 X-ray crystallographic study of natural products.....................................................S15
1.6 HRMS, UV, IR, NMR spectra of **3**, **5-7**..................................................................S17

2. Synthetic experimental procedures...............................................................................S35

2.1. General Information....................................................................................................S35
2.2 General procedure for the synthesis of **11** and **11a-11j**..........................................S36
2.3 General procedure for the synthesis of **12a** and **12aa-12af**...................................S41
2.4 General procedure for the synthesis of **12** and **12a-12f**..........................................S45
2.5 Table S1. Optimization of reaction conditions for the Friedel–Crafts type Michael (FCM) additions[a]...........................................................................................................S47
2. 6 Table S2. Substrate Scope of the Organocatalytic Enantioselective FCM additions[a,b] ....................................................................................................................................S49
2.7 Synthesis of **13**, ***ent*-13** and **13a-13w**......................................................................S50
2.8 Synthesis of **9**.............................................................................................................S70
2.9 Synthesis of **2a**, **3a**......................................................................................................S71
2.10 Synthesis of **6a**..........................................................................................................S77

3. X-ray crystal structures of **3a**, **4**, **6**............................................................................. S87

4. Synthetic $^{1}H$ and $^{13}C$ NMR Spectra.............................................................................S88

5. HPLC chromatogram of **13**, ***ent*-13**, **13a-13w**..........................................................S166

6. Antibacterial activity assay of **2-7**.................................................................................S193

6.1. Microorganisms.........................................................................................................S193
6.2 Antimicrobial agents and medium............................................................................S193
6.3 MIC determination......................................................................................................S193
6.4 In vitro antibacterial activities of 6 synthetic compounds...................................... S194

## 1. Experimental detail for isolation and structural elucidation of natural products

### 1.1 General experimental procedure

IR spectra (KBr disks, in $cm^{-1}$) were obtained using a Jasco FT/IR-480 Plus Fourier Transform spectrometer (Jasco, Tokyo, Japan). UV spectra were recorded on a Jasco V-550 UV/Vis spectrometer (Jasco, Tokyo, Japan). Optical rotations were measured on a Jasco P-2000 polarimeter (Jasco, Tokyo, Japan) with a 1 cm cell at room temperature. Melting points were obtained on an X-5 micro-melting point apparatus (Fukai Instrument, Beijing, China) without correction. X-ray crystallographic analysis was carried out on an Agilent Gemini S Ultra CCD diffractometer with Cu K$\alpha$ radiation ($\lambda$ = 1.54178 Å). CD spectra were obtained on a Jasco J-810 spectropolarimeter (Jasco, Tokyo, Japan) at room temperature. HR-ESI-MS spectra were detected using an Agilent 6210 LC/MSD TOF-MS spectrometer (Agilent Technologies, CA, USA). NMR spectra were measured with a Bruker AV-500 spectrometer (Bruker, Fällanden, Switzerland). Column chromatographies (CC) were performed on silica gel (300–400 mesh, Qingdao Marine Chemical Plant, China), ODS (Merck, Darmstadt, Germany) and Sephadex LH-20 (Pharmacia Uppsala, Sweden). HPLC were carried out using Agilent 1260 Series instrument equipped with 1260 series multiple wavelength detector, as well as Cosmosil 5C18-MS-II and chiral Phenomenex Lux cellulose (4.6 × 250 mm) columns. Preparative HPLC were carried out using Agilent 1260 Series instrument and Cosmosil 5C18-MS-II (250×20 mm; 250×10 mm) column. All solvents used in CC and HPLC were analytical (Tianjin Fuyu Fine Chemical Company, Tianjin, China) and chromatographic grade (Fisher Scientific, NJ, USA), respectively.

## 1.2 Isolation of natural products

*Myrtus communis* (Myrtaceae) is an evergreen sclerophyll shrub widely distributed over the Mediterranean region. It has been traditionally used in folk medicine as an antibacterial, anti-inflammatory, and analgesic agent. The leaves of *M. communis* were collected in Shanghai City of China, in August of 2014, and authenticated by Prof. Guang-Xiong Zhou (Jinan University). A voucher specimen (No. 2014082401) was deposited in the Institute of Traditional Chinese Medicine and Natural Products, College of Pharmacy, Jinan University, Guangzhou, P. R. China.

The air-dried and powdered leaves of *M. Communis* (8.0 kg) were percolated with 95% EtOH (24 h × 5) at room temperature. After filtration, the extract was evaporated under reduced pressure to give 1.2 kg crude extract, which was then suspended in $H_2O$ and extracted with petroleum ether (PE, b.p. 60–90 °C). The PE solution was concentrated to give a residue (389 g), which was subjected to a silica gel column eluted with a gradient mixture of petroleum ether/EtOAc (100:0 → 0:100). Ten fractions (Frs. A−J) were collected. Fr. C (25.0 g) was subjected to ODS column using $CH_3OH$-$H_2O$ (70:30 → 100:0) as eluent to yield nine subfractions (Frs. C1–C9). The subfraction C4 was purified by reversed-phase preparative HPLC (flow rate: 6 mL/min; detection wavelength: 280 nm; mobile phase: 85% $CH_3CN$-$H_2O$) to afford **3** (11.3 mg, $t_R$ 55.3 min).

*Callistemon rigidus* (Myrtaceae) is an evergreen shrub distributed in the tropics, which has been traditionally used as an herbal medicine in the treatment of eczema and cold. The leaves of *C. rigidus* were collected from Guangzhou city, Guangdong Province of China in July 2014, and authenticated by Prof. Guang-Xiong Zhou (Jinan University, Guangzhou, China). A voucher specimen (No. 2014072801) was deposited in the Institute of Traditional Chinese Medicine and Natural Products, Jinan University, Guangzhou, China.

The air-dried leaves of *C. rigidus* (10 kg) were percolated with 95% EtOH (V/V, 50 L) for three times at room temperature. The crude extract (1.05 kg) was suspended in water and then extracted with PE (b.p. 60–90 °C). The PE extract (236.5 g) was subjected to silica gel column eluted with a gradient mixture of cyclohexane/EtOAc (100:0 → 0:100) to afford thirteen fractions (Fr. A–M). Fr. D (36.6 g) was separated by silica gel column with PE/EtOAc (100:0 → 90:10)

as eluent to yield seven subfractions (Fr. D1–D7). Then, subfraction D3 (5.7 g) was subjected to ODS column using MeOH/$H_2O$ (70:30 → 100:0) as eluent and further purified by reversed-phase preparative HPLC [column: Phenomenex Luna 5u PFP(2) 250×21.2 mm; flow rate: 8 mL/min; detection wavelength: 280 nm; mobile phase: 80% $CH_3CN/H_2O$, 0.05% TFA] to afford compound **5** (10.6 mg, $t_R$ 68.3 min). Fr. E (54.6 g) was separated by silica gel column with PE-EtOAc (100:0 → 90:10) as eluent to yield six subfractions (Frs. E1–E6). Subfraction Fr. E4 (7.9 g) was subjected to ODS column using MeOH-$H_2O$ (60:40 → 100:0) as eluent to yield subfractions E4a–E4d. The subfraction E4a (0.4 g) was subjected to Sephadex LH-20 ($CH_3OH$) and further purified by reversed-phase semi-preparative HPLC (column: Cosmosil 5C18-MS-II 250×10 mm; flow rate: 3 mL/min; detection wavelength: 280 nm; mobile phase: 85% $CH_3CN$-$H_2O$) to afford compound **6** (10.9 mg, $t_R$ 70.8 min). The subfraction E4b (2.5 g) was recrystallized from methanol and further separated by HPLC [column: Phenomenex Luna 5u PFP(2) 250×4.6 mm, flow rate: 1 mL/min; detection wavelength: 280 nm; mobile phase: 80% $CH_3CN$ (0.1% HCOOH)-$H_2O$(0.1% HCOOH)] to afford compound **7** (5.5 mg, $t_R$ 54.5 min).

**1.3 Structural determination**

The molecular formula of myrtucommuacetalone B (**3**) was established as $C_{38}H_{52}O_9$ based on its HRESIMS data (*m/z* 653.3688 $[M+H]^+$, calcd for $C_{38}H_{53}O_9$: 653.3684). The optical activity and Cotton effects of **3** were undetectable, which suggested that **3** could be a racemate. The IR spectrum suggested the presence of aromatic (1592 and 1470 $cm^{-1}$), hydroxy (3202 $cm^{-1}$), and carbonyl groups (1707 $cm^{-1}$). Although this compound was homogeneous on RP HPLC and TLC, both $^1H$ and $^{13}C$ NMR spectra of **3** displayed two sets of signals in a ratio of approximately 5:4, which suggested this compound exists as a pair of rotamers.[1] Detailed examination of 1D and 2D NMR spectra of **3** and comparison with known compound myrtucommuacetalone (**2**) [2] revealed their chemical shifts were similar, except for the differences of C-17, C-5 and C-2' were observed, indicating **3** was a C-17 epimer of **2**. Fortunately, crystals suitable for single-crystal X-ray diffraction were obtained. The intramolecular hydrogen-bondings between 7-OH and C-2' carbonyl, and between 6'-OH and 5-oxygen atom (Figure S1-1) made **3** exist in two rotamers (**3A** and **3B**).[1] Based on the analysis of $^1H$-$^1H$ COSY, HSQC, HMBC and NOESY spectra (Figure S1-2), the $^1H$ and $^{13}C$ NMR data of **3** were assigned as shown in Table S1-1.

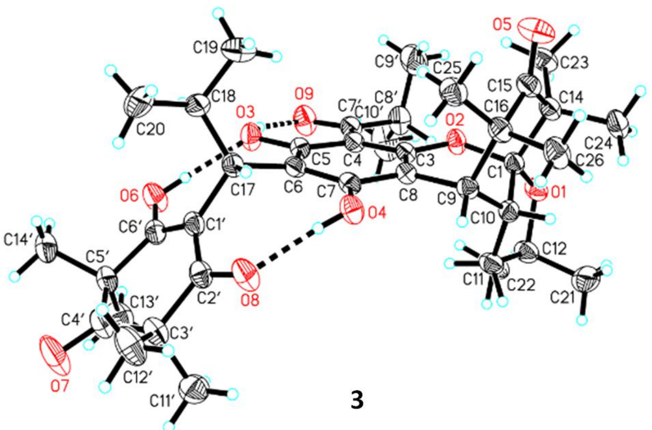

**3**

**Figure S1-1.** X-ray ORTEP drawing of myrtucommuacetalone B (**3**)

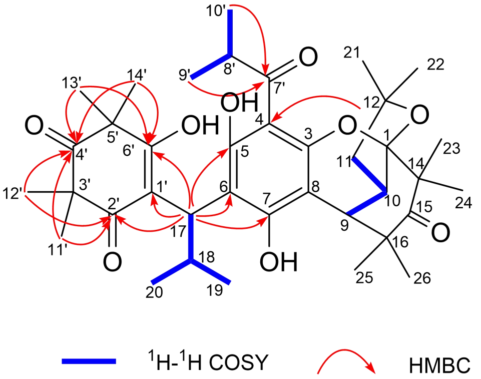


**Figure S1-2.** Key $^1$H-$^1$H COSY and HMBC correlations of **3**

**Table S1-1.** $^1$H (500 MHz) and $^{13}$C (125 MHz) NMR data of myrtucommuacetalone B (**3**) in $CDCl_3$ ($\delta$ in ppm, $J$ in Hz) $^{a,b}$

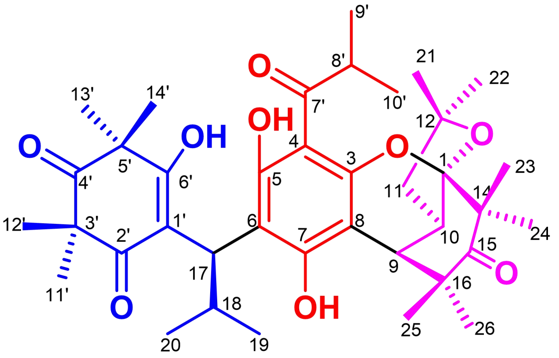

**myrtucommuacetalone B (3)**

| position | $\delta_H$ **(3A)** | $\delta_C$ **(3A)** | positio | $\delta_H$ **(3B)** | $\delta_C$ **(3B)** |
|---|---|---|---|---|---|
| 1 | – | 114.3 | 1 | – | 114.1 |
| 3 | – | 162.1 | 3 | – | 162.5 |
| 4 | – | 102.8 | 4 | – | 102.5 |
| 5 | 16.63* | 162.9 | 5 | 17.01* | 163.1 |
| 6 | – | 108.8 | 6 | – | 108.3 |
| 7 | 11.54* | 154.3 | 7 | 10.80* | 154.5 |
| 8 | – | 103.6 | 8 | – | 103.2 |
| 9 | 3.42 d (3.0) | 38.1 | 9 | 3.43 d (3.0) | 38.0 |

| | | | | | |
|---|---|---|---|---|---|
| 10 | 3.38 m | 35.4 | 10 | 3.38 m | 35.1 |
| 11 | 1.89 dd (12.2, 7.4) | 39.4 | 11 | 1.89 dd (12.2, 7.4) | 39.4 |
| | 1.44 m | – | | 1.44 m | – |
| 12 | – | 85.2 | 12 | – | 85.2 |
| 14 | – | 54.1 | 14 | – | 54.1 |
| 15 | – | 217.8 | 15 | – | 218.0 |
| 16 | – | 50.1 | 16 | – | 50.4 |
| 17 | 3.73 d (3.5) | 39.5 | 17 | 3.67 d (3.5) | 41.4 |
| 18 | 3.02 m | 26.3 | 18 | 3.02 m | 26.2 |
| 19 | 0.67 d (6.6) | 22.2 | 19 | 0.67 d (6.6) | 22.2 |
| 20 | 0.81 d (6.6) | 22.1 | 20 | 0.85 d (6.6) | 22.2 |
| 21 | 1.29 s | 30.4 | 21 | 1.29 s | 30.4 |
| 22 | 1.32 s | 28.8 | 22 | 1.32 s | 28.8 |
| 23 | 1.28 s | 19.8 | 23 | 1.30 s | 19.8 |
| 24 | 1.27 s | 24.7 | 24 | 1.27 s | 24.7 |
| 25 | 0.86 s | 23.4 | 25 | 0.98 s | 23.7 |
| 26 | 1.37 s | 29.2 | 26 | 1.38 s | 29.2 |
| 1' | – | 114.1 | 1' | – | 114.3 |
| 2' | – | 203.3 | 2' | – | 204.0 |
| 3' | – | 55.0 | 3' | – | 54.6 |
| 4' | – | 212.5 | 4' | – | 213.0 |
| 5' | – | 49.0 | 5' | – | 49.3 |
| 6' | 10.37* | 178.9 | 6' | 11.42* | 177.0 |
| 7' | – | 211.7 | 7' | – | 212.2 |
| 8' | 4.04 septet (6.8) | 37.9 | 8' | 4.03 septet (6.8) | 37.9 |
| 9' | 1.15 d (6.7) | 19.6 | 9' | 1.12 d (6.7) | 21.0 |
| 10' | 1.13 d (6.7) | 19.2 | 10' | 1.11 d (6.7) | 20.6 |
| 11' | 1.25 s | 24.8 | 11' | 1.31 s | 23.9 |
| 12' | 1.37 s | 25.4 | 12' | 1.36 s | 26.9 |
| 13' | 1.31 s | 26.7 | 13' | 1.48 s | 25.2 |
| 14' | 1.45 s | 24.9 | 14' | 1.36 s | 25.6 |

[a)] Overlapped signals are reported without designating multiplicity. [b)] *OH

Callistrilone C (**5**) was obtained as colorless gum with $[\alpha]^{26}_{D}$ = + 79.5° (*c* 0.20, $CH_3OH$). The HRESIMS of **5** showed a quasimolecular ion peak at *m/z* 565.3162 $[M+H]^+$ (calcd for $C_{34}H_{45}O_7$ 565.3160), consistent with the molecular formula $C_{34}H_{44}O_7$. The $^1H$ and $^{13}C$ NMR data of **5** were almost identical with the known compound callistrilone A (**4**),[3] except for some slight differences at H-13, H-14' and H-15' were observed, suggesting that **5** could be the C-13 epimer of **4**. The planar structure of **5** was further confirmed by the HMBC and $^1H$–$^1H$ COSY correlations (Figure S1-3). The NOESY cross peaks between H-7b and H-9'/12', between H-14' and H-2'/4', as well as the lack of NOE correlations between H-12' and H-14'/H-15' suggested the relative configurations of **5** (Figure S1-4).[3] Finally, the structure with absolute configurations was deduced by our total synthesis.

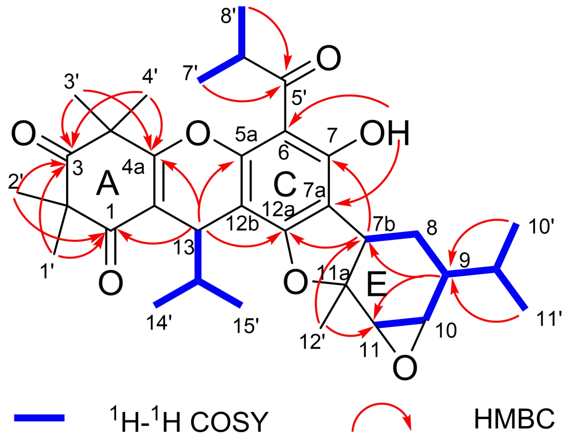


**Figure S1-3.** Key $^1$H-$^1$H COSY and HMBC correlations of **5**

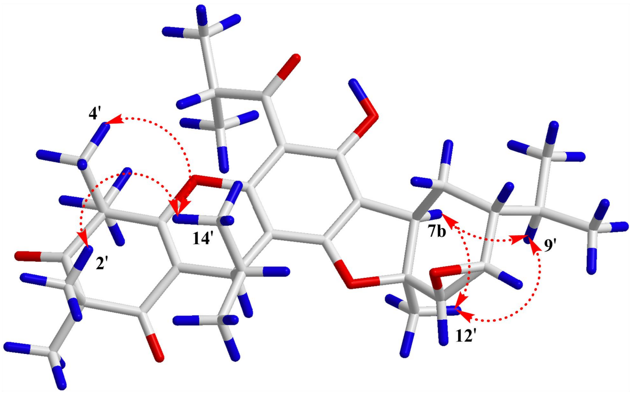


**Figure S1-4.** Key NOESY correlations of **5**

**Table S1-2.** $^1$H (500 MHz) and $^{13}$C (125 MHz) NMR Data of **5** in CDCl$_3$ ($\delta$ in ppm, $J$ in Hz) [a,b]

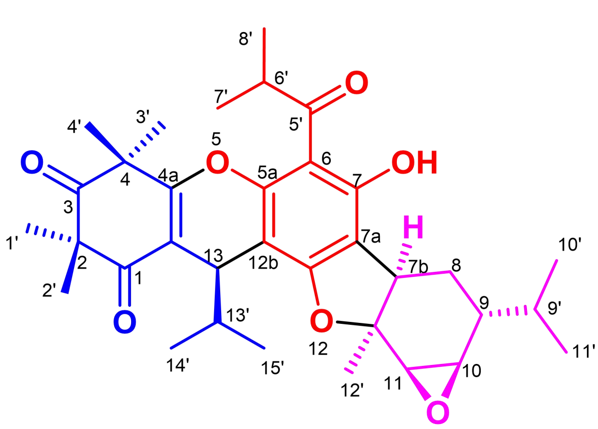


callistrilone C (**5**)

| position | $\delta_H$ | $\delta_C$ | position | $\delta_H$ | $\delta_C$ |
|---|---|---|---|---|---|

| | | | | | |
|---|---|---|---|---|---|
| 1 | – | 197.8 | 13 | 4.11 d (3.5) | 32.7 |
| 2 | – | 56.4 | 13a | – | 112.5 |
| 3 | – | 212.2 | 1' | 1.36 s | 24.3 |
| 4 | – | 47.5 | 2' | 1.40 s | 25.3 |
| 4a | – | 167.5 | 3' | 1.40 s | 24.9 |
| 5a | – | 153.6 | 4' | 1.58 s | 25.2 |
| 6 | – | 104.2 | 5' | – | 209.3 |
| 7 | 13.30* | 160.4 | 6' | 3.87 m | 39.7 |
| 7a | – | 113.5 | 7' | 1.21 d (7.0) | 18.0 |
| 7b | 3.02 dd (12.4, 5.9) | 40.7 | 8' | 1.21 d (6.9) | 21.2 |
| 8 | 2.10 ddd (14.1, 5.9, 2.7) | 23.8 | 9' | 1.63 m | 28.6 |
| | 1.67 m | – | 10' | 1.08 d (6.5) | 22.1 |
| 9 | 1.82 m | 39.3 | 11' | 1.08 d (6.5) | 21.5 |
| 10 | 3.42 dd (4.2, 3.3) | 56.0 | 12' | 1.45 s | 26.3 |
| 11 | 3.26 dd (4.2) | 55.1 | 13' | 1.89 m | 34.9 |
| 11a | – | 88.4 | 14' | 0.71 d (6.9) | 18.2 |
| 12a | – | 162.2 | 15' | 0.90 d (6.9) | 19.7 |
| 12b | – | 99.2 | | | |

[a)] Overlapped signals are reported without designating multiplicity. [b)] *OH

Callistrilone D (**6**) was obtained as amorphous powder with $[\alpha]^{26}_{D}$ = +63.5° (*c* 0.10, $CH_3OH$). The molecular formula of **6** was established as $C_{34}H_{44}O_6$ by the quasi-molecular ion at *m/z* 549.3227 $[M+H]^+$ in its HRESIMS (calcd for $C_{34}H_{45}O_6$: 549.3211). The IR spectrum showed characteristic bands for aromatic ring (1621, 1460 $cm^{-1}$) and carbonyl group (1718 $cm^{-1}$). Comparison of the NMR data of **6** with those of **5** suggested that they possessed the same framework, except the signals for epoxy carbons in **5** were replaced by olefinic carbons in **6.** The HMBC correlations between H-13 and C-1/C-4a/C-5a, between OH-7 ($\delta_H$ 13.39) and C-6/C-7a, as well as between H-12' and C-7b/C-11 (Figure S1-5) further confirmed its planar structure. A comprehensive analysis of the $^1H$–$^1H$ COSY, HSQC, HMBC, and NOESY spectra (Figure S1-5 and S1-6) allowed the assignment of NMR data of **6** as shown in Table S1-3. The unambiguous structural assignments and stereochemistry of **6** could be elucidated by successful total synthesis.

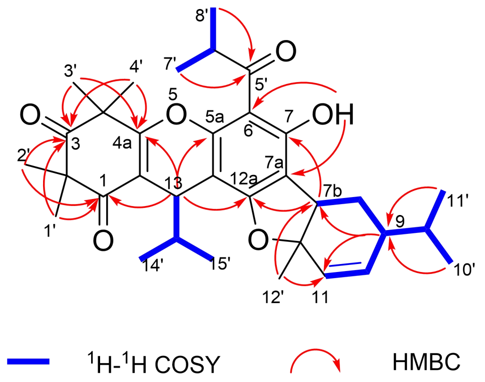


**Figure S1-5.** Key $^1$H-$^1$H COSY and HMBC correlations of **6**

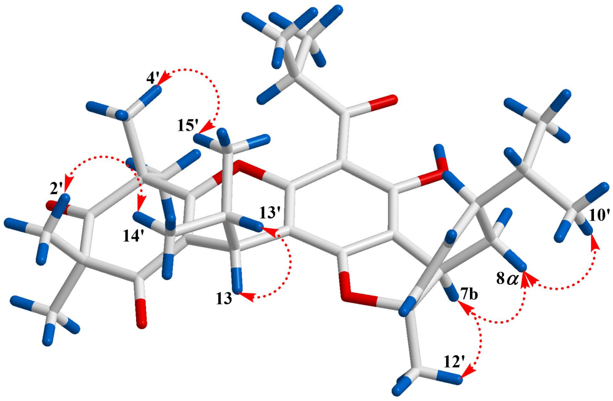

**Figure S1-6.** Key NOESY correlations of **6**

**Table S1-3.** $^1$H (500 MHz) and $^{13}$C (125 MHz) NMR data of callistrilone D (**6**) in $CDCl_3$ ($\delta$ in ppm, $J$ in Hz) [a,,b]

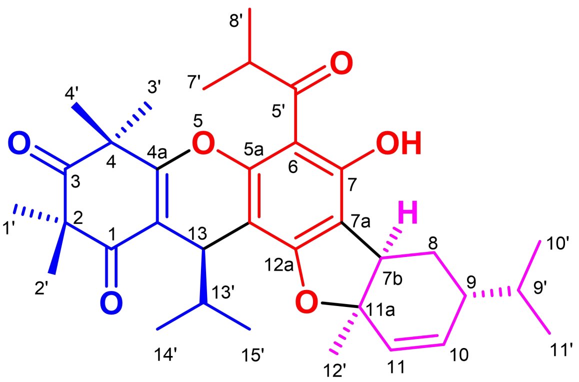

callistrilone D (**6**)

| position | $\delta_H$ | $\delta_C$ | position | $\delta_H$ | $\delta_C$ |
|---|---|---|---|---|---|
| 1 | – | 197.6 | 13 | 4.05 d (3.6) | 32.4 |
| 2 | – | 56.3 | 13a | – | 112.4 |

| | | | | | |
|---|---|---|---|---|---|
| 3 | – | 212.3 | 1' | 1.35 s | 24.7 |
| 4 | – | 47.5 | 2' | 1.39 s | 24.5 |
| 4a | – | 167.7 | 3' | 1.39 s | 25.3 |
| 5a | – | 154.0 | 4' | 1.56 s | 25.0 |
| 6 | – | 103.9 | 5' | – | 209.3 |
| 7 | 13.39* | 161.2 | 6' | 3.86 m | 39.7 |
| 7a | – | 112.7 | 7' | 1.22 d (7.0) | 18.1 |
| 7b | 3.48 dd (7.0, 4.5) | 45.1 | 8' | 1.22 d (7.0) | 21.3 |
| 8 | 2.42 m | 25.8 | 9' | 1.61 m | 31.7 |
| | 1.64 m | – | 10' | 0.91 d (6.7) | 19.8 |
| 9 | 1.99 m | 38.1 | 11' | 0.91 d (6.7) | 20.0 |
| 10 | 5.85 dd (10.3, 3.3) | 135.3 | 12' | 1.54 s | 26.5 |
| 11 | 5.58 dd (10.3, 2.0) | 129.2 | 13' | 1.80 m | 34.8 |
| 11a | – | 89.9 | 14' | 0.80 d (6.9) | 20.0 |
| 12a | – | 163.3 | 15' | 0.68 d (6.9) | 18.2 |

[a)] Overlapped signals are reported without designating multiplicity. [b)] *OH

The molecular formula of callistrilone E (**7**) was deduced as $C_{34}H_{46}O_7$ by the quasi-molecular ion at *m/z* 567.3328 $[M + H]^+$ in its HR-ESI-MS (calcd for $C_{34}H_{47}O_7$: 567.3316). The IR spectrum showed characteristic bands for hydroxyl group (3188 $cm^{-1}$), aromatic ring (1585, 1471 $cm^{-1}$), and carbonyl group (1720 $cm^{-1}$). Although this compound showed single peak on RP HPLC, both $^1H$ and $^{13}C$ NMR spectra of the compound displayed a pair of signals in a ratio of approximately 5:4, which suggested that compound **7** was an equilibrium mixture of two rotamers (**7A** and **7B**) caused by hydrogen-bonding.[1] Based on the analysis of $^1H$–$^1H$ COSY, HSQC, HMBC and NOESY spectra, the $^1H$ and $^{13}C$ NMR data of **7A** and **7B** were assigned as shown in Table S1-4. Comprehensive analysis of the NMR data of **7A** indicated that it shared the same framework as callistrilone A (**4**),[3] except the signals for epoxy carbons in **4** were replaced by olefinic carbons as well as the presence of two additional hydroxyl signals ($\delta_H$ 9.73, 10.21) in **7A**. The HMBC correlations between OH-4a and C-4/C-13a, between OH-5a and C-6/C-12a, between H-9 and C-7b/C-11, and between H-12' and C-7b/C-11 (Figure S1-7) confirmed its planar structure. The NOESY correlations between H-12' and H-7b, between H-8α and H-7b/H-9' indicate that the relative configurations of C-7b, C-9, and C-12' are identical to those of callistrilone A. Similarly, the structure with relative configuration of **7B** was assigned to be identical to that of **7A** by combined analysis of their 1D and 2D NMR data. Furthermore, the structure of **7** was confirmed by X-ray crystallographic experiments (Figure S1-8) and our successful total synthesis. Due to the

intramolecular hydrogen bonds between OH-5a and C-1 carbonyl, and between OH-4a and the ether oxygen atom, the rotation of C-13a–C-13 and C-13–C-12b bonds of **7** were blocked, which led to the formation of rotamers **7A** and **7B**. [1]

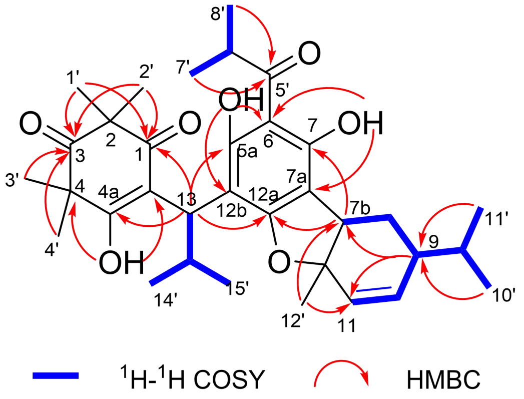


**Figure S1-7.** Key $^1$H-$^1$H COSY and HMBC correlations of **7**

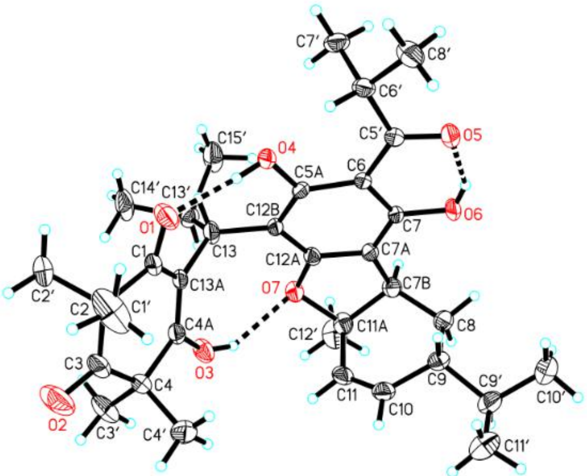

**Figure S1-8**. X-ray ORTEP drawing of callistrilone E **(7)**

**Table S1-4.** $^1$H (500 MHz) and $^{13}$C (125 MHz) NMR data of **7** in $CDCl_3$ ($\delta$ in ppm, $J$ in Hz) [a,b]

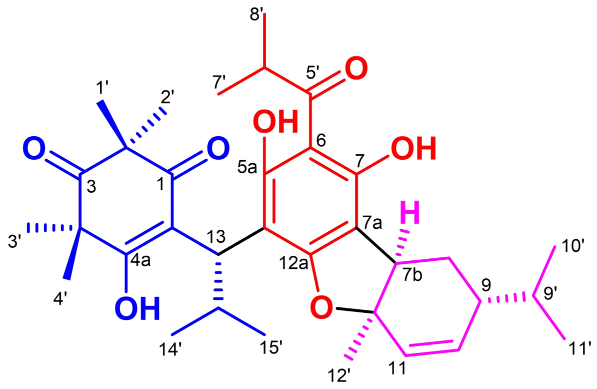


callistrilone E (**7**)

| position | $\delta_H$ (**7A**) | $\delta_C$ (**7A**) | position | $\delta_H$ (**7B**) | $\delta_C$ (**7B**) |
|---|---|---|---|---|---|
| 1 | – | 202.9 | 1 | – | 204.0 |
| 2 | – | 55.3 | 2 | – | 54.6 |
| 3 | – | 212.4 | 3 | – | 212.6 |
| 4 | – | 49.0 | 4 | – | 48.6 |
| 4a | 9.73* | 174.9 | 4a | 9.25* | 176.2 |
| 5a | 10.21* | 160.0 | 5a | 11.17* | 160.1 |
| 6 | – | 107.3 | 6 | – | 107.0 |
| 7 | 13.80* | 160.4 | 7 | 13.77* | 161.3 |
| 7a | – | 105.9 | 7a | – | 106.9 |
| 7b | 3.45 dd (5.8, 4.5) | 44.1 | 7b | 3.55 dd (5.8, 4.5) | 45.4 |
| 8 | 2.40 m | 26.0 | 8 | 2.55 m | 25.5 |
| | 1.67 m | – | | 1.56 m | – |
| 9 | 1.98 m | 38.0 | 9 | 1.93 m | 37.6 |
| 10 | 5.94 dd (10.3, 2.2) | 137.0 | 10 | 5.80 dd (10.3, 2.2) | 137.0 |
| 11 | 5.62 dd (10.2, 2.0) | 128.1 | 11 | 5.50 dd (10.2, 2.0) | 128.0 |
| 11a | – | 91.3 | 11a | – | 91.2 |
| 12a | – | 161.5 | 12a | – | 161.9 |
| 12b | – | 103.4 | 12b | – | 103.6 |
| 13 | 3.72 d (11.5) | 40.3 | 13 | 3.32 d (11.5) | 41.1 |
| 13a | – | 114.8 | 13a | – | 115.0 |
| 1' | 1.40 s | 27.3 | 1' | 1.37 s | 26.5 |
| 2' | 1.31 s | 23.8 | 2' | 1.31 s | 22.7 |
| 3' | 1.49 s | 24.8 | 3' | 1.48 s | 24.7 |
| 4' | 1.32 s | 25.6 | 4' | 1.34 s | 25.8 |
| 5' | – | 212.4 | 5' | – | 212.4 |
| 6' | 4.04 m | 39.7 | 6' | 4.04 m | 39.7 |
| 7' | 1.17 d (6.9) | 19.9 | 7' | 1.17 d (6.9) | 20.3 |
| 8' | 1.13 d (6.9) | 18.9 | 8' | 1.13 d (6.9) | 19.4 |
| 9' | 1.59 m | 31.5 | 9' | 1.59 m | 31.5 |
| 10' | 0.89 d (6.5) | 19.8 | 10' | 0.89 d (6.5) | 19.6 |
| 11' | 0.86 d (6.5) | 19.7 | 11' | 0.84 d (6.5) | 19.5 |
| 12' | 1.64 s | 26.2 | 12' | 1.58 s | 26.1 |

| | | | | | |
|---|---|---|---|---|---|
| 13' | 2.85 m | 27.1 | 13' | 2.97 m | 25.8 |
| 14' | 0.86 d (6.9) | 19.7 | 14' | 0.82 d (6.9) | 22.1 |
| 15' | 0.73 d (6.9) | 22.0 | 15' | 0.79 d (6.9) | 22.0 |

[a] Overlapped signals are reported without designating multiplicity. [b] *OH

**References**

[1] W.-Y. Tsui, G. D. Brown, *Tetrahedron.* 1996, **52**, 9735.

[2] M. I. Choudhary, N. Khan, M. Ahmad, S. Yousuf, H. K. Fun, S. Soomro, M. Asif, M. A. Mesaik, F. Shaheen, *Org. Lett.* 2013, **15**, 1862.

[3] J. Q. Cao, X. J. Huang, Y. T. Li, Y. Wang, L. Wang, R. W. Jiang, W. C. Ye, *Org. Lett.* 2016, **18**, 120.

**1.4 Physico-chemical data of natural products**

Myrtucommuacetalone B (**3**): colorless crystals; m. p. 224-226 °C; $[\alpha]^{26}_{D}$ = 0° (*c* 0.10, $CH_3OH$); UV ($CH_3OH$) $\lambda_{max}$ (log $\varepsilon$) 242 (4.48), 299 (4.50) nm; IR (KBr) $\nu_{max}$ 3202, 2975, 2870, 1707, 1592, 1470, 1383, 1300, 1245, 1134 $cm^{-1}$; HR-ESI-MS *m/z* 653.3688 $[M+H]^+$ (calcd for $C_{38}H_{53}O_9$: 653.3684).

Callistrilone C (**5**): colorless gum; $[\alpha]^{26}_{D}$ = +79.5° (*c* 0.20 $CH_3OH$); UV ($CH_3OH$) $\lambda_{max}$ (log $\varepsilon$) 206 (4.44), 234 (4.42), 298 (4.44) nm; IR (KBr) $\nu_{max}$ 3447, 2961, 2872, 1660, 1624, 1461, 1385, 1244, 1160 $cm^{-1}$; HR-ESI-MS *m/z* 565.3162 $[M+H]^+$ (calcd for $C_{34}H_{45}O_7$: 565.3160).

Callistrilone D (**6**): amorphous powder; $[\alpha]^{26}_{D}$ = +63.5° (*c* 0.10 $CH_3OH$); UV ($CH_3OH$) $\lambda_{max}$ (log $\varepsilon$) 206 (4.05), 218 (4.04), 308 (3.98) nm; IR (KBr) $\nu_{max}$ 3371, 2969, 2871, 1652, 1621, 1460, 1381, 1248, 1158 $cm^{-1}$; HR-ESI-MS *m/z* 549.3227 $[M+H]^+$ (calcd for $C_{34}H_{45}O_6$: 549.3211).

Callistrilone E (**7**): colorless crystals; m. p. 139-141 °C; $[\alpha]^{26}_{D}$ = -57.6° (*c* 0.10 $CH_3OH$); UV ($CH_3OH$) $\lambda_{max}$ (log $\varepsilon$) 206 (4.34), 232 (4.32), 298 (4.34) nm; IR (KBr) $\nu_{max}$ 3188, 2973, 2871, 1720, 1624, 1585, 1471, 1424, 1381, 1231, 1058 $cm^{-1}$; HR-ESI-MS *m/z* 567.3328 $[M+H]^+$ (calcd for $C_{34}H_{47}O_7$: 567.3316).

## 1.5 X-ray crystallographic study of natural products

Crytallographic data for myrtucommuacetalone B (**3**) have been deposited with the Cambridge Crystallographic Data Centre as supplementary publication no. CCDC 1526145. Copies of the data can be obtained, free of charge, on application to the Director, CCDC, 12 Union Road, Cambridge CB2 IEZ, UK (fax: +44-(0)1223-336033 or email: deposit@ccdc.cam.ac.uk).

**Table S1-5** Crystal data and structure refinement for myrtucommuacetalone B (**3**)

| | |
|---|---|
| Empirical formula | $C_{39}H_{53}Cl_3O_9$ |
| Formula weight | 772.16 |
| Temperature | 293(2) |
| Wavelength | 1.54184 Å |
| Crystal system, space group | triclinic, P-1 |
| Unit cell dimensions | a = 12.7541(5) A, alpha = 94.892(4) deg. |
| | b = 13.5008(6) A, beta = 105.455(4) deg. |
| | c = 13.6961(6) A, gamma = 113.791(4) deg. |
| Volume | 2029.59(15) A^3 |
| Z, Calculated density | 2, 1.264 Mg/m^3 |
| Absorption coefficient | 2.463 mm^-1 |
| F(000) | 820.0 |
| Crystal size | 0.28 x 0.24 x 0.20 mm |
| Theta range for data collection | 6.862 to 125.498 deg. |
| Limiting indices | −14<=h<=13, −11<=k<=15, −15<=l<=15 |
| Reflections collected / unique | 17119 / 6474 [R(int) = 0.0270] |
| Max. and min. transmission | 1.00000 and 0.64058 |
| Data / restraints / parameters | 6474/0/507 |
| Goodness-of-fit on F^2 | 1.041 |
| Final R indices [I>2sigma(I)] | $R_1$ = 0.0914, $wR_2$ = 0.2714 |
| R indices (all data) | $R_1$ = 0.0995, $wR_2$ = 0.2842 |
| Largest diff. peak and hole | 0.84 and −0.89 e.A^-3 |

Crytallographic data for **7** have been deposited with the Cambridge Crystallographic Data Centre as supplementary publication no. CCDC 1526146. Copies of the data can be obtained, free of charge, on application to the Director, CCDC, 12 Union Road, Cambridge CB2 IEZ, UK (fax: +44-(0)1223-336033 or email: deposit@ccdc.cam.ac.uk).

**Table S1-6.** Crystal data and structure refinement for callistrilone E (**7**)

| | |
|---|---|
| Empirical formula | $C_{34}H_{46}O_7$ |
| Formula weight | 566.71 |
| Temperature | 173.00(10) |
| Wavelength | 1.54184 A |
| Crystal system, space group | orthorhombic, $P2_12_12_1$ |
| Unit cell dimensions | a = 12.4836(2) A, alpha = 90 deg. |
| | b = 12.5791(2) A, beta = 90 deg. |
| | c = 20.1204(3) A, gamma = 90 deg. |
| Volume | 3159.58(9) A^3 |
| Z, Calculated density | 4, 1.1913 Mg/m^3 |
| Absorption coefficient | 0.660 mm^-1 |
| F(000) | 1224.0 |
| Crystal size | 0.43 x 0.33 x 0.21 mm |
| Theta range for data collection | 8.29 to 125.626 deg. |
| Limiting indices | −14<=h<=14, −14<=k<=14, −21<=l<=23 |
| Reflections collected / unique | 50336 / 5062 [R(int) = 0.0485] |
| Max. and min. transmission | 1.00000 and 0.74933 |
| Data / restraints / parameters | 5062/0/391 |
| Goodness-of-fit on F^2 | 1.043 |
| Final R indices [I>2sigma(I)] | $R_1$ = 0.0456, $wR_2$ = 0.1213 |
| R indices (all data) | $R_1$ = 0.0464 $wR_2$ = 0.1222 |
| Largest diff. peak and hole | 0.49 and −0.37 e.A^-3 |
| Flack parameter | −0.10 (10) |

## 1.6 HRMS, UV, IR, NMR spectra of 3, 5-7

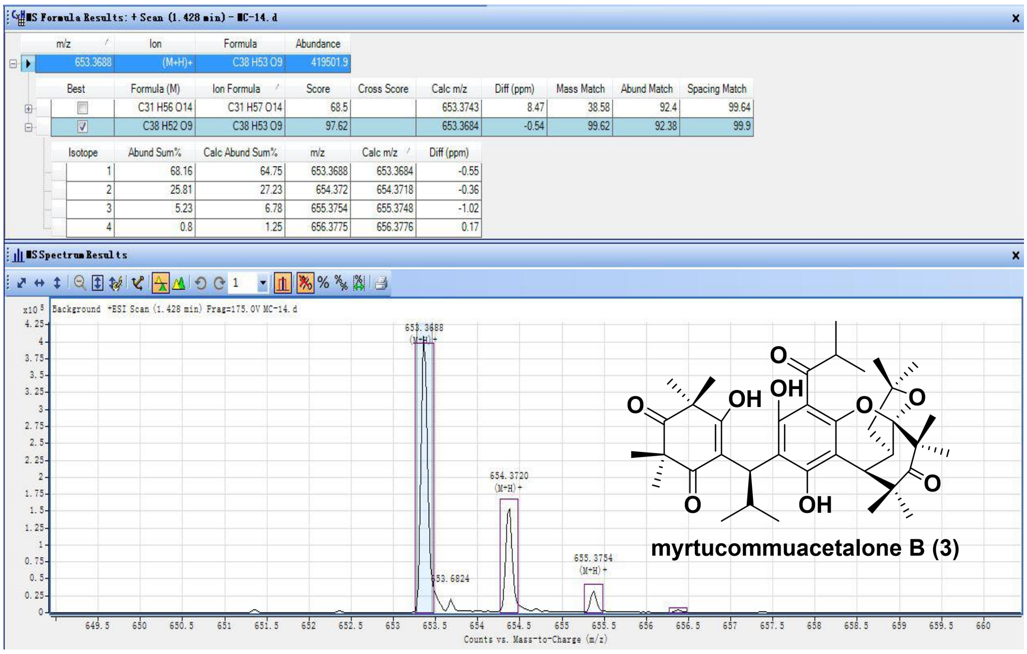

| m/z | Ion | Formula | Abundance |
|---|---|---|---|
| 653.3688 | (M+H)+ | C38 H53 O9 | 419501.9 |

| Best | Formula (M) | Ion Formula | Score | Cross Score | Calc m/z | Diff (ppm) | Mass Match | Abund Match | Spacing Match |
|---|---|---|---|---|---|---|---|---|---|
| ☐ | C31 H56 O14 | C31 H57 O14 | 68.5 | | 653.3743 | 8.47 | 38.58 | 92.4 | 99.64 |
| ☑ | C38 H52 O9 | C38 H53 O9 | 97.62 | | 653.3684 | -0.54 | 99.62 | 92.38 | 99.9 |

| Isotope | Abund Sum% | Calc Abund Sum% | m/z | Calc m/z | Diff (ppm) |
|---|---|---|---|---|---|
| 1 | 68.16 | 64.75 | 653.3688 | 653.3684 | -0.55 |
| 2 | 25.81 | 27.23 | 654.372 | 654.3718 | -0.36 |
| 3 | 5.23 | 6.78 | 655.3754 | 655.3748 | -1.02 |
| 4 | 0.8 | 1.25 | 656.3775 | 656.3776 | 0.17 |



HR-ESI-MS spectrum of myrtucommuacetalone B (**3**)

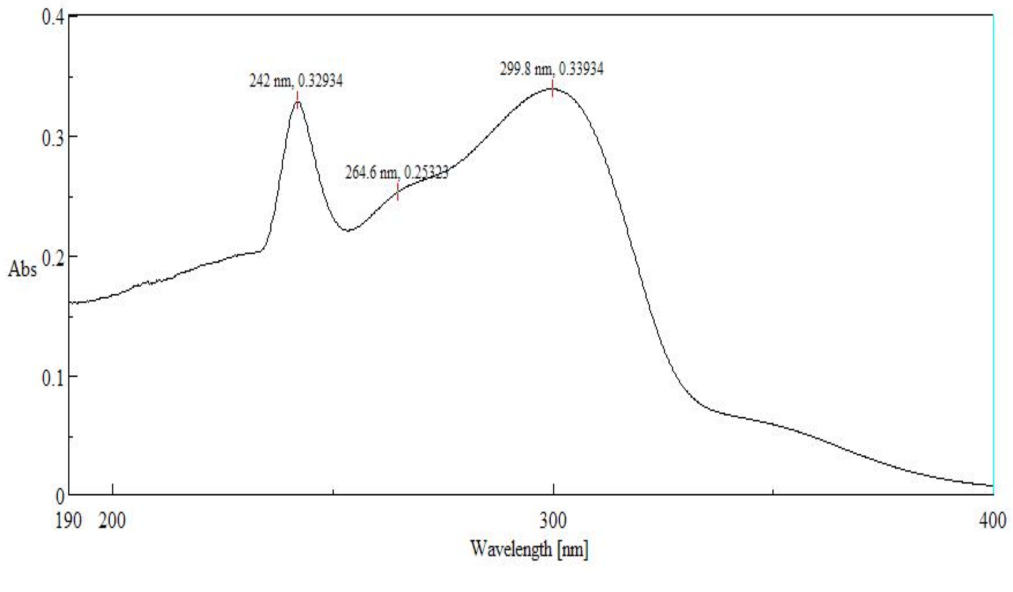


UV spectrum of myrtucommuacetalone B (**3**)

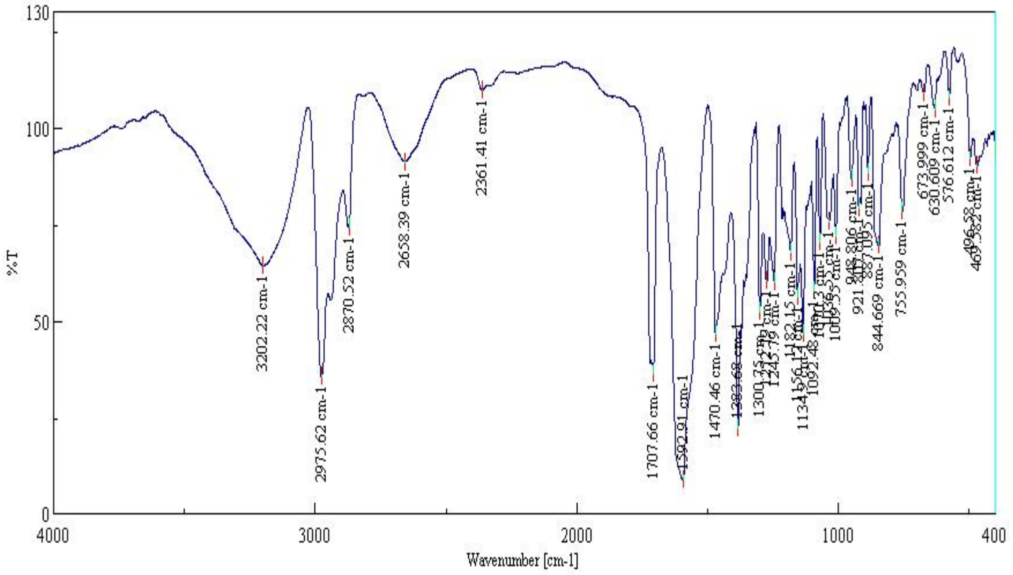

IR spectrum of myrtucommuacetalone B (**3**)

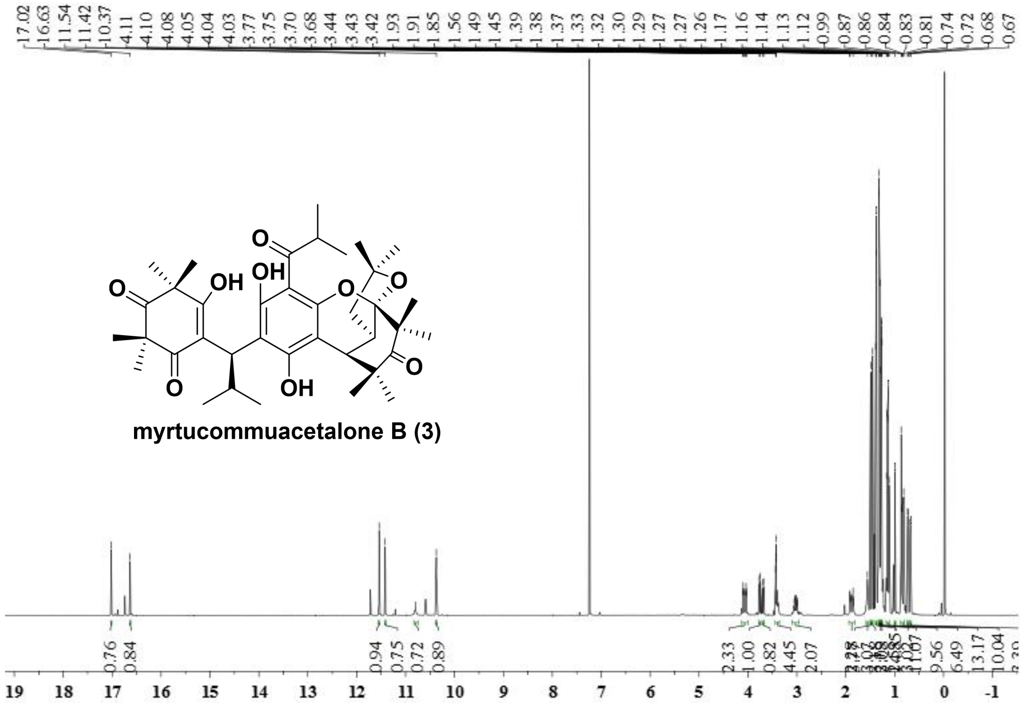

$^{1}$H NMR spectrum of myrtucommuacetalone B (**3**)

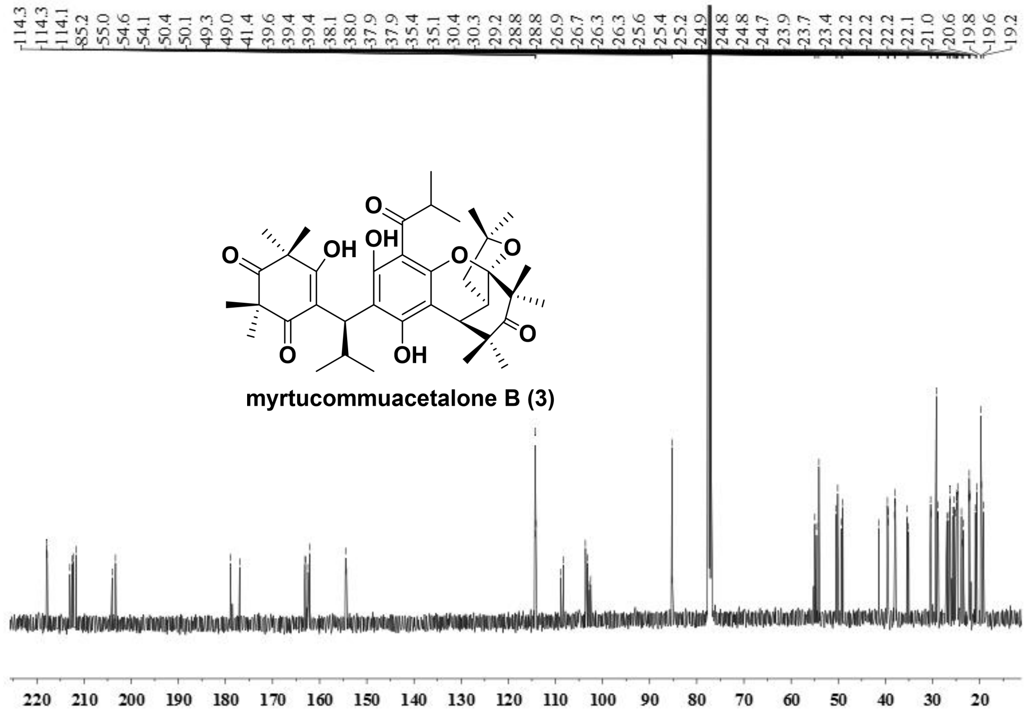


$^{13}$C NMR spectrum of myrtucommuacetalone B (**3**)

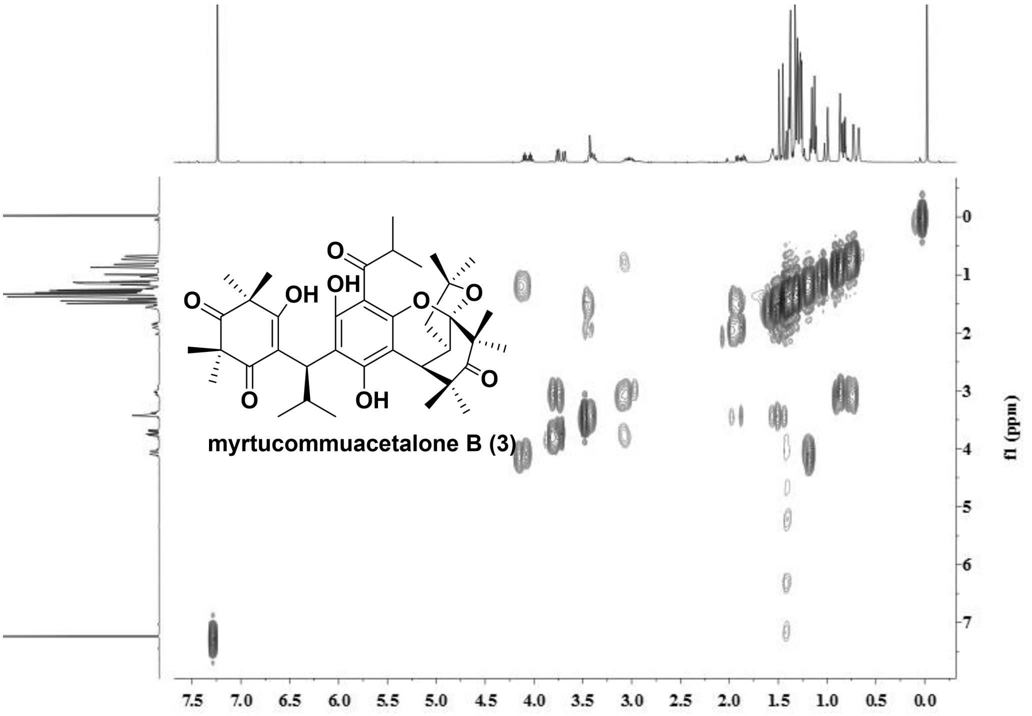


$^{1}$H-$^{1}$H COSY spectrum of myrtucommuacetalone B (**3**)

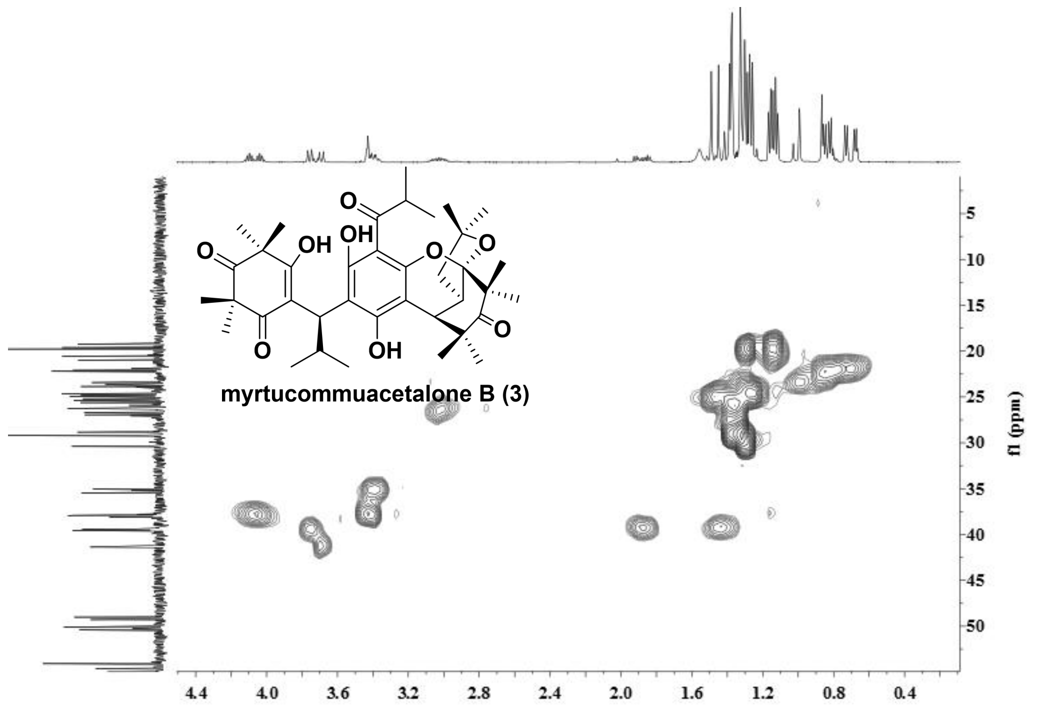


HSQC spectrum of myrtucommuacetalone B (**3**)

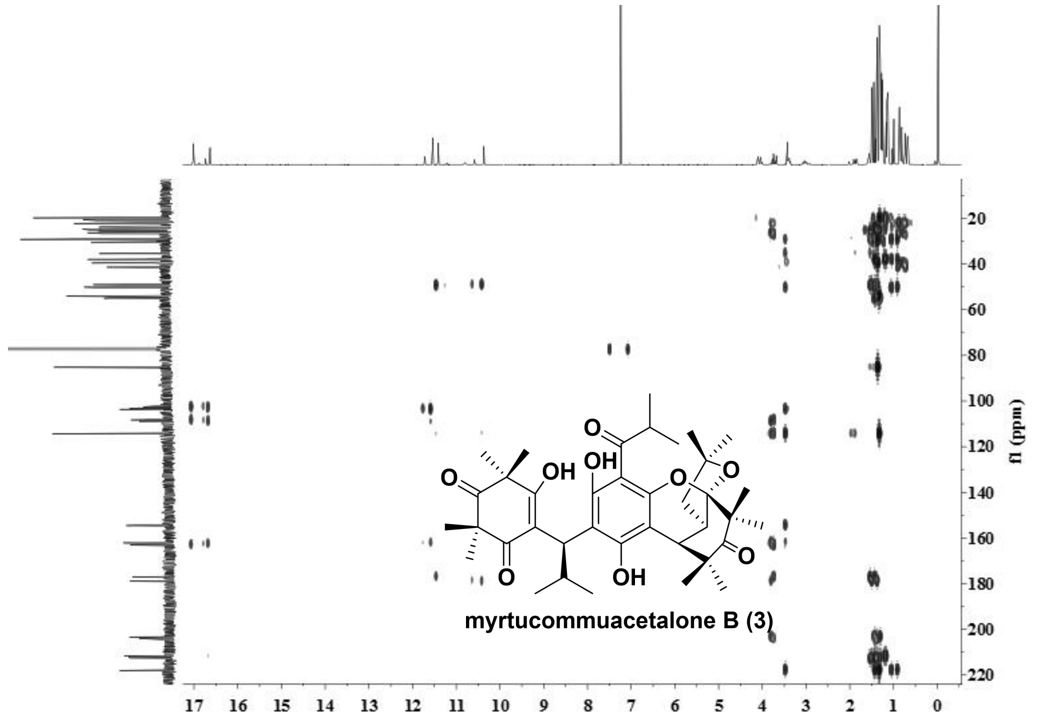


HMBC spectrum of myrtucommuacetalone B (**3**)

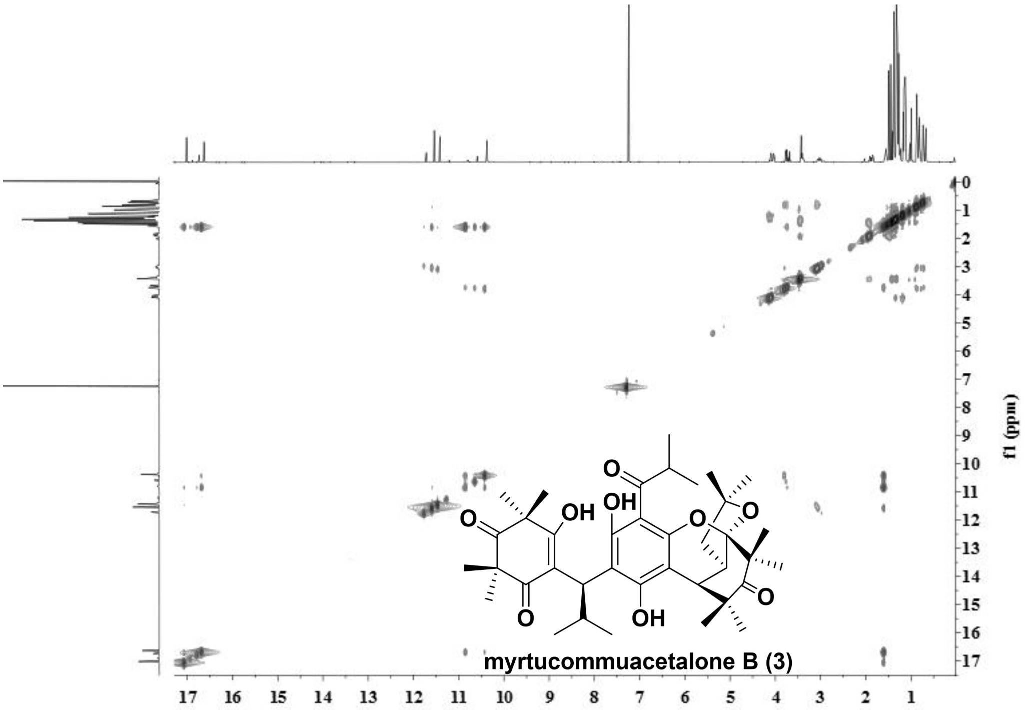


NOESY spectrum of myrtucommuacetalone B (**3**)

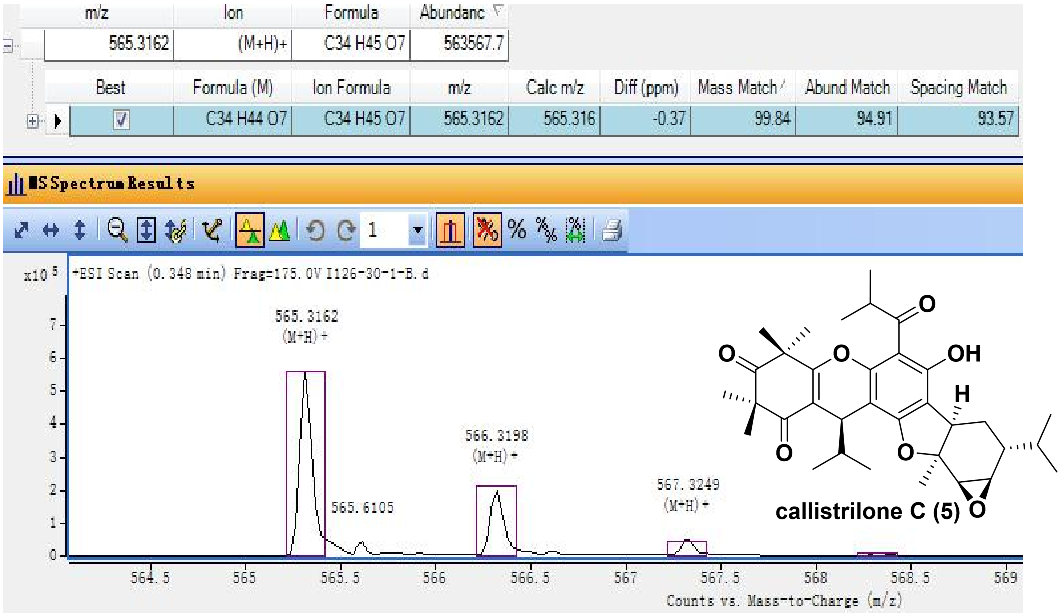


HR-ESI-MS spectrum of callistrilone C (**5**)

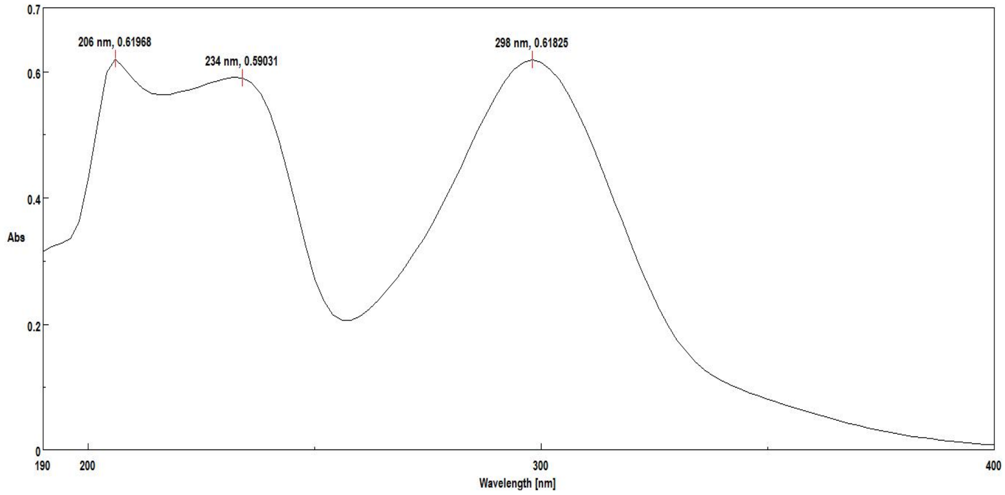


UV spectrum of callistrilone C (**5**)

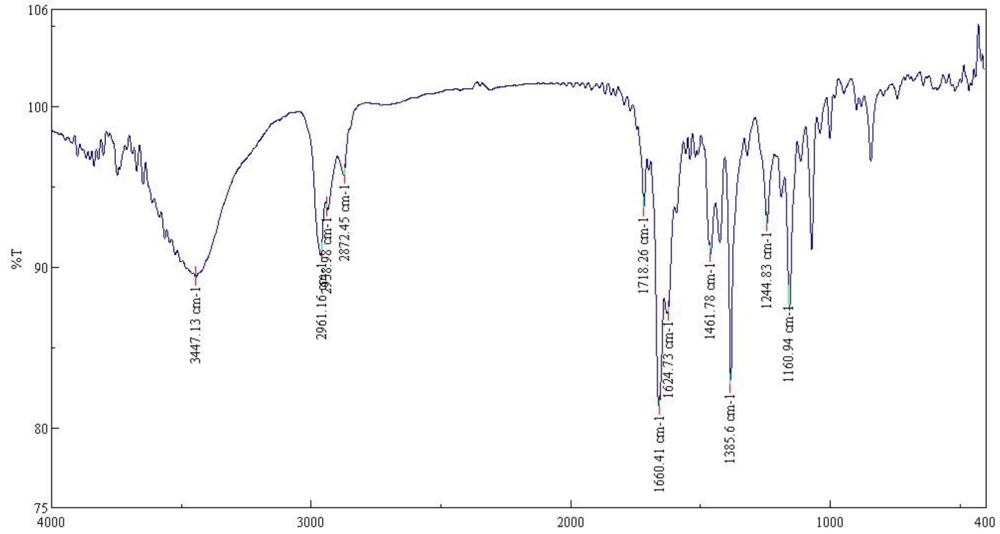


IR spectrum of callistrilone C (**5**)

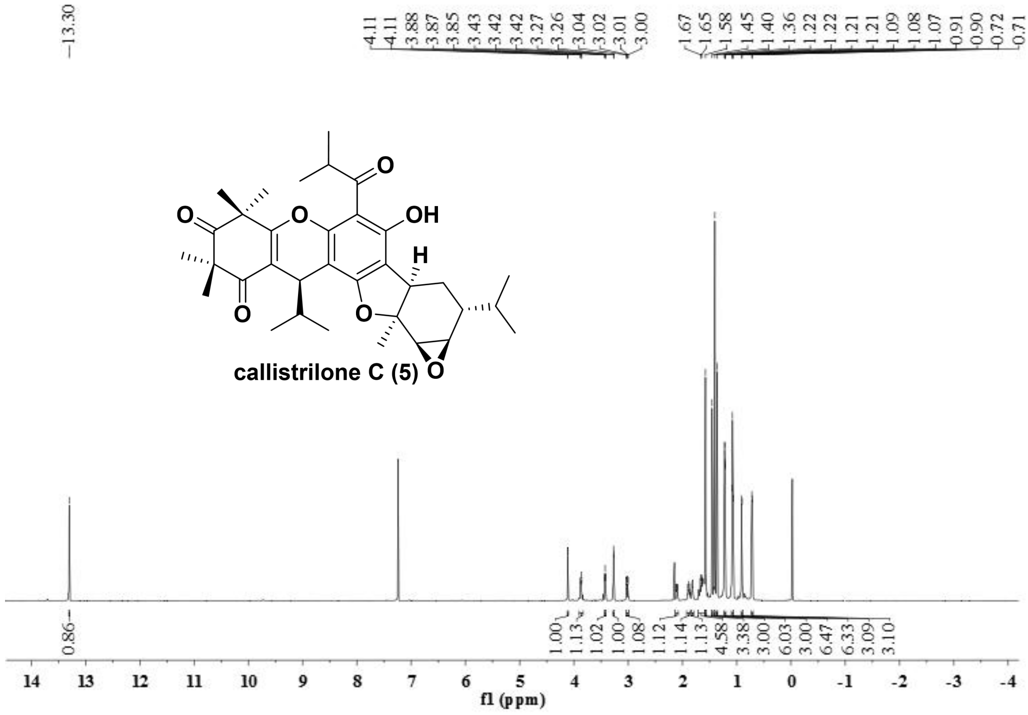


$^{1}$H NMR spectrum of callistrilone C (**5**)

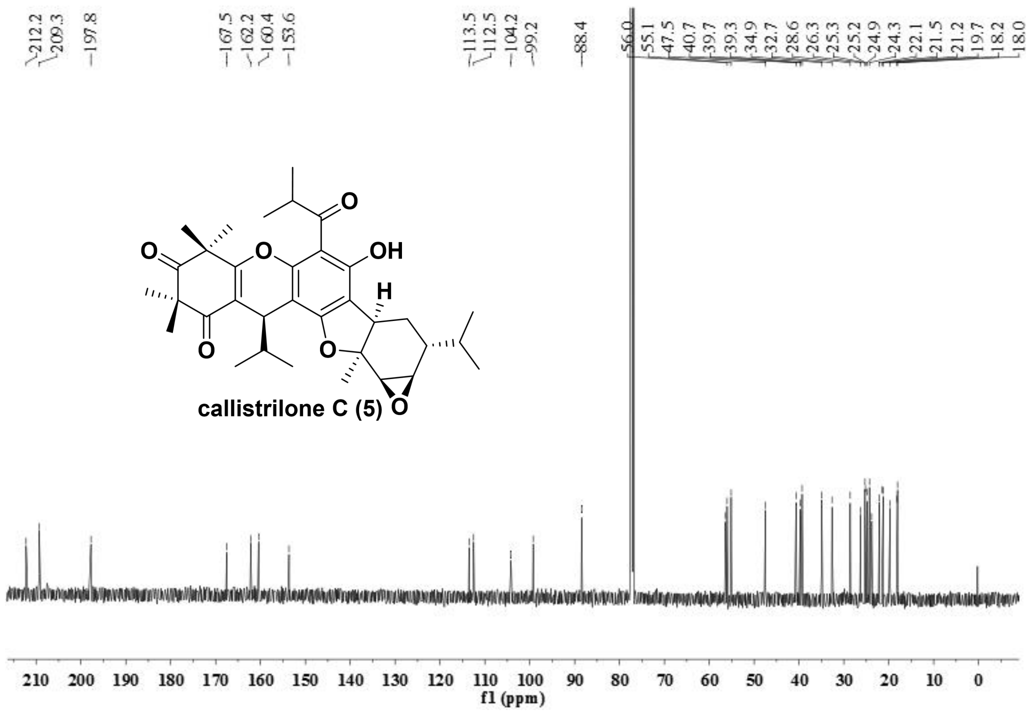


$^{13}$C NMR spectrum of callistrilone C (**5**)

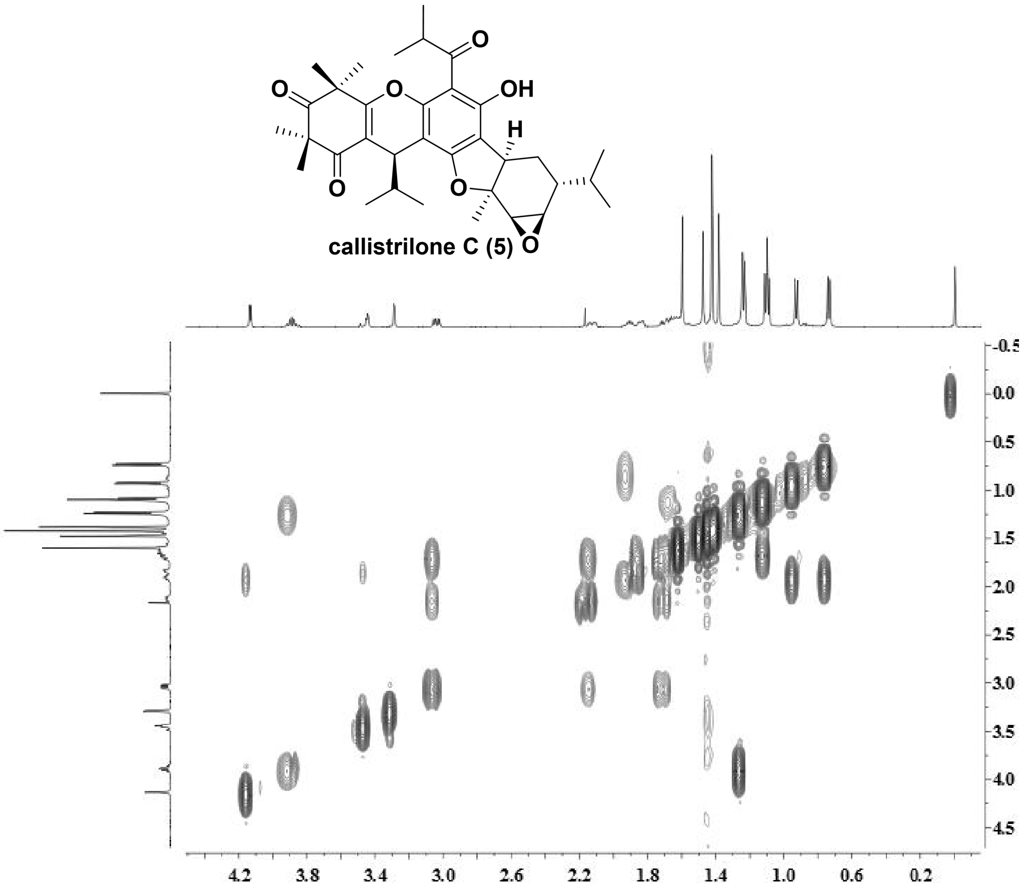


$^{1}$H-$^{1}$H COSY spectrum of callistrilone C (**5**)

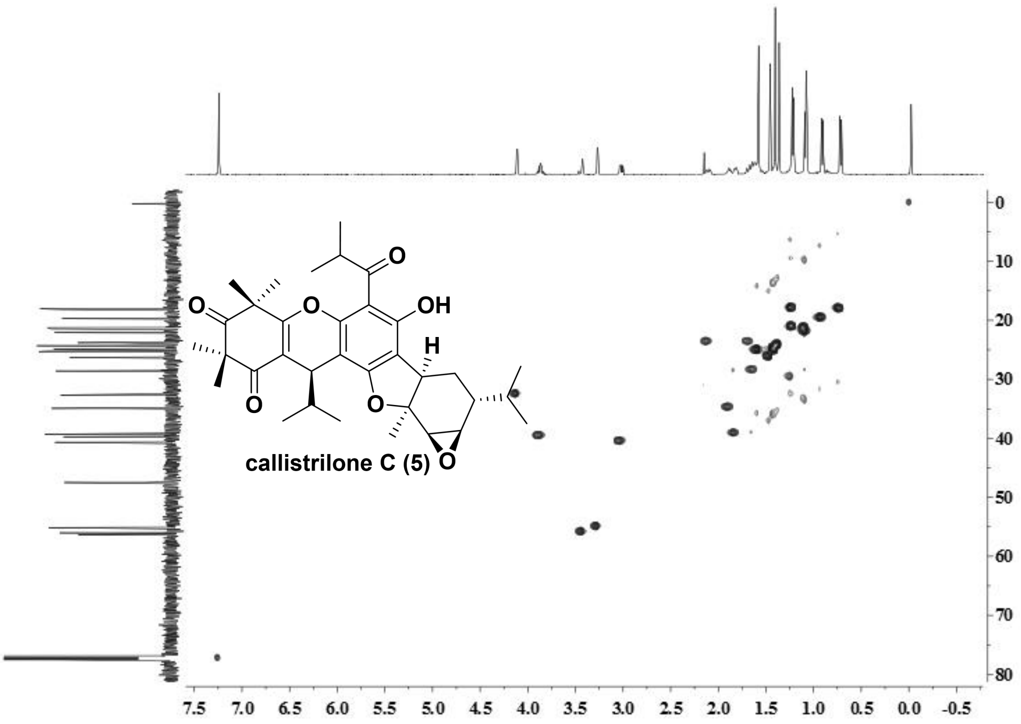


HSQC spectrum of callistrilone C (**5**)

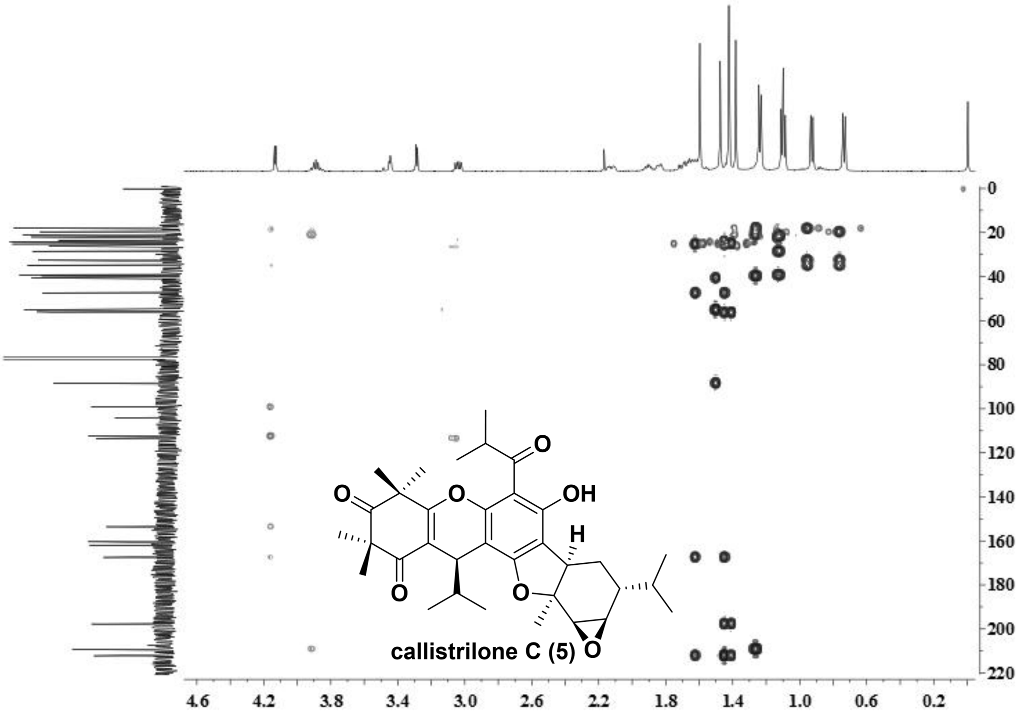


HMBC spectrum of callistrilone C (**5**)

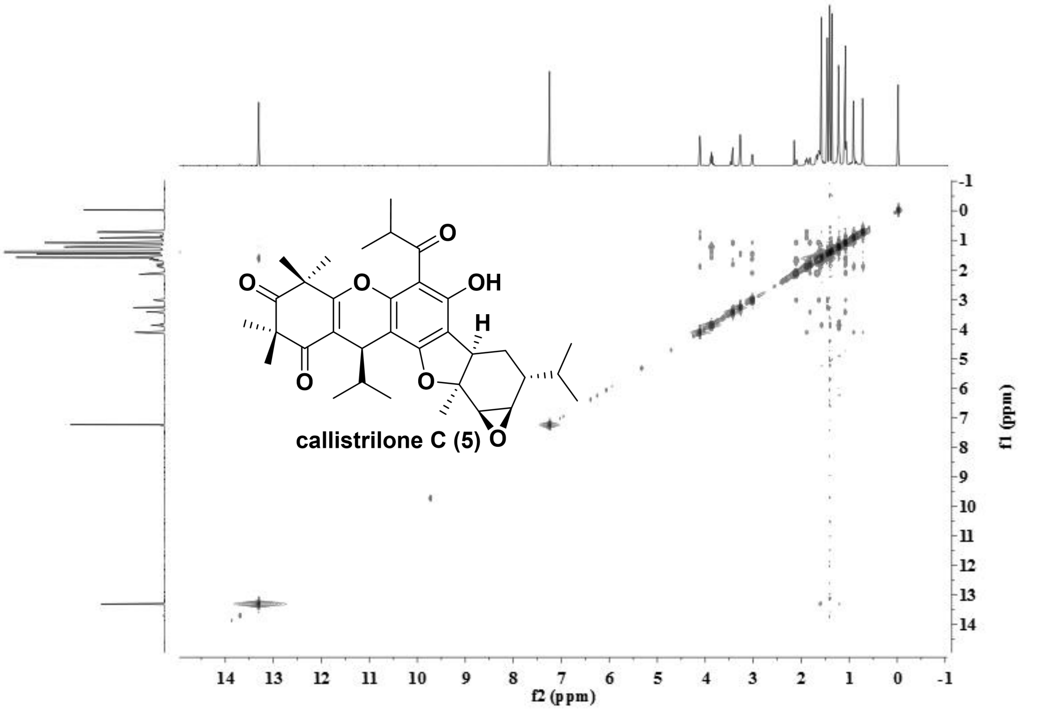


NOESY spectrum of callistrilone C (**5**)

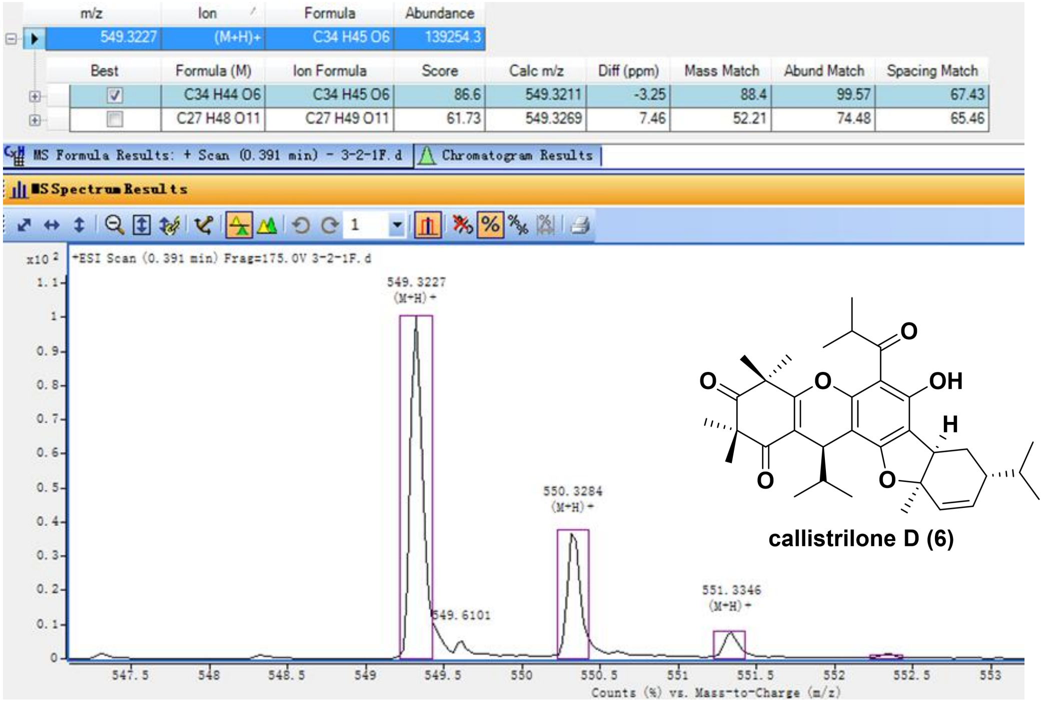


HR-ESI-MS spectrum of callistrilone D (**6**)

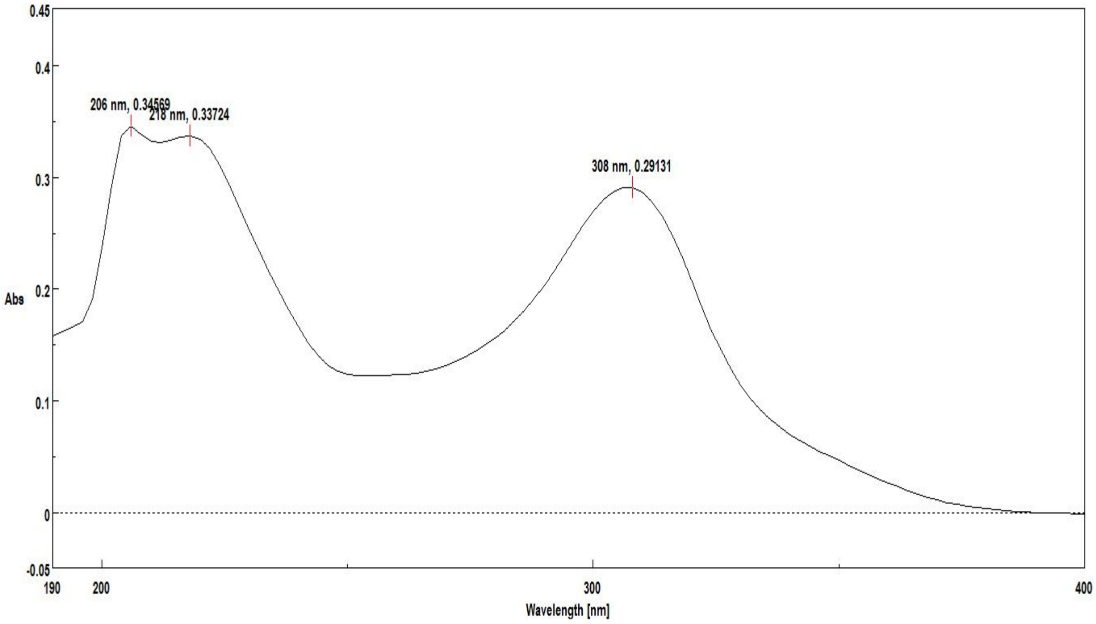


UV spectrum of callistrilone D (**6**)

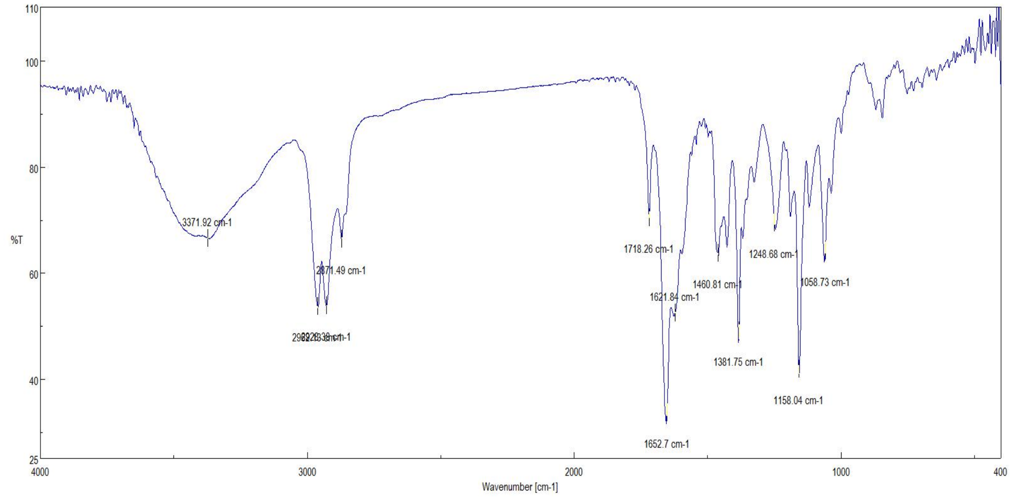


IR spectrum of callistrilone D (**6**)

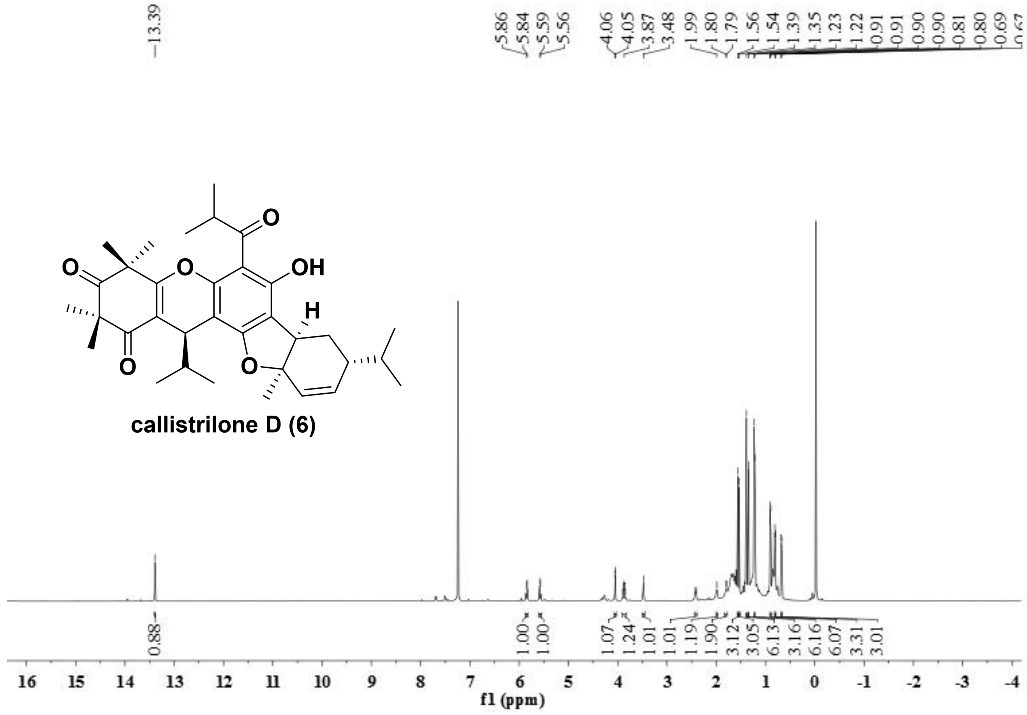


$^{1}$H NMR spectrum of callistrilone D (**6**)

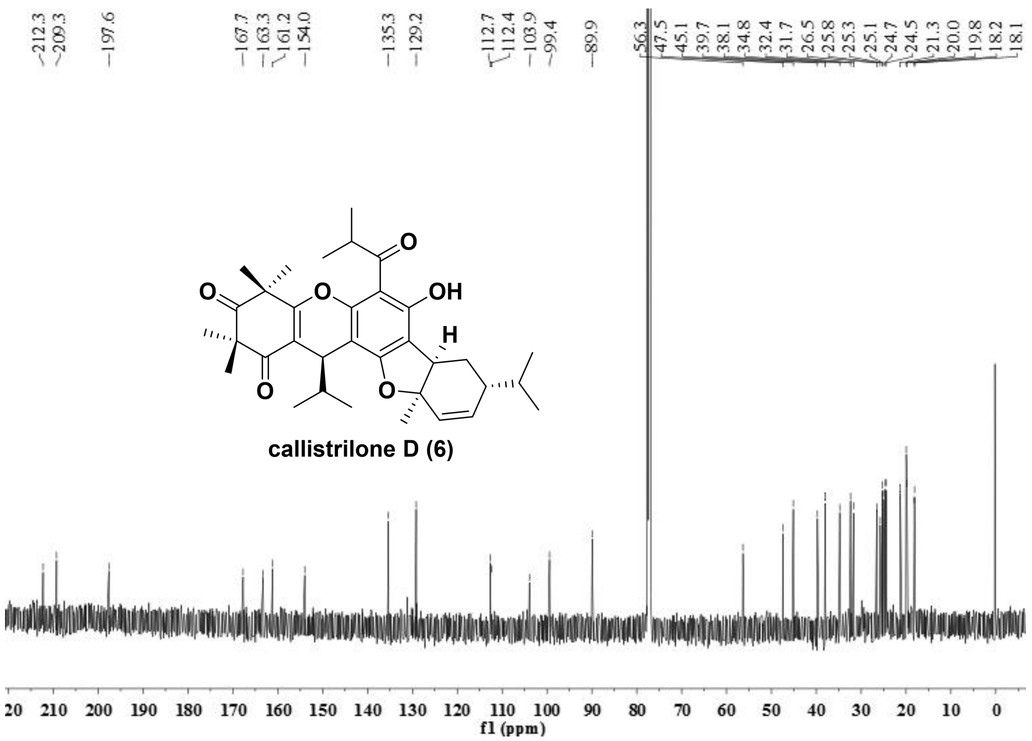


$^{13}C$ NMR spectrum of callistrilone D (**6**)

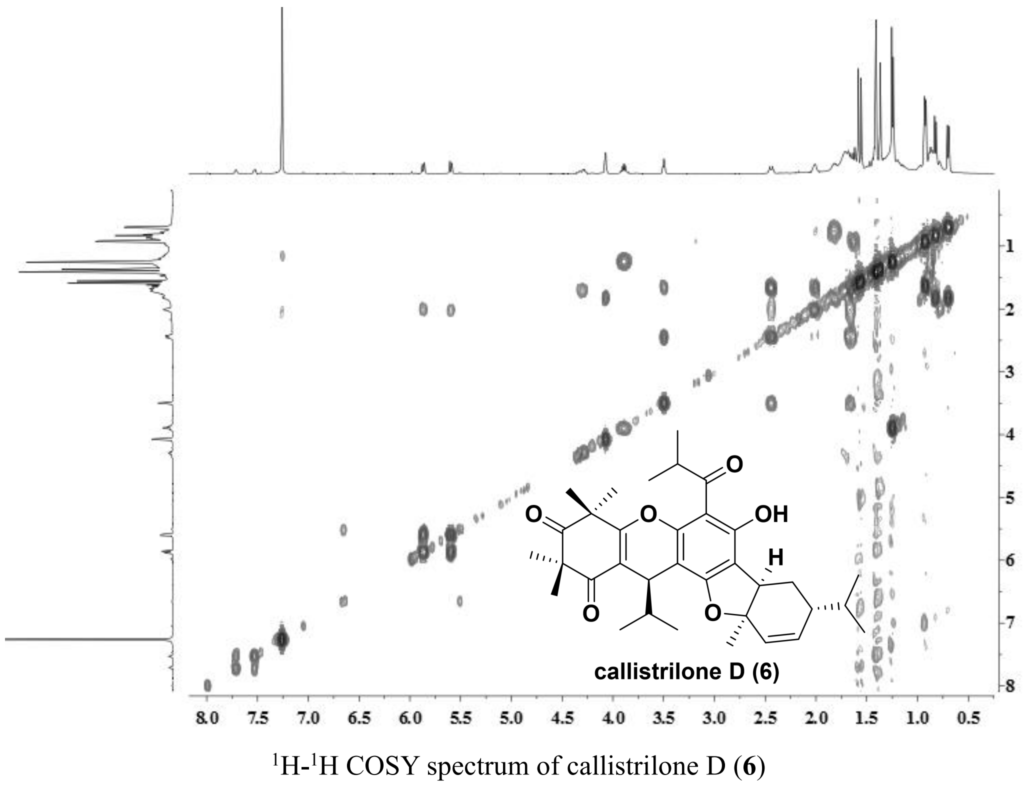


$^1H$-$^1H$ COSY spectrum of callistrilone D (**6**)

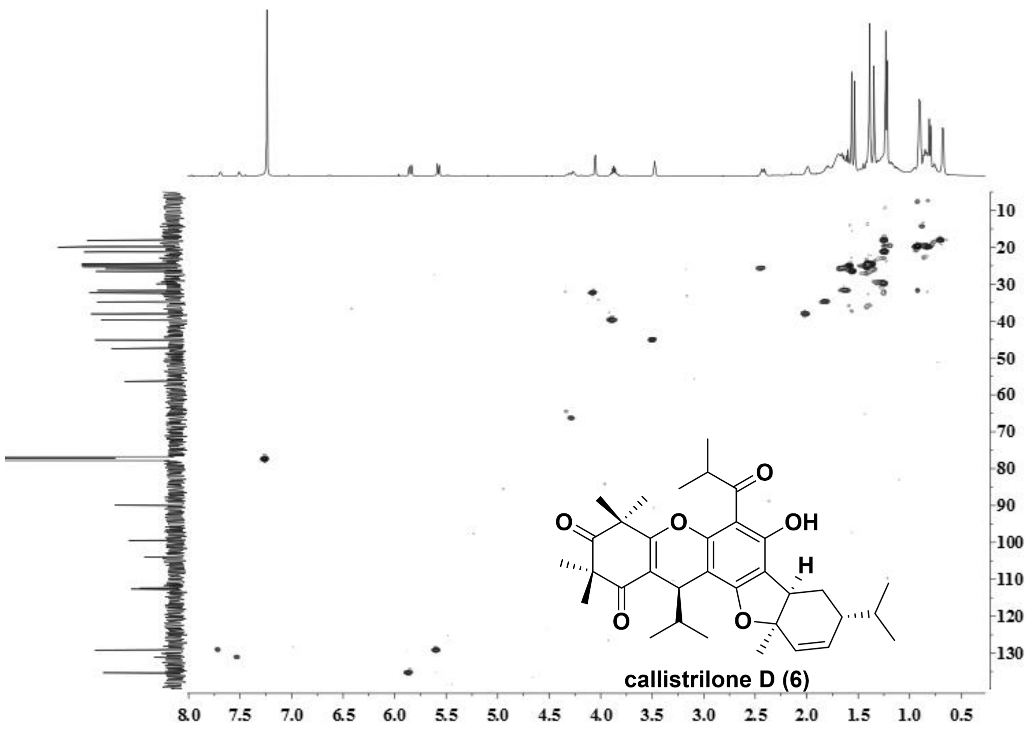


HSQC spectrum of callistrilone D (**6**)

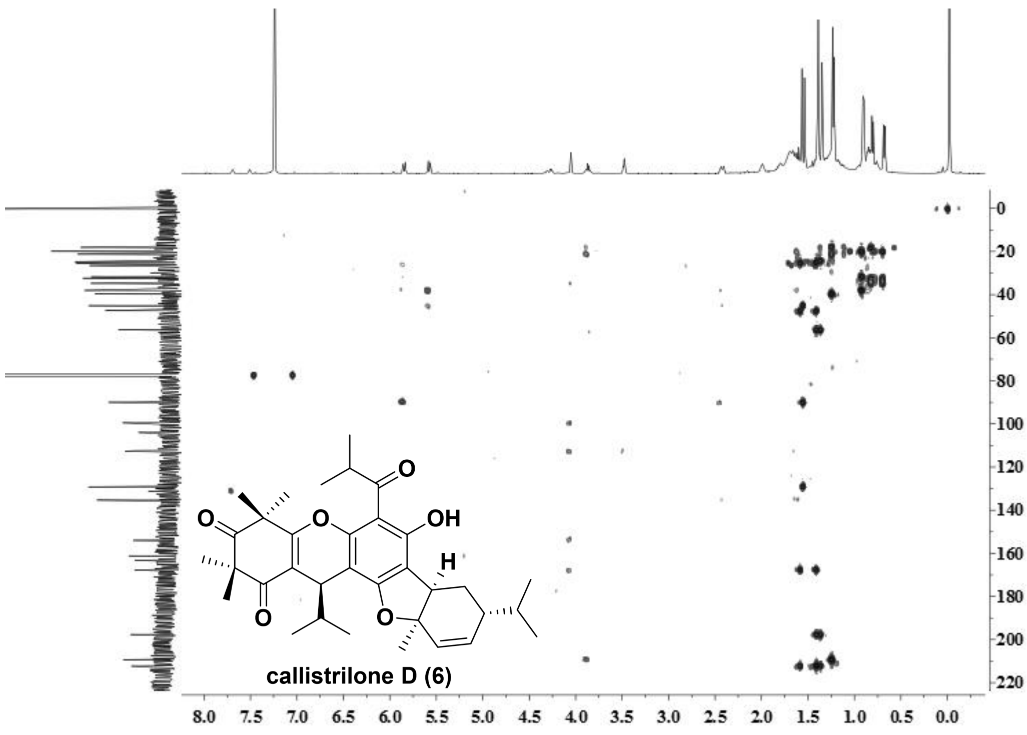


HMBC spectrum of callistrilone D (**6**)

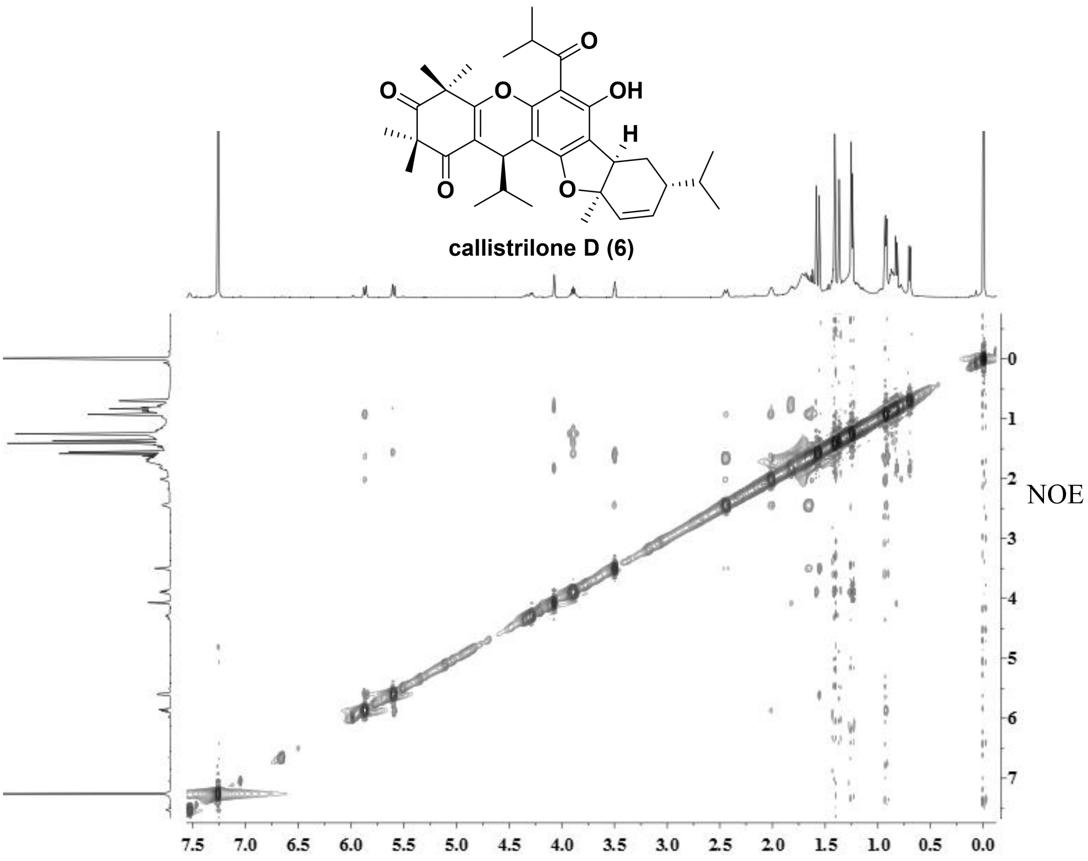


SY spectrum of callistrilone D (**6**)

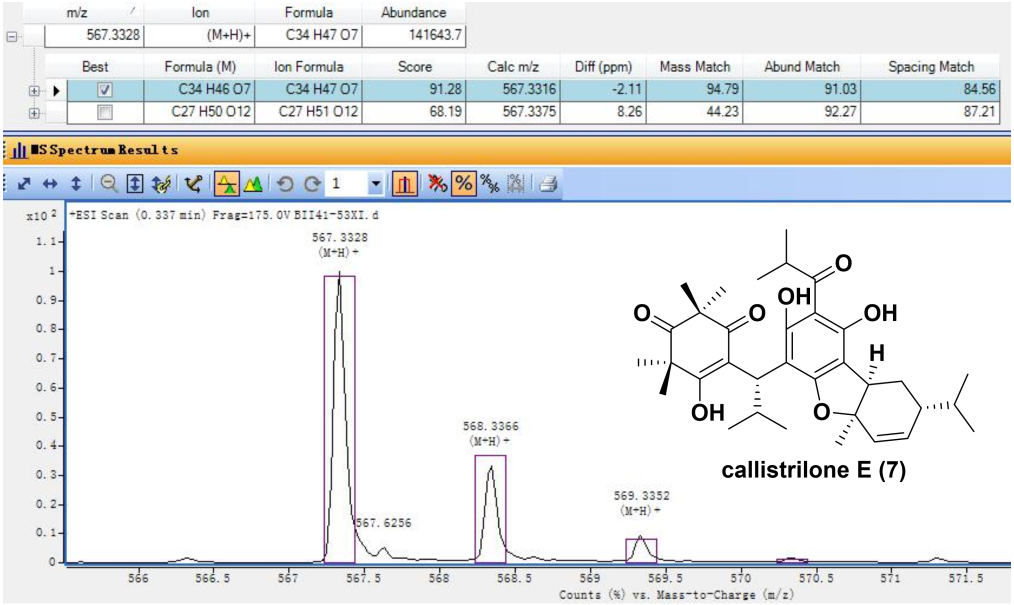

| m/z | Ion | Formula | Abundance |
|---|---|---|---|
| 567.3328 | (M+H)+ | C34 H47 O7 | 141643.7 |

| Best | Formula (M) | Ion Formula | Score | Calc m/z | Diff (ppm) | Mass Match | Abund Match | Spacing Match |
|---|---|---|---|---|---|---|---|---|
| ☑ | C34 H46 O7 | C34 H47 O7 | 91.28 | 567.3316 | -2.11 | 94.79 | 91.03 | 84.56 |
| ☐ | C27 H50 O12 | C27 H51 O12 | 68.19 | 567.3375 | 8.26 | 44.23 | 92.27 | 87.21 |



HR-ESI-MS spectrum of callistrilone E (**7**)

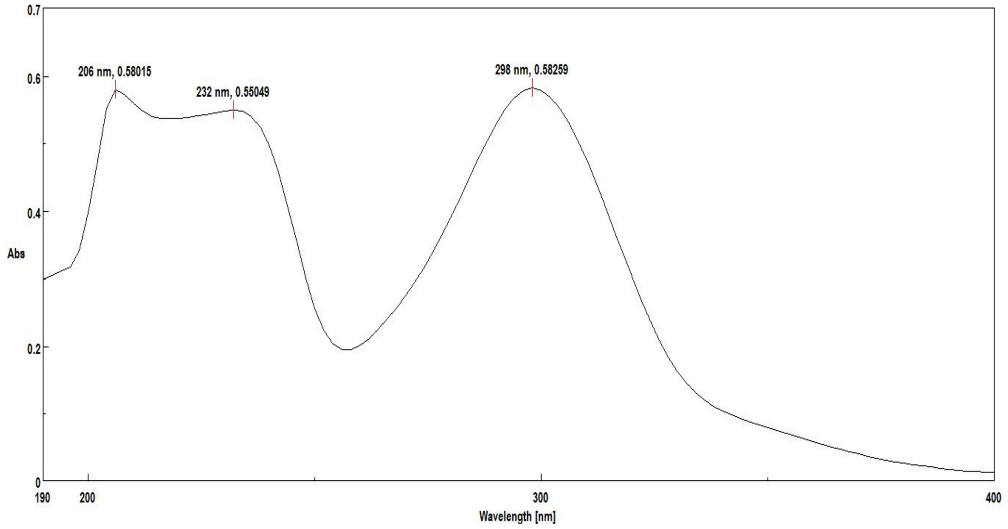


UV spectrum of callistrilone E (**7**)

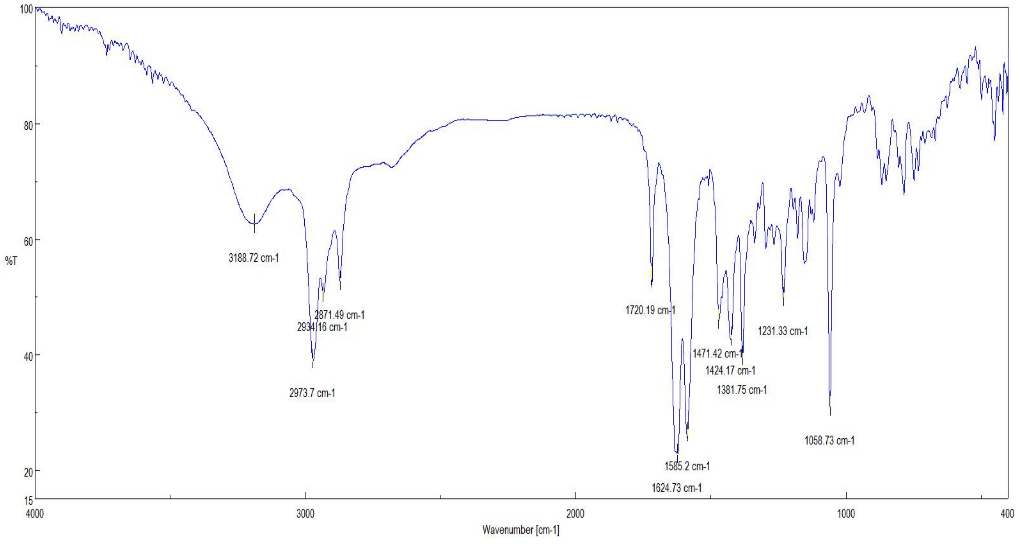


IR spectrum of callistrilone E (**7**)

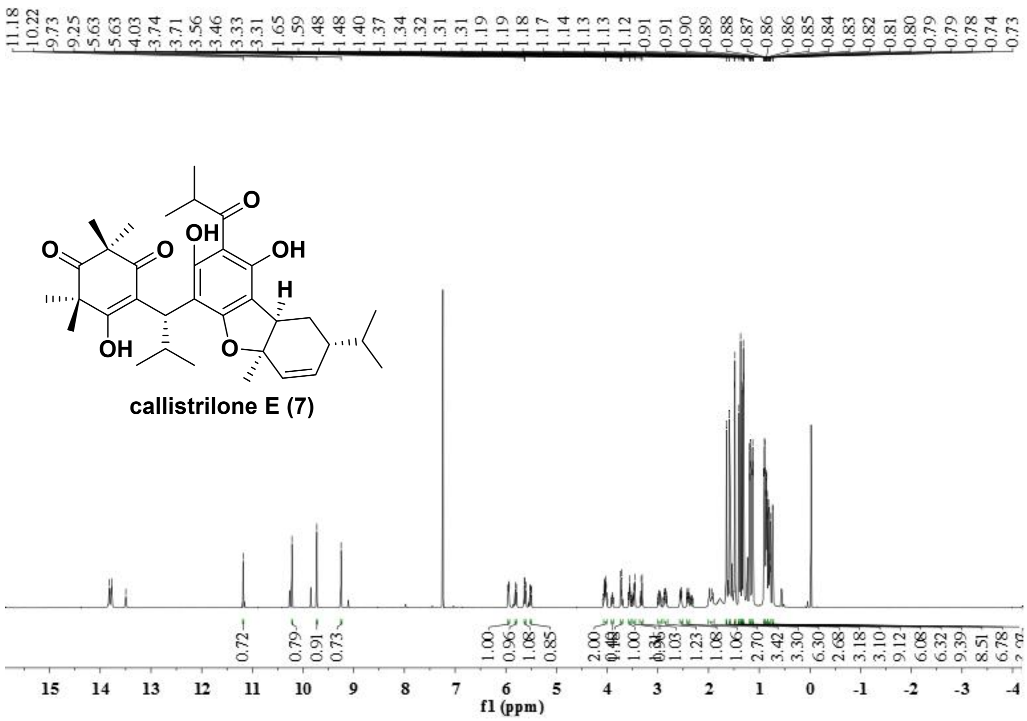


$^{1}$H NMR spectrum of callistrilone E (**7**)

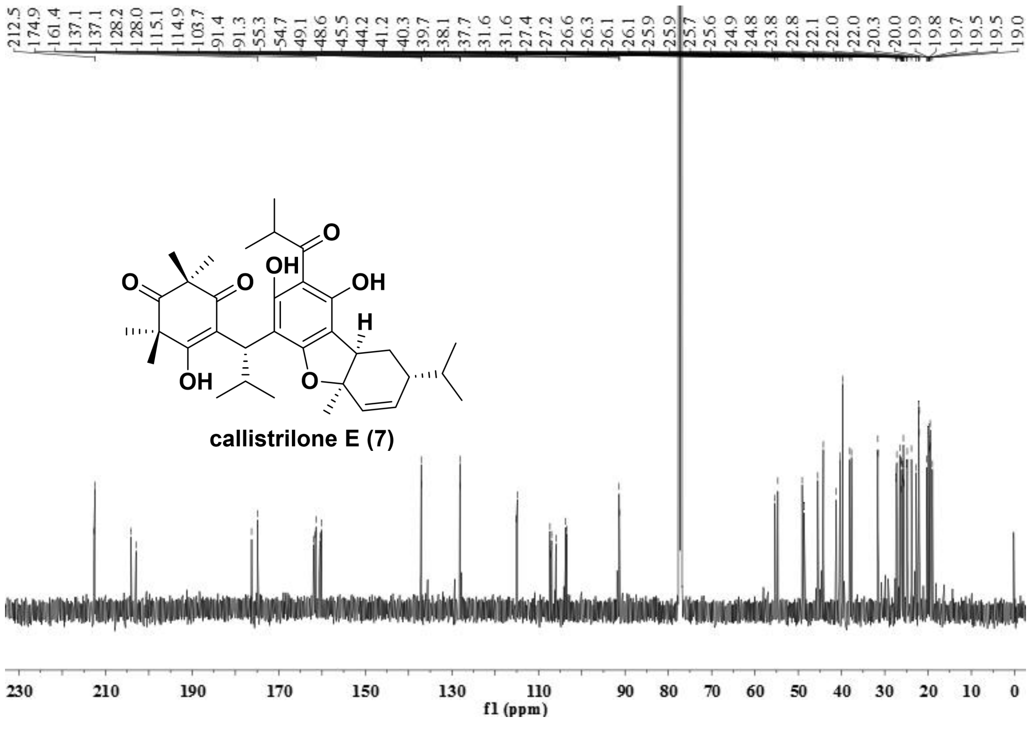


$^{13}$C NMR spectrum of callistrilone E (**7**)

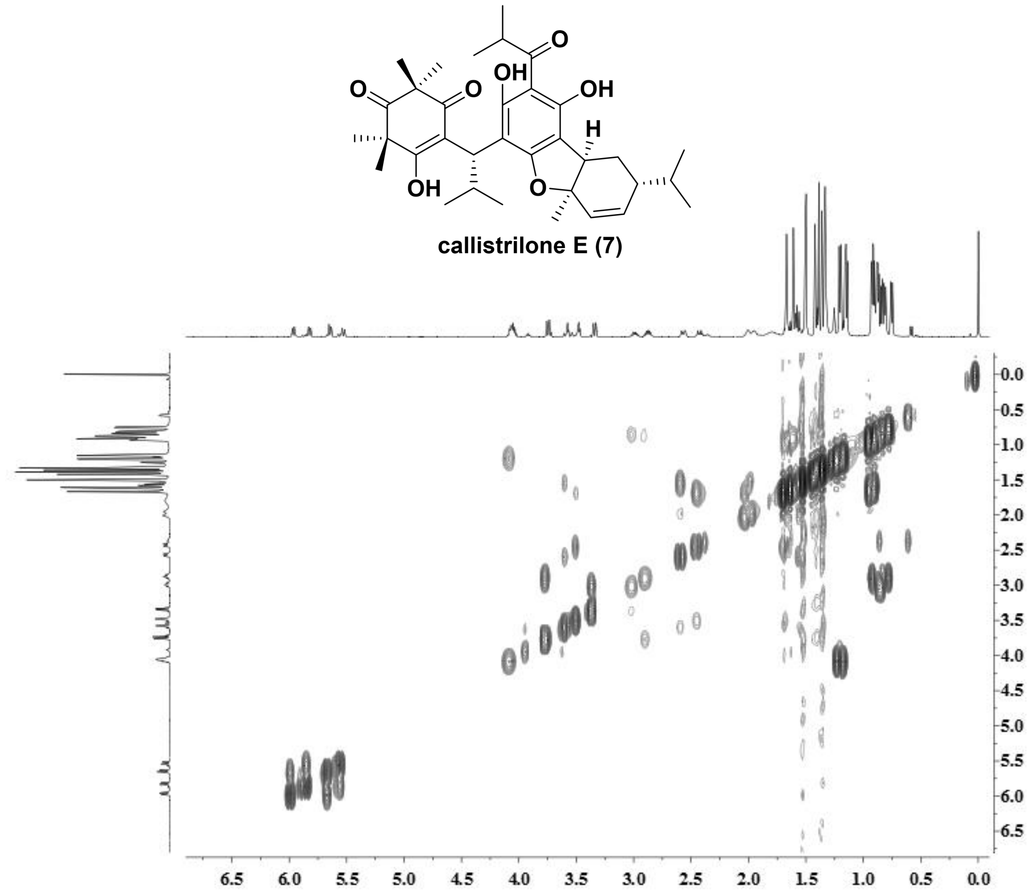


$^{1}H$-$^{1}H$ COSY spectrum of callistrilone E (**7**)

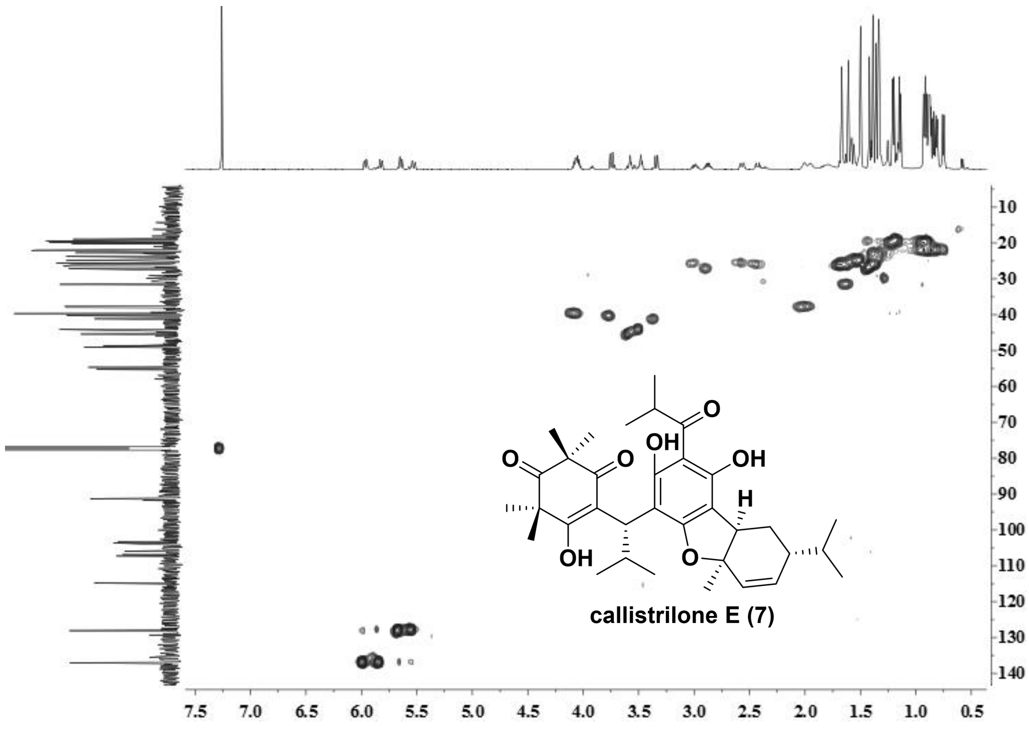


HSQC spectrum of callistrilone E (**7**)

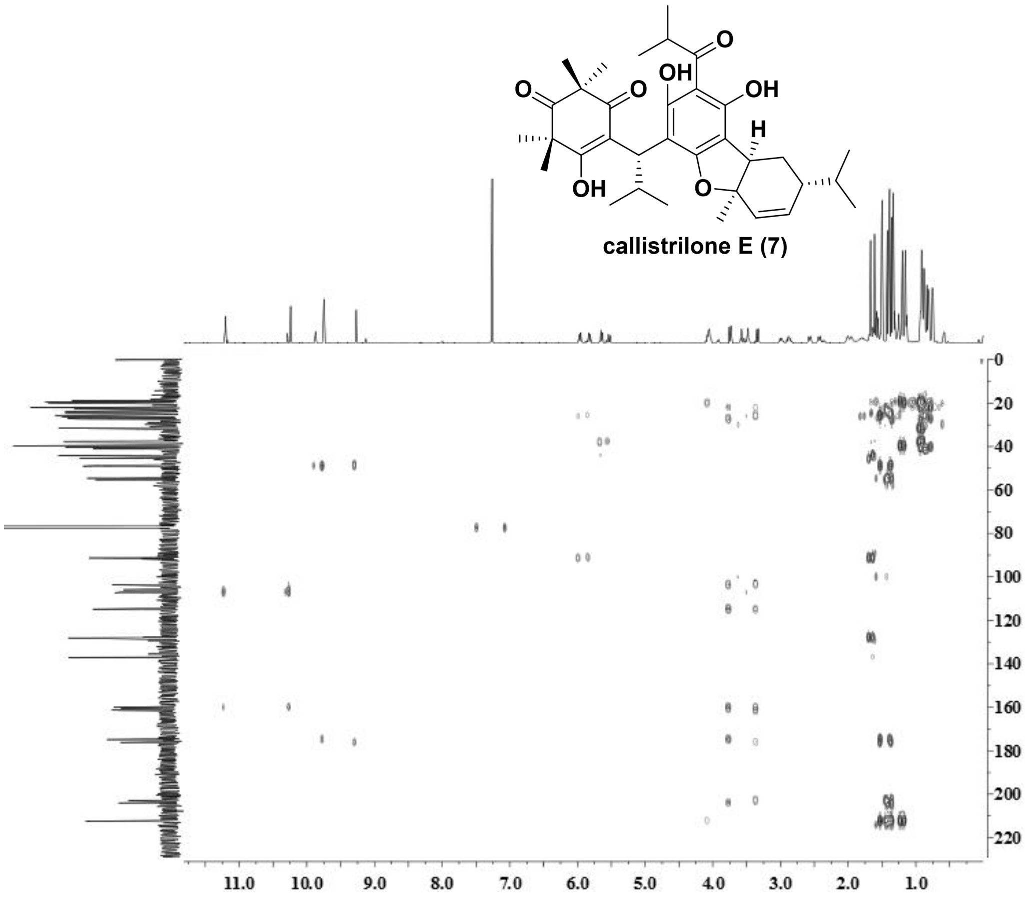


HMBC spectrum of callistrilone E (**7**)

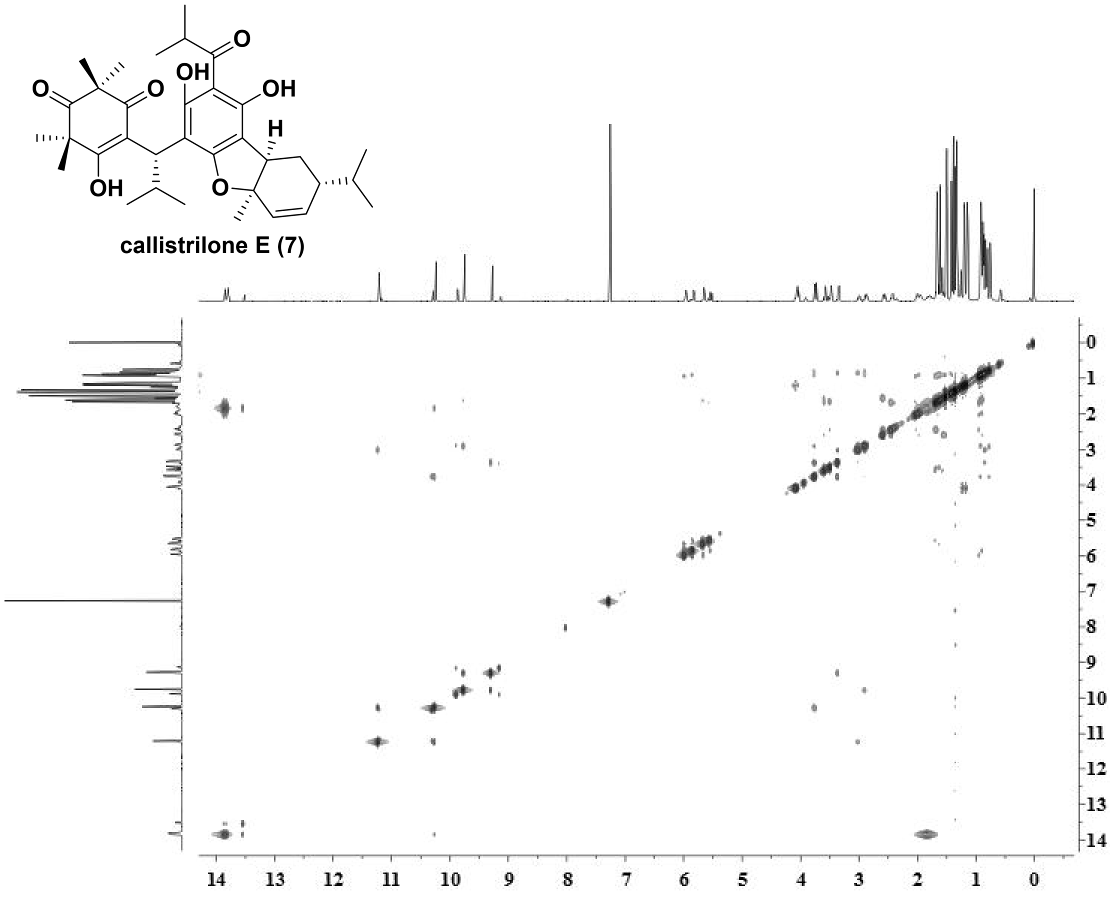


NOESY spectrum of callistrilone E (**7**)

## 2. Synthetic experimental procedures

### 2.1. General Information

Unless otherwise mentioned, all reactions were carried out under a nitrogen atmosphere under anhydrous conditions and all reagents were purchased from commercial suppliers without further purification. Solvent purification was conducted according to *Purification of Laboratory Chemicals* (Peerrin, D. D.; Armarego, W. L. and Perrins, D. R., Pergamon Press: Oxford, 1980). Yields refer to chromatographically and spectroscopically ($^1$H NMR) homogeneous materials, unless otherwise stated. Reactions were monitored by Thin Layer Chromatography on plates (GF254) supplied by Yantai Chemicals (China) using UV light as visualizing agent, an ethanolic solution of phosphomolybdic acid, or basic aqueous potassium permanganate ($KMnO_4$), and heat as developing agents. If not specially mentioned, flash column chromatography uses silica gel (200-300 mesh) supplied by Tsingtao Haiyang Chemicals (China), Preparative thin layer chromatography (PTLC) separations were carried out 0.50 mm Yantai (China) silica gel plates. NMR spectra were recorded on Bruker AV500, Bruker ARX400, and calibrated using residual undeuterated solvent as an internal reference ($CHCl_3$, δ 7.26 ppm $^1$H NMR, δ 77.00 $^{13}$C NMR). The following abbreviations were used to explain the multiplicities: s = singlet, d = doublet, t = triplet, q = quartet, b = broad, m = multiplet.

High-resolution mass spectra (HRMS) were recorded on a Bruker Apex IV FTMS mass spectrometer using ESI (electrospray ionization). Infrared spectra were recorded on a Shimadzu IR Prestige 21, using thin films of the sample on KBr plates. Optical rotations were measured with a Rudolph autopol I automatic polarimeter using 10 cm glass cells with a sodium 589 nm filter.

## 2.2 General procedure for the synthesis of 11 and 11a-11j

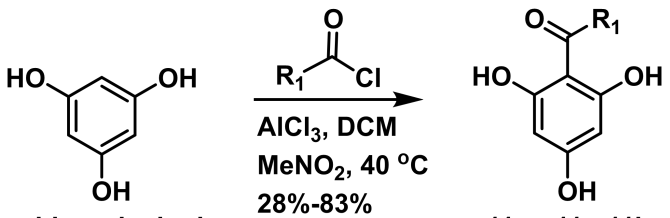


Aluminium trichloride (53.4 g, 400 mmol, 4 equiv) was added to a stirred suspension of phloroglucinol (8) (12.6 g, 100 mmol) in a mixture of dichloromethane (DCM, 100 mL) and nitromethane (100 mL), and the mixture was stirred at 26 °C. After 30 min, acyl chloride (105 mol, 1.05 equiv) was added slowly and the resulting mixture was stirred at 40 °C for 6 h. Afterwards, the mixture was cooled down to 26 °C and poured into ice-water (about 350 mL). The organic solvents were removed under a reduced pressure. This oily residue containing the acylphloroglucinol was extracted with ethyl acetate (5×350 mL) and the combined organic layers were dried over $Na_2SO_4$, filtered and concentrated in vacuo. The crude residue was purified by silica gel column chromatography (25%-50% hexanes/ethyl acetate) to afford corresponding products **11** or **11a-11j**.

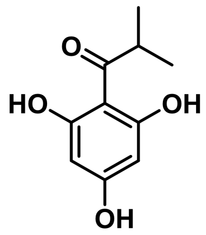


**11**

Compound **11**: 16.3 g, 83% yield, yellowish crystals, mp = 79-81 °C;

**$R_f$** = 0.5 (hexane/ethyl acetate = 1/1);

**IR (film)** $\lambda_{max}$ 3424, 3325, 2976, 1636, 1603, 1572, 1518, 1458, 1292, 1238, 1152, 974, 812;

**$^1$H NMR** (400 MHz, MeOD) δ 5.83 (s, 2H), 4.04 - 3.94 (m, 1H), 1.15 (d, J = 6.7 Hz, 6H );

**$^{13}$C NMR** (101 MHz, MeOD) δ 210.3, 164.5, 164.4, 103.2, 94.5, 38.5, 18.3;

**HRMS** (ESI) calcd for $C_{10}H_{13}O_4$ [$(M+H)^+$] Exact Mass: 197.0808; found: 197.0803.

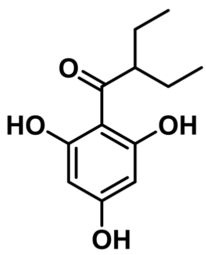

**11a**

Compound **11a**: 15.9 g, 71% yield, yellowish oil;

**R$_f$** = 0.6 (hexane/ethyl acetate = 1/1);

**IR (film)** $\lambda_{max}$ 3337, 2967, 2932, 2878, 1628, 1600, 1520, 1458, 1384, 1219, 1168, 829;

**$^1$H NMR** (500 MHz, Acetone) δ 11.87 (s, 2H), 9.30 (s, 1H), 5.94 (s, 2H), 3.86 (m, 1H), 1.78 (m, 2H), 1.49 (m, 2H), 0.88 (t, *J* = 7.4 Hz, 6H);

**$^{13}$C NMR** (125 MHz, Acetone) δ 209.3, 164.6, 164.3, 105.0, 95.1, 52.1, 24.6, 11.3;

**HRMS** (ESI) calcd for $C_{12}H_{17}O_4$ [$(M+H)^+$] Exact Mass: 225.1121; found: 225.1118.

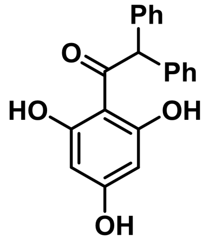

**11b**

Compound **11b**: 18.6 g, 58% yield, yellowish crystals, mp = 113-114 ºC;

**R$_f$** = 0.58 (hexane/ethyl acetate = 1/1);

**IR (film)** $\lambda_{max}$ 3371, 3028, 2959, 1624, 1600, 1597, 1520, 1450, 1354, 1215, 1177, 1072, 826;

**$^1$H NMR** (500 MHz, MeOD) δ 7.27 (m, 4H), 7.20 (m, 6H), 6.71 (s, 1H), 5.82 (s, 2H);

**$^{13}$C NMR** (125 MHz, MeOD) δ 203.7, 165.1, 164.4, 140.4, 129.3, 127.7, 126.2, 104.4, 94.6, 61.3;

**HRMS** (ESI) calcd for $C_{20}H_{17}O_4$ [$(M+H)^+$] Exact Mass: 321.1121; found: 321.1116.

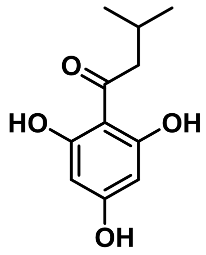

**11c**

Compound **11c**: 15.8 g, 75% yield, yellowish crystals, mp = 130-131 °C;

**R$_f$** = 0.41 (hexane/ethyl acetate = 4/1);

**IR (film)** $\lambda_{max}$ 3333, 3198, 2975, 1628, 1605, 1520, 1466, 1288, 1204, 1161, 1080, 818;

**$^1$H NMR** (500 MHz, MeOD) δ 5.82 (s, 2H), 2.92 (d, *J* = 6.8 Hz, 2H), 2.22 (m, 1H), 0.97 (s, 3H), 0.96 (s, 3H);

**$^{13}$C NMR** (125 MHz, MeOD) δ 205.6, 164.6, 164.4, 104.2, 94.3, 52.3, 25.3, 21.8;

**HRMS** (ESI) calcd for $C_{11}H_{15}O_4$ [(M+H)$^+$] Exact Mass: 211.0965; found: 211.0959.

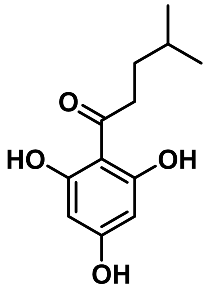

**11d**

Compound **11d**: 15.9 g, 71% yield, yellowish crystals, mp = 104-105 °C;

**R$_f$** = 0.41 (hexane/ethyl acetate = 2/1);

**IR (film)** $\lambda_{max}$ 3314, 2954, 2927, 2870, 1655, 1597, 1520, 1470, 1389, 1215, 1069, 988, 814;

**$^1$H NMR** (500 MHz, MeOD) δ 5.82 (s, 2H), 3.04 (m, 2H), 1.62 (m, 1H), 1.55 (m, 2H), 0.95 (s, 3H), 0.94 (s, 3H);

**$^{13}$C NMR** (125 MHz, MeOD) δ 206.4, 164.6, 164.4, 103.9, 94.3, 41.6, 34.0, 27.9, 21.5;

**HRMS** (ESI) calcd for $C_{12}H_{17}O_4$ [(M+H)$^+$] Exact Mass: 225.1121; found: 225.1116.

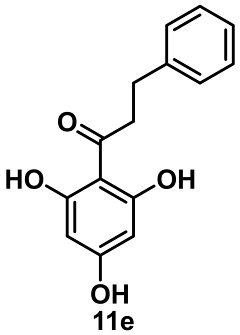

**11e**

Compound **11e**: 7.2 g, 28% yield, yellowish crystals, mp = 134-135 °C;

**R$_f$** = 0.41 (hexane/ethyl acetate = 2/1);

**IR (film)** $\lambda_{max}$ 3298, 3024, 2870, 1651, 1609, 1520, 1470, 1378, 1207, 1157, 1080, 976, 818;

**$^1$H NMR** (500 MHz, Acetone) δ 11.78 (s, 2H), 9.30 (s, 1H), 7.27 (m, 4H), 7.17 (m, 1H), 5.95 (s, 2H), 3.40 (m, 2H), 2.98 (m, 2H);

**$^{13}$C NMR** (125 MHz, Acetone) δ 204.3, 164.6, 164.5, 142.0, 128.4, 128.3, 125.8, 104.2, 95.0, 45.5, 30.5;

**HRMS** (ESI) calcd for $C_{15}H_{15}O_4$ [$(M+H)^+$] Exact Mass: 259.0965; found: 259.0960.

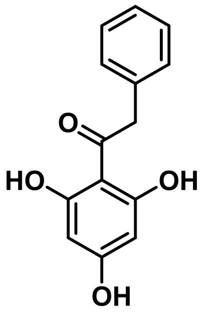

**11f**

Compound **11f**: 18.5 g, 76% yield, yellowish crystals, mp = 157-159 °C;

**$R_f$** = 0.52 (hexane/ethyl acetate = 1/1);

**IR (film)** $\lambda_{max}$ 3282, 3179, 1697, 1636, 1597, 1520, 1458, 1354, 1231, 1165, 1076, 991, 821;

**$^1$H NMR** (500 MHz, MeOD) δ 7.26 (m, 4H), 7.20 (m, 1H), 5.84 (s, 2H), 4.39 (s, 2H);

**$^{13}$C NMR** (125 MHz, MeOD) δ 203.1, 165.0, 164.5, 136.0, 129.4, 127.8, 126.0, 103.9, 94.4, 49.1;

**HRMS** (ESI) calcd for $C_{14}H_{13}O_4$ [$(M+H)^+$] Exact Mass: 245.0808; found: 245.0806.

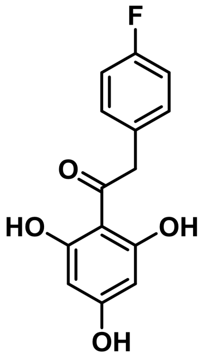

**11g**

Compound **11g**: 17.8 g, 68% yield, yellowish crystals, mp = 184-186 °C;

**$R_f$** = 0.5 (hexane/ethyl acetate = 1/1);

**IR (film)** $\lambda_{max}$ 3368, 3275, 1643, 1605, 1566, 1508, 1458, 1346, 1231, 1153, 1076, 988, 818;

**$^1$H NMR** (500 MHz, Acetone) δ 11.83 (s, 2H), 9.36 (s, 1H), 7.32 (m, 2H), 7.06 (m, 2H), 5.98 (s,

2H), 4.42 (s, 2H);

**$^{13}$C NMR** (125 MHz, Acetone) δ 205.8, 202.5, 164.6, 164.3, 162.6, 160.7, 132.1, 132.1, 131.6, 131.6, 114.7, 114.5, 95.0, 48.5;

**HRMS** (ESI) calcd for $C_{14}H_{12}O_4F$ [$(M+H)^+$] Exact Mass: 263.0714; found: 263.0708.

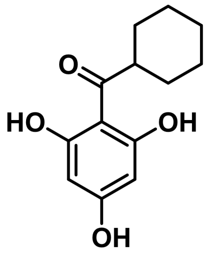

**11h**

Compound **11h**: 17.0 g, 72% yield, yellowish crystals, 76-78 °C;

**R$_f$** = 0.6 (hexane/ethyl acetate = 1/1);

**IR (film)** $\lambda_{max}$ 3310, 2920, 2851, 1643, 1601, 1558, 1520, 1443, 1377, 1211, 1165, 1072, 810;

**$^1$H NMR** (500 MHz, MeOD) δ 5.82 (s, 2H), 3.70 (m, 1H), 1.81 (m, 5H), 1.38 (m, 3H), 1.26 (m, 1H);

**$^{13}$C NMR** (125 MHz, MeOD) δ 209.4, 164.4, 164.4, 103.4, 94.5, 49.2, 29.3, 26.0, 25.9;

**HRMS** (ESI) calcd for $C_{13}H_{17}O_4$ [$(M+H)^+$] Exact Mass: 237.1121; found: 237.1116.

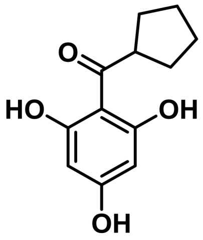

**11i**

Compound **11i**: 15.5 g, 70% yield, yellowish crystals, mp = 100-101 °C;

**R$_f$** = 0.6 (hexane/ethyl acetate = 1/1);

**IR (film)** $\lambda_{max}$ 3260, 2955, 2870, 1628, 1609, 1582, 1524, 1470, 1366, 1223, 1169, 1076, 818;

**$^1$H NMR** (500 MHz, MeOD) δ 5.82 (s, 2H), 4.15 (m, 1H), 1.92 (m, 2H), 1.83 (m, 2H), 1.73 (m, 2H), 1.61 (m, 2H);

**$^{13}$C NMR** (125 MHz, MeOD) δ 208.6, 164.4, 164.4, 103.7, 94.4, 50.0, 29.6, 25.7;

**HRMS** (ESI) calcd for $C_{12}H_{15}O_4$ [$(M+H)^+$] Exact Mass: 223.0965; found: 223.0961.

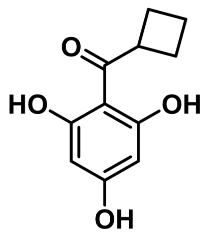

**11j**

Compound **11j**: 13.7 g, 66% yield, yellowish crystals, mp = 98-99 °C;

**R$_f$** = 0.6 (hexane/ethyl acetate = 1/1);

**IR (film)** $\lambda_{max}$ 3240, 2982, 2866, 1639, 1605, 1574, 1520, 1458, 1362, 1229, 1165, 1053, 822;

**$^1$H NMR** (500 MHz, MeOD) δ 5.80 (s, 2H), 4.25 (m, 1H), 2.33 (m, 2H), 2.26 (m, 2H), 1.97 (m, 1H), 1.82 (m, 1H);

**$^{13}$C NMR** (125 MHz, MeOD) δ 205.9, 164.5, 164.4, 102.8, 94.2, 46.0, 24.4, 17.1;

**HRMS** (ESI) calcd for $C_{11}H_{13}O_4$ [(M+H)$^+$] Exact Mass: 209.0808; found: 209.0805.

### 2.3 General procedure for the synthesis of 12a and 12aa-12af

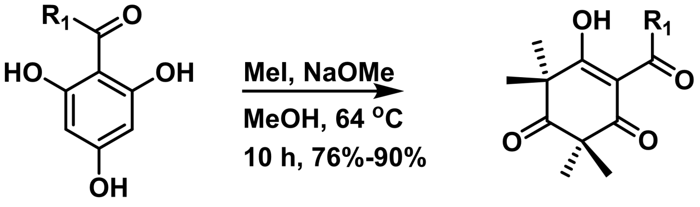


**11** or **11a-11j** **12a** or **12aa-12af**

To a solution of NaOMe (44.1 g, 816 mmol, 8 equiv.) and **11** or **11a-11j** (102 mmol, 1 equiv.) in MeOH (350 mL), iodomethane (89 mL, 1430 mmol, 14 equiv.) was added and the resulting mixture was refluxed for 8 h. Then the mixture was cooled down to 26 °C and concentrated under reduced pressure. The residue was re-dissolved in water (100 mL), acidified with 2M HCl (400 mL), before it was extracted with ethyl acetate (3×400 mL). Then the combined organic layers were washed with saturated aqueous sodium sulfite solution (2×700 mL), dried over $Na_2SO_4$, filtered and concentrated in vacuo. The crude residue was purified by silica gel column chromatography (1%-4% hexanes/ethyl acetate) to afford corresponding products **12a** or **12aa-12af**.

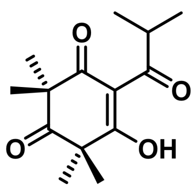

**12a**

Compound **12a**: 23.1 g, 90% yield, yellow oil;

**R$_f$** = 0.5 (hexane/ethyl acetate = 20/1);

**IR (film)** $\lambda_{max}$ 3412, 2962, 2940, 2876, 1724, 1672, 1558, 1474, 1420, 1314, 1049, 939;

**$^1$H NMR** (400 MHz, CDCl3) δ 3.84 – 3.74 (m, 1H), 1.43 (s, 6H), 1.35 (s, 6H), 1.17 (s, 3H), 1.16 (s, 3H);

**$^{13}$C NMR** (100 MHz, CDCl3) δ 209.9, 208.6, 199.3, 196.9, 108.2, 56.9, 52.2, 35.2, 24.3, 23.9, 19.1;

**HRMS** (ESI) calcd for $C_{14}H_{19}O_4$ [(M-H)$^-$] Exact Mass: 251.1289; found: 251.1287.

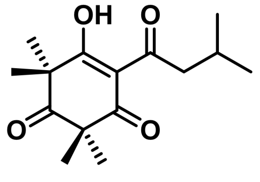

**12aa**

Compound **12aa**: 23.9 g, 88% yield, yellow oil;

**R$_f$** = 0.5 (hexane/ethyl acetate = 20/1);

**IR (film)** $\lambda_{max}$ 3422, 2963, 2940, 2874, 1721, 1674, 1543, 1470, 1420, 1385, 1049, 968, 872;

**$^1$H NMR** (500 MHz, $CDCl_3$) δ 2.85 (d, $J$ = 7.0 Hz, 2H), 2.15 (m, 1H), 1.43 (s, 6H), 1.34 (s, 6H), 0.97 (s, 3H), 0.96 (s, 3H);

**$^{13}$C NMR** (125 MHz, $CDCl_3$) δ 209.9, 203.6, 199.5, 196.8, 109.4, 56.8, 52.3, 47.1, 26.0, 24.2, 23.8, 22.6;

**HRMS** (ESI) calcd for $C_{15}H_{23}O_4$ [(M+H)$^+$] Exact Mass: 267.1591; found: 267.1585.

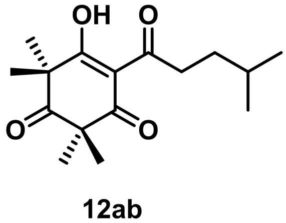

**12ab**

Compound **12ab**: 25.1 g, 88% yield, yellow oil;

**$R_f$** = 0.5 (hexane/ethyl acetate = 20/1);

**IR (film)** $\lambda_{max}$ 3445, 2959, 2874, 1721, 1670, 1558, 1470, 1420, 1385, 1049, 964;

**$^1$H NMR** (400 MHz, $CDCl_3$) δ 2.97 (m, 2H), 1.62 (m, 1H), 1.53 (m, 2H), 1.44 (s, 6H), 1.35 (s, 6H), 0.93 (d, $J$ = 1.9 Hz, 3H), 0.92 (d, $J$ = 1.9 Hz, 3H);

**$^{13}$C NMR** (100 MHz, $CDCl_3$) δ 210.0, 205.0, 199.0, 196.7, 109.0, 56.8, 52.1, 37.2, 34.0, 27.9, 24.3, 23.8, 22.3;

**HRMS** (ESI) calcd for $C_{16}H_{25}O_4$ [$(M+H)^+$] Exact Mass: 281.1747; found: 281.1743.

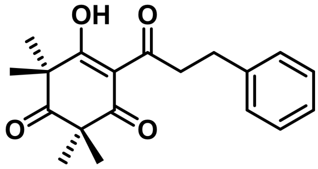

**12ac**

Compound **12ac**: 24.3 g, 76% yield, yellow oil;

**$R_f$** = 0.4 (hexane/ethyl acetate = 20/1);

**IR (film)** $\lambda_{max}$ 3433, 3028, 2982, 2940, 2874, 1721, 1670, 1558, 1459, 1385, 1049, 964, 698;

**$^1$H NMR** (500 MHz, $CDCl_3$) δ 7.29 (m, 4H), 7.23 (m, 1H), 3.36 (t, $J$ = 9.5, 2H), 3.01 (t, $J$ = 9.5, 2H), 1.48 (s, 6H), 1.36 (s, 6H);

**$^{13}$C NMR** (125 MHz, $CDCl_3$) δ 210.0, 203.7, 198.7, 196.7, 140.5, 128.5, 128.5, 126.3, 109.3, 56.9, 51.9, 41.0, 30.9, 24.4, 23.9;

**HRMS** (ESI) calcd for $C_{19}H_{23}O_4$ [$(M+H)^+$] Exact Mass: 315.1591; found: 315.1583.

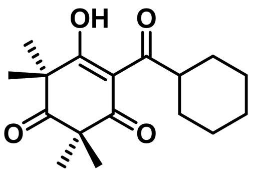

**12ad**

Compound **12ad**: 25.3 g, 85% yield, yellow oil;

**$R_f$** = 0.4 (hexane/ethyl acetate = 20/1);

**IR (film)** $\lambda_{max}$ 2982, 2936, 2855, 1721, 1670, 1543, 1458, 1361, 1223, 1049, 961, 891;

**$^1$H NMR** (500 MHz, $CDCl_3$) δ 3.52 (m, 1H), 1.81 (m, 4H), 1.74 (m, 1H), 1.48 (m, 1H), 1.44 (s,

6H), 1.37 (m, 3H), 1.37 (s, 6H), 1.24 (m, 1H);

**$^{13}$C NMR** (125 MHz, $CDCl_3$) δ 210.0, 207.3, 199.8, 196.8, 108.2, 57.0, 52.4, 45.1, 29.3, 25.8, 25.7, 24.3, 23.9;

**HRMS** (ESI) calcd for $C_{17}H_{25}O_4$ [$(M+H)^+$] Exact Mass: 293.1747; found: 293.1743.

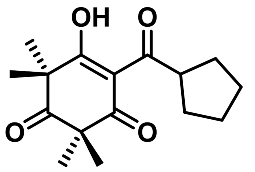

**12ae**

Compound **12ae**: 24.7 g, 87% yield, yellow oil;

**R$_f$** = 0.5 (hexane/ethyl acetate = 20/1);

**IR (film)** $\lambda_{max}$ 3433, 2974, 2947, 2871, 1721, 1674, 1558, 1458, 1381, 1230, 1049, 968;

**$^1$H NMR** (500 MHz, $CDCl_3$) δ 3.93 (m, 1H), 1.94 (m, 2H), 1.76 (m, 4H), 1.65 (m, 2H), 1.44 (s, 6H), 1.37 (s, 6H);

**$^{13}$C NMR** (125 MHz, $CDCl_3$) δ 210.0, 207.3, 198.7, 197.0, 108.8, 56.9, 52.1, 46.3, 30.5, 26.3, 24.2, 23.9, 23.9;

**HRMS** (ESI) calcd for $C_{16}H_{23}O_4$ [$(M+H)^+$] Exact Mass: 279.1591; found: 279.1584.

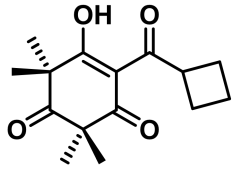

**12af**

Compound **12af**: 22.4 g, 83% yield, yellow crystals, mp = 45-47 $^o$C;

**R$_f$** = 0.5 (hexane/ethyl acetate = 20/1);

**IR (film)** $\lambda_{max}$ 2982, 2940, 2866, 1713, 1674, 1543, 1474, 1385, 1223, 1045, 931;

**$^1$H NMR** (500 MHz, $CDCl_3$) δ 4.19 (m, 1H), 2.29 (m, 4H), 2.04 (m, 1H), 1.87 (m, 1H), 1.46 (s, 6H), 1.35 (s, 6H);

**$^{13}$C NMR** (125 MHz, $CDCl_3$) δ 210.2, 205.2, 198.2, 196.5, 108.1, 56.8, 51.8, 42.5, 25.0, 24.4, 23.9, 17.8;

**HRMS** (ESI) calcd for $C_{15}H_{21}O_4$ [$(M+H)^+$] Exact Mass: 265.1434; found: 265.1429.

## 2.4 General procedure for the synthesis of 12 and 12a-12f

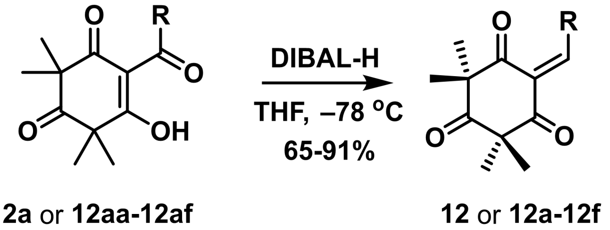


To a stirred cooled (−78 °C) solution of **12a** or **12aa-12af** (5 mmol) in tetrahydrofuran (THF; 25 mL) under argon was added diisobutylaluminum hydride (DIBAL-H; 7.5 mL, 1.5 equiv., 1.0 M in hexane). After the mixture was stirred for 30 min, it was quenched with water (50 mL) at –78 °C, and diluted with dichloromethane (100 mL) at 26 °C. The mixture was extracted with dichloromethane (3×100 mL). The combined organic phases were dried over $Na_2SO_4$, filtered, and concentrated in vacuum. The crude residue was purified by silica gel column chromatography (100% dichloroethane) to afford corresponding products **12** or **12a-12f**.

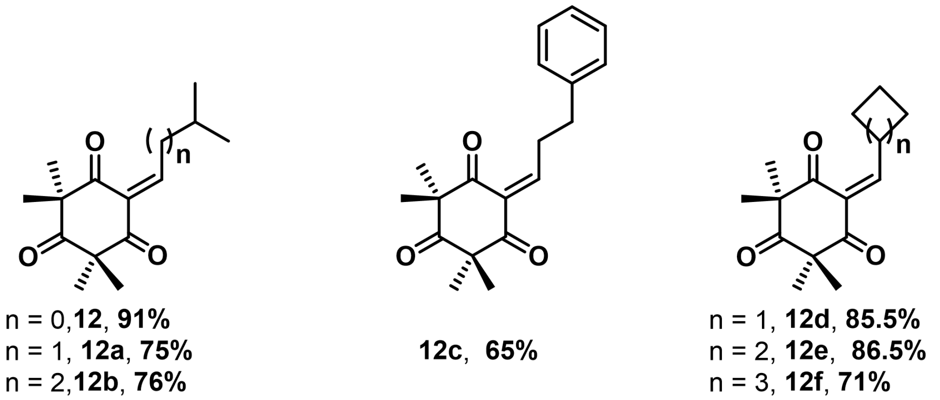


**Note**: compounds **12** and **12a-12f** are unstable as they can isomerize to the dienol and react with oxygen. For example, **12** can isomerize to the dienol **12'** and react with oxygen to afford endoperoxide **G3**.[6] According to this reason, **12** or **12a-12f** should be used as soon as possibly.

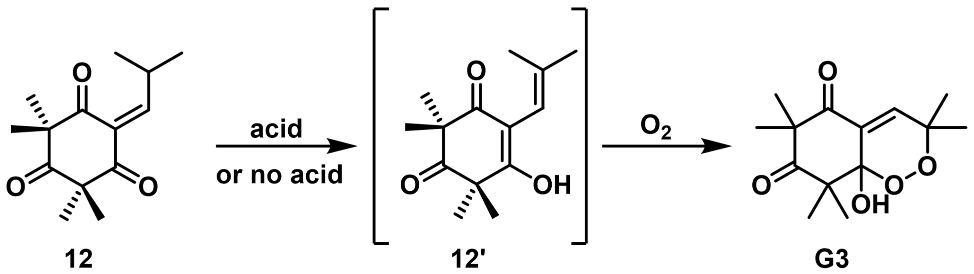


Compound **G3**: white crystals, mp = 145-147 °C;

**R$_f$** = 0.5 (hexane/ethyl acetate = 5/1);

**IR (film)** $\lambda_{max}$ 3503, 2982, 2936, 2874, 1717, 1686, 1636, 1466, 1350, 1288, 1096, 995;

**$^{1}$H NMR** (500 MHz, $CDCl_3$) δ 7.17 (m, 1H), 3.72 (s, 1H), 1.52 (m, 3H), 1.40 (s, 6H), 1.38 (s, 3H), 1.35 (s, 3H), 1.07 (s, 3H);

**$^{13}$C NMR** (125 MHz, $CDCl_3$) δ 210.7, 198.4, 143.1, 131.7, 97.4, 79.4, 55.0, 51.7, 26.6, 24.1, 23.9, 23.7, 20.9, 15.2;

**HRMS** (ESI) calcd for $C_{14}H_{21}O_5$ [$(M+H)^+$] Exact Mass: 269.1384; found: 269.1378.

## 2.5 Table S1. Optimization of reaction conditions for the Friedel–Crafts type Michael (FCM) additions[a]

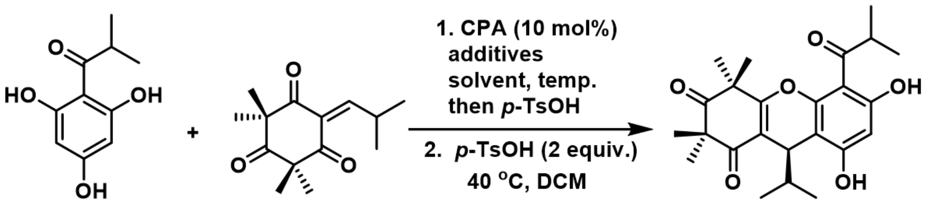


**11** + **12** → **13**

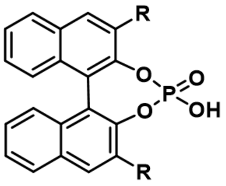

**(S)-C1: R = H2,4,6-(iPr)$_2$C$_6$H$_3$**
**(S)-C2: R = H**
**(S)-C3: R = 4-biphenyl**
**(S)-C4: R = 1-pyrenyl**
**(S)-C5: R = 9-anthryl**
**(S)-C6: R = triphenylsilyl**
**(R)-C7: R = 4-CF$_3$C$_6$H$_3$**
**(S)-C8: R = 3,5-(CF$_3$)$_2$C$_6$H$_3$**
**(S)-C9: R = 3,5-(NO$_2$)C$_6$H$_3$**

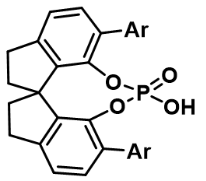

**(S)-C10: Ar = 4-tBu-C$_6$H$_4$**
**(S)-C11: Ar = 4-biphenyl**
**(S)-C12: Ar = 2,4,6-(iPr)$_2$C$_6$H$_3$**
**(S)-C13: Ar = 9-phenanthryl**
**(S)-C14: Ar = 4-Cl-C$_6$H$_4$**
**(S)-C15: Ar = 3,5-(CF$_3$)$_2$C$_6$H$_3$**
**(R)-C16: Ar = 3,5-(CF$_3$)$_2$C$_6$H$_3$**

| entry | catalyst | additive | *T* (°C) | solvent | time | yield (%)[b] | *er*[c] |
|---|---|---|---|---|---|---|---|
| 1 | (*S*)-**C1** | | –40 | toluene | 24 | 35 | 82.5:17.5 |
| 2 | (*S*)-**C2** | | –40-26 | toluene | 24 | 73 | 50:50 |
| 3 | (*S*)-**C3** | | –40 | toluene | 24 | trace | - |
| 4 | (*S*)-**C4** | | –40 | toluene | 24 | trace | - |
| 5 | (*S*)-**C5** | | –40 | toluene | 24 | 18 | 52.5:47.5 |
| 6 | (*S*)-**C6** | | –40 | toluene | 24 | 24 | 55:45 |
| 7 | (*R*)-**C7** | | –40 | toluene | 24 | 30 | 40:60 |
| 8 | (*S*)-**C8** | | –40 | toluene | 24 | 36 | 60:40 |
| 9 | (*S*)-**C9** | | –40 | toluene | 24 | trace | - |
| 10 | (*S*)-**C10** | | –40 | toluene | 24 | 23 | 62:38 |
| 11 | (*S*)-**C11** | | –40 | toluene | 24 | 21 | 0 |
| 12 | (*S*)-**C12** | | –40 | toluene | 24 | trace | - |
| 13 | (*S*)-**C13** | | –40 | toluene | 24 | 25 | 64:36 |
| 14 | (*S*)-**C14** | | –40 | toluene | 24 | 18 | 50:50 |
| 15 | (*S*)-**C15** | | –40 | toluene | 24 | 73 | 88:12 |
| 16 | (*S*)-**C15** | | –60 | toluene | 108 | 76 | 91:9 |
| 17 | (*S*)-**C15** | | –60 | DCM | 108 | 70 | 89.5:10.5 |
| 18 | (*S*)-**C15** | | –60 | CHCl$_3$ | 108 | 30 | 91:9 |
| 19 | (*S*)-**C15**[d] | | –60 | toluene | 108 | 74 | 92:8 |
| 20 | (*S*)-**C15**[d] | MgF$_2$ (10%) | –60 | toluene | 108 | 76 | 93:7 |
| 22 | (*S*)-C**15**[d] | Fe(OTf)$_3$ (10%) | –60 | toluene | 108 | 74 | 91:9 |
| 23 | (*S*)-**C15**[d] | Zn(OTf)$_2$ (10%) | –60 | toluene | 108 | 72 | 92.5:7.5 |
| 24 | (*S*)-**C15**[d] | AlF$_3$ (10%) | –60 | toluene | 72 | 71 | 93.5:6.5 |
| 25 | (*S*)-**C15**[d] | CaF$_2$ (10%) | –60 | toluene | 72 | 74 | 92.5:7.5 |
| 26 | (*S*)-**C15**[d] | AlF$_3$ (10%) | –76 | toluene | 7d | 55 | 95.5:4.5 |
| 27 | (*S*)-**C15**[e] | AlF$_3$ (10%) | –76 | toluene | 6d | 25 | 96.5:3.5 |
| 28 | (*S*)-**C15**[f] | AlF$_3$ (10%) | –76 | toluene | 6d | 28 | 96:4 |
| **29** | **(*S*)-C15[e,g]** | **AlF$_3$ (100%)** | **–70** | **toluene** | **6d** | **77** | **95:5** |
| **30** | **(*R*)-C16[e,g]** | **AlF$_3$ (100%)** | **–70** | **toluene** | **6d** | **76** | **5:95** |
| **31** | **(*S*)-C15[e,g]** | **AlF$_3$ (100%)** | **–70** | **toluene** | **6d** | **75** | **95:5(99.5:0.5)[h]** |
| **32** | **(*R*)-C16[e,g]** | **AlF$_3$ (100%)** | **–70** | **toluene** | **6d** | **75** | **5:95(0.5:99.5)[h]** |

[a]Unless otherwise stated, the reactions of entries 1-30 were carried out with **11** (0.1 mmol), **12** (0.2

mmol), **CPA** (0.01 mmol) in toluene (2 mL) at −76 °C to 26 °C. [b]Isolated yield. [c] Determined by chiral HPLC analysis with a ChiralCel OD-H column (*n*-hexane/*i*-propanol = 95:5, 0.8 mL/min). [d]4 Å MS (35 mg) was added. [e]3 Å MS (35 mg) was added. [f]5 Å MS (35 mg) was added. [g]The reactions of entries 31-32 were carried out with **11** (2.0 g, 10.2 mmol), **12** (20.4 mmol), (*S*)-**C15** or (*R*)-**C16** (1.02 mmol) and 3 Å MS (357 mg) in toluene (204 mL) at −70 °C. [f]The *er* value could be easily improved by recrystallization (up to 99.5:0.5).

**Note**:

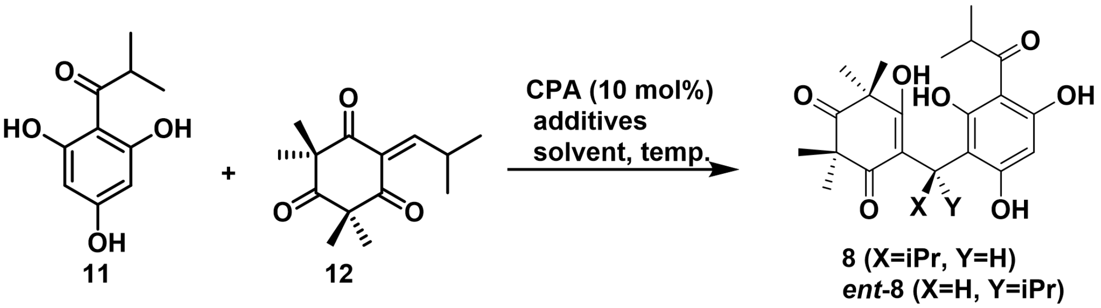

8 (X=iPr, Y=H)
*ent*-8 (X=H, Y=iPr)

First of all, in this reaction, both $^{1}$H and $^{13}$C NMR spectra of product **8** or ***ent*-8** showed doubled signal patterns, which was probably because of the presence of rotamers or keto-enol tautomers, second, as we would not separate **8** or ***ent*-8** into its enantiomers. So further cyclization of **8** or ***ent*-8** was carried out to afford specify **13** or ***ent*-13**. The *er* values of **13** and ***ent*-13** are equal to the *er* values of **8** and ***ent*-8** respectively[7,8].

Compound **8**: $[\alpha]_D^{26}$ = -77.8 (*c* = 0.2 in MeOH), (when the *er* value of **13** is 95:5);

Compound ***ent*-8**: $[\alpha]_D^{26}$ = +80.1 (*c* = 0.2 in MeOH), (when the *er* value of ***ent*-13** is 95:5);

**$R_f$** = 0.3 (hexane/ethyl acetate = 2/1);

**$^{1}$H NMR** (400 MHz, $CDCl_3$) δ 11.66 (d, *J* = 30.2 Hz, 1H), 10.44 (s, 1H), 6.83 (s, 1H), 5.98 (s, 1H), 3.96 (m, 1H), 3.80 (d, *J* = 10.8 Hz, 1H), 3.01 (m, 1H), 2.26 (s, 1H), 1.45 (s, 3H), 1.38 (s, 3H), 1.35 (s, 3H), 1.23 (t, *J* = 6.3 Hz, 6H), 0.87 (*J* = 6.4 Hz, 3H), 0.79 (d, *J* = 6.4 Hz, 3H);

**$^{13}$C NMR** (100 MHz, $CDCl_3$) δ 213.1, 211.1, 202.9, 178.3, 163.6, 163.5, 158.7, 114.20, 109.0, 102.8, 98.0, 54.9, 48.8, 39.1, 39.0, 26.5, 26.0, 25.9, 24.8, 23.4, 22.0, 21.8, 19.3, 19.3;

**HRMS** (ESI) calcd for $C_{24}H_{33}O_7$ [$(M+H)^+$] Exact Mass: 433.2221; found: 433.2216.

### 2. 6 Table S2. Substrate Scope of the Organocatalytic Enantioselective FCM additions[a,b]

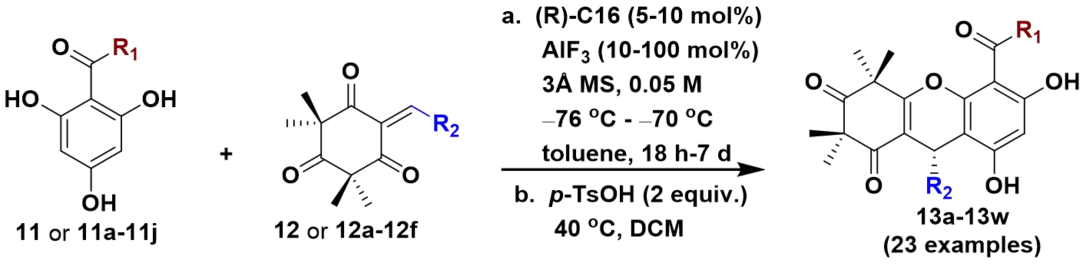


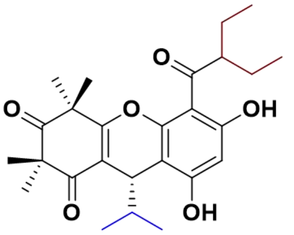

[c]13a: 76%, 93.5:6.5 *er*, 4 d

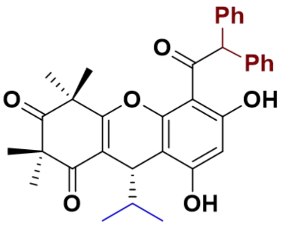

[d]13b: 73%, 91:9 *er*, 2 d

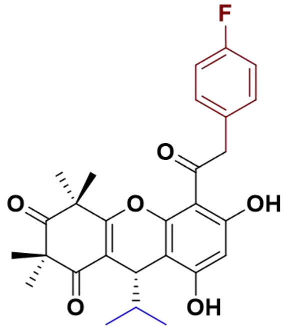

13c: 62%, 92.5:7.5 *er*, 7 d
(98.5:1.5 *er*)[e]

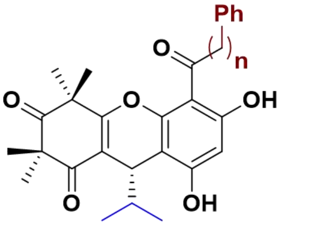

13d: n=2, 69%, 93.5:6.5 *er*, 6 d
13e: n=1, 70%, 94.5:5.5 *er*, 6 d
(99:1 *er*)[e]

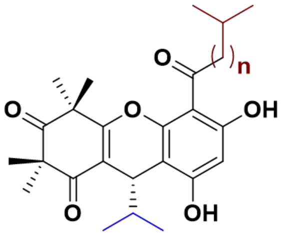

13f: n=1, 72%, 94:6 *er*, 6 d
13g: n=2, 75%, 94:6 *er*, 6 d
(99.5:0.5 *er*)[e]

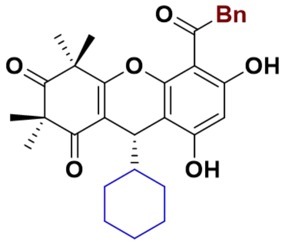

13h: 69%, 95.5:4.5 *er*, 5 d
(99:1 *er*)[e]

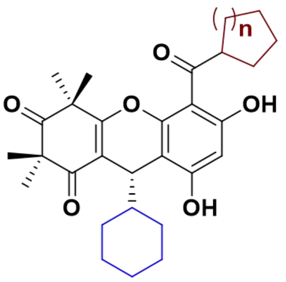

13i: n=2, 73%, 97:3 *er*, 5 d
(99:1 *er*)[e]
13j: n=1, 73%, 94:6 *er*, 6 d
(99:1 *er*)[e]

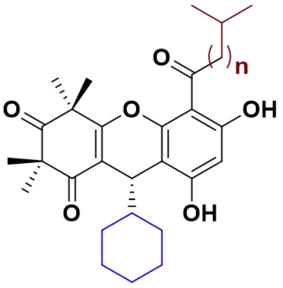

13k: n=0, 75%, 93:7 *er*, 6 d
13l: n=1, 72%, 96.5:3.5 *er*, 6 d
(99.5:0.5 *er*)[e]
13m: n=2, 72%, 97:3 *er*, 6 d
(99:1 *er*)[e]

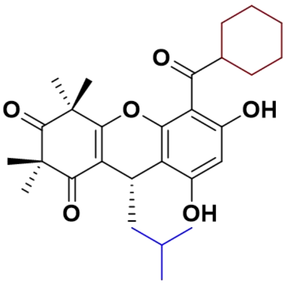

13n: 55%, 90.5:9.5 *er*, 3 d

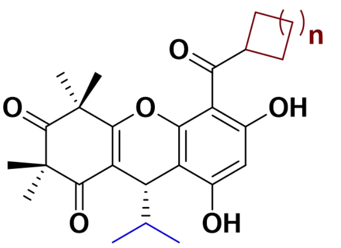

13o: n=3, 73%, 95:5 *er*, 5 d (99.5:0.5 *er*)[e]
13p: n=2, 74%, 94.5:5.5 *er* 6d, (99:1 *er*)[e]
13q: n=1, 71%, 93:7 *er*, 6 d (99.5:0.5 *er*)[e]

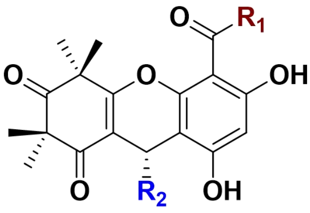

13r: $R_1$=$R_2$=$CH_2CH(CH_3)_2$ 51%, 92:8 *er*, 3 d
13s: $R_1$=$R_2$=$CH_2CH_2CH(CH_3)_2$ 70%, 93.5:6.5 *er*, 3 d (99.8:0.2 *er*)[e]
[f]13t: $R_1$=$R_2$=$CH_2Bn$, 74% 95:5 *er*, 5 d

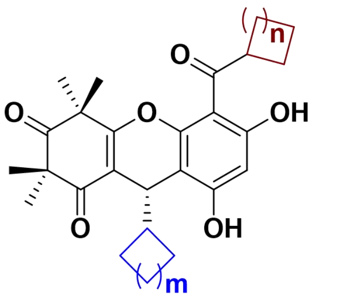

13u: n=m=2, 73%, 92.5:7.5 *er* 4 d, (99.8:0.2 *er*)[e]
13v: m=2, n=3, 76%, 94:6 *er* 4 d, (99:1 *er*)[e]
13w: n=m=1, 50%, 89:11 *er* 18 h

[a]Unless otherwise noted, reaction conditions: **11** or **11a-11j** (0.1 mmol), **12** or **12a-12f** (0.2 mmol), $AlF_3$(100 mol%), 3 Å MS (35 mg) and (*R*)-**C16** (10 mol%) in toluene (2 mL) for 18 h-7 d at −70 °C. [b]Isolated yield and enantiomeric excesses were determined by HPLC analysis. [c] reaction condition: **11a** (0.1 mmol), **12** (0.2 mmol), $AlF_3$(10 mol%), 3 Å MS (35 mg) and (*R*)-**C16** (10 mol%) in toluene (2 mL) for 5 d at −76 °C. [d] reaction condition: **11b** (0.1 mmol), **12** (0.2 mmol), $AlF_3$(10 mol%), 3 Å MS (35 mg) and (*R*)-**C16** (5 mol%) in toluene (2 mL) for 3 d at −76 °C. [e]These compounds (**13c**, **13e**, **13g**, **13h**, **13i**, **13j**, **13l**, **13m**, **13o**, **13p**, **13q**, **13t**, **13u, 13v**) were easily re-crystallised from *n*-hexane containing a few drops of $CH_2Cl_2$. [f]reaction condition: **11e** (0.1 mmol), **12c** (0.4 mmol), $AlF_3$(100 mol%), 3 Å MS (35 mg) and (*R*)-**C16** (10 mol%) in toluene (2 mL) for 3 d at −76 °C.

### 2.7 Synthesis of 13, *ent*-13 and 13a-13w

**Procedure A: General procedure for the synthesis of racemic 13 and 13a-13w.**

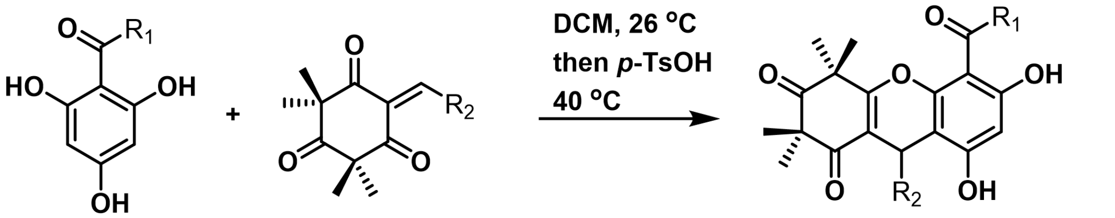


**11** or **11a-11j** **12** or **12a-12f** ***rac*-13** or ***rac*-13a-13w**

To a solution of compound **11** or **11a-11j** (1 equiv.) in dichloromethane (DCM) under argon was added compound **12** or **12a-12f** (2-4 equiv.) and then stirred at 26 °C. After 5 h,

*p*-toluenesulfonic acid (*p*-TsOH; 2 equiv.) was added and the resulting mixture was stirred at 40 °C. After the reaction was finished according to TLC, the mixture was quenched with saturated aqueous sodium bicarbonate and extracted with dichloromethane. Then the combined organic layers were dried over $Na_2SO_4$, filtered and concentrated in vacuo. The crude residue was purified by silica gel column chromatography (2%-20% hexanes/ethyl acetate) to afford the corresponding products *rac*-**13** or *rac*-**13a-13w**.

**Procedure B: General procedure for the synthesis of enantioenriched products 13, *ent*-13 and 13a-13w.**

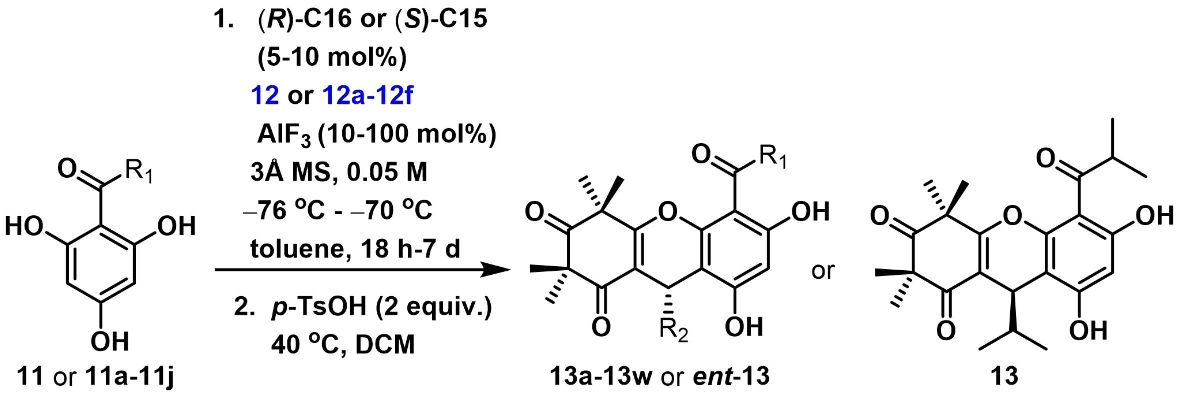


(11*aS*)-3,7-Bis[3,5-bis(trifluoromethyl)phenyl]-10,11,12,13-tetrahydro-5-hydroxy-diindeno[7,1-de:1',7'-fg][1,3,2]dioxaphosphocin 5-oxide [(*R*)-**C16** or (*S*)-**C15**; 5-10 mol%], aluminum fluoride ($AlF_3$; 10-100 mol%) under argon were added to a solution of Compound **11** or **11a-11j** (1 equiv.) and 3 Å MS in toluene. The resulting mixture was stirred for 30 min at 26 °C and cooled down to −76 °C - −70 °C. Compound **12** or **12a-12f** (2-4 equiv.) was added. After starting material was consumed (TLC), the mixture was directly purified by silica gel column chromatography (2.5%-20% hexanes/ethyl acetate) to afford the crude residue. Then to a solution of the crude residue was added *p*-toluenesulfonic acid (*p*-TsOH; 2 equiv.) in dichloromethane (DCM) then stirred at 40 °C. After the reaction was finished according to TLC, the mixture was quenched with saturated aqueous sodium bicarbonate and extracted with ethyl acetate. Then the combined organic layers were dried over $Na_2SO_4$, filtered and concentrated in vacuo. The crude residue was purified by silica gel column chromatography (2%-20% hexanes/ethyl acetate) to afford corresponding chiral substitution products **13** or ***ent*-13** or **13a-13w**.

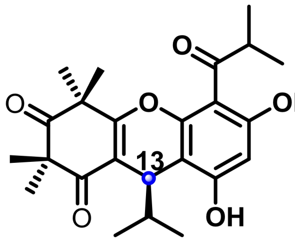

**13**

Compound **13**: 3.2 g, 75% yield, 6 d, white crystals, mp = 158-160 °C, after recrystallization: 2.2 g; according to procedure B;

**R$_f$**= 0.45 (hexane/ethyl acetate = 4/1);

$[\alpha]_D^{25}$ = +208 (*c* = 0.2 in $CHCl_3$);

Compound ***ent*-13**: 3.2 g, 75% yield, 6 d, white crystals, after recrystallization: 2.1 g; according to procedure B;

$[\alpha]_D^{25}$ = -204 (*c* = 0.2 in $CHCl_3$);

**IR (film)** $\lambda_{max}$ 3240, 2969, 2930, 2870, 1719, 1624, 1591, 1396, 1383, 1229, 1155, 1001, 833;

**$^1$H NMR** (500 MHz, $CDCl_3$) $\delta$ 13.41 (s, 1H), 7.57 (s, 1H), 6.33 (s, 1H), 4.40 (d, *J* = 3.6 Hz, 1H), 4.05 – 3.74 (m, 1H), 2.00 – 1.90 (m, 1H), 1.64 (s, 3H), 1.48 (s, 3H), 1.44 (s, 3H), 1.42 (s, 3H), 1.28 (d, *J* = 4.9 Hz, 3H), 1.26 (d, *J* = 5.5 Hz, 3H), 0.86 (d, *J* = 6.9 Hz, 3H), 0.82 (d, *J* = 6.9 Hz, 3H);

**$^{13}$C NMR** (125 MHz, $CDCl_3$) $\delta$ 211.8, 208.9, 199.0, 168.3, 164.8, 159.8, 153.4, 112.1, 103.8, 103.7, 100.6, 56.2, 47.3 39.7, 34.8, 31.4, 25.2, 25.0, 25.0, 24.2, 20.9, 18.9, 18.7, 17.7;

**HRMS** (ESI) calcd for $C_{24}H_{31}O_6$ [$(M+H)^+$] Exact Mass: 415.2115; found: 415.2107;

**HPLC condition of 13**: Daicel Chiralpak OD-H column; *n*-hexane/*i*-propanol = 95:5, 0.8 mL/min, $\lambda$ = 280 nm; major enantiomer: $t_R$ = 15.9 min, minor enantiomer: $t_R$ = 9.2 min. 95:5 *er*; re-crystallised: 99.7: 0.3 *er*;

**HPLC condition of *ent*-13**: ChiralCel OD-H column; *n*-hexane/*i*-propanol = 95:5, 0.8 mL/min, $\lambda$ = 280 nm; major enantiomer: $t_R$ = 8.6 min, minor enantiomer: $t_R$ = 16.9 min. 95:5 *er*; re-crystallised: > 99.5:0.5 *er*.

**Note**: (1). The C-13 absolute configurations of ***ent*-13** and **13** were determined to be respectively *R* and *S* based on the reference[7].

(2). The recoverable compound (*S*)-C**15** and (*R*)-C**16** can be reused several times without significant loss of activity. The *er* values of **13** and ***ent*-13** were 95:5.

(3). According to procedure B, a total of 10.0 g of both **13** and ***ent*-13** was prepared readily after 5 simple parallel operations.

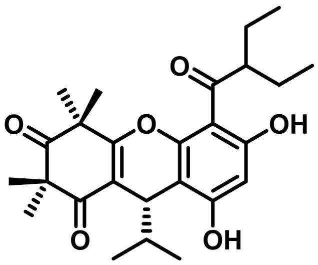

**13a**

Compound **13a**: 34 mg, 76% yield, 4 d, white crystals, mp = 202-204 °C; according to procedure B;

**$R_f$**= 0.45 (hexane/ethyl acetate = 4/1);

$[\alpha]_D^{27}$ = -88.2 ($c$ = 0.2 in MeOH);

**IR (film)** $\lambda_{max}$ 3210, 2963, 2936, 2874, 1721, 1636, 1500, 1462, 1389, 1250, 1153, 1034, 968, 840;

**$^1$H NMR** (500 MHz, $CDCl_3$) $\delta$ 13.50 (s, 1H), 7.10 (s, 1H), 6.33 (s, 1H), 4.38 (d, $J$ = 3.5 Hz, 1H), 3.97 – 3.81 (m, 1H), 2.00 – 1.88 (m, 2H), 1.79 – 1.69 (m, 2H), 1.67 (s, 3H), 1.61 (td, $J$ = 13.8, 6.8 Hz, 1H), 1.48 (s, 3H), 1.46 (s, 3H), 1.41 (s, 3H), 1.00 (t, $J$ = 7.4 Hz, 3H), 0.85 (d, $J$ = 7.1 Hz, 3H), 0.84 (d, $J$ = 7.5 Hz, 3H), 0.81 (d, $J$ = 6.9 Hz, 3H);

**$^{13}$C NMR** (125 MHz, $CDCl_3$) δ 211.8, 208.6, 198.7, 168.2, 164.5, 159.5, 153.6, 112.2, 105.3, 103.7, 100.6, 56.2, 52.3, 47.4, 34.8, 31.5, 26.1, 25.2, 25.1, 25.0, 24.1, 22.1, 18.9, 18.6, 12.2, 10.5;

**HRMS** (ESI) calcd for $C_{26}H_{35}O_6$ [$(M+H)^+$] Exact Mass: 443.2428; found: 443.2417;

**HPLC condition**: ChiralCel OD-H column; $n$-hexane/$i$-propanol = 90:10, 1 mL/min, λ = 280 nm; major enantiomer: $t_R$ = 4.6 min, minor enantiomer: $t_R$ = 6.7 min. 93.5:6.5 *er*.

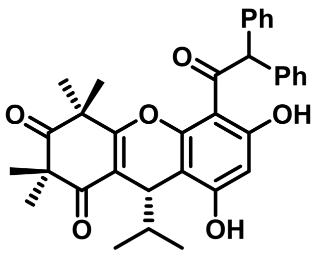

**13b**

Compound **13b**: 39 mg, 73% yield, 2 d, white crystals, mp = 191-193 °C; according to procedure B;

**$R_f$**= 0.28 (hexane/ethyl acetate = 4/1);

$[\alpha]_D^{27}$ = -83.6 ($c$ = 0.2 in MeOH);

**IR (film)** $\lambda_{max}$ 3244, 2986, 2955, 2870, 1717, 1628, 1585, 1496, 1392, 1230, 1157, 1007, 826;

**$^1$H NMR** (500 MHz, $CDCl_3$) δ 12.78 (s, 1H), 8.09 (s, 1H), 7.34 (t, $J$ = 7.4 Hz, 2H), 7.25 (ddd, $J$ = 21.6, 9.6, 4.7 Hz, 4H), 7.17 (d, $J$ = 7.4 Hz, 4H), 6.55 (s, 1H), 6.31 (s, 1H), 4.34 (d, $J$ = 3.5 Hz, 1H), 1.76 (m, 1H), 1.66 (s, 3H), 1.45 (s, 3H), 1.37 (s, 3H), 1.35 (s, 3H), 0.58 (t, $J$ = 6.5 Hz, 6H);

**$^{13}$C NMR** (125 MHz, $CDCl_3$) δ 211.6, 201.3, 199.7, 168.6, 164.6, 163.1, 161.1, 160.8, 153.7, 131.4, 131.4, 129.8, 129.8, 115.6, 115.5, 112.5, 104.6, 104.4, 100.6, 56.2, 49.4, 47.5, 34.8, 31.5, 25.5, 25.4, 25.3, 24.0, 19.0, 18.6;

**HRMS** (ESI) calcd for $C_{34}H_{35}O_6$ [$(M+H)^+$] Exact Mass: 539.2428; found: 539.2426;

**HPLC condition**: ChiralCel OD-H column; $n$-hexane/$i$-propanol = 90:10, 1 mL/min, λ = 280 nm; major enantiomer: $t_R$ = 6.7 min, minor enantiomer: $t_R$ = 8.3 min. 91:9 *er*.

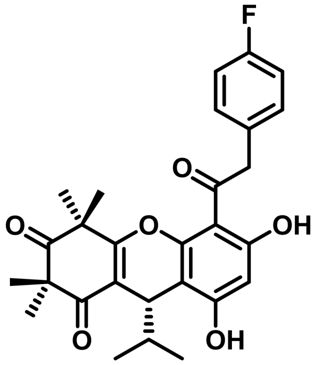

**13c**

Compound **13c**: 29.8 mg, 62% yield, 7 d, white crystals, mp = 204-205 °C, after recrystallization: 11.9 mg; according to procedure B;

**$R_f$**= 0.35 (hexane/ethyl acetate = 4/1);

$[\alpha]_D^{27}$ = -113.5 ($c$ = 0.2 in MeOH);

**IR (film)** $\lambda_{max}$ 3321, 2963, 2935, 2874, 1717, 1655, 1636, 1525, 1508, 1466, 1350, 1254, 1157, 1007, 972, 837;

**$^1$H NMR** (500 MHz, $CDCl_3$) δ 13.20 (s, 1H), 8.30 (s, 1H), 7.23 (dd, $J$ = 8.3, 5.4 Hz, 2H), 7.06 (t, $J$ = 8.6 Hz, 2H), 6.37 (s, 1H), 4.62 (d, $J$ = 16.9 Hz, 1H), 4.46 (d, $J$ = 3.7 Hz, 1H), 4.34 (d, $J$ = 16.9 Hz, 1H), 1.94 (m, 1H), 1.70 (s, 3H), 1.52 (d, $J$ = 9.9 Hz, 6H), 1.44 (s, 3H), 0.86 (d, $J$ = 6.8 Hz, 3H), 0.83 (d, $J$ = 6.9 Hz, 3H);

**$^{13}$C NMR** (125 MHz, $CDCl_3$) δ 211.6, 201.3, 199.7, 168.6, 164.6, 163.1, 161.1, 160.8, 153.7, 131.4, 131.4, 129.8, 129.8, 115.6, 115.5, 112.5, 104.6, 104.4, 100.5, 56.2, 49.4, 47.5, 34.8, 31.5,

25.5, 25.4, 25.3, 24.0, 19.0, 18.6;

**HRMS** (ESI) calcd for $C_{28}H_{30}O_6F[(M+H)^+]$ Exact Mass: 481.2021; found:481.2019;

**HPLC condition**: ChiralCel OD-H column; *n*-hexane/*i*-propanol = 90:10, 1 mL/min, λ = 280 nm; major enantiomer: $t_R$ = 6.8 min, minor enantiomer: $t_R$ = 9.5 min. 92.5:7.5 *er*, re-crystallised: 98.5:1.5 *er*.

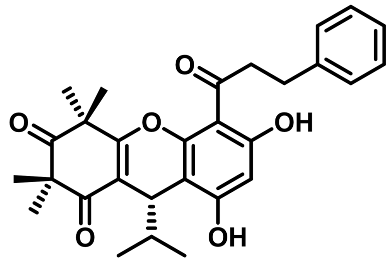


**13d**

Compound **13d**: 32.8 mg, 69% yield, 6 d, white crystals, mp = 177-178 °C; according to procedure B;

**R$_f$**= 0.36 (hexane/ethyl acetate = 4/1);

$[\alpha]_D^{27}$ = -83.4 (*c* = 0.2 in MeOH);

**IR (film)** $\lambda_{max}$ 3495, 3140, 3089, 2963, 2936, 2870, 1721, 1636, 1593, 1508, 1435, 1393, 1246, 1161, 1041, 833;

**$^1$H NMR** (500 MHz, MeOD) δ 7.27 (dt, *J* = 14.9, 7.5 Hz, 4H), 7.19 (t, *J* = 7.1 Hz, 1H), 6.22 (s, 1H), 4.25 (d, *J* = 3.5 Hz, 1H), 3.53 (m, 1H), 3.43 (m, 1H), 3.09 (m, 1H), 2.99 (m, 1H), 1.87 (m, 1H), 1.36 (s, 6H), 1.32 (d, *J* = 2.0 Hz, 6H), 0.78 (d, *J* = 6.9 Hz, 3H), 0.76 (d, *J* = 6.9 Hz, 3H);

**$^{13}$C NMR** (125 MHz, MeOD) δ 211.7, 202.8, 198.2, 168.0, 163.7, 161.4, 153.8, 141.3, 128.2, 128.1, 125.8, 111.8, 104.0, 103.9, 99.0, 55.7, 47.1, 46.8, 34.6, 31.3, 30.0, 24.2, 24.0, 24.0, 23.1, 18.3, 17.6;

**HRMS** (ESI) calcd for $C_{29}H_{33}O_6$ $[(M+H)^+]$ Exact Mass: 477.2272; found: 477.2272;

**HPLC condition**: ChiralCel OD-H column; *n*-hexane/*i*-propanol = 90:10, 1 mL/min, λ = 280 nm; major enantiomer: $t_R$ = 5.9 min, minor enantiomer: $t_R$ = 9.4 min. 93.5:6.5 *er*.

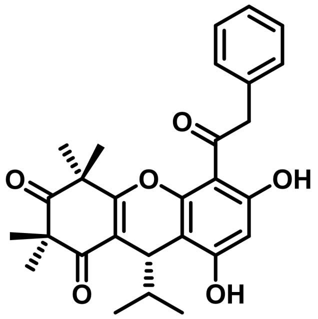

**13e**

Compound **13e**: 32.7 mg, 70% yield, 6 d, white crystals, mp = 182-184 °C, after recrystallization: 9.8 mg; according to procedure B;

$\mathbf{R_f}$= 0.35 (hexane/ethyl acetate = 4/1);

$[\alpha]_D^{25}$ = -156.3 ($c$ = 0.2 in MeOH);

**IR (film)** $\lambda_{max}$ 3275, 3163, 3089, 2963, 2932, 2870, 1709, 1632, 1597, 1501, 1466, 1383, 1261, 1007, 841;

$^1$**H NMR** (500 MHz, MeOD) δ 7.27 (d, $J$ = 6.6 Hz, 2H), 7.22 (d, $J$ = 6.6 Hz, 3H), 6.24 (s, 1H), 4.60 (d, $J$ = 15.7 Hz, 1H), 4.30 (d, $J$ = 15.7 Hz, 1H), 4.26 (d, $J$ = 3.7 Hz, 1H), 1.87 (m, 1H), 1.59 (s, 3H), 1.43 (s, 3H), 1.39 (s, 3H), 1.34 (s, 3H), 0.77 (d, $J$ = 7.3 Hz, 3H), 0.75 (d, $J$ = 7.3 Hz, 3H);

$^{13}$**C NMR** (125 MHz, MeOD) δ 211.7, 201.5, 198.2, 168.2, 162.4, 161.0, 152.9, 134.9, 129.4, 128.1, 126.5, 111.8, 104.8, 104.1, 98.9, 55.7, 50.0, 47.2, 34.6, 31.4, 24.4, 24.2, 24.1, 23.0, 18.3, 17.7;

**HRMS** (ESI) calcd for $C_{28}H_{31}O_6$ $[(M+H)^+]$ Exact Mass: 469.2585; found: 469.2577;

**HPLC condition**: ChiralCel OD-H column; $n$-hexane/$i$-propanol = 90:10, 1 mL/min, λ = 280 nm; major enantiomer: $t_R$ = 7.5 min, minor enantiomer: $t_R$ = 9.9 min. 94.5:5.5 *er*, re-crystallised: 99:1 *er*.

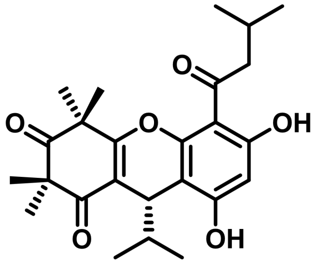

**13f**

Compound **13f**: 72% yield, 6 d, white crystals, mp = 157-159 °C; according to procedure B;

$R_f$= 0.4 (hexane/ethyl acetate = 4/1);

$[\alpha]_D^{25}$ = -63.0 (*c* = 0.2 in MeOH);

**IR (film)** $\lambda_{max}$ 3256, 2959, 2932, 2870, 1717, 1643, 1593, 1504, 1466, 1392, 1250, 1161, 829;

**$^1$H NMR** (500 MHz, $CDCl_3$) δ 13.49 (s, 1H), 7.91 (s, 1H), 6.36 (s, 1H), 4.42 (d, *J* = 3.4 Hz, 1H), 3.21 (dd, *J* = 16.7, 7.4 Hz, 1H), 2.92 (dd, *J* = 16.7, 6.3 Hz, 1H), 2.39 – 2.33 (m, 1H), 1.94 – 1.91 (m, 1H), 1.69 (s, 3H), 1.48 (d, *J* = 7.0 Hz, 6H), 1.42 (s, 3H), 1.04 (d, *J* = 6.6 Hz, 3H), 1.01 (d, *J* = 6.6 Hz, 3H), 0.84 (d, *J* = 6.8 Hz, 3H), 0.80 (d, *J* = 6.8 Hz, 3H);

**$^{13}$C NMR** (125 MHz, $CDCl_3$) δ 211.8, 204.0, 199.4, 168.6, 164.3, 160.1, 153.7, 112.2, 105.2, 104.0, 100.4, 56.2, 53.3, 47.4, 34.9, 31.4, 25.3, 25.1, 25.1, 24.8, 24.1, 22.9, 22.7, 19.0, 18.6;

**HRMS** (ESI) calcd for $C_{25}H_{33}O_6$ [$(M+H)^+$] Exact Mass: 429.2272; found: 429.2269;

**HPLC condition**: ChiralCel OD-H column; *n*-hexane/*i*-propanol = 90:10, 1 mL/min, λ = 280 nm; major enantiomer: $t_R$ = 4.6 min, minor enantiomer: $t_R$ = 6.8 min. 94:6 *er*.

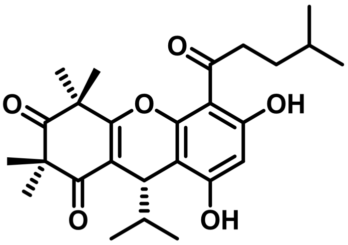


**13g**

Compound **13g:** 33.1 mg, 75% yield, 6 d, white crystals, mp = 197-198 °C, after recrystallization: 15.5 mg; according to procedure B;

$R_f$= 0.43 (hexane/ethyl acetate = 4/1);

$[\alpha]_D^{27}$ = -67.0 (*c* = 0.2 in MeOH);

**IR (film)** $\lambda_{max}$ 3448, 2955, 2936, 2870, 1720, 1639, 1589, 1508, 1435, 1393, 1242, 1099, 833;

**$^1$H NMR** (500 MHz, $CDCl_3$) δ 13.49 (s, 1H), 8.00 (s, 1H), 6.36 (s, 1H), 4.42 (d, *J* = 3.7 Hz, 1H), 3.30 (ddd, *J* = 17.2, 9.5, 5.2 Hz, 1H), 3.06 (ddd, *J* = 17.1, 9.3, 5.4 Hz, 1H), 1.96 – 1.88 (m, 1H), 1.75 – 1.70 (m, 1H), 1.69 (s, 3H), 1.68 – 1.60 (m, 2H), 1.48 (d, *J* = 2.8 Hz, 6H), 1.42 (s, 3H), 0.96 (t, *J* = 8.8 Hz, 6H), 0.84 (d, *J* = 6.9 Hz, 3H), 0.80 (d, *J* = 6.9 Hz, 3H);

**$^{13}$C NMR** (125 MHz, $CDCl_3$) δ 211.9, 204.7, 199.6, 168.7, 164.2, 160.3, 153.7, 112.3, 104.9, 104.0, 100.4, 56.2, 47.4, 42.5, 34.9, 33.2, 31.4, 27.7, 25.3, 25.2, 25.1, 24.2, 22.7, 22.6, 18.9, 18.6;

**HRMS** (ESI) calcd for $C_{26}H_{35}O_6$ [$(M+H)^+$] Exact Mass: 443.2428; found: 443.2419;

**HPLC condition**: ChiralCel OD-H column; *n*-hexane/*i*-propanol = 90:10, 1 mL/min, λ = 280 nm; major enantiomer: $t_R$ = 4.5 min, minor enantiomer: $t_R$ = 6.7 min. 94:6 *er*, re-crystallised: 99.5:0.5 *er*.

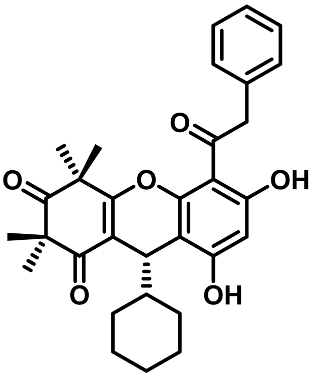


**13h**

Compound **13h**: 34.6 mg, 69% yield, 5 d, white crystals, mp = 250-252 °C, after recrystallization: 16.6 mg; according to procedure B;

$\mathbf{R_f}$= 0.38 (hexane/ethyl acetate = 4/1);

$[\alpha]_D^{27}$ = -41.6 (*c* = 0.2 in MeOH);

**IR (film)** $\lambda_{max}$ 3279, 2982, 2928, 2851, 1717, 1632, 1504, 1454, 1389, 1254, 1153, 1042, 972, 841;

**$^1$H NMR** (500 MHz, $CDCl_3$) δ 13.15 (s, 1H), 8.09 (s, 1H), 7.28 (t, *J* = 7.3 Hz, 2H), 7.22 (m, 1H), 7.18 (dd, *J* = 5.3, 3.8 Hz, 2H), 6.25 (s, 1H), 4.58 (d, *J* = 16.6 Hz, 1H), 4.30 (d, *J* = 3.8 Hz, 1H), 4.25 (d, *J* = 16.6 Hz, 1H), 1.60 (s, 3H), 1.53 (m, 4H), 1.42 (s, 3H), 1.41 (s, 3H), 1.33 (s, 3H), 1.03 (m, 2H), 0.87 (m, 2H), 0.72 (m, 1H);

**$^{13}$C NMR** (125 MHz, $CDCl_3$) δ 211.7, 201.6, 199.5, 199.5, 168.6, 164.5, 160.6, 160.6, 153.8, 134.3, 129.8, 128.6, 127.3, 112.6, 104.7, 104.4, 100.5, 56.2, 50.3, 47.5, 45.1, 31.2, 29.4, 29.1, 26.6, 26.5, 26.3, 25.5, 25.4, 25.1, 24.1;

**HRMS** (ESI) calcd for $C_{31}H_{35}O_6$ [$(M+H)^+$] Exact Mass: 503.2428; found: 503.2422;

**HPLC condition**: ChiralCel IE-3 column; *n*-hexane/*i*-propanol = 95:5, 1 mL/min, λ = 280 nm; major enantiomer: $t_R$ = 12.0 min, minor enantiomer: $t_R$ = 8.9 min. 95.5:4.5 *er*, re-crystallised: 99:1 *er*.

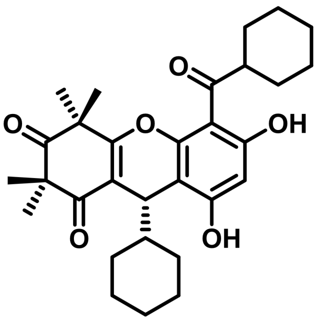

**13i**

Compound **13i**: 36.1 mg, 73% yield, 5 d, white crystals, mp = 177-179 °C, after recrystallization: 21.7 mg; according to procedure B;

**R$_f$**= 0.46 (hexane/ethyl acetate = 4/1);

$[\alpha]_D^{27}$ = -120.4 (*c* = 0.2 in MeOH);

**IR (film)** $\lambda_{max}$ 3248, 2986, 2923, 2855, 1717, 1624, 1597, 1504, 1427, 1392, 1250, 1157, 1034, 964, 841;

**$^1$H NMR** (400 MHz, $CDCl_3$) δ 13.21 (s, 1H), 7.88 (s, 1H), 6.35 (s, 1H), 4.37 (d, *J* = 3.8 Hz, 1H), 3.76 (m, 1H), 1.95 (m, 3H), 1.83 (m, 1H), 1.76 (m, 1H), 1.72 (s, 3H), 1.66 (m, 4H), 1.56 (m, 2H), 1.50 (s, 3H), 1.44 (s, 3H), 1.41 (s, 3H), 1.34 (m, 4H), 1.13 (m, 2H), 1.01 (m, 2H), 0.85 (m, 2H);

**$^{13}$C NMR** (100 MHz, $CDCl_3$) δ 211.8, 208.4, 199.2, 168.7, 164.3, 159.7, 153.6, 112.6, 104.2, 104.1, 100.6, 56.3, 49.3, 47.4, 45.1, 31.8, 31.3, 29.3, 29.2, 27.4, 26.6, 26.5, 26.4, 26.3, 25.9, 25.6, 25.4, 25.1, 24.9, 24.1;

**HRMS** (ESI) calcd for $C_{30}H_{39}O_6$ [$(M+H)^+$] Exact Mass: 495.2741; found: 495.2728;

**HPLC condition**: ChiralCel IE-3 column; *n*-hexane/*i*-propanol = 95:5, 1 mL/min, λ = 280 nm; major enantiomer: $t_R$ = 9.2 min, minor enantiomer: $t_R$ = 7.0 min. 97:3 *er*, re-crystallised: 99:1 *er*.

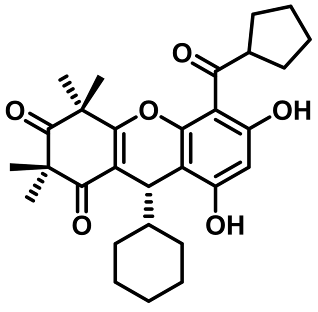

**13j**

Compound **13j**: 35.0 mg, 73% yield, 6 d, white crystals, mp = 208-209 °C, after recrystallization: 15.7 mg; according to procedure B;

**$R_f$**= 0.42 (hexane/ethyl acetate = 4/1);

$[\alpha]_D^{27}$ = -133.4 (*c* = 0.2 in MeOH);

**IR (film)** $\lambda_{max}$ 3244, 2978, 2932, 2847, 1717, 1637, 1589, 1504, 1393, 1254, 1165, 1006, 841;

**$^1$H NMR** (500 MHz, $CDCl_3$) δ 13.43 (s, 1H), 7.60 (s, 1H), 6.34 (s, 1H), 4.37 (d, *J* = 3.7 Hz, 1H), 4.09 (m, 1H), 2.24 (m, 1H), 2.11 (m, 1H), 1.82 (m, 2H), 1.70 (m, 8H), 1.64 (s, 3H), 1.56 (m, 2H), 1.49 (s, 3H), 1.44 (s, 3H), 1.41 (s, 3H), 1.13 (m, 2H), 0.98 (m, 2H), 0.82 (m, 1H);

**$^{13}$C NMR** (125 MHz, $CDCl_3$) δ 211.8, 207.5, 199.1, 168.4, 164.5, 159.7, 153.6, 112.4, 104.5, 104.0, 100.5, 56.2, 50.9, 47.3, 45.1, 32.3, 31.3, 29.4, 29.1, 27.8, 26.6, 26.5, 26.3, 26.0, 26.0, 25.2, 25.0, 25.0, 24.1;

**HRMS** (ESI) calcd for $C_{29}H_{37}O_6$ [$(M+H)^+$] Exact Mass: 481.2585; found: 481.2568;

**HPLC condition**: ChiralCel IE-3 column; *n*-hexane/*i*-propanol = 95:5, 1 mL/min, λ = 280 nm; major enantiomer: $t_R$ = 8.5 min, minor enantiomer: $t_R$ = 6.7 min. 94:6 er, re-crystallised: 99:1 *er*.

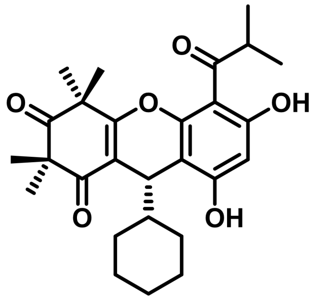

**13k**

Compound **13k**: 34.1 mg, 75% yield, 6 d, white crystals, mp = 230-232 °C; according to procedure B;

**$R_f$**= 0.4 (hexane/ethyl acetate = 4/1);

$[\alpha]_D^{27}$ = -67.5 (*c* = 0.2 in MeOH);

**IR (film)** $\lambda_{max}$ 3279, 2978, 2928, 2851, 1721, 1643, 1585, 1504, 1458, 1393, 1234, 1153, 1096, 964, 841;

**$^1$H NMR** (500 MHz, $CDCl_3$) δ 13.48 (s, 1H), 7.93 (s, 1H), 6.36 (s, 1H), 4.39 (d, *J* = 2.0 Hz, 1H), 3.92 (m, 1H), 1.66 (m, 4H), 1.65 (s, 3H), 1.55 (m, 2H), 1.49 (s, 3H), 1.44 (s, 3H), 1.42 (s, 3H), 1.28 (d, *J* = 0.8 Hz, 3H), 1.27 (d, *J* = 1.8 Hz, 3H), 1.11 (m, 2H), 0.98 (m, 2H), 0.81 (m, 1H);

**$^{13}$C NMR** (125 MHz, $CDCl_3$) δ 211.9, 208.9, 199.3, 168.5, 164.7, 160.0, 153.4, 112.4, 104.0, 103.6, 100.7, 56.2, 47.4, 45.1, 39.6, 31.2, 29.4, 29.1, 26.6, 26.5, 26.3, 25.2, 25.0, 24.9, 24.3, 20.9, 17.8;

**HRMS** (ESI) calcd for $C_{27}H_{35}O_6$ [(M+H)$^+$] Exact Mass: 455.2428; found: 455.2418;

**HPLC condition**: ChiralCel IE-3 column; *n*-hexane/*i*-propanol = 95:5, 1 mL/min, λ = 280 nm; major enantiomer: $t_R$ = 8.1 min, minor enantiomer: $t_R$ = 6.7 min. 93:7 *er*.

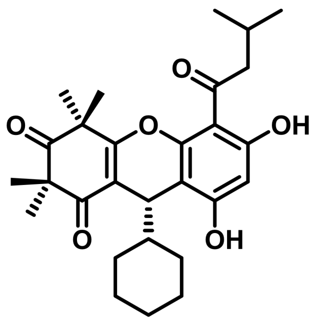

**13l**

Compound **13l**: 33.7 mg, 72% yield, 5 d, white crystals, mp = 198-200 °C, after recrystallization: 11.8 mg; according to procedure B;

**R$_f$**= 0.45 (hexane/ethyl acetate = 4/1);

$[\alpha]_D^{27}$ = -72.3 (*c* = 0.2 in MeOH);

**IR (film)** $\lambda_{max}$ 3348, 2928, 2855, 1717, 1636, 1585, 1470, 1373, 1207, 1157, 1010, 845;

**$^1$H NMR** (500 MHz, $CDCl_3$) δ 13.48 (s, 1H), 7.24 (d, *J* = 9.8 Hz, 1H), 6.31 (s, 1H), 4.32 (d, *J* = 3.8 Hz, 1H), 3.20 (dd, *J* = 17.0, 7.4 Hz, 1H), 2.95 (dd, *J* = 17.0, 6.2 Hz, 1H), 2.38 (m, 1H), 1.68 (s, 3H), 1.64 (m, 6H), 1.48 (s, 3H), 1.47 (s, 3H), 1.41 (s, 3H), 1.13 (m, 2H), 1.05 (d, *J* = 6.7 Hz, 3H), 1.03 (d, *J* = 6.6 Hz, 3H), 0.94 (m, 2H), 0.81 (m, 1H);

**$^{13}$C NMR** (125 MHz, $CDCl_3$) δ 211.8, 204.0, 199.2, 168.4, 164.2, 160.0, 153.8, 112.4, 105.2, 104.1, 100.4, 56.2, 53.3, 47.4, 45.1, 31.2, 29.4, 29.1, 26.6, 26.5, 26.3, 25.4, 25.1, 25.0, 24.6, 24.2, 22.9, 22.7;

**HRMS** (ESI) calcd for $C_{28}H_{37}O_6$ [(M+H)$^+$] Exact Mass: 469.2585; found: 469.2577;

**HPLC condition**: ChiralCel IE-3 column; *n*-hexane/*i*-propanol = 95:5, 1 mL/min, λ = 280 nm; major enantiomer: $t_R$ = 8.6 min, minor enantiomer: $t_R$ = 6.2 min. 96.5:3.5 *er*, re-crystallised: 99.5:0.5 *er*.

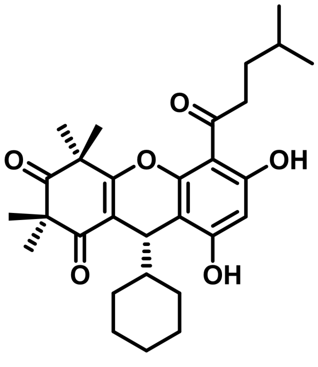

**13m**

Compound **13m**: 34.7 mg, 72% yield, 6 d, white crystals, mp = 205-207 °C, after recrystallization: 19.1 mg; according to procedure B;

**R$_f$**= 0.45 (hexane/ethyl acetate = 4/1);

$[\alpha]_D^{27}$ = -110.9 (*c* = 0.2 in MeOH);

**IR (film)** $\lambda_{max}$ 3345, 2986, 2931, 2855, 1717, 1643, 1597, 1466, 1389, 1204, 1153, 1041, 999, 833;

**$^1$H NMR** (500 MHz, $CDCl_3$) δ 13.52 (s, 1H), 8.37 (s, 1H), 6.37 (s, 1H), 4.40 (d, *J* = 3.8 Hz, 1H), 3.31 (ddd, *J* = 16.9, 9.5, 5.1 Hz, 1H), 3.05 (ddd, *J* = 17.0, 9.3, 5.5 Hz, 1H), 1.69 (s, 3H), 1.67 (m, 7H), 1.54 (m, 2H), 1.49 (s, 3H), 1.48 (s, 3H), 1.41 (s, 3H), 1.11 (m, 2H), 0.98 (m, 2H), 0.97 (d, *J* = 1.6 Hz, 3H), 0.96 (d, *J* = 1.7 Hz, 3H), 0.82 (dd, *J* = 12.3, 2.4 Hz, 1H);

**$^{13}$C NMR** (125 MHz, CDCl3) δ 211.8, 204.7, 199.2, 168.4, 164.2, 159.9, 153.8, 112.4, 105.0, 104.0, 100.4, 56.2, 47.4, 45.1, 42.6, 33.3, 31.2, 29.4, 29.2, 27.7, 26.6, 26.5, 26.3, 25.3, 25.2, 25.0, 24.2, 22.7, 22.6;

**HRMS** (ESI) calcd for $C_{29}H_{39}O_6$ $[(M+H)^+]$ Exact Mass: 483.2741; found: 483.2729;

**HPLC condition**: ChiralCel IE-3 column; *n*-hexane/*i*-propanol = 95:5, 1 mL/min, λ = 280 nm; major enantiomer: $t_R$ = 8.6 min, minor enantiomer: $t_R$ = 6.3 min. 97:3 *er*, re-crystallised: 99:1 *er*.

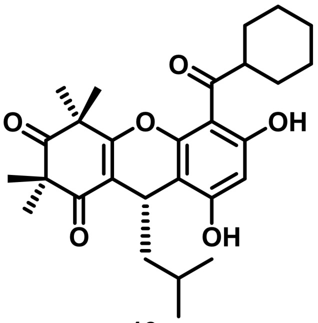

**13n**

Compound **13n**: 25.7 mg, 55% yield, 3 d, white crystals, mp = 232-233 °C; according to procedure B;

**$R_f$**= 0.44 (hexane/ethyl acetate = 4/1);

$[\alpha]_D^{27}$ = -82.5 (*c* = 0.2 in MeOH);

**IR (film)** $\lambda_{max}$ 3233, 2982, 2936, 2855, 1717, 1636, 1504, 1427, 1392, 1258, 1157, 1038, 991, 845;

**$^1$H NMR** (500 MHz, $CDCl_3$) δ 13.19 (s, 1H), 7.22 (s, 1H), 6.30 (s, 1H), 4.33 (t, *J* = 6.1 Hz, 1H), 3.77 (m, 1H), 1.93 (m, 3H), 1.76 (m, 2H), 1.69 (s, 3H), 1.52 (m, 1H), 1.46 (s, 6H), 1.41 (s, 3H), 1.35 (m, 7H), 0.91 (d, *J* = 4.3 Hz, 3H), 0.90 (d, *J* = 4.2 Hz, 3H);

**$^{13}$C NMR** (125 MHz, $CDCl_3$) δ 211.8, 208.6, 198.4, 167.6, 164.3, 159.4, 152.9, 114.8, 106.2, 104.5, 100.3, 56.2, 49.2, 47.3, 46.7, 31.6, 27.6, 26.3, 25.8, 25.3, 25.1, 25.1, 24.9, 24.8, 24.7, 24.1, 23.5, 23.1;

**HRMS** (ESI) calcd for $C_{28}H_{37}O_6$ [$(M+H)^+$] Exact Mass: 469.2585; found: 469.2580;

**HPLC condition**: ChiralCel IE-3 column; *n*-hexane/*i*-propanol = 95:5, 1 mL/min, λ = 280 nm; major enantiomer: $t_R$ = 7.2 min, minor enantiomer: $t_R$ = 5.9 min. 90.5:9.5 *er*.

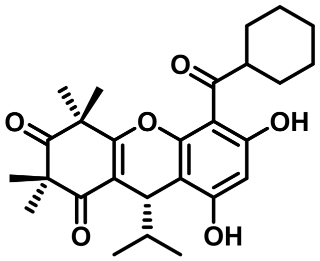

**13o**

Compound **13o**: 33.1 mg, 73% yield, white crystals, mp = 188-190 °C, after recrystallization: 19.8 mg; according to procedure B;

**$R_f$**= 0.42 (hexane/ethyl acetate = 4/1);

$[\alpha]_D^{27}$ = -71.3 (*c* = 0.2 in MeOH);

**IR (film)** $\lambda_{max}$ 3271, 2936, 2855, 1717, 1635, 1575, 1504, 1427, 1393, 1254, 1161, 1038, 995, 840;

**$^1$H NMR** (500 MHz, $CDCl_3$) δ 13.15 (s, 1H), 7.26 (s, 1H), 6.33 (s, 1H), 4.37 (d, *J* = 3.8 Hz, 1H), 3.76 (dd, *J* = 10.8, 2.9 Hz, 1H), 1.94 (m, 4H), 1.82 (m, 2H), 1.72 (s, 3H), 1.49 (s, 3H), 1.45 (s, 3H), 1.41 (s, 3H), 1.37 (m, 5H), 0.86 (d, *J* = 6.9 Hz, 3H), 0.82 (d, *J* = 6.9 Hz, 3H);

**$^{13}$C NMR** (125 MHz, $CDCl_3$) δ 211.8, 208.4, 198.7, 168.5, 164.3, 159.5, 153.6, 112.4, 104.2, 103.9, 100.5, 56.3, 49.3, 47.4, 34.8, 31.8, 31.5, 27.4, 26.4, 25.9, 25.6, 25.3, 25.1, 24.9, 24.0, 18.9, 18.7;

**HRMS** (ESI) calcd for $C_{27}H_{35}O_6$ [$(M+H)^+$] Exact Mass: 455.2428; found: 455.2425;

**HPLC condition**: ChiralCel OD-H column; *i*-PrOH/*n*-hexane = 10:90, 1 mL/min, λ = 280 nm; major enantiomer: $t_R$ = 4.8 min, minor enantiomer: $t_R$ = 7.0 min. 95:5 *er*, re-crystallised: 99.5:0.5 *er*.

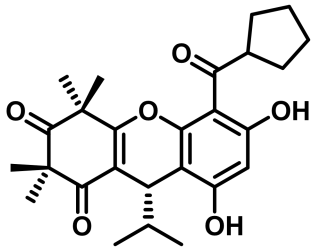

**13p**

Compound **13p**: 32.6 mg, 74% yield, white crystals, mp = 199-200 °C, after recrystallization: 17.3 mg; according to procedure B;

**R$_f$**= 0.42 (hexane/ethyl acetate = 4/1);

$[\alpha]_D^{27}$ = -49.7 (*c* = 0.2 in MeOH);

**IR (film)** $\lambda_{max}$ 3290, 2951, 2866, 1721, 1636, 1589, 1508, 1462, 1393, 1242, 1142, 1034, 999, 829;

**$^1$H NMR** (500 MHz, $CDCl_3$) δ 13.41 (s, 1H), 7.86 (s, 1H), 6.34 (s, 1H), 4.41 (d, *J* = 3.4 Hz, 1H), 4.07 (m, 1H), 2.24 (m, 1H), 2.09 (m, 2H), 1.92 (m, 1H), 1.79 (m, 2H), 1.69 (m, 3H), 1.63 (s, 3H), 1.48 (d, *J* = 10.8 Hz, 3H), 1.43 (s, 3H), 1.40 (s, 3H), 0.85 (d, *J* = 6.9 Hz, 3H), 0.80 (d, *J* = 6.9 Hz, 3H);

**$^{13}$C NMR** (125 MHz, $CDCl_3$) δ 212.0, 207.4, 199.5, 168.7, 164.4, 160.23, 153.5, 112.2, 104.3, 104.1, 100.5, 56.2, 50.9, 47.4, 34.8, 32.4, 31.4, 27.7, 26.0, 25.9, 25.2, 25.0, 24.1, 18.9, 18.6;

**HRMS** (ESI) calcd for $C_{26}H_{33}O_6$ [$(M+H)^+$] Exact Mass: 441.2272; found: 441.2266;

**HPLC condition**: ChiralCel OD-H column; *n*-hexane/*i*-propanol = 90:10, 1 mL/min, λ = 280 nm; major enantiomer: $t_R$ = 4.8 min, minor enantiomer: $t_R$ = 7.0 min. 94.5:5.5 *er*, re-crystallised: 99:1 *er*.

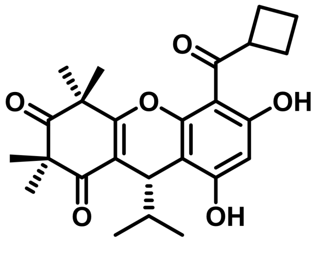

**13q**

Compound **13q**: 30.2 mg, 71% yield, white crystals, mp = 185-187 °C; according to procedure B;

**R$_f$**= 0.42 (hexane/ethyl acetate = 4/1);

$[\alpha]_D^{27}$ = -177.5 (*c* = 0.2 in MeOH);

**IR (film)** $\lambda_{max}$ 3271, 2962, 2934, 2870, 1721, 1643, 1593, 1501, 1462, 1393, 1261, 1110, 1034, 960, 844;

**$^1$H NMR** (500 MHz, $CDCl_3$) δ 13.33 (s, 1H), 8.09 (s, 1H), 6.37 (s, 1H), 4.42 (d, *J* = 3.3 Hz, 1H), 4.19 (m, 1H), 2.66 (m, 1H), 2.44 (m, 1H), 2.17 (m, 2H), 2.02 (m, 1H), 1.90 (m, 2H), 1.74 (s, 3H), 1.49 (s, 3H), 1.46 (s, 3H), 1.41 (s, 3H), 0.83 (d, *J* = 6.8 Hz, 3H), 0.78 (d, *J* = 6.8 Hz, 3H);

**$^{13}$C NMR** (125 MHz, $CDCl_3$) δ 211.9, 205.7, 199.4, 168.7, 164.3, 160.1, 153.6, 112.3, 103.8, 103.1, 100.5, 56.2, 47.4, 46.4, 34.7, 31.4, 28.4, 25.4, 25.2, 25.0, 23.8, 22.9, 18.8, 18.6, 17.9;

**HRMS** (ESI) calcd for $C_{25}H_{31}O_6$ [$(M+H)^+$] Exact Mass: 427.2115; found: 427.2106;

**HPLC condition**: ChiralCel OD-H column; *n*-hexane/*i*-propanol = 90:10, 1 mL/min, λ = 280 nm; major enantiomer: $t_R$ = 5.1 min, minor enantiomer: $t_R$ = 7.0 min. 93:7 *er*, re-crystallised: 99.5:0.5 *er*.

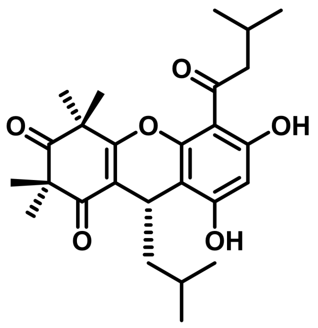


**13r**

Compound **13r**: 22.5 mg, 51% yield, 3 d, white crystals, mp = 137-139 °C; according to procedure B;

**R$_f$**= 0.42 (hexane/ethyl acetate = 4/1);

$[\alpha]_D^{27}$ = -125.6 (*c* = 0.2 in MeOH);

**IR (film)** $\lambda_{max}$ 3356, 2978, 2855, 1717, 1628, 1593, 1427, 1312, 1207, 1153, 1072, 964, 833;

**$^1$H NMR** (500 MHz, $CDCl_3$) δ 13.53 (s, 1H), 7.44 (s, 1H), 6.31 (s, 1H), 4.35 (t, *J* = 6.1 Hz, 1H), 3.21 (dd, *J* = 17.2, 7.5 Hz, 1H), 2.98 (dd, *J* = 17.2, 6.1 Hz, 1H), 2.38 (m, 1H), 1.66 (s, 3H), 1.50 (m, 1H), 1.49 (s, 3H), 1.46 (s, 3H), 1.42 (s, 3H), 1.39 (m, 2H), 1.05 (d, *J* = 6.7 Hz, 3H), 1.03 (d, *J*

= 6.6 Hz, 3H), 0.89 (dd, $J$ = 11.2, 5.1 Hz, 6H);

**$^{13}$C NMR** (125 MHz, $CDCl_3$) δ 211.8, 204.0, 198.8, 167.5, 164.3, 159.7, 153.1, 114.6, 106.0, 105.5, 100.2, 56.2, 53.4, 47.3, 46.9, 25.4, 25.0, 24.7, 24.6, 24.5, 24.2, 23.4, 23.1, 22.9, 22.7;

**HRMS** (ESI) calcd for $C_{26}H_{35}O_6$ [(M+H)$^+$] Exact Mass: 443.2428; found: 443.2428;

**HPLC condition**: ChiralCel AD-H column; *n*-hexane/*i*-propanol = 95:5, 1 mL/min, λ = 280 nm; major enantiomer: $t_R$ = 6.3 min, minor enantiomer: $t_R$ = 4.7 min. 92:8 *er*.

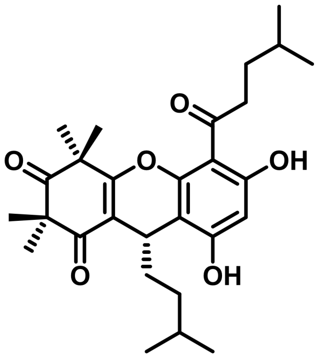

**13s**

Compound **13s**: 32.9 mg, 70% yield, 3 d, white crystals, mp = 138-140 °C, after recrystallization: 13.5 mg; according to procedure B;

**$R_f$**= 0.44 (hexane/ethyl acetate = 4/1);

$[\alpha]_D^{27}$ = -94.0 ($c$ = 0.2 in MeOH);

**IR (film)** $\lambda_{max}$ 3402, 2958, 2904, 2870, 1705, 1654, 1628, 1593, 1504, 1462, 1389, 1250, 1119, 1003, 841;

**$^1$H NMR** (500 MHz, $CDCl_3$) δ 13.50 (s, 1H), 7.21 (s, 1H), 4.35 (t, $J$ = 4.6 Hz, 1H), 3.30 (ddd, $J$ = 17.3, 9.2, 5.4 Hz, 1H), 3.05 (ddd, $J$ = 17.3, 9.0, 5.5 Hz, 1H), 1.64 (m, 6H), 1.66 (s, 3H), 1.51 (s, 3H), 1.44 (s, 3H), 1.42 (s, 3H), 0.98 (d, $J$ = 2.2 Hz, 3H), 0.97 (d, $J$ = 2.2 Hz, 3H), 0.89 (m, 3H), 0.78 (s, 3H), 0.77 (s, 3H);

**$^{13}$C NMR** (125 MHz, $CDCl_3$) δ 211.8, 204.8, 198.8, 167.7, 164.3, 160.1, 152.8, 112.6, 104.7, 104.4, 100.4, 56.2, 47.2, 42.7, 33.7, 33.2, 32.4, 28.0, 27.7, 26.4, 25.2, 24.6, 24.1, 22.7, 22.6, 22.6;

**HRMS** (ESI) calcd for $C_{28}H_{39}O_6$ [(M+H)$^+$] Exact Mass: 471.2741; found: 471.2731;

**HPLC condition**: ChiralCel IE-3 column; *n*-hexane/*i*-propanol = 95:5, 1 mL/min, λ = 280 nm; major enantiomer: $t_R$ = 7.0 min, minor enantiomer: $t_R$ = 5.7 min. 93.5:6.5 *er*, re-crystallised: 99.8:0.2 *er*.

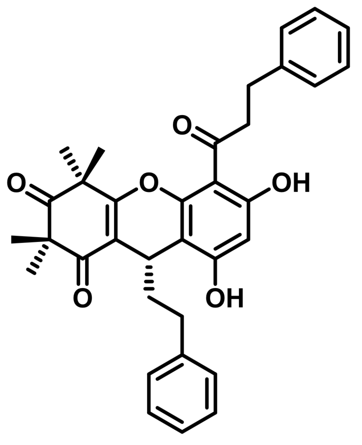

**13t**

Compound **13t**: 39.8 mg, 74% yield, 5 d, white crystals, mp = 126-127 °C; according to procedure B;

**R$_f$**= 0.43 (hexane/ethyl acetate = 4/1);

$[\alpha]_D^{27}$ = -41.7 ($c$ = 0.2 in MeOH);

**IR (film)** $\lambda_{max}$ 3283, 3024, 2982, 2935, 2855, 1717, 1655, 1616, 1593, 1454, 1385, 1180, 1037, 841, 744;

**$^1$H NMR** (500 MHz, $CDCl_3$) δ 13.66 (s, 1H), 8.14 (s, 1H), 7.33 (t, $J$ = 7.4 Hz, 2H), 7.25 (dd, $J$ = 18.0, 7.3 Hz, 3H), 7.18 (t, $J$ = 7.3 Hz, 2H), 7.13 (d, $J$ = 7.1 Hz, 1H), 7.01 (d, $J$ = 7.3 Hz, 2H), 6.35 (s, 1H), 4.48 (t, $J$ = 4.8 Hz, 1H), 3.50 (m, 2H), 3.11 (m, 2H), 2.45 (m, 2H), 2.07 (m, 1H), 1.89 (m, 1H), 1.44 (s, 3H), 1.42 (s, 3H), 1.38 (s, 3H), 1.37 (s, 3H);

**$^{13}$C NMR** (125 MHz, $CDCl_3$) δ 211.5, 202.9, 198.4, 167.6, 164.8, 159.9, 152.9, 141.6, 141.0, 128.6, 128.4, 128.3, 128.2, 126.3, 125.9, 112.4, 104.8, 104.0, 100.4, 56.2, 47.2, 47.2, 36.4, 31.43, 30.0, 26.6, 25.3, 24.8, 24.5, 24.2;

**HRMS** (ESI) calcd for $C_{34}H_{35}O_6$ [$(M+H)^+$] Exact Mass: 539.2428; found: 539.2416;

**HPLC condition**: ChiralCel IE-3 column; $n$-hexane/$i$-propanol = 95:5, 1 mL/min, λ = 280 nm; major enantiomer: $t_R$ = 11.7 min, minor enantiomer: $t_R$ = 9.4 min. 95:5 *er*.

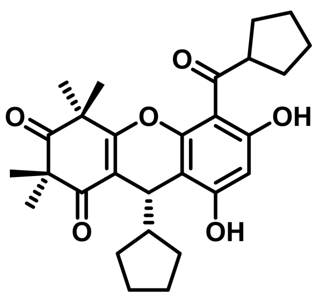

**13u**

Compound **13u**: 34.0 mg, 73% yield, 4 d, white crystals, mp = 220-221 °C, after recrystallization: 13.9 mg; according to procedure B;

**R$_f$**= 0.45 (hexane/ethyl acetate = 4/1);

$[\alpha]_D^{27}$ = -161.2 ($c$ = 0.2 in MeOH);

**IR (film)** $\lambda_{max}$ 3283, 2955, 2870, 1717, 1651, 1628, 1508, 1447, 1393, 1254, 1157, 1034, 837;

**$^1$H NMR** (500 MHz, $CDCl_3$) δ 13.48 (s, 1H), 8.35 (s, 1H), 6.37 (s, 1H), 4.53 (d, $J$ = 5.2 Hz, 1H), 4.09 (m, 1H), 2.24 (m, 1H), 2.13 (m, 1H), 2.00 (m, 1H), 1.83 (m, 2H), 1.70 (m, 4H), 1.63 (s, 3H), 1.54 (m, 4H), 1.47 (s, 3H), 1.45 (s, 3H), 1.41 (m, 2H), 1.41 (s, 3H), 1.16 (m, 2H);

**$^{13}$C NMR** (125 MHz, $CDCl_3$) δ 212.0, 207.5, 199.4, 168.7, 164.4, 160.1, 153.5, 113.5, 104.7, 104.5, 100.5, 56.2, 50.9, 47.7, 47.4, 32.4, 28.7, 28.3, 27.8, 26.0, 26.0, 25.2, 24.9, 24.7, 24.5, 24.29, 24.1;

**HRMS** (ESI) calcd for $C_{28}H_{35}O_6$ $[(M+H)^+]$ Exact Mass: 467.2428; found: 467.2425;

**HPLC condition**: ChiralCel IE-3 column; $n$-hexane/$i$-propanol = 95:5, 1 mL/min, λ = 280 nm; major enantiomer: $t_R$ = 9.5 min, minor enantiomer: $t_R$ = 7.3 min. 92.5:7.5 *er*, re-crystallised: 99.8:0.2 *er*.

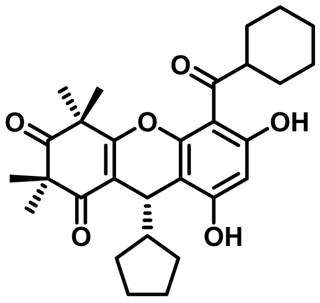

**13v**

Compound **13v**: 36.4 mg, 76% yield, 4 d, white crystals, mp = 204-205 °C, after recrystallization: 17.1 mg; according to procedure B;

**R$_f$**= 0.54 (hexane/ethyl acetate = 4/1);

$[\alpha]_D^{27}$ = -75.2 ($c$ = 0.2 in MeOH);

**IR (film)** $\lambda_{max}$ 3341, 2978, 2932, 2855, 1717, 1647, 1628, 1593, 1454, 1389, 1250, 1153, 1038, 964, 833;

**$^1$H NMR** (500 MHz, MeOD) δ 6.24 (s, 1H), 4.36 (d, $J$ = 5.2 Hz, 1H), 3.63 (m, 1H), 2.04 (m, 1H), 1.82 (m, 6H), 1.65 (s, 3H), 1.53 (m, 4H), 1.47 (m, 2H), 1.44 (s, 3H), 1.40 (s, 3H), 1.40 (m, 2H), 1.35 (s, 3H), 1.29 (m, 4H);

**$^{13}$C NMR** (125 MHz, MeOD) δ 211.8, 207.8, 198.1, 168.4, 161.5, 160.0, 152.5, 113.1, 104.8, 104.5, 98.9, 55.7, 49.7, 47.5, 47.2, 30.7, 28.4, 28.3, 28.1, 27.5, 25.9, 25.7, 25.2, 24.6, 24.0, 23.9, 23.9, 23.8, 23.3;

**HRMS** (ESI) calcd for $C_{29}H_{37}O_6$ [$(M+H)^+$] Exact Mass: 481.2585; found: 481.2573;

**HPLC condition**: ChiralCel IE-3 column; *n*-hexane/*i*-propanol = 95:5, 1 mL/min, λ = 280 nm; major enantiomer: $t_R$ = 9.7 min, minor enantiomer: $t_R$ = 7.3 min. 94:6 *er*, re-crystallised: 99:1 *er*.

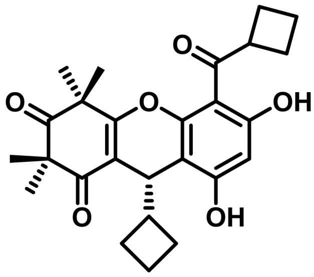

**13w**

Compound **13w**: 21.9 mg, 50% yield, 18 h, white crystals, mp = 198-200 °C; according to procedure B;

**R$_f$**= 0.42 (hexane/ethyl acetate = 4/1);

$[\alpha]_D^{27}$ = -109.0 ($c$ = 0.2 in MeOH);

**IR (film)** $\lambda_{max}$ 3356, 2978, 2932, 2855, 1717, 1659, 1632, 1593, 1454, 1366, 1250, 1153, 1038, 999, 833;

**$^1$H NMR** (500 MHz, $CDCl_3$) δ 13.34 (s, 1H), 7.95 (s, 1H), 6.38 (s, 1H), 4.35 (d, $J$ = 5.5 Hz, 1H), 4.20 (m, 1H), 2.64 (m, 2H), 2.46 (m, 1H), 2.20 (m, 3H), 2.00 (m, 1H), 1.91 (m, 1H), 1.71 (s, 3H), 1.64 (m, 5H), 1.48 (s, 6H), 1.41 (s, 3H);

**$^{13}$C NMR** (125 MHz, $CDCl_3$) δ 211.8, 205.7, 199.1, 168.3, 164.3, 160.2, 153.1, 112.0, 103.4, 103.2, 100.4, 56.2, 47.3, 46.4, 41.5, 29.1, 28.3, 25.2, 25.1, 24.9, 24.8, 24.3, 24.2, 23.0, 17.9, 17.8;

**HRMS** (ESI) calcd for $C_{26}H_{31}O_6$ [(M+H)$^+$] Exact Mass: 439.2115; found: 439.2105;

**HPLC condition**: ChiralCel AD-H column; *n*-hexane/*i*-propanol = 95:5, 1 mL/min, λ = 280 nm; major enantiomer: $t_R$ = 7.8 min, minor enantiomer: $t_R$ = 5.7 min. 89:11 *er*.

**2.8 Synthesis of 9**

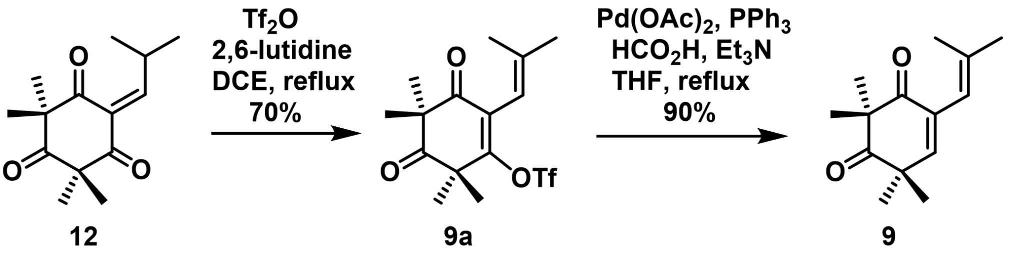


To a solution of compound **12** (5.9 g, 25 mmol) in 1,2-dichloroethane (DCE; 80 mL) under argon was added 2,6-lutidine (4.4 mL, 37.5 mmol, 1.5 equiv.), and the resulting mixture was warmed to reflux. Then, trifluoromethanesulfonic anhydride ($Tf_2O$; 6.3 mL, 37.5mmol, 1.5 equiv.) was added. After stirred for 1.5 h, the mixture was cooled down to 26 $^oC$ and quenched with saturated aqueous sodium bicarbonate (50 mL). The mixture was extracted with dichloromethane (2×50 mL). The combined organic layers were washed with saturated aqueous sodium chloride (2×80 mL), dried over $Na_2SO_4$, filtered and concentrated in vacuo. The crude residue was purified by silica gel column chromatography (0.5%-1.5% hexanes/ethyl acetate) to afford the compound **9a** (6.45 g, 70%) as yellow oil.

Palladium acetate ($Pd(OAc)_2$; 47.5 wt%, 141.5 mg, 0.3 mmol, 2 mol%) and triphenylphosphine ($PPh_3$; 157.4 mg, 0.6 mmol, 4 mol%) under argon were added to a solution of compound **9a** (5.52 g, 15 mmol) in tetrahydrofuran (THF; 60 mL), and the resulting solution was stirred for 10 min at 26 $^oC$. A solution of triethylamine (4.2 mL, 30 mmol, 2 equiv.) and formic acid (1.2 mL, 31.5 mmol, 2.1 equiv.) in THF (63 mL) were then added dropwise via cannula over 5 min to the reaction mixture. The resulting mixture was refluxed overnight and the mixture was cooled down to 26 $^oC$ and poured into saturated aqueous sodium chloride solution (80 mL). The mixture was extracted with ethyl acetate (2×80 mL) and the combined organic layers were dried over $Na_2SO_4$, filtered and concentrated in vacuo. The crude residue was purified by silica gel column chromatography (1% hexanes/ethyl acetate) to afford the title compound **9** (2.97 g, 90%) as yellowish crystals.

Compound **9a**:

**R$_f$**= 0.6 (hexane/ethyl acetate = 12/1);

**IR (film)** $\lambda_{max}$ 2986, 2944, 2877, 1730, 1690, 1633, 1470, 1449, 1420, 1176, 1136, 1020, 936, 837;

**$^1$H NMR** (500 MHz, $CDCl_3$) $\delta$ 5.62 (s, 1H), 1.86 (s, 3H), 1.55 (s, 3H), 1.46 (s, 6H), 1.39 (s, 6H);

**$^{13}$C NMR** (125 MHz, $CDCl_3$) $\delta$ 208.8, 197.7, 162.3, 144.4, 127.1, 122.2, 119.7, 117.1, 114.6, 113.6, 57.4, 49.0, 25.6, 24.6, 23.5, 20.0;

**HRMS** (ESI) calcd for $C_{15}H_{20}F_3O_5S$ [$(M+H)^+$] Exact Mass: 369.0978; found: 369.0971.

Compound **9**: mp = 33-34 $^o$C;

**R$_f$**= 0.5 (hexane/ethyl acetate = 12/1);

**IR (film)** $\lambda_{max}$ 2976, 2936, 2870, 1719, 1676, 1638, 1468, 1379, 1360, 1302, 1250, 1042, 854;

**$^1$H NMR** (400 MHz, $CDCl_3$) $\delta$ 6.50 (s, 1H), 5.93 (s, 1H), 1.87 (s, 3H), 1.75 (s, 3H), 1.35 (s, 6H), 1.34 (s, 6H);

**$^{13}$C NMR** (100 MHz, $CDCl_3$) $\delta$ 213.4, 201.1, 149.3, 138.5, 132.5, 118.9, 57.5, 45.1, 27.5, 26.6, 23.7, 19.5;

**HRMS** (ESI) calcd for $C_{14}H_{21}O_2$ [$(M+H)^+$] Exact Mass: 221.1536; found: 221.1533.

**2.9 Synthesis of 2a, 3a**

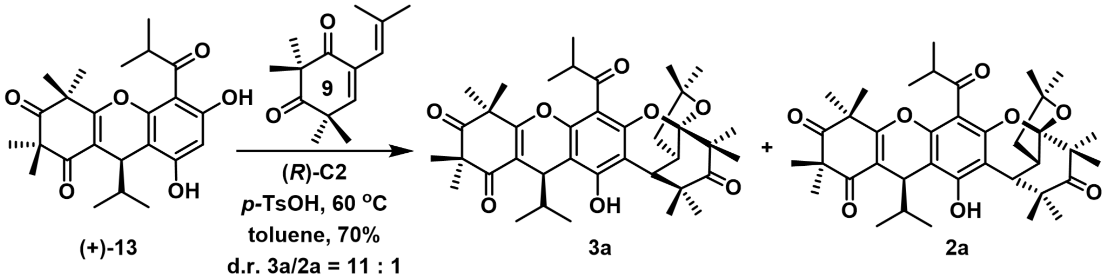


To a solution of compound (+)-**13** (2.0 g, 4.8 mmol), compound **9** (1.6 g, 7.2 mmol, 1.5 equiv.) and (*R*)-1,1'-Binaphthyl-2,2'-diyl hydrogenphosphate [(*R*)-**C2**; 1.67 g, 4.8 mmol, 1 equiv.] in toluene (48 mL) under argon were added *p*-toluenesulfonic acid (*p*-TsOH; 1.4 g, 7.2 mmol, 1.5 equiv.), and the resulting mixture was warmed to 60 °C and stirred for 24 h. The mixture was cooled down to 26 $^o$C and quenched with saturated aqueous sodium bicarbonate (30 mL). The mixture was extracted with dichloromethane (3×30 mL) and the combined organic layers were dried over $Na_2SO_4$, filtered and concentrated in vacuo. The crude residue was purified by silica gel column chromatography (2.5%-10% hexanes/ethyl acetate) to afford the mixture of title

compound **2a** with compound **3a** (2.13 g, 70%) and (*R*)-**C2** (1.55 g). The mixture of compound **2a** with **3a** (2.13 g) was re-crystallised from methanol containing a few drops of $CH_2Cl_2$ to provide the compound **2a** (177.3 mg, 5.8 %) as white crystals and compound **3a** (1.95 g, 64.2%) as white crystals.

Compound **2a**: mp = 244-246 °C;

**R$_f$**= 0.3 (hexane/ethyl acetate = 4/1);

$[\alpha]_D^{25}$ = +84.9 (*c* = 0.1 in $CHCl_3$);

**IR (film)** $\lambda_{max}$ 3410, 2968, 2930, 2874, 1703, 1658, 1603, 1139, 1188, 1172, 1096, 959;

**$^1$H NMR** (600 MHz, $CDCl_3$) $\delta$ 7.00 (s, 1H), 4.50 (d, *J* = 3.2 Hz, 1H), 3.43 – 3.39 (m, 1H), 3.36 (d, *J* = 3.1 Hz, 1H), 3.24 – 3.19 (m, 1H), 1.93 (dd, *J* = 12.2, 7.4 Hz, 1H), 1.89 – 1.83 (m, 1H), 1.51 (s, 3H), 1.49 – 1.47 (m, 1H), 1.44 (s, 3H), 1.42 (s, 3H), 1.40 (s, 3H), 1.40 (s, 3H), 1.34(s, 6H), 1.26 (s, 3H), 1.23 (s, 3H), 1.15 (d, *J* = 7.0 Hz, 3H), 1.12 (d, *J* = 6.9 Hz, 3H), 0.97 (s, 3H), 0.78 (d, *J* = 6.8 Hz, 3H), 0.72 (d, *J* = 6.9 Hz, 3H);

**$^{13}$C NMR** (150 MHz, $CDCl_3$) $\delta$ 217.2, 212.0, 205.9, 199.2, 170.1, 152.9, 149.3, 148.2, 112.2, 110.6, 110.1, 106.4, 102.8, 84.6, 55.7, 53.9, 50.1, 47.7, 41.5, 39.1, 38.6, 35.2, 35.2, 31.9, 30.0, 29.2, 28.7, 25.1, 25.0, 24.8, 24.6, 24.2, 24.0, 19.1, 18.9, 18.8, 18.3, 18.0;

**HRMS** (ESI) calcd for $C_{38}H_{51}O_8$ [$(M+H)^+$] Exact Mass: 635.3578; found: 635.3579.

Compound **3a**: mp = 236-237 °C;

**R$_f$**= 0.3 (hexane/ethyl acetate = 4/1);

$[\alpha]_D^{25}$ = -43.2 (*c* = 0.1 in $CHCl_3$);

**IR (film)** $\lambda_{max}$ 3398, 2973, 2938, 2873, 1714, 1671, 1653, 1588, 1465, 1442, 1385, 1197, 1136, 1069, 961, 863;

**$^1$H NMR** (600 MHz, $CDCl_3$) $\delta$ 7.90 (s, 1H), 4.64 (d, *J* = 3.6 Hz, 1H), 3.44 (d, *J* = 3.1 Hz, 1H), 3.38 – 3.34 (m, 1H), 3.14 – 3.10 (m, 1H), 1.85 – 1.81 (m, 1H), 1.77 (dd, *J* = 12.1, 7.6 Hz, 1H), 1.47 (m, 1H), 1.45 (s, 3H), 1.42 (s, 6H), 1.41 (s, 3H), 1.35 (s, 3H), 1.28 (s, 3H), 1.26 (s, 3H), 1.25 (s, 3H), 1.22 (s, 3H), 1.13 (d, *J* = 6.7 Hz, 3H), 1.10 (d, *J* = 7.1 Hz, 3H), 1.07 (s, 3H), 0.74 (d, *J* = 6.8 Hz, 3H), 0.66 (d, *J* = 6.9 Hz, 3H);

**$^{13}$C NMR** (150 MHz, $CDCl_3$) $\delta$ 217.8, 211.9, 206.6, 199.8, 170.4, 153.1, 149.0, 148.0, 112.1, 110.9, 110.2, 106.3, 103.0, 84.6, 55.6, 53.8, 50.3, 47.7, 41.9, 38.8, 38.5, 35.3, 34.9, 31.7, 29.9, 28.9, 28.6, 25.0, 24.9, 24.7, 24.5, 24.2, 23.9, 19.2, 18.7, 18.5, 17.3;

**HRMS** (ESI) calcd for $C_{38}H_{51}O_8$ $[(M+H)^+]$ Exact Mass: 635.3578; found: 635.3577.

**Synthesis of (+)-myrtucommuacetalone (2)**

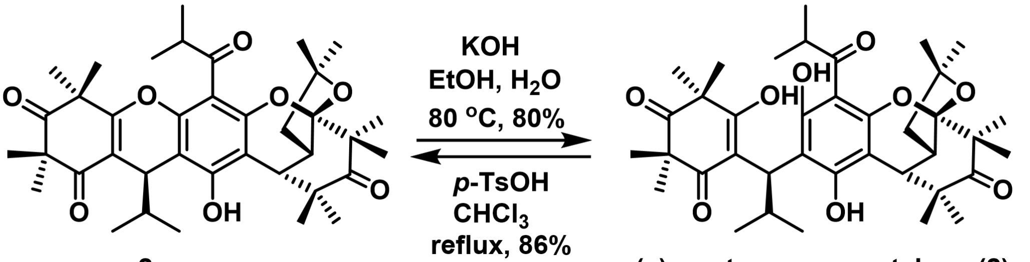


**2a** **(+)-myrtucommuacetalone (2)**

To a solution of compound **2a** (150 mg, 0.24 mmol) in ethyl alcohol-water (EtOH-$H_2O$; 1:1, v/v) (12 mL) was added potassium hydroxide (KOH; 670 mg, 12 mmol, 50 equiv.), and the resulting solution was stirred overnight at 80 °C. The mixture was cooled down to 26 $^oC$ and quenched with 1M HCl (12 mL). The mixture was extracted with ethyl acetate (3×15 mL) and the combined organic layers were dried over $Na_2SO_4$, filtered and concentrated in vacuo. The crude residue was purified by silica gel column chromatography (2.5%-4% hexanes/ethyl acetate) to afford the title compound (+)-myrtucommuacetalone (**2**) (125 mg, 80%) as yellow crystals.

**Note**: 1. Both $^1H$ and $^{13}C$ NMR spectra of the (+)-myrtucommuacetalone (**2**) showed doubled signal patterns, which was probably because of the presence of rotamers or keto-enol tautomers. Further cyclization of compound **2** was carried out to afford **2a** with a single $^1H$ NMR signal pattern, suggesting that compound **2** was indeed to exist as rotamers or keto-enol tautomers.

2. The potassium hydroxide (KOH) concentration would be kept at about 1 mol/L and the reaction temperature would be controlled at 80 °C, at the same time the ratio of ethyl alcohol and water would be about 1:1 and in this condition few of substrate **2a** and product **2** would be decomposed. If the concentration of KOH or temperature is too high, the substrate **2a** and product **2** may be decomposed and the yield will be decreased. If the concentration of KOH or temperature is too low, the reaction would be very slow, even not take place.

To a solution of (+)-myrtucommuacetalone (**2**) (65.2 mg, 0.1 mmol) in chloroform ($CHCl_3$; 3 mL) under argon were added *p*-toluenesulfonic acid (*p*-TsOH; 9.5 mg, 0.05 mmol, 0.5 equiv.), and the resulting mixture was warmed up to reflux for 3 h. The mixture was cooled down to 26 $^oC$ and quenched with saturated aqueous sodium bicarbonate (5 mL). The mixture was extracted with dichloromethane (3×5 mL). The combined organic layers were dried over $Na_2SO_4$, filtered and

concentrated in vacuo. The crude residue was purified by silica gel column chromatography (4% hexanes/ethyl acetate) to afford the mixture of compound **2a** (54.5 mg, 86%) as white crystals.

(+)-myrtucommuacetalone (**2**): mp = 134-136 °C;

**$R_f$**= 0.35 (hexane/ethyl acetate = 8/1);

$[\alpha]_D^{25}$ = +24.0 ($c$ = 0.1 in $CHCl_3$);

**IR (film)** $\lambda_{max}$ 3151, 2978, 2940, 2873, 1719, 1705, 1613, 1565, 1472, 1383, 1132, 1092, 851;

**$^1$H NMR** (500 MHz, $CDCl_3$) $\delta$ 16.74 (s, 1H), 11.72 (s, 1H), 10.60 (s, 1H), 4.13 – 4.07 (m, 1H), 3.72 (d, $J$ = 10.9 Hz, 1H), 3.43 (d, $J$ = 3.2 Hz, 1H), 3.41 – 3.36 (m, 1H), 2.96 – 2.89 (m, 1H), 1.85 (d, $J$ = 12.2 Hz, 7.4 Hz, 1H), 1.49 (s, 3H), 1.46 – 1.44 (m, 1H), 1.41 (s, 3H), 1.39 (s, 3H), 1.37 (s, 3H), 1.33 (s, 3H), 1.32 (s, 3H), 1.30 (s, 6H), 1.27 (s, 3H), 1.15 (d, 5.1 Hz, 3H), 1.14 (d, 5.2 Hz, 3H), 1.03 (s, 3H), 0.81 (d, $J$ = 6.3 Hz, 3H), 0.67 (d, $J$ = 6.5 Hz, 3H);

**$^{13}$C NMR** (125 MHz, $CDCl_3$) $\delta$ 217.8, 212.5, 211.5, 202.9, 178.3, 162.5, 162.0, 154.3, 114.2, 113.9, 108.3, 103.1, 102.3, 84.9, 55.0, 53.8, 50.0, 48.8, 39.2, 39.2, 37.6, 37.5, 34.9, 30.1, 29.0, 28.6, 26.8, 25.9, 25.7, 25.2, 24.5, 23.7, 23.1, 22.0, 21.6, 20.8, 19.5, 18.9;

**HRMS** (ESI) calcd for $C_{38}H_{53}O_9$ [$(M+H)^+$] Exact Mass: 653.3684; found: 653.3685.

**Table S3. Compared NMR data [$CDCl_3$] between our synthetic (+)-myrtucommuacetalone (2) and the isolated natural product.**

| position | $^1H$ δ ppm (J) isolated (300M) | synthesized (500M) | error (iso. - syn.) | $^{13}C$ δ ppm isolated (125M) | synthesized (125M) | error (iso. - syn.) |
|---|---|---|---|---|---|---|
| 1 | - | - | - | 113.9 | 113.9 | 0 |
| 2 | - | - | - | - | - | - |
| 3 | - | - | - | 162.5 | 162.5 | 0 |
| 4 | - | - | - | 103.1 | 103.1 | 0 |
| 5 | 16.73* (s, 1H) | 16.74* (s, 1H) | -0.01 | 162.0 | 162.0 | 0 |
| 6 | - | - | - | 108.3 | 108.3 | 0 |
| 7 | 10.59* (s, 1H) | 10.60* (s, 1H) | -0.01 | 154.3 | 154.3 | 0 |
| 8 | - | - | - | 102.3 | 102.3 | 0 |
| 9 | 3.43 (m) | 3.43 (d, *J* = 3.2 Hz, 1H) | 0 | 37.5 | 37.5 | 0 |
| 10 | 3.40 (m, 1H) | 3.39 (m, 1H) | 0.01 | 34.9 | 34.9 | 0 |
| 11 | 1.86 (m, 1H) | 1.85 (d, *J* = 12.2, 7.4 Hz, 1H) | 0.01 | 39.2 | 39.2 | 0 |
| 12 | - | - | - | 84.9 | 84.9 | 0 |
| 13 | - | - | - | - | - | - |
| 14 | - | - | - | 53.8 | 53.8 | 0 |
| 15 | - | - | - | 217.8 | 217.8 | 0 |
| 16 | - | - | - | 50.0 | 50.0 | 0 |
| 17 | 3.73 (d, *J* = 10.5 Hz, 1H) | 3.72 (d, *J* = 10.9 Hz, 1H) | 0.01 | 39.2 | 39.2 | 0 |
| 18 | 2.92 (m, 1H) | 2.93 (m, 1H) | -0.01 | 25.9 | 25.9 | 0 |
| 19 | 0.68 (d, *J* = 6.0 Hz, 3H) | 0.67 (d, *J* = 6.5 Hz, 3H) | -0.01 | 21.6 | 21.6 | 0 |
| 20 | 0.80 (d, *J* = 6.0 Hz, 3H) | 0.81 (d, *J* = 6.3 Hz, 3H) | -0.01 | 22.0 | 22.0 | 0 |
| 21 | 1.30 (s, 3H) | 1.30 (s, 3H) | 0 | 30.1 | 30.1 | 0 |
| 22 | 1.31 (s, 3H) | 1.32 (s, 3H) | -0.01 | 28.5 | 28.6 | 0 |
| 23 | 1.30 (s, 3H) | 1.30 (s, 3H) | 0 | 19.5 | 19.5 | 0 |
| 24 | 1.27 (s, 3H) | 1.27 (s, 3H) | 0 | 24.5 | 24.5 | 0 |
| 25 | 1.03 (s, 3H) | 1.03 (s, 3H) | 0 | 23.7 | 23.7 | 0 |
| 26 | 1.39 (s, 3H) | 1.39 (s, 3H) | 0 | 29.0 | 29.0 | 0 |
| 1' | - | - | - | 114.2 | 114.2 | 0 |
| 2' | - | - | - | 202.9 | 202.9 | 0 |
| 3 | - | - | - | 55.0 | 55.0 | 0 |
| 4' | - | - | - | 212.5 | 212.5 | 0 |
| 5' | - | - | - | 48.8 | 48.8 | 0 |
| 6' | 11.72* (s, 1H) | 11.72* (s, 1H) | 0 | 178.3 | 178.3 | 0 |
| 7' | - | - | - | 211.5 | 211.5 | 0 |
| 8' | 4.09 (septet, 1H) | 4.10 (m, 1H) | -0.01 | 37.6 | 37.6 | 0 |
| 9' | 1.13 (d, *J* = 5.0 Hz, 3H) | 1.14 (d, *J* = 5.2 Hz, 3H) | -0.01 | 20.8 | 20.8 | 0 |
| 10' | 1.14 (d, *J* = 6.0 Hz, 3H) | 1.15 (d, *J* = 5.1 Hz, 3H) | -0.01 | 18.9 | 18.9 | 0 |
| 11' | 1.33(s, 3H) | 1.33(s, 3H) | 0 | 23.1 | 23.1 | 0 |
| 12' | 1.41 (s, 3H) | 1.41 (s, 3H) | 0 | 26.8 | 26.8 | 0 |
| 13' | 1.37 (s, 3H) | 1.37 (s, 3H) | 0 | 25.7 | 25.7 | 0 |
| 14' | 1.49 (s, 3H) | 1.49 (s, 3H) | 0 | 25.2 | 25.2 | 0 |

*OH

**Synthesis of (-)-myrtucommuacetalone B (3)**

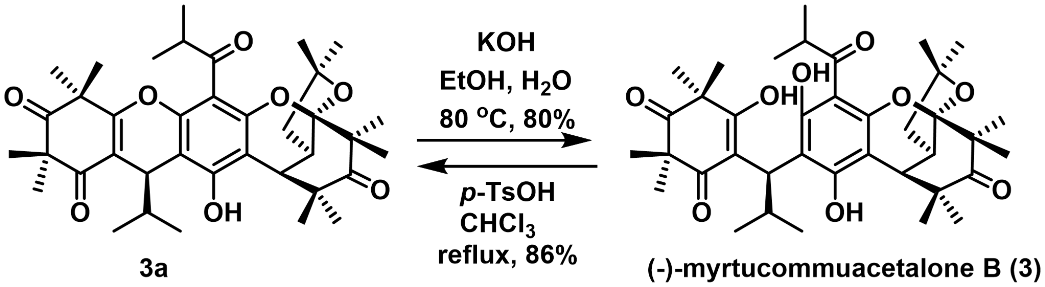


3a

(-)-myrtucommuacetalone B (3)

To a solution of compound **3a** (500 mg, 0.79 mmol) in ethyl alcohol-water (EtOH-$H_2O$; 1:1, v/v) (40 mL) was added potassium hydroxide (KOH; 2.2 g, 39.5 mmol, 50 equiv.), and the resulting solution was stirred overnight at 80 °C. The mixture was cooled down to 26 °C and quenched with 1M HCl (40 mL). The mixture was extracted with ethyl acetate (3×40 mL) and the combined organic layers were dried over $Na_2SO_4$, filtered and concentrated in vacuo. The crude residue was purified by silica gel column chromatography (2.5%-4% hexanes/ethyl acetate) to

afford the title compound (-)-myrtucommuacetalone B (**3**) (412 mg, 80%) as yellow crystals.

**Note**: 1. Both $^{1}H$ and $^{13}C$ NMR spectra of the (-)-myrtucommuacetalone B (**3**) showed doubled signal patterns, which was probably because of the presence of rotamers or keto-enol tautomers. Further cyclization of compound **3** was carried out to afford **3a** with a single $^{1}H$ NMR signal pattern, suggesting that compound **3** was indeed to exist as rotamers or keto-enol tautomers.

2. The potassium hydroxide (KOH) concentration would be kept at about 1 mol/L and the reaction temperature would be controlled at 80 °C, at the same time the ratio of ethyl alcohol and water would be about 1:1 and in this condition few of substrate **3a** product **3** would be decomposed. If the concentration of KOH or temperature is too high, the substrate **3a** and product **3** may be decomposed and the yield will be decreased. If the concentration of KOH or temperature is too low, the reaction would be very slow, even not take place.

To a solution of (-)-myrtucommuacetalone B (**3**) (65.2 mg, 0.1 mmol) in chloroform ($CHCl_3$; 3 mL) under argon were added *p*-toluenesulfonic acid (*p*-TsOH; 9.5 mg, 0.05 mmol, 0.5 equiv.), and the resulting mixture was warmed up and refluxed for 3 h. The mixture was cooled down to 26 °C and quenched with saturated aqueous sodium bicarbonate (5 mL). The mixture was extracted with dichloromethane (3×5 mL). The combined organic layers were dried over $Na_2SO_4$, filtered and concentrated in vacuo. The crude residue was purified by silica gel column chromatography (4% hexanes/ethyl acetate) to afford the compound **3a** (54.5 mg, 86%) as white crystals.

Compound (-)-myrtucommuacetalone B (**3**): mp = 215-217 °C;

$\mathbf{R_f}$= 0.4 (hexane/ethyl acetate = 8/1);

$[\alpha]_D^{25}$ = -161.4 (*c* = 0.1 in $CHCl_3$);

**IR (film)** $\lambda_{max}$ 3152, 2978, 2941, 2873, 1719, 1706, 1613, 1565, 1472, 1383, 1301, 1132, 851;

**$^{1}$H NMR** (400 MHz, $CDCl_3$) $\delta$ 17.02 (s, 1H), 11.41 (s, 1H), 10.80 (s, 1H), 4.06 – 4.00 (m, 1H), 3.68 (d, *J* = 11.2 Hz, 1H), 3.42 (d, *J* = 1.9 Hz, 1H), 3.39 – 3.36 (m, 1H), 3.05 – 2.94 (m, 1H), 1.90 (dd, *J* = 10.4, 5.6 Hz, 1H), 1.48 (s, 3H), 1.52 – 1.40 (m, 1H), 1.38 (s, 3H), 1.36 (s, 6H), 1.31 (s, 6H), 1.30 (s, 3H), 1.29 (s, 3H), 1.26 (s, 3H), 1.12 (d, *J* = 4.8 Hz, 3H), 1.11 (d, *J* = 5.2 Hz, 3H), 0.98 (s, 3H), 0.84 (d, *J* = 6.5 Hz, 3H), 0.67 (d, *J* = 6.3 Hz, 3H);

**$^{13}$C NMR** (100 MHz, $CDCl_3$) $\delta$ 217.9, 212.9, 212.1, 204.0, 176.9, 163.1, 162.5, 154.5, 114.1,

114.3, 108.2, 103.1, 102.5, 85.2, 54.6, 54.0, 50.4, 49.2, 41.3, 39.4, 38.0, 37.9, 35.0, 30.3, 29.1, 28.8, 26.9, 26.2, 25.6, 25.3, 24.6, 23.8, 23.6, 22.2, 22.2, 20.9, 20.5, 19.7;

**HRMS** (ESI) calcd for $C_{38}H_{53}O_9$ [(M+H)$^+$] Exact Mass: 653.3684; found: 653.3682.

**Table S4. Compared NMR data [$CDCl_3$] between our synthetic (-)-myrtucommuacetalone B (3) and the isolated natural product.**

| | *$^1H$ & ppm (J)* | | | *$^{13}C$ & ppm* | | |
|---|---|---|---|---|---|---|
| position | isolated (500M) | synthesized (400M) | error (iso. - syn.) | isolated (125M) | synthesized (100M) | error (iso. - syn.) |
| 1 | - | - | - | 114.1 | 114.1 | 0 |
| 3 | - | - | - | 162.5 | 162.5 | 0 |
| 4 | - | - | - | 102.5 | 102.5 | 0 |
| 5 | 17.01*(s, 1H) | 17.01*(s, 1H) | 0 | 163.1 | 163.1 | 0 |
| 6 | - | - | - | 108.3 | 108.2 | 0.1 |
| 7 | 10.80*(s, 1H) | 10.80*(s, 1H) | 0 | 154.5 | 154.5 | 0 |
| 8 | - | - | - | 103.2 | 103.1 | 0.1 |
| 9 | 3.43 (d, *J* = 3.0 Hz, 1H) | 3.42 (d, *J* = 1.9 Hz, 1H) | 0.01 | 38.0 | 38.0 | 0 |
| 10 | 3.38 (m, 1H) | 3.37 (m, 1H) | 0.01 | 35.1 | 35.0 | 0.1 |
| 11 | 1.89 (dd, *J* = 12.2, 7.4 Hz, 1H) | 1.90 (dd, *J* = 10.4, 5.6 Hz, 1H) | -0.01 | 39.4 | 39.4 | 0 |
| | 1.44 (m, 1H) | 1.44 (m, 1H) | 0 | - | - | - |
| 12 | - | - | - | 85.2 | 85.2 | 0 |
| 14 | - | - | - | 54.1 | 54.0 | 0.1 |
| 15 | - | - | - | 218.0 | 217.9 | 0.1 |
| 16 | - | - | - | 50.4 | 50.4 | 0 |
| 17 | 3.67 (d, *J* = 3.5 Hz, 1H) | 3.68 (d, *J* = 11.2 Hz, 1H) | -0.01 | 41.4 | 41.3 | 0.1 |
| 18 | 3.02 (m, 1H) | 3.02 (m, 1H) | 0 | 26.2 | 26.2 | 0 |
| 19 | 0.67 (d, *J* = 6.6 Hz, 3H) | 0.67 (d, *J* = 6.3 Hz, 3H) | 0 | 22.2 | 22.2 | 0 |
| 20 | 0.84 (d, *J* = 6.6 Hz, 3H) | 0.84 (d, *J* = 6.5 Hz, 3H) | 0 | 22.2 | 22.2 | 0 |
| 21 | 1.29 (s, 3H) | 1.29 (s, 3H) | 0 | 30.4 | 30.3 | 0.1 |
| 22 | 1.32 (s, 3H) | 1.31 (s, 3H) | 0.01 | 28.8 | 28.8 | 0 |
| 23 | 1.30 (s, 3H) | 1.30 (s, 3H) | 0 | 19.8 | 19.7 | 0.1 |
| 24 | 1.26 (s, 3H) | 1.26 (s, 3H) | 0 | 24.7 | 24.6 | 0.1 |
| 25 | 0.98 (s, 3H) | 0.98 (s, 3H) | 0 | 23.7 | 23.6 | 0.1 |
| 26 | 1.38 (s, 3H) | 1.38 (s, 3H) | 0 | 29.2 | 29.1 | 0.1 |
| 1' | - | - | - | 114.3 | 114.3 | 0 |
| 2' | - | - | - | 204.0 | 204.0 | 0 |
| 3 | - | - | - | 54.6 | 54.6 | 0 |
| 4' | - | - | - | 213.0 | 212.9 | 0.1 |
| 5' | - | - | - | 49.3 | 49.2 | 0.1 |
| 6' | 11.42*(s, 1H) | 11.41*(s, 1H) | 0.01 | 177.0 | 176.9 | 0.1 |
| 7' | - | - | - | 212.2 | 212.1 | 0.1 |
| 8' | 4.03 (m, 1H) | 4.03 (m, 1H) | 0 | 37.9 | 37.9 | 0 |
| 9' | 1.12 (d, *J* = 6.7 Hz, 3H) | 1.12 (d, *J* = 4.8 Hz, 3H) | 0 | 21.0 | 20.9 | 0.1 |
| 10' | 1.11 (d, *J* = 6.7 Hz, 3H) | 1.11 (d, *J* = 5.2 Hz, 3H) | 0 | 20.6 | 20.5 | 0.1 |
| 11' | 1.31 (s, 3H) | 1.31 (s, 3H) | 0 | 23.9 | 23.8 | 0.1 |
| 12' | 1.36 (s, 3H) | 1.36 (s, 3H) | 0 | 26.9 | 26.9 | 0 |
| 13' | 1.48 (s, 3H) | 1.48 (s, 3H) | 0 | 25.2 | 25.3 | -0.1 |
| 14' | 1.36 (s, 3H) | 1.36 (s, 3H) | 0 | 25.6 | 25.6 | 0 |

*OH

## 2.10 Synthesis of 6a

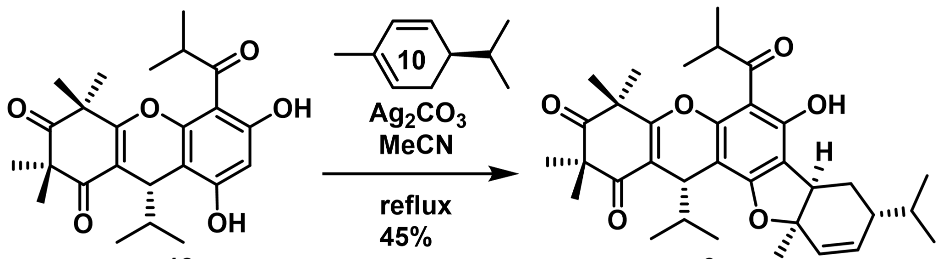


***ent*-13** **6a**

Compound **10** [(-)-$\alpha$-phellandrene; 65 wt%, 4.0 g, 19.3 mmol, 8 equiv.] and compound ***ent*-13** (1.0 g, 2.4 mmol) under argon were added to a solution of silver carbonate ($Ag_2CO_3$; 2.6 g, 9.6

mmol, 4 equiv.) and celite (2.6 g) in acetonitrile (MeCN; 24 mL), and the resulting solution was refluxed for 8 h. The mixture was cooled down to 26 °C, filtered and concentrated in vacuo. The crude residue was purified by silica gel column chromatography (1%-2% hexanes/ethyl acetate) to afford the title compound **6a** (590 mg, 45%) as white crystals.

Compound **6a**: mp = 53-55 °C;

**R$_f$**= 0.4 (hexane/ethyl acetate = 20/1);

$[\alpha]_D^{25}$ = -140.6 (*c* = 0.1 in MeOH);

**IR (film)** $\lambda_{max}$ 3190, 2967, 2935, 2872, 1719, 1654, 1624, 1466, 1427, 1383, 1156, 1060, 969, 844;

**$^1$H NMR** (400 MHz, $CDCl_3$) $\delta$ 13.40 (s, 1H), 5.86 (dd, *J* = 10.3, 2.0 Hz, 1H), 5.62 (dd, *J* = 10.3, 2.2 Hz, 1H), 4.08 (d, *J* = 3.4 Hz, 1H), 3.94 – 3.88 (m, 1H), 3.54 (t, *J* = 4.7 Hz, 1H), 2.47 – 2.41 (m, 1H), 1.93 – 1.89 (m, 1H), 1.87 – 1.81 (m, 1H), 1.68 – 1.61 (m, 2H), 1.58 (s, 3H), 1.58 (s, 3H), 1.41 (s, 3H), 1.41 (s, 3H), 1.37 (s, 3H), 1.23 (t, *J* = 7.0 Hz, 6H), 0.91 (s, 3H), 0.89 (s, 3H), 0.83 (d, *J* = 6.9 Hz, 3H), 0.72 (d, *J* = 6.9 Hz, 3H);

**$^{13}$C NMR** (100 MHz, $CDCl_3$) $\delta$ 212.1, 209.1, 197.4, 167.4, 163.1, 160.5, 153.5, 135.2, 129.2, 112.2, 112.2, 103.8, 99.4, 89.3, 56.1, 47.3, 44.9, 39.5, 37.9, 34.6, 32.3, 31.4, 26.4, 25.7, 25.1, 25.1, 24.6, 24.3, 21.1, 19.7, 19.6, 19.4, 18.1, 17.8;

**HRMS** (ESI) calcd for $C_{34}H_{45}O_6$ [(M+H)$^+$] Exact Mass: 549.3211; found: 549.3210.

**Synthesis of callistrilone D (6)**

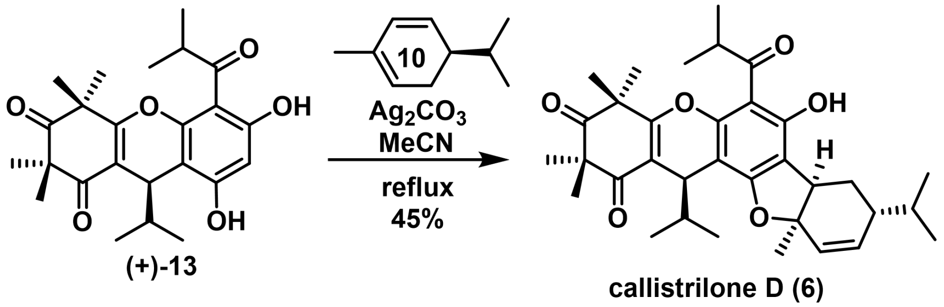


Compound **10** [(-)-$\alpha$-phellandrene; 65 wt%, 4.0 g, 19.3 mmol, 8 equiv.] and compound (+)-**13** (1 g, 2.4 mmol) under argon were added to a solution of silver carbonate ($Ag_2CO_3$; 2.6 g, 9.6mmol, 4 equiv.) and celite (1.3 g) in acetonitrile (MeCN; 24 mL), and the resulting solution was refluxed for 8 h. The mixture was cooled down to 26 °C, filtered and concentrated in vacuo. The crude residue was purified by silica gel column chromatography (1%-2% hexanes/ethyl

acetate) to afford the title compound callistrilone D (**6**) (590 mg, 45%) as white crystals.

Compound callistrilone D (**6**): mp = 48-49 °C;

**R$_f$**= 0.4 (hexane/ethyl acetate = 20/1);

$[\alpha]_D^{25}$ = +112.1 ($c$ = 0.1 in MeOH);

**IR (film)** $\lambda_{max}$ 3200, 2959, 2925, 2880, 1717, 1655, 1624, 1593, 1458, 1383, 1260, 1061, 802;

**$^1$H NMR** (500 MHz, $CDCl_3$) $\delta$ 13.40 (s, 1H), 5.85 (d, $J$ = 10.3 Hz, 1H), 5.58 (d, $J$ = 10.3 Hz, 1H), 4.05 (d, $J$ = 3.3 Hz, 1H), 3.91 – 3.83 (m, 1H), 3.47 (t, $J$ = 4.6 Hz, 1H), 2.44 – 2.40 (m, 1H), 1.99 (brs, 1H), 1.83 – 1.77 (m, 1H), 1.67 – 1.61 (m, 2H), 1.56 (s, 3H), 1.53 (s, 3H), 1.39 (s, 6H), 1.35 (s, 3H), 1.23 (s, 3H), 1.22 (s, 3H), 0.91 (d, $J$ = 2.1 Hz, 3H), 0.90 (d, $J$ = 2.3 Hz, 3H), 0.80 (d, $J$ = 6.9 Hz, 3H), 0.68 (d, $J$ = 6.8 Hz, 3H);

**$^{13}$C NMR** (125 MHz, $CDCl_3$) $\delta$ 212.3, 209.2, 197.6, 167.7, 163.2, 161.1, 153.9, 135.3, 129.1, 112.6, 112.3, 103.9, 99.4, 89.9, 56.3, 47.4, 45.1, 39.7, 38.0, 34.7, 32.3, 31.6, 26.5, 25.7, 25.3, 25.1, 24.7, 24.4, 21.3, 19.9, 19.7, 18.1, 18.1;

**HRMS** (ESI) calcd for $C_{34}H_{45}O_6$ [$(M+H)^+$] Exact Mass: 549.3211; found: 549.3207.

**Table S5. Compared NMR data [$CDCl_3$] between our synthetic callistrilone D (6) and the isolated natural product.**

| position | $^1H$ & ppm (J) isolated (500M) | synthesized (500M) | error (iso. - syn.) | $^{13}C$ & ppm isolated (125M) | synthesized (125M) | error (iso. - syn.) |
|---|---|---|---|---|---|---|
| 1 | - | - | - | 197.6 | 197.6 | 0 |
| 2 | - | - | - | 56.3 | 56.3 | 0 |
| 3 | - | - | - | 212.3 | 212.3 | 0 |
| 4 | - | - | - | 47.5 | 47.4 | 0.1 |
| 4a | - | - | - | 167.7 | 167.7 | 0 |
| 5a | - | - | - | 154.0 | 153.9 | 0.1 |
| 6 | - | - | - | 103.9 | 103.9 | 0 |
| 7 | 13.39 (s, 1H) | 13.40 (s, 1H) | -0.01 | 161.2 | 161.1 | 0.1 |
| 7a | - | - | - | 112.7 | 112.6 | 0.1 |
| 7b | 3.48 (dd, *J* = 7.0, 4.5 Hz, 1H) | 3.47 (t, *J* = 4.6 Hz, 1H) | 0.01 | 45.1 | 45.1 | 0 |
| 8 | 2.42 (m, 1H) | 2.42 (m, 1H) | 0 | 25.8 | 25.7 | 0.1 |
| | 1.64 (m, 1H) | 1.64 (m, 1H) | 0 | - | - | - |
| 9 | 1.99 (m, 1H) | 1.99 (brs, 1H) | 0 | 38.1 | 38.0 | 0.1 |
| 10 | 5.58 (dd, *J* = 10.3, 2.0 Hz, 1H) | 5.58 (d, *J* = 10.3 Hz, 1H) | 0 | 129.2 | 129.1 | 0.1 |
| 11 | 5.85 (dd, *J* = 10.3, 3.3 Hz, 1H) | 5.85 (d, *J* = 10.3 Hz, 1H) | 0 | 135.3 | 135.3 | 0 |
| 11a | - | - | - | 89.9 | 89.9 | 0 |
| 12a | - | - | - | 163.3 | 163.2 | 0.1 |
| 13 | 4.05 (d, *J* = 3.6 Hz, 1H) | 4.05 (d, *J* = 3.3 Hz, 1H) | 0 | 32.4 | 32.3 | 0.1 |
| 13a | - | - | - | 112.4 | 112.3 | 0.1 |
| 1' | 1.35 (s, 3H) | 1.35 (s, 3H) | 0 | 24.7 | 24.7 | 0 |
| 2' | 1.39 (s, 3H) | 1.39 (s, 3H) | 0 | 24.5 | 24.4 | 0.1 |
| 3' | 1.39 (s, 3H) | 1.39 (s, 3H) | 0 | 25.3 | 25.3 | 0 |
| 4' | 1.56 (s, 3H) | 1.56 (s, 3H) | 0 | 25.0 | 25.1 | 0.1 |
| 5' | - | - | - | 209.3 | 209.2 | 0.1 |
| 6' | 3.86 (m, 1H) | 3.87 (m, 1H) | -0.01 | 39.7 | 39.7 | 0 |
| 7' | 1.22 (d, *J* = 7.0 Hz, 3H) | 1.23 (s, 3H) | -0.01 | 18.1 | 18.1 | 0 |
| 8' | 1.22 (d, *J* = 7.0 Hz, 3H) | 1.22 (s, 3H) | 0 | 21.3 | 21.3 | 0 |
| 9' | 1.61 (m, 1H) | 1.61 (m, 1H) | 0 | 31.7 | 31.6 | 0.1 |
| 10' | 0.91 (d, *J* = 6.7 Hz, 3H) | 0.91 (d, *J* = 2.1 Hz, 3H) | 0 | 19.8 | 19.7 | 0.1 |
| 11' | 0.91 (d, *J* = 6.7 Hz, 3H) | 0.90 (d, *J* = 2.3 Hz, 3H) | 0.01 | 20.0 | 19.9 | 0.1 |
| 12' | 1.54 (s, 3H) | 1.53 (s, 3H) | 0.01 | 26.5 | 26.5 | 0 |
| 13' | 1.80 (m, 1H) | 1.80 (m, 1H) | 0 | 34.8 | 34.7 | 0.1 |
| 14' | 0.80 (d, *J* = 6.9 Hz, 3H) | 0.80 (d, *J* = 6.9 Hz, 3H) | 0 | 20.0 | 19.9 | 0.1 |
| 15' | 0.68 (d, *J* = 6.9 Hz, 3H) | 0.68 (d, *J* = 6.8 Hz, 3H) | 0 | 18.2 | 18.1 | 0.1 |

*OH

**Synthesis of callistrilone A (4)**

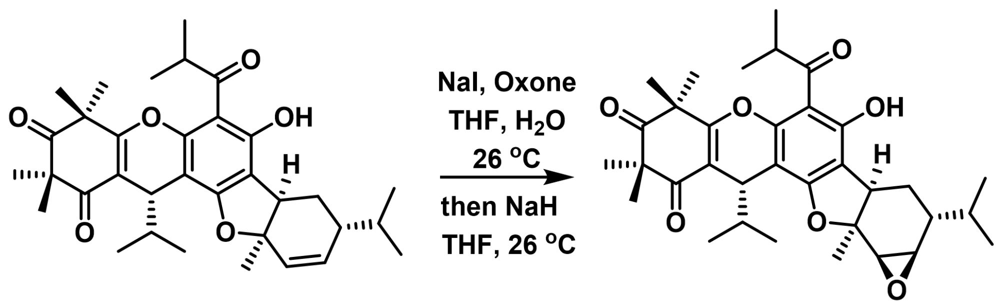


**6a** **callistrilone A (4)**

To a solution of compound **6a** (302 mg, 0.55 mmol) in tetrahydrofuran-water (THF-$H_2O$; 5 : 1, v/v) (10 mL) were added potassium peroxomonosulfate (Oxone; 4.1 g, 6.6 mmol, 12 equiv.), sodium iodide dihydrate (NaI; 825 mg, 5.5 mmol, 10 equiv.) in small portions over 5 min. The entire set-up was covered with aluminum foil, placed in the dark and stirred for 10 h until all the starting material was consumed (TLC). The mixture was quenched with saturated aqueous sodium sulfite (10 mL). Then the mixture was extracted with ethyl acetate (3×15 mL) and the combined organic layers were dried over $Na_2SO_4$, filtered and concentrated in vacuo. To a solution of the crude residue (346 mg) in tetrahydrofuran (10 mL) was added sodium hydride (NaH; 60 wt%, 66

mg, 1.65 mmol, 3 equiv.), and the resulting solution was stirred at 26 °C for 2 h. The mixture was quenched with saturated aqueous sodium sulfite (10 mL). Then the mixture was extracted with ethyl acetate (3×15 mL) and the combined organic layers were dried over $Na_2SO_4$, filtered and concentrated in vacuo. The crude residue was purified by silica gel column chromatography (1%-3.3% hexanes/ethyl acetate) to afford the title compound callistrilone A (**4**) (187 mg, 60%) as white crystals.

**Note**: The specific rotations of the natural and synthetic product callistrilone A (**4**) have been tested again.

Callistrilone A (**4**): mp = 247-249 °C;

**$R_f$**= 0.5 (hexane/ethyl acetate = 10/1);

Natural callistrilone A (**4**): $[\alpha]_D^{27}$ = -98.5 ($c$ = 0.2 in MeOH);

Synthetic callistrilone A (**4**): $[\alpha]_D^{27}$ = -103.3 ($c$ = 0.2 in MeOH);

**IR (film)** $\lambda_{max}$ 2978, 2962, 2874, 1719, 1665, 1642, 1589, 1466, 1383, 1244, 1161, 1072, 834;

**$^1$H NMR** (500 MHz, $CDCl_3$) $\delta$ 13.32 (s, 1H), 4.14 (d, $J$ = 3.6 Hz, 1H), 3.94 – 3.80 (m, 1H), 3.45 (t, $J$ = 3.4 Hz, 1H), 3.29 (d, $J$ = 4.1 Hz, 1H), 3.02 (dd, $J$ = 12.4, 5.9 Hz, 1H), 2.15 – 2.08 (m, 1H), 1.92 – 1.84 (m, 1H), 1.84 – 1.78 (m, 1H), 1.66 – 1.57 (m, 2H), 1.55 (s, 3H), 1.48 (s, 3H), 1.40 (s, 3H), 1.38 (s, 3H), 1.36 (s, 3H), 1.22 (d, $J$ = 6.6 Hz, 3H), 1.20 (d, $J$ = 7.1 Hz, 3H), 1.08 (d, $J$ = 4.2 Hz, 3H), 1.07 (d, $J$ = 4.3 Hz, 3H), 0.84 (d, $J$ = 6.9 Hz, 3H), 0.68 (d, $J$ = 6.9 Hz, 3H);

**$^{13}$C NMR** (125 MHz, $CDCl_3$) $\delta$ 212.3, 209.2, 197.3, 167.7, 161.9, 160.4, 154.1, 113.6, 112.6, 104.3, 99.2, 88.6, 56.4, 56.1, 55.1, 47.4, 40.4, 39.7, 39.2, 34.6, 32.6, 28.5, 26.3, 25.4, 25.4, 25.1, 23.8, 23.6, 22.1, 21.5, 21.2, 20.1, 18.0, 18.0;

**HRMS** (ESI) calcd for $C_{34}H_{45}O_7$ [$(M+H)^+$] Exact Mass: 565.3160; found: 565.3149.

**Table S6. Compared NMR data [$CDCl_3$] between our synthetic callistrilone A (4) and the isolated natural product.**

| | $^{1}H$ & ppm (J) | | | $^{13}C$ & ppm | | |
|---|---|---|---|---|---|---|
| position | isolated (500M) | synthesized (500M) | error (iso. - syn.) | isolated (125M) | synthesized (125M) | error (iso. - syn.) |
| 1 | - | - | - | 197.3 | 197.3 | 0 |
| 2 | - | - | - | 56.4 | 56.4 | 0 |
| 3 | - | - | - | 212.3 | 212.3 | 0 |
| 4 | - | - | - | 47.4 | 47.4 | 0 |
| 4a | - | - | - | 167.7 | 167.7 | 0 |
| 5a | - | - | - | 154.1 | 154.1 | 0 |
| 6 | - | - | - | 104.3 | 104.3 | 0 |
| 6a | - | - | - | | | |
| 7 | - | - | - | 160.4 | 160.4 | 0 |
| 7a | - | - | - | 113.6 | 113.6 | 0 |
| 7b | 3.03 (dd, *J* = 12.4, 5.9 Hz, 1H) | 3.02 (dd, *J* = 12.4, 5.9 Hz, 1H) | 0.01 | 40.5 | 40.4 | 0.1 |
| 8 | 2.12 (m, 1H) | 2.11 (m, 1H) | 0.01 | 23.6 | 23.6 | 0 |
| | 1.58 (m, 1H) | 1.58 (m, 1H) | 0 | | | |
| 9 | 1.82 (m, 1H) | 1.81 (m, 1H) | 0.01 | 39.3 | 39.2 | 0.1 |
| 10 | 3.45 (dd, *J* = 4.1, 3.3 Hz, 1H) | 3.45 (t, *J* = 3.4 Hz, 1H) | 0 | 56.1 | 56.1 | 0 |
| 11 | 3.29 (d, *J* = 4.1 Hz, 1H) | 3.29 (d, *J* = 4.1 Hz, 1H) | 0 | 55.1 | 55.1 | 0 |
| 11a | - | - | - | 88.6 | 88.6 | 0 |
| 11b | - | - | - | | | |
| 12 | - | - | - | | | |
| 12a | - | - | - | 161.9 | 161.9 | 0 |
| 12b | - | - | - | 99.3 | 99.2 | 0.1 |
| 13 | 4.15 (d, *J* = 3.6 Hz, 1H) | 4.14 (d, *J* = 3.6 Hz, 1H) | 0.01 | 32.6 | 32.6 | 0 |
| 13a | - | - | - | 112.6 | 112.6 | 0 |
| 1' | 1.38 (s, 3H) | 1.38 (s, 3H) | 0 | 23.9 | 23.8 | 0.1 |
| 2' | 1.37 (s, 3H) | 1.36 (s, 3H) | 0.01 | 25.4 | 25.4 | 0 |
| 3' | 1.56 (s, 3H) | 1.55 (s, 3H) | 0.01 | 25.1 | 25.1 | 0 |
| 4' | 1.40 (s, 3H) | 1.40 (s, 3H) | 0 | 25.4 | 25.4 | 0 |
| 5' | - | - | - | 209.2 | 209.2 | 0 |
| 6' | 3.87 (m, 1H) | 3.87 (m, 1H) | 0 | 39.7 | 39.7 | 0 |
| 7' | 1.20 (d, *J* = 7.0 Hz, 3H) | 1.20 (d, *J* = 7.1 Hz, 3H) | 0 | 18.0 | 18.0 | 0 |
| 8' | 1.22 (d, *J* = 6.6 Hz, 3H) | 1.22 (d, *J* = 6.6 Hz, 3H) | 0 | 21.2 | 21.2 | 0 |
| 9' | 1.62 (m, 1H) | 1.62 (m, 1H) | 0 | 28.5 | 28.5 | 0 |
| 10' | 1.09 (d, *J* = 6.5 Hz, 3H) | 1.08 (d, *J* = 4.2 Hz, 3H) | 0.01 | 22.1 | 22.1 | 0 |
| 11' | 1.08 (d, *J* = 6.5 Hz, 3H) | 1.07 (d, *J* = 4.3 Hz, 3H) | 0.01 | 21.5 | 21.5 | 0 |
| 12' | 1.48 (s, 3H) | 1.48 (s, 3H) | 0 | 26.3 | 26.3 | 0 |
| 13' | 1.86 (m, 1H) | 1.86 (m, 1H) | 0 | 34.7 | 34.6 | 0.1 |
| 14' | 0.69 (d, *J* = 6.9 Hz, 3H) | 0.68 (d, *J* = 6.9 Hz, 3H) | 0.01 | 18.0 | 18.0 | 0 |
| 15' | 0.85 (d, *J* = 6.9 Hz, 3H) | 0.84 (d, *J* = 6.9 Hz, 3H | 0.01 | 20.1 | 20.1 | 0 |
| 7-OH* | 13.31 (s, 1H) | 13.32 (s, 1H) | -0.01 | | | |

*OH

**Synthesis of callistrilone C (5)**

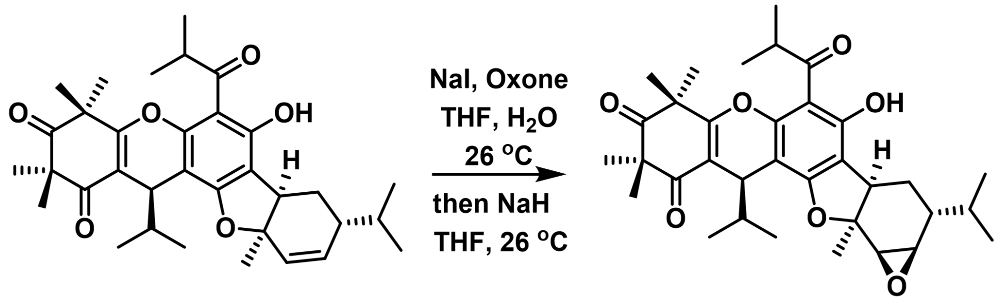


6

callistrilone C (5)

To a solution of compound **6** (200 mg, 0.365 mmol) in tetrahydrofuran-water (THF-$H_2O$; 5 : 1, v/v) (10 mL) were added potassium peroxomonosulfate (Oxone; 2.7 g, 4.38 mmol, 12 equiv.), sodium iodide dihydrate (NaI; 548 mg, 3.65 mmol, 10 equiv.) in small portions over 5 min. The entire set-up was covered with aluminum foil, placed in the dark and stirred until all the starting material was consumed (TLC). The mixture was quenched with saturated aqueous sodium sulfite (10 mL). The mixture was extracted with ethyl acetate (3×10 mL) and the combined organic layers

were dried over $Na_2SO_4$, filtered and concentrated in vacuo. To a solution of the crude residue (235 mg) in tetrahydrofuran (10 mL) was added sodium hydride (NaH; 60 wt%, 43.8 mg, 1.1 mmol, 3 equiv.), and the resulting solution was stirred at 26 °C for 2 h. The mixture was quenched with saturated aqueous sodium sulfite (10 mL). Then the mixture was extracted with ethyl acetate (3×10 mL) and the combined organic layers were dried over $Na_2SO_4$, filtered and concentrated in vacuo. The crude residue was purified by silica gel column chromatography (1%-3.3% hexanes/ethyl acetate) to afford the title compound callistrilone C (**5**) (124 mg, 60%) as white crystals.

Compound callistrilones C (**5**): mp = 167-169 °C;

**$R_f$**= 0.5 (hexane/ethyl acetate = 10/1);

$[\alpha]_D^{25}$ = +91.5 (*c* = 0.1 in MeOH);

**IR (film)** $\lambda_{max}$ 2963, 2922, 2851, 1719, 1655, 1621, 1460, 1385, 1242, 1155, 1070, 845;

**$^1$H NMR** (500 MHz, $CDCl_3$) $\delta$ 13.31 (s, 1H), 4.10 (d, $J$ = 3.5 Hz, 1H), 3.94 – 3.79 (m, 1H), 3.42 (t, $J$ = 3.3 Hz, 1H), 3.26 (d, $J$ = 4.1 Hz, 1H), 3.01 (dd, $J$ = 12.2, 5.9 Hz, 1H), 2.14 – 2.05 (m, 1H), 1.90 – 1.85 (m, 1H), 1.83 – 1.79 (m, 1H), 1.71 – 1.58 (m, 2H), 1.57 (s, 3H), 1.45 (s, 3H), 1.40 (s, 6H), 1.35 (s, 3H), 1.21 (d, $J$ = 6.4 Hz, 6H), 1.08 (d, $J$ = 6.3 Hz, 3H), 1.06 (d, $J$ = 6.4 Hz, 3H), 0.90 (d, $J$ = 6.9 Hz, 3H), 0.71 (d, $J$ = 6.9 Hz, 3H);

**$^{13}$C NMR** (125 MHz, $CDCl_3$) $\delta$ 212.2, 209.2, 197.7, 167.5, 162.1, 160.4, 153.6, 113.5, 112.4, 104.1, 99.1, 88.4, 56.3, 56.0, 55.1, 47.4, 40.6, 39.7, 39.2, 34.8, 32.6, 28.5, 26.2, 25.2, 25.2, 24.8, 24.3, 23.7, 22.1, 21.4, 21.2, 19.7, 18.1, 18.0;

**HRMS** (ESI) calcd for $C_{34}H_{45}O_7$ [$(M+H)^+$] Exact Mass: 565.3160; found: 565.3151.

**Table S7. Compared NMR data [$CDCl_3$] between our synthetic callistrilone C (5) and the isolated natural product.**

| | $^1H$ δ ppm (J) | | | $^{13}C$ δ ppm | | |
|---|---|---|---|---|---|---|
| position | isolated (500M) | synthesized (500M) | error (iso. - syn.) | isolated (125M) | synthesized (125M) | error (iso. - syn.) |
| 1 | - | - | - | 197.8 | 197.7 | 0.1 |
| 2 | - | - | - | 56.4 | 56.3 | 0.1 |
| 3 | - | - | - | 212.2 | 212.2 | 0 |
| 4 | - | - | - | 47.5 | 47.4 | 0.1 |
| 4a | - | - | - | 167.5 | 167.5 | 0 |
| 5a | - | - | - | 153.6 | 153.6 | 0 |
| 6 | - | - | - | 104.2 | 104.1 | 0.1 |
| 7 | 13.30* (s, 1H) | 13.31* (s, 1H) | -0.01 | 160.4 | 160.4 | 0 |
| 7a | - | - | - | 113.5 | 113.5 | 0 |
| 7b | 3.02 (dd, *J* = 12.4, 5.9 Hz, 1H) | 3.01 (dd, *J* = 12.2, 5.9 Hz, 1H) | 0.01 | 40.7 | 40.6 | 0.1 |
| 8 | 2.10 (m, 1H) | 2.10 (m, 1H) | 0 | 23.8 | 23.7 | 0.1 |
| | 1.67 (m, 1H) | 1.66 (m, 1H) | 0.01 | - | - | - |
| 9 | 1.82 (m, 1H) | 1.81 (m, 1H) | 0.01 | 39.3 | 39.2 | 0.1 |
| 10 | 3.42 (dd, *J* = 4.2, 3.3, 1H) | 3.42 (t, *J* = 3.3 Hz, 1H) | 0 | 56.0 | 56.0 | 0 |
| 11 | 3.26 (d, *J* = 4.2 Hz, 1H) | 3.26 (d, *J* = 4.1 Hz, 1H) | 0 | 55.1 | 55.1 | 0 |
| 11a | - | - | - | 88.4 | 88.4 | 0 |
| 12a | - | - | - | 162.2 | 162.1 | 0.1 |
| 12b | - | - | - | 99.2 | 99.1 | 0.1 |
| 13 | 4.11 (d, *J* = 3.5 Hz, 1H) | 4.10 (d, *J* = 3.5 Hz, 1H) | 0.01 | 32.7 | 32.6 | 0.1 |
| 13a | - | - | - | 112.5 | 112.4 | 0.1 |
| 1' | 1.36 (s, 3H) | 1.35 (s, 3H) | 0.01 | 24.3 | 24.3 | 0 |
| 2' | 1.40 (s, 3H) | 1.40 (s, 3H) | 0 | 25.3 | 25.2 | 0.1 |
| 3' | 1.58 (s, 3H) | 1.57 (s, 3H) | 0.01 | 25.2 | 25.2 | 0 |
| 4' | 1.40 (s, 3H) | 1.40 (s, 3H) | 0 | 24.9 | 24.8 | 0.1 |
| 5' | - | - | - | 209.3 | 209.2 | 0.1 |
| 6' | 3.87 (m, 1H) | 3.87 (m, 1H) | 0 | 39.7 | 39.7 | 0 |
| 7' | 1.21 (d, *J* = 7.0 Hz, 3H) | 1.21 (d, *J* = 6.4 Hz, 3H) | 0 | 18.0 | 18.0 | 0 |
| 8' | 1.21 (d, *J* = 6.9 Hz, 3H) | 1.21 (d, *J* = 6.9 Hz, 3H) | 0 | 21.2 | 21.2 | 0 |
| 9' | 1.63 (m, 1H) | 1.63 (m, 1H) | 0 | 28.6 | 28.5 | 0.1 |
| 10' | 1.08 (d, *J* = 6.5 Hz, 3H) | 1.08 (d, *J* = 6.3 Hz, 3H) | 0 | 22.1 | 22.1 | 0 |
| 11' | 1.08 (d, *J* = 6.5 Hz, 3H) | 1.06 (d, *J* = 6.4 Hz, 3H) | 0.02 | 21.5 | 21.4 | 0.1 |
| 12' | 1.45 (s, 3H) | 1.45 (s, 3H) | 0 | 26.3 | 26.2 | 0.1 |
| 13' | 1.89 (m, 1H) | 1.89 (m, 1H) | 0 | 34.9 | 34.8 | 0.1 |
| 14' | 0.71 (d, *J* = 6.9 Hz, 3H) | 0.71 (d, *J* = 6.9 Hz, 3H) | 0 | 18.2 | 18.1 | 0.1 |
| 15' | 0.90 (d, *J* = 6.9 Hz, 3H) | 0.90 (d, *J* = 6.9 Hz, 3H) | 0 | 19.7 | 19.7 | 0 |

*OH

**Synthesis of callistrilone E (7)**

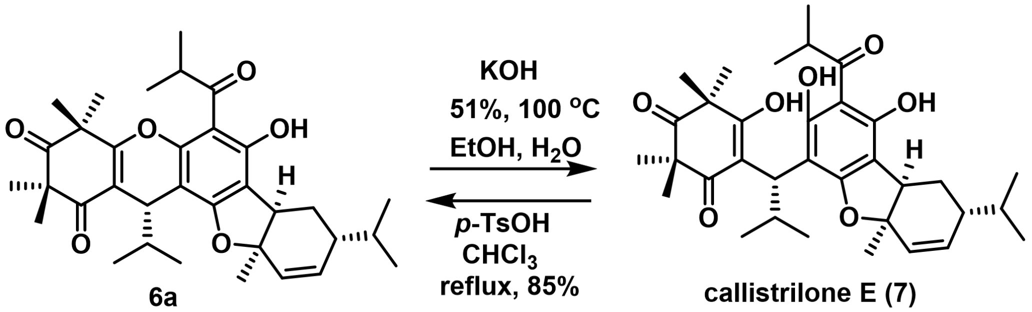


To a solution of compound **6a** (250 mg, 0.46 mmol) in ethyl alcohol -water (EtOH-$H_2O$; 4:1, v/v) (31.3 mL) was added saturated aqueous potassium hydroxide (2.5 mL), and the resulting solution was stirred at 100 °C for 5 h. The mixture was cooled down to 26 °C and quenched with 2M HCl (32 mL). The mixture was extracted with ethyl acetate (3×30 mL) and the combined organic layers were dried over $Na_2SO_4$, filtered and concentrated in vacuo. The crude residue was purified by silica gel column chromatography (1%-2.5% hexanes/ethyl acetate) to afford the title compound callistrilone E (**7**) (132.3 mg, 51%) as yellowish crystals.

**Note**: 1. Both $^1H$ and $^{13}C$ NMR spectra of the myrtucommulone E (**7**) showed doubled signal

patterns, which was probably because of the presence of rotamers or keto-enol tautomers. Further cyclization of compound **7** was carried out to afford **6a** with a single $^{1}$H NMR signal pattern, suggesting that compound **7** was indeed to exist as rotamers or keto-enol tautomers.

2. The potassium hydroxide (KOH) concentration would be kept at about 1.5 mol/L and the reaction temperature would be controlled at 100 °C, at the same time the ratio of ethyl alcohol and water would be from 3:1 to 5:1, and of course in this condition some substrate **6a** and product myrtucommulone E (**7**) would be decomposed. If the concentration of KOH too high, the substrate **6a** and product myrtucommulone E (**7**) may be decomposed and the yield will be decreased. If the concentration of KOH or temperature or the rate of ethyl alcohol is too low, the reaction would be very slow, even not take place.

To a solution of callistrilone E (**7**) (56.6 mg, 0.1 mmol) in chloroform ($CHCl_3$; 3 mL) under argon were added *p*-toluenesulfonic acid (*p*-TsOH; 9.5 mg, 0.05 mmol, 0.5 equiv.), and the resulting mixture was warmed up and refluxed for 3 h. The mixture was cooled down to 26 $^{o}$C and quenched with saturated aqueous sodium bicarbonate (5 mL). The mixture was extracted with dichloromethane (3×5 mL). The combined organic layers were dried over $Na_2SO_4$, filtered and concentrated in vacuo. The crude residue was purified by silica gel column chromatography (1%-2.5% hexanes/ethyl acetate) to afford the mixture of compound **6a** (46.6 mg, 85%) as white crystals.

Compound callistrilone E (**7**): mp = 140-141 $^{o}$C;

**R$_f$**= 0.3 (hexane/ethyl acetate = 20/1);

$[\alpha]_D^{25}$ = -22.5 (*c* = 0.1 in MeOH);

**IR (film)** $\lambda_{max}$ 3238, 2965, 2931, 2871, 1719, 1632, 1591, 1466, 1436, 1384, 1233, 1159, 1060, 869;

**$^{1}$H NMR** (400 MHz, $CDCl_3$) $\delta$ 13.82 (s, 1H), 10.21 (s, 1H), 9.72 (s, 1H), 5.94 (d, *J* = 10.2 Hz, 1H), 5.62 (d, *J* = 10.2 Hz, 1H), 4.09 – 4.02 (m, 1H), 3.72 (d, *J* = 11.5 Hz, 1H), 3.45 (t, *J* = 4.6 Hz, 1H), 2.91 – 2.79 (m, 1H), 2.43 – 2.38 (m, 1H), 1.97 (brs, 1H), 1.64 (s, 3H), 1.69 – 1.58 (m, 2H), 1.48 (s, 3H), 1.40 (s, 3H), 1.34 (s, 3H), 1.31 (s, 3H), 1.17 (d, *J* = 3.8 Hz, 3H), 1.17 (d, *J* = 3.8 Hz, 3H), 0.89 (s, 3H), 0.89 (s, 3H), 0.86 (d, *J* = 3.4 Hz, 3H), 0.84 (d, *J* = 3.0 Hz, 3H);

**$^{13}$C NMR** (100 MHz, $CDCl_3$) $\delta$ 212.4, 212.4, 202.9, 174.9, 161.5, 160.4, 160.0, 137.0, 128.1,

114.8, 107.3, 105.9, 103.4, 91.3, 55.3, 49.0, 44.1, 40.3, 39.7, 38.0, 31.5, 27.3, 27.2, 26.2, 26.1, 25.8, 24.8, 24.7 , 22.0, 21.9, 19.9, 19.8, 19.7, 18.9;

**HRMS** (ESI) calcd for $C_{34}H_{47}O_7$ [(M+H)$^+$] Exact Mass:567.3316; found:567.3301.

**Table S8. Compared NMR data [CDCl3] between our synthetic callistrilone E (7) and the isolated natural product.**

| | $^1H$ & ppm (J) | | | $^{13}C$ & ppm | | |
|---|---|---|---|---|---|---|
| position | isolated (500M) | synthesized (400M) | error (iso. - syn.) | isolated (125M) | synthesized (100M) | error (iso. - syn.) |
| 1 | - | - | - | 202.9 | 202.9 | 0 |
| 2 | - | - | - | 55.3 | 55.3 | 0 |
| 3 | - | - | - | 212.4 | 212.4 | 0 |
| 4 | - | - | - | 49.0 | 49.0 | 0 |
| 4a | 9.73* (s, 1H) | 9.72* (s, 1H) | 0.01 | 174.9 | 174.9 | 0 |
| 5a | 10.21* (s, 1H) | 10.21* (s, 1H) | 0 | 160.0 | 160.0 | 0 |
| 6 | - | - | - | 107.3 | 107.3 | 0 |
| 7 | 13.80* (s, 1H) | 13.82* (s, 1H) | -0.02 | 160.4 | 160.4 | 0 |
| 7a | - | - | - | 105.9 | 105.9 | 0 |
| 7b | 3.45 (dd, *J* = 5.8, 4.5) | 3.45 (t, *J* = 4.6 Hz, 1H) | 0 | 44.1 | 44.1 | 0 |
| 8 | 2.40 (m, 1H) | 2.40 (m, 1H) | 0 | 26.0 | 26.1 | -0.1 |
| | 1.67 (m, 1H) | 1.67 (m, 1H) | 0 | | | |
| 9 | 1.98 (m, 1H) | 1.97 (brs, 1H) | 0.01 | 38.0 | 38.0 | 0 |
| 10 | 5.94 (dd, *J* = 10.3, 2.2, 1H) | 5.94 (d, *J* = 10.2 Hz, 1H) | 0 | 137.0 | 137.0 | 0 |
| 11 | 5.62 (dd, *J* = 10.2, 2.0, 1H) | 5.62 (d, *J* = 10.2 Hz, 1H) | 0 | 128.1 | 128.1 | 0 |
| 11a | - | - | - | 91.3 | 91.3 | 0 |
| 12a | - | - | - | 161.5 | 161.5 | 0 |
| 12b | - | - | - | 103.4 | 103.4 | 0 |
| 13 | 3.72 (d, *J* = 11.5 Hz, 1H) | 3.72 (d, *J* = 11.5 Hz, 1H) | 0 | 40.3 | 40.3 | -0 |
| 13a | - | - | - | 114.8 | 114.8 | 0 |
| 1' | 1.40 (s, 3H) | 1.40 (s, 3H) | 0 | 27.3 | 27.3 | 0 |
| 2' | 1.31 (s, 3H) | 1.31 (s, 3H) | 0 | 25.0 | 24.8 | 0.2 |
| 3' | 1.49 (s, 3H) | 1.48 (s, 3H) | 0.01 | 24.8 | 24.7 | 0.1 |
| 4' | 1.32 (s, 3H) | 1.34 (s, 3H) | -0.02 | 25.8 | 25.8 | 0 |
| 5' | - | - | - | 212.4 | 212.4 | 0 |
| 6' | 4.04 (m, 1H) | 4.04 (m, 1H) | 0 | 39.7 | 39.7 | 0 |
| 7' | 1.17 (d, *J* = 6.9 Hz, 3H) | 1.17 (d, *J* = 3.8 Hz, 3H) | 0 | 19.9 | 19.9 | 0 |
| 8' | 1.17 (d, *J* = 6.9 Hz, 3H) | 1.17 (d, *J* = 3.8 Hz, 3H) | 0 | 18.9 | 18.9 | 0 |
| 9' | 1.59 (m, 1H) | 1.59 (m, 1H) | 0 | 31.5 | 31.5 | 0 |
| 10' | 0.89 (d, *J* = 6.5 Hz, 3H) | 0.89 (s, 3H) | 0 | 22.1 | 22.0 | 0 |
| 11' | 0.89 (d, *J* = 6.5 Hz, 3H) | 0.89 (s, 3H) | 0 | 19.8 | 19.8 | 0 |
| 12' | 1.64 (s, 3H) | 1.64 (s, 3H) | 0 | 26.2 | 26.2 | 0 |
| 13' | 2.85 (m, 1H) | 2.85 (m, 1H) | 0 | 27.1 | 27.2 | -0.1 |
| 14' | 0.86 (d, *J* = 6.9 Hz, 3H) | 0.86 (d, *J* = 3.4 Hz, 3H) | 0 | 19.7 | 19.7 | 0 |
| 15' | 0.82 (d, *J* = 6.9 Hz, 3H) | 0.84 (d, *J* = 3.0 Hz, 3H) | -0.02 | 22.0 | 21.9 | 0.1 |

*OH

## References


[6] L. Lv, Y. Li, Y. Zhang, Z. Xie, *Tetrahedron* 2017, **73**, 3691.

[7] M. Hans, M. Charpentier, V. Huch, J. Jauch, T. Bruhn, G. Bringmann, D. Quandt, *J. Nat. Prod.* 2015, **78,** 2381.

[8] M. Charpentier, M. Hans, J. Jauch, *Eur. J. Org. Chem.* 2013, 4078.

## 3. X-ray crystal structures of 3a, 4, 6

### 3.1 X-ray crystal structure of 3a

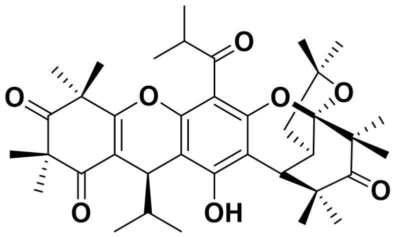

**3a**

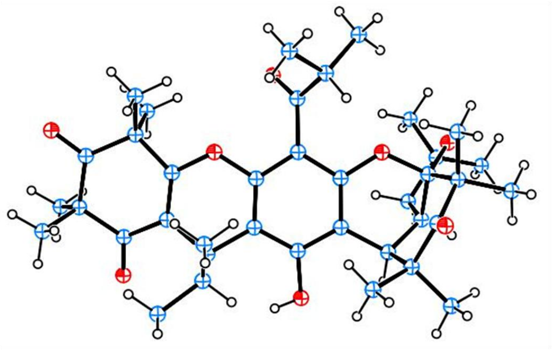

### 3.2 X-ray crystal structure of 4

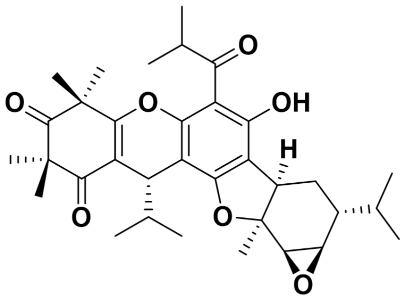

callistrilone A (**4**)

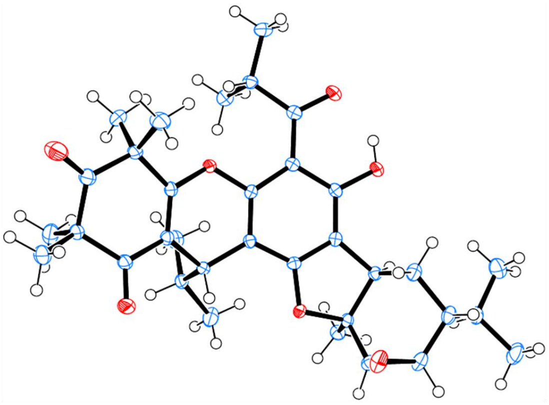

### 3.3 X-ray crystal structure of 6

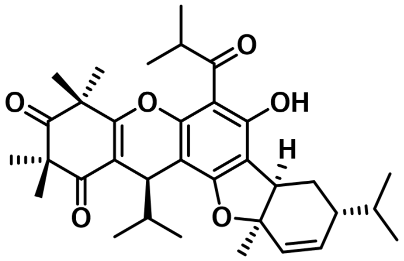

callistrilone D (**6**)

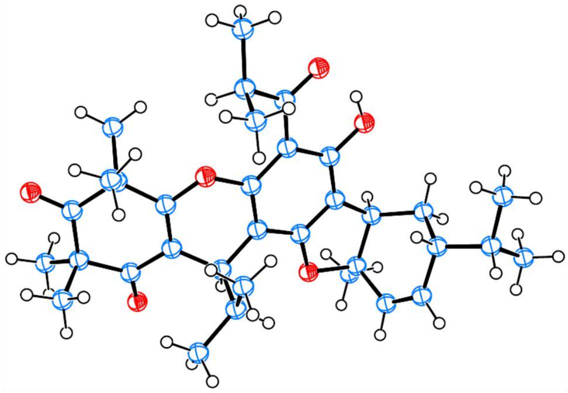

## 4. Synthetic $^{1}H$ and $^{13}C$ NMR Spectra

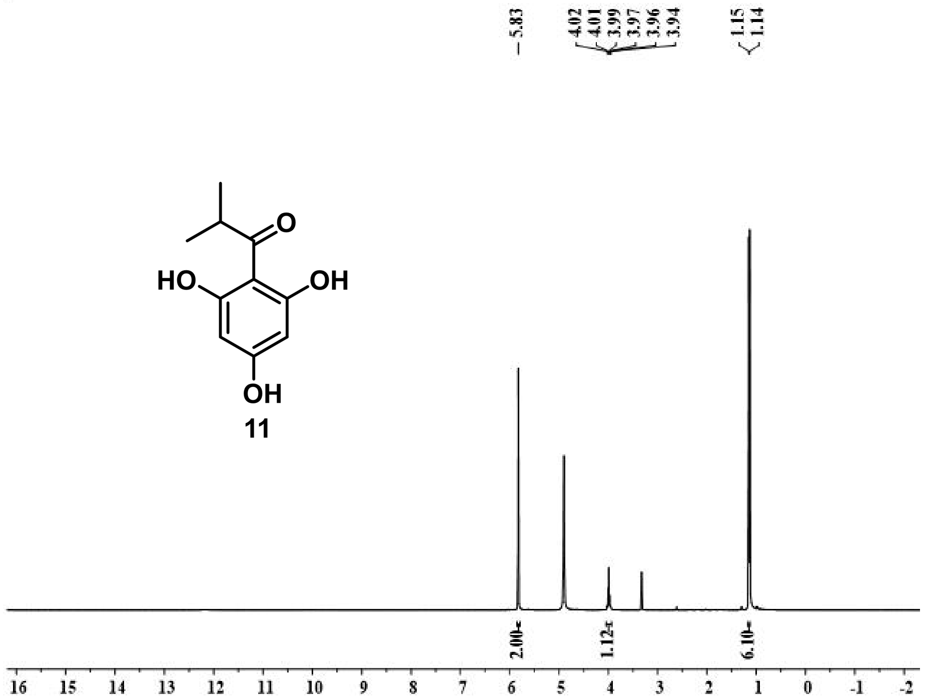


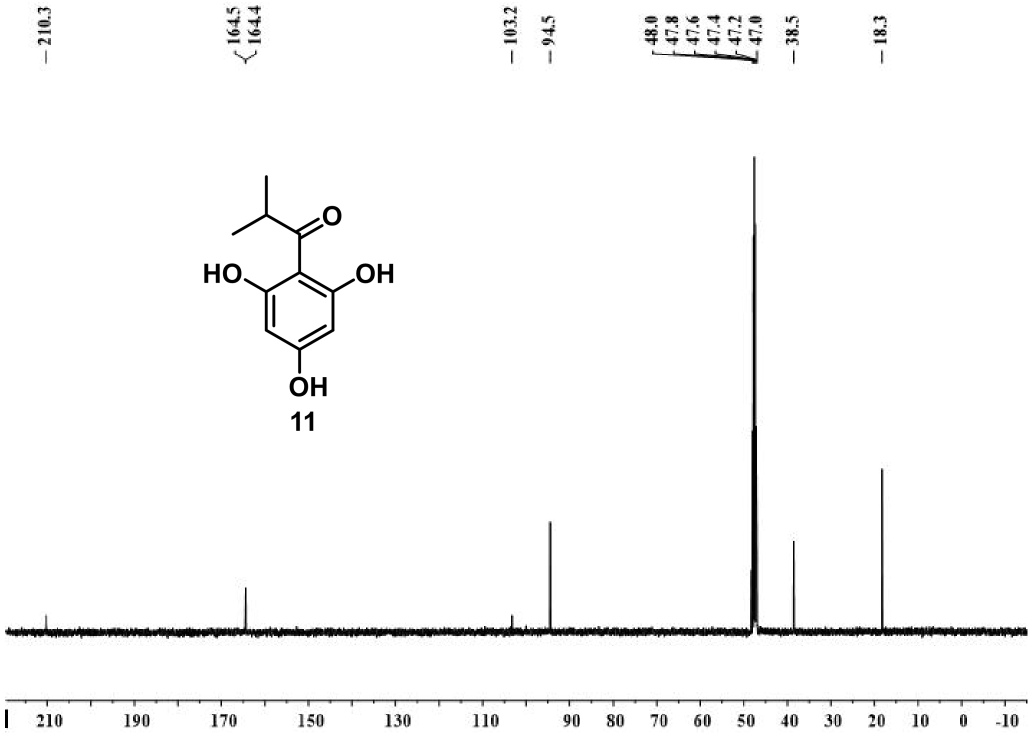

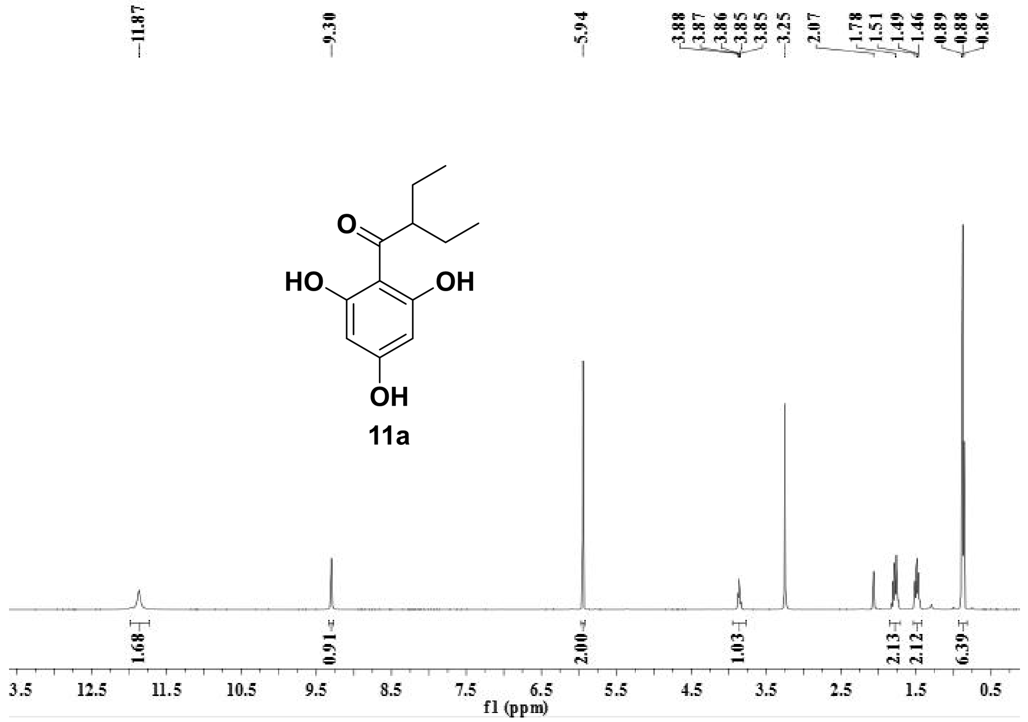
11.87
9.30
5.94
3.88
3.87
3.86
3.85
3.85
3.25
2.07
1.78
1.51
1.49
1.46
0.89
0.88
0.86
O
HO
OH
OH
11a
1.68
0.91
2.00
1.03
2.13
2.12
6.39
12.5
11.5
10.5
9.5
8.5
7.5
6.5
5.5
4.5
3.5
2.5
1.5
0.5
f1 (ppm)

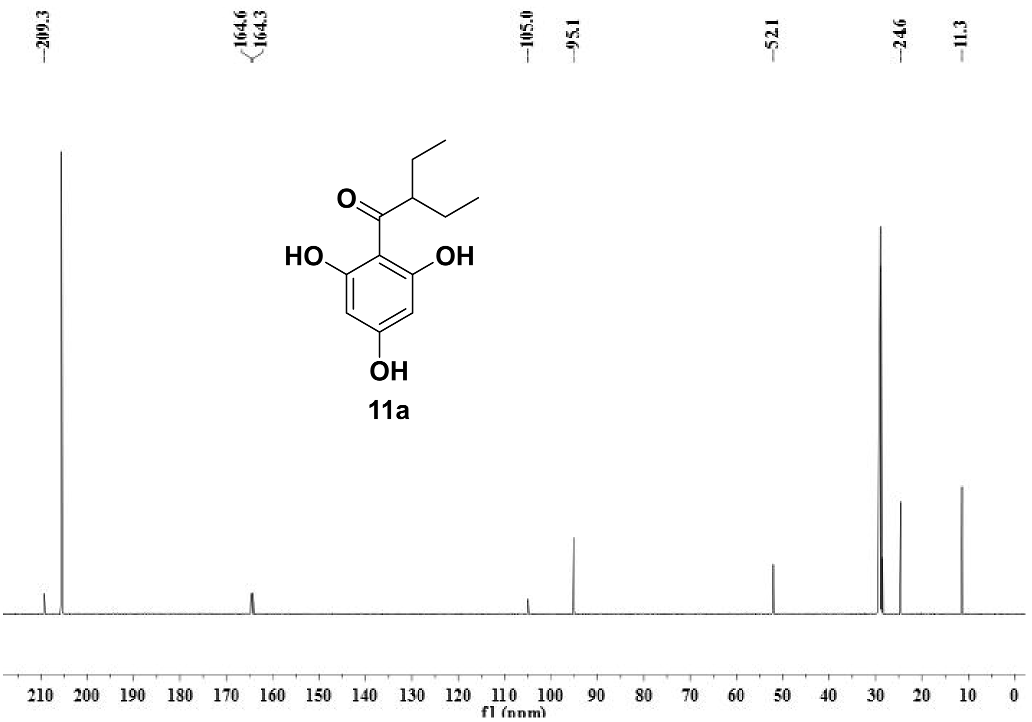
209.3
164.6
164.3
105.0
95.1
52.1
24.6
11.3
O
HO
OH
OH
11a
210
200
190
180
170
160
150
140
130
120
110
100
90
80
70
60
50
40
30
20
10
0

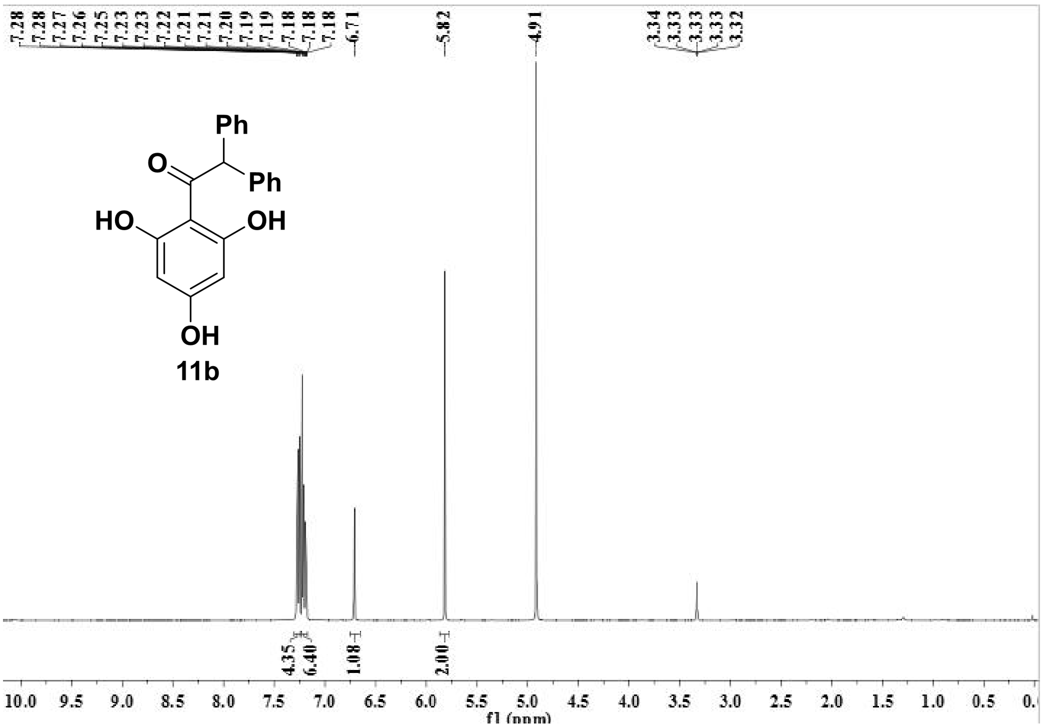
7.28 7.28 7.27 7.26 7.25 7.23 7.23 7.22 7.21 7.21 7.20 7.19 7.19 7.18 7.18 7.18
6.71
5.82
4.91
3.34 3.33 3.33 3.33 3.32
Ph
O
Ph
HO
OH
OH
11b
4.35
6.40
1.08
2.00
10.0 9.5 9.0 8.5 8.0 7.5 7.0 6.5 6.0 5.5 5.0 4.5 4.0 3.5 3.0 2.5 2.0 1.5 1.0 0.5
f1 (ppm)

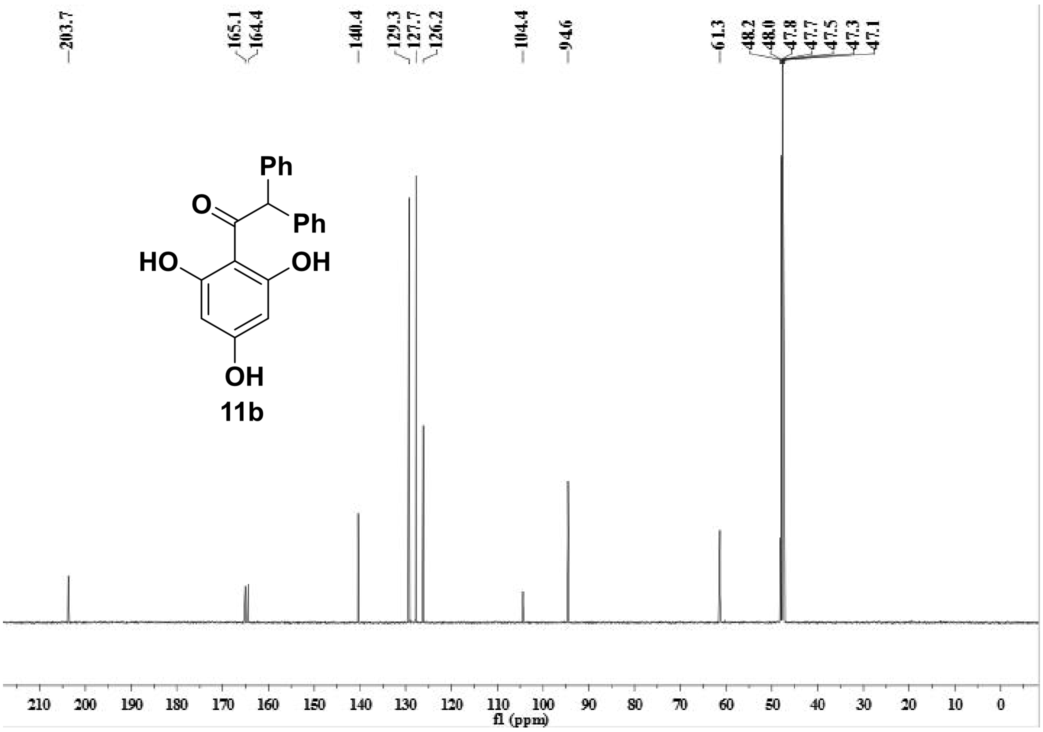
203.7
165.1 164.4
140.4
129.3 127.7 126.2
104.4
94.6
61.3
48.2 48.0 47.8 47.7 47.5 47.3 47.1
Ph
O
Ph
HO
OH
OH
11b
210 200 190 180 170 160 150 140 130 120 110 100 90 80 70 60 50 40 30 20 10 0
f1 (ppm)

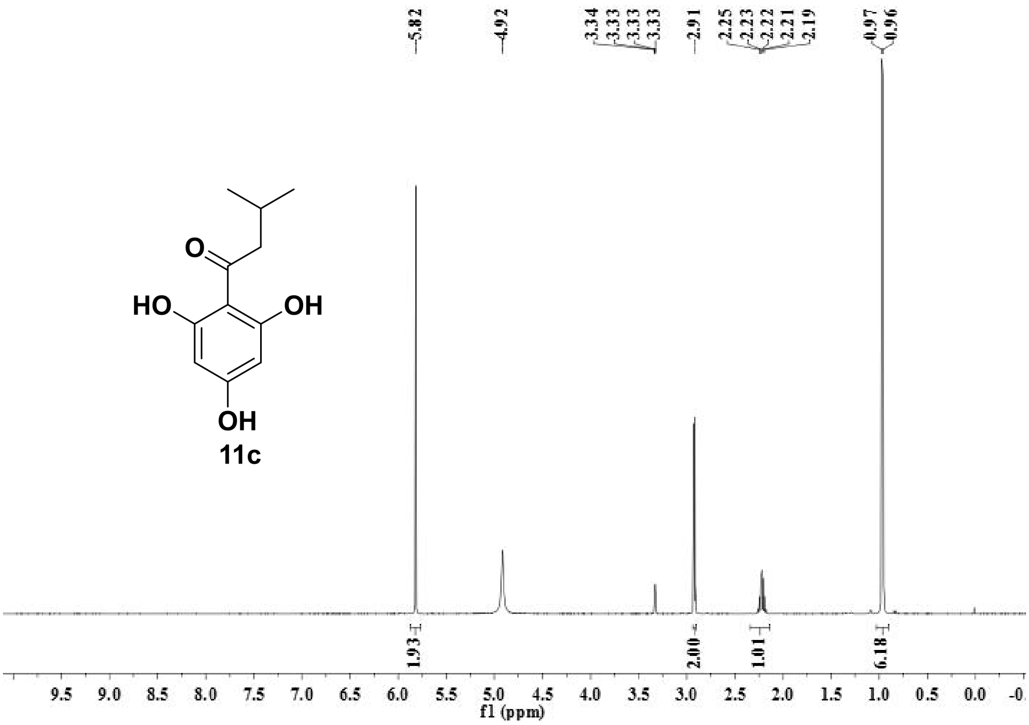

5.82
4.92
3.34
3.33
3.33
3.33
2.91
2.25
2.23
2.22
2.21
2.19
0.97
0.96
1.93
2.00
1.01
6.18
9.5 9.0 8.5 8.0 7.5 7.0 6.5 6.0 5.5 5.0 4.5 4.0 3.5 3.0 2.5 2.0 1.5 1.0 0.5 0.0
f1 (ppm)


**11c**

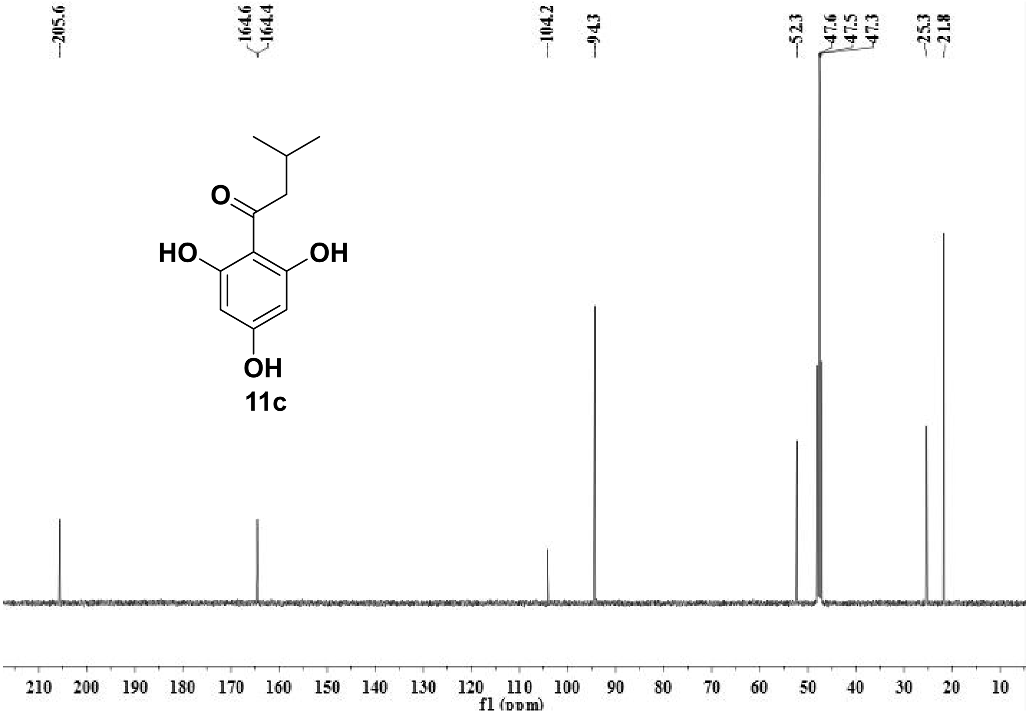

205.6
164.6
164.4
104.2
94.3
52.3
47.6
47.5
47.3
25.3
21.8
210 200 190 180 170 160 150 140 130 120 110 100 90 80 70 60 50 40 30 20 10
f1 (ppm)


**11c**

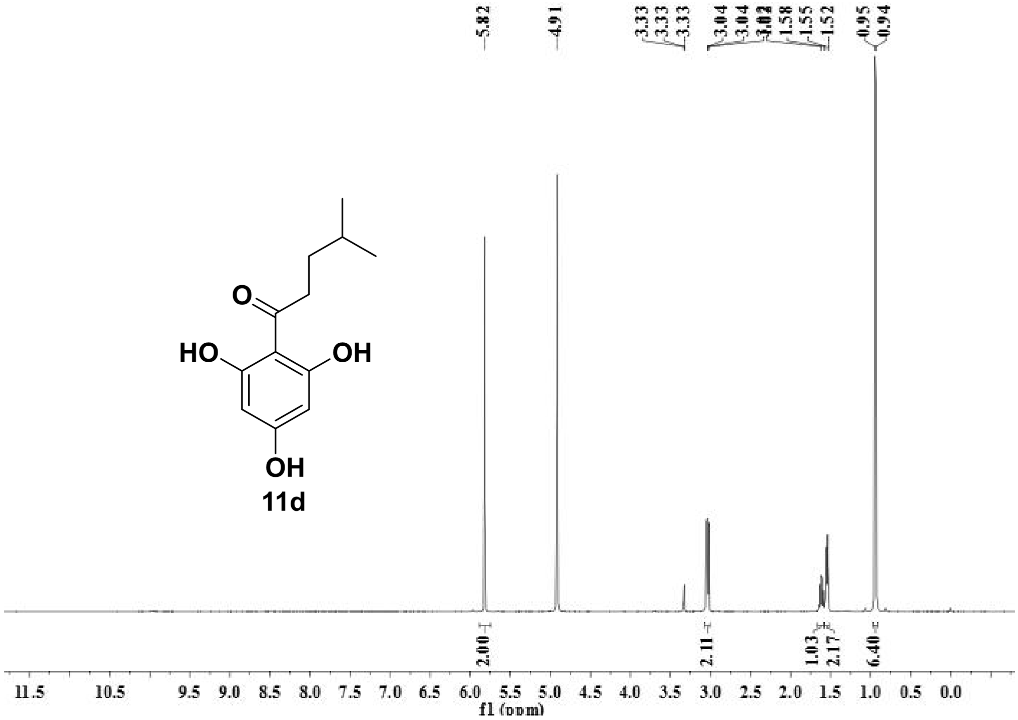
5.82
4.91
3.33
3.33
3.33
3.04
3.04
3.02
1.58
1.55
1.52
0.95
0.94
O
HO
OH
OH
11d
2.00
2.11
1.03
2.17
6.40
11.5 10.5 9.5 9.0 8.5 8.0 7.5 7.0 6.5 6.0 5.5 5.0 4.5 4.0 3.5 3.0 2.5 2.0 1.5 1.0 0.5 0.0
f1 (ppm)

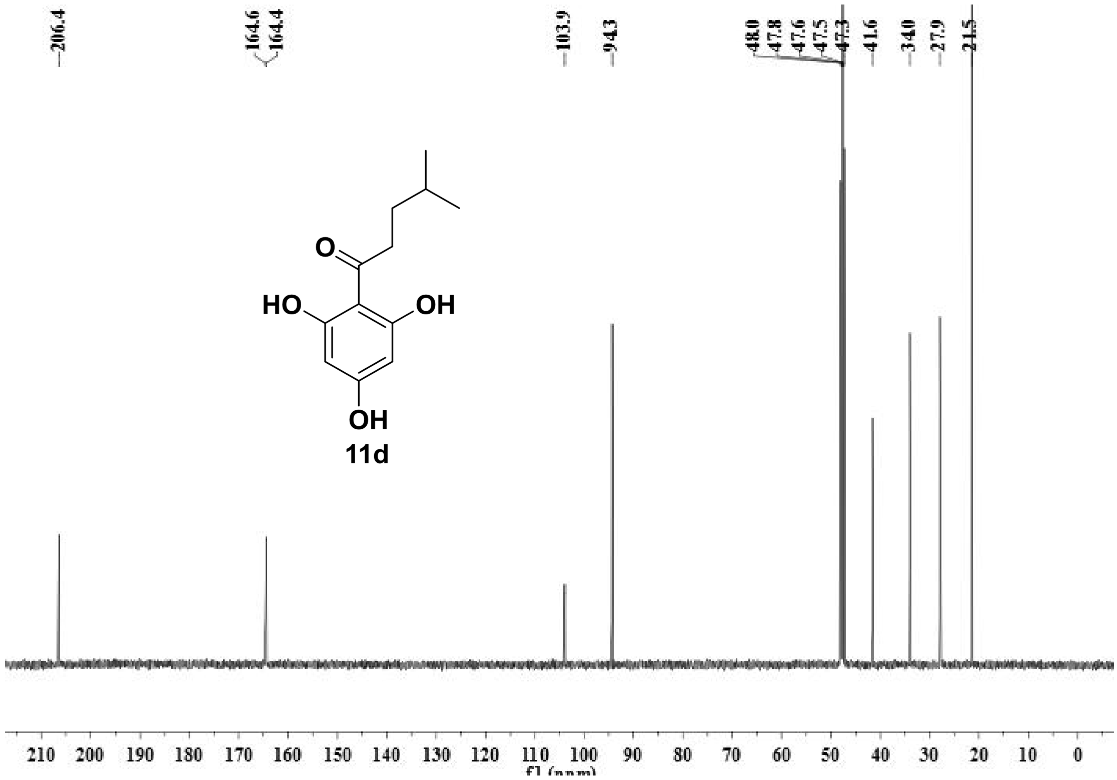
206.4
164.6
164.4
103.9
94.3
48.0
47.8
47.6
47.5
47.3
41.6
34.0
27.9
21.5
O
HO
OH
OH
11d
210 200 190 180 170 160 150 140 130 120 110 100 90 80 70 60 50 40 30 20 10 0
f1 (ppm)

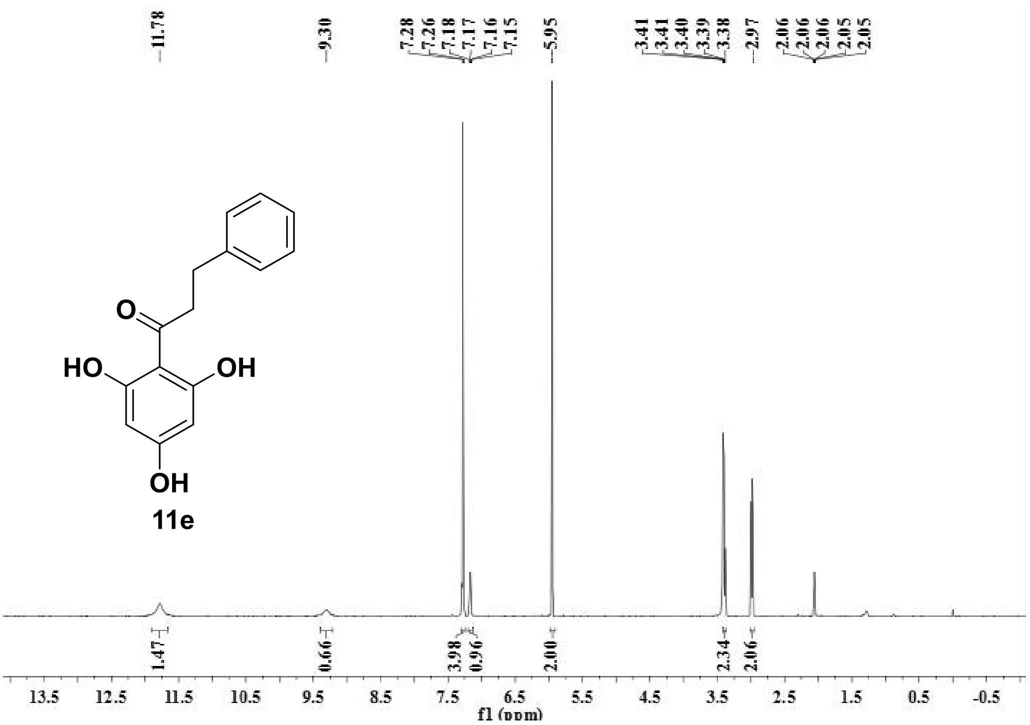

11.78
9.30
7.28 7.26 7.18 7.17 7.16 7.15
5.95
3.41 3.41 3.40 3.39 3.38
2.97
2.06 2.06 2.06 2.05 2.05
O
HO
OH
OH
11e
1.47
0.66
3.98
0.96
2.00
2.34
2.06
13.5 12.5 11.5 10.5 9.5 8.5 7.5 6.5 5.5 4.5 3.5 2.5 1.5 0.5 -0.5
f1 (ppm)


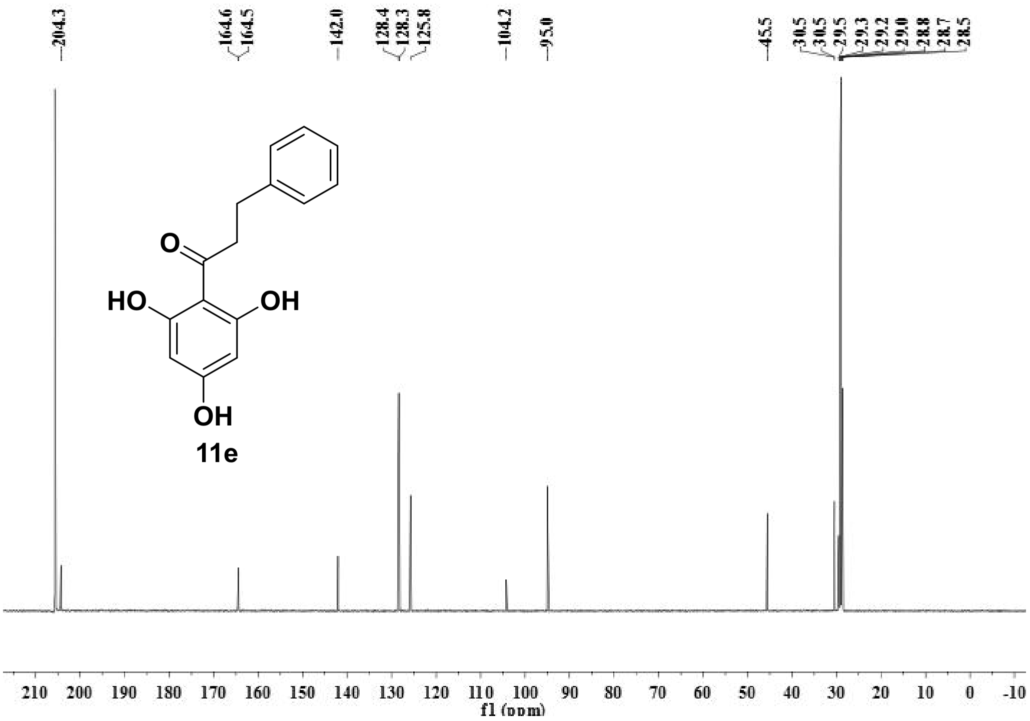

204.3
164.6 164.5
142.0
128.4 128.3 125.8
104.2
95.0
45.5
30.5 30.5 29.5 29.3 29.2 29.0 28.8 28.7 28.5
O
HO
OH
OH
11e
210 200 190 180 170 160 150 140 130 120 110 100 90 80 70 60 50 40 30 20 10 0 -10
f1 (ppm)

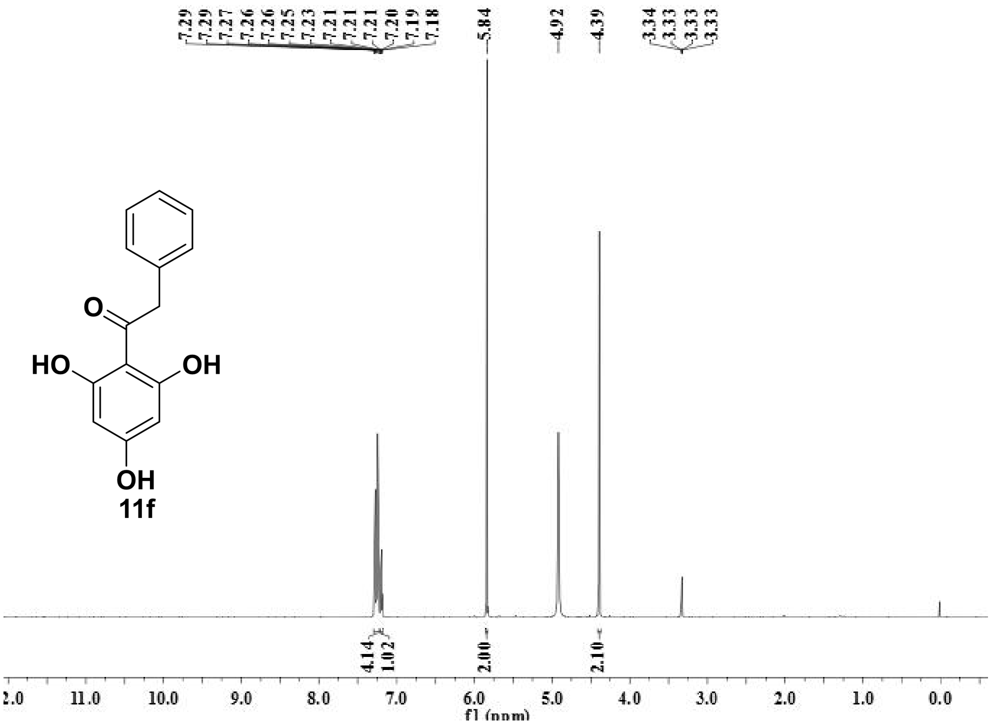
7.29 7.29 7.27 7.26 7.26 7.25 7.23 7.21 7.21 7.21 7.20 7.19 7.18
5.84
4.92
4.39
3.34 3.33 3.33 3.33
HO
OH
O
OH
11f
4.14
1.02
2.00
2.10
12.0 11.0 10.0 9.0 8.0 7.0 6.0 5.0 4.0 3.0 2.0 1.0 0.0
f1 (ppm)

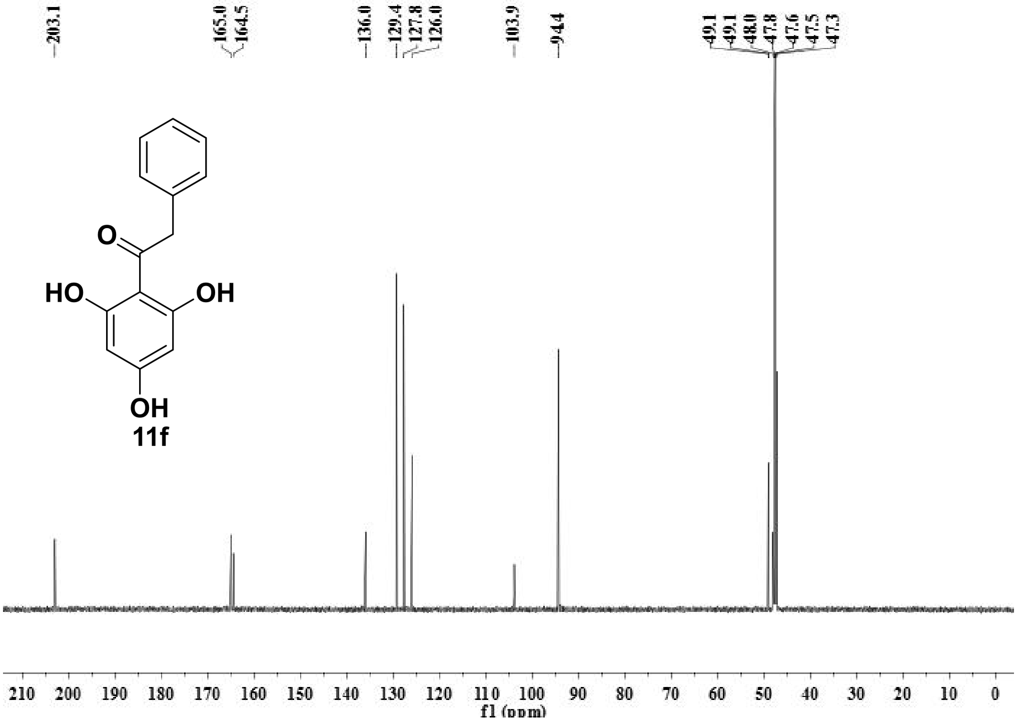
203.1
165.0 164.5
136.0
129.4 127.8 126.0
103.9
94.4
49.1 49.1 48.0 47.8 47.6 47.5 47.3
HO
OH
O
OH
11f
210 200 190 180 170 160 150 140 130 120 110 100 90 80 70 60 50 40 30 20 10 0
f1 (ppm)

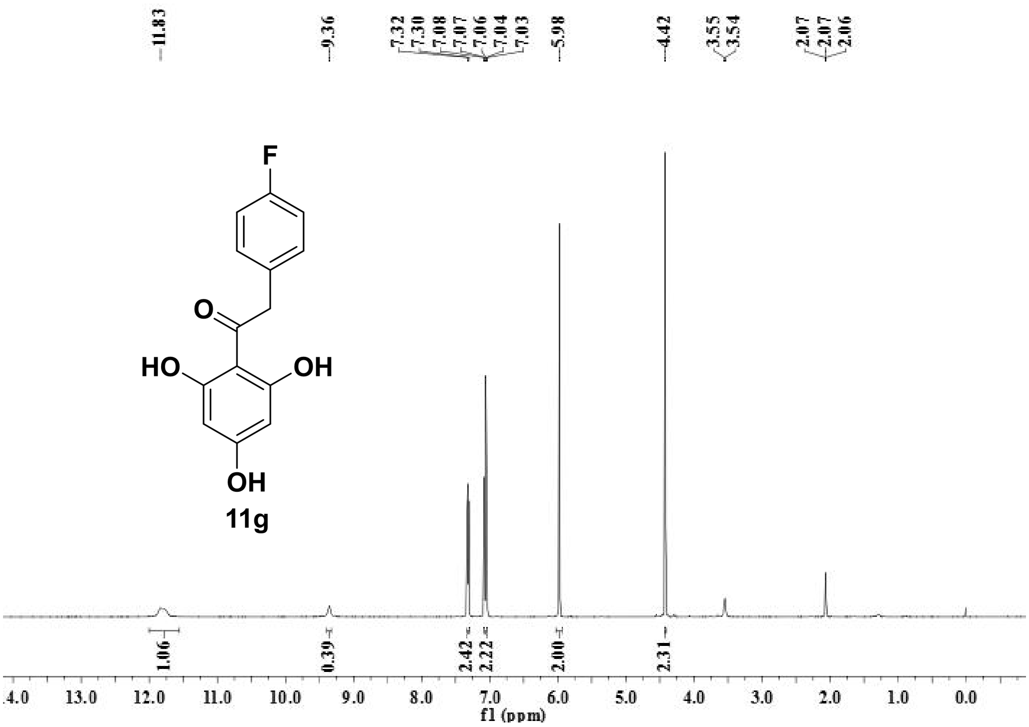
F
O
HO
OH
OH
11g
f1 (ppm)

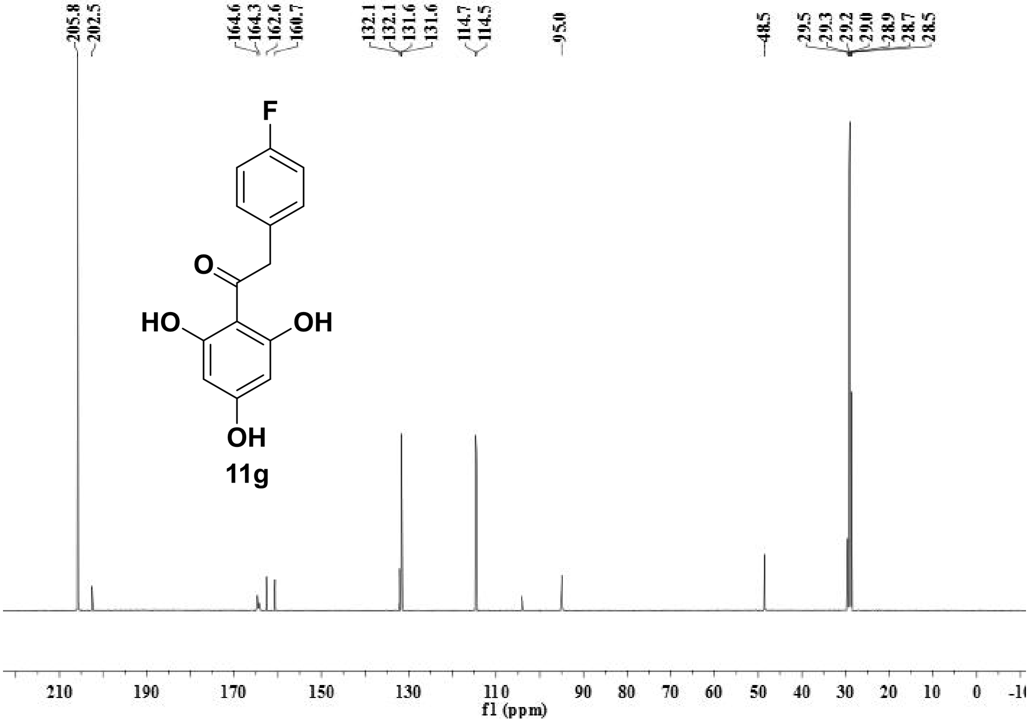
F
O
HO
OH
OH
11g
f1 (ppm)

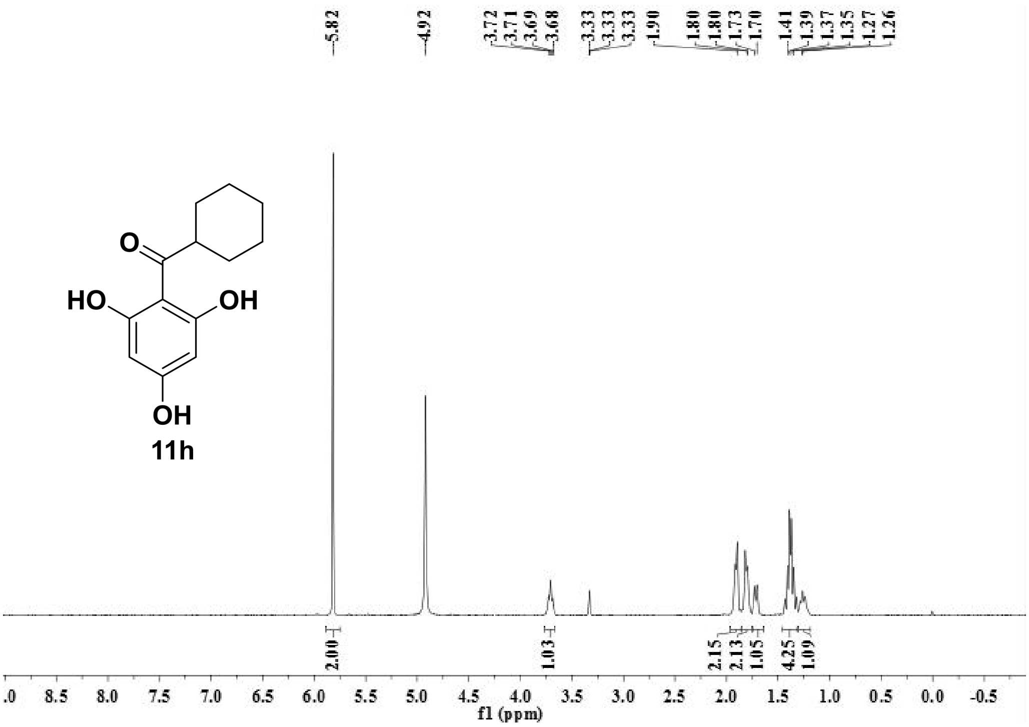

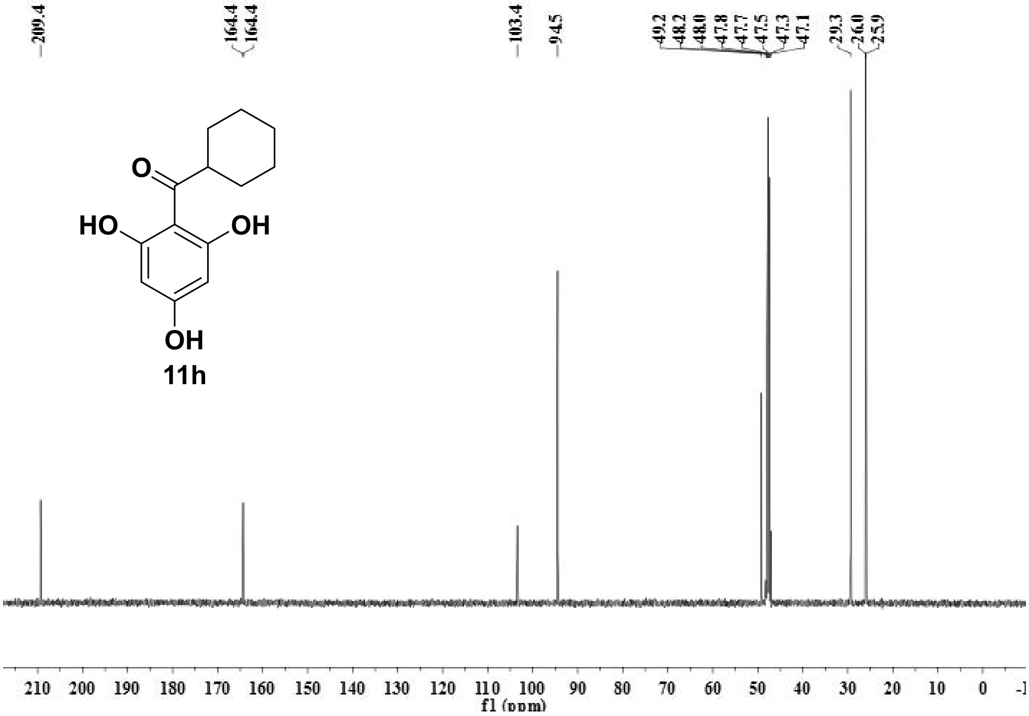

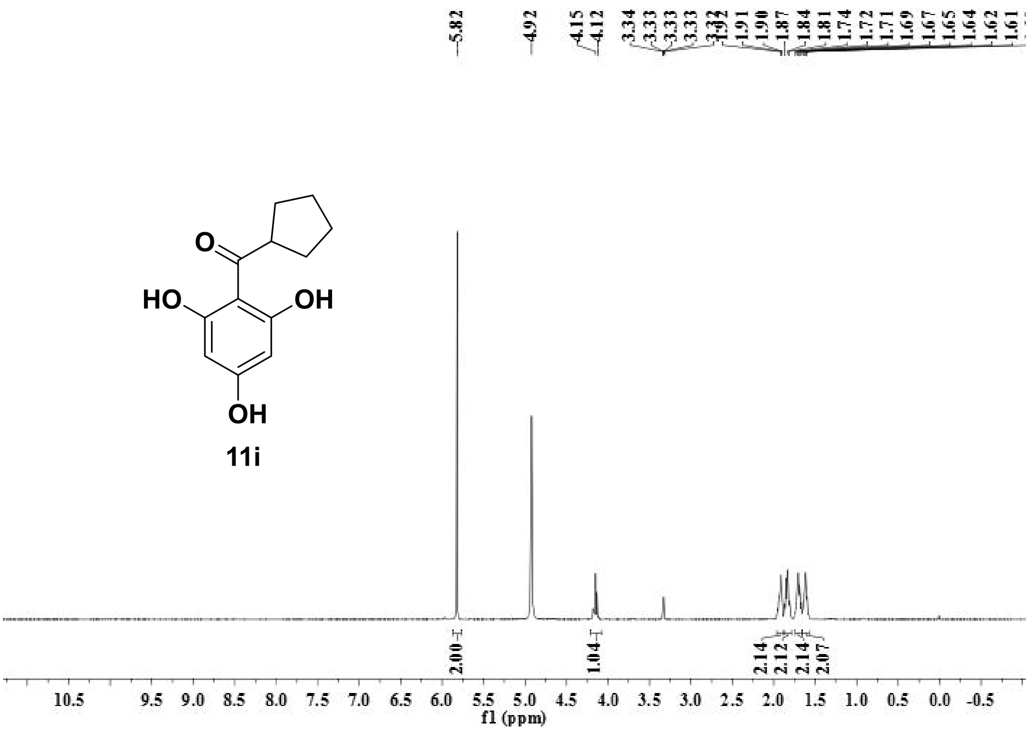

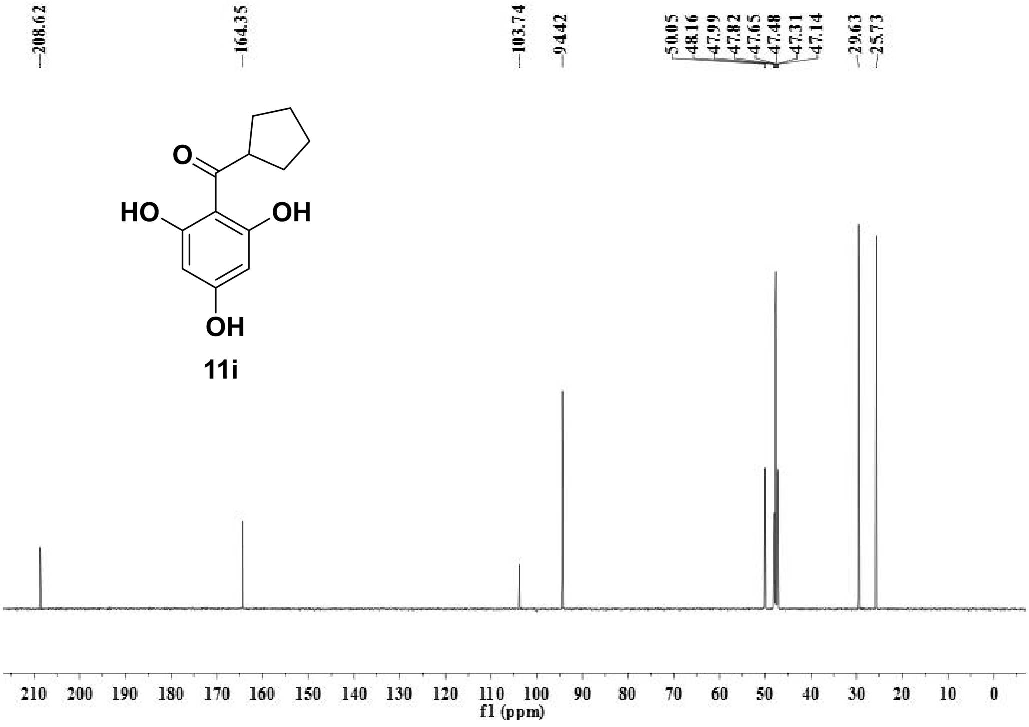

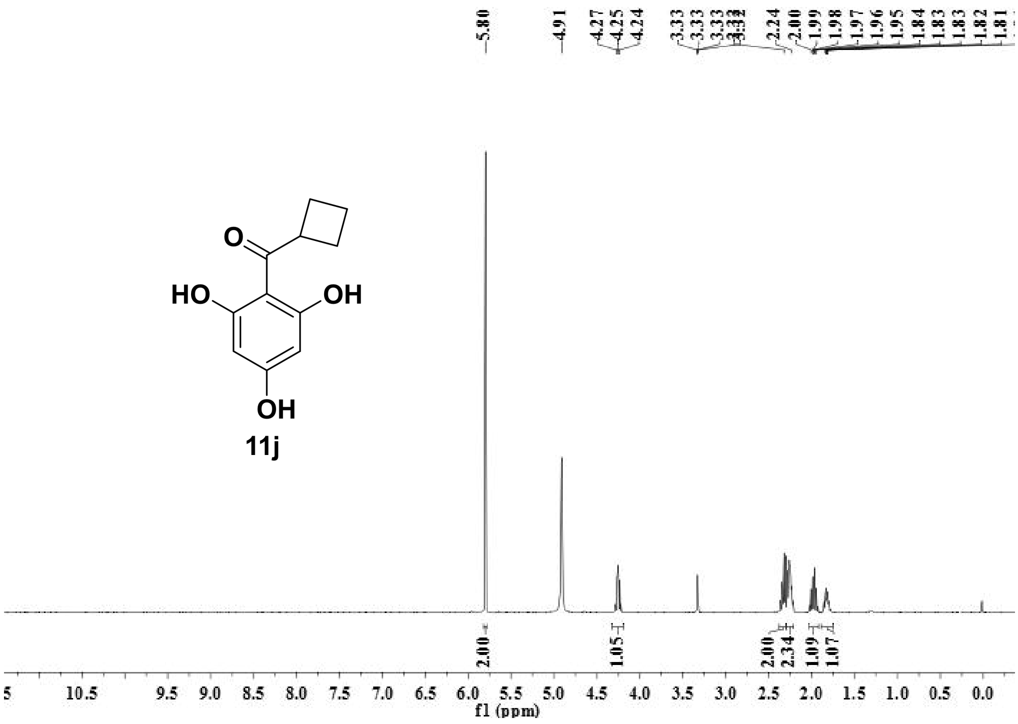

HO
OH
OH
11j
f1 (ppm)


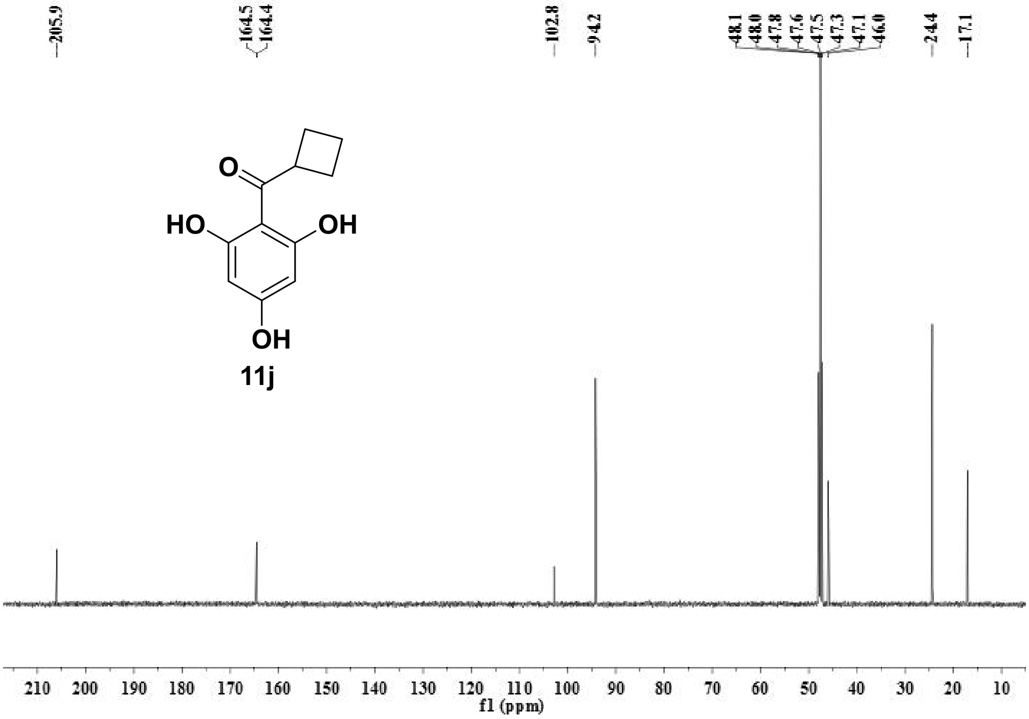

HO
OH
OH
11j
f1 (ppm)

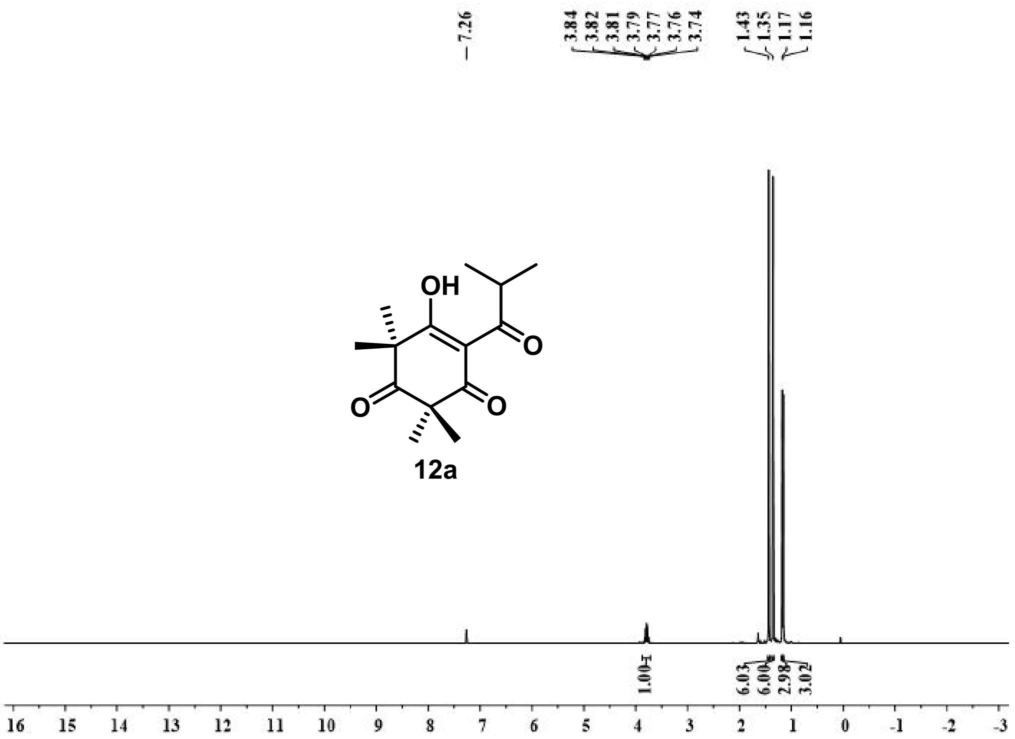
7.26
3.84
3.82
3.81
3.79
3.77
3.76
3.74
1.43
1.35
1.17
1.16
OH
O
O
O
12a
1.00
6.03
6.00
2.98
3.02
16 15 14 13 12 11 10 9 8 7 6 5 4 3 2 1 0 -1 -2 -3

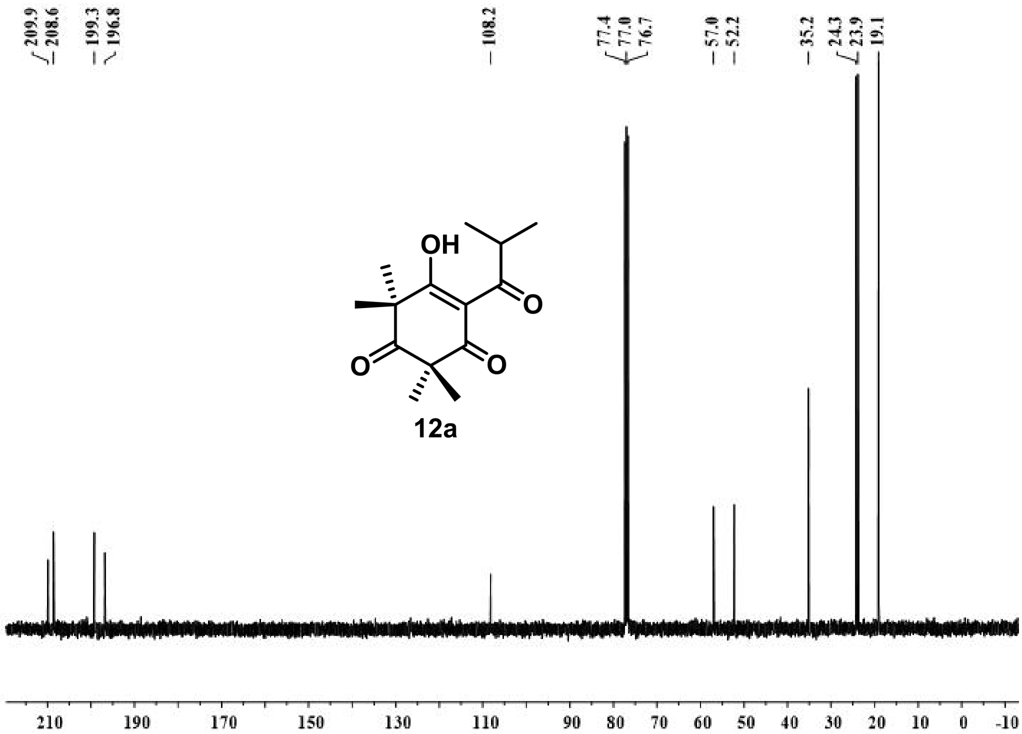
209.9
208.6
199.3
196.8
108.2
77.4
77.0
76.7
57.0
52.2
35.2
24.3
23.9
19.1
OH
O
O
O
12a
210 190 170 150 130 110 90 80 70 60 50 40 30 20 10 0 -10

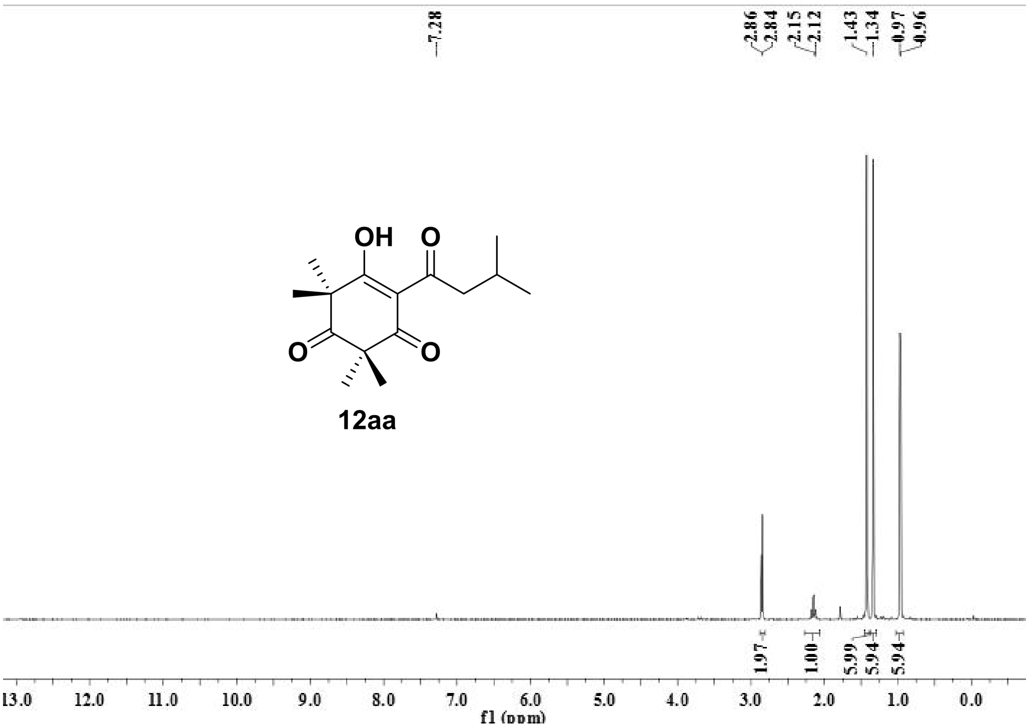

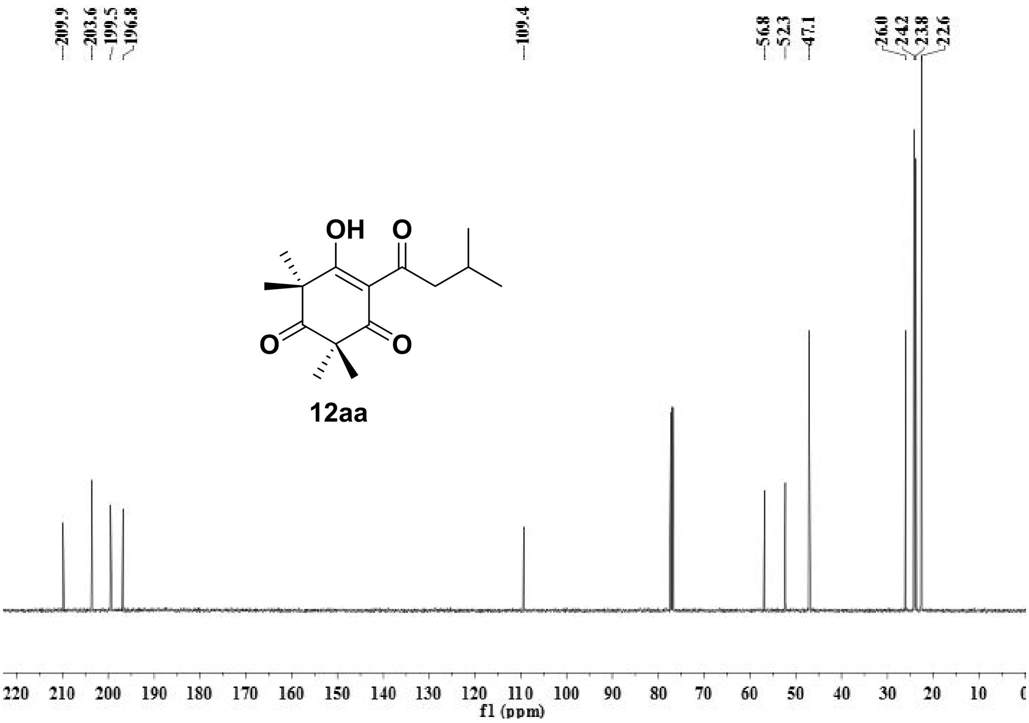

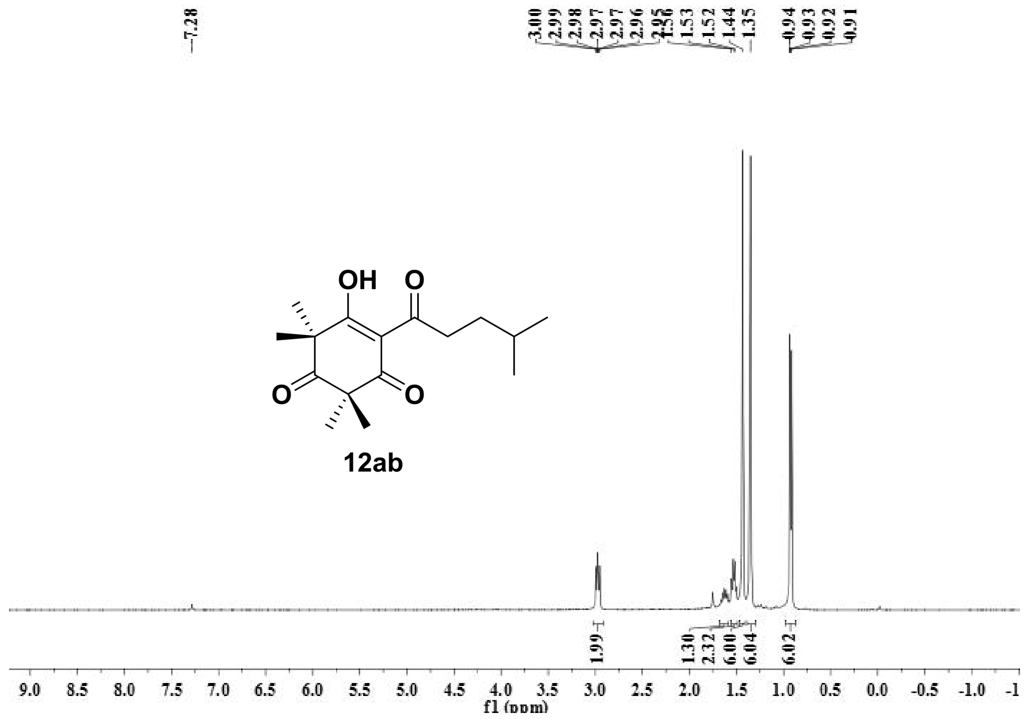
7.28
3.00
2.99
2.98
2.97
2.97
2.96
2.95
1.56
1.53
1.52
1.44
1.35
0.94
0.93
0.92
0.91
OH
O
O
O
12ab
1.99
1.30
2.32
6.00
6.04
6.02
fl (ppm)

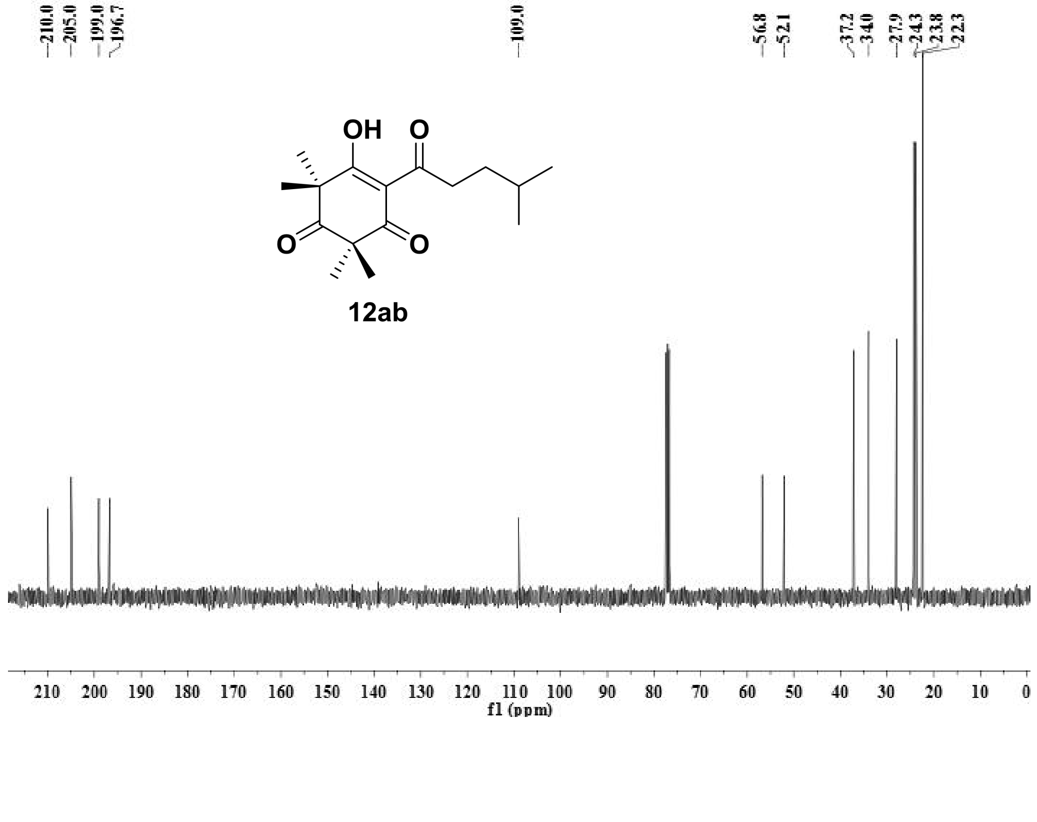
210.0
205.0
199.0
196.7
109.0
56.8
52.1
37.2
34.0
27.9
24.3
23.8
22.3
OH
O
O
O
12ab
fl (ppm)

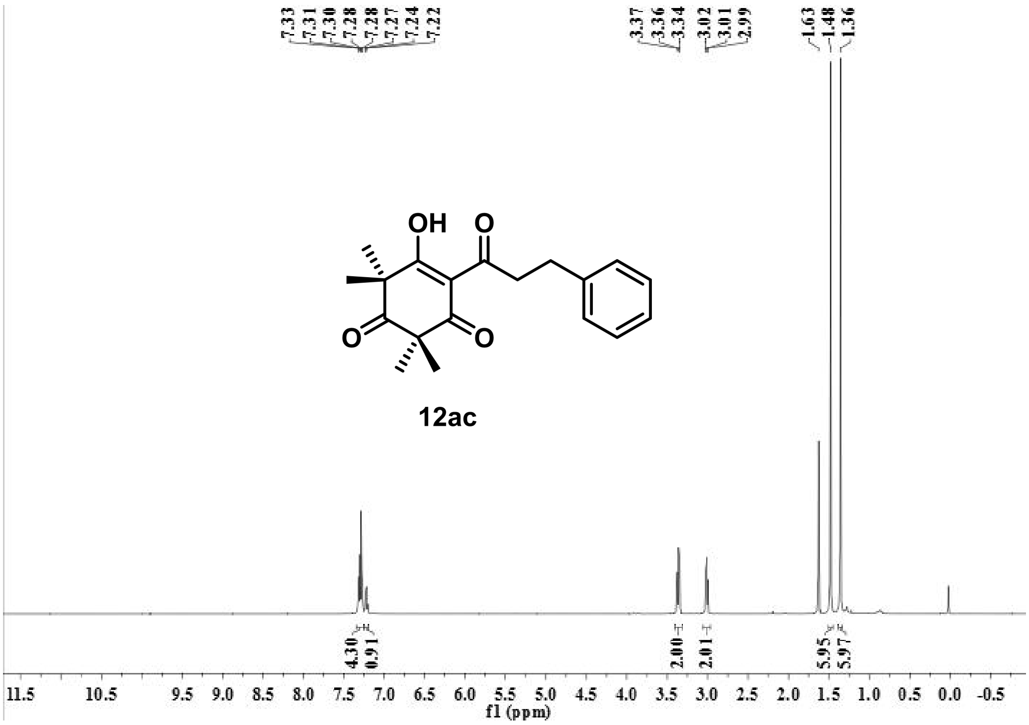
7.33
7.31
7.30
7.28
7.28
7.27
7.24
7.22
3.37
3.36
3.34
3.02
3.01
2.99
1.63
1.48
1.36
OH
O
O
O
12ac
4.30
0.91
2.00
2.01
5.95
5.97
11.5
10.5
9.5
9.0
8.5
8.0
7.5
7.0
6.5
6.0
5.5
5.0
4.5
4.0
3.5
3.0
2.5
2.0
1.5
1.0
0.5
0.0
-0.5
f1 (ppm)

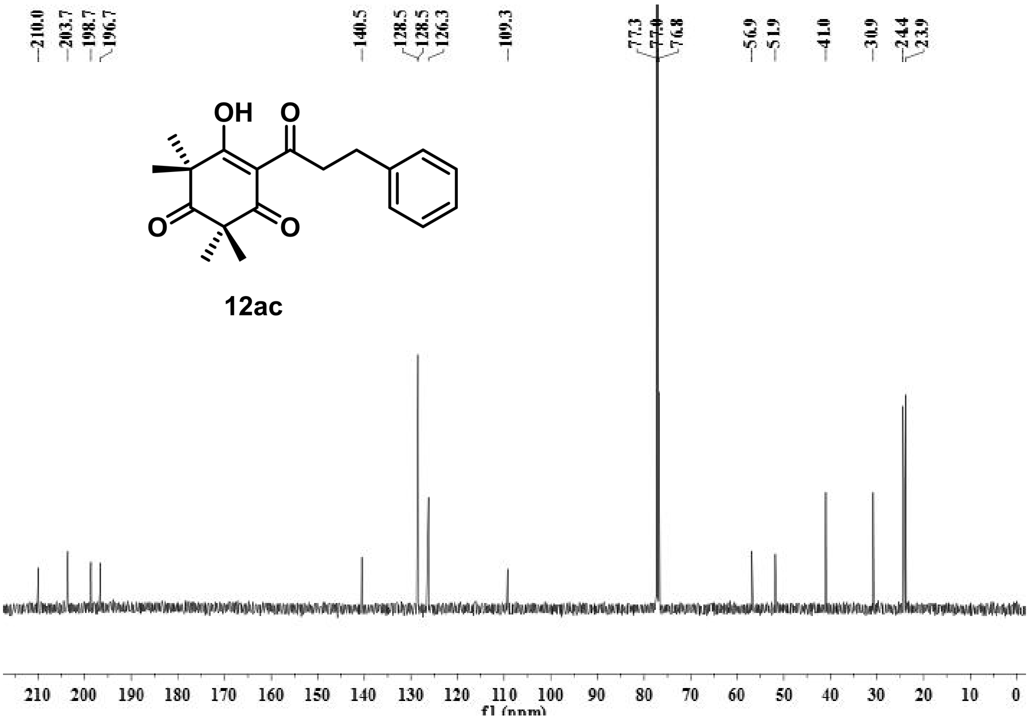
210.0
203.7
198.7
196.7
140.5
128.5
128.5
126.3
109.3
77.3
77.0
76.8
56.9
51.9
41.0
30.9
24.4
23.9
OH
O
O
O
12ac
210
200
190
180
170
160
150
140
130
120
110
100
90
80
70
60
50
40
30
20
10
0

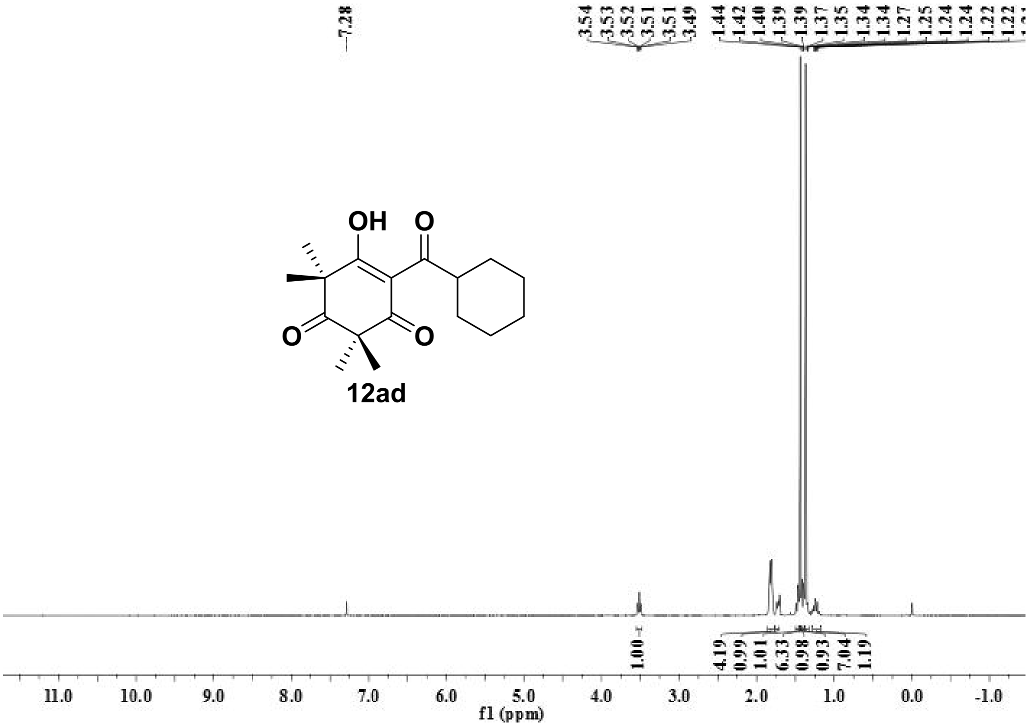
7.28
3.54 3.53 3.52 3.51 3.51 3.49
1.44 1.42 1.40 1.39 1.39 1.37 1.35 1.34 1.34 1.27 1.25 1.24 1.24 1.22 1.22
OH
O
O
O
12ad
1.00
4.19 0.99 1.01 6.33 0.98 0.93 7.04 1.19
11.0 10.0 9.0 8.0 7.0 6.0 5.0 4.0 3.0 2.0 1.0 0.0 -1.0
f1 (ppm)

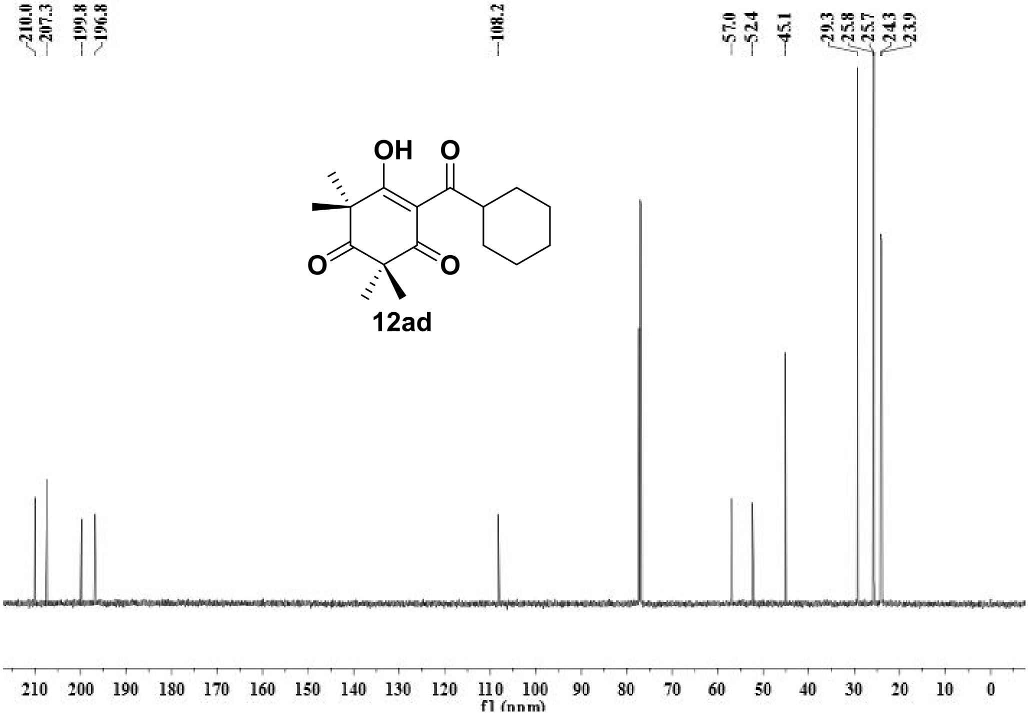
210.0 207.3 199.8 196.8
108.2
57.0 52.4 45.1
29.3 25.8 25.7 24.3 23.9
OH
O
O
O
12ad
210 200 190 180 170 160 150 140 130 120 110 100 90 80 70 60 50 40 30 20 10 0
f1 (ppm)

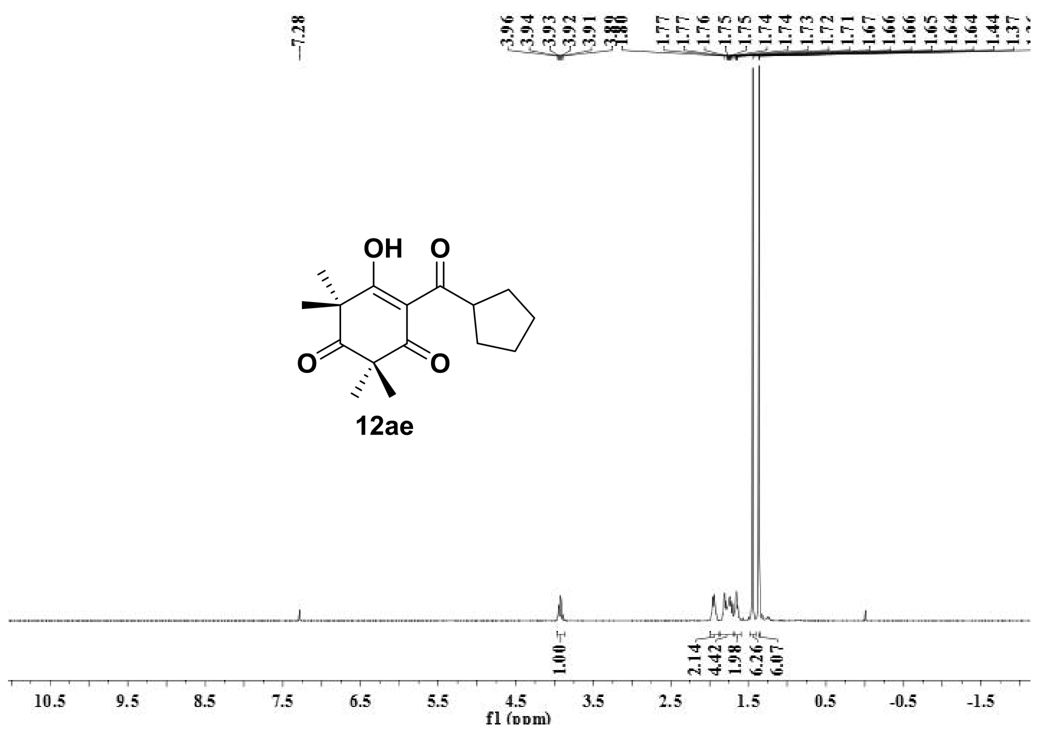

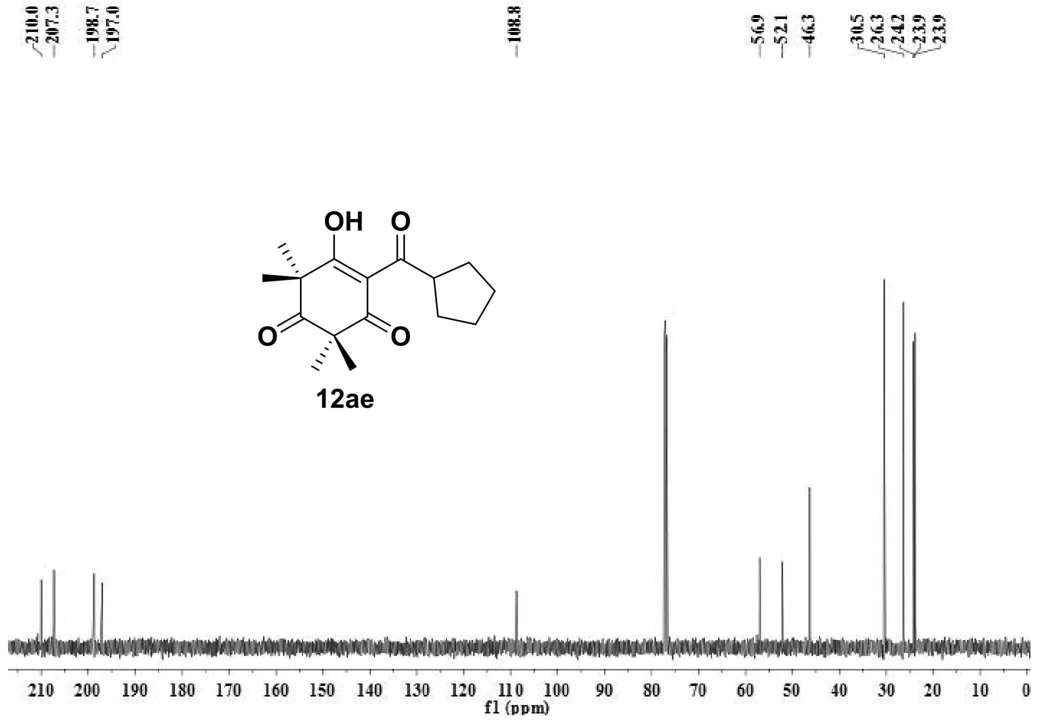

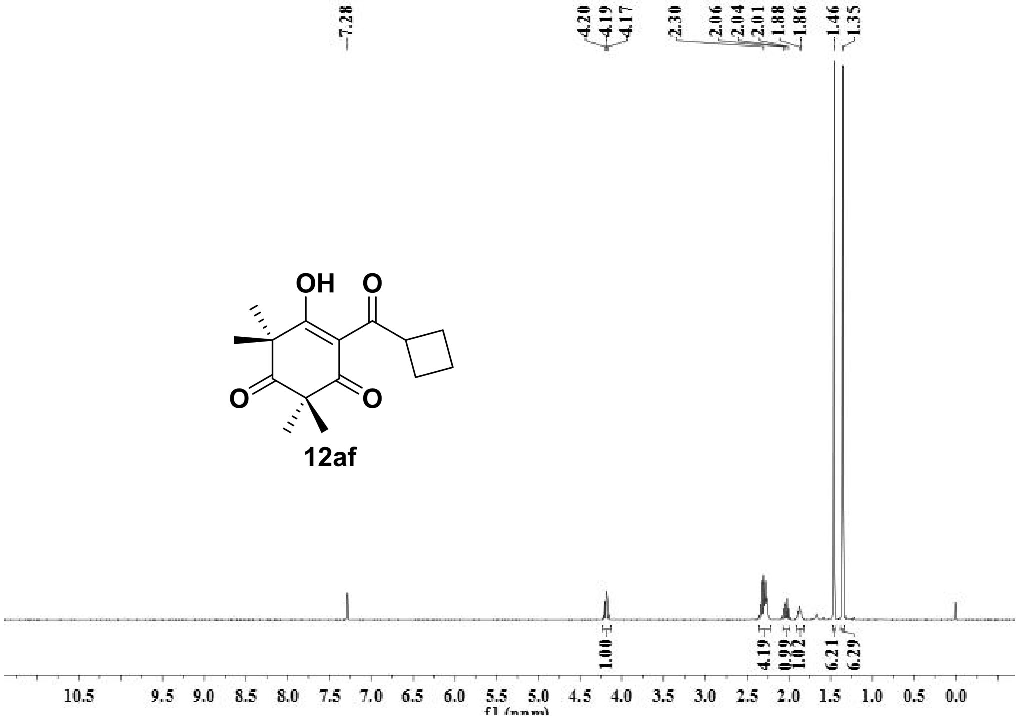
7.28
4.20
4.19
4.17
2.30
2.06
2.04
2.01
1.88
1.86
1.46
1.35
OH
O
O
O
12af
1.00
4.19
0.99
1.02
6.21
6.29
10.5 9.5 9.0 8.5 8.0 7.5 7.0 6.5 6.0 5.5 5.0 4.5 4.0 3.5 3.0 2.5 2.0 1.5 1.0 0.5 0.0
f1 (ppm)

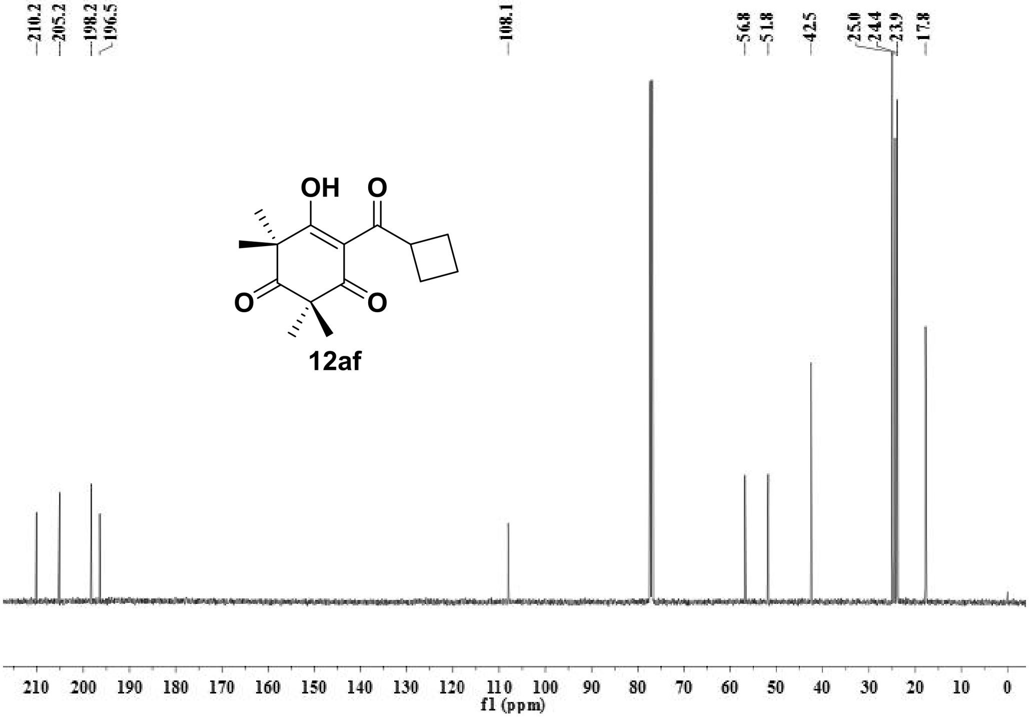
210.2
205.2
198.2
196.5
108.1
56.8
51.8
42.5
25.0
24.4
23.9
17.8
OH
O
O
O
12af
210 200 190 180 170 160 150 140 130 120 110 100 90 80 70 60 50 40 30 20 10 0
f1 (ppm)

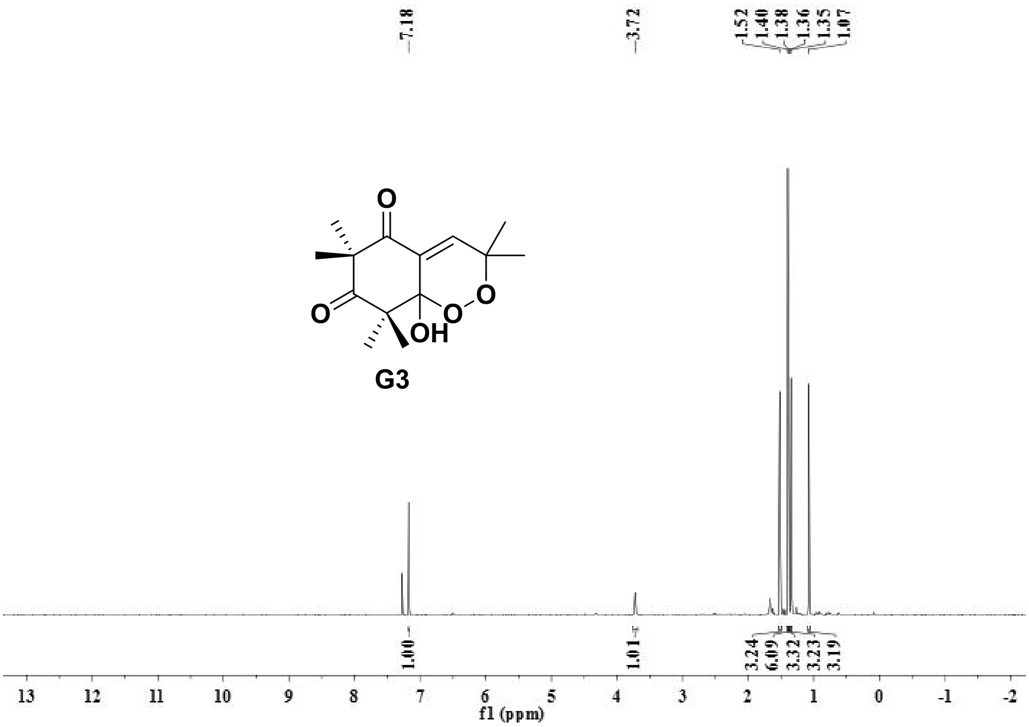

7.18
3.72
1.52
1.40
1.38
1.36
1.35
1.07
O
O
O
O
OH
G3
1.00
1.01
3.24
6.09
3.32
3.23
3.19
13
12
11
10
9
8
7
6
5
4
3
2
1
0
-1
-2
f1 (ppm)


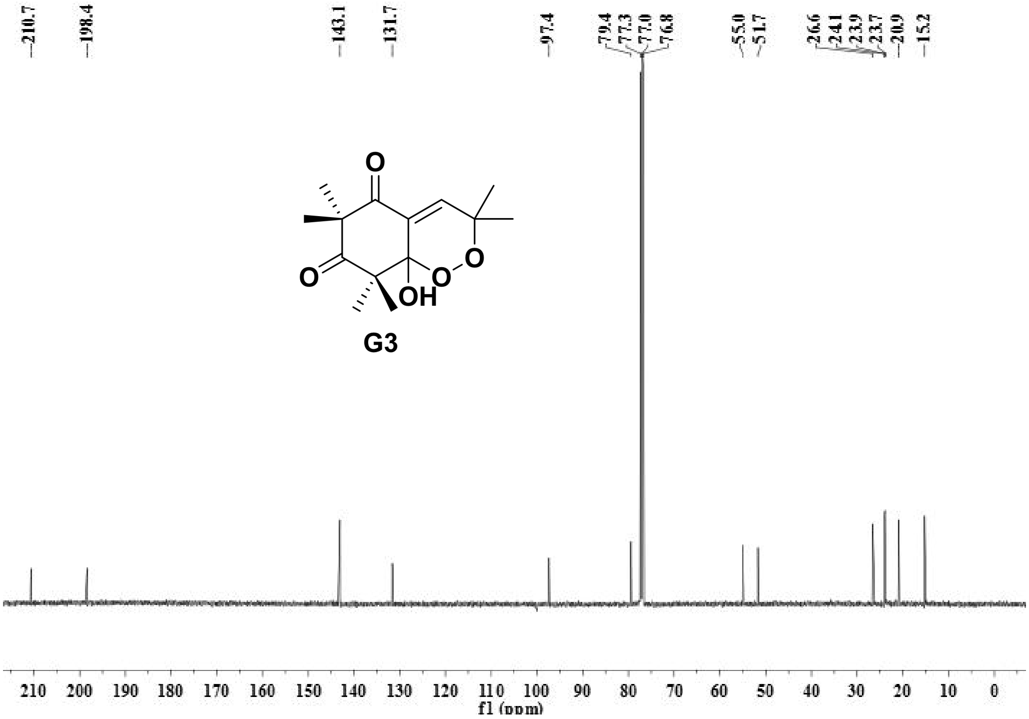

210.7
198.4
143.1
131.7
97.4
79.4
77.3
77.0
76.8
55.0
51.7
26.6
24.1
23.9
23.7
20.9
15.2
O
O
O
O
OH
G3
210
200
190
180
170
160
150
140
130
120
110
100
90
80
70
60
50
40
30
20
10
0
f1 (ppm)

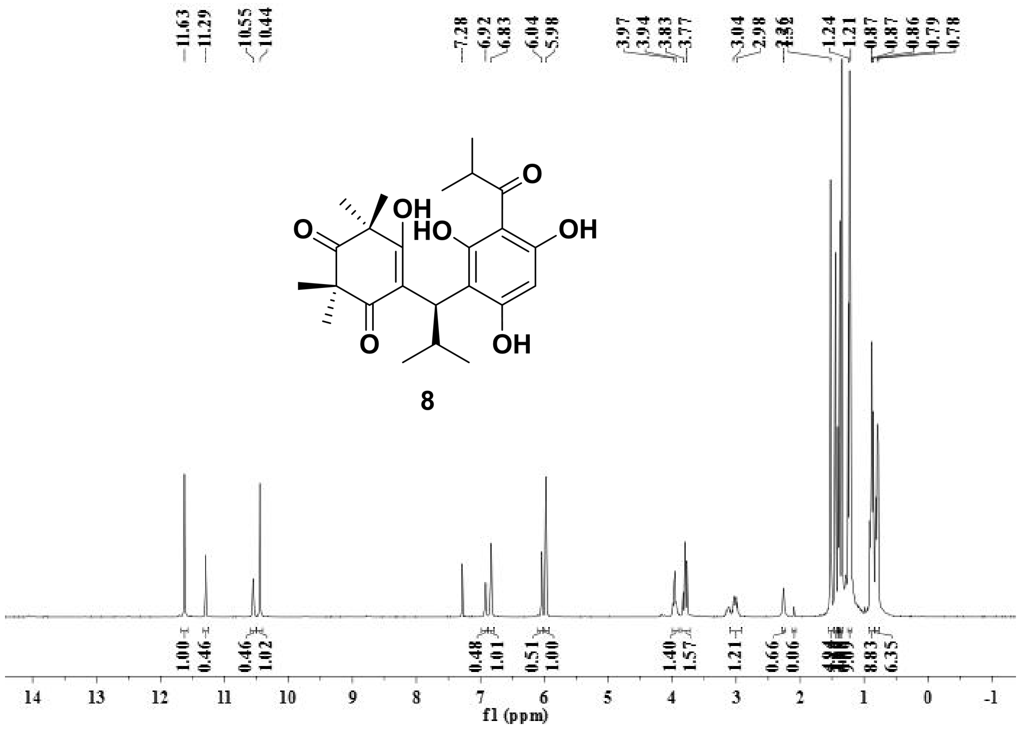

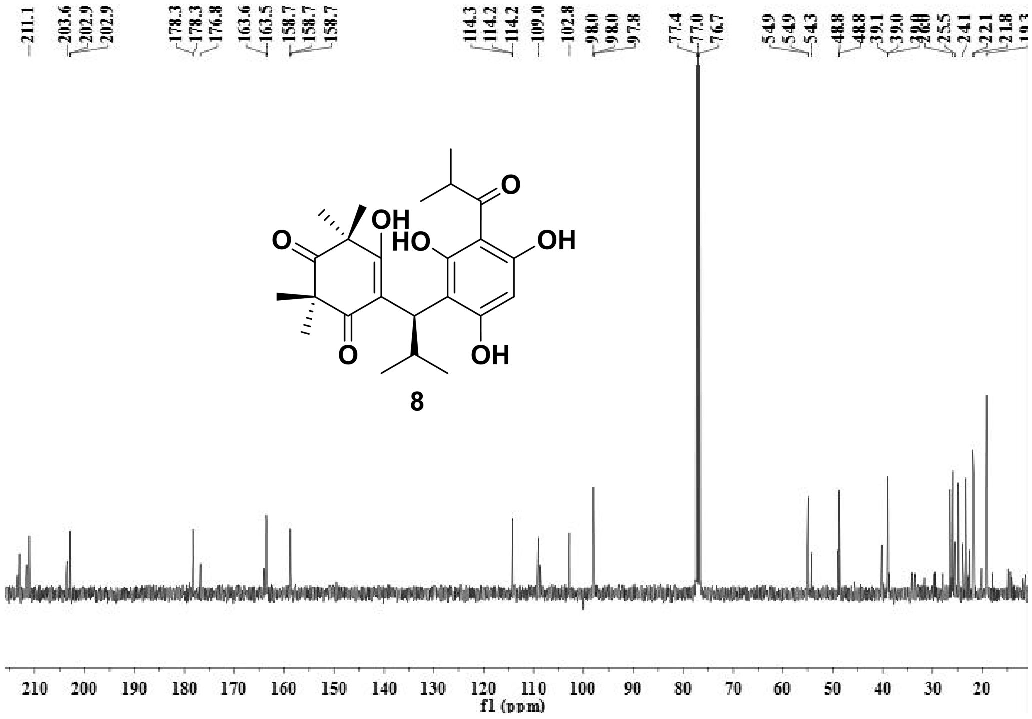

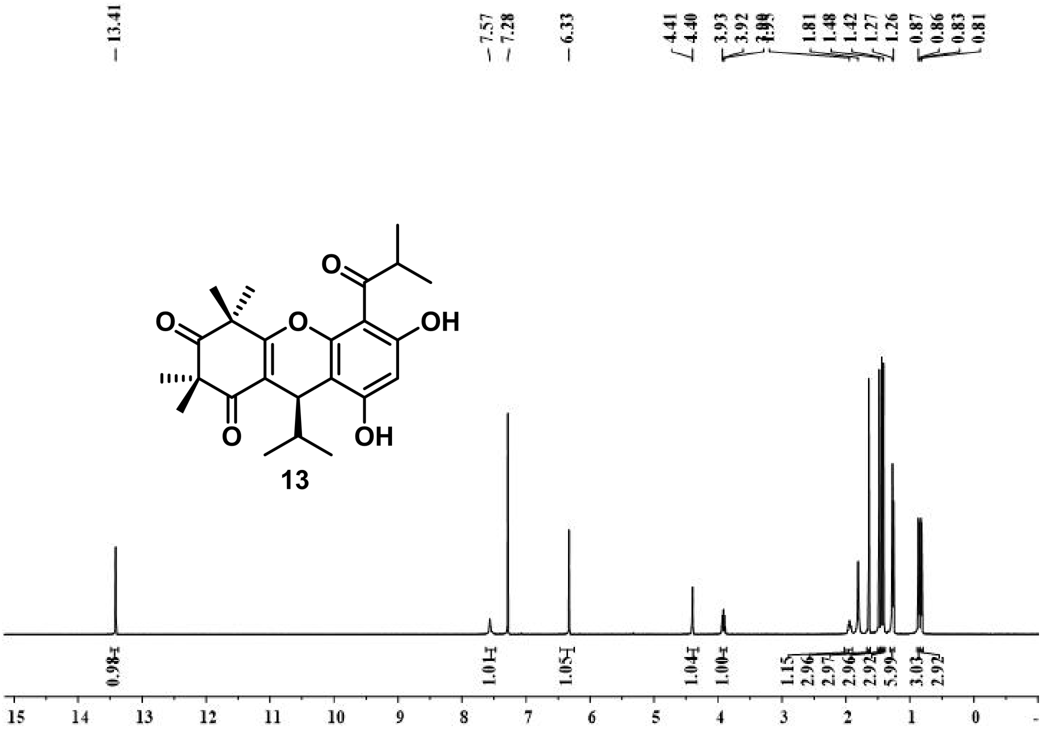
13.41
7.57
7.28
6.33
4.41
4.40
3.93
3.92
1.81
1.48
1.42
1.27
1.26
0.87
0.86
0.83
0.81
OH
OH
O
O
O
O
13
0.98
1.01
1.05
1.04
1.00
1.15
2.96
2.97
2.96
2.92
5.99
3.03
2.92
15 14 13 12 11 10 9 8 7 6 5 4 3 2 1 0

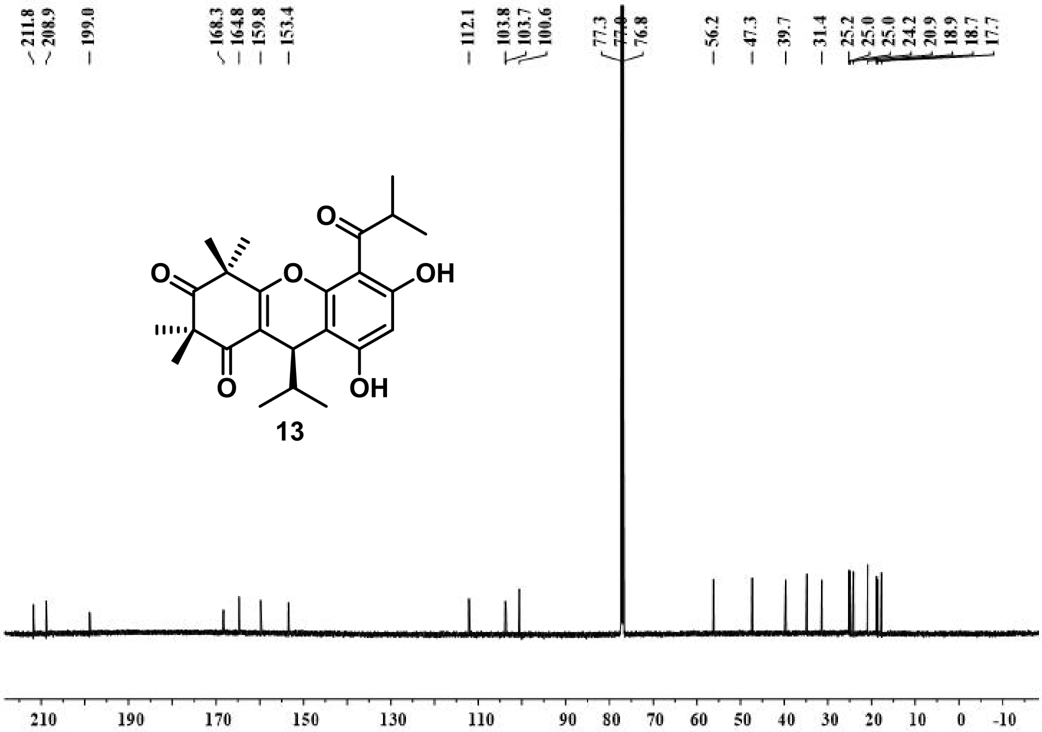
211.8
208.9
199.0
168.3
164.8
159.8
153.4
112.1
103.8
103.7
100.6
77.3
76.8
56.2
47.3
39.7
31.4
25.2
25.0
25.0
24.2
20.9
18.9
18.7
17.7
OH
OH
O
O
O
O
13
210 190 170 150 130 110 90 80 70 60 50 40 30 20 10 0 -10

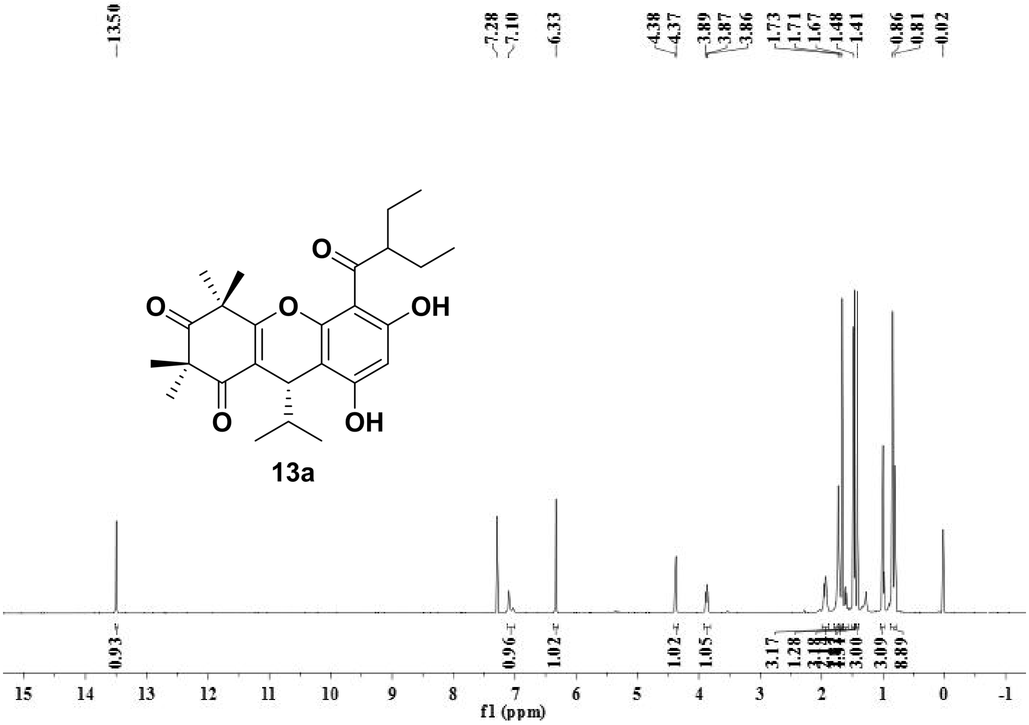
13a
f1 (ppm)

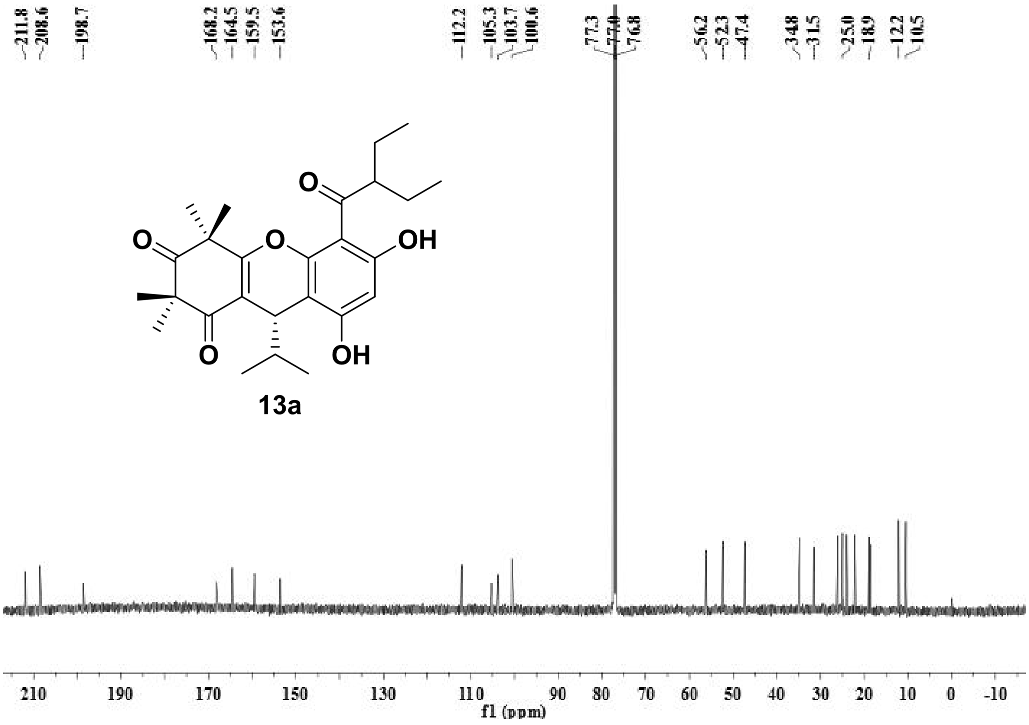
13a
f1 (ppm)

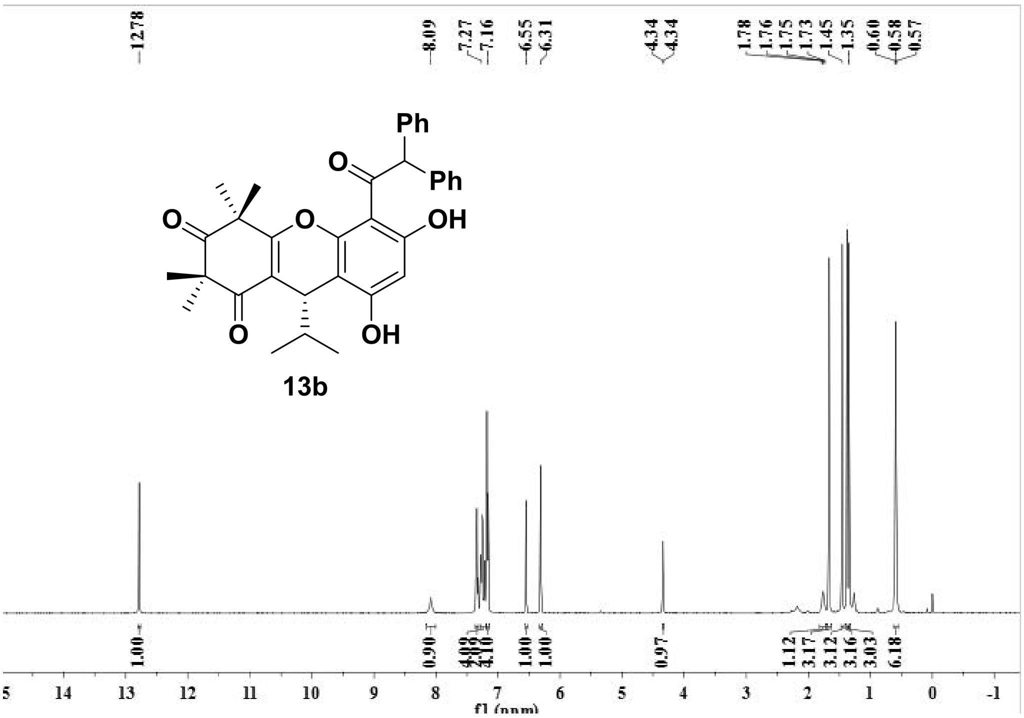
12.78
8.09
7.27
7.16
6.55
6.31
4.34
4.34
1.78
1.76
1.75
1.73
1.45
1.35
0.60
0.58
0.57
Ph
O
Ph
O
O
OH
O
OH
13b
1.00
0.90
4.10
1.00
1.00
0.97
1.12
3.17
3.12
3.16
3.03
6.18
f1 (ppm)

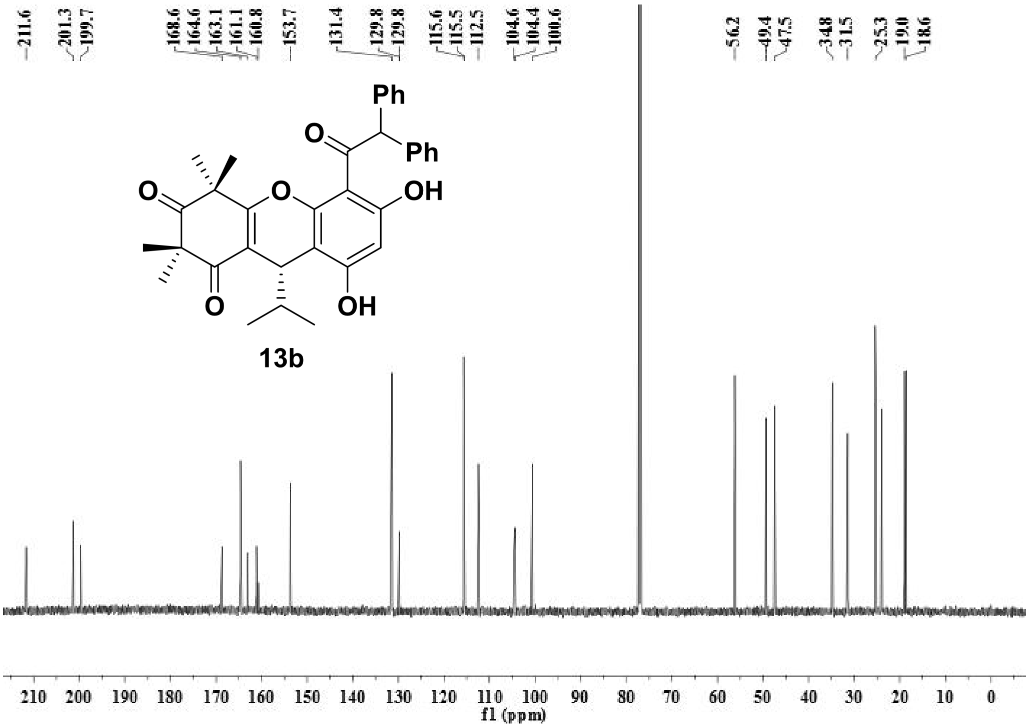
211.6
201.3
199.7
168.6
164.6
163.1
161.1
160.8
153.7
131.4
129.8
129.8
115.6
115.5
112.5
104.6
104.4
100.6
56.2
49.4
47.5
34.8
31.5
25.3
19.0
18.6
Ph
O
Ph
O
O
OH
O
OH
13b
f1 (ppm)

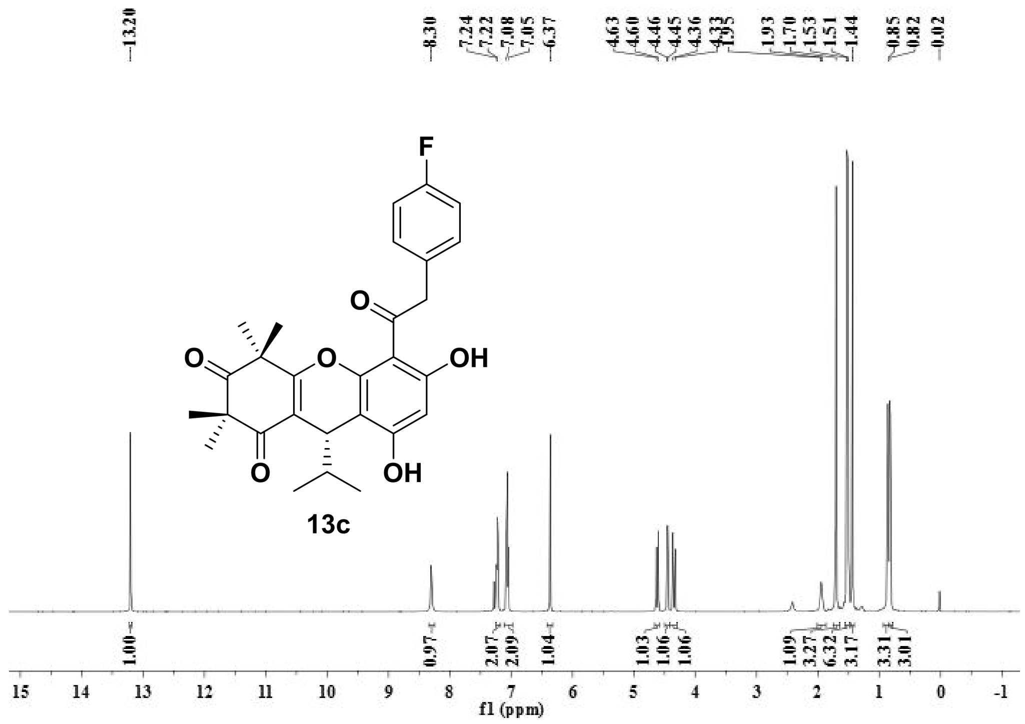
13.20
8.30
7.24
7.22
7.08
7.05
6.37
4.63
4.60
4.46
4.45
4.36
4.33
1.95
1.93
1.70
1.53
1.51
1.44
0.85
0.82
0.02
F
O
OH
13c
1.00
0.97
2.07
2.09
1.04
1.03
1.06
1.06
1.09
3.27
6.32
3.17
3.31
3.01
f1 (ppm)

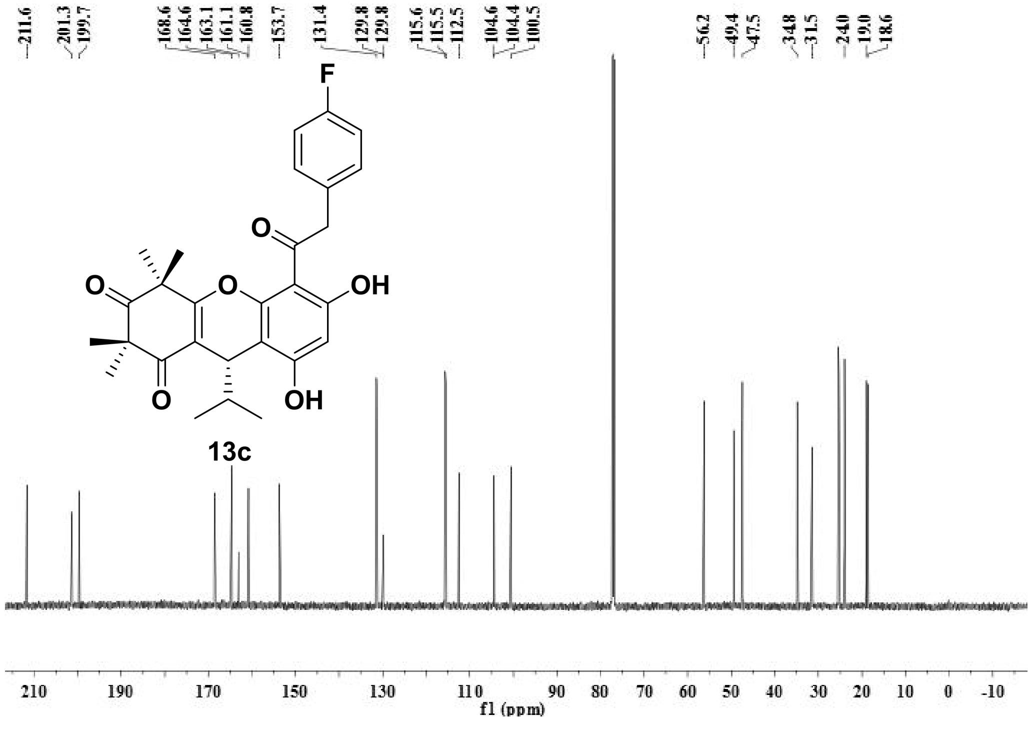
211.6
201.3
199.7
168.6
164.6
163.1
161.1
160.8
153.7
131.4
129.8
129.8
115.6
115.5
112.5
104.6
104.4
100.5
56.2
49.4
47.5
34.8
31.5
24.0
19.0
18.6
F
O
OH
13c
f1 (ppm)

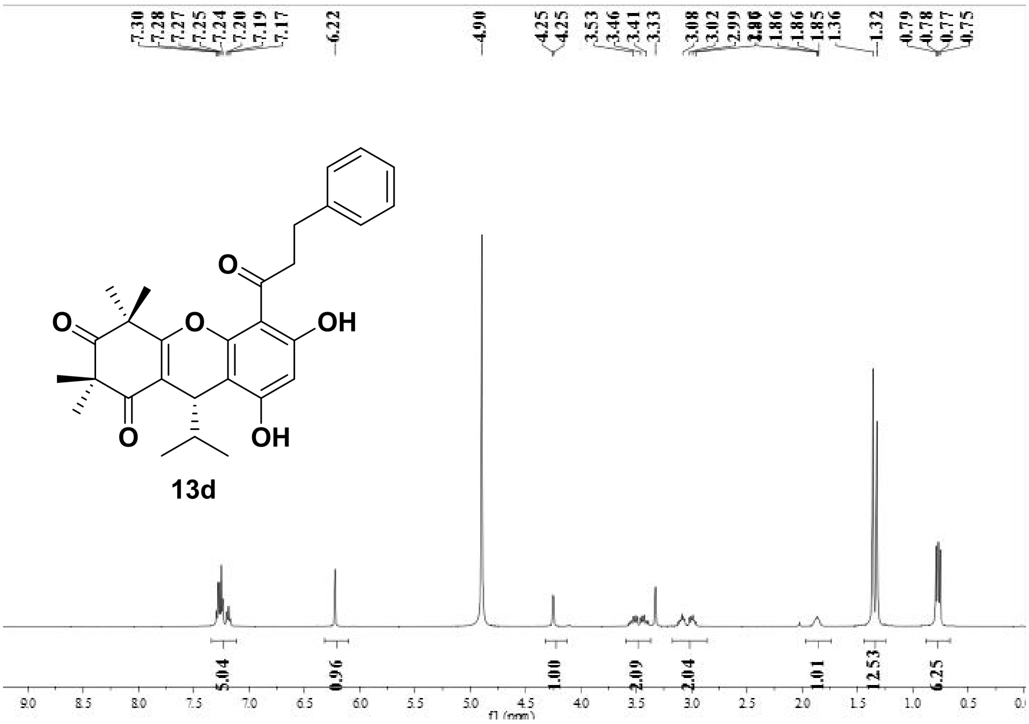
7.30
7.28
7.27
7.25
7.24
7.20
7.19
7.17
6.22
4.90
4.25
4.25
3.53
3.46
3.41
3.33
3.08
3.02
2.99
1.86
1.86
1.85
1.36
1.32
0.79
0.78
0.77
0.75
O
O
O
OH
OH
O
13d
5.04
0.96
1.00
2.09
2.04
1.01
12.53
6.25
f1 (ppm)

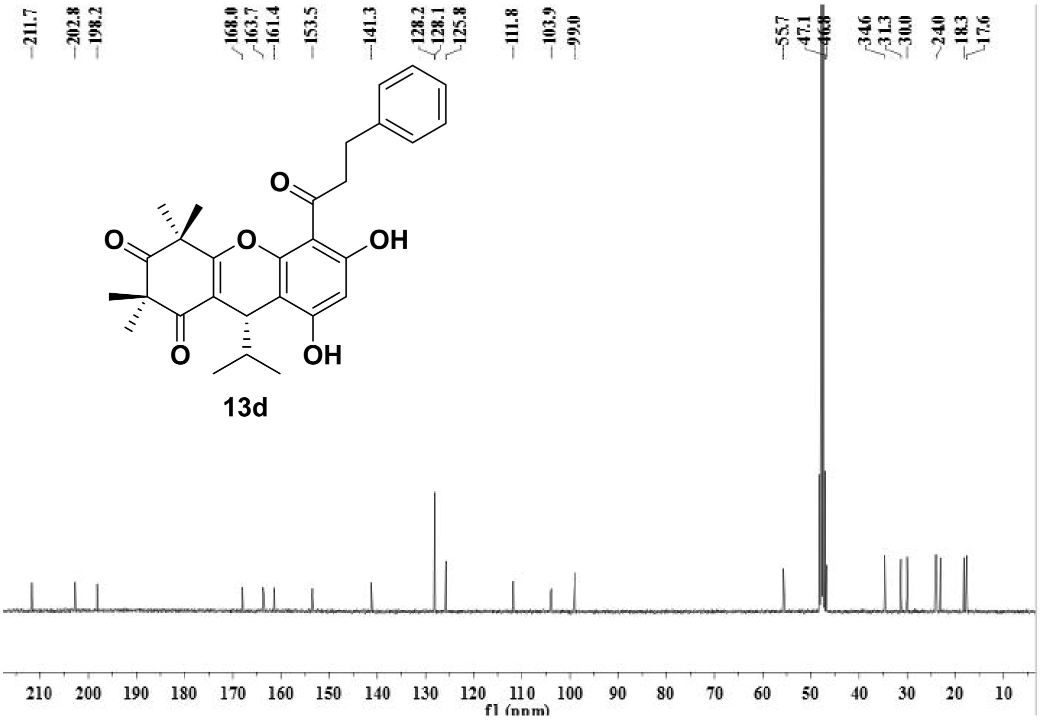
211.7
202.8
198.2
168.0
163.7
161.4
153.5
141.3
128.2
128.1
125.8
111.8
103.9
99.0
55.7
47.1
34.6
31.3
30.0
24.0
18.3
17.6
O
O
O
OH
OH
O
13d
f1 (ppm)

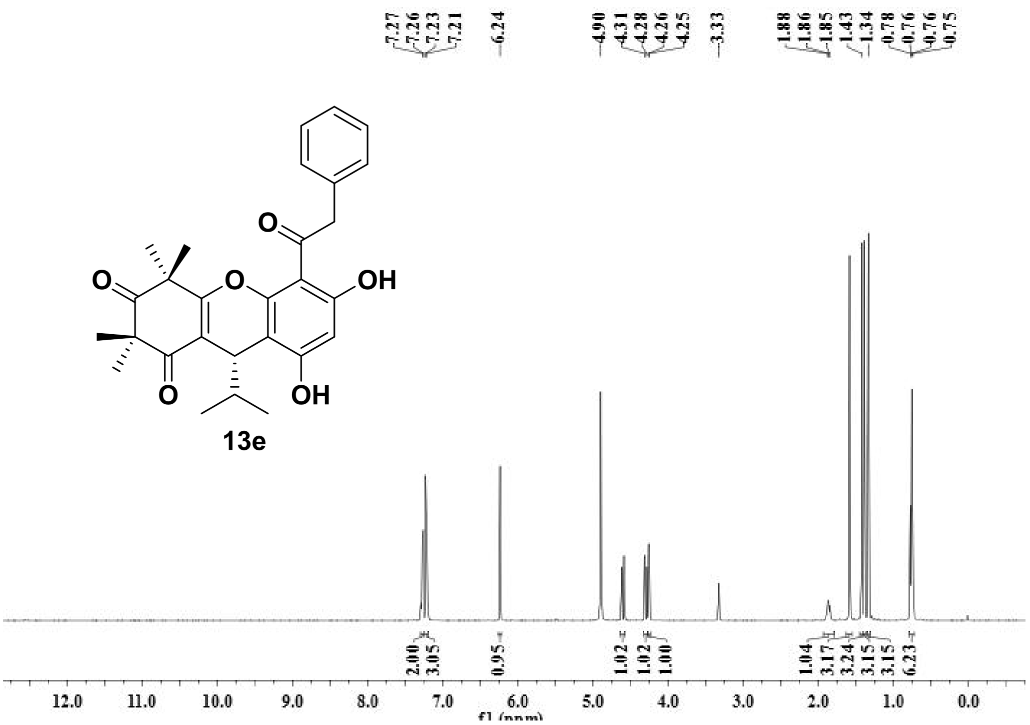
7.27 7.26 7.23 7.21
6.24
4.90 4.31 4.28 4.26 4.25
3.33
1.88 1.86 1.85 1.43 1.34 0.78 0.76 0.76 0.75
O
OH
O
O
O
OH
13e
2.00 3.05
0.95
1.02 1.02 1.00
1.04 3.17 3.24 3.15 3.15 6.23
12.0 11.0 10.0 9.0 8.0 7.0 6.0 5.0 4.0 3.0 2.0 1.0 0.0
f1 (ppm)

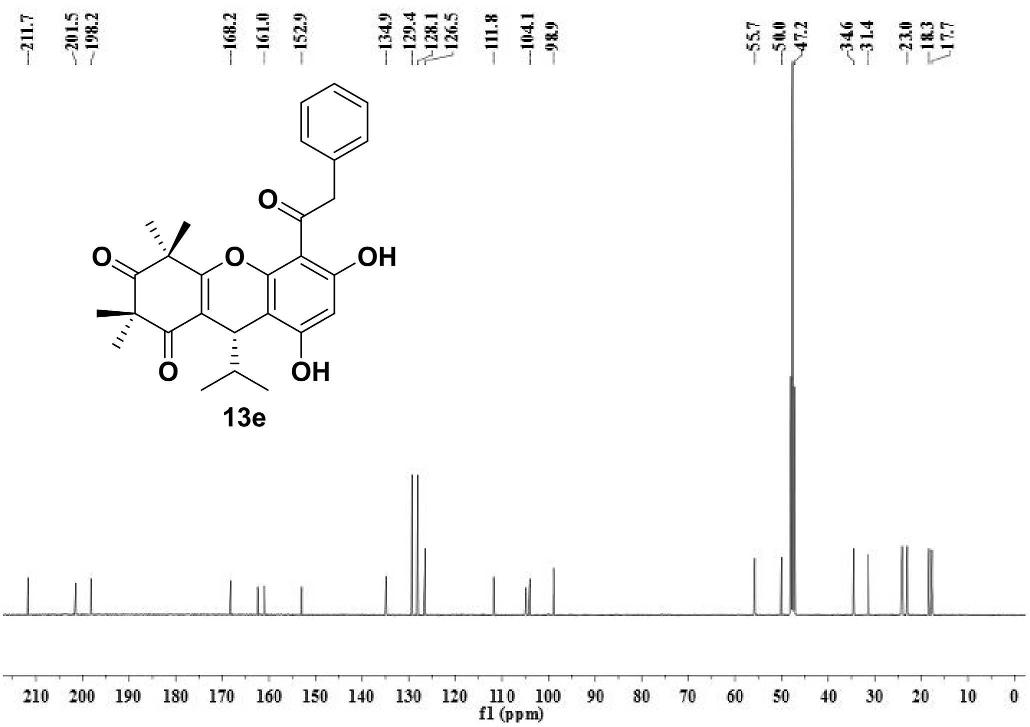
211.7
201.5 198.2
168.2 161.0 152.9
134.9 129.4 128.1 126.5
111.8 104.1 98.9
55.7 50.0 47.2
34.6 31.4
23.0 18.3 17.7
O
OH
O
O
O
OH
13e
210 200 190 180 170 160 150 140 130 120 110 100 90 80 70 60 50 40 30 20 10 0
f1 (ppm)

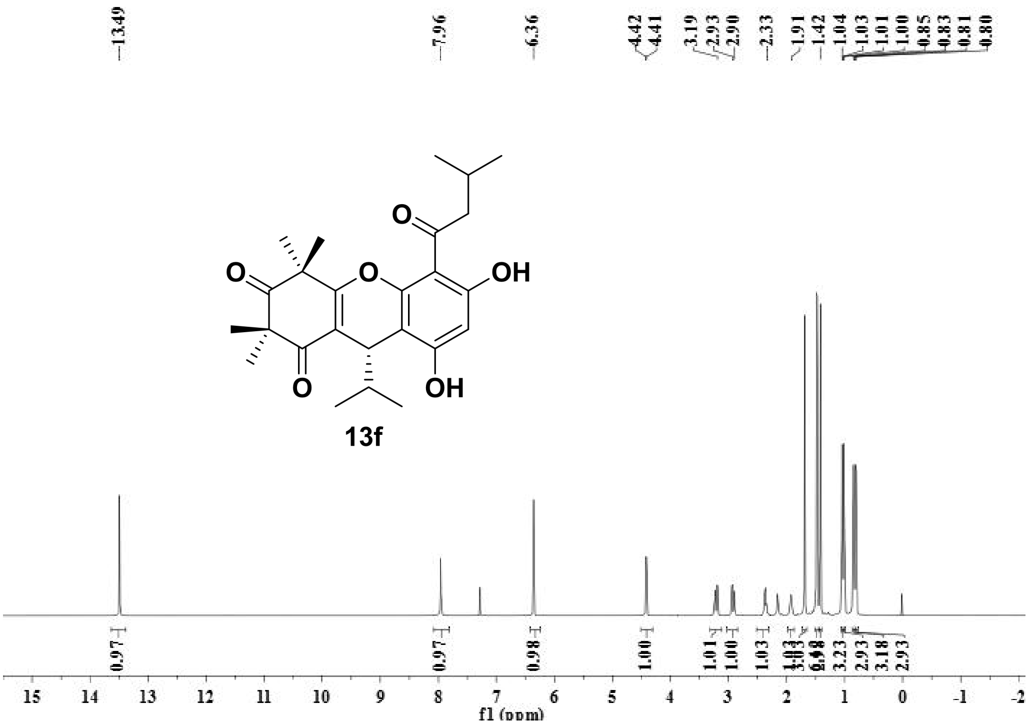
13f
O
OH
f1 (ppm)

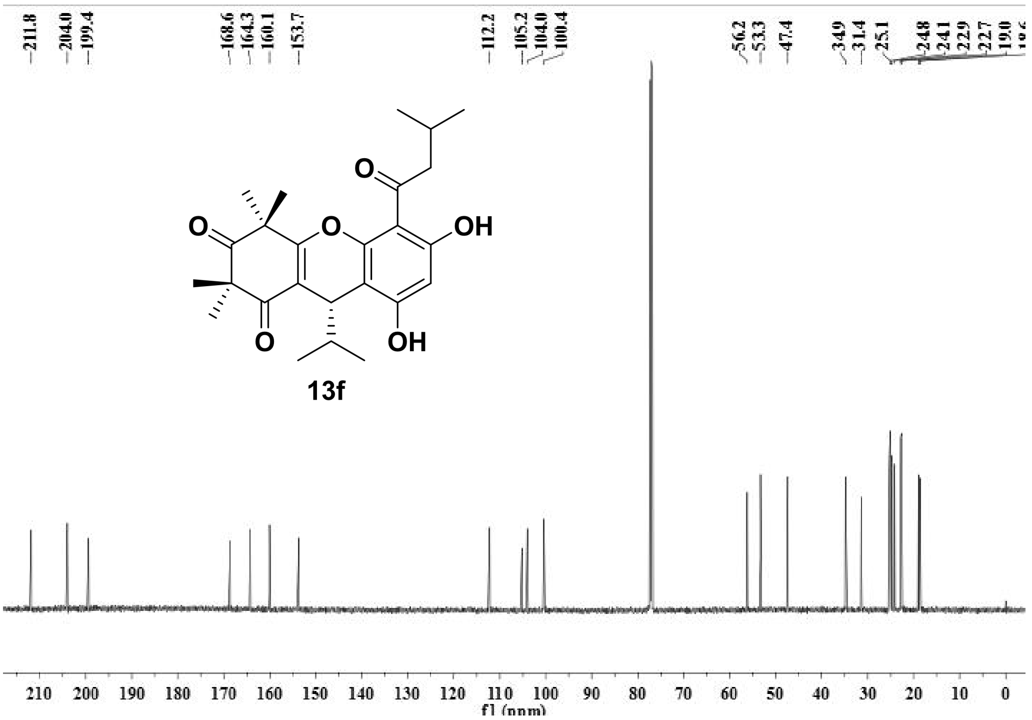
13f
O
OH
f1 (ppm)

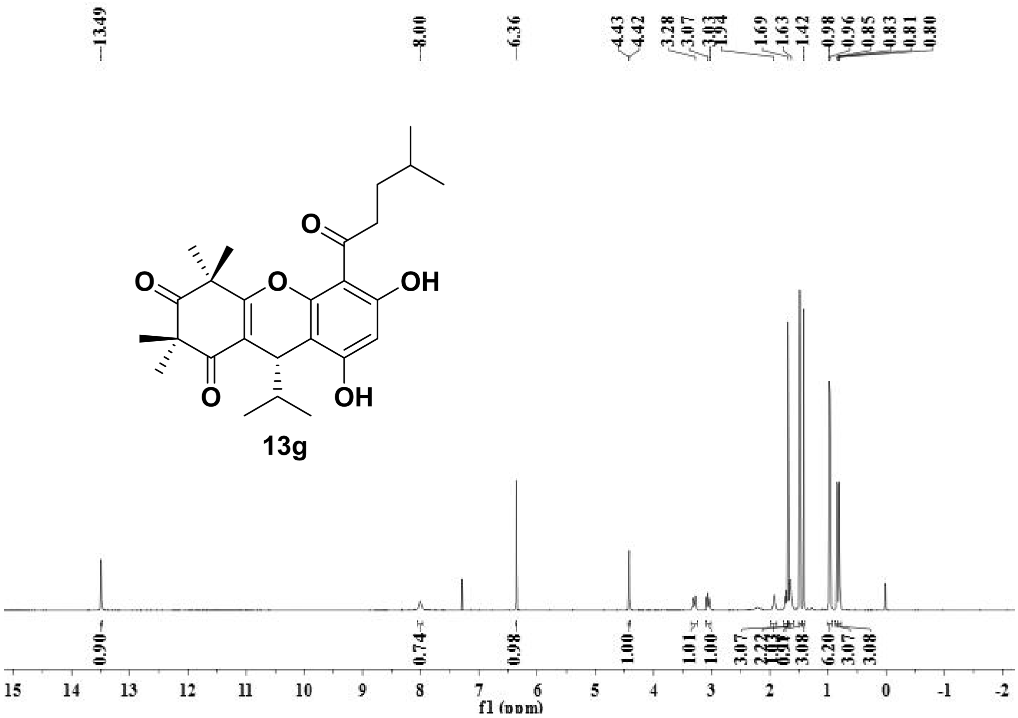
13.49
8.00
6.36
4.43
4.42
3.28
3.07
1.69
1.63
1.42
0.98
0.96
0.85
0.83
0.81
0.80
13g
0.90
0.74
0.98
1.00
1.01
1.00
6.20
3.07
3.08
f1 (ppm)

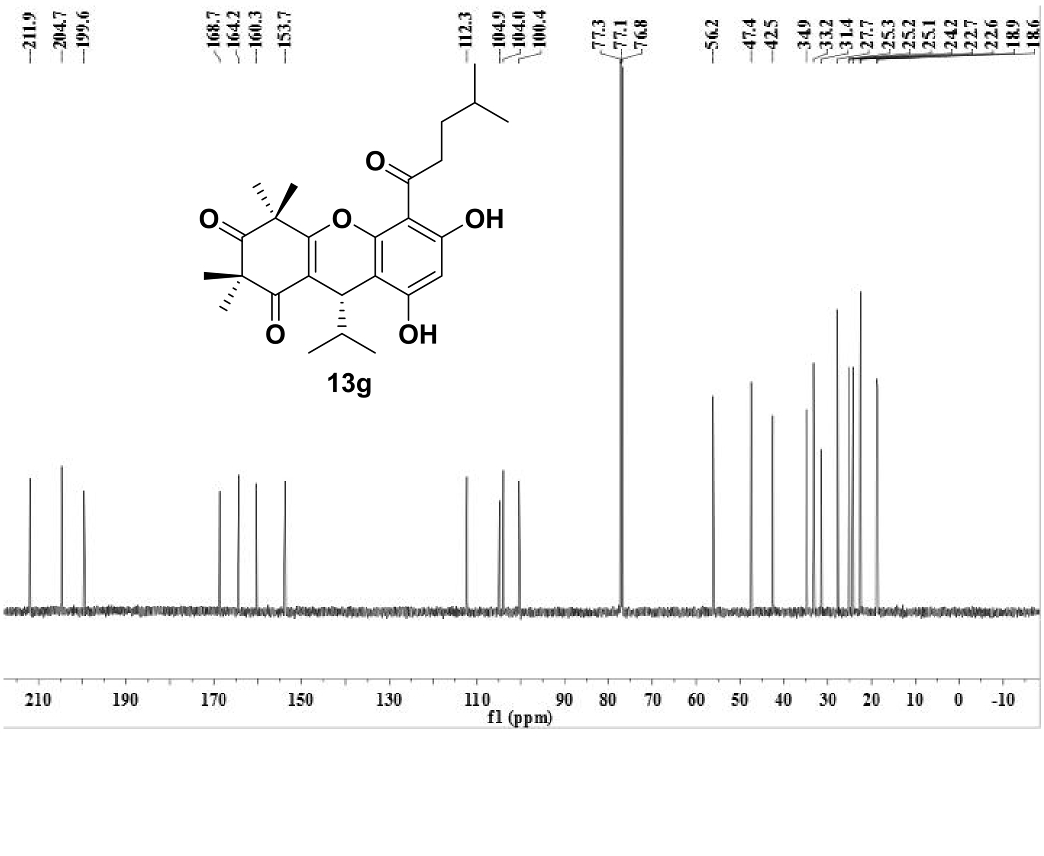
211.9
204.7
199.6
168.7
164.2
160.3
153.7
112.3
104.9
104.0
100.4
77.3
77.1
76.8
56.2
47.4
42.5
34.9
33.2
31.4
27.7
25.3
25.2
25.1
24.2
22.7
22.6
18.9
18.6
13g
f1 (ppm)

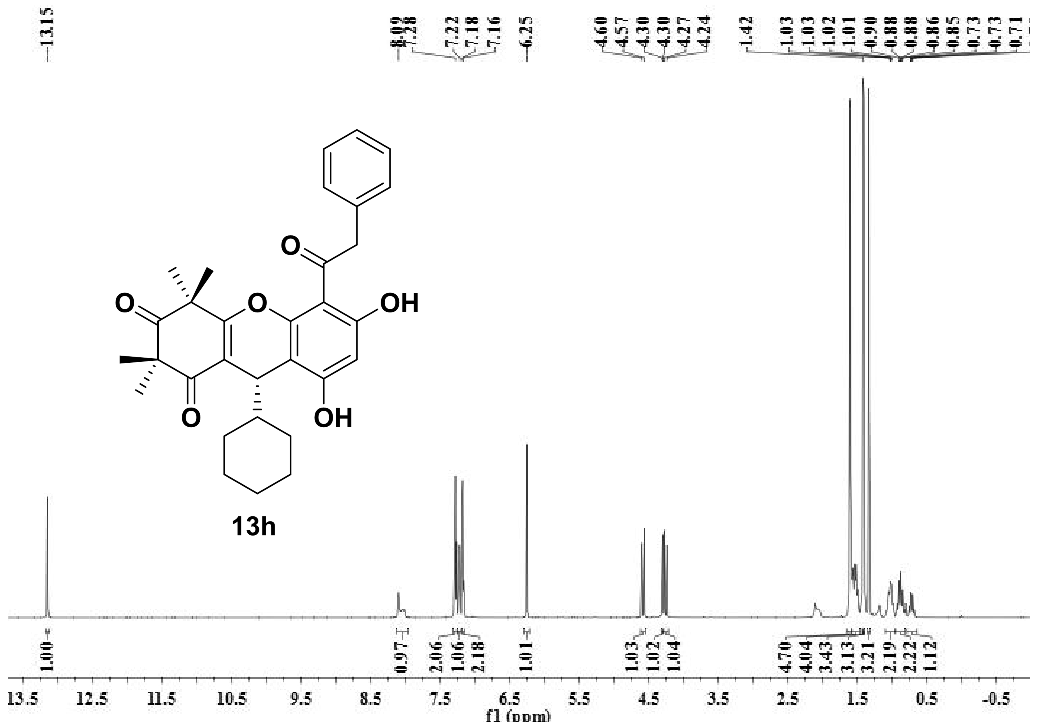

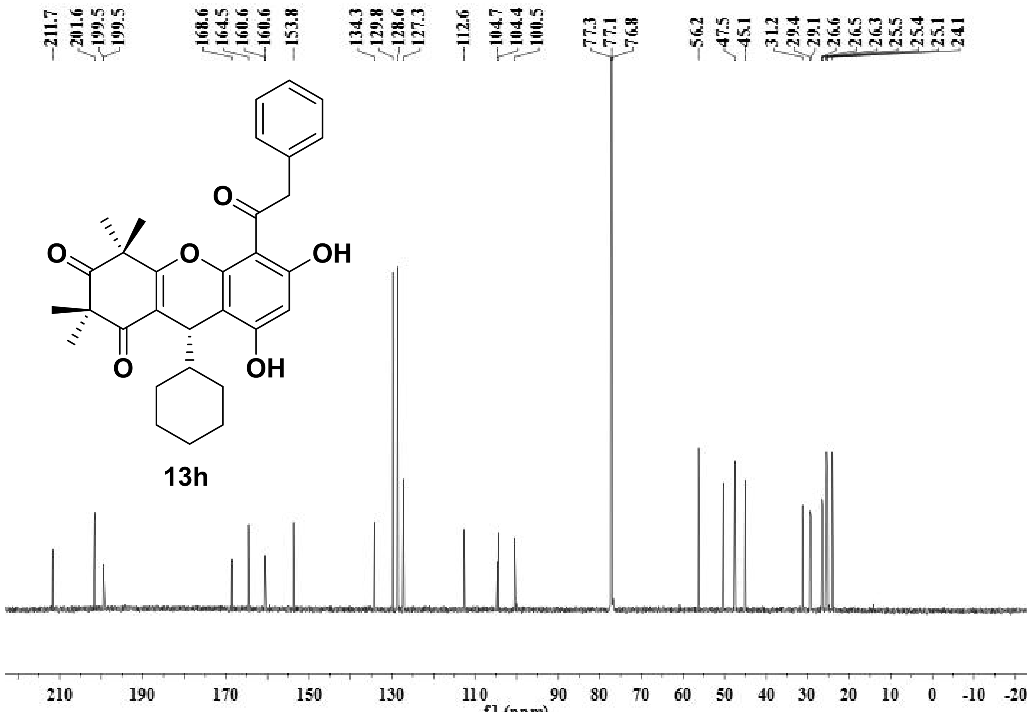

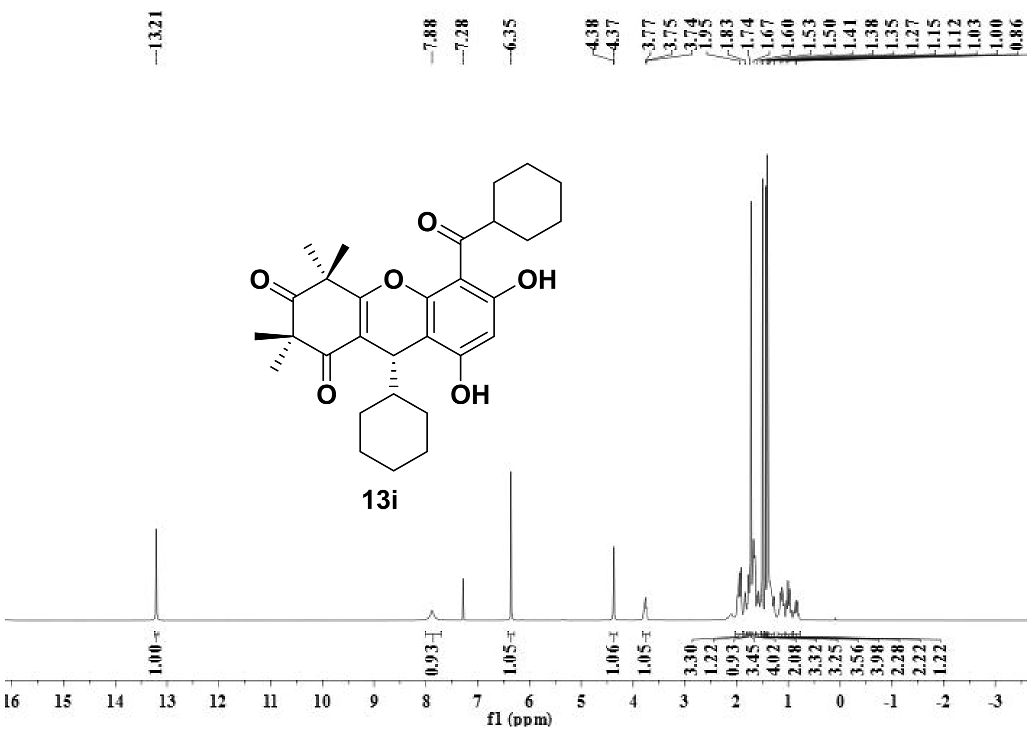

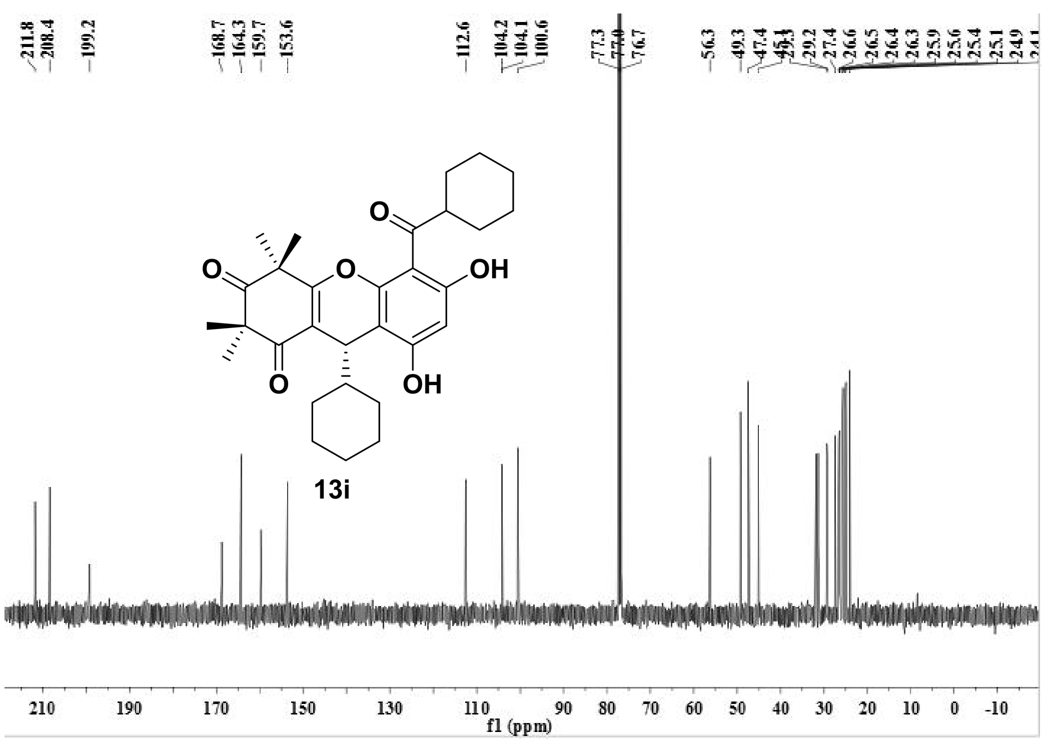

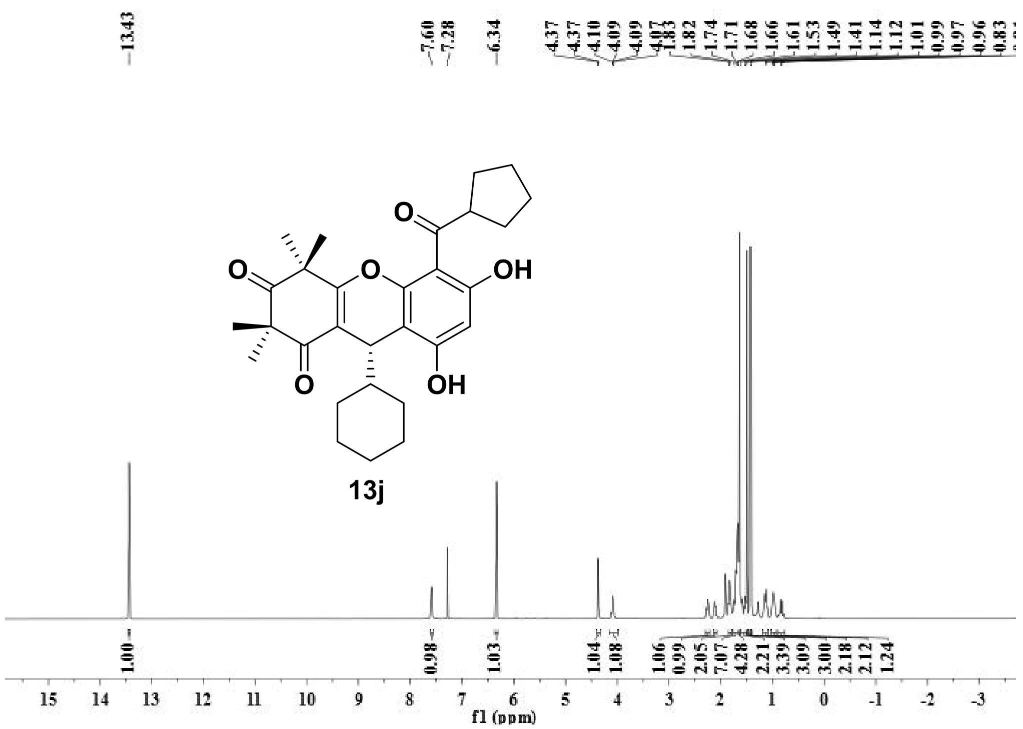

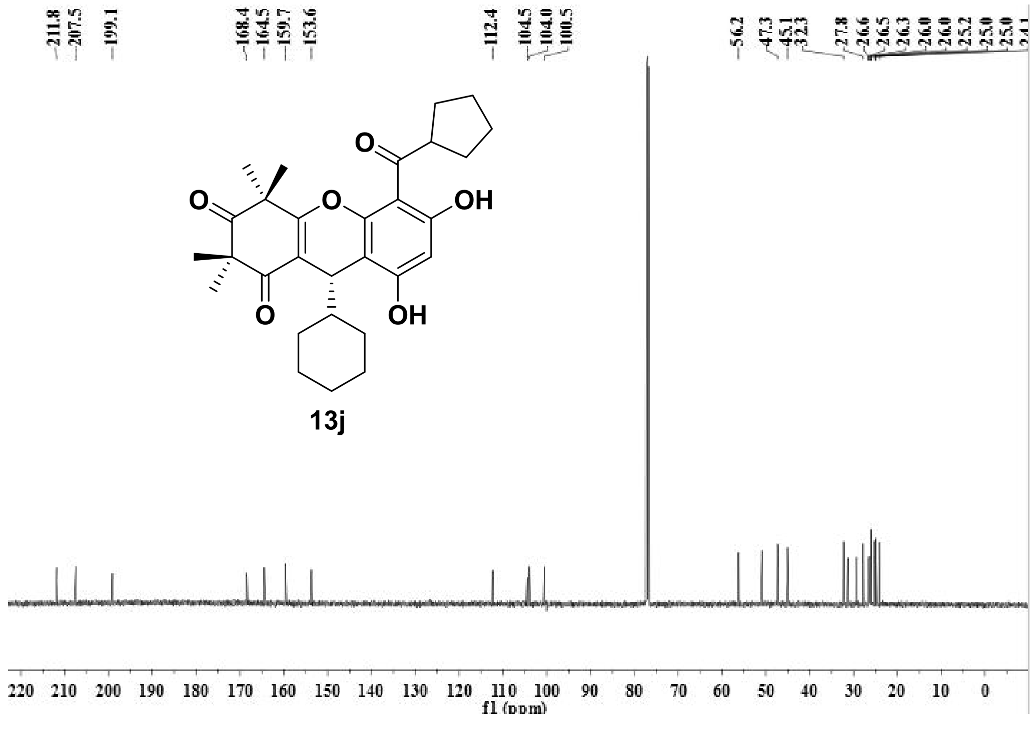

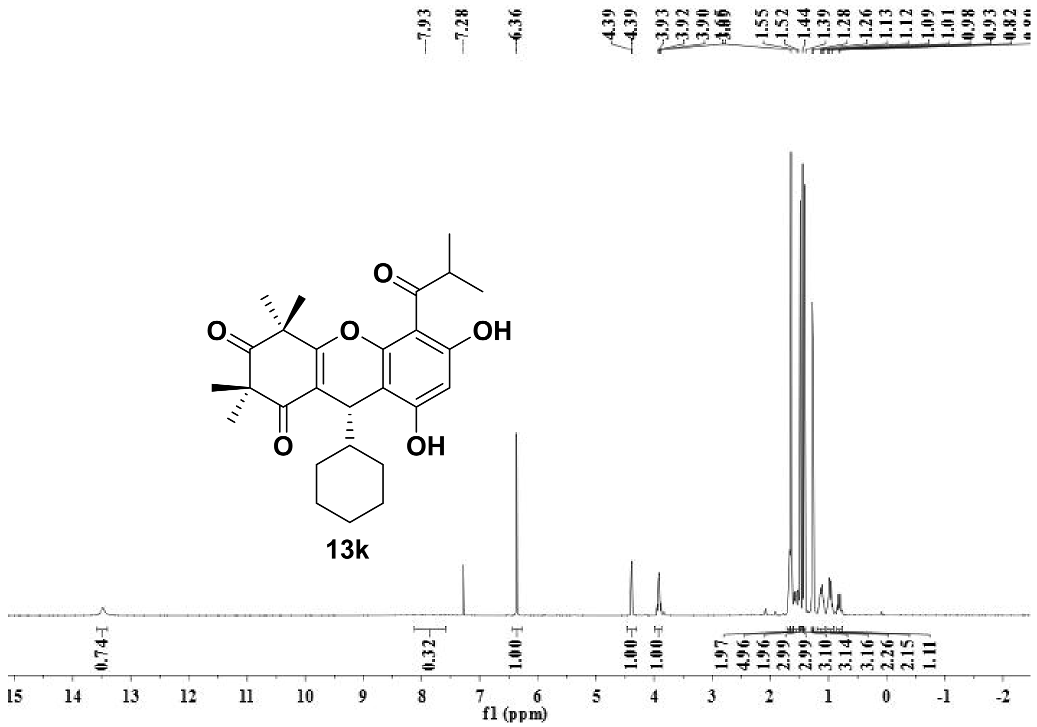
13k
fl (ppm)

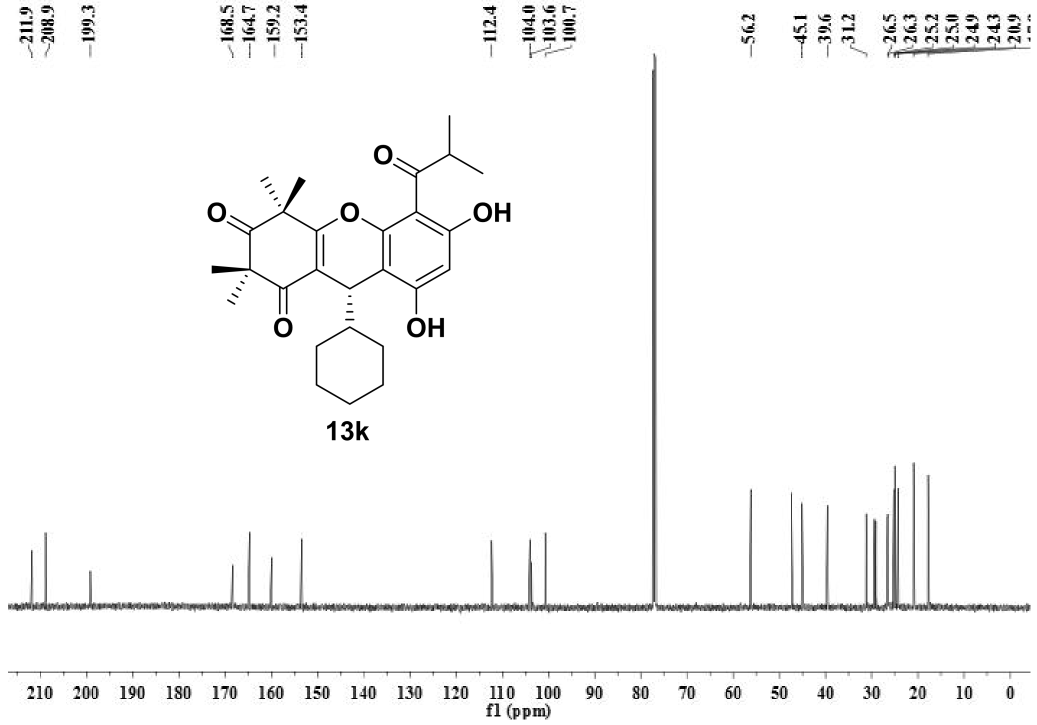
13k
fl (ppm)

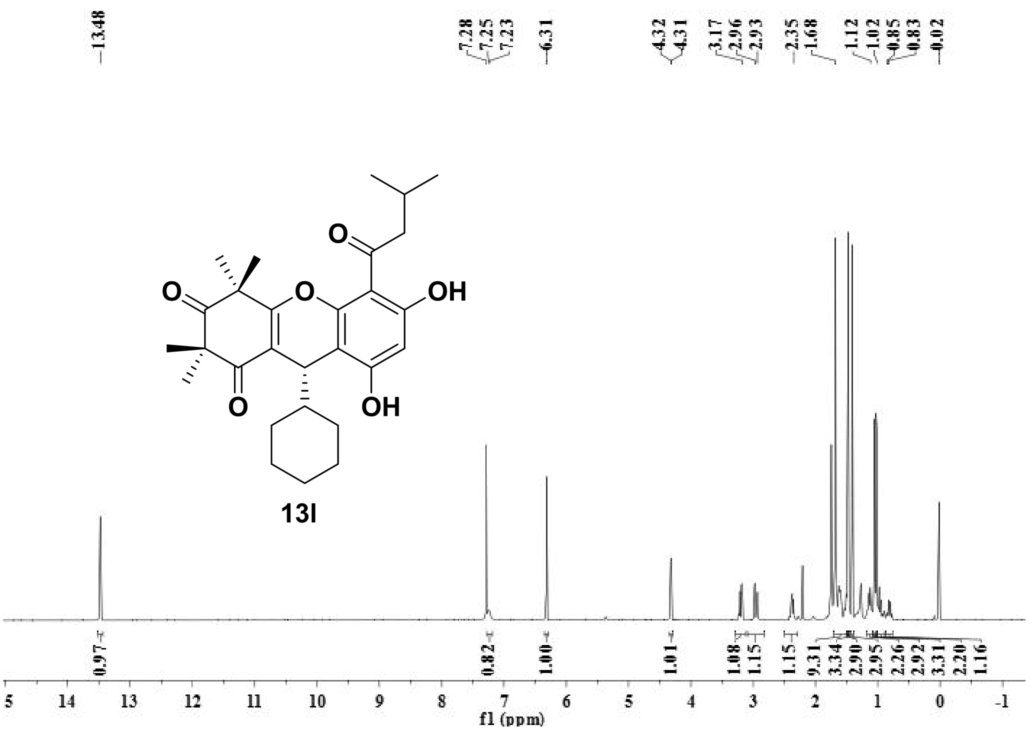
13l
OH
OH
O
O
O
O
f1 (ppm)

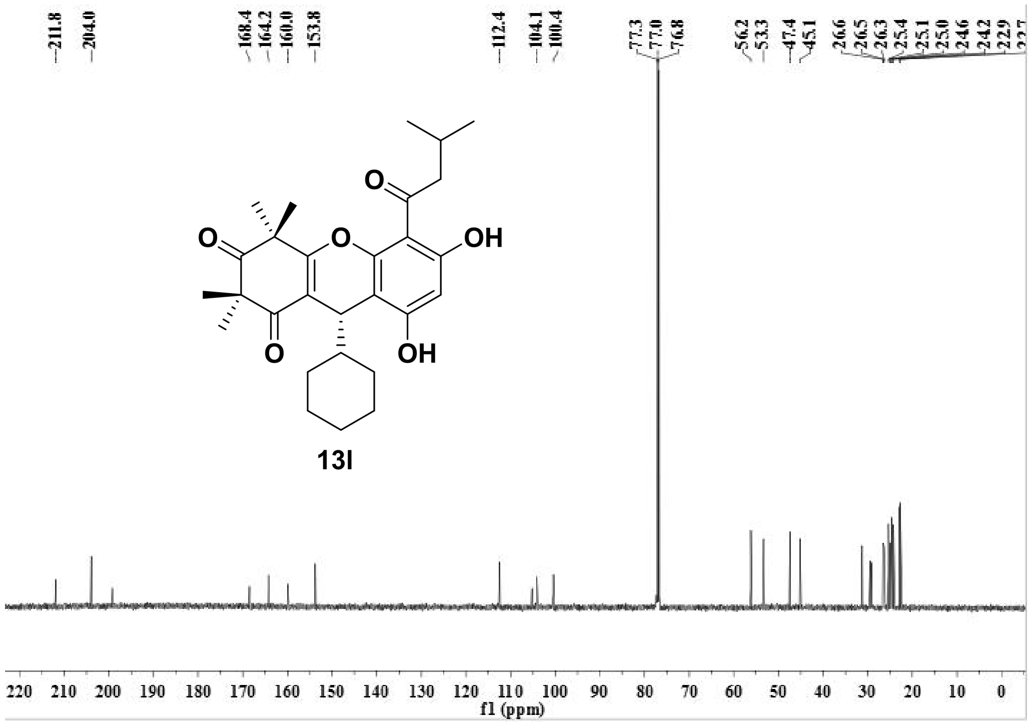
13l
OH
OH
O
O
O
O
f1 (ppm)

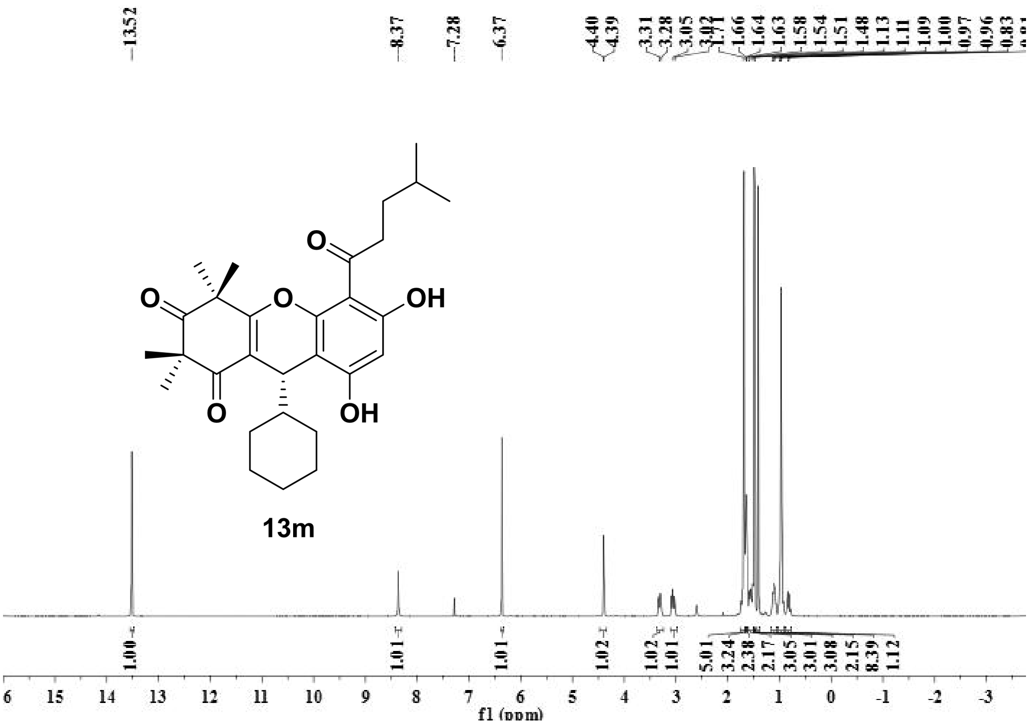

13.52
8.37
7.28
6.37
4.40
4.39
3.31
3.28
3.05
1.66
1.64
1.63
1.58
1.54
1.51
1.48
1.13
1.11
1.09
1.00
0.97
0.96
0.83
O
OH
OH
13m
1.00
1.01
1.01
1.02
1.02
1.01
5.01
3.24
2.38
2.17
3.05
3.01
3.08
2.15
8.39
1.12
fl (ppm)


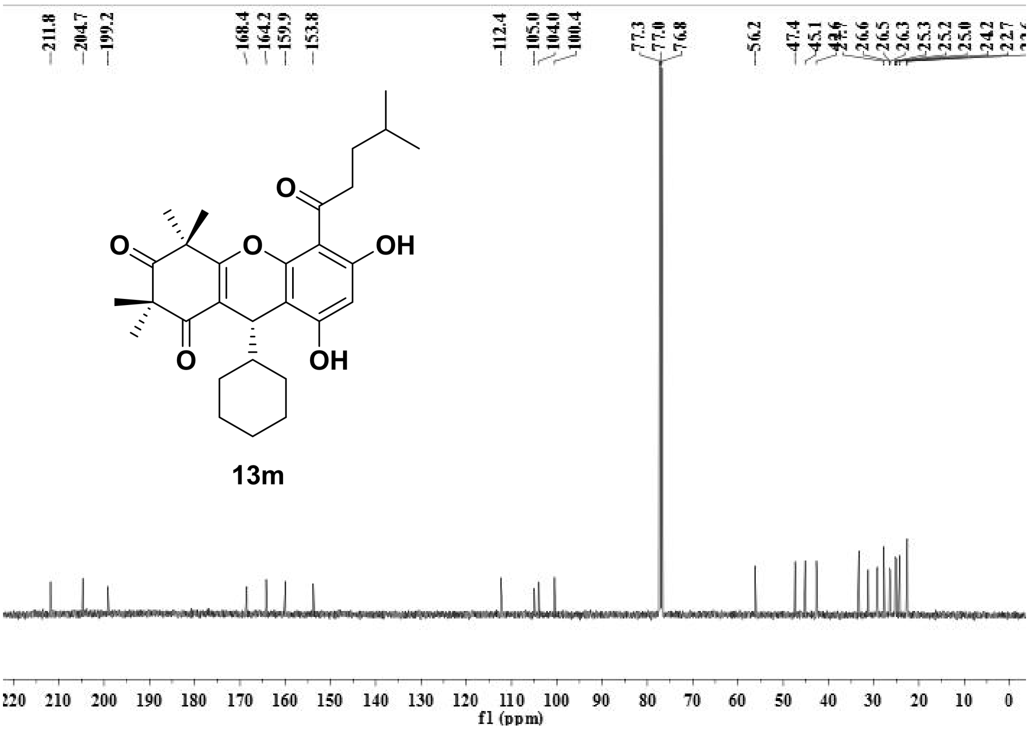

211.8
204.7
199.2
168.4
164.2
159.9
153.8
112.4
105.0
104.0
100.4
77.3
77.0
76.8
56.2
47.4
45.1
26.6
26.5
26.3
25.3
25.2
25.0
24.2
22.7
O
OH
OH
13m
fl (ppm)

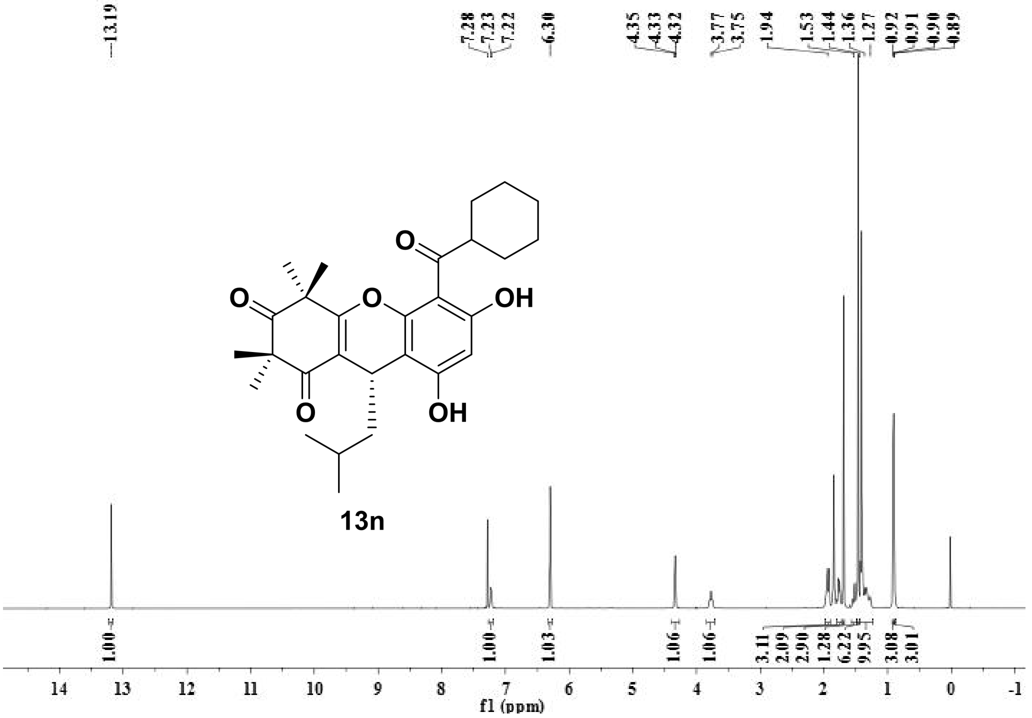

13n

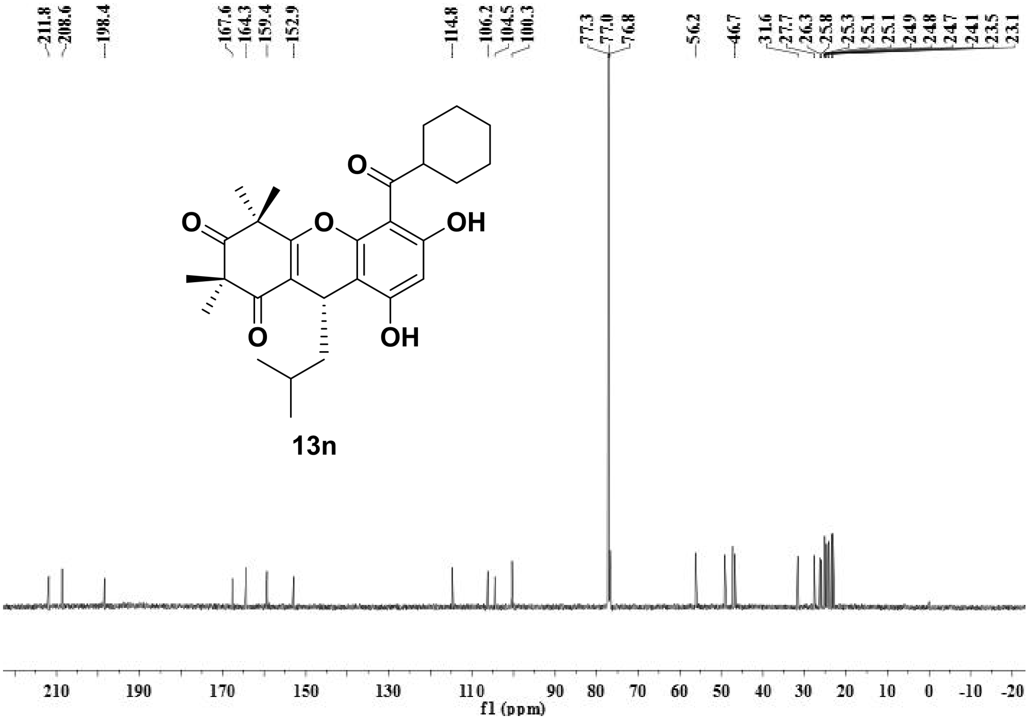

13n

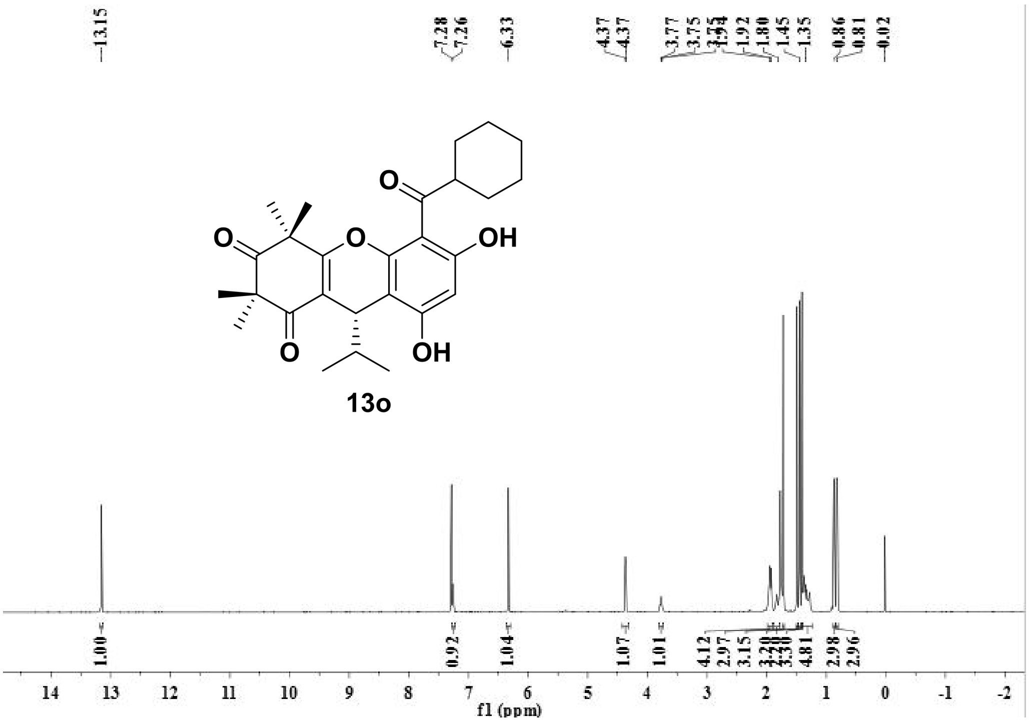
13.15
7.28
7.26
6.33
4.37
4.37
3.77
3.75
1.92
1.80
1.45
1.35
0.86
0.81
0.02
OH
OH
O
O
O
O
13o
1.00
0.92
1.04
1.07
1.01
4.12
2.97
3.15
4.81
2.98
2.96
f1 (ppm)

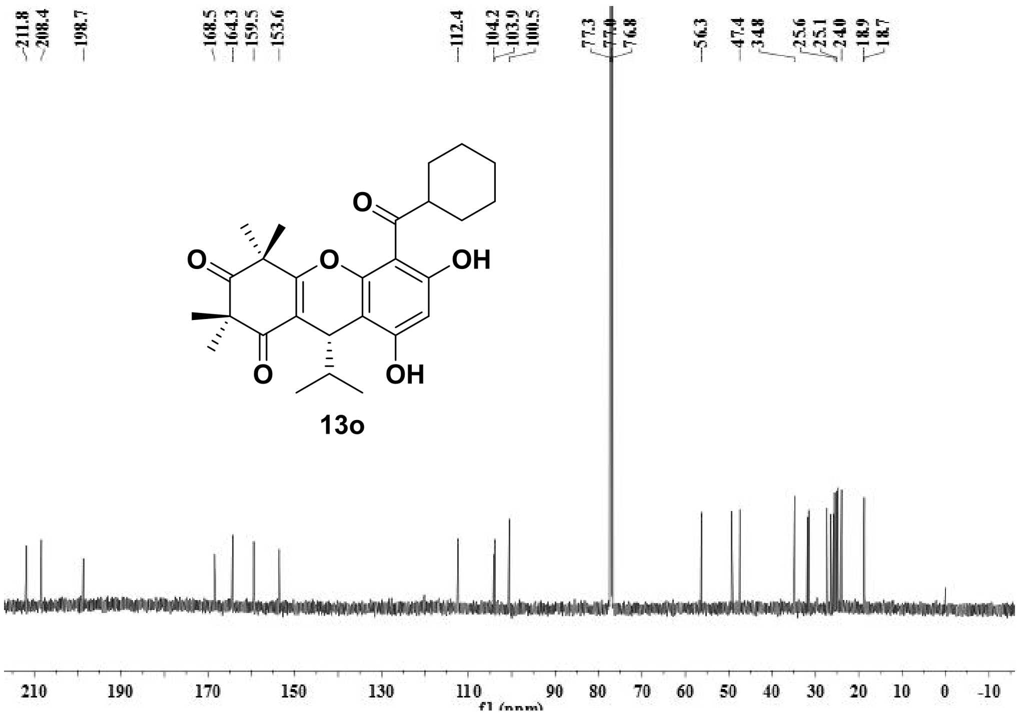
211.8
208.4
198.7
168.5
164.3
159.5
153.6
112.4
104.2
103.9
100.5
77.3
77.0
76.8
56.3
47.4
34.8
25.6
25.1
24.0
18.9
18.7
OH
OH
O
O
O
O
13o

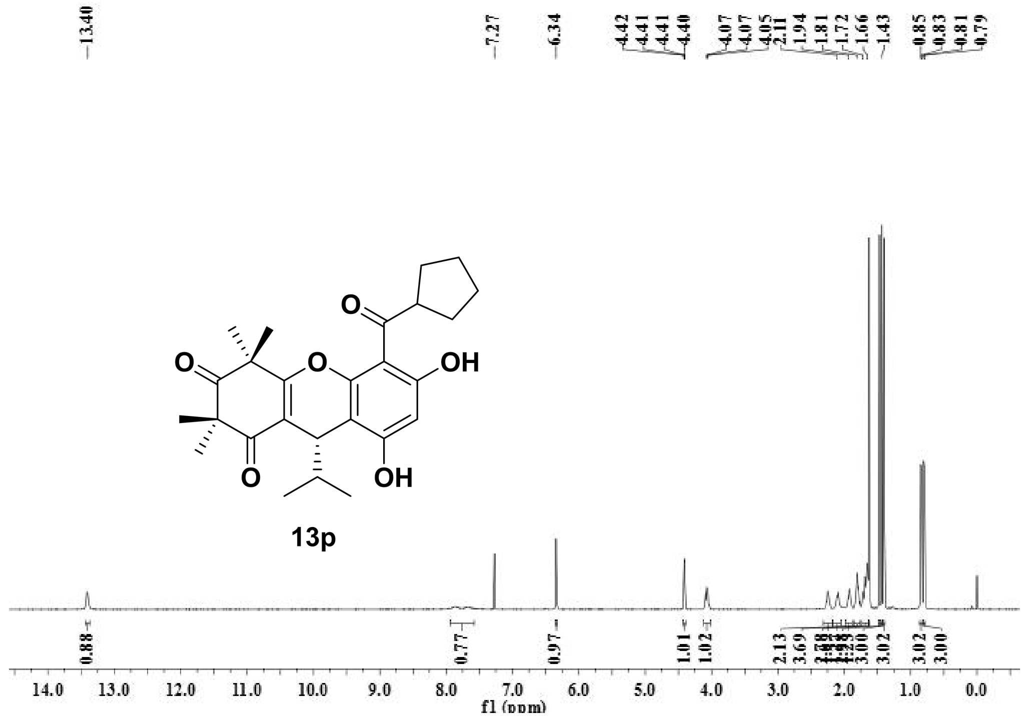

13p
OH
OH
O
f1 (ppm)


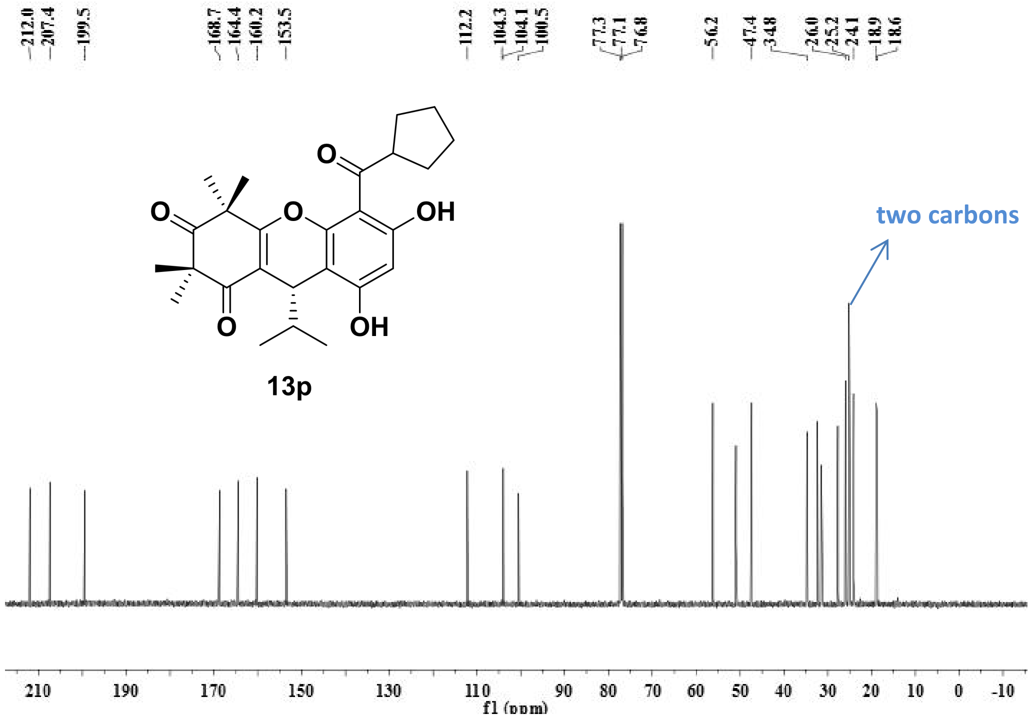

13p
two carbons
f1 (ppm)

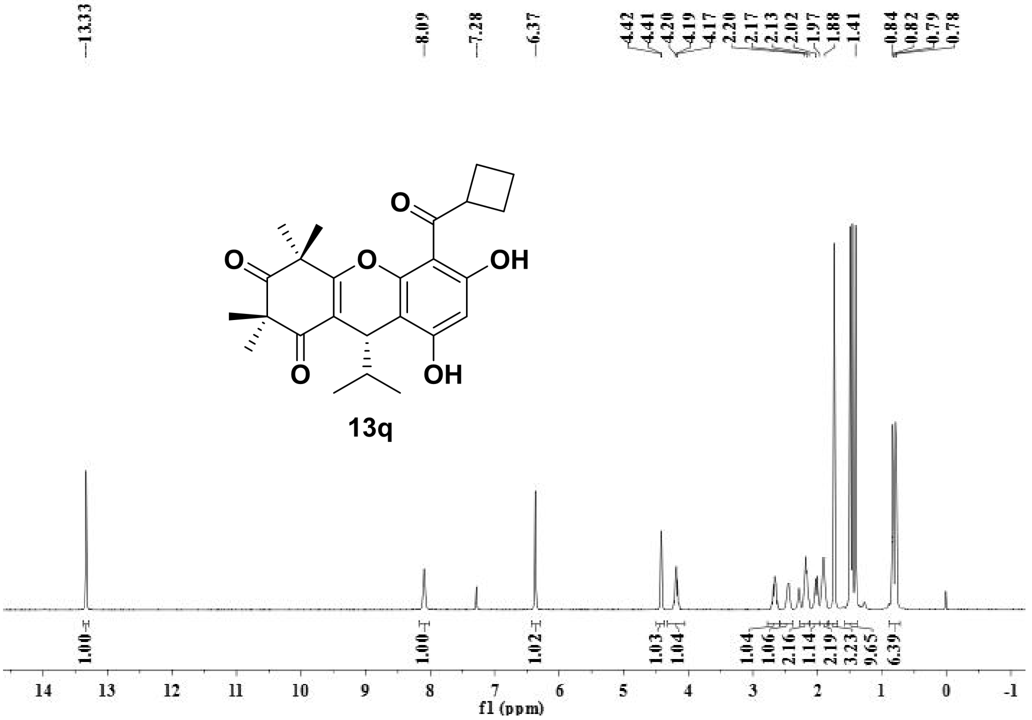

13.33
8.09
7.28
6.37
4.42
4.41
4.20
4.19
4.17
2.20
2.17
2.13
2.02
1.97
1.88
1.41
0.84
0.82
0.79
0.78
O
OH
O
O
O
OH
13q
1.00
1.00
1.02
1.03
1.04
1.04
1.06
2.16
1.14
2.19
3.23
9.65
6.39
14 13 12 11 10 9 8 7 6 5 4 3 2 1 0 -1
f1 (ppm)


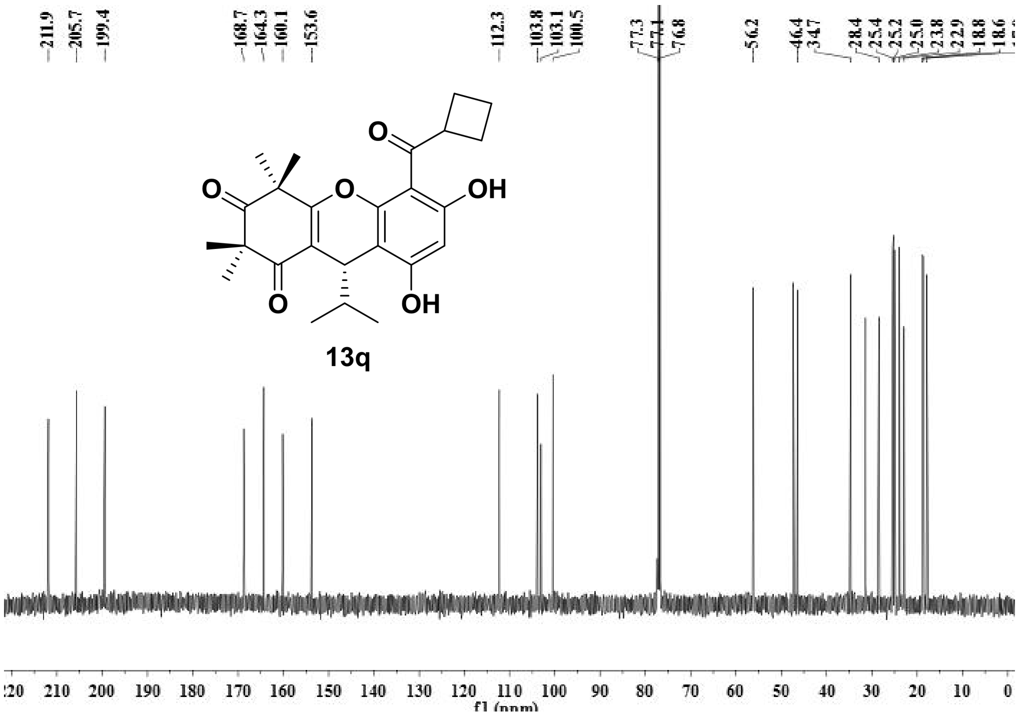

211.9
205.7
199.4
168.7
164.3
160.1
153.6
112.3
103.8
103.1
100.5
77.3
77.1
76.8
56.2
46.4
34.7
28.4
25.4
25.2
25.0
23.8
22.9
18.8
18.6
O
OH
O
O
O
OH
13q
210 200 190 180 170 160 150 140 130 120 110 100 90 80 70 60 50 40 30 20 10 0
f1 (ppm)

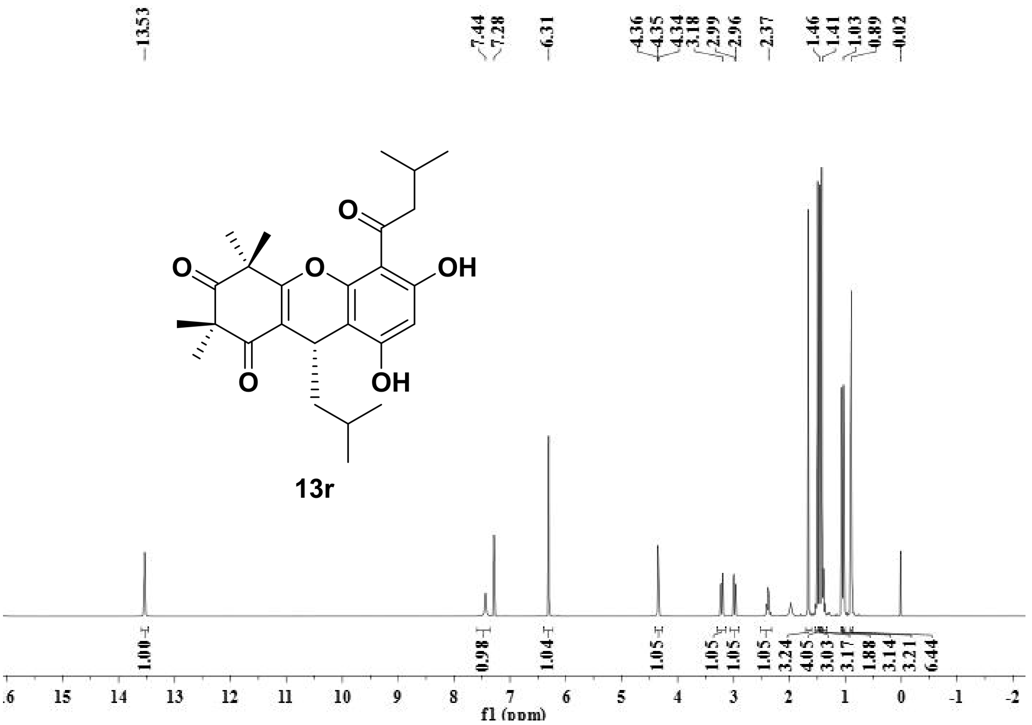
13.53
7.44
7.28
6.31
4.36
4.35
4.34
3.18
2.99
2.96
2.37
1.46
1.41
1.03
0.89
0.02
OH
OH
O
O
O
O
13r
1.00
0.98
1.04
1.05
1.05
1.05
1.05
3.24
4.05
3.03
3.17
1.88
3.14
3.21
6.44
f1 (ppm)

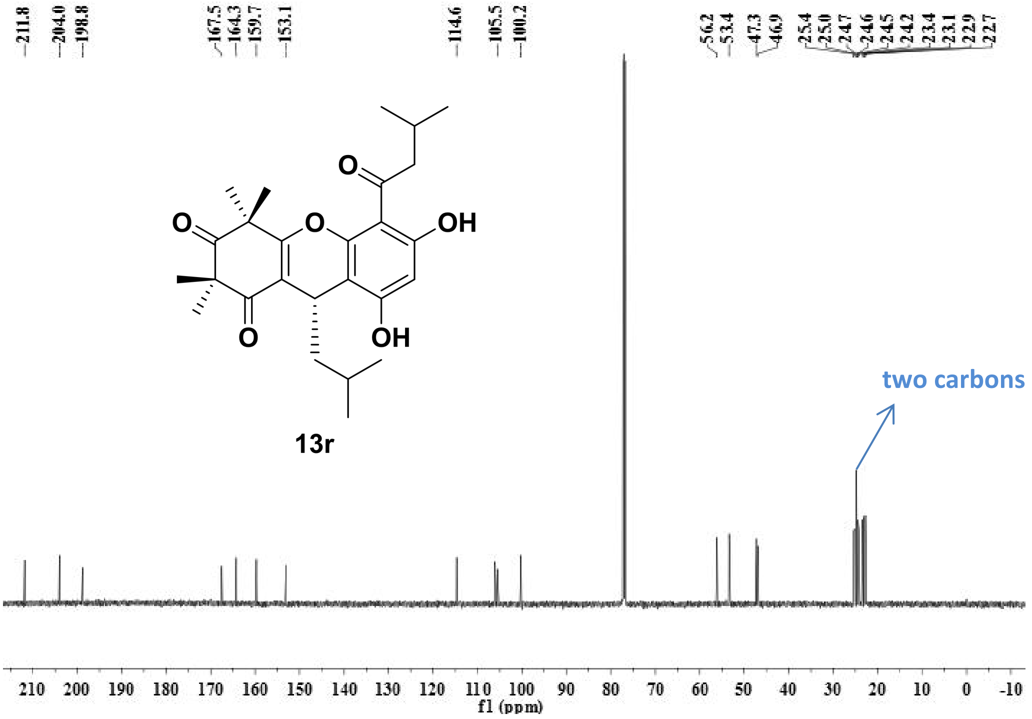
211.8
204.0
198.8
167.5
164.3
159.7
153.1
114.6
105.5
100.2
56.2
53.4
47.3
46.9
25.4
25.0
24.7
24.6
24.5
24.2
23.4
23.1
22.9
22.7
13r
two carbons
f1 (ppm)

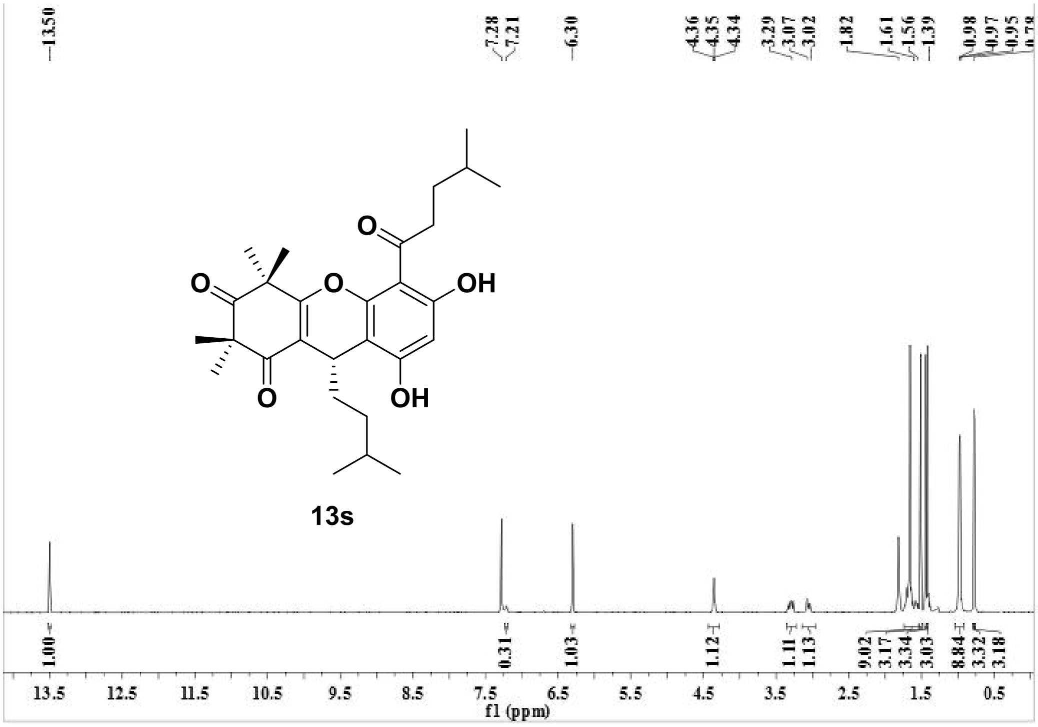

13.50
7.28
7.21
6.30
4.36
4.35
4.34
3.29
3.07
3.02
1.82
1.61
1.56
1.39
0.98
0.97
0.95
O
O
OH
O
O
OH
13s
1.00
0.31
1.03
1.12
1.11
1.13
9.02
3.17
3.34
3.03
8.84
3.32
3.18
13.5
12.5
11.5
10.5
9.5
8.5
7.5
6.5
5.5
4.5
3.5
2.5
1.5
0.5
f1 (ppm)


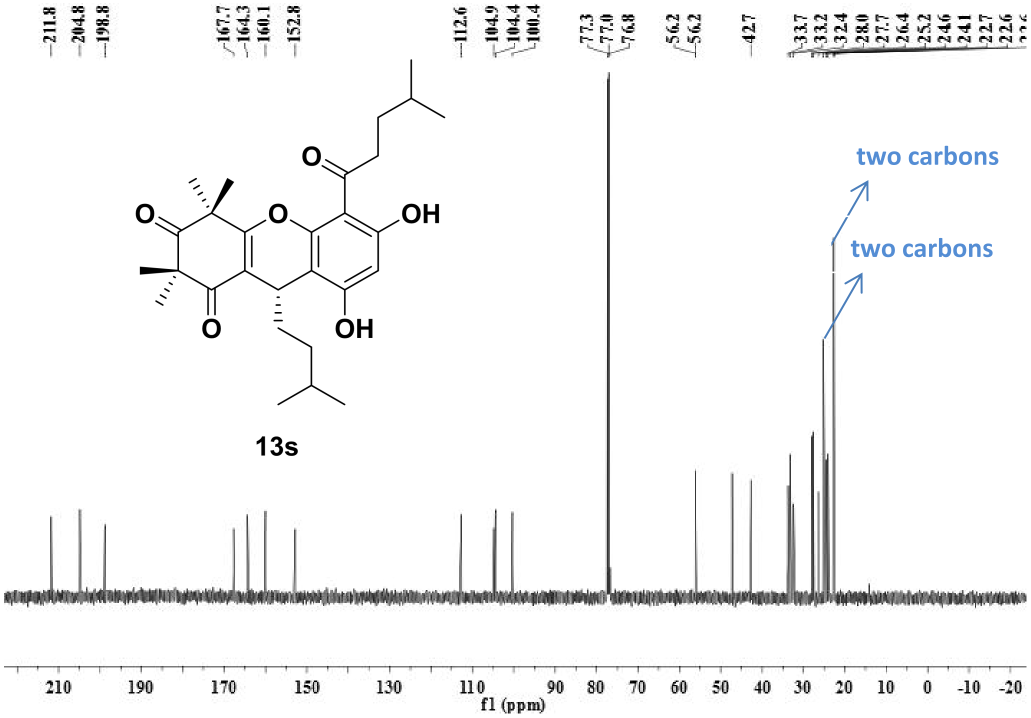

211.8
204.8
198.8
167.7
164.3
160.1
152.8
112.6
104.9
104.4
100.4
77.3
77.0
76.8
56.2
56.2
42.7
33.7
33.2
32.4
28.0
27.7
26.4
25.2
24.6
24.1
22.7
22.6
two carbons
two carbons
O
O
OH
O
O
OH
13s
210
190
170
150
130
110
90
80
70
60
50
40
30
20
10
0
-10
-20
f1 (ppm)

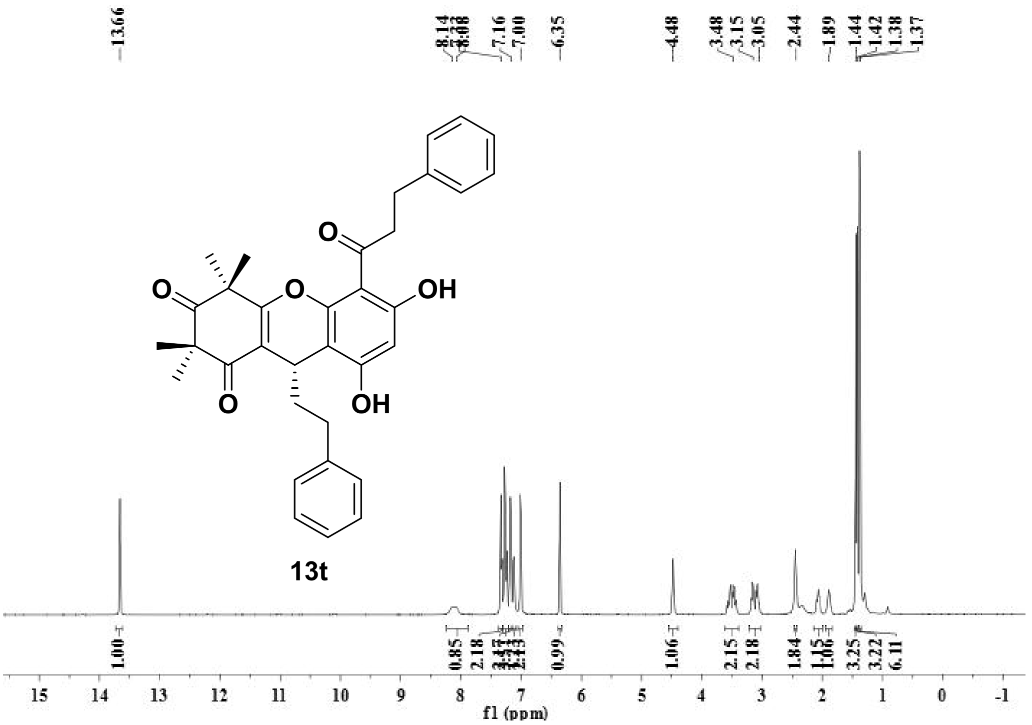
13t
fl (ppm)

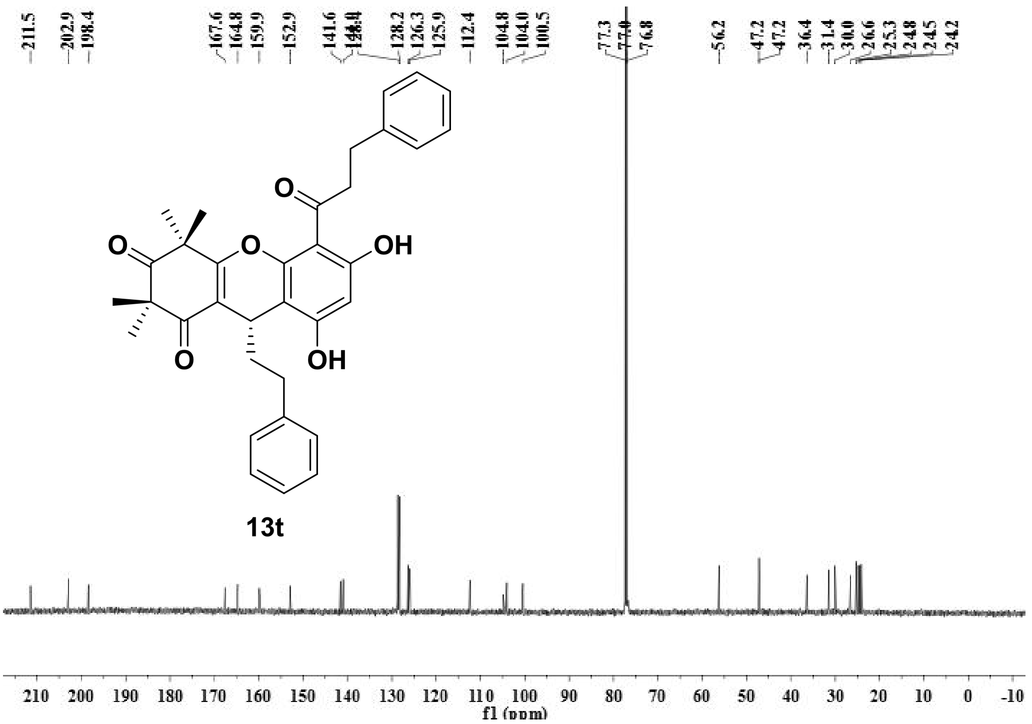
13t
fl (ppm)

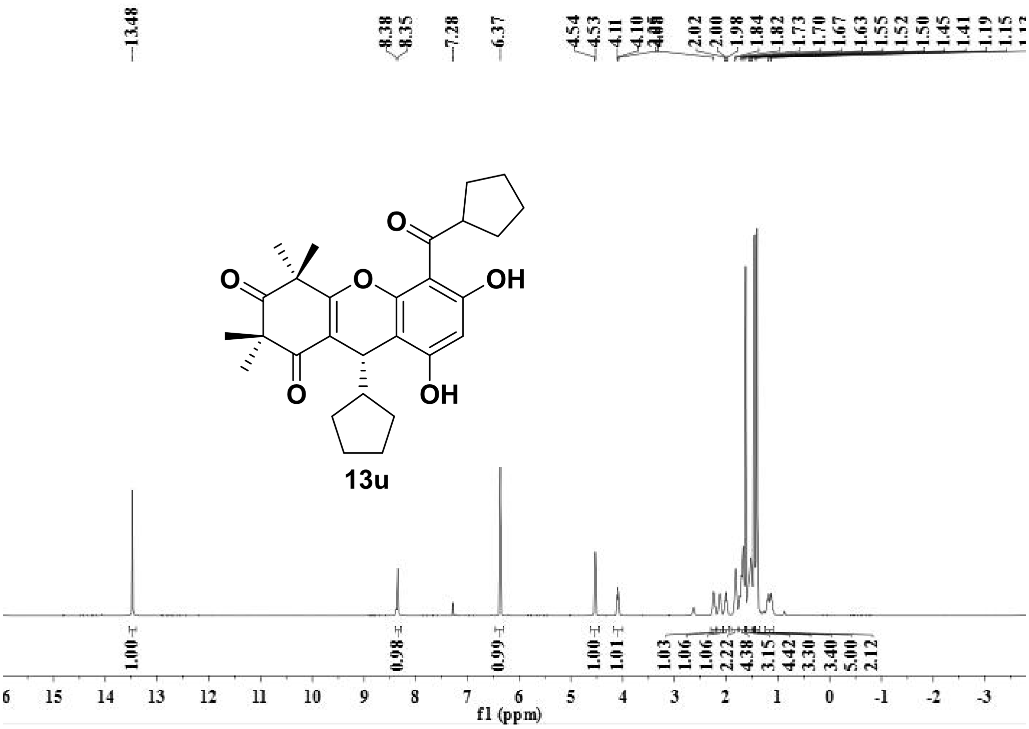
13.48
8.38
8.35
7.28
6.37
4.54
4.53
4.11
4.10
2.02
2.00
1.98
1.84
1.82
1.73
1.70
1.67
1.63
1.55
1.52
1.50
1.45
1.41
1.19
1.15
O
O
O
O
OH
OH
O
13u
1.00
0.98
0.99
1.00
1.01
1.03
1.06
1.06
2.22
4.38
3.15
4.42
3.30
3.40
5.00
2.12
f1 (ppm)

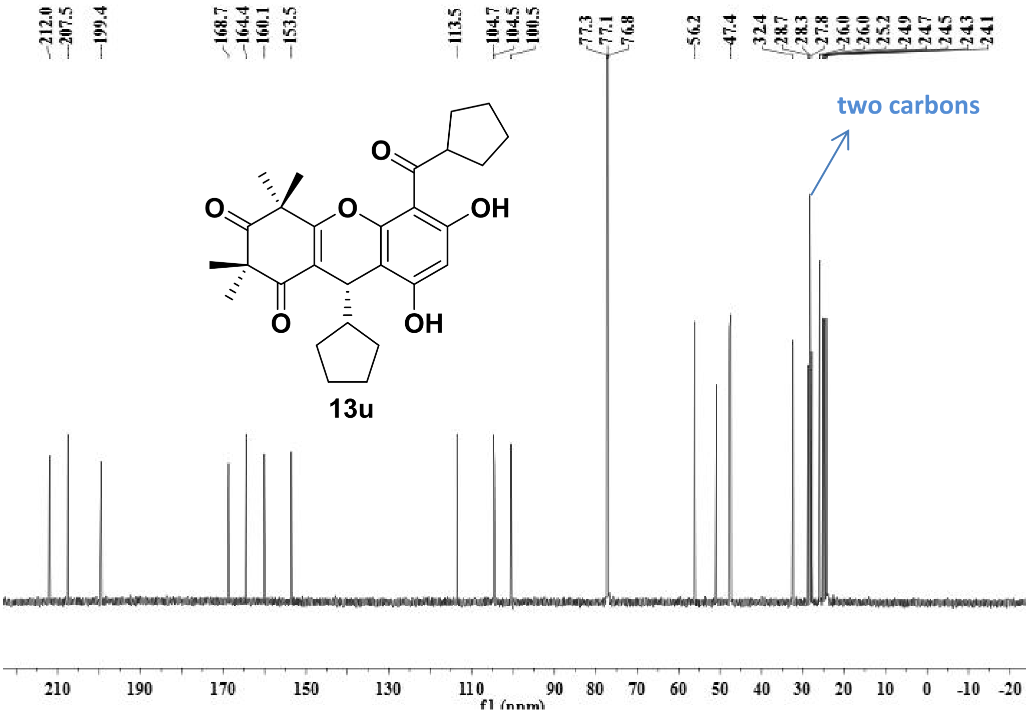
212.0
207.5
199.4
168.7
164.4
160.1
153.5
113.5
104.7
104.5
100.5
77.3
77.1
76.8
56.2
47.4
32.4
28.7
28.3
27.8
26.0
26.0
25.2
24.9
24.7
24.5
24.3
24.1
two carbons
O
O
O
O
OH
OH
O
13u

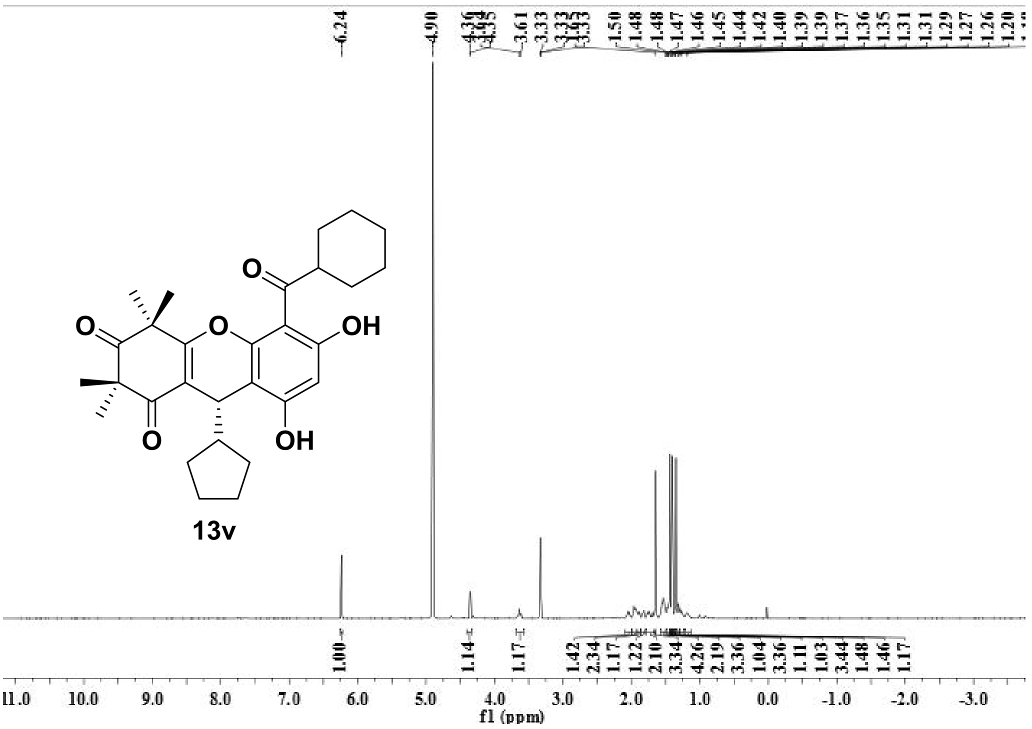

6.24
4.90
3.61
3.33
1.00
1.14
1.17
O
O
O
OH
OH
13v
f1 (ppm)


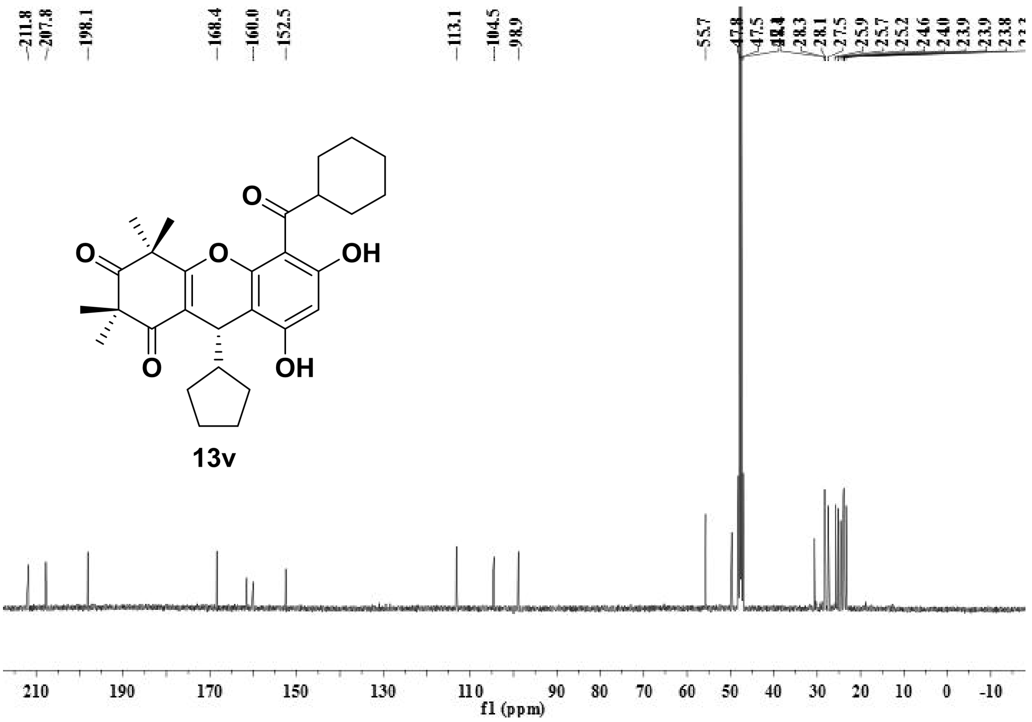

211.8
207.8
198.1
168.4
160.0
152.5
113.1
104.5
98.9
55.7
O
O
O
OH
OH
13v
f1 (ppm)

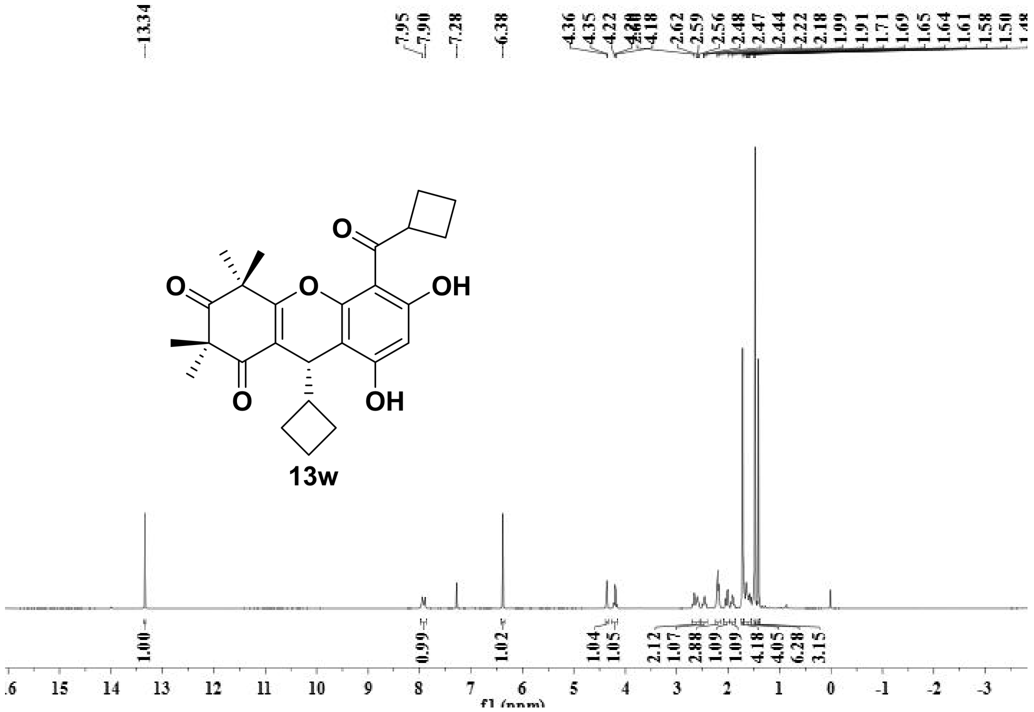
13.34
7.95
7.90
7.28
6.38
4.36
4.35
4.22
4.18
2.62
2.59
2.56
2.48
2.47
2.44
2.22
2.18
1.99
1.91
1.71
1.69
1.65
1.64
1.61
1.58
1.50
O
OH
OH
O
O
O
13w
1.00
0.99
1.02
1.04
1.05
2.12
1.07
2.88
1.09
1.09
4.18
4.05
6.28
3.15
f1 (ppm)

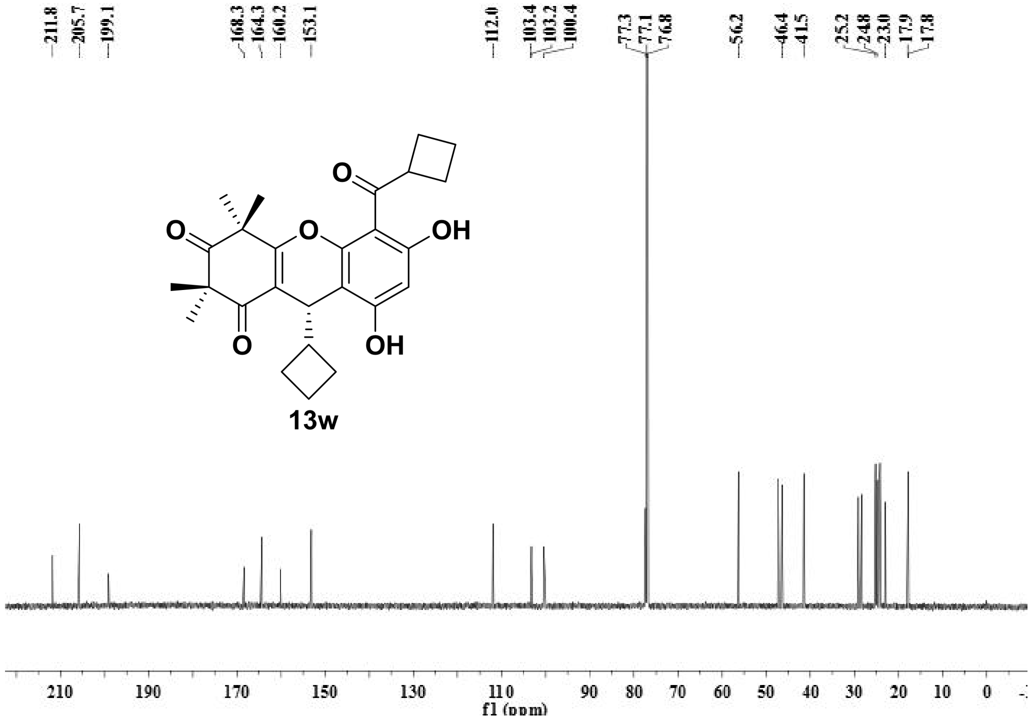
211.8
205.7
199.1
168.3
164.3
160.2
153.1
112.0
103.4
103.2
100.4
77.3
77.1
76.8
56.2
46.4
41.5
25.2
24.8
23.0
17.9
17.8
O
OH
OH
O
O
O
13w
f1 (ppm)

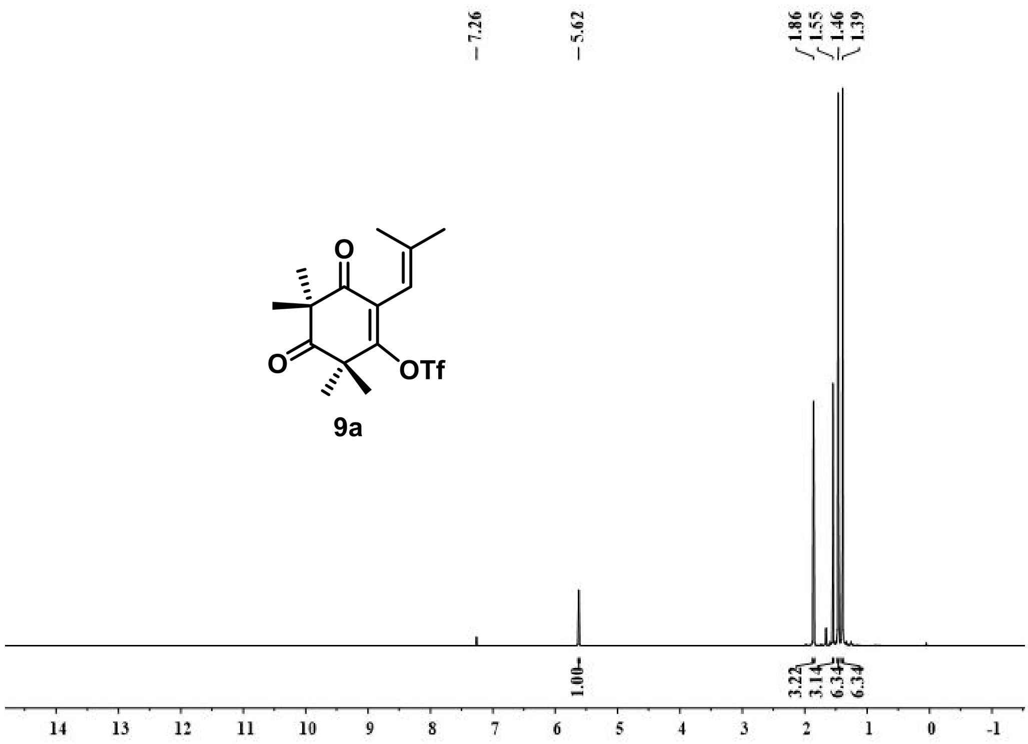
7.26
5.62
1.86
1.55
1.46
1.39
O
OTf
9a
1.00
3.22
3.14
6.34
6.34
14 13 12 11 10 9 8 7 6 5 4 3 2 1 0 -1

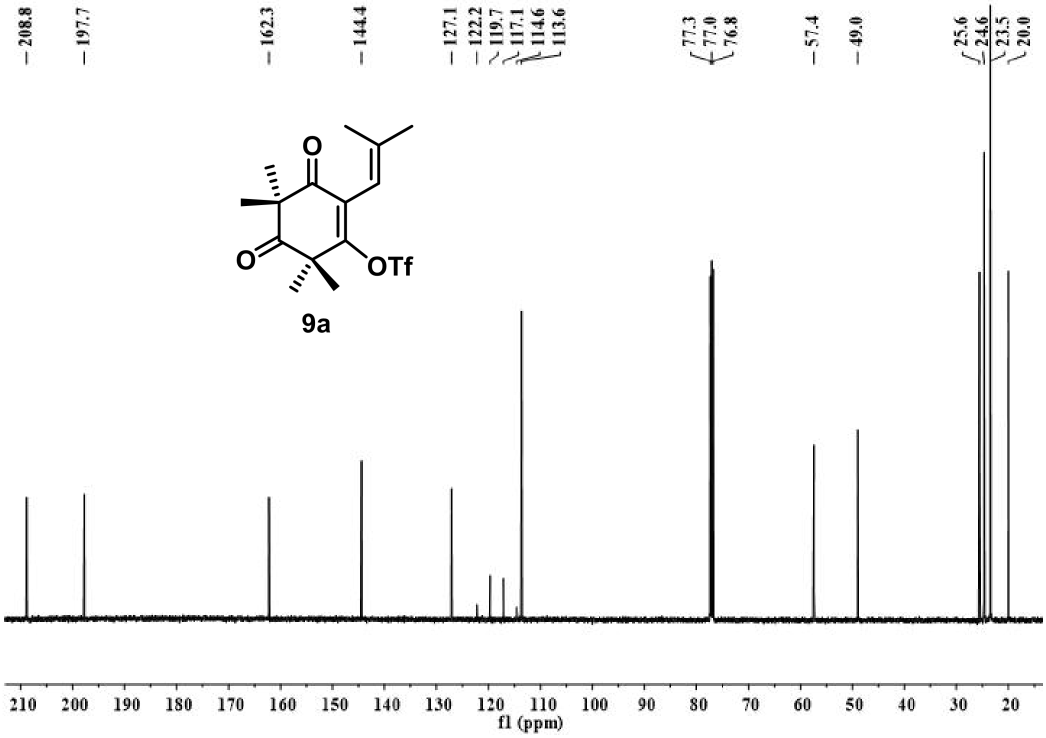
208.8
197.7
162.3
144.4
127.1
122.2
119.7
117.1
114.6
113.6
77.3
77.0
76.8
57.4
49.0
25.6
24.6
23.5
20.0
O
OTf
9a
210 200 190 180 170 160 150 140 130 120 110 100 90 80 70 60 50 40 30 20
f1 (ppm)

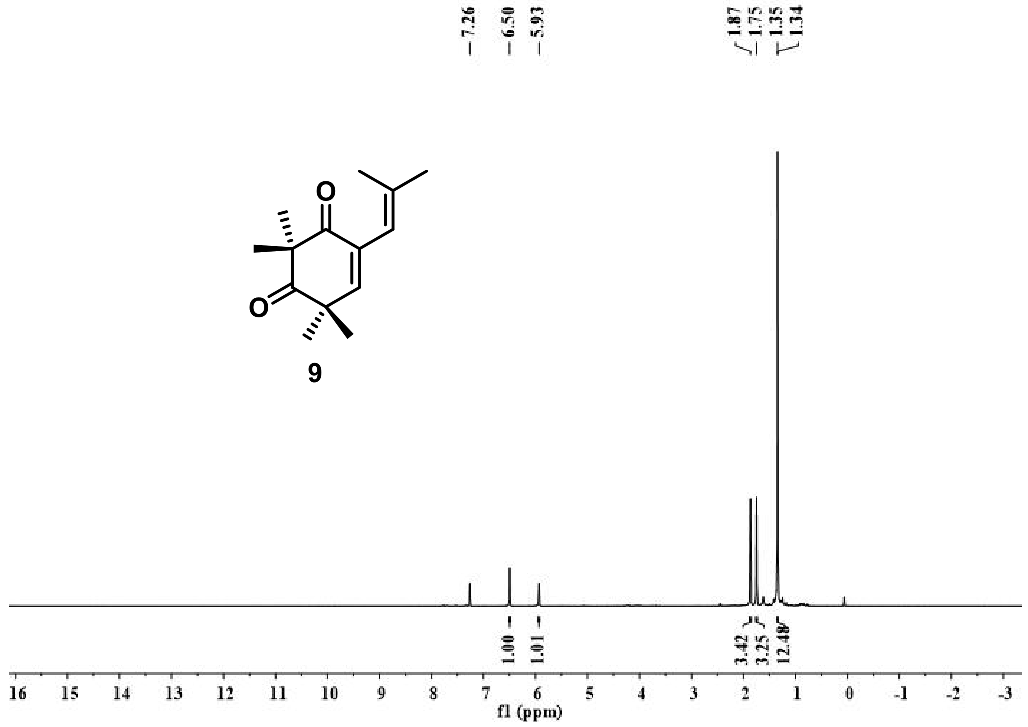
7.26
6.50
5.93
1.87
1.75
1.35
1.34
9
1.00
1.01
3.42
3.25
12.48
16 15 14 13 12 11 10 9 8 7 6 5 4 3 2 1 0 -1 -2 -3
f1 (ppm)

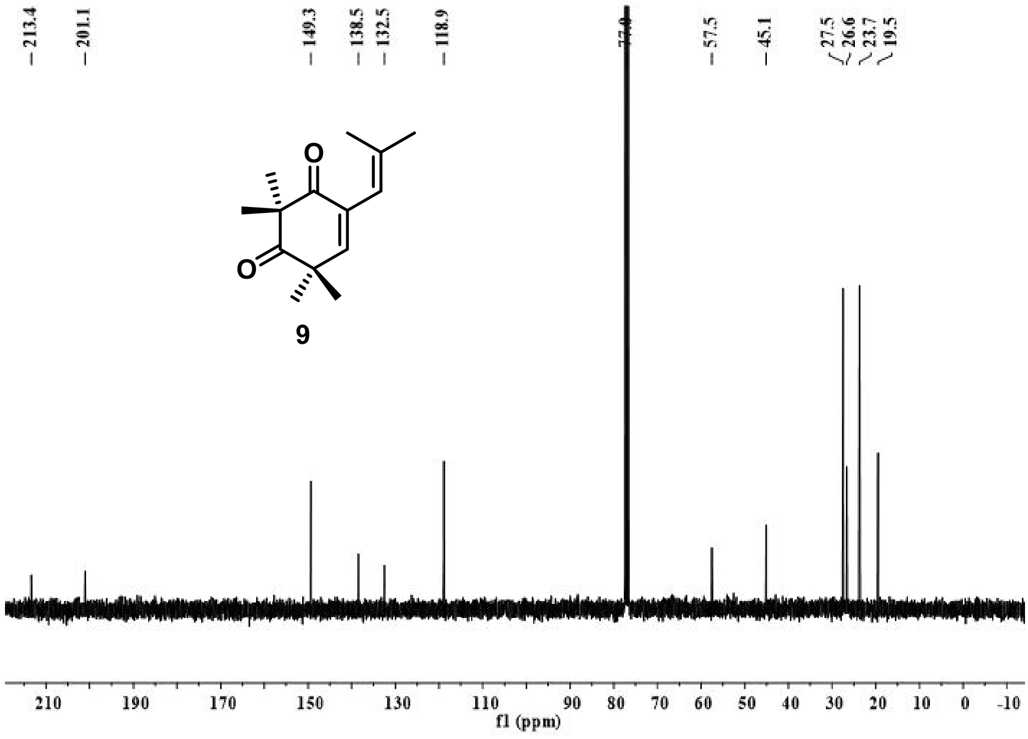
213.4
201.1
149.3
138.5
132.5
118.9
77.0
57.5
45.1
27.5
26.6
23.7
19.5
9
210 190 170 150 130 110 90 80 70 60 50 40 30 20 10 0 -10
f1 (ppm)

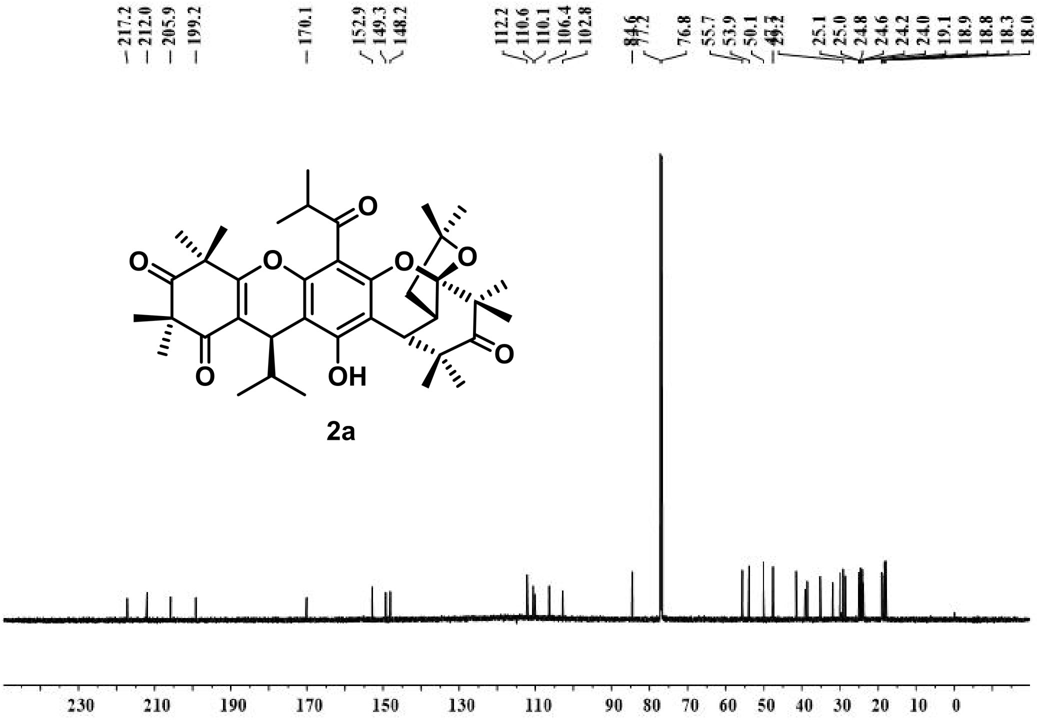
2a

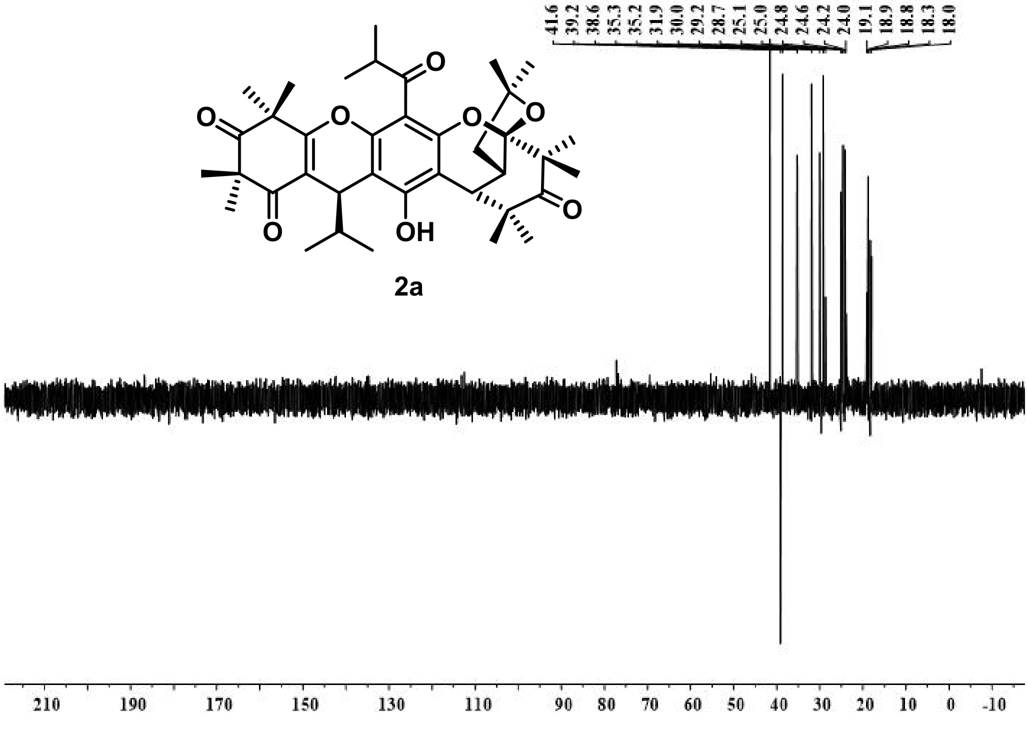
2a

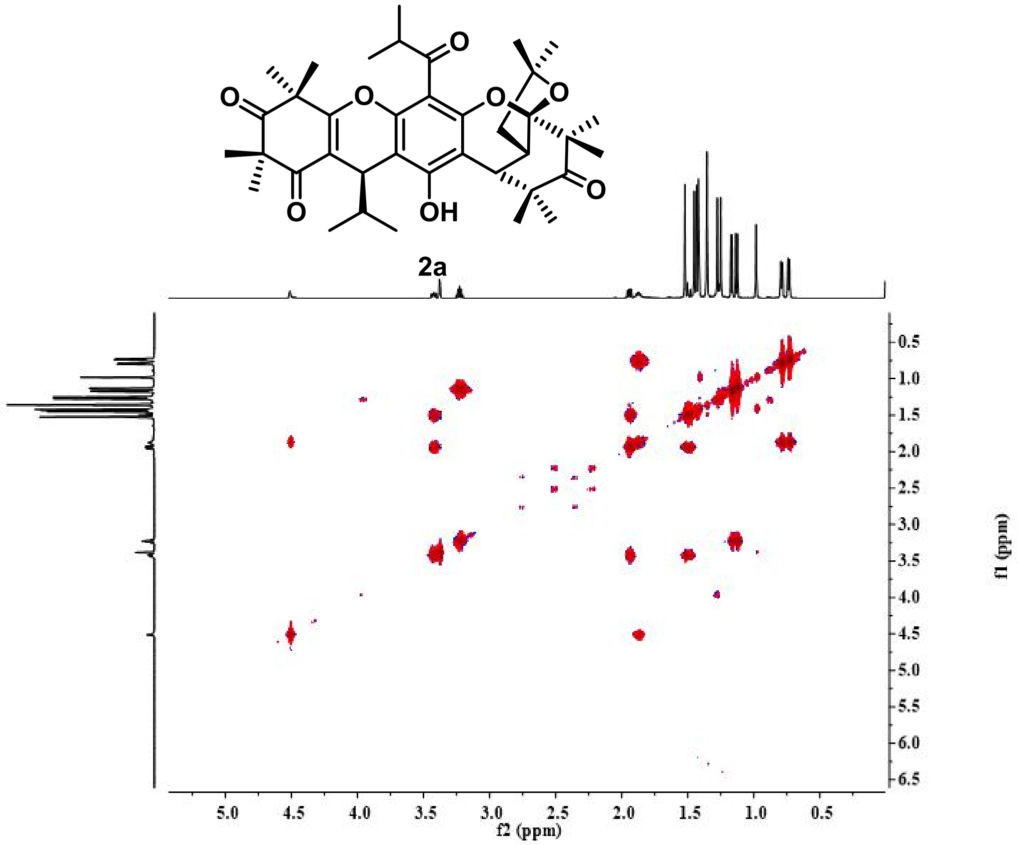


$^{1}H$-$^{1}H$ COSY spectrum of **2a**

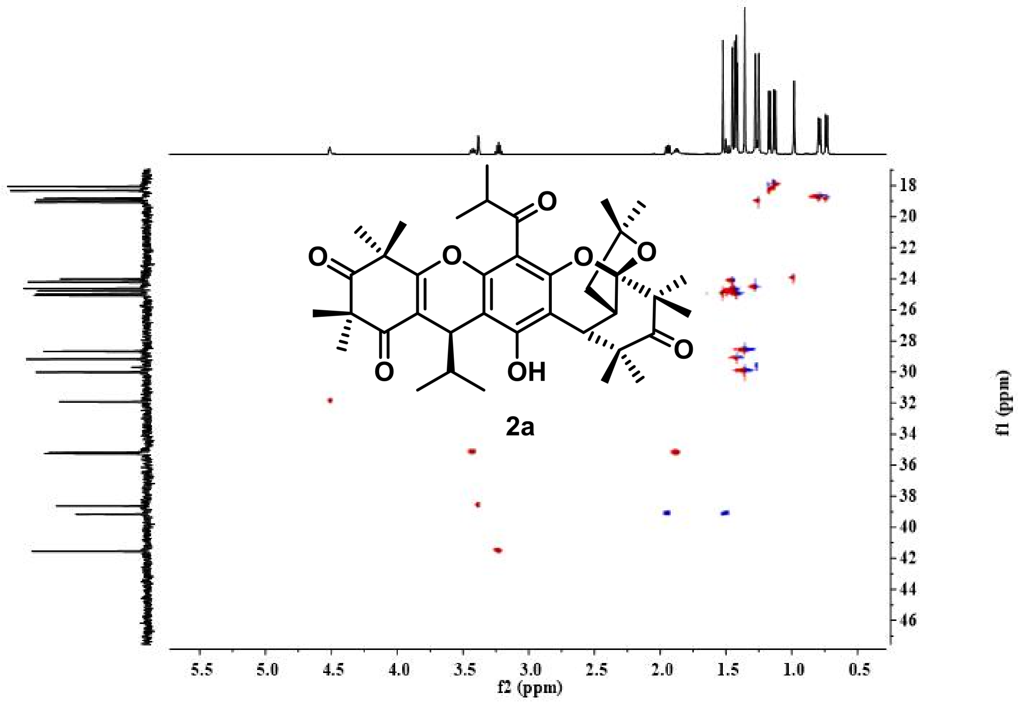


HSQC spectrum of **2a**

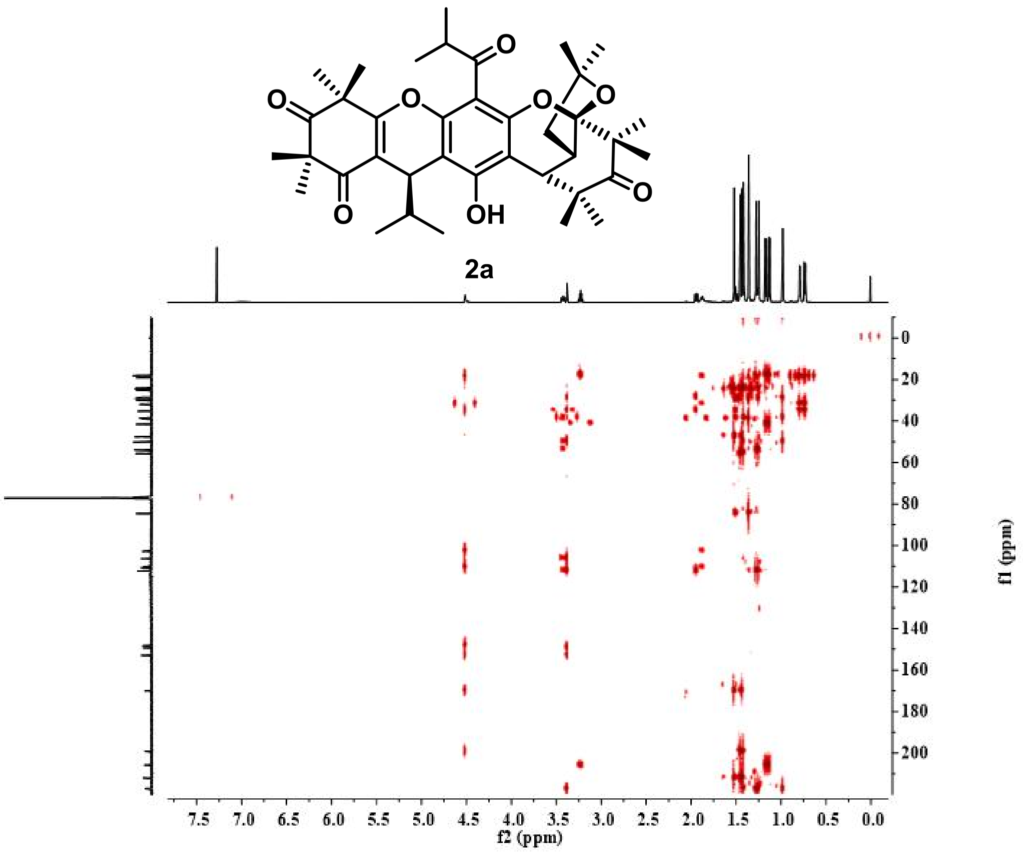


HMBC spectrum of **2a**

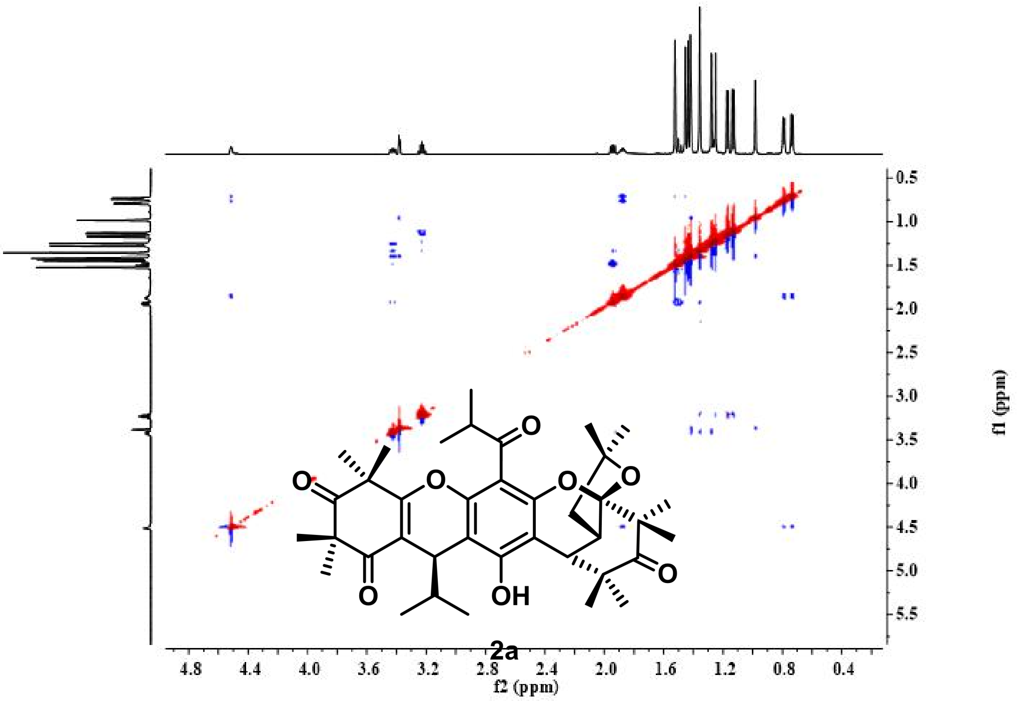


NOESY spectrum of **2a**

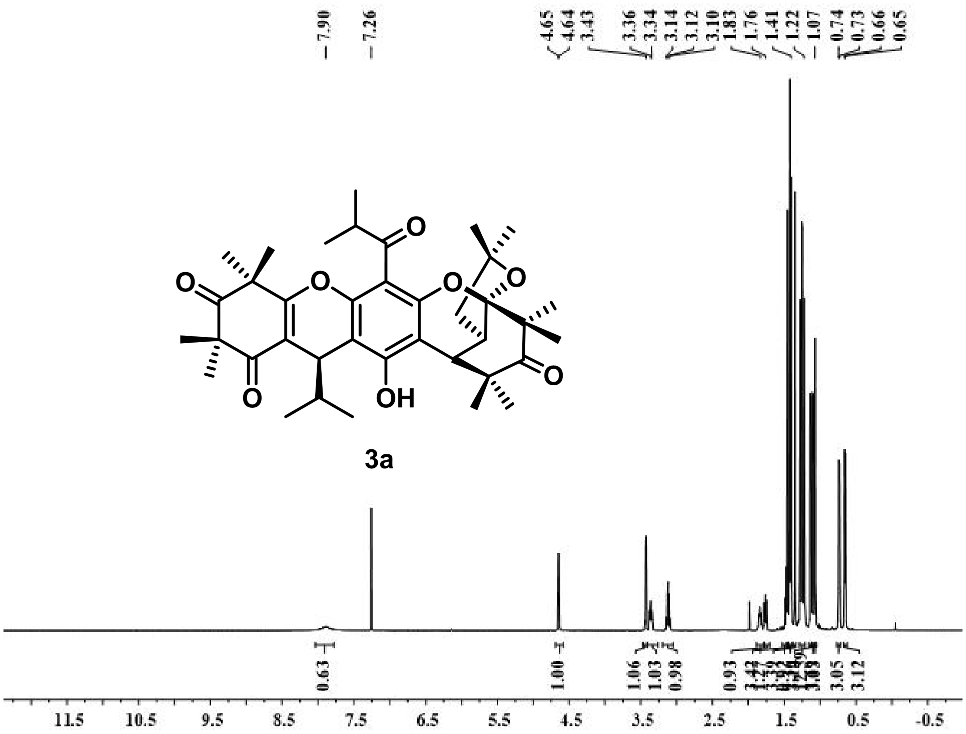
3a
11.5
10.5
9.5
8.5
7.5
6.5
5.5
4.5
3.5
2.5
1.5
0.5
-0.5

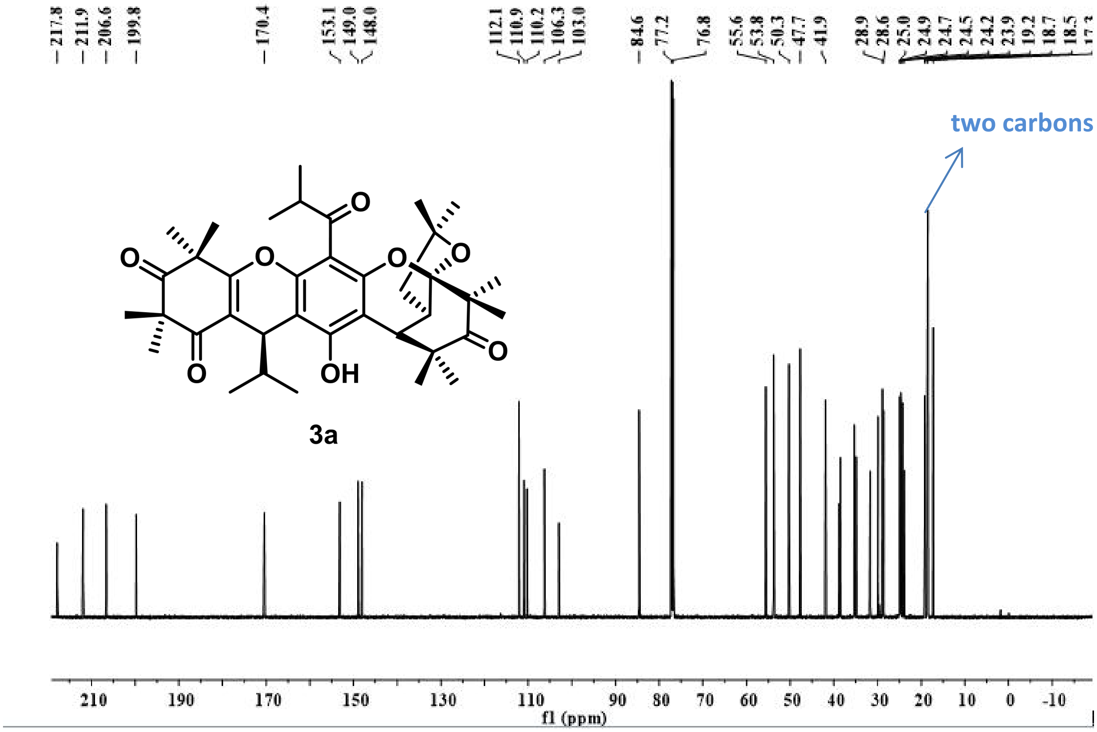
3a
two carbons
210
190
170
150
130
110
90
80
70
60
50
40
30
20
10
0
-10
f1 (ppm)

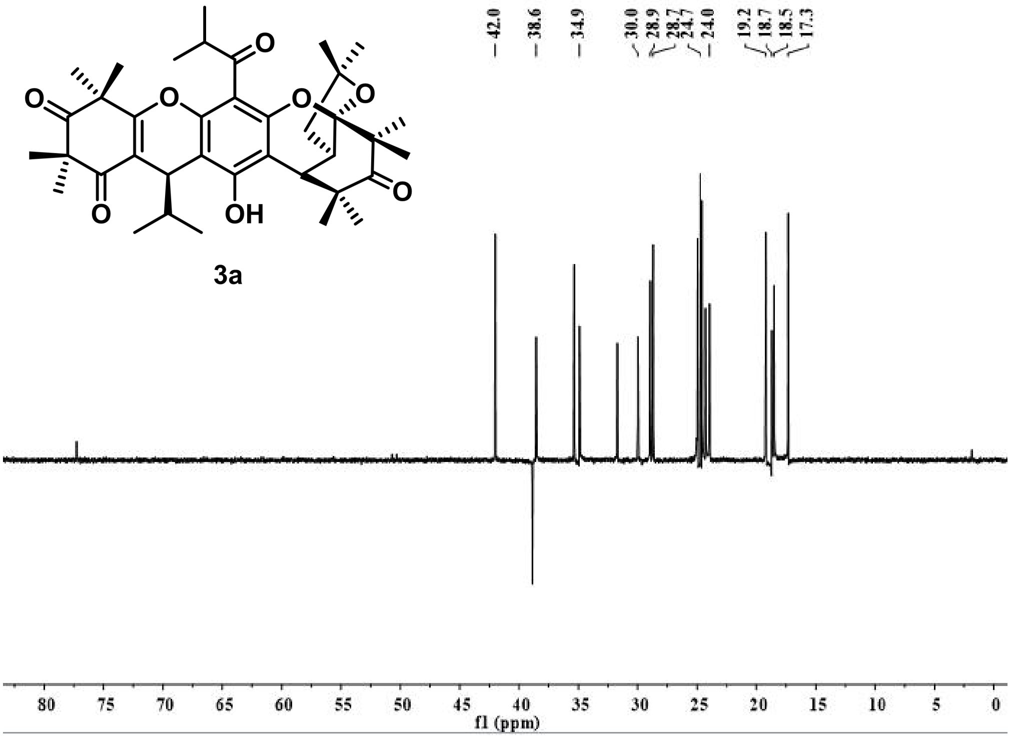


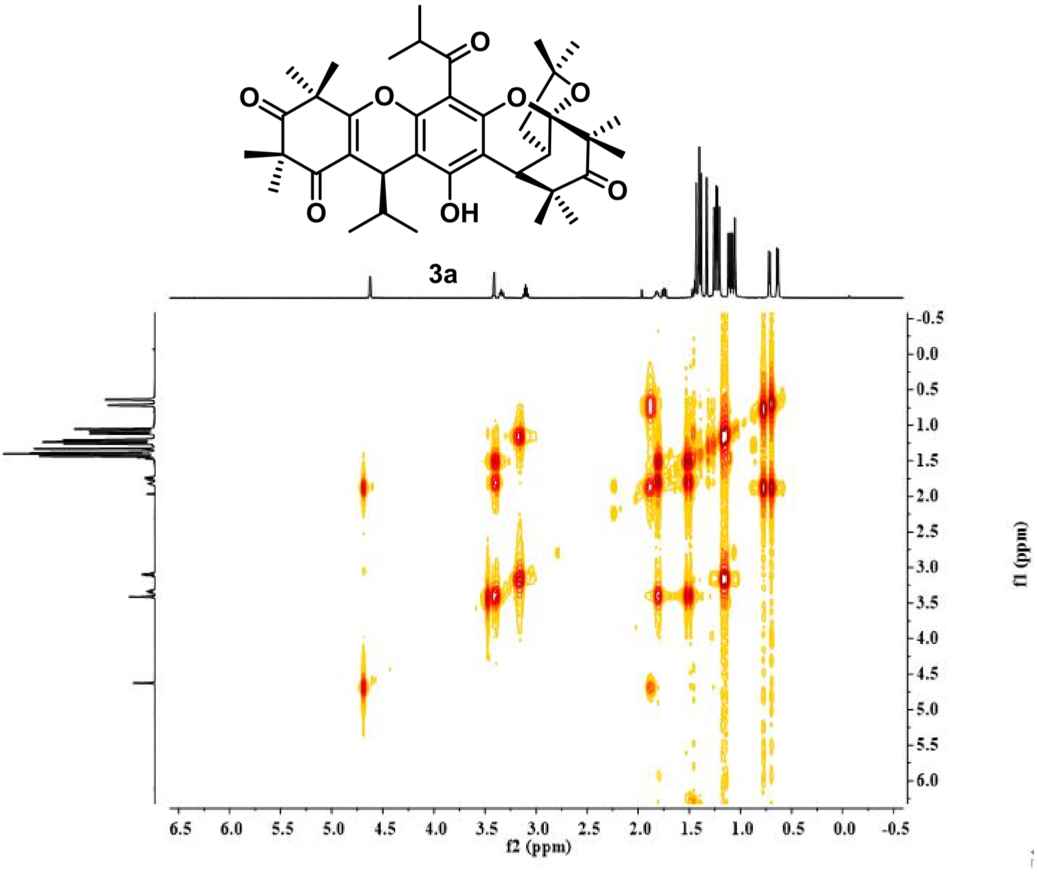


$^{1}H$-$^{1}H$ COSY spectrum of **3a**

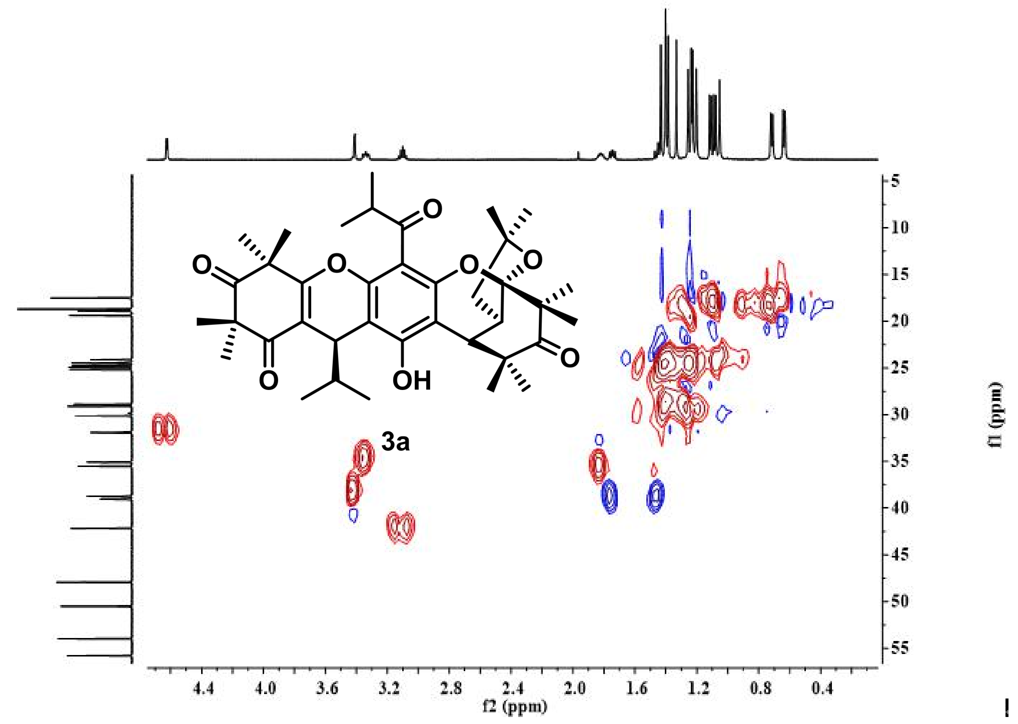

HSQC spectrum of **3a**

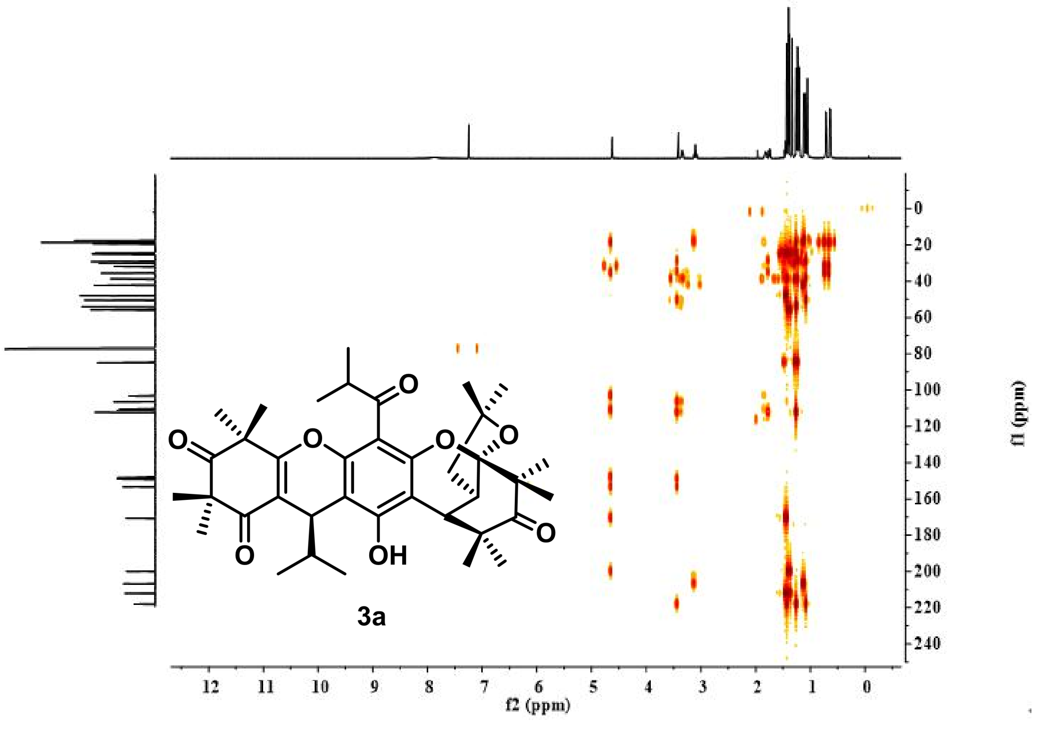

HMBC spectrum of **3a**

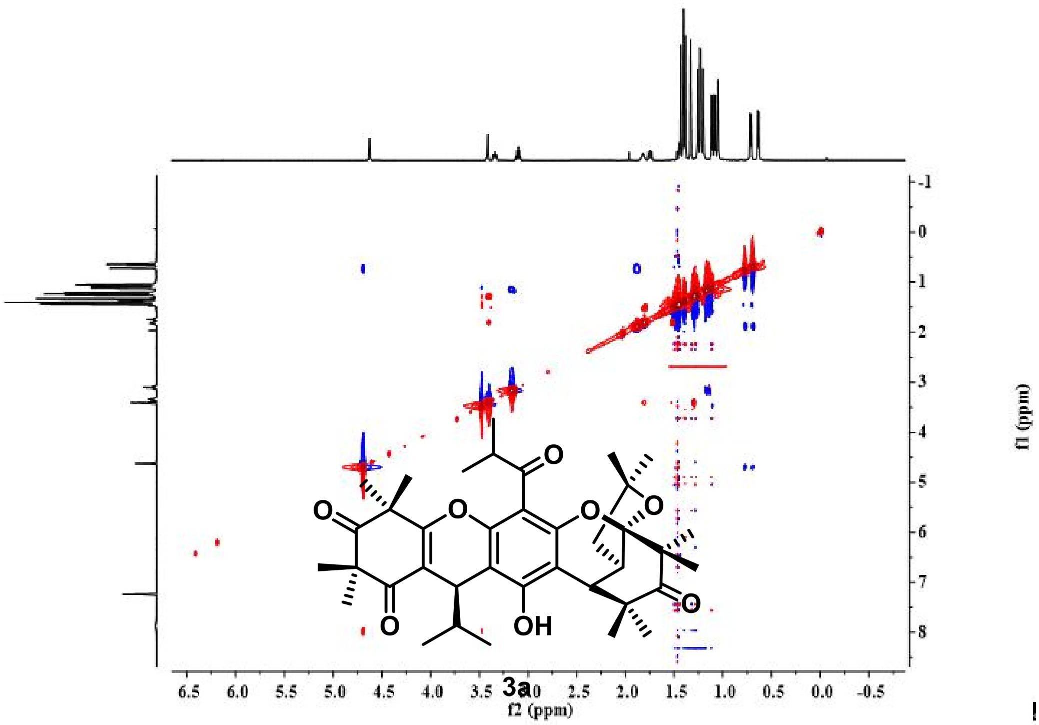

3a

NOESY spectrum of **3a**

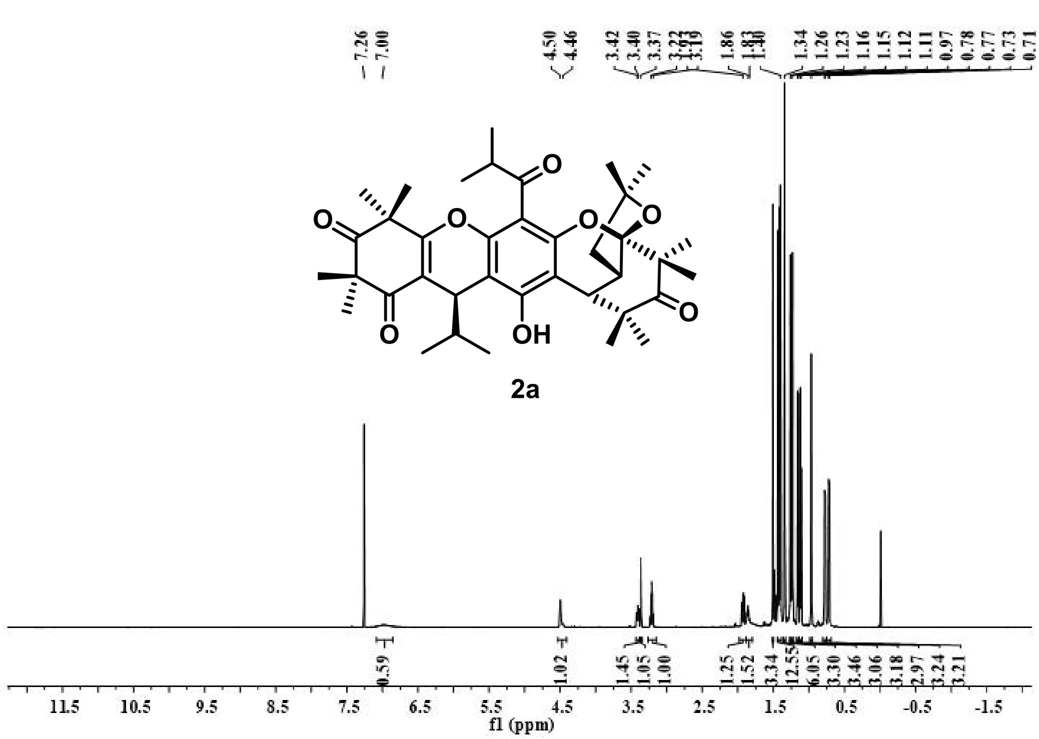

2a

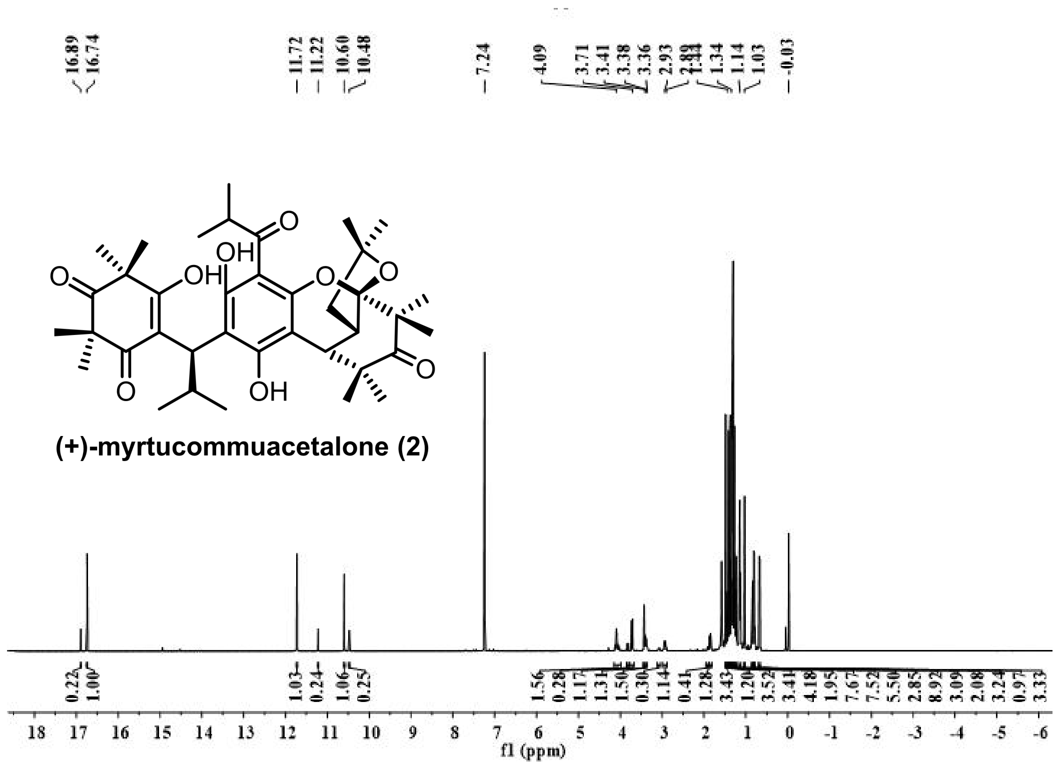
(+)-myrtucommuacetalone (2)
f1 (ppm)

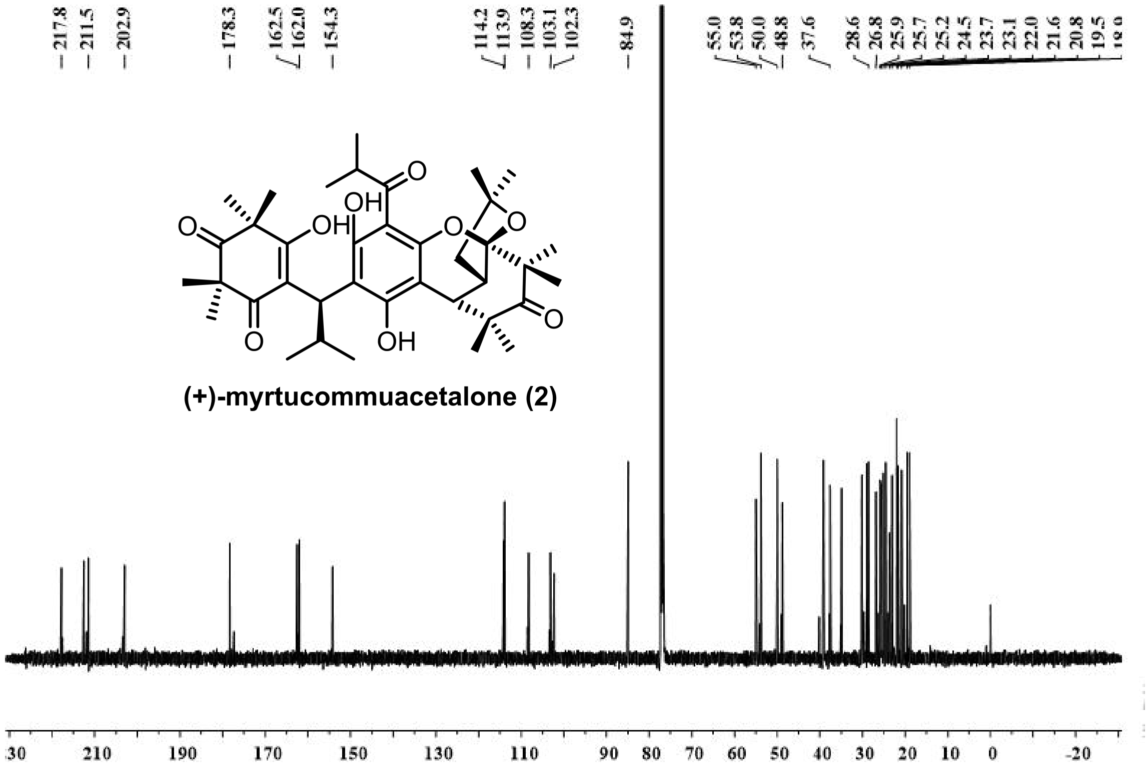
(+)-myrtucommuacetalone (2)

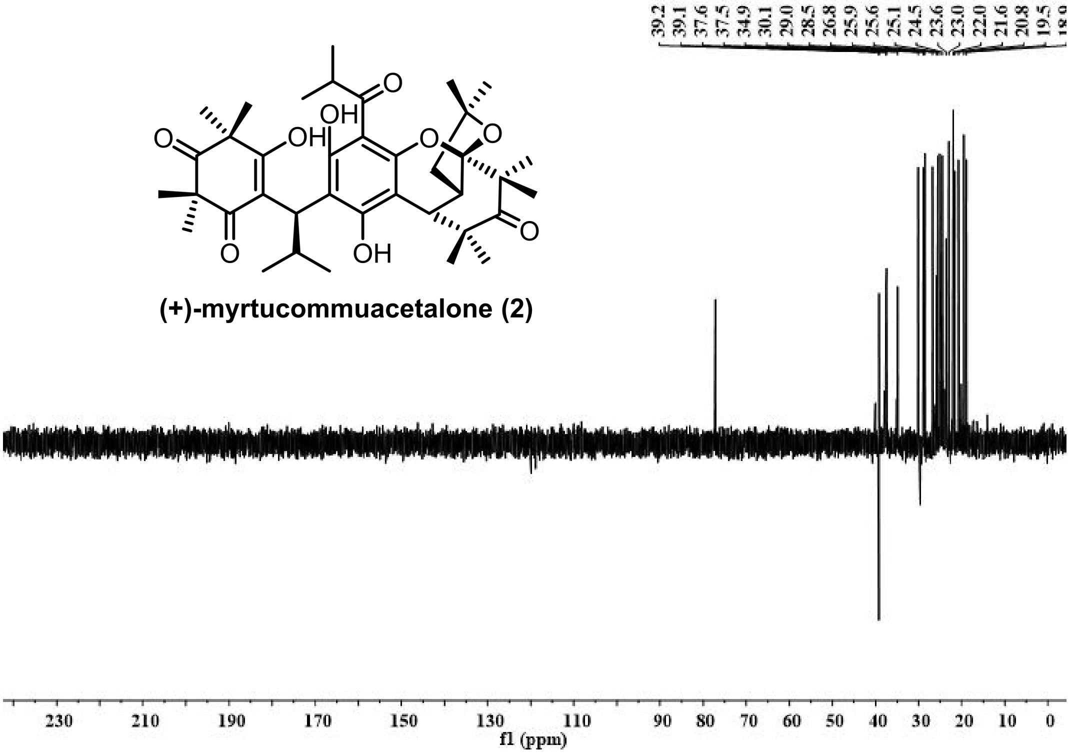


(+)-myrtucommuacetalone (2)

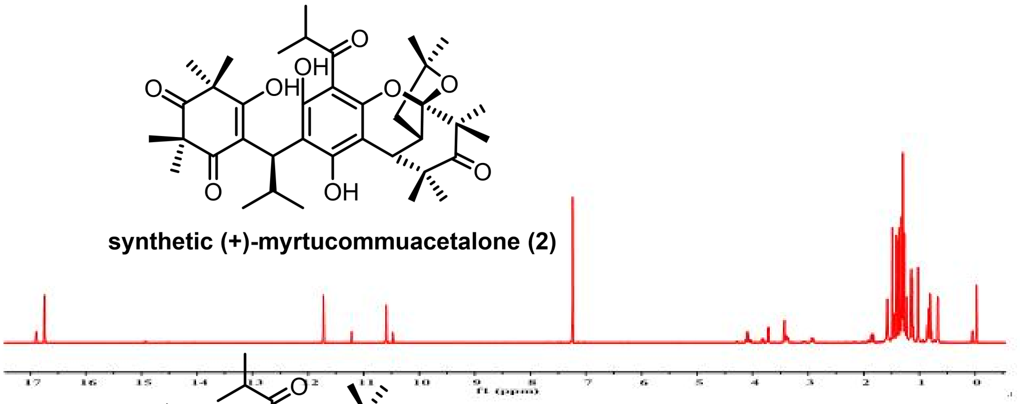

synthetic (+)-myrtucommuacetalone (2)

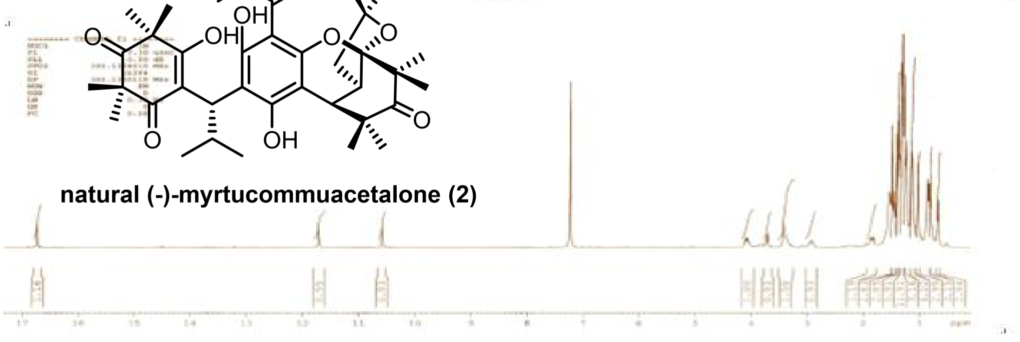

natural (-)-myrtucommuacetalone (2)

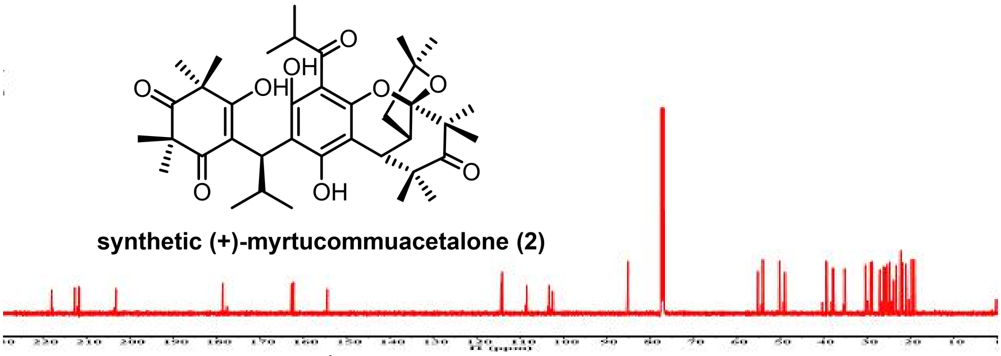

synthetic (+)-myrtucommuacetalone (2)

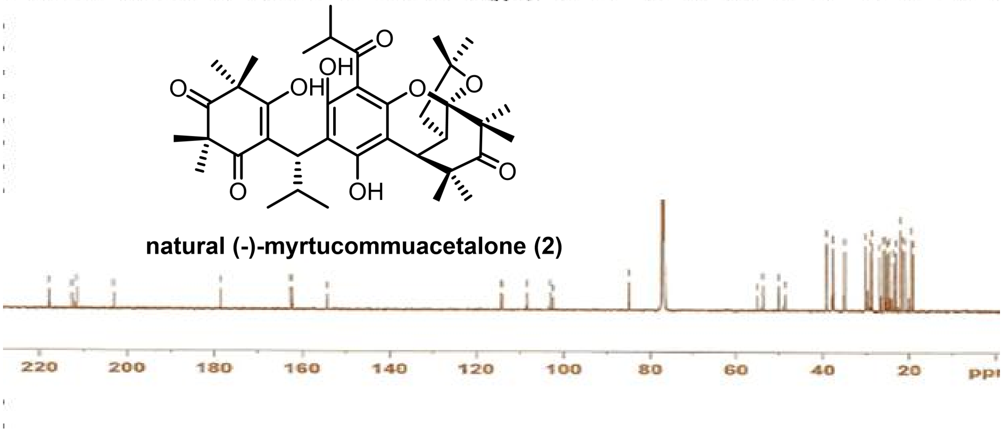

natural (-)-myrtucommuacetalone (2)

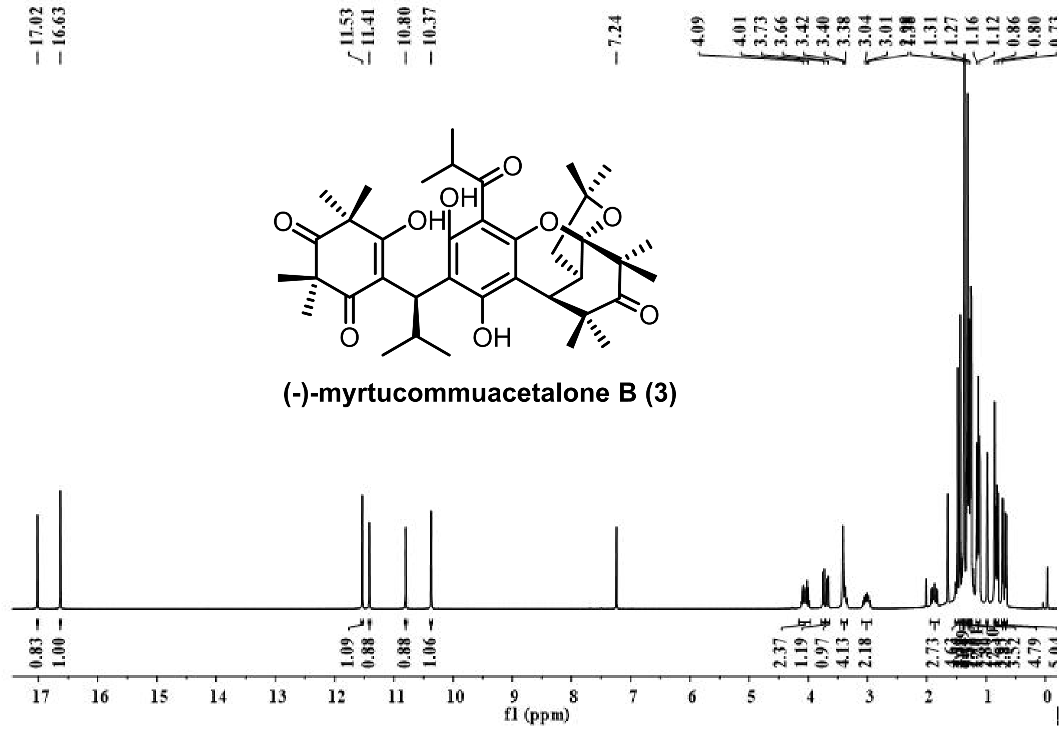


(-)-myrtucommuacetalone B (3)

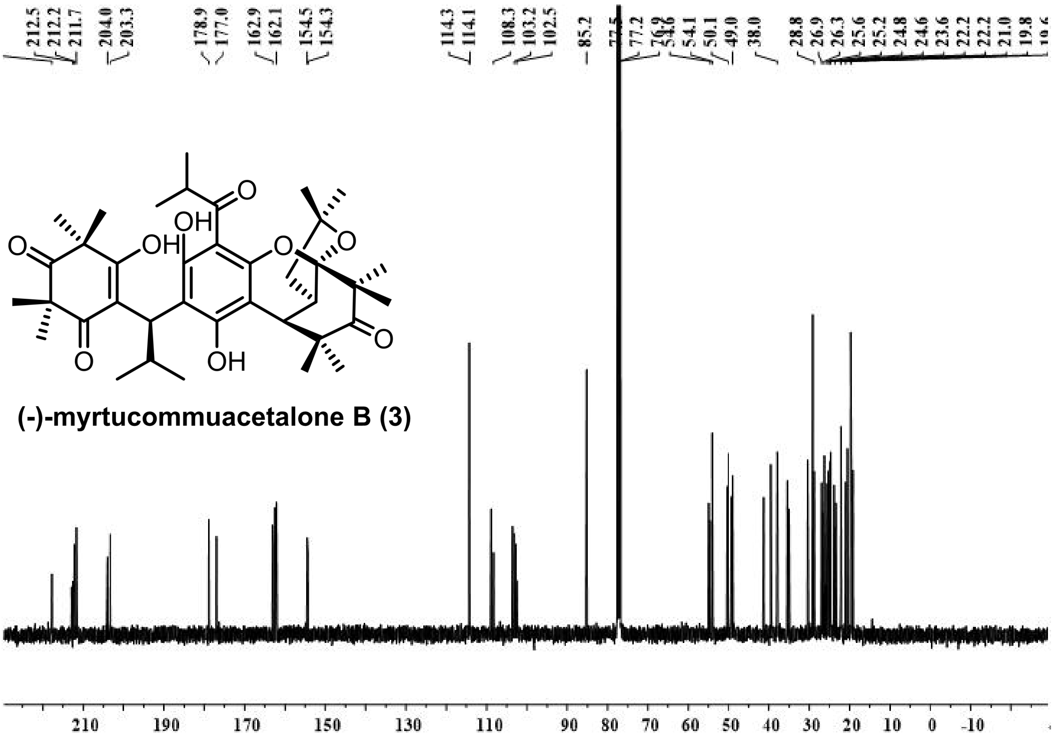
212.5
212.2
211.7
204.0
203.3
178.9
177.0
162.9
162.1
154.5
154.3
114.3
114.1
108.3
103.2
102.5
85.2
77.5
77.2
76.9
54.6
54.1
50.1
49.0
38.0
28.8
26.9
26.3
25.6
25.2
24.8
24.6
23.6
22.2
22.2
21.0
19.8
[illegible]
O
OH
OH
O
O
O
O
OH
O
(-)-myrtucommuacetalone B (3)

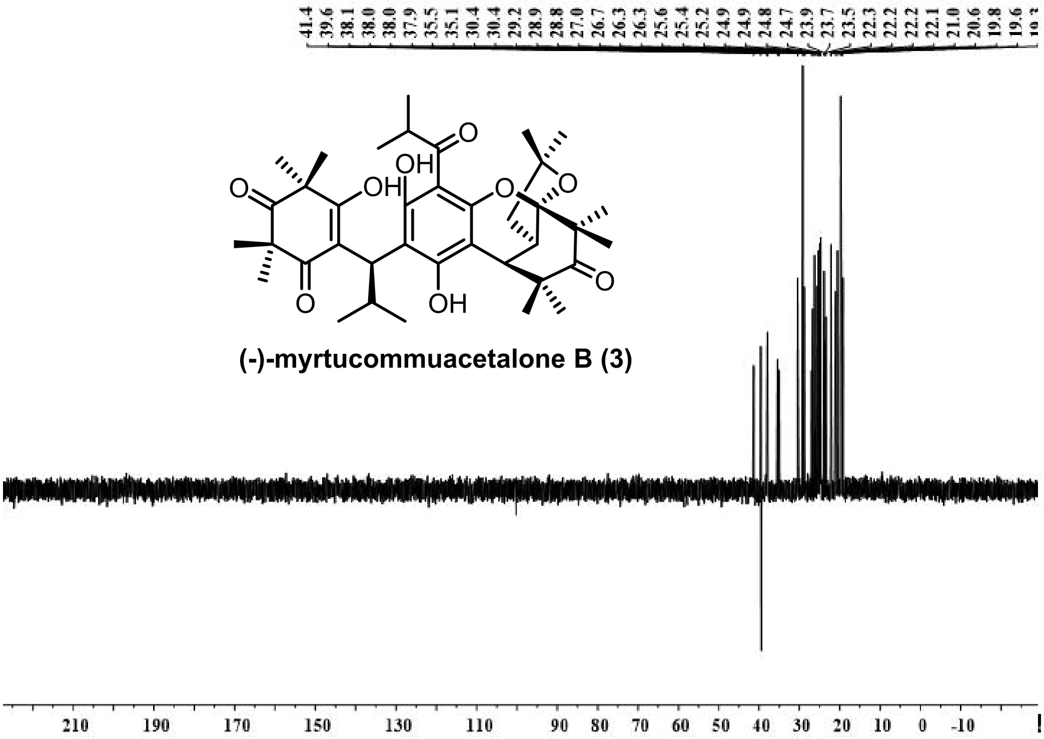
41.4
39.6
38.1
38.0
38.0
37.9
35.5
35.1
30.4
30.4
29.2
28.9
28.8
27.0
26.7
26.3
26.3
25.6
25.4
25.2
24.9
24.9
24.8
24.7
23.9
23.7
23.5
22.3
22.2
22.2
22.1
21.0
20.6
19.8
19.6
[illegible]
O
OH
OH
O
O
O
O
OH
O
(-)-myrtucommuacetalone B (3)

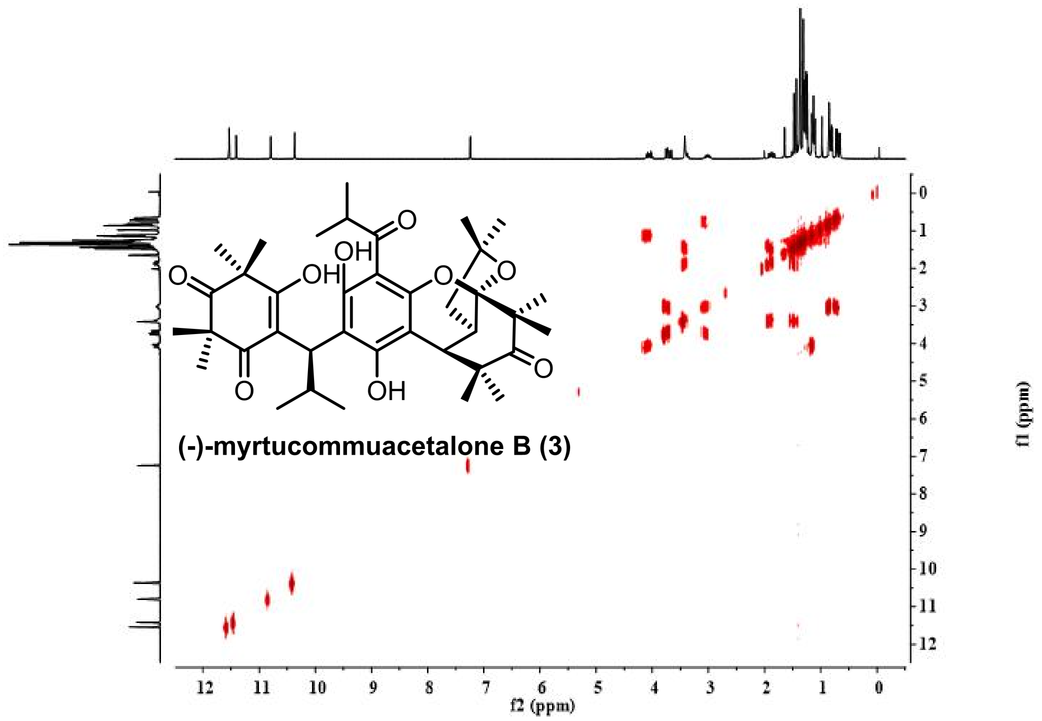


$^{1}$H-$^{1}$H COSY spectrum of (-)-myrtucommuacetalone B (**3**)

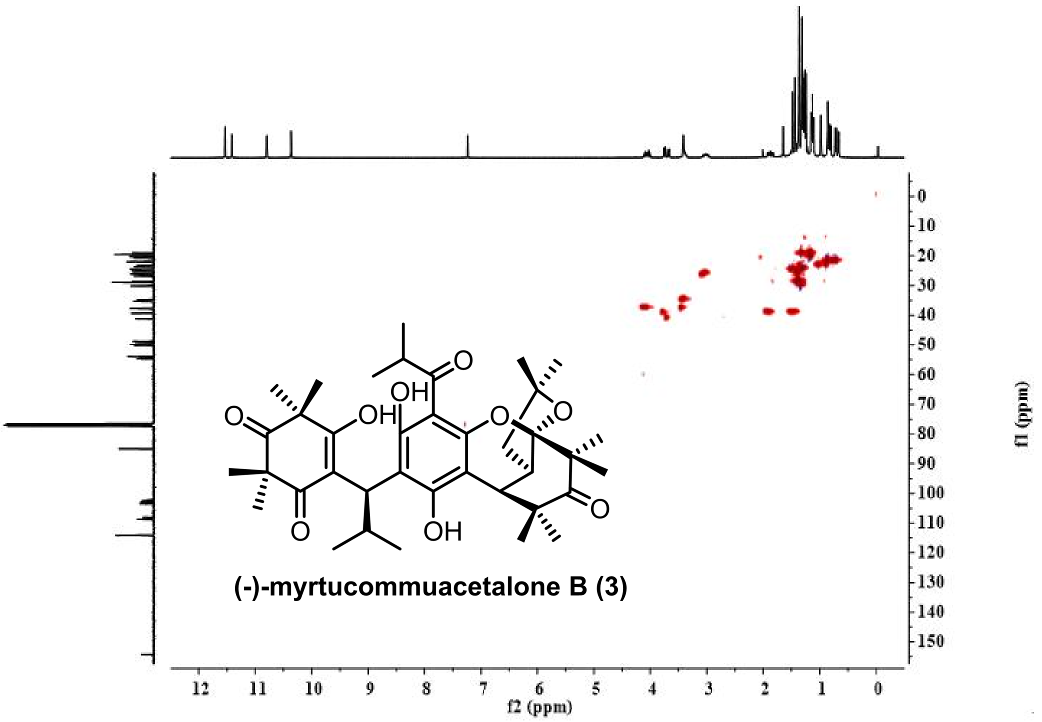


HSQC spectrum of (-)-myrtucommuacetalone B (**3**)

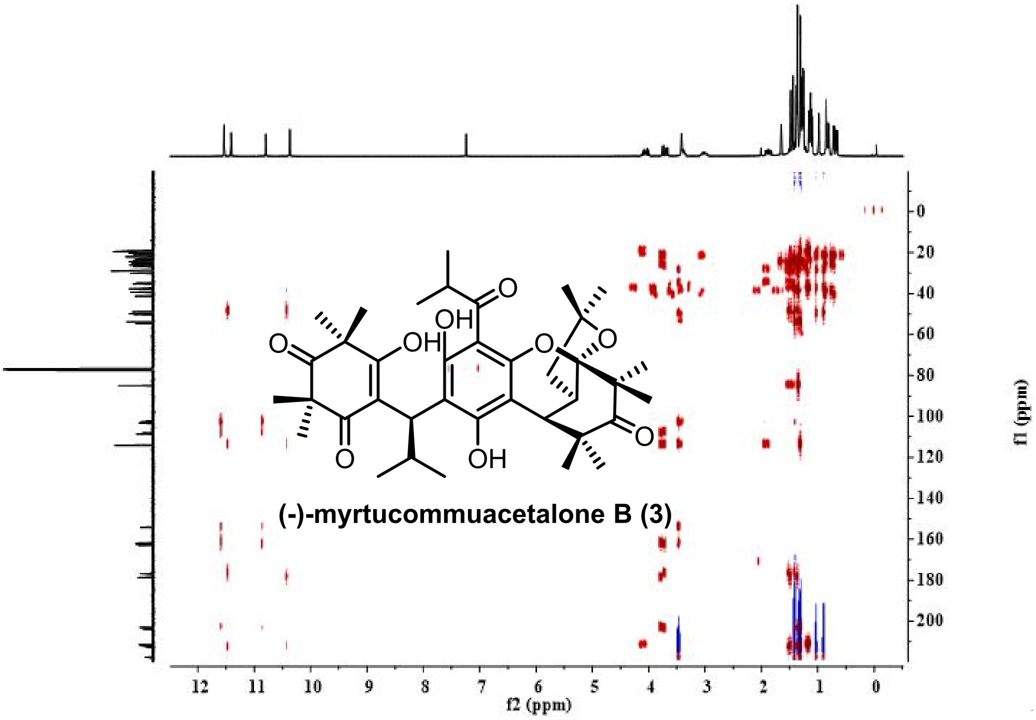


HMBC spectrum of (-)-myrtucommuacetalone B (**3**)

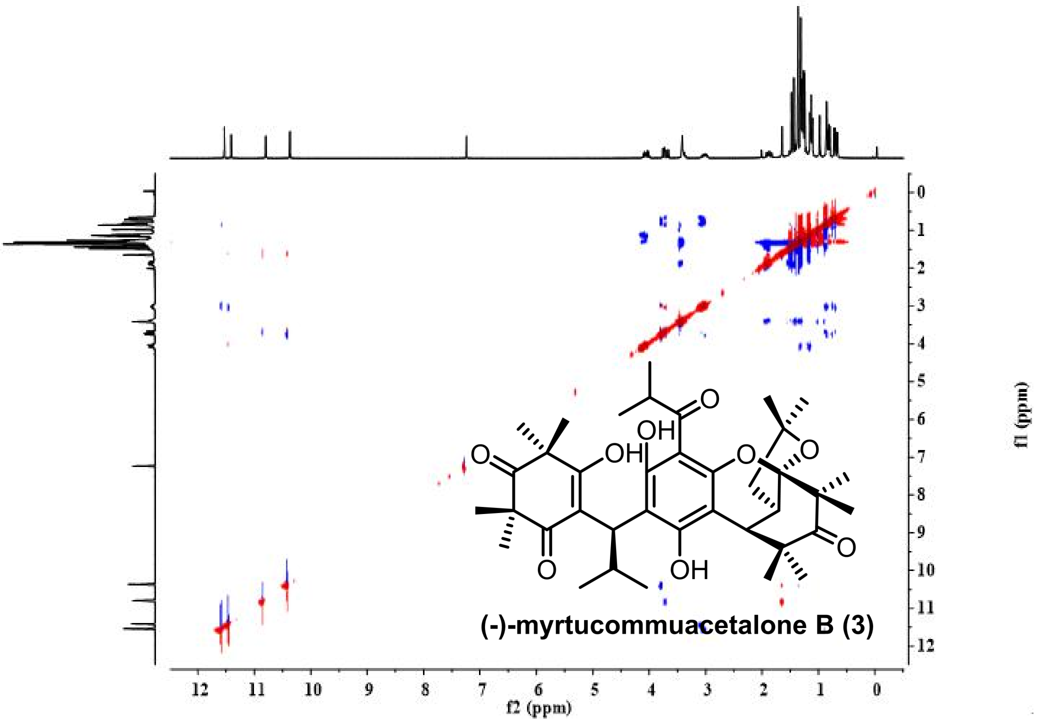


NOESY spectrum of (-)-myrtucommuacetalone B (**3**)

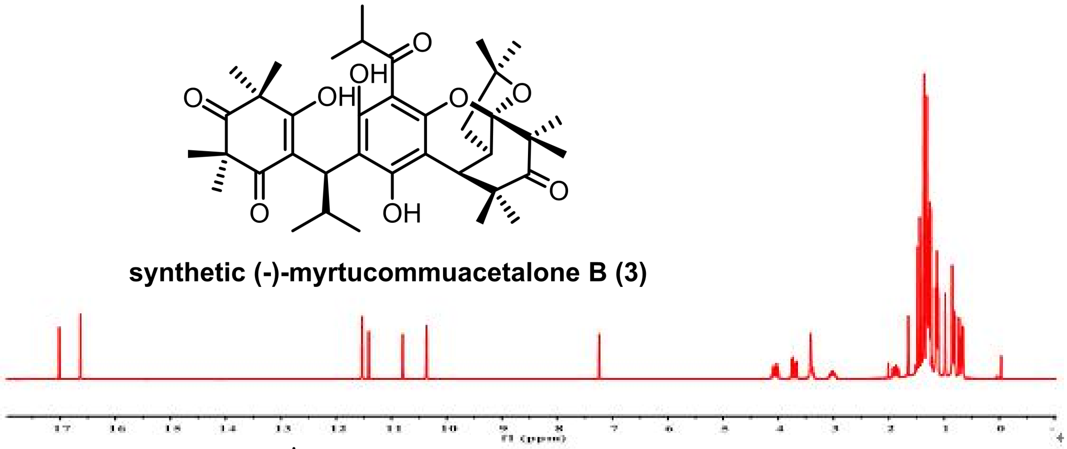

**synthetic (-)-myrtucommuacetalone B (3)**

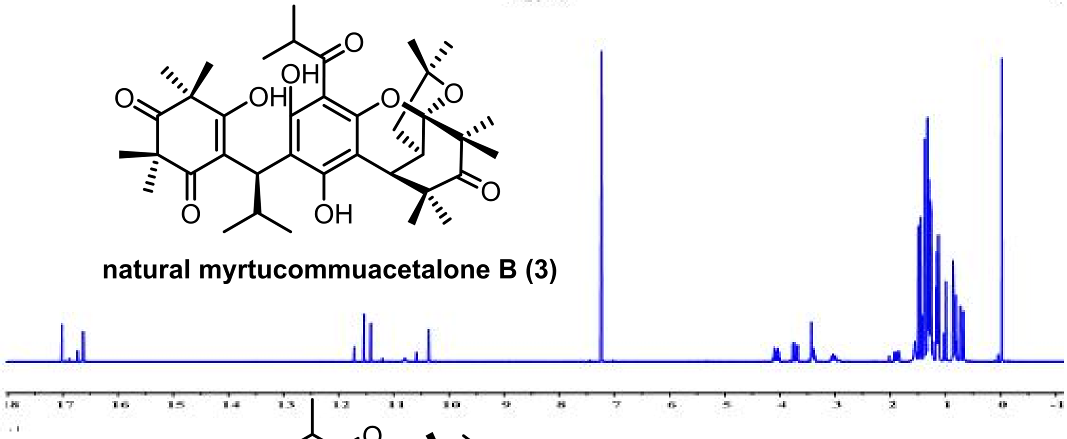

**natural myrtucommuacetalone B (3)**

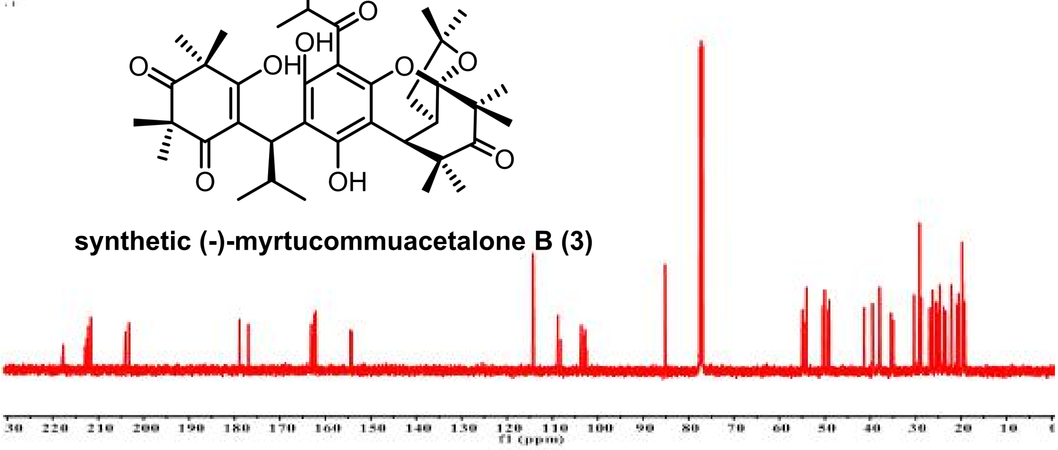

**synthetic (-)-myrtucommuacetalone B (3)**

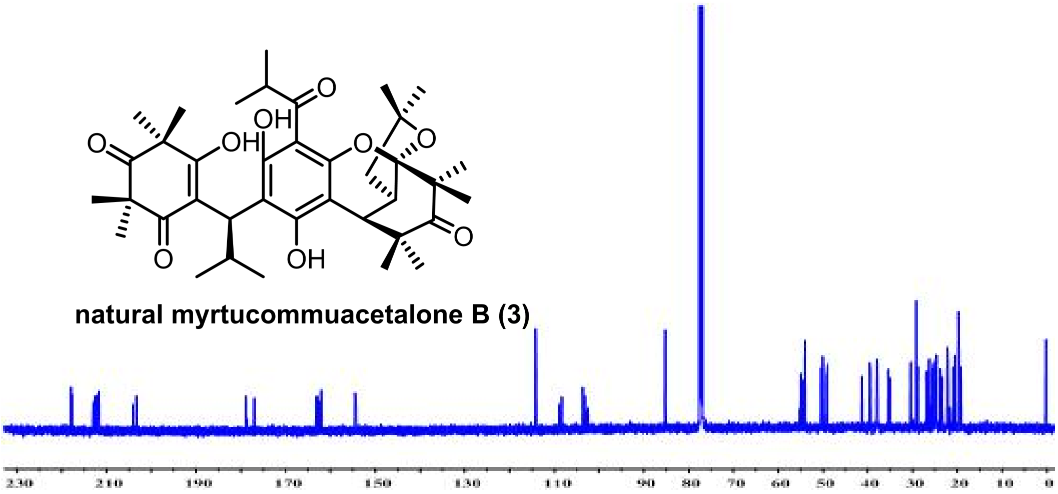

**natural myrtucommuacetalone B (3)**

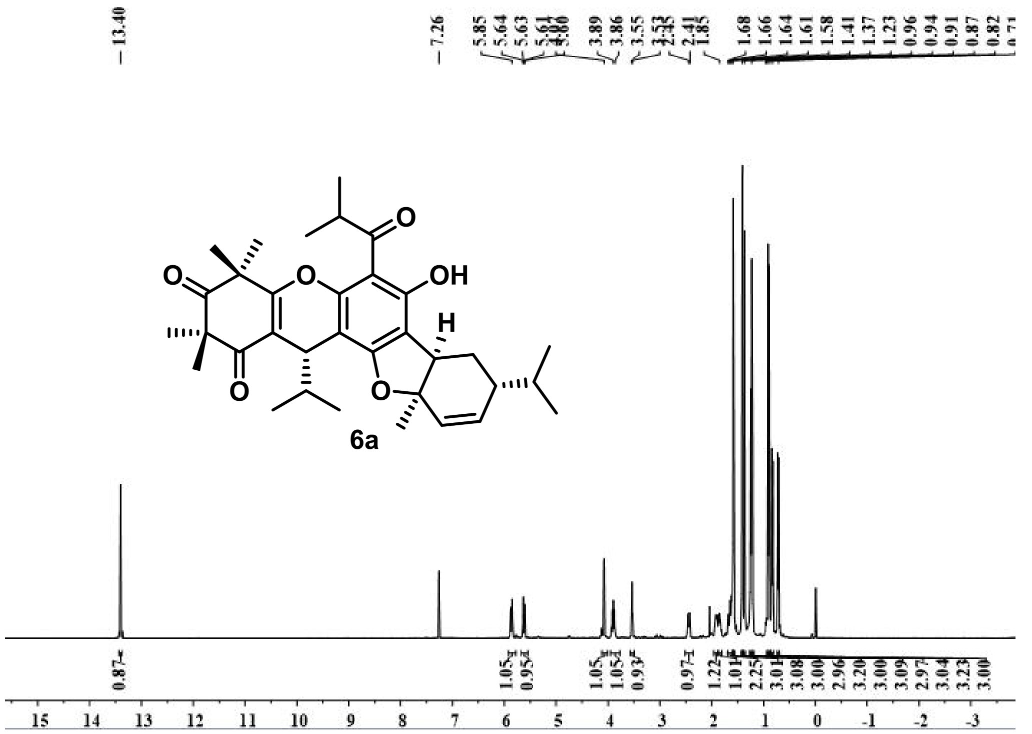
6a

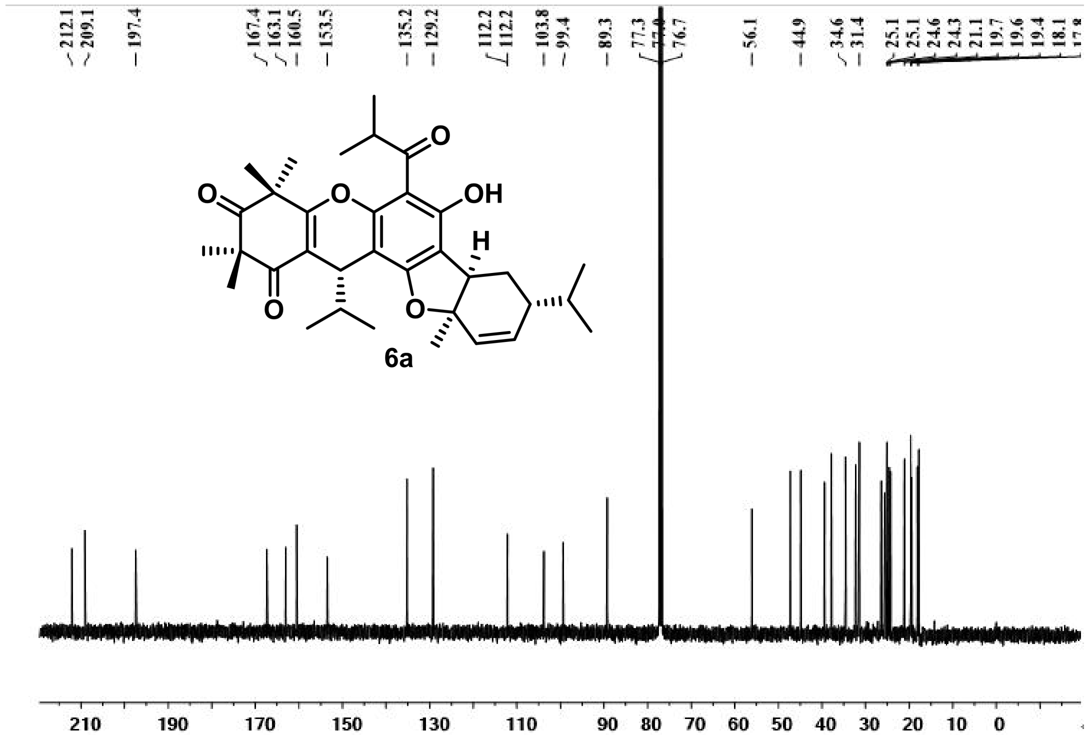
6a

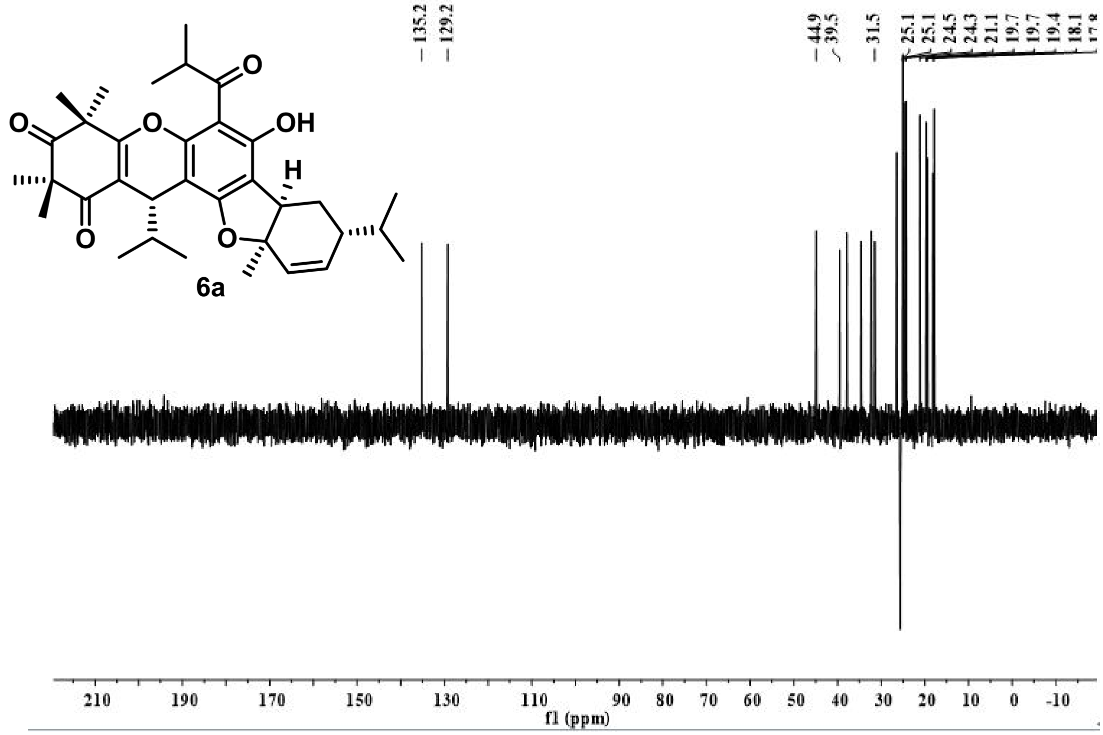

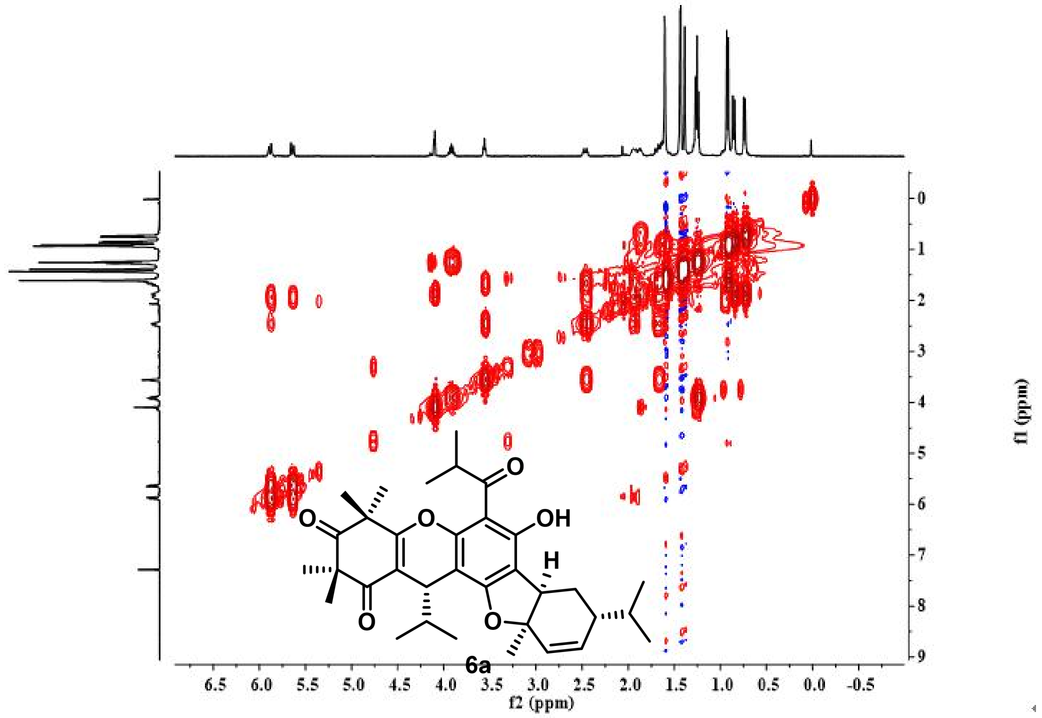

$^{1}$H-$^{1}$H COSY spectrum of **6a**

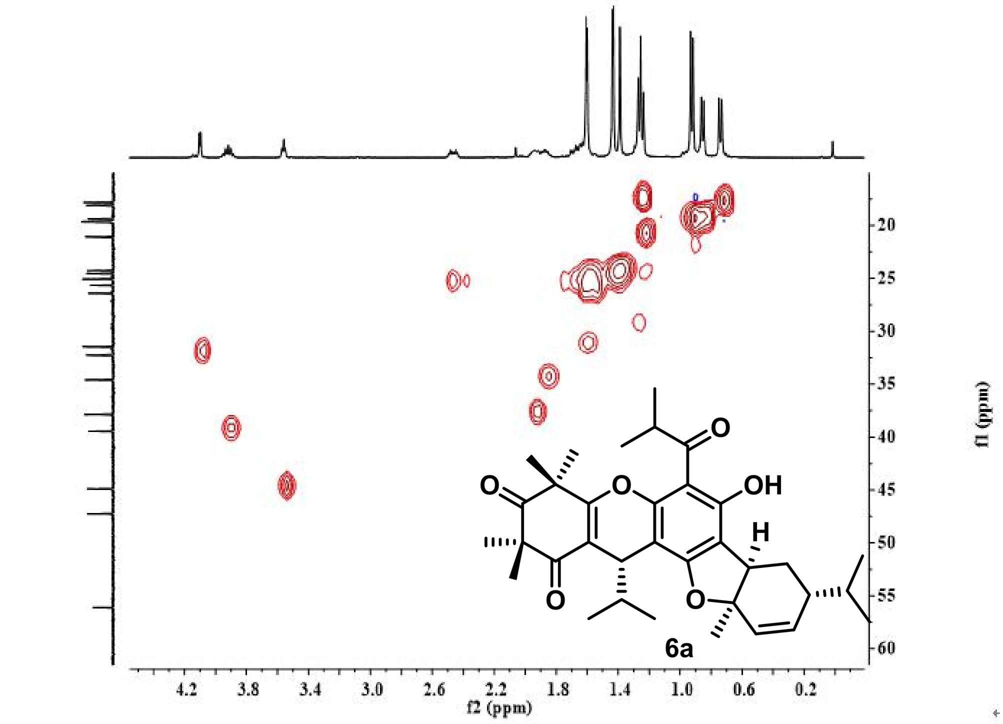

HSQC spectrum of **6a**

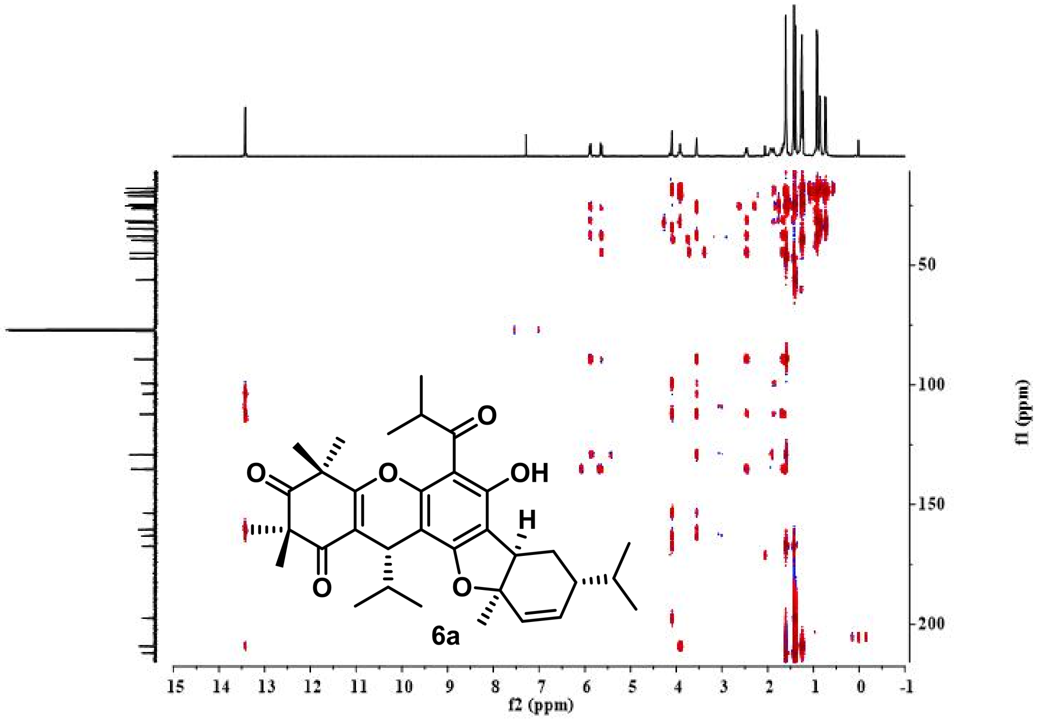

HMBC spectrum of **6a**

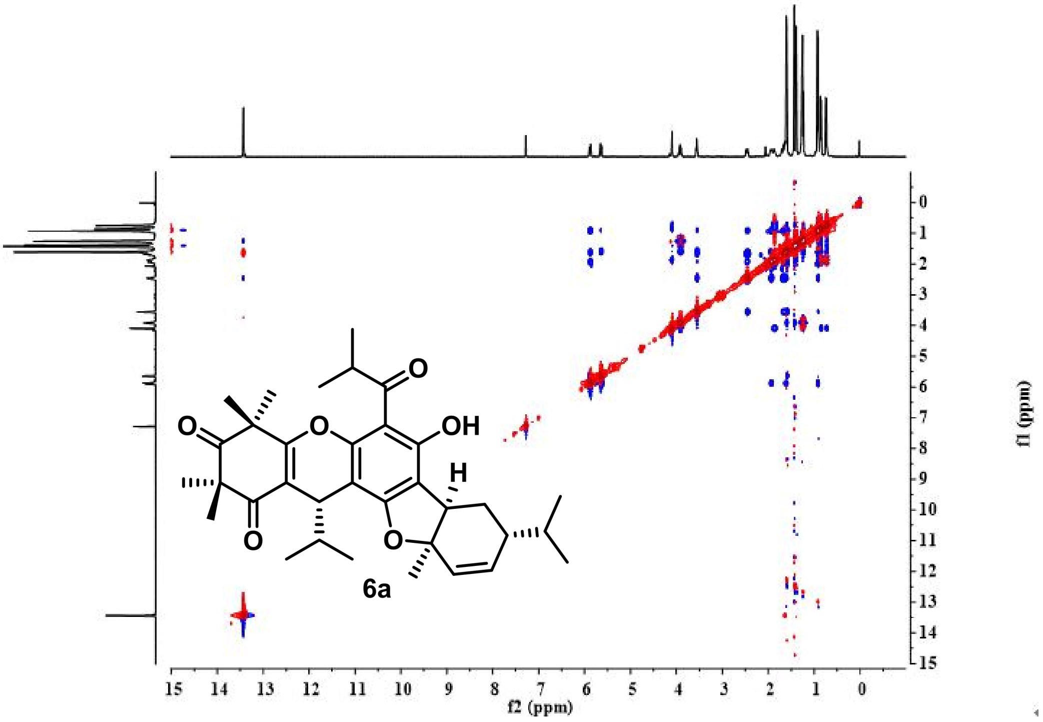


NOESY spectrum of **6a**

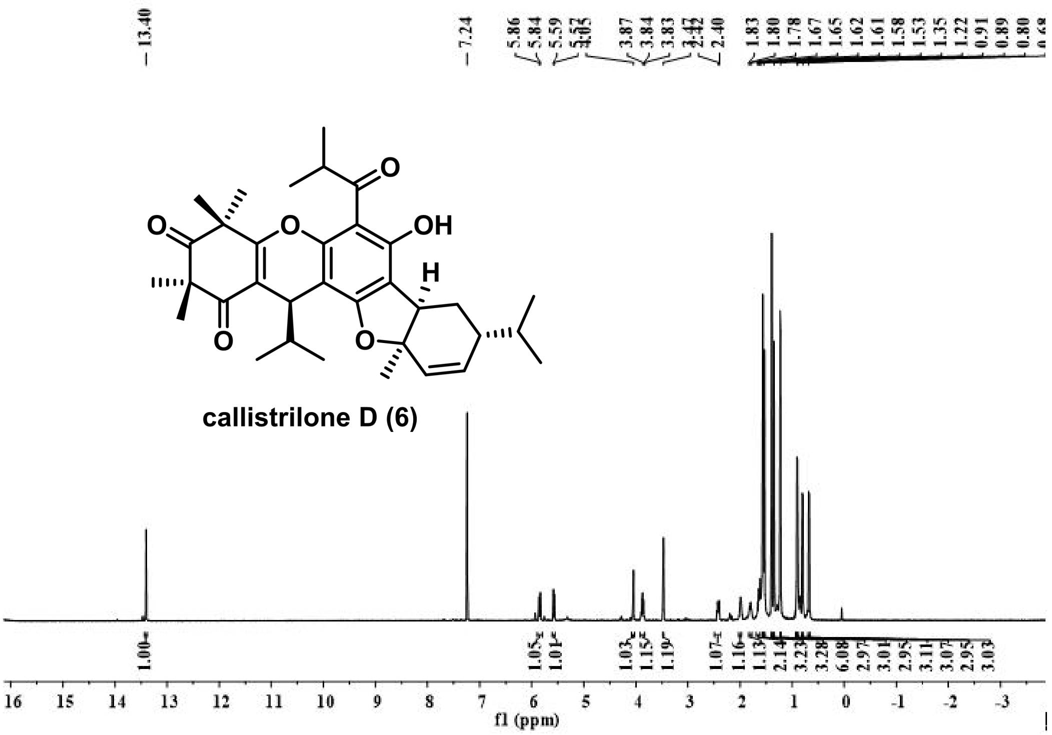

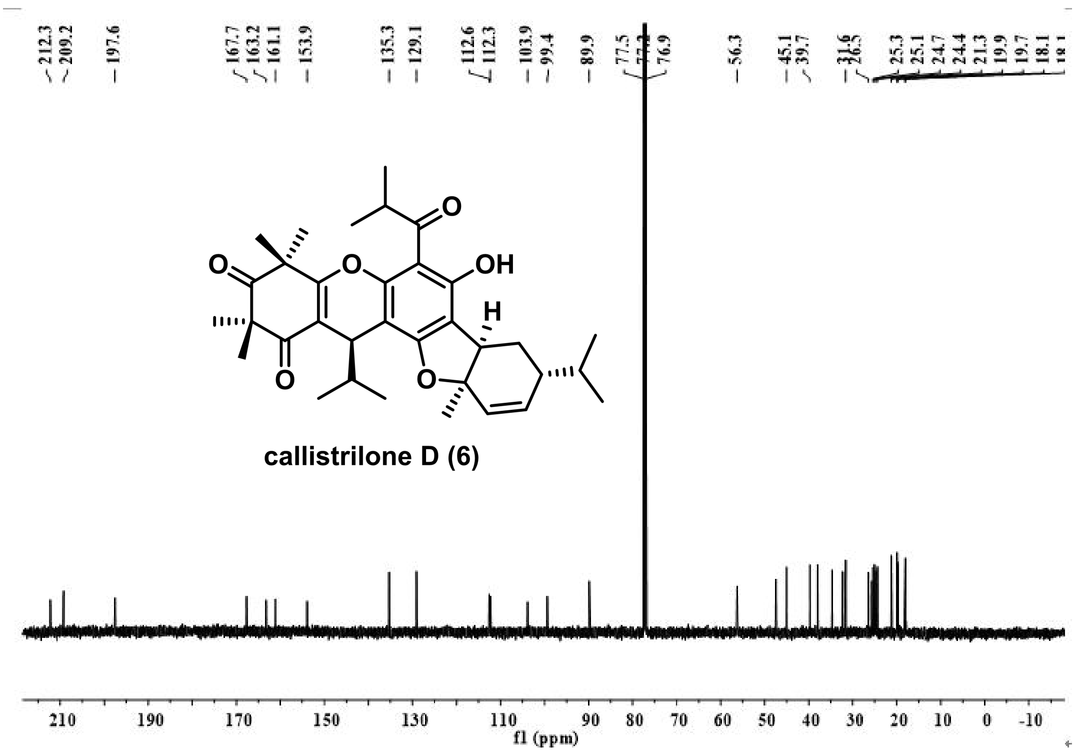
212.3
209.2
197.6
167.7
163.2
161.1
153.9
135.3
129.1
112.6
112.3
103.9
99.4
89.9
77.5
76.9
56.3
45.1
39.7
31.6
26.5
25.3
25.1
24.7
24.4
21.3
19.9
19.7
18.1
callistrilone D (6)
f1 (ppm)

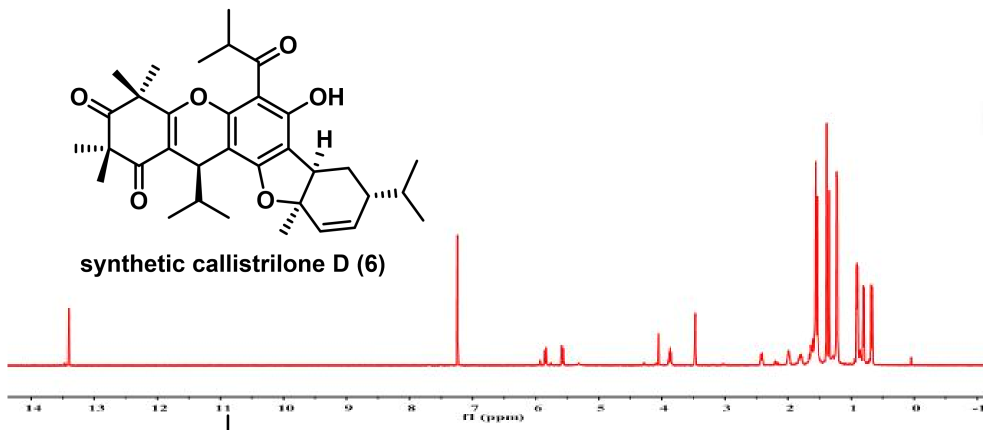
synthetic callistrilone D (6)
f1 (ppm)

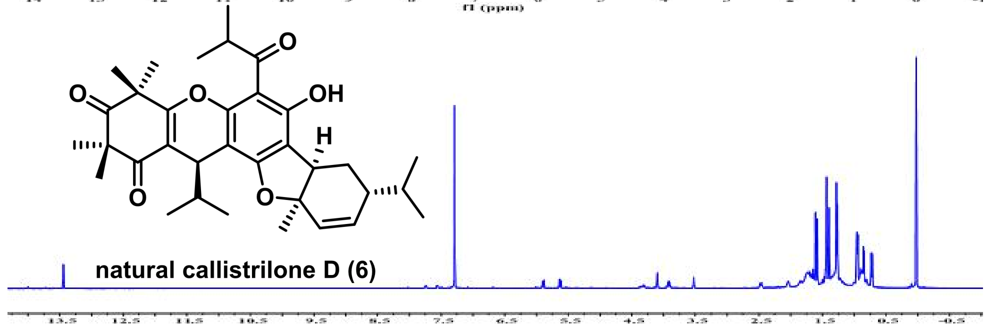
natural callistrilone D (6)

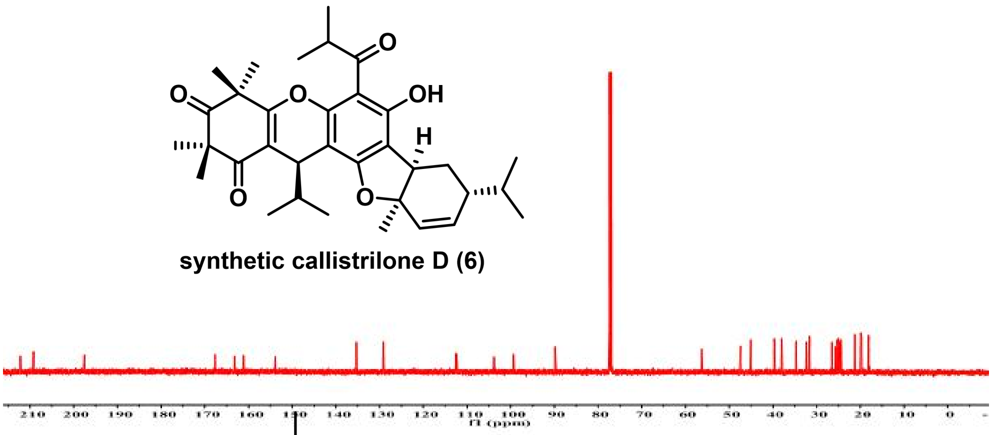


synthetic callistrilone D (6)

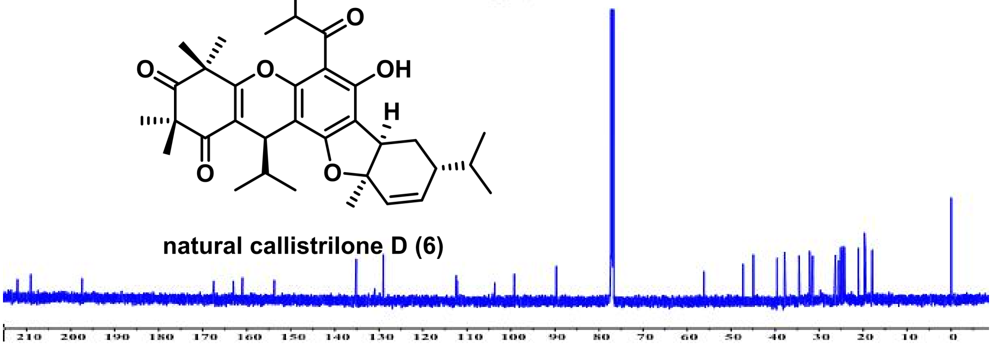

natural callistrilone D (6)

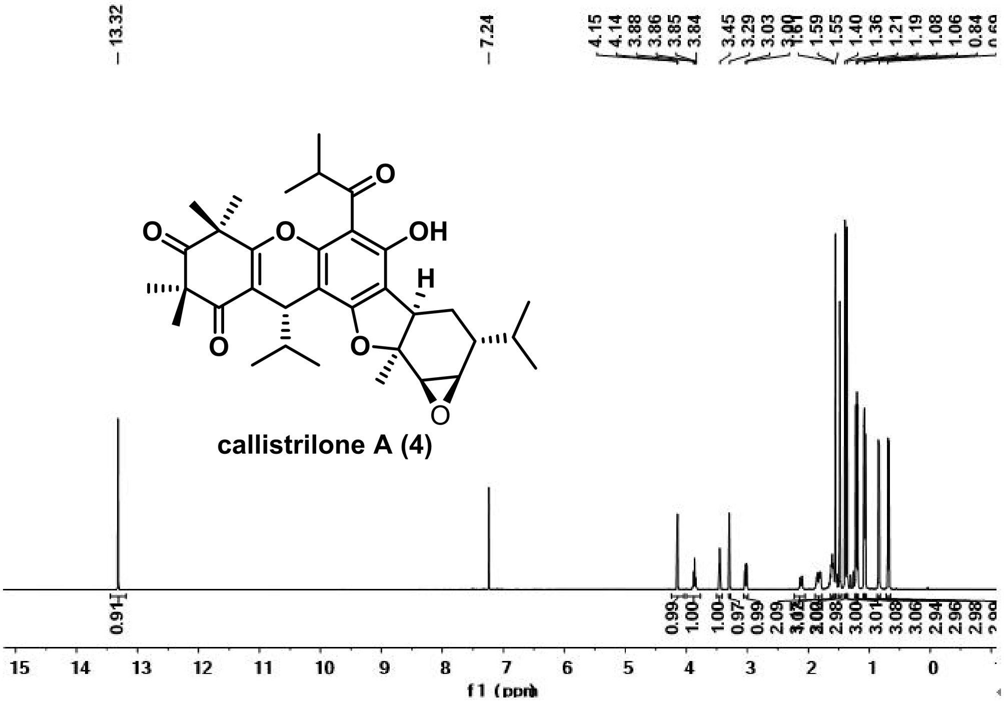


callistrilone A (4)

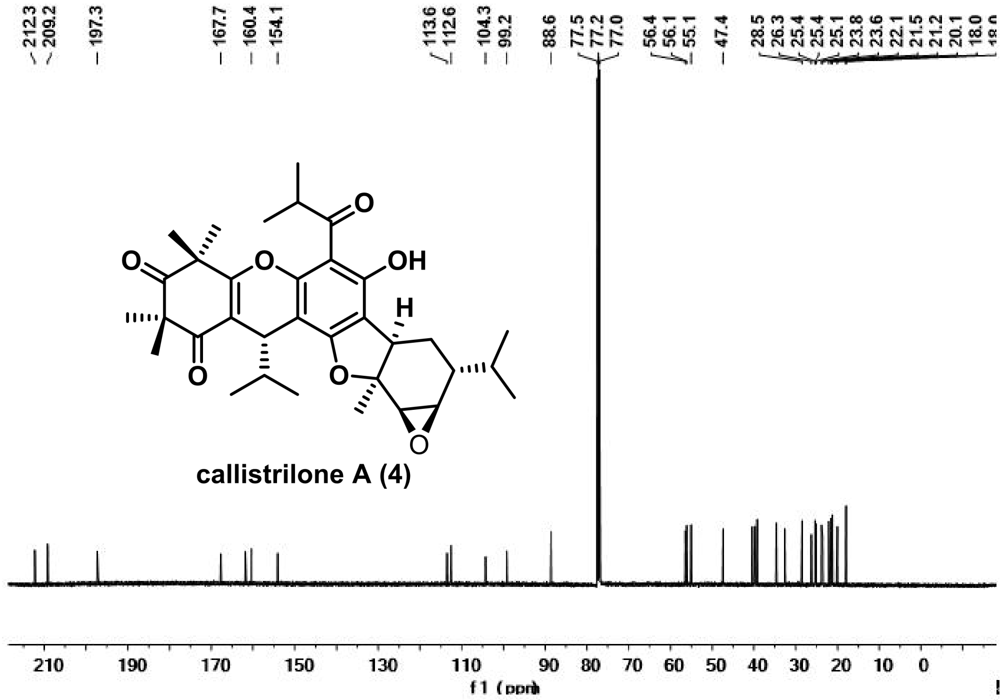
212.3
209.2
197.3
167.7
160.4
154.1
113.6
112.6
104.3
99.2
88.6
77.5
77.2
77.0
56.4
56.1
55.1
47.4
28.5
26.3
25.4
25.4
25.1
23.8
23.6
22.1
21.5
21.2
20.1
18.0
O
OH
H
callistrilone A (4)
210
190
170
150
130
110
90
80
70
60
50
40
30
20
10
0
f1 (ppm)

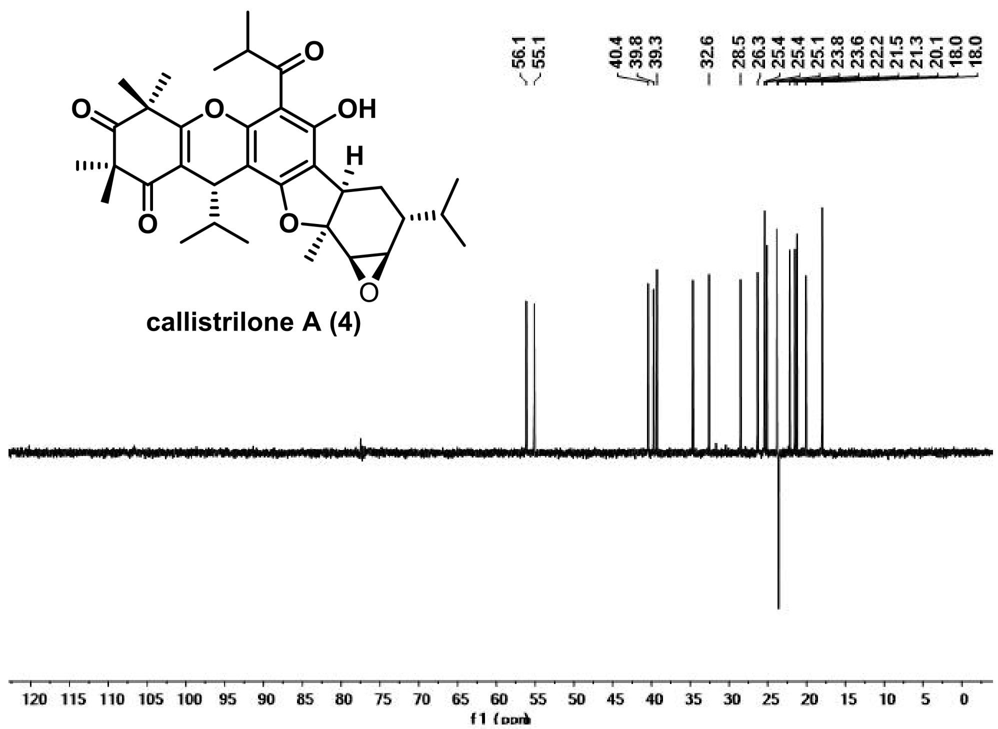
56.1
55.1
40.4
39.8
39.3
32.6
28.5
26.3
25.4
25.4
25.1
23.8
23.6
22.2
21.5
21.3
20.1
18.0
18.0
O
OH
H
callistrilone A (4)
120 115 110 105 100 95 90 85 80 75 70 65 60 55 50 45 40 35 30 25 20 15 10 5 0
f1 (ppm)

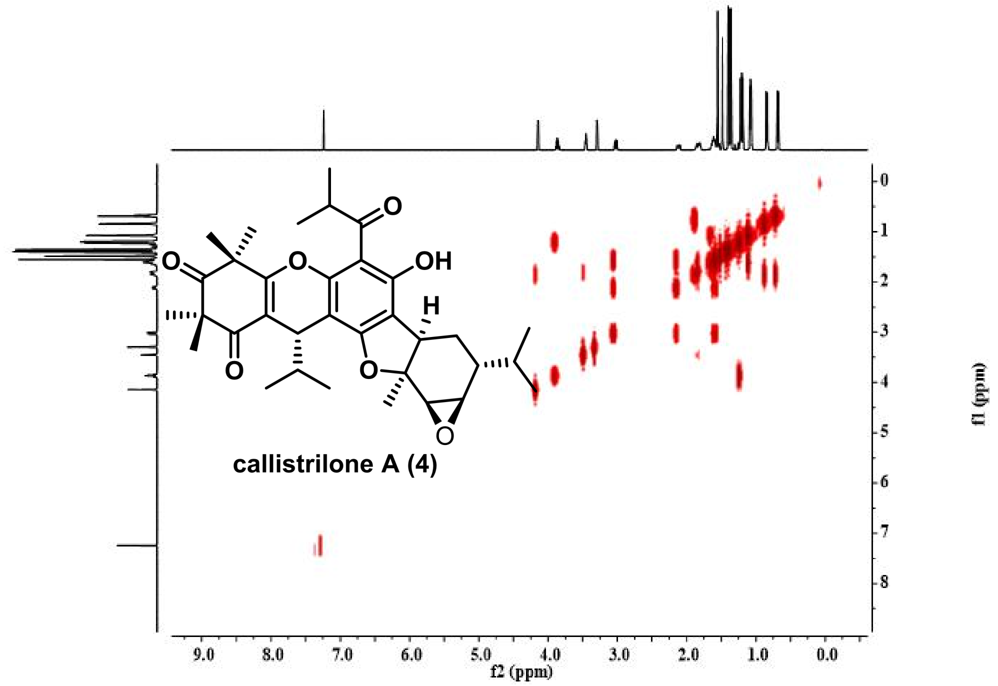


$^{1}$H-$^{1}$H COSY spectrum of callistrilone A (**4**)

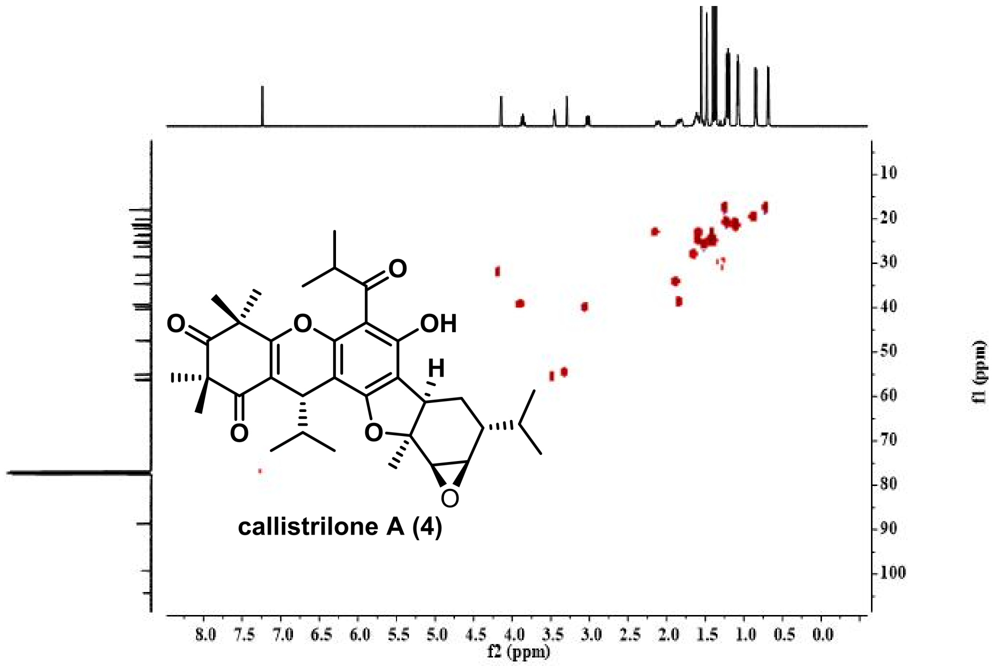


HSQC spectrum of callistrilone A (**4**)

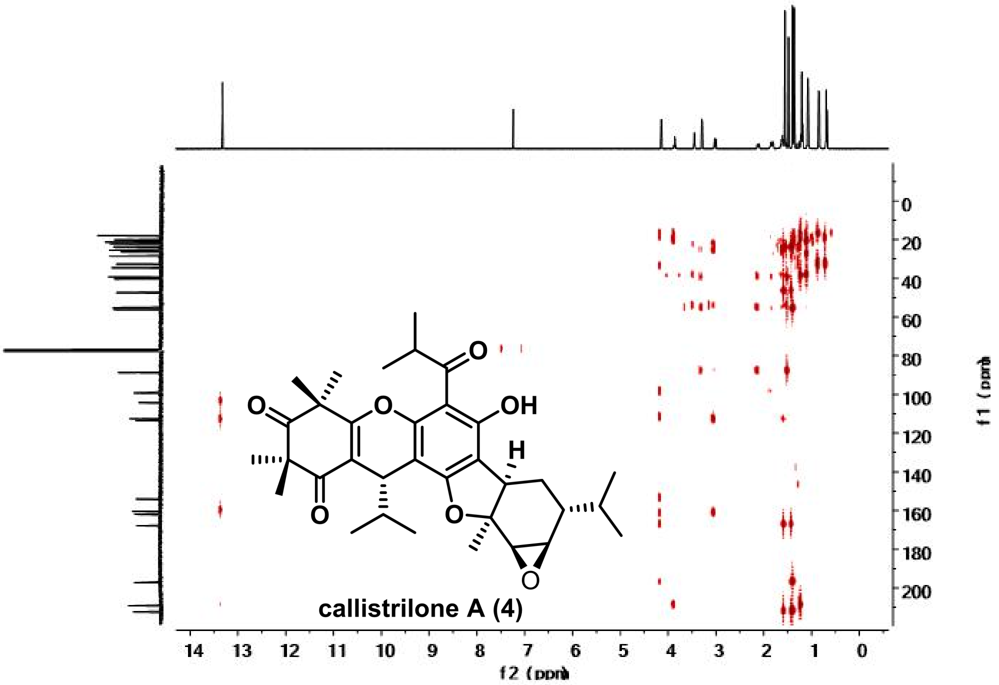


HMBC spectrum of callistrilone A (**4**)

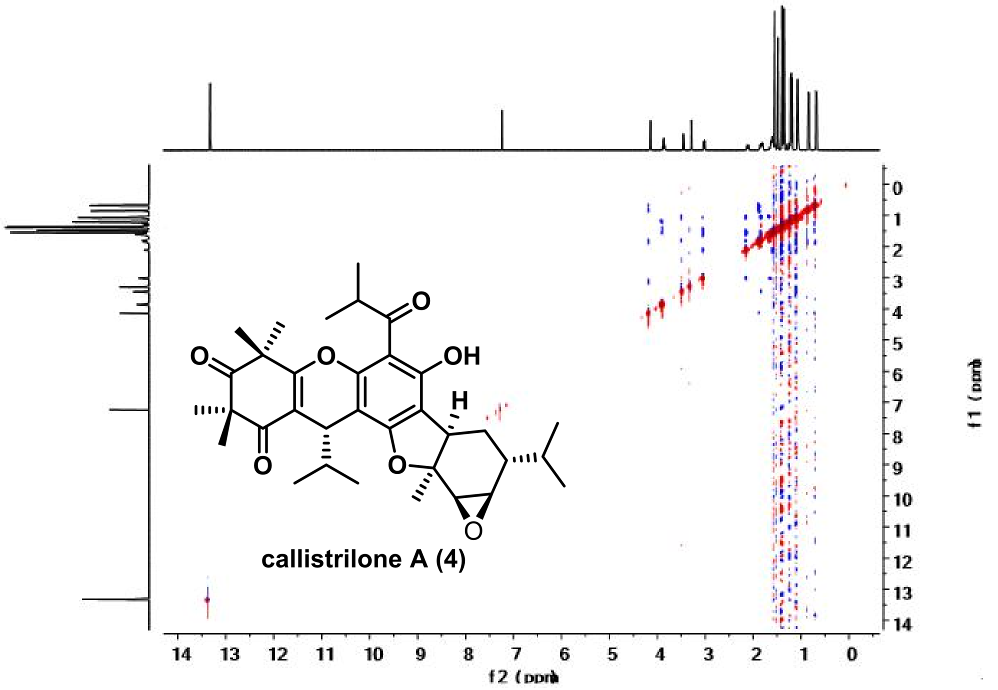


NOESY spectrum of callistrilone A (**4**)

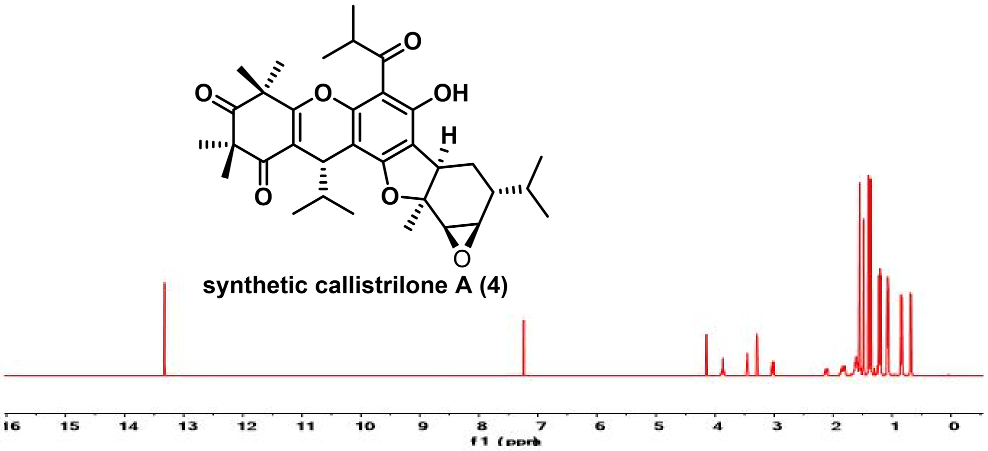

synthetic callistrilone A (4)

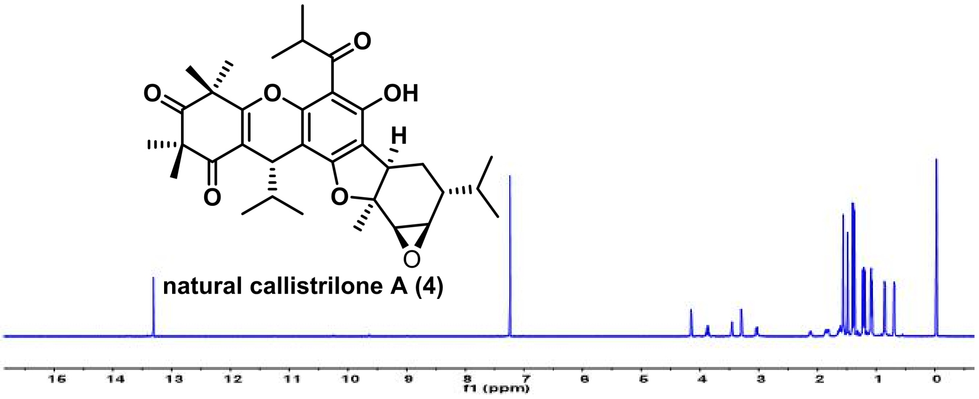

natural callistrilone A (4)

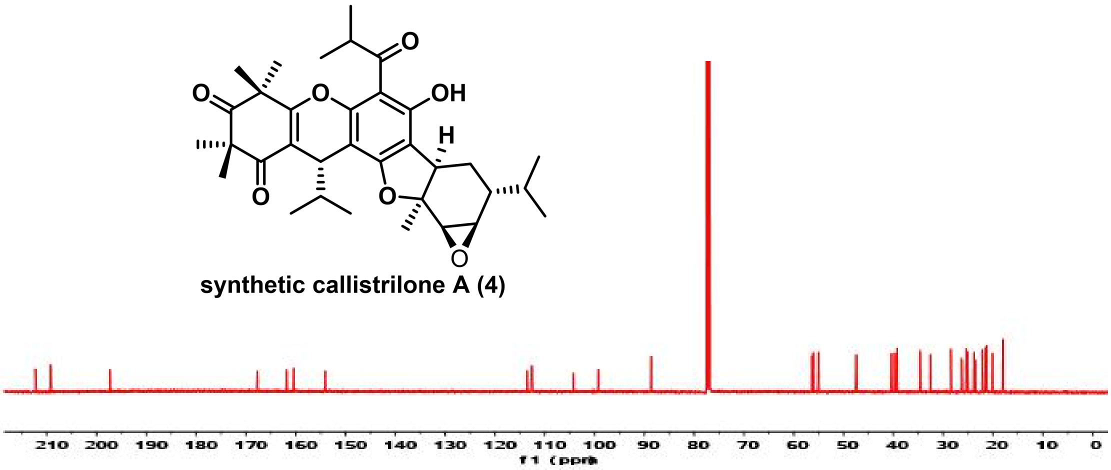

synthetic callistrilone A (4)

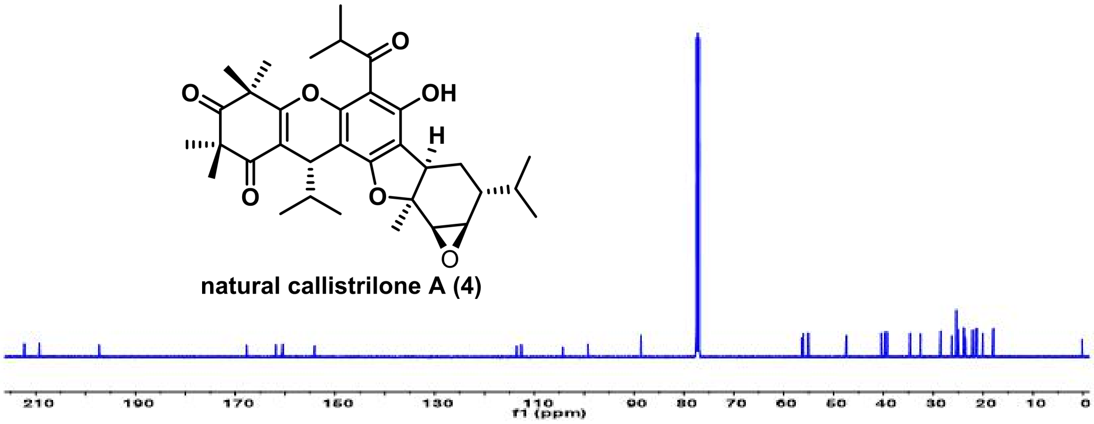

natural callistrilone A (4)

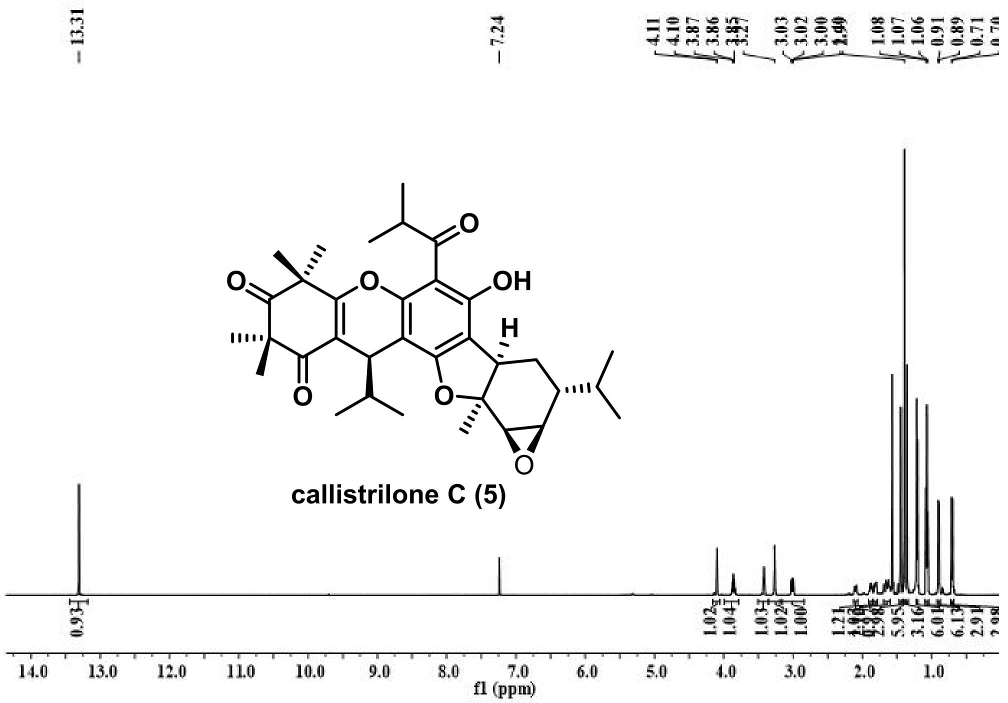
callistrilone C (5)
f1 (ppm)

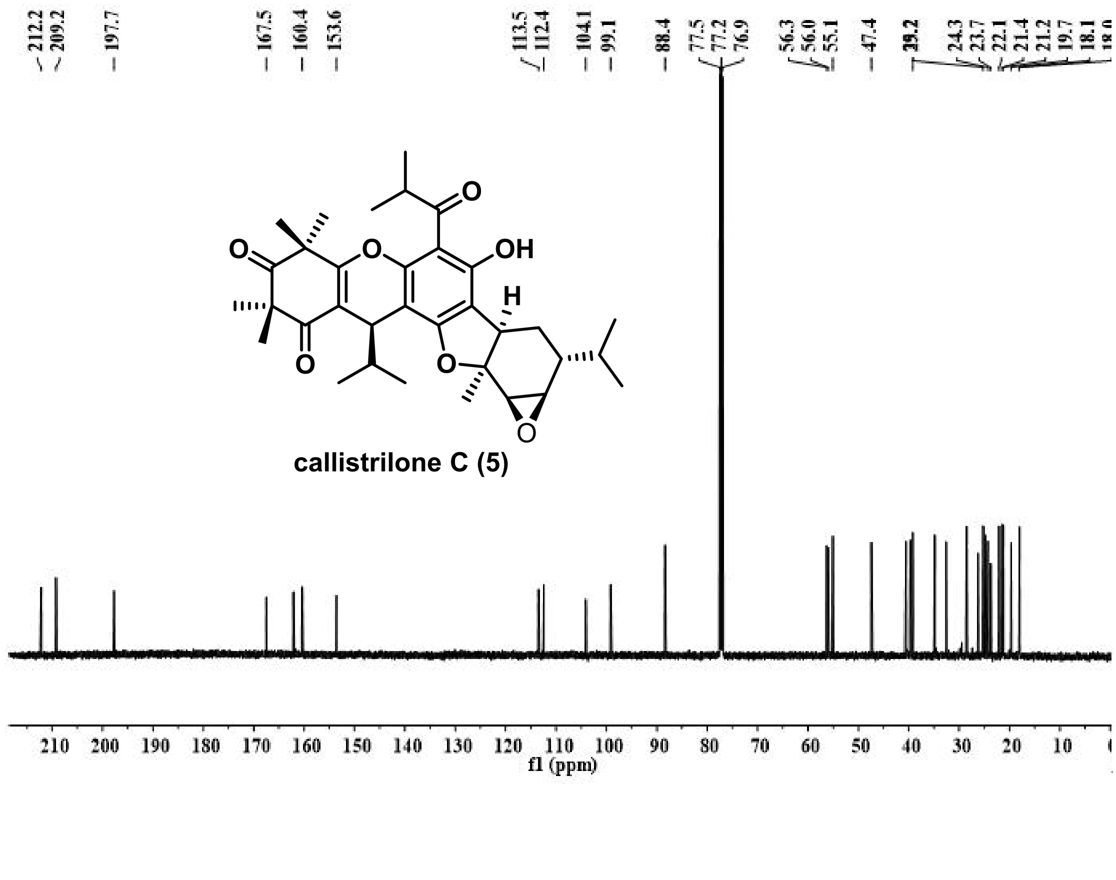
callistrilone C (5)
f1 (ppm)

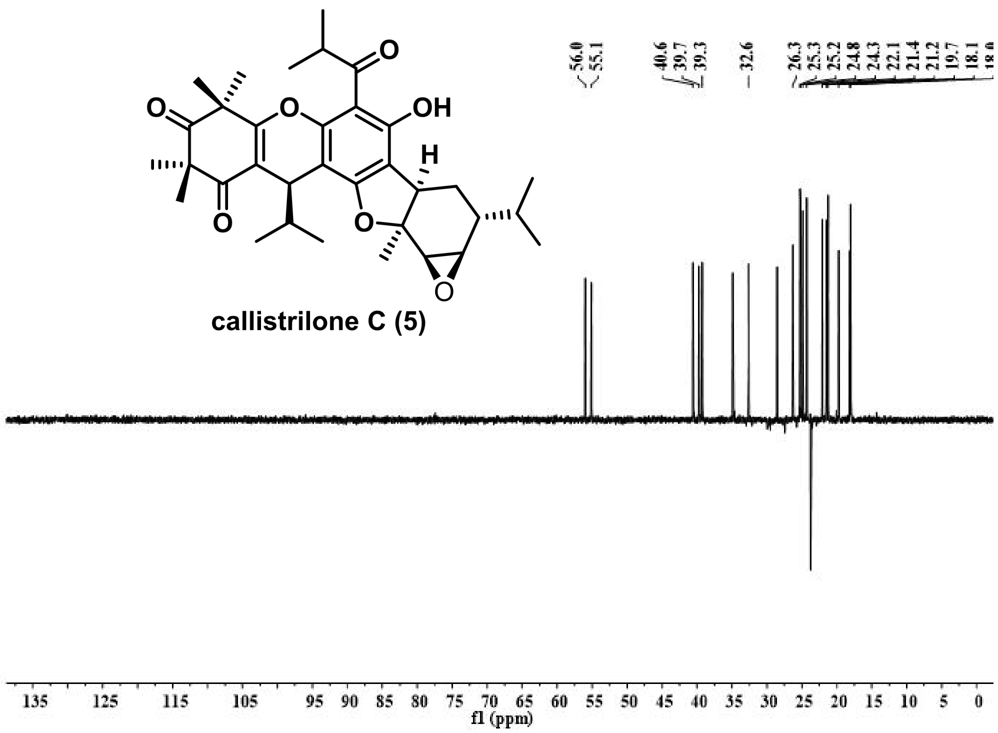


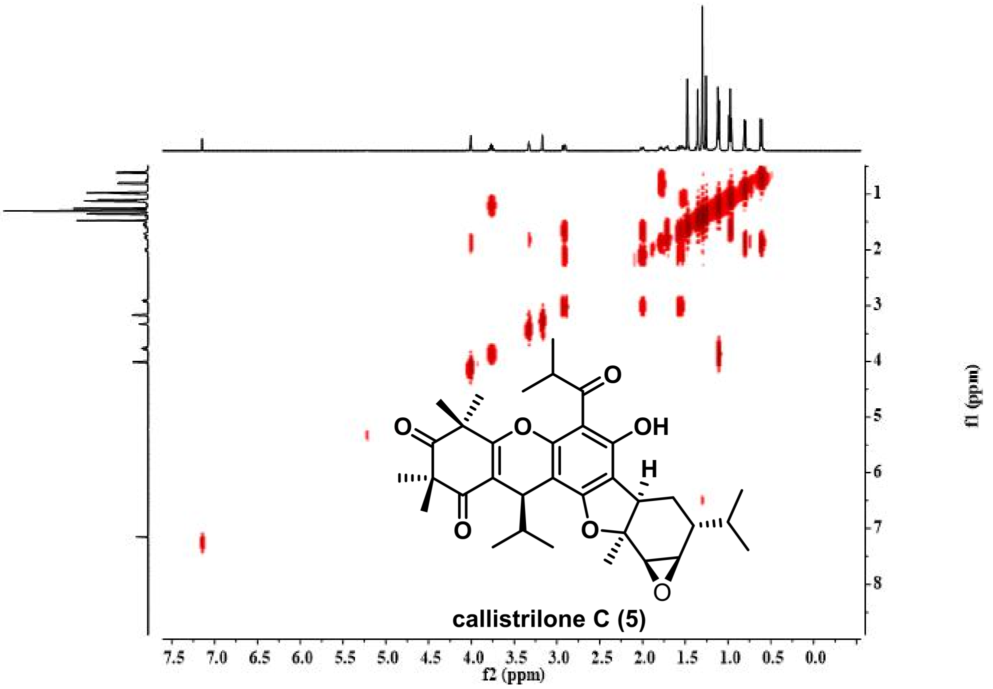


$^1$H-$^1$H COSY spectrum of callistrilone C (**5**)

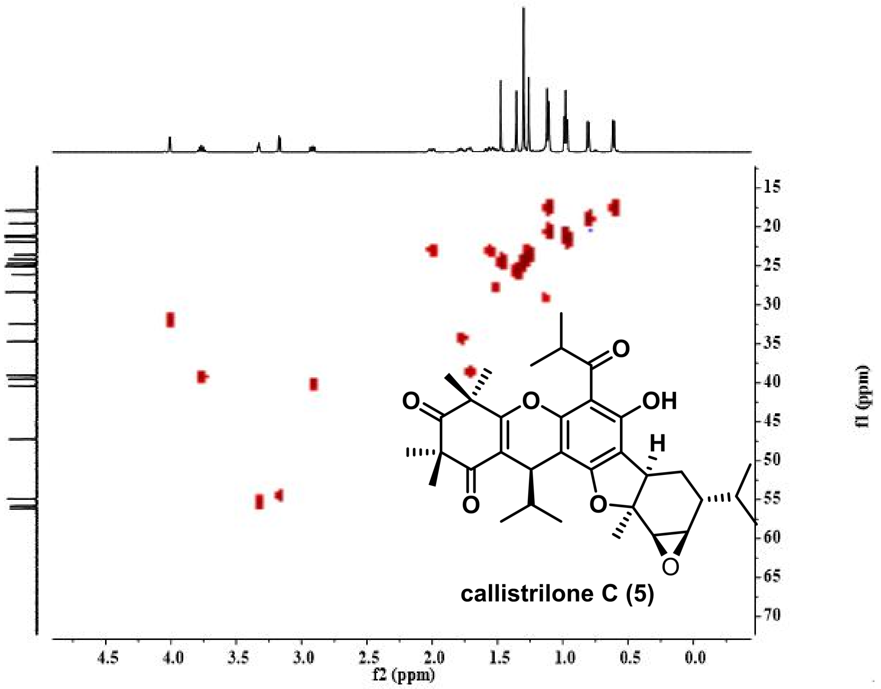


HSQC spectrum of callistrilone C (**5**)

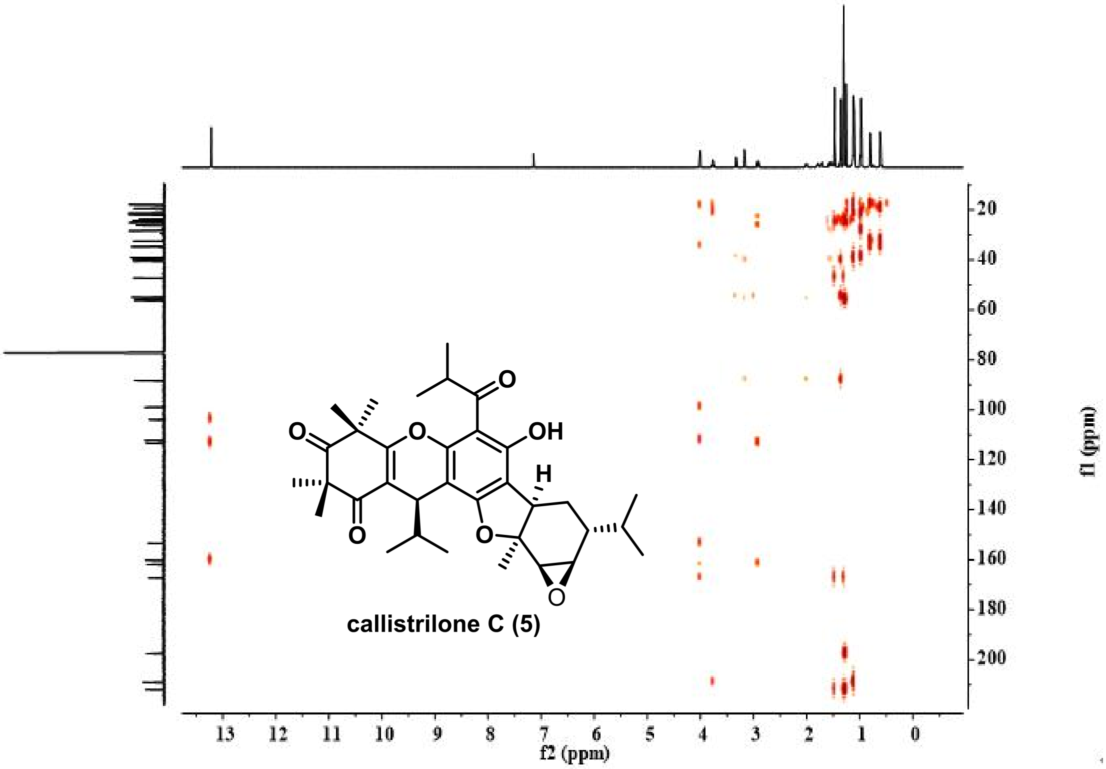


HMBC spectrum of callistrilone C (**5**)

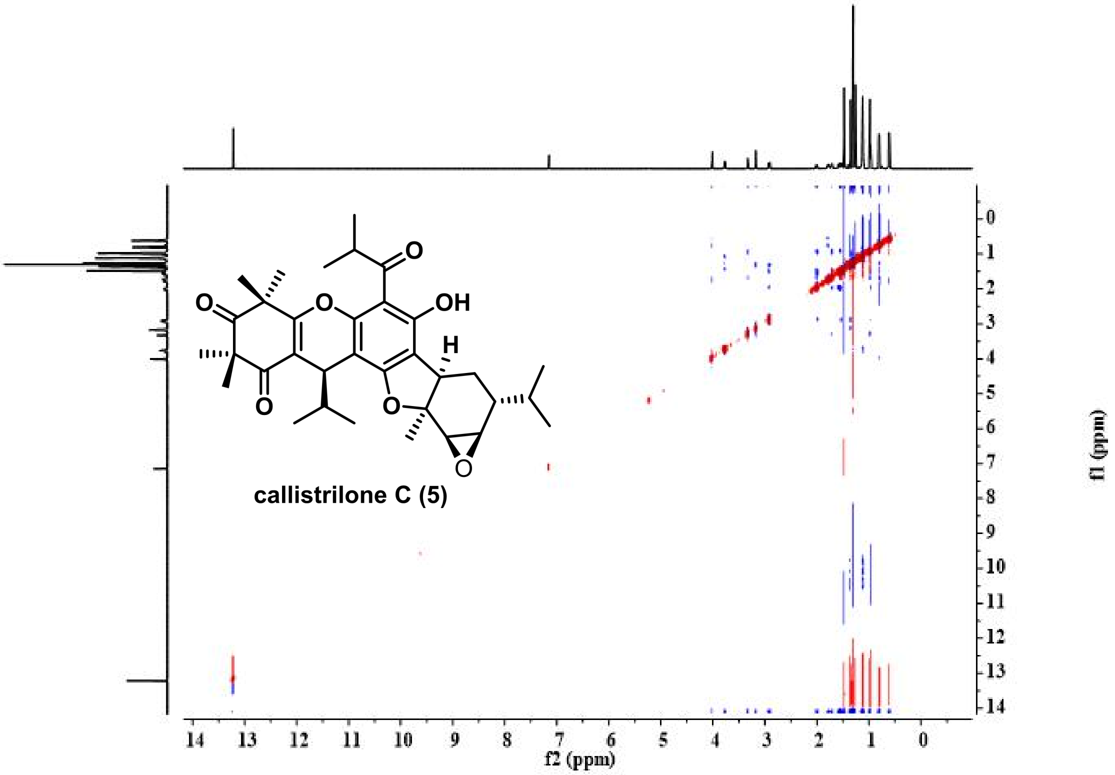


NOESY spectrum of callistrilone C (**5**)

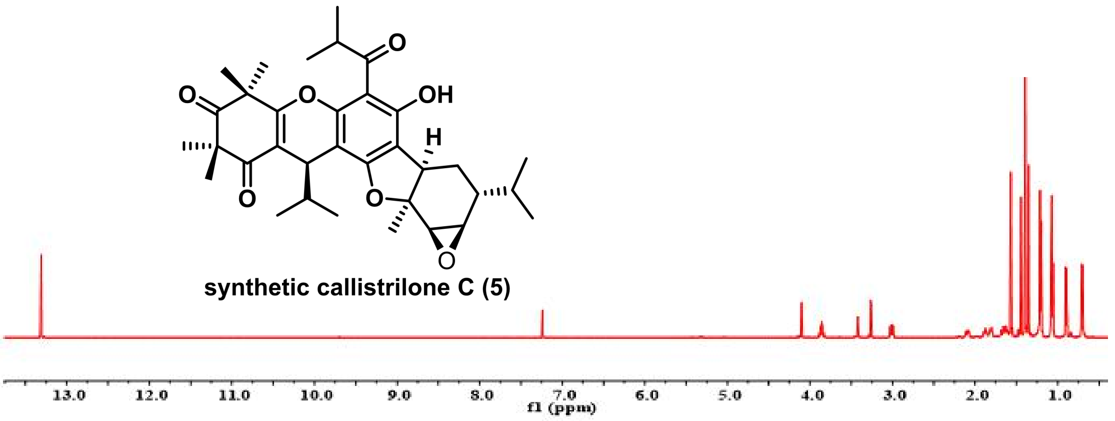


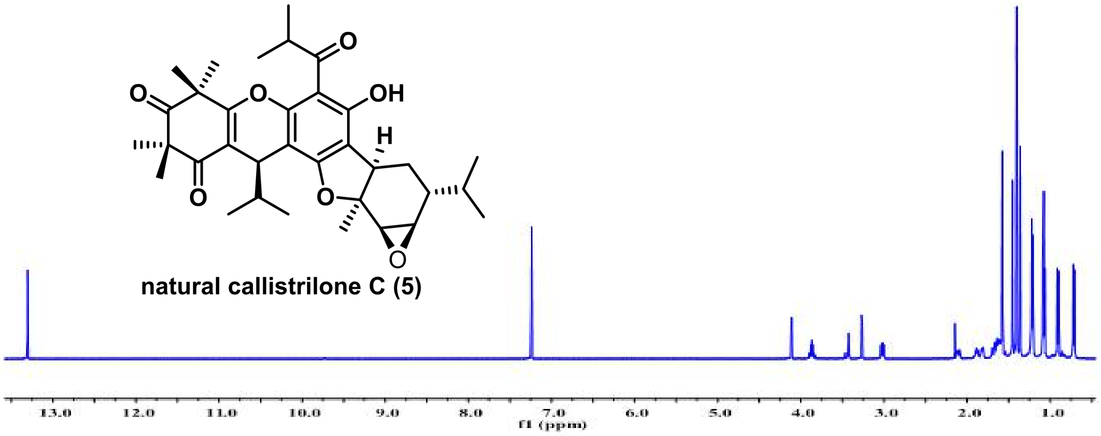

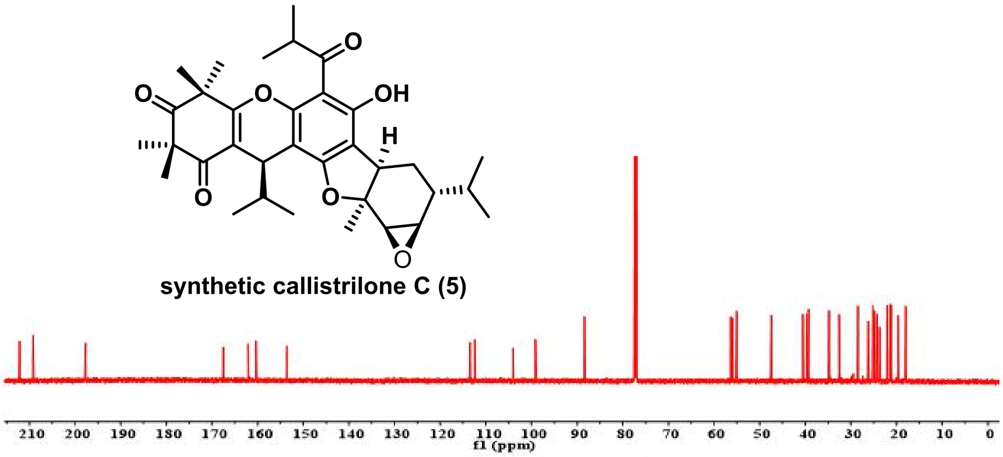
synthetic callistrilone C (5)
210 200 190 180 170 160 150 140 130 120 110 100 90 80 70 60 50 40 30 20 10 0
f1 (ppm)

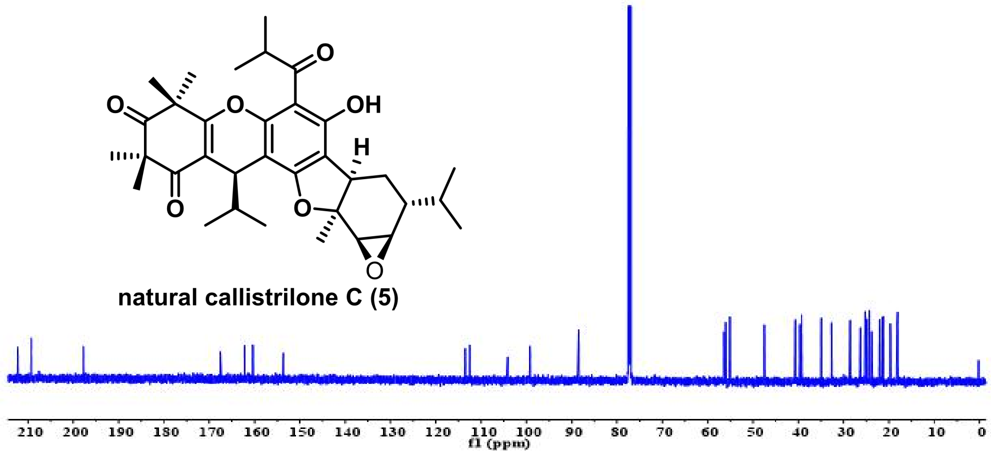
natural callistrilone C (5)
210 200 190 180 170 160 150 140 130 120 110 100 90 80 70 60 50 40 30 20 10 0
f1 (ppm)

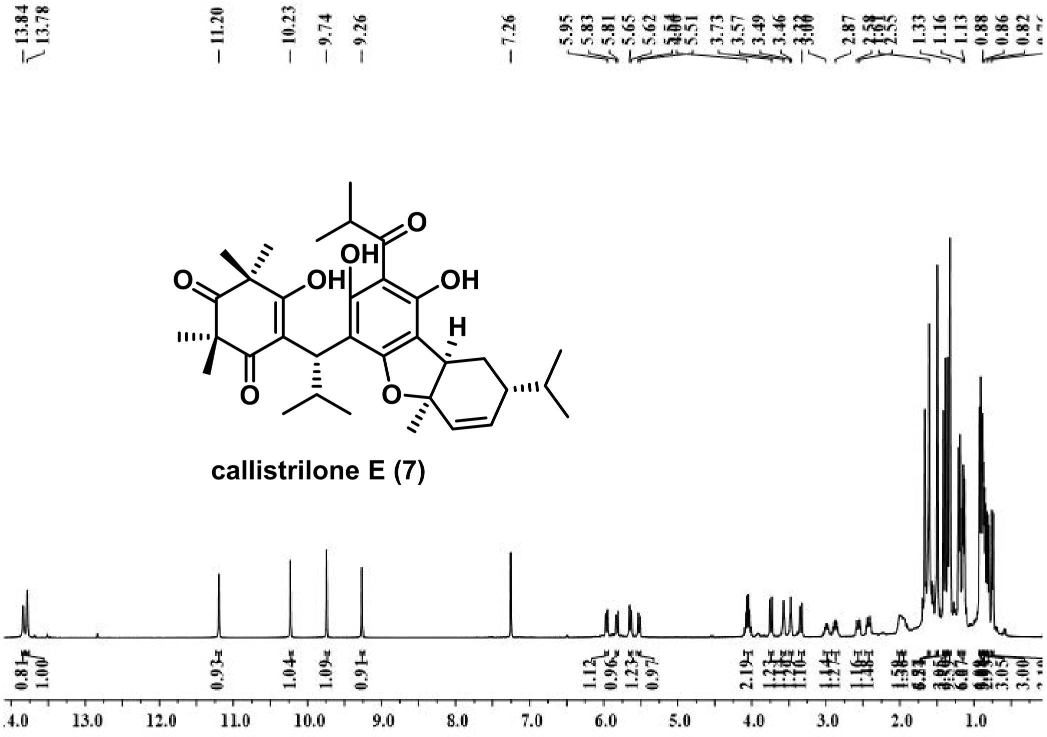
callistrilone E (7)
14.0 13.0 12.0 11.0 10.0 9.0 8.0 7.0 6.0 5.0 4.0 3.0 2.0 1.0

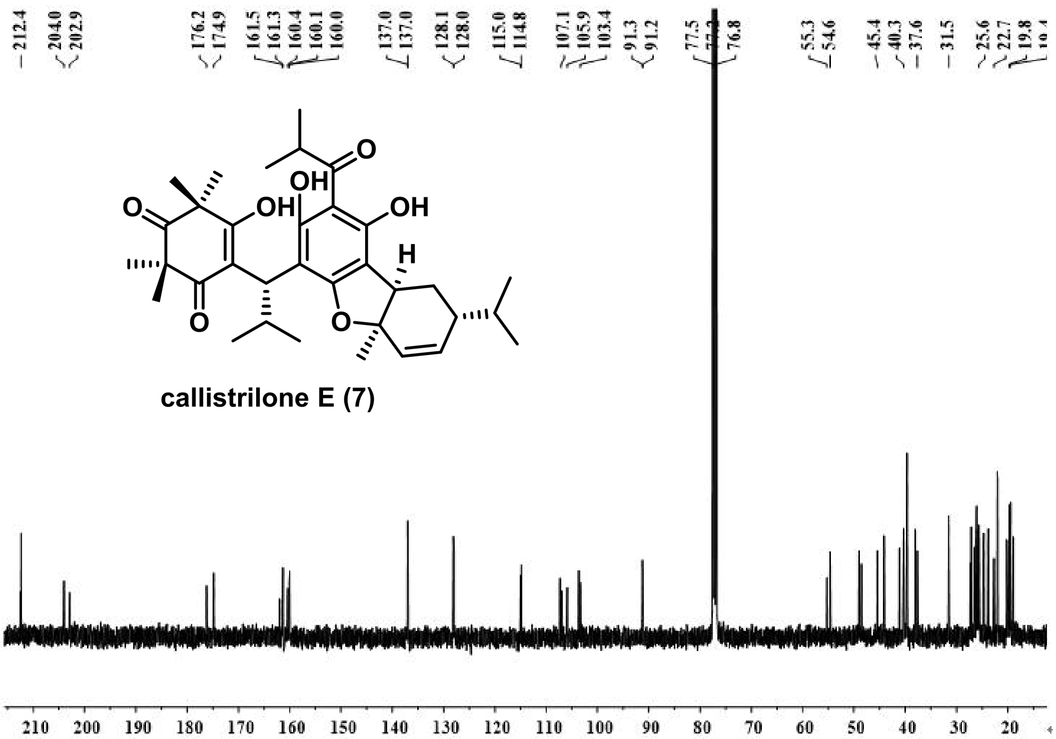

callistrilone E (7)

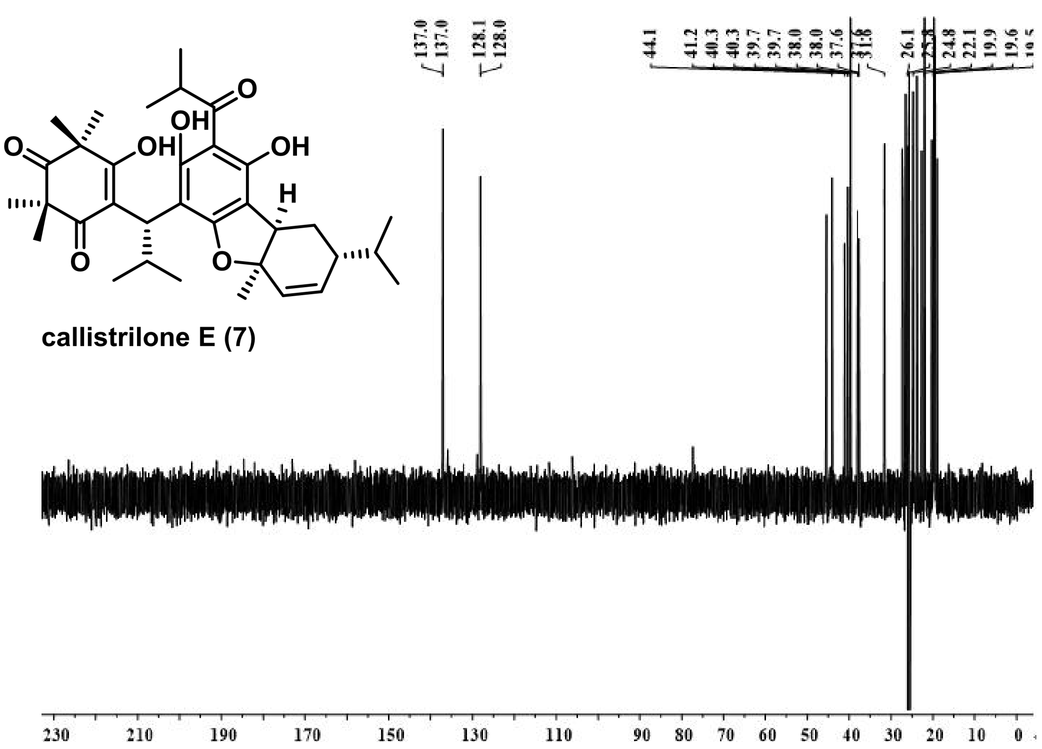

callistrilone E (7)

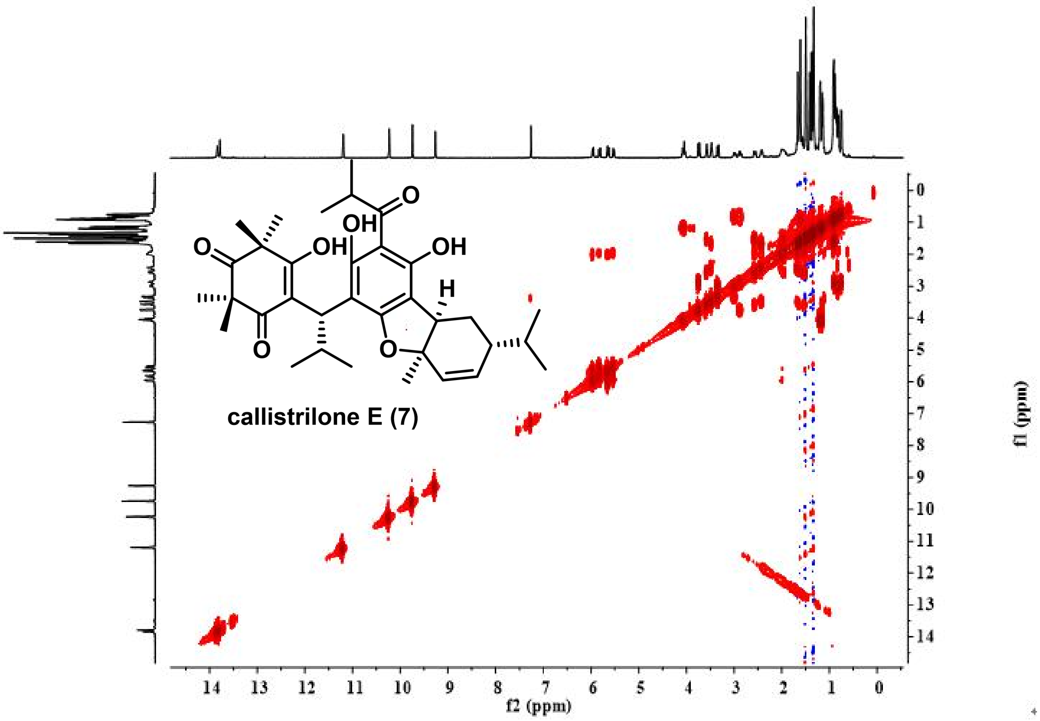


$^{1}$H-$^{1}$H COSY spectrum of callistrilone E (**7**)

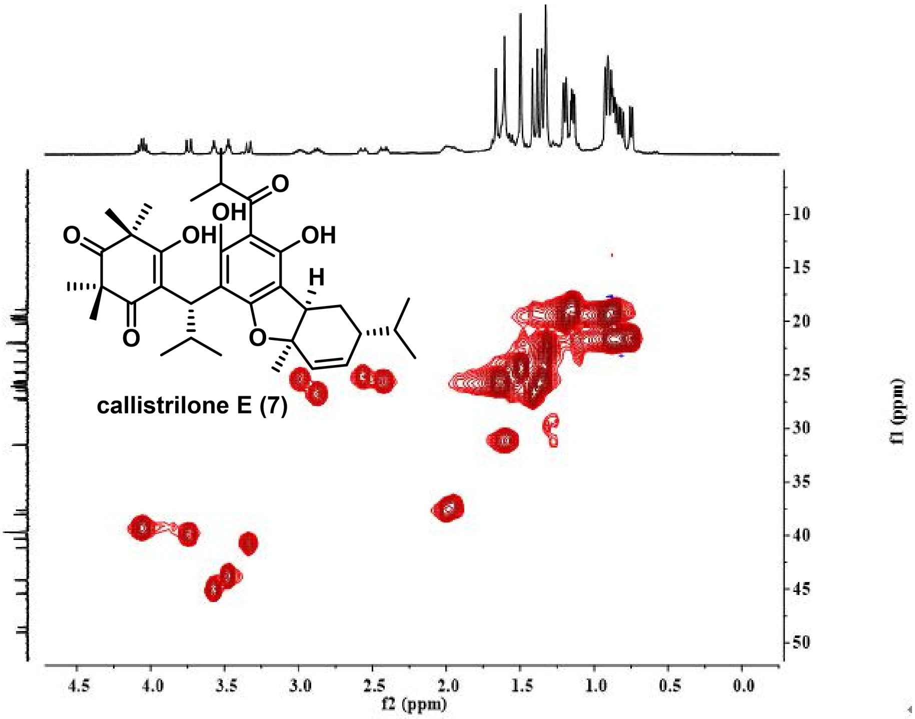


HSQC spectrum of callistrilone E (**7**)

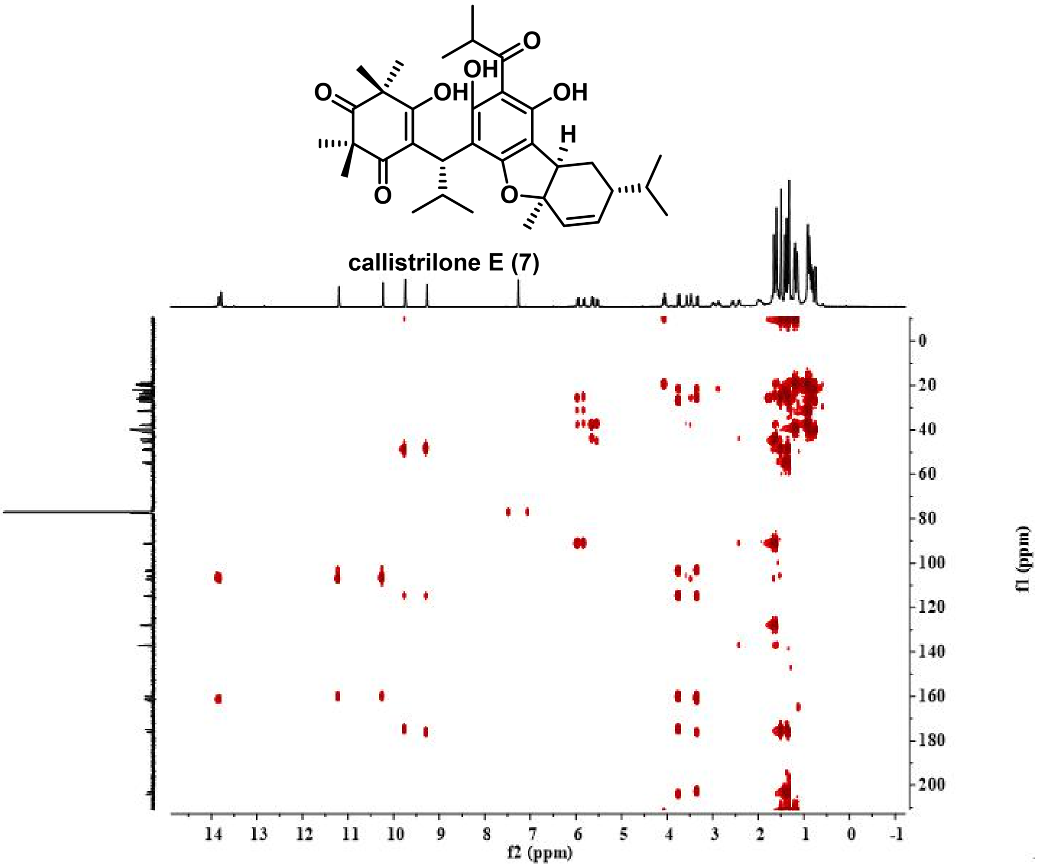


HMBC spectrum of callistrilone E (**7**)

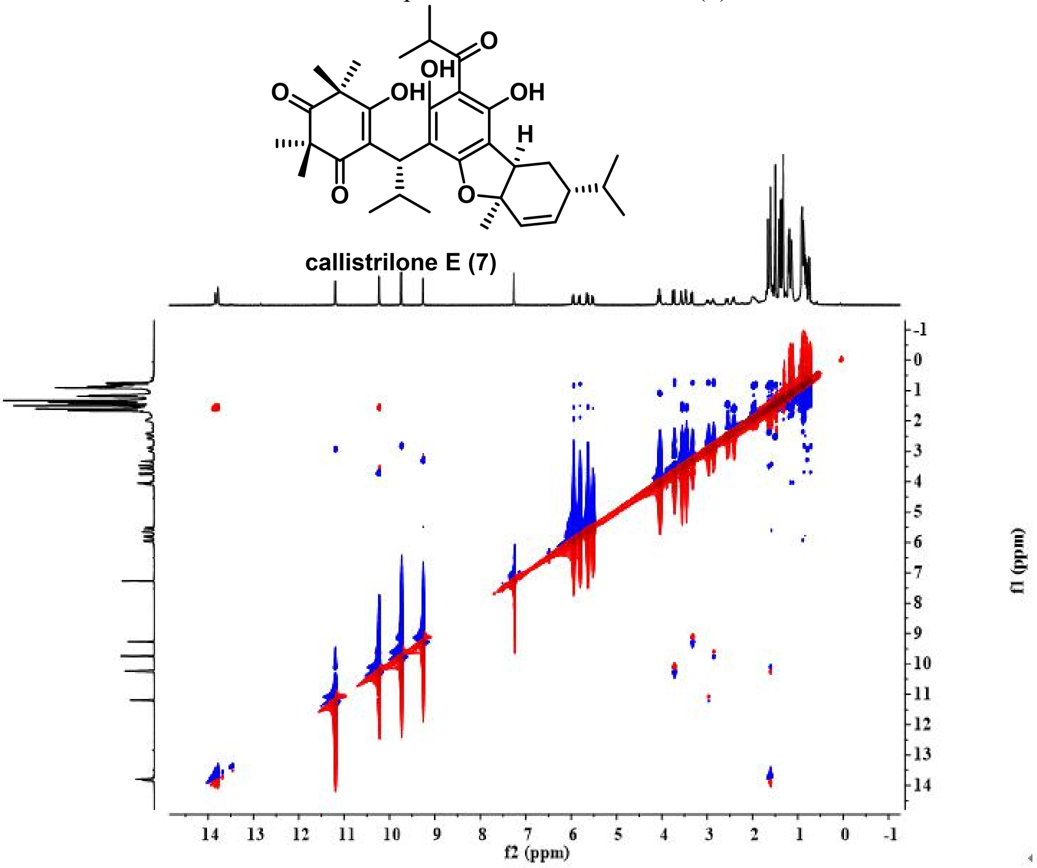


NOESY spectrum of callistrilone E (**7**)

## 5. HPLC chromatogram of 13, *ent*-13, 13a-13w

**13:** HPLC analysis: Daicel Chiralpak OD-H column; *n*-hexane/*i*-propanol = 95:5, 0.8 mL/min, $\lambda$ = 280 nm; major enantiomer: $t_R$ = 15.9 min, minor enantiomer: $t_R$ = 9.2 min. 95:5 *er*; re-crystallised: 99.7: 0.3 *er*.

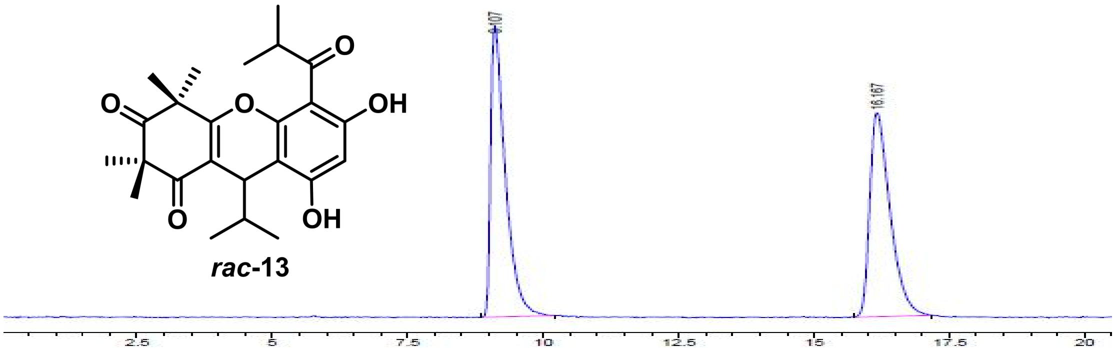


***rac*-13**

Signal 1: DAD1 G, Sig=280,4 Ref=360,100

| Peak # | RetTime [min] | Type | Width [min] | Area [mAU*s] | Height [mAU] | Area % |
|---|---|---|---|---|---|---|
| 1 | 9.107 | BB | 0.2732 | 2704.94263 | 143.99800 | 50.2785 |
| 2 | 16.167 | BB | 0.3952 | 2674.97778 | 100.91621 | 49.7215 |

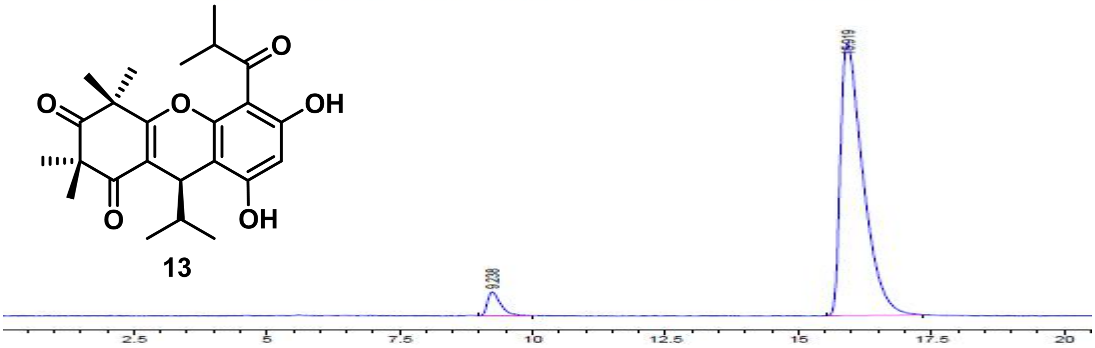


**13**

Signal 1: DAD1 G, Sig=280,4 Ref=360,100

| Peak # | RetTime [min] | Type | Width [min] | Area [mAU*s] | Height [mAU] | Area % |
|---|---|---|---|---|---|---|
| 1 | 9.238 | BB | 0.2479 | 378.64197 | 22.09569 | 4.8231 |
| 2 | 15.919 | BB | 0.4253 | 7471.87598 | 252.45042 | 95.1769 |

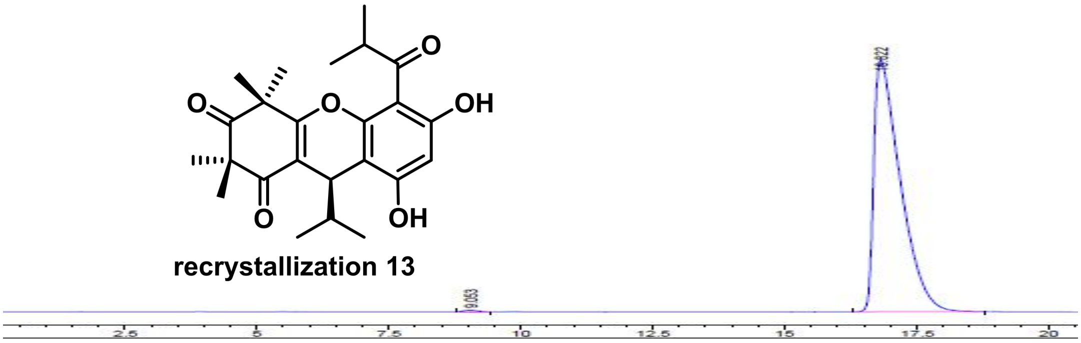


**recrystallization 13**

Signal 1: DAD1 G, Sig=280,4 Ref=360,100

| Peak # | RetTime [min] | Type | Width [min] | Area [mAU*s] | Height [mAU] | Area % |
|---|---|---|---|---|---|---|
| 1 | 9.053 | BB | 0.2386 | 92.20867 | 5.05904 | 0.3050 |
| 2 | 16.822 | BB | 0.4770 | 3.01355e4 | 863.91779 | 99.6950 |

***Ent*-13:** HPLC analysis: Daicel Chiralpak OD-H column; *n*-hexane/*i*-propanol = 95:5, 0.8 mL/min, $\lambda$ = 280 nm; major enantiomer: $t_R$ = 8.6 min, minor enantiomer: $t_R$ = 16.9 min. 95:5 *er*; re-crystallised: > 99.5:0.5 *er*.

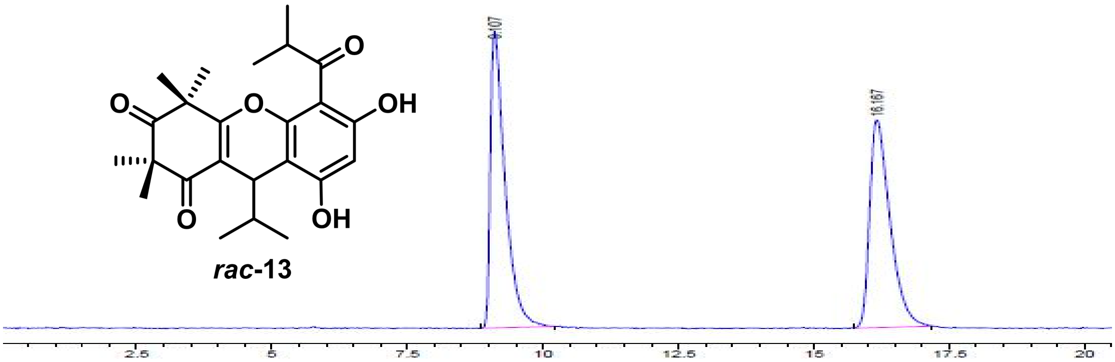


***rac*-13**

Signal 1: DAD1 G, Sig=280,4 Ref=360,100

| Peak # | RetTime [min] | Type | Width [min] | Area [mAU*s] | Height [mAU] | Area % |
|---|---|---|---|---|---|---|
| 1 | 9.107 | BB | 0.2732 | 2704.94263 | 143.99800 | 50.2785 |
| 2 | 16.167 | BB | 0.3952 | 2674.97778 | 100.91621 | 49.7215 |

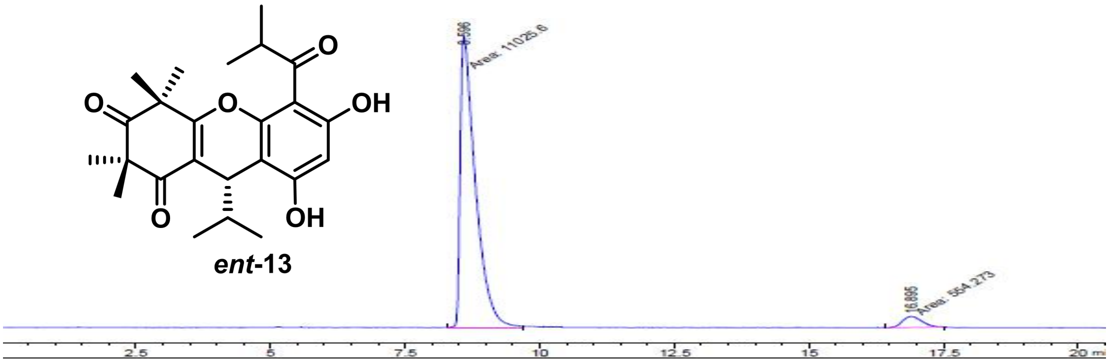


***ent*-13**

Signal 1: DAD1 G, Sig=280,4 Ref=360,100

| Peak # | RetTime [min] | Type | Width [min] | Area [mAU*s] | Height [mAU] | Area % |
|---|---|---|---|---|---|---|
| 1 | 8.596 | MM | 0.3289 | 1.10256e4 | 558.71802 | 95.2135 |
| 2 | 16.895 | MM | 0.4307 | 554.27350 | 21.45082 | 4.7865 |

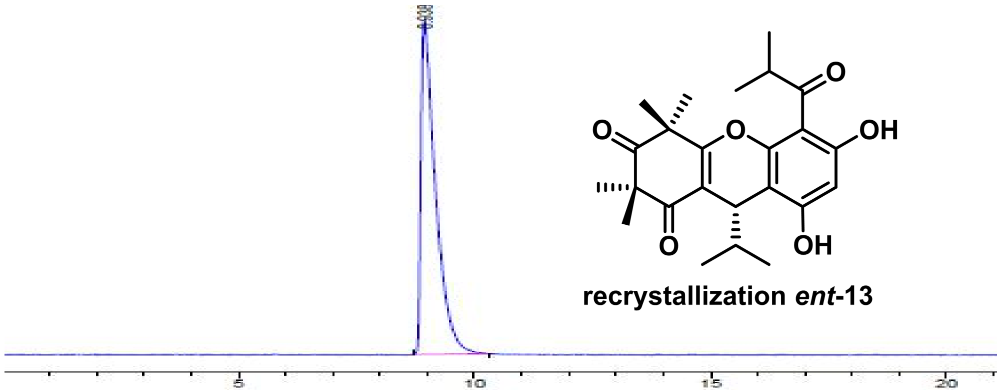


Signal 1: DAD1 G, Sig=280,4 Ref=360,100

| Peak # | RetTime [min] | Type | Width [min] | Area [mAU*s] | Height [mAU] | Area % |
|---|---|---|---|---|---|---|
| 1 | 8.938 | BB | 0.3008 | 7719.45215 | 359.14020 | 100.0000 |

**13a:** HPLC analysis: Daicel Chiralpak OD-H column; *n*-hexane/*i*-propanol = 90:10, 1 mL/min, $\lambda$ = 280 nm; major enantiomer: $t_R$ = 4.6 min, minor enantiomer: $t_R$ = 6.7 min. 93.5:6.5 *er*.

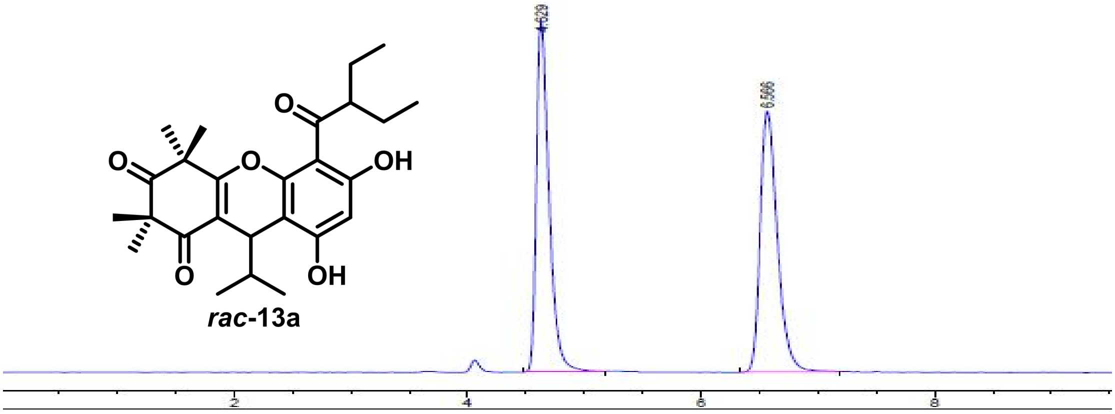


| Peak # | RetTime [min] | Type | Width [min] | Area [mAU*s] | Height [mAU] | Area % |
|---|---|---|---|---|---|---|
| 1 | 4.629 | BV | 0.1153 | 3362.11914 | 439.39526 | 50.0883 |
| 2 | 6.566 | BB | 0.1552 | 3350.26172 | 327.90692 | 49.9117 |

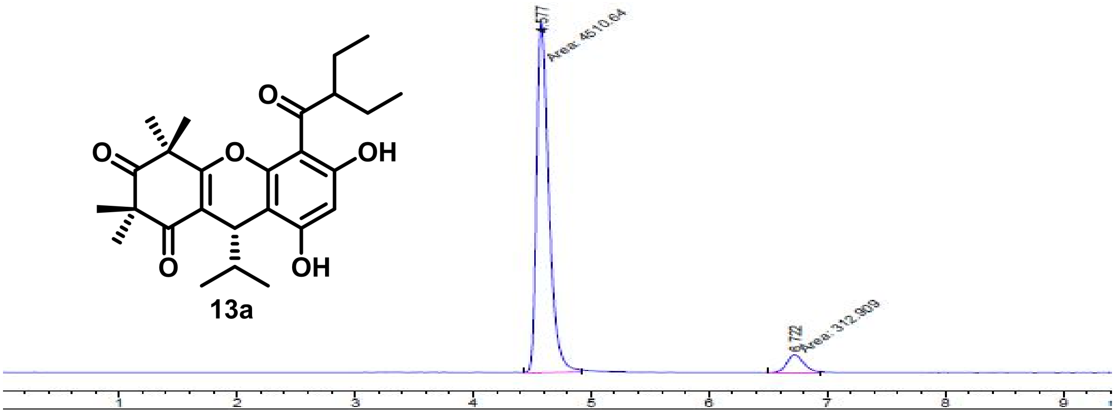


13a

| Peak # | RetTime [min] | Type | Width [min] | Area [mAU*s] | Height [mAU] | Area % |
|---|---|---|---|---|---|---|
| 1 | 4.577 | MM | 0.1202 | 4510.64258 | 625.34534 | 93.5129 |
| 2 | 6.722 | MF | 0.1646 | 312.90939 | 31.68290 | 6.4871 |

**13b:** HPLC analysis: Daicel Chiralpak OD-H column; *n*-hexane/*i*-propanol = 90:10, 1 mL/min, $\lambda$ = 280 nm; major enantiomer: $t_R$ = 6.7 min, minor enantiomer: $t_R$ = 8.3 min. 91:9 *er*.

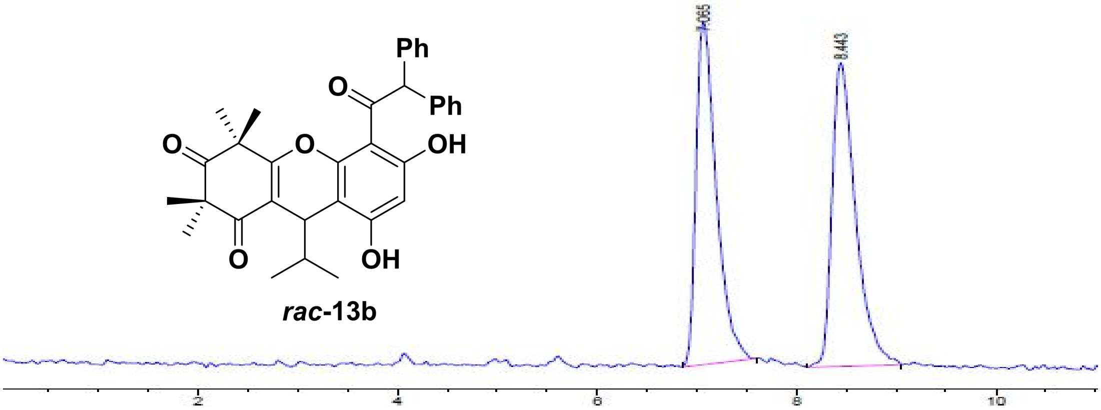


*rac*-13b

| Peak # | RetTime [min] | Type | Width [min] | Area [mAU*s] | Height [mAU] | Area % |
|---|---|---|---|---|---|---|
| 1 | 7.065 | BB | 0.2071 | 472.65036 | 33.39808 | 49.2101 |
| 2 | 8.443 | BB | 0.2404 | 487.82321 | 29.58364 | 50.7899 |

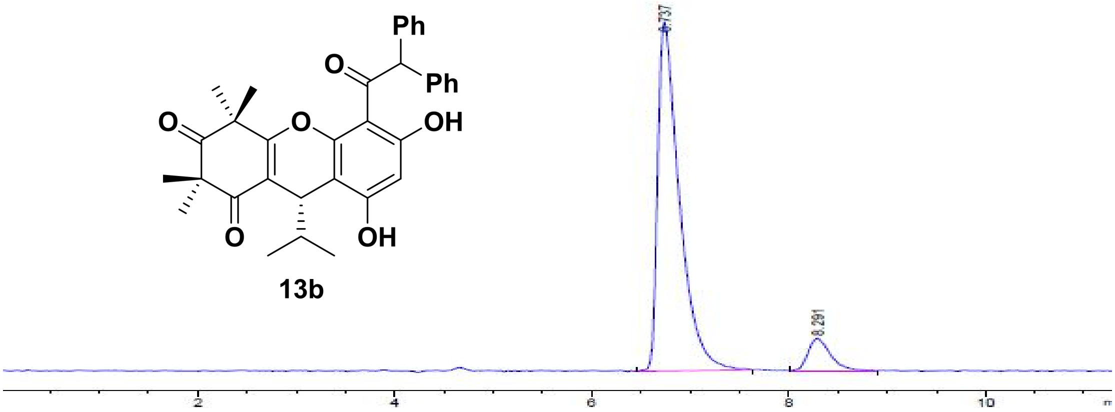


13b

| Peak # | RetTime [min] | Type | Width [min] | Area [mAU*s] | Height [mAU] | Area % |
|---|---|---|---|---|---|---|
| 1 | 6.737 | BB | 0.2292 | 2177.91821 | 141.81833 | 91.2038 |
| 2 | 8.291 | BB | 0.2263 | 210.04982 | 13.15269 | 8.7962 |

**13c:** HPLC analysis: Daicel Chiralpak OD-H column; *n*-hexane/*i*-propanol = 90:10, 1 mL/min, $\lambda$ = 280 nm; major enantiomer: $t_R$ = 6.8 min, minor enantiomer: $t_R$ = 9.5 min. 92.5:7.5 *er*, re-crystallised: 98.5:1.5 *er*.

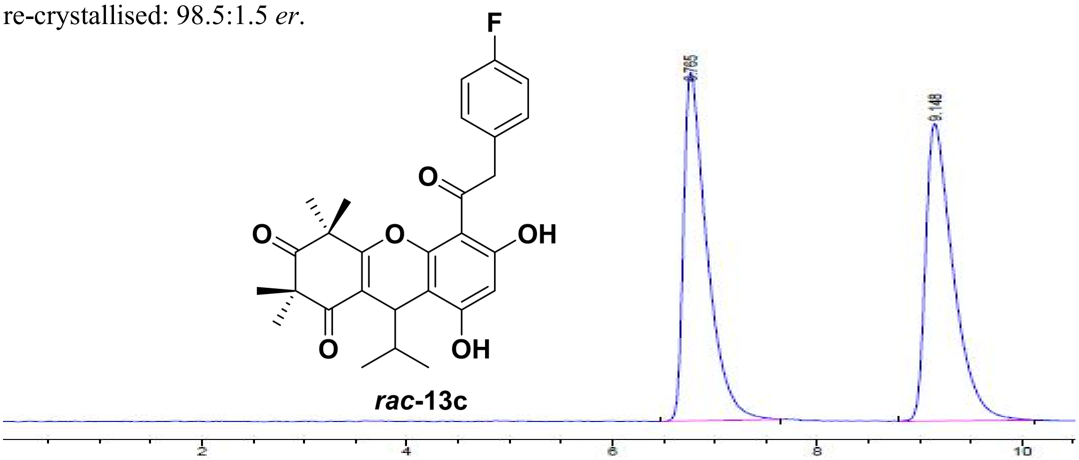


| Peak # | RetTime [min] | Type | Width [min] | Area [mAU*s] | Height [mAU] | Area % |
|---|---|---|---|---|---|---|
| 1 | 6.765 | BB | 0.2292 | 3451.85620 | 217.39728 | 49.8771 |
| 2 | 9.148 | VB | 0.2763 | 3468.86890 | 185.40590 | 50.1229 |

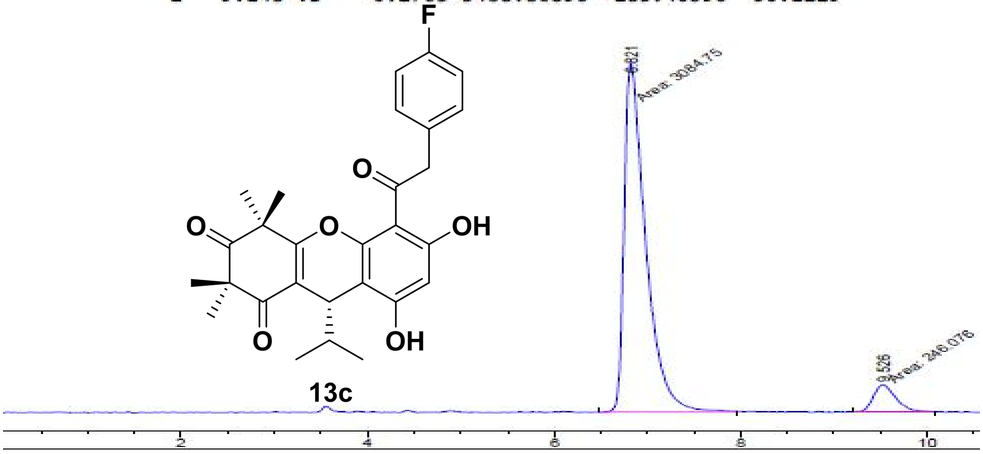


| Peak # | RetTime [min] | Type | Width [min] | Area [mAU*s] | Height [mAU] | Area % |
|---|---|---|---|---|---|---|
| 1 | 6.821 | MM | 0.2664 | 3084.75220 | 193.01083 | 92.6122 |
| 2 | 9.526 | PM | 0.2757 | 246.07602 | 14.87812 | 7.3878 |

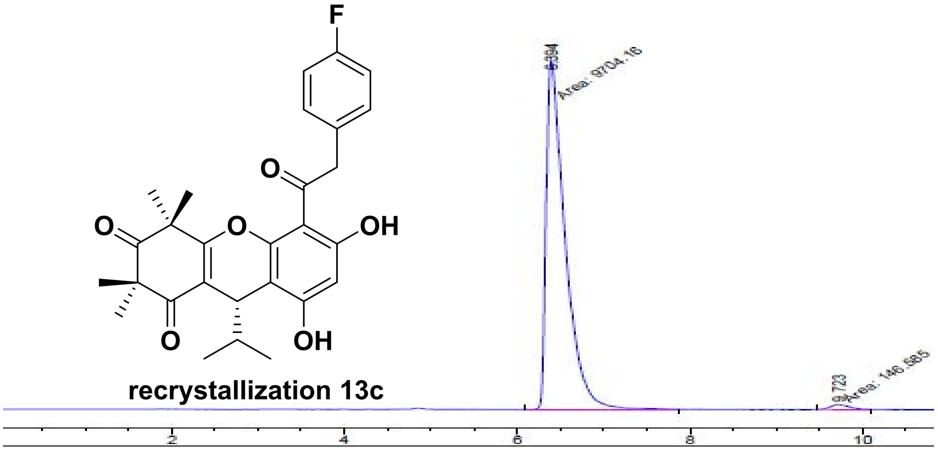


**recrystallization 13c**

| Peak # | RetTime [min] | Type | Width [min] | Area [mAU*s] | Height [mAU] | Area % |
|---|---|---|---|---|---|---|
| 1 | 6.394 | MM | 0.2548 | 9704.16406 | 634.76617 | 98.5119 |
| 2 | 9.723 | PM | 0.2703 | 146.58549 | 9.03719 | 1.4881 |

**13d:** HPLC analysis: Daicel Chiralpak OD-H column; *n*-hexane/*i*-propanol = 90:10, 1 mL/min, $\lambda$ = 280 nm;major enantiomer: $t_R$ = 5.9 min, minor enantiomer: $t_R$ = 9.4 min. 93.5:6.5 *er*.

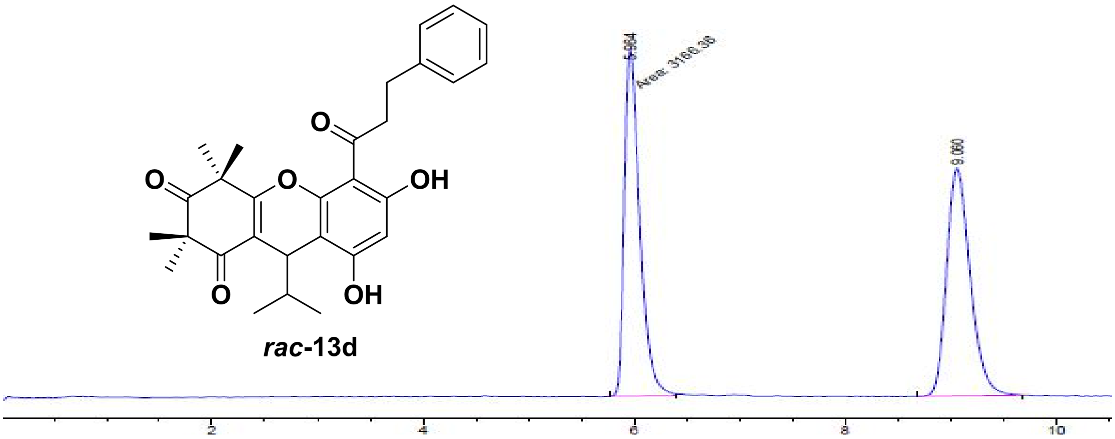


***rac*-13d**

| Peak # | RetTime [min] | Type | Width [min] | Area [mAU*s] | Height [mAU] | Area % |
|---|---|---|---|---|---|---|
| 1 | 5.964 | MM | 0.1697 | 3166.35913 | 311.03177 | 49.8578 |
| 2 | 9.060 | BB | 0.2392 | 3184.41943 | 205.07071 | 50.1422 |

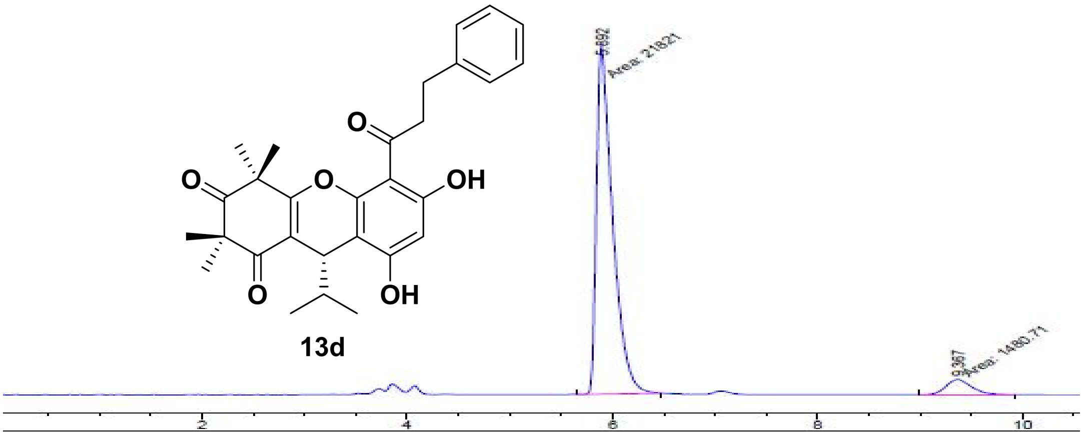


**13d**

| Peak # | RetTime [min] | Type | Width [min] | Area [mAU*s] | Height [mAU] | Area % |
|---|---|---|---|---|---|---|
| 1 | 5.892 | MP | 0.1818 | 2.18210e4 | 1999.93726 | 93.6455 |
| 2 | 9.367 | PM | 0.2874 | 1480.70862 | 85.87373 | 6.3545 |

**13e:** HPLC analysis: Daicel Chiralpak OD-H column; *n*-hexane/*i*-propanol = 90:10, 1 mL/min, $\lambda$ = 280 nm; major enantiomer: $t_R$ = 7.5 min, minor enantiomer: $t_R$ = 9.9 min. 94.5:5.5 *er*, re-crystallised: 99:1 *er*.

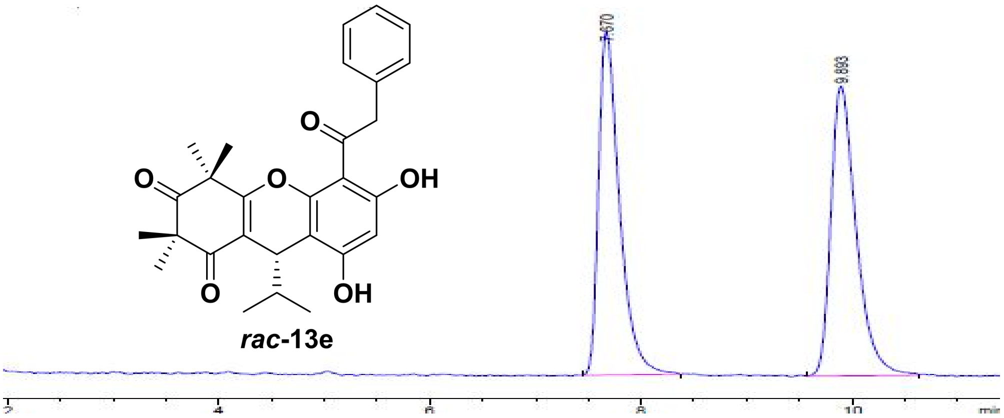


| Peak # | RetTime [min] | Type | Width [min] | Area [mAU*s] | Height [mAU] | Area % |
|---|---|---|---|---|---|---|
| 1 | 7.670 | BB | 0.2090 | 1456.64600 | 105.57006 | 49.4805 |
| 2 | 9.893 | VV | 0.2528 | 1487.23047 | 89.07196 | 50.5195 |

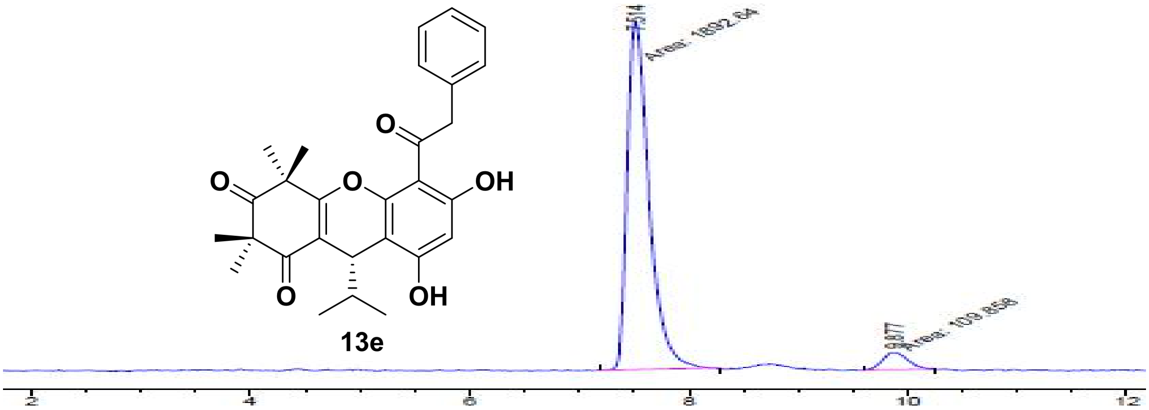


| Peak # | RetTime [min] | Type | Width [min] | Area [mAU*s] | Height [mAU] | Area % |
|---|---|---|---|---|---|---|
| 1 | 7.514 | MM | 0.2294 | 1892.63684 | 137.52380 | 94.5139 |
| 2 | 9.877 | PM | 0.2707 | 109.85832 | 6.76342 | 5.4861 |

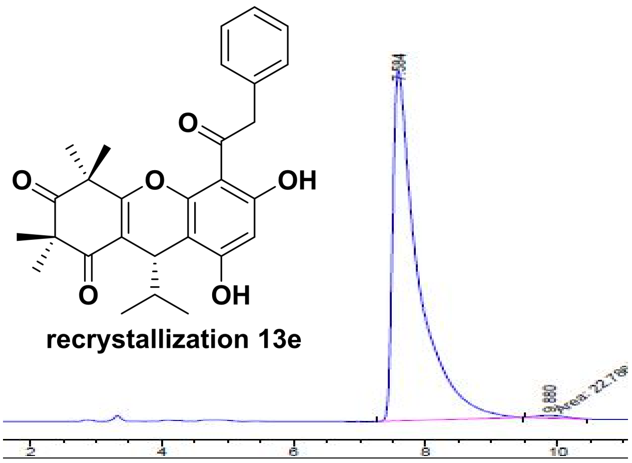


Signal 1: DAD1 G, Sig=280,4 Ref=360,100

| Peak # | RetTime [min] | Type | Width [min] | Area [mAU*s] | Height [mAU] | Area % |
|---|---|---|---|---|---|---|
| 1 | 7.584 | BB | 0.3708 | 2758.43872 | 102.89391 | 99.1807 |
| 2 | 9.880 | MP | 0.4772 | 22.78646 | 7.95888e-1 | 0.8193 |

**13f:** HPLC analysis: Daicel Chiralpak OD-H column; *n*-hexane/*i*-propanol = 90:10, 1 mL/min, $\lambda$ = 280 nm; major enantiomer: $t_R$ = 4.6 min, minor enantiomer: $t_R$ = 6.8 min. 94:6 *er*.

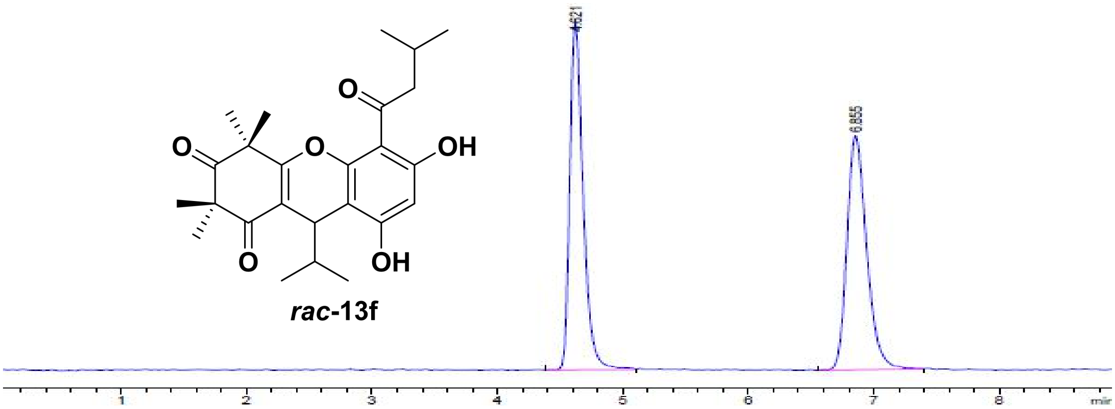


| Peak # | RetTime [min] | Type | Width [min] | Area [mAU*s] | Height [mAU] | Area % |
|---|---|---|---|---|---|---|
| 1 | 4.621 | BV | 0.1121 | 1504.49805 | 203.83351 | 50.1442 |
| 2 | 6.855 | BB | 0.1629 | 1495.84729 | 137.56551 | 49.8558 |

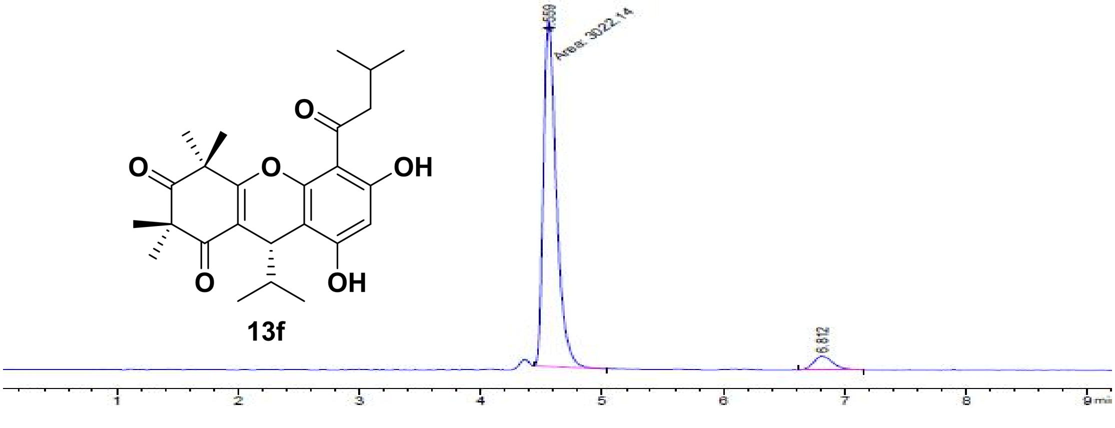

| Peak # | RetTime [min] | Type | Width [min] | Area [mAU*s] | Height [mAU] | Area % |
|---|---|---|---|---|---|---|
| 1 | 4.559 | MM | 0.1234 | 3022.14331 | 408.17206 | 94.2630 |
| 2 | 6.812 | VV | 0.1730 | 183.93231 | 16.38622 | 5.7370 |

**13g:** HPLC analysis: Daicel Chiralpak OD-H column; *n*-hexane/*i*-propanol = 90:10, 1 mL/min, $\lambda$ = 280 nm; major enantiomer: $t_R$ = 4.5 min, minor enantiomer: $t_R$ = 6.7 min. 94:6 *er*, re-crystallised: >99.5:0.5 *er*.

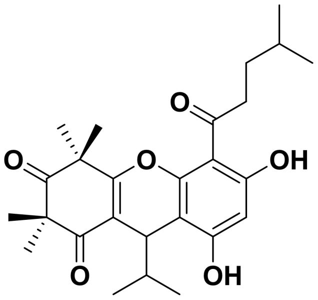


***rac*-13g**

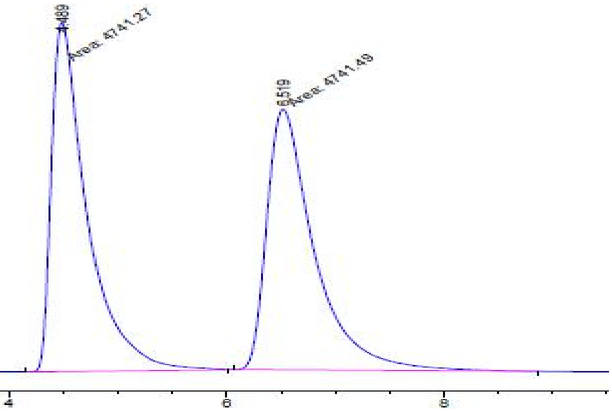


| Peak # | RetTime [min] | Type | Width [min] | Area [mAU*s] | Height [mAU] | Area % |
|---|---|---|---|---|---|---|
| 1 | 4.489 | MM | 0.3784 | 4741.27295 | 208.85277 | 49.9989 |
| 2 | 6.519 | MP | 0.5062 | 4741.48975 | 156.11006 | 50.0011 |

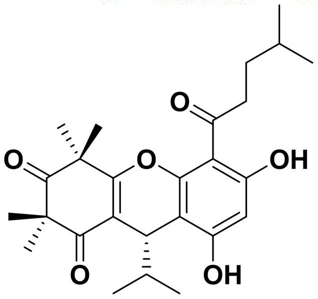


**13g**

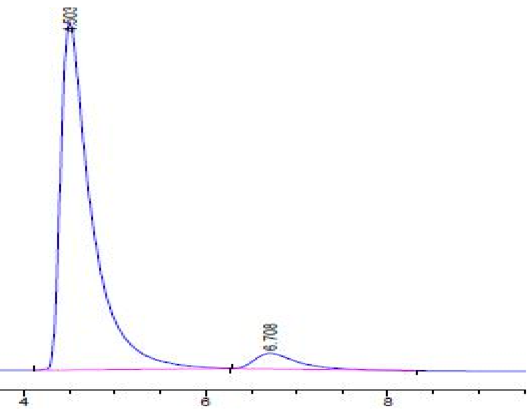


| Peak # | RetTime [min] | Type | Width [min] | Area [mAU*s] | Height [mAU] | Area % |
|---|---|---|---|---|---|---|
| 1 | 4.503 | BB | 0.3433 | 4405.78564 | 185.37852 | 94.2229 |
| 2 | 6.708 | BB | 0.4860 | 270.13196 | 8.29392 | 5.7771 |

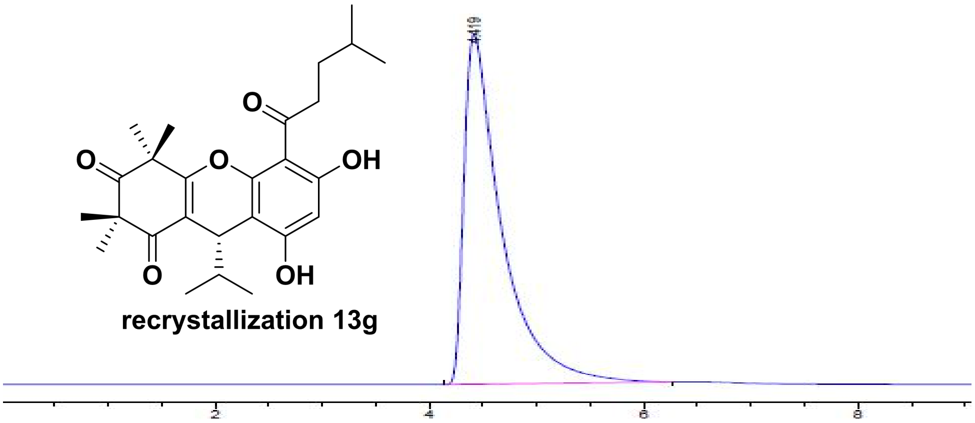


Signal 1: DAD1 G, Sig=280,4 Ref=360,100

| Peak # | RetTime [min] | Type | Width [min] | Area [mAU*s] | Height [mAU] | Area % |
|---|---|---|---|---|---|---|
| 1 | 4.419 | BB | 0.3509 | 8287.19727 | 339.40604 | 100.0000 |

**13h:** HPLC analysis: Daicel Chiralpak IE-3 column; *n*-hexane/*i*-propanol = 95:5, 1 mL/min, $\lambda$ = 280 nm; major enantiomer: $t_R$ = 12.0 min, minor enantiomer: $t_R$ = 8.9 min. 95.5:4.5 *er*, re-crystallised: 99:1 *er*.

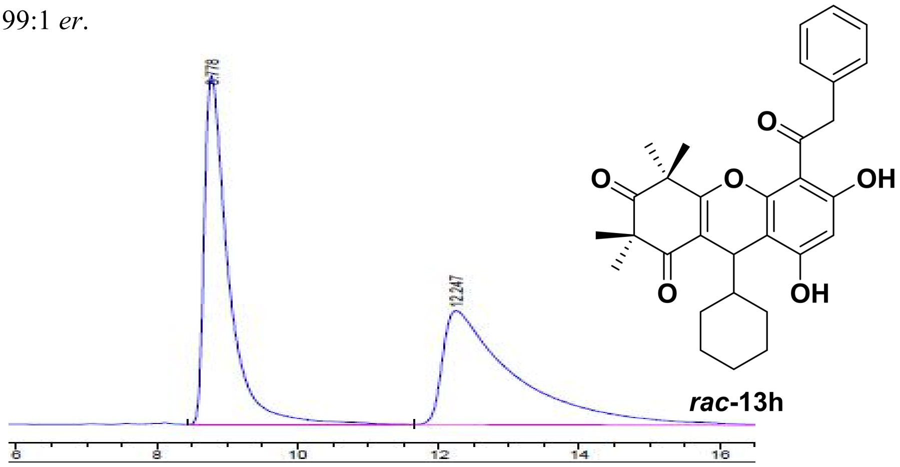


| Peak # | RetTime [min] | Type | Width [min] | Area [mAU*s] | Height [mAU] | Area % |
|---|---|---|---|---|---|---|
| 1 | 8.778 | BB | 0.3499 | 3708.04785 | 155.67624 | 50.6769 |
| 2 | 12.247 | BBA | 0.9509 | 3608.98560 | 50.79818 | 49.3231 |

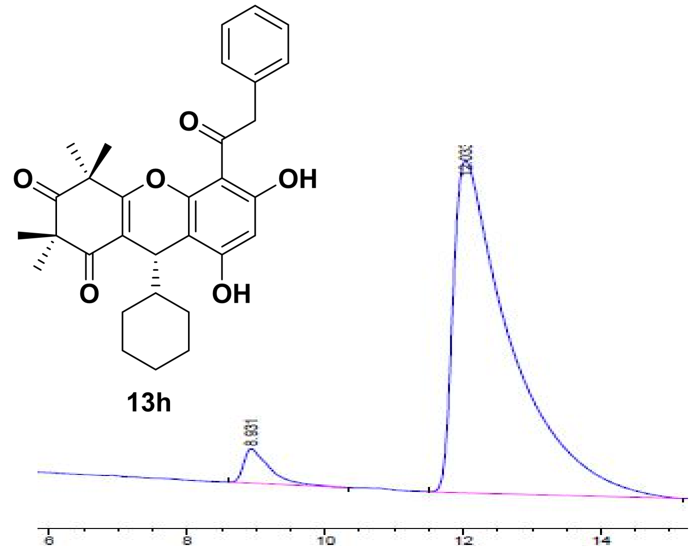


| Peak # | RetTime [min] | Type | Width [min] | Area [mAU*s] | Height [mAU] | Area % |
|---|---|---|---|---|---|---|
| 1 | 8.931 | BB | 0.3581 | 120.50346 | 4.65311 | 4.4160 |
| 2 | 12.033 | BB | 0.8042 | 2608.29419 | 45.00141 | 95.5840 |

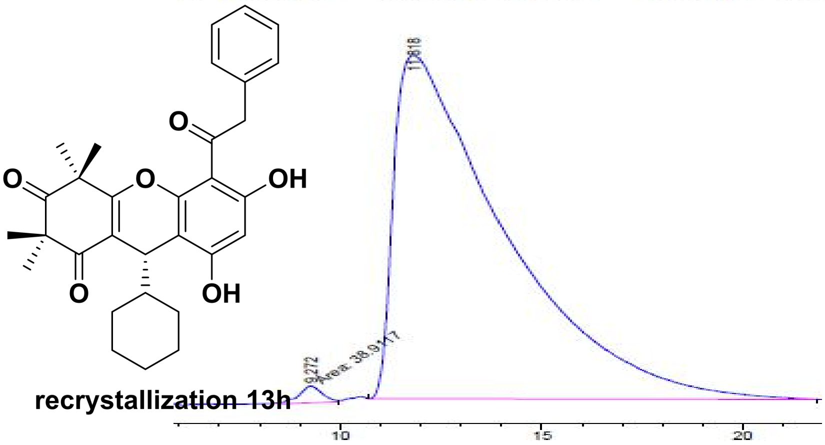


| Peak # | RetTime [min] | Type | Width [min] | Area [mAU*s] | Height [mAU] | Area % |
|---|---|---|---|---|---|---|
| 1 | 9.272 | MM | 0.6685 | 38.91173 | 9.70192e-1 | 1.0108 |
| 2 | 11.818 | BB | 2.4268 | 3810.82104 | 19.84101 | 98.9892 |

**13i:** HPLC analysis: Daicel Chiralpak IE-3 column; *n*-hexane/*i*-propanol = 95:5, 1 mL/min, $\lambda$ = 280 nm; major enantiomer: $t_R$ = 9.2 min, minor enantiomer: $t_R$ = 7.0 min. 97:3 *er*, re-crystallised: 99:1 *er*.

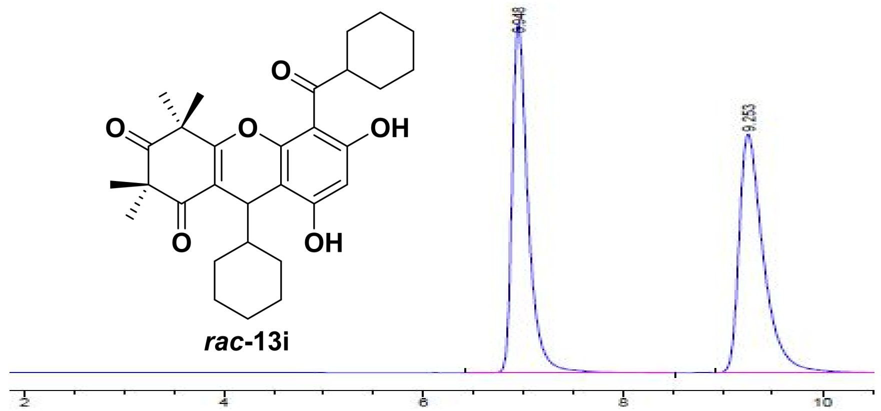


| Peak # | RetTime [min] | Type | Width [min] | Area [mAU*s] | Height [mAU] | Area % |
|---|---|---|---|---|---|---|
| 1 | 6.948 | BB | 0.1657 | 1216.80664 | 109.44376 | 48.9101 |
| 2 | 9.253 | BB | 0.2543 | 1271.03687 | 74.78799 | 51.0899 |

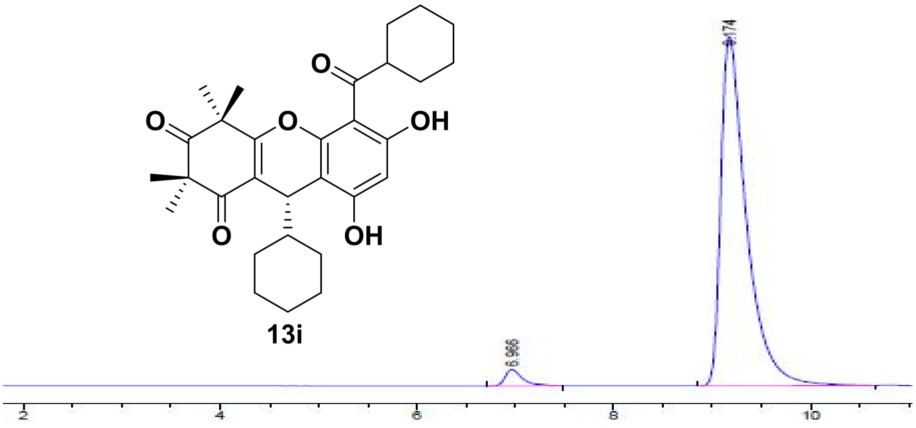


| Peak # | RetTime [min] | Type | Width [min] | Area [mAU*s] | Height [mAU] | Area % |
|---|---|---|---|---|---|---|
| 1 | 6.966 | BB | 0.1791 | 74.82505 | 6.18860 | 3.0058 |
| 2 | 9.174 | BB | 0.2669 | 2414.51904 | 133.57817 | 96.9942 |

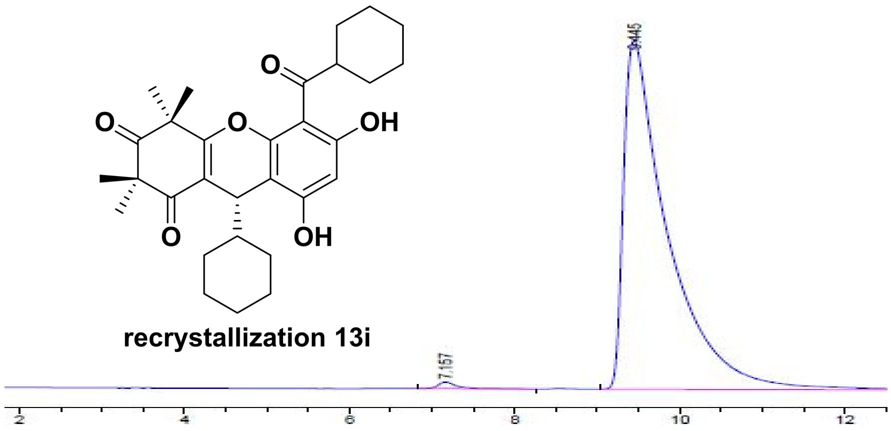


| Peak # | RetTime [min] | Type | Width [min] | Area [mAU*s] | Height [mAU] | Area % |
|---|---|---|---|---|---|---|
| 1 | 7.157 | BB | 0.2285 | 35.60913 | 2.27665 | 0.7739 |
| 2 | 9.445 | BBA | 0.5228 | 4565.48779 | 121.95530 | 99.2261 |

**13j:** HPLC analysis: Daicel Chiralpak IE-3 column; *n*-hexane/*i*-propanol = 95:5, 1 mL/min, $\lambda$ = 280 nm; major enantiomer: $t_R$ = 8.5 min, minor enantiomer: $t_R$ = 6.7 min. 94:6 *er*, re-crystallised: 99:1 *er*.

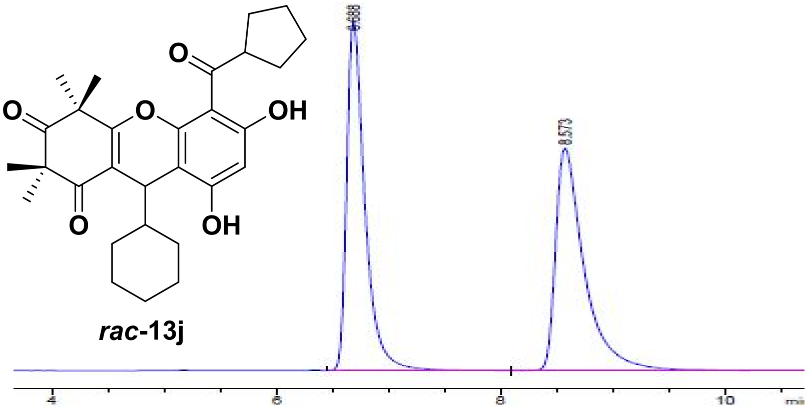


| Peak # | RetTime [min] | Type | Width [min] | Area [mAU*s] | Height [mAU] | Area % |
|---|---|---|---|---|---|---|
| 1 | 6.688 | BB | 0.1706 | 2945.67603 | 259.21820 | 49.9042 |
| 2 | 8.573 | BBA | 0.2648 | 2956.97998 | 165.20952 | 50.0958 |

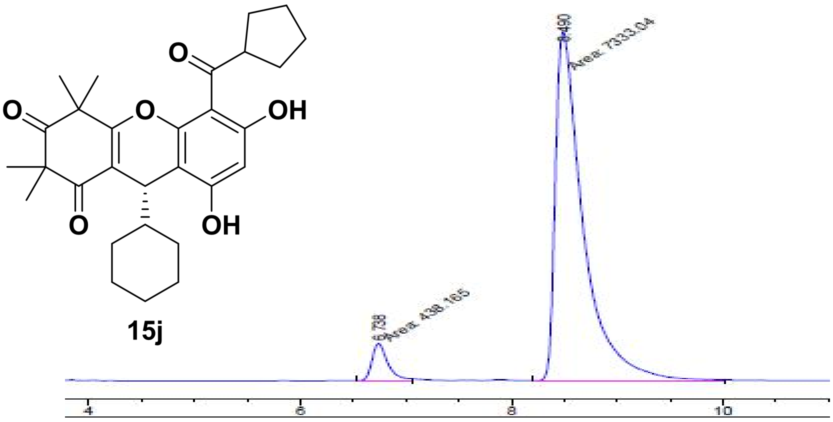


| Peak # | RetTime [min] | Type | Width [min] | Area [mAU*s] | Height [mAU] | Area % |
|---|---|---|---|---|---|---|
| 1 | 6.738 | MF | 0.1718 | 438.16470 | 42.51755 | 5.6383 |
| 2 | 8.490 | MF | 0.3065 | 7333.03564 | 398.74600 | 94.3617 |

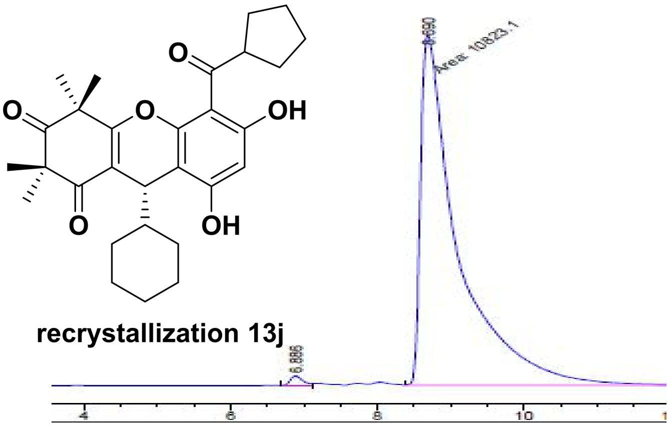

Signal 1: DAD1 G, Sig=280,4 Ref=360,100

| Peak # | RetTime [min] | Type | Width [min] | Area [mAU*s] | Height [mAU] | Area % |
|---|---|---|---|---|---|---|
| 1 | 6.886 | BV | 0.1722 | 95.26072 | 8.28332 | 0.8725 |
| 2 | 8.690 | MF | 0.5942 | 1.08231e4 | 303.59097 | 99.1275 |

**13k:** HPLC analysis: Daicel Chiralpak IE-3 column; *n*-hexane/*i*-propanol = 95:5, 1 mL/min, $\lambda$ = 280 nm; major enantiomer: $t_R$ = 8.1 min, minor enantiomer: $t_R$ = 6.7 min. 93:7 *er*.

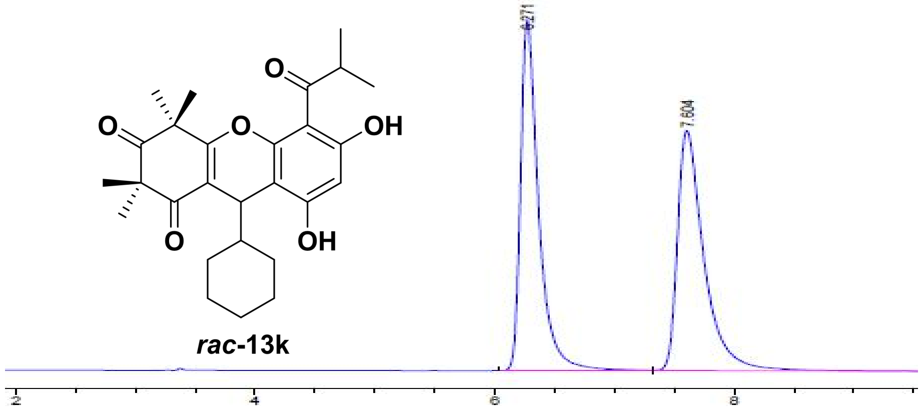


| Peak # | RetTime [min] | Type | Width [min] | Area [mAU*s] | Height [mAU] | Area % |
|---|---|---|---|---|---|---|
| 1 | 6.271 | BB | 0.1568 | 1329.09070 | 126.27782 | 49.9803 |
| 2 | 7.604 | BB | 0.2270 | 1330.13831 | 86.71401 | 50.0197 |

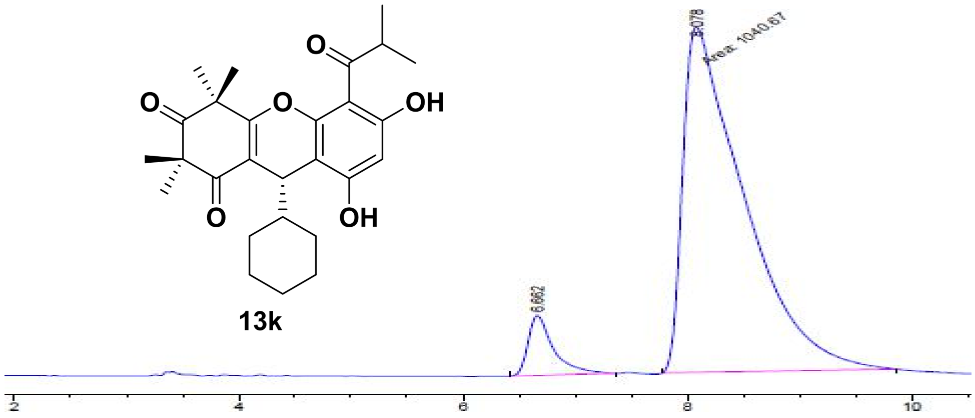


| Peak # | RetTime [min] | Type | Width [min] | Area [mAU*s] | Height [mAU] | Area % |
|---|---|---|---|---|---|---|
| 1 | 6.662 | BB | 0.2282 | 74.33395 | 4.76084 | 6.6667 |
| 2 | 8.078 | PP | 0.6312 | 1040.66638 | 27.47929 | 93.3333 |

**13l:** HPLC analysis: Daicel Chiralpak IE-3 column; *n*-hexane/*i*-propanol = 95:5, 1 mL/min, $\lambda$ = 280 nm; major enantiomer: $t_R$ = 8.6 min, minor enantiomer: $t_R$ = 6.2 min. 96.5:3.5 *er*, re-crystallised: 99.5:0.5 *er*.

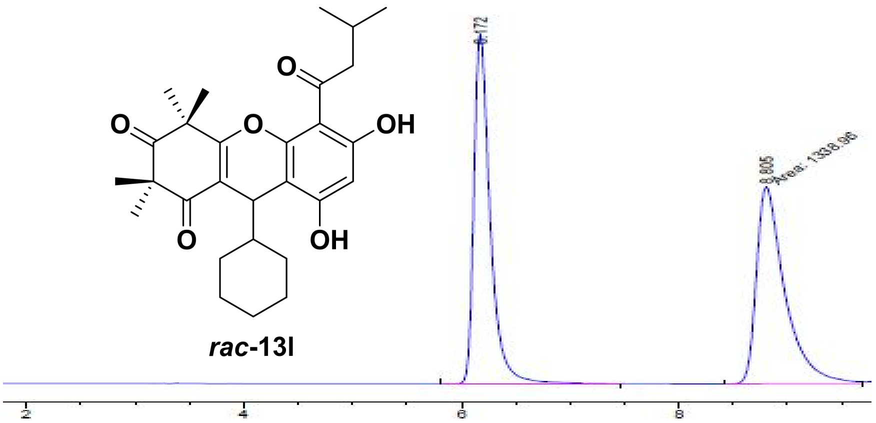


| Peak # | RetTime [min] | Type | Width [min] | Area [mAU*s] | Height [mAU] | Area % |
|---|---|---|---|---|---|---|
| 1 | 6.172 | BB | 0.1558 | 1357.41248 | 129.99460 | 50.3422 |
| 2 | 8.805 | MF | 0.3043 | 1338.96106 | 73.32545 | 49.6578 |

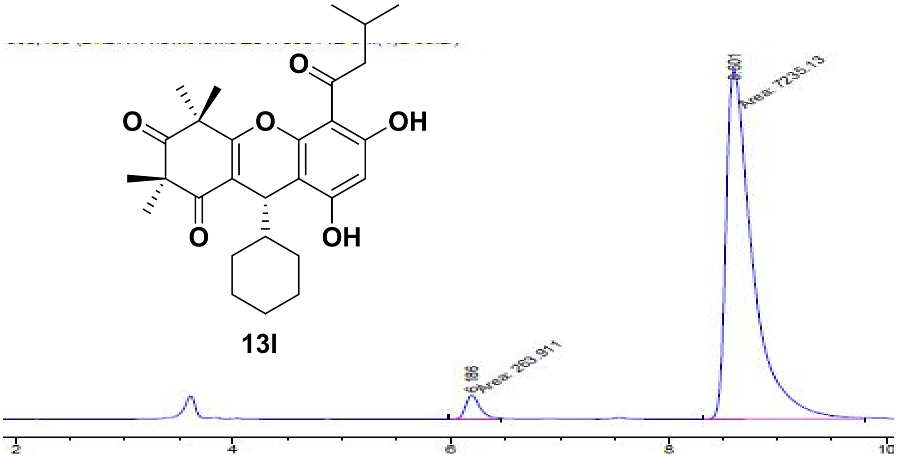


| Peak # | RetTime [min] | Type | Width [min] | Area [mAU*s] | Height [mAU] | Area % |
|---|---|---|---|---|---|---|
| 1 | 6.186 | MF | 0.1528 | 263.91138 | 28.79164 | 3.5193 |
| 2 | 8.601 | MF | 0.2851 | 7235.13330 | 423.00006 | 96.4807 |

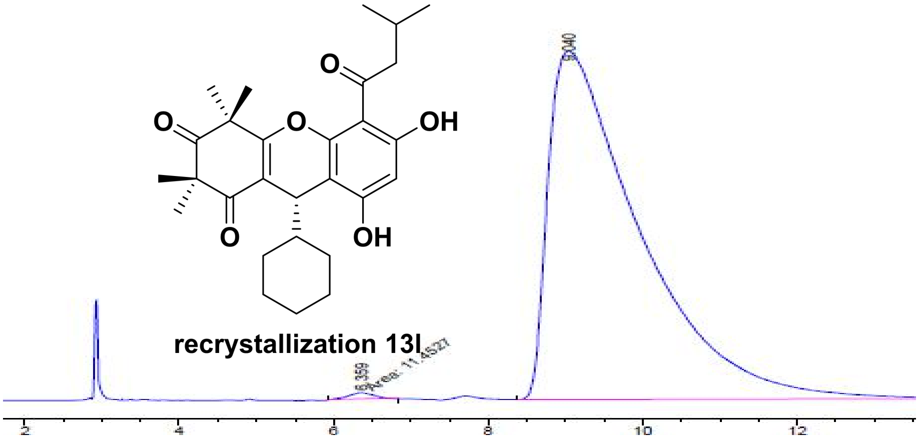

| Peak # | RetTime [min] | Type | Width [min] | Area [mAU*s] | Height [mAU] | Area % |
|---|---|---|---|---|---|---|
| 1 | 6.359 | MM | 0.3704 | 11.45273 | 5.15359e-1 | 0.4505 |
| 2 | 9.040 | BB | 1.1717 | 2530.69141 | 30.51147 | 99.5495 |

**13m:** HPLC analysis: Daicel Chiralpak IE-3 column; *n*-hexane/*i*-propanol = 95:5, 1 mL/min, $\lambda$ = 280 nm; major enantiomer: $t_R$ = 8.6 min, minor enantiomer: $t_R$ = 6.3 min. 97:3 *er*, re-crystallised: 99:1 *er*.

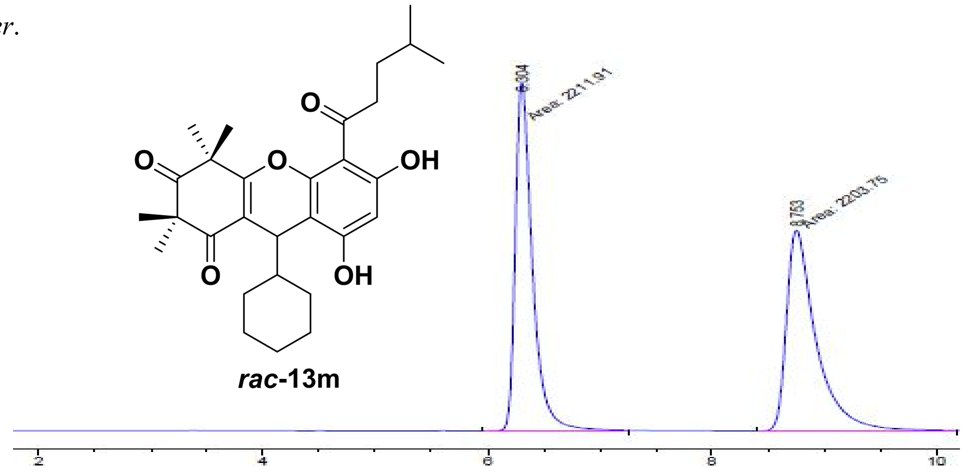


| Peak # | RetTime [min] | Type | Width [min] | Area [mAU*s] | Height [mAU] | Area % |
|---|---|---|---|---|---|---|
| 1 | 6.304 | MF | 0.1773 | 2211.90576 | 207.88939 | 50.0924 |
| 2 | 8.753 | MF | 0.3073 | 2203.74805 | 119.53747 | 49.9076 |

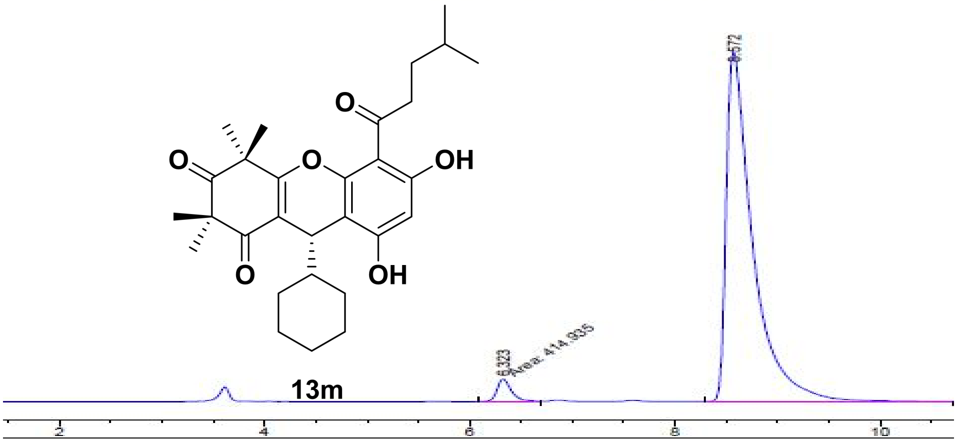


| Peak # | RetTime [min] | Type | Width [min] | Area [mAU*s] | Height [mAU] | Area % |
|---|---|---|---|---|---|---|
| 1 | 6.323 | PM | 0.1602 | 414.93506 | 43.16123 | 3.2951 |
| 2 | 8.572 | BB | 0.2611 | 1.21774e4 | 673.06976 | 96.7049 |

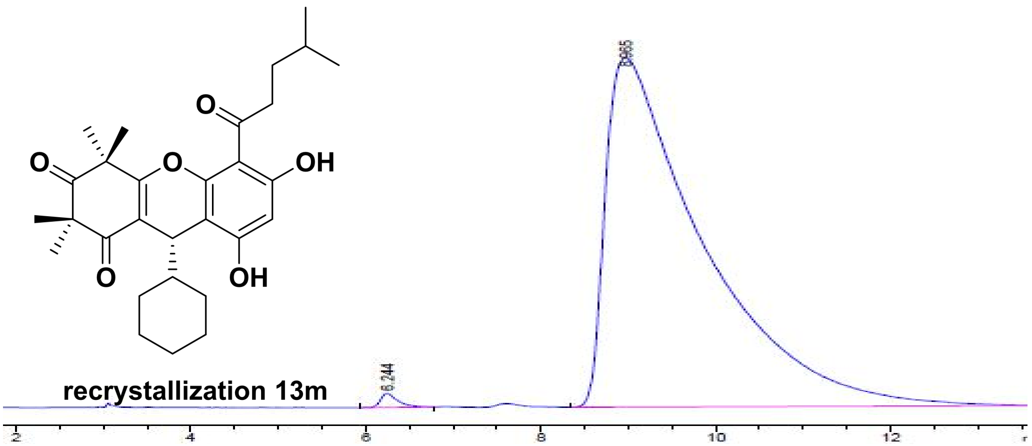


Signal 1: DAD1 G, Sig=280,4 Ref=360,100

| Peak # | RetTime [min] | Type | Width [min] | Area [mAU*s] | Height [mAU] | Area % |
|---|---|---|---|---|---|---|
| 1 | 6.244 | BB | 0.2036 | 34.97317 | 2.52505 | 0.7059 |
| 2 | 8.965 | BBA | 1.0698 | 4919.57080 | 63.36427 | 99.2941 |

**13n:** HPLC analysis: Daicel Chiralpak IE-3 column; *n*-hexane/*i*-propanol = 95:5, 1 mL/min, $\lambda$ = 280 nm; major enantiomer: $t_R$ = 7.2 min, minor enantiomer: $t_R$ = 5.9 min. 90.5:9.5 *er*.

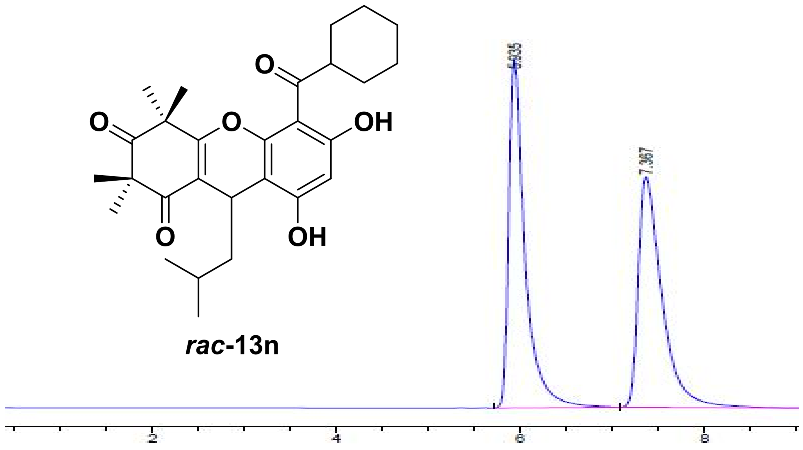


| Peak # | RetTime [min] | Type | Width [min] | Area [mAU*s] | Height [mAU] | Area % |
|---|---|---|---|---|---|---|
| 1 | 5.935 | BB | 0.1800 | 1791.29395 | 145.15936 | 50.9788 |
| 2 | 7.367 | BB | 0.2675 | 1722.50671 | 95.92169 | 49.0212 |

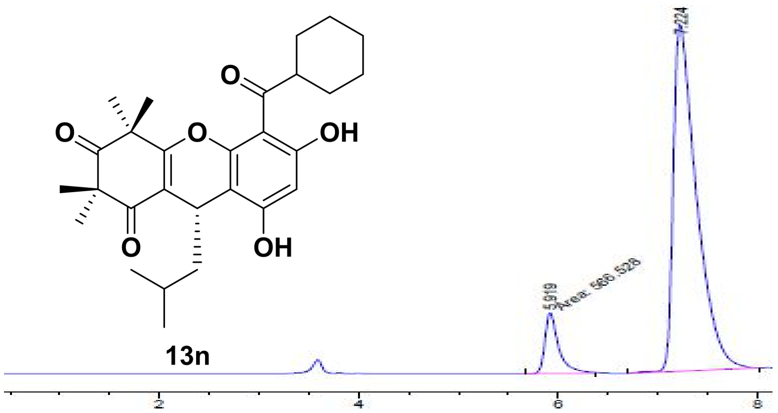


| Peak # | RetTime [min] | Type | Width [min] | Area [mAU*s] | Height [mAU] | Area % |
|---|---|---|---|---|---|---|
| 1 | 5.919 | MF | 0.1614 | 566.52765 | 58.48677 | 9.4755 |
| 2 | 7.224 | BB | 0.2355 | 5412.36768 | 333.21771 | 90.5245 |

**13o:** HPLC analysis: Daicel Chiralpak OD-H column; *n*-hexane/*i*-propanol = 90:10, 1 mL/min, $\lambda$ = 280 nm; major enantiomer: $t_R$ = 4.8 min, minor enantiomer: $t_R$ = 7.0 min. 95:5 *er*, re-crystallised: 99.5:0.5 *er*.

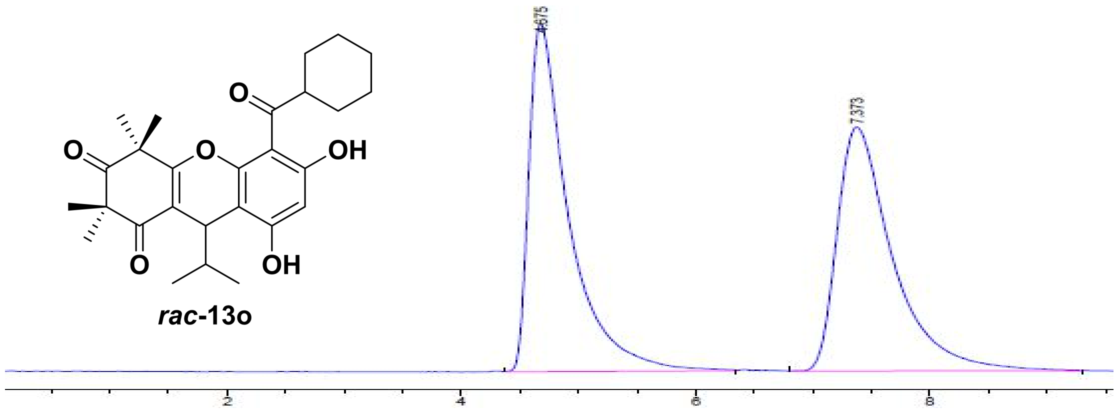


| Peak # | RetTime [min] | Type | Width [min] | Area [mAU*s] | Height [mAU] | Area % |
|---|---|---|---|---|---|---|
| 1 | 4.675 | VV | 0.3434 | 7197.76855 | 302.72830 | 50.6216 |
| 2 | 7.373 | BV | 0.4875 | 7020.98975 | 212.49187 | 49.3784 |

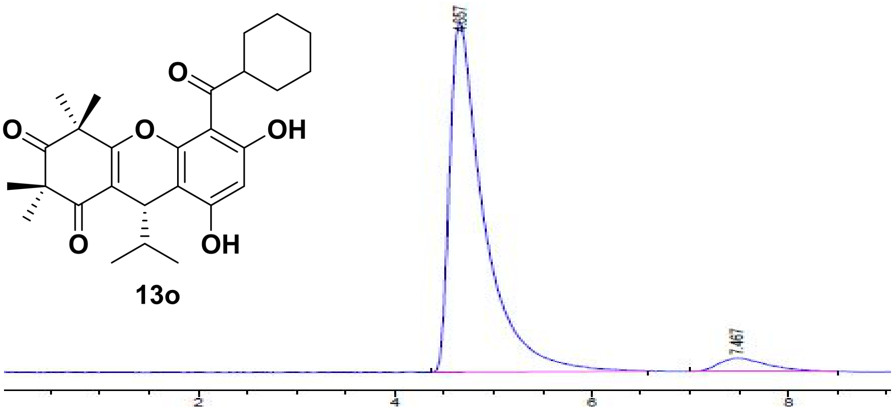


| Peak # | RetTime [min] | Type | Width [min] | Area [mAU*s] | Height [mAU] | Area % |
|---|---|---|---|---|---|---|
| 1 | 4.657 | BV | 0.3472 | 8671.30176 | 357.25836 | 94.9079 |
| 2 | 7.467 | BB | 0.4230 | 465.24008 | 13.40340 | 5.0921 |

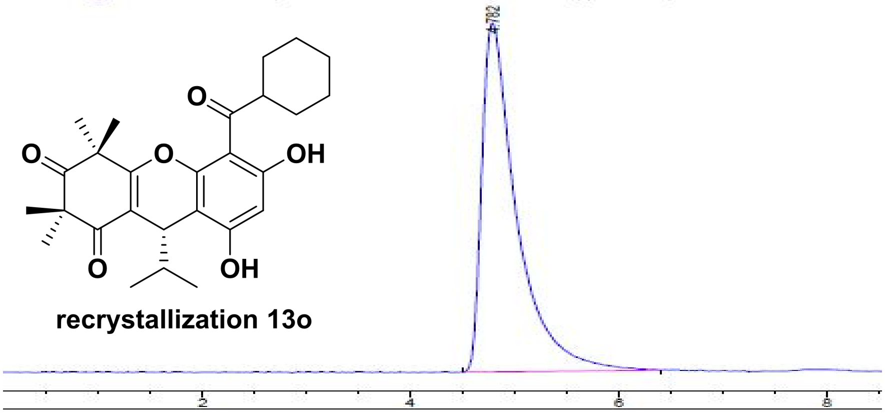


| Peak # | RetTime [min] | Type | Width [min] | Area [mAU*s] | Height [mAU] | Area % |
|---|---|---|---|---|---|---|
| 1 | 4.782 | BB | 0.3306 | 5167.88818 | 221.18550 | 100.0000 |

**13p:** HPLC analysis: Daicel Chiralpak OD-H column; *n*-hexane/*i*-propanol = 90:10, 1 mL/min, $\lambda$ = 280 nm; major enantiomer: $t_R$ = 4.8 min, minor enantiomer: $t_R$ = 7.0 min. 94.5:5.5 *er*, re-crystallised: 99:1 *er*.

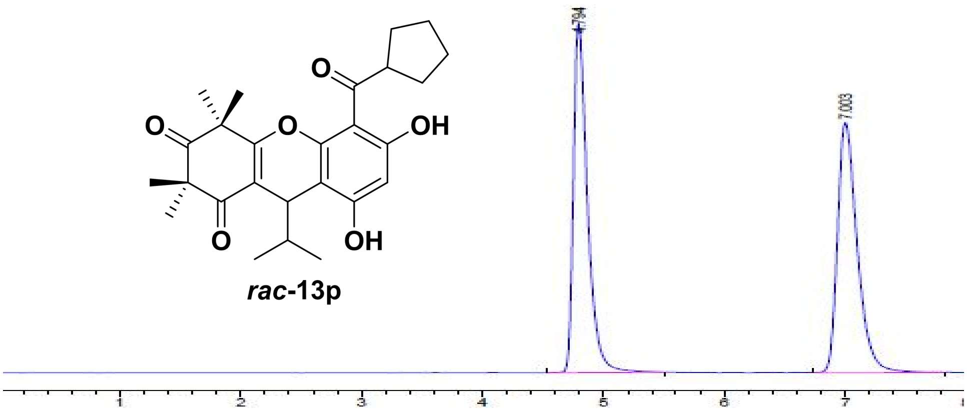


*rac*-13p

| Peak # | RetTime [min] | Type | Width [min] | Area [mAU*s] | Height [mAU] | Area % |
|---|---|---|---|---|---|---|
| 1 | 4.794 | BB | 0.1203 | 4542.59863 | 562.28375 | 49.8141 |
| 2 | 7.003 | VV | 0.1725 | 4576.51123 | 402.84271 | 50.1859 |

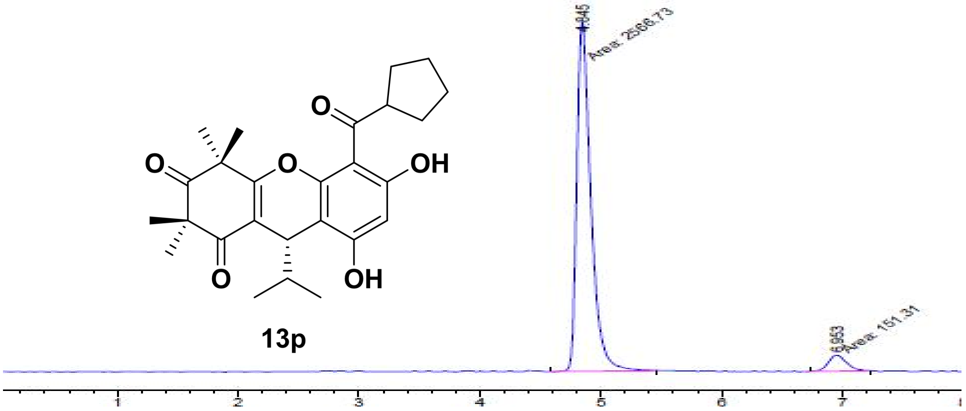


13p

| Peak # | RetTime [min] | Type | Width [min] | Area [mAU*s] | Height [mAU] | Area % |
|---|---|---|---|---|---|---|
| 1 | 4.845 | PM | 0.1350 | 2566.73291 | 316.89755 | 94.4331 |
| 2 | 6.953 | MP | 0.1735 | 151.30992 | 14.53629 | 5.5669 |

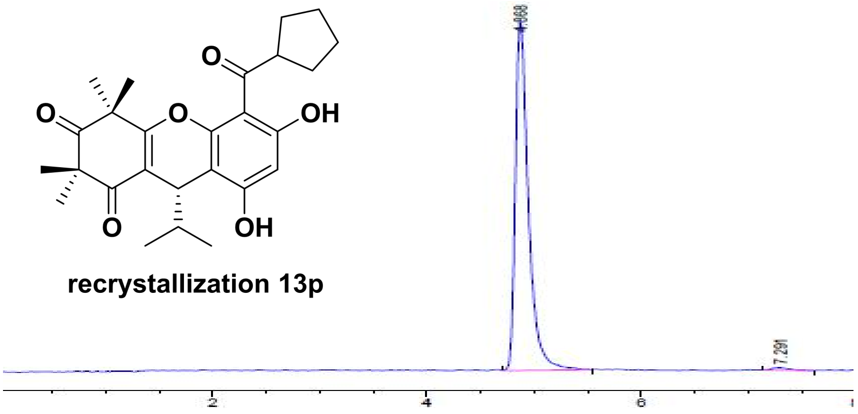


recrystallization 13p

| Peak # | RetTime [min] | Type | Width [min] | Area [mAU*s] | Height [mAU] | Area % |
|---|---|---|---|---|---|---|
| 1 | 4.868 | VV | 0.1291 | 2101.52588 | 247.45172 | 98.9610 |
| 2 | 7.291 | BB | 0.1423 | 22.06454 | 1.91670 | 1.0390 |

**13q:** HPLC analysis: Daicel Chiralpak OD-H column; *n*-hexane/*i*-propanol = 90:10, 1 mL/min, $\lambda$ = 280 nm; major enantiomer: $t_R$ = 5.1 min, minor enantiomer: $t_R$ = 7.0 min. 93:7 *er*, re-crystallised: 99.5:0.5 *er*.

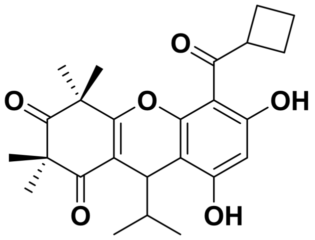


***rac*-13q**

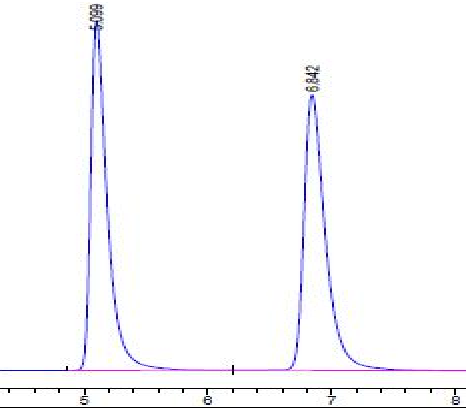


| Peak # | RetTime [min] | Type | Width [min] | Area [mAU*s] | Height [mAU] | Area % |
|---|---|---|---|---|---|---|
| 1 | 5.099 | BB | 0.1405 | 2179.85840 | 230.16489 | 49.8451 |
| 2 | 6.842 | BB | 0.1809 | 2193.40259 | 181.62161 | 50.1549 |

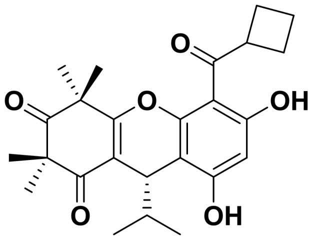


**13q**

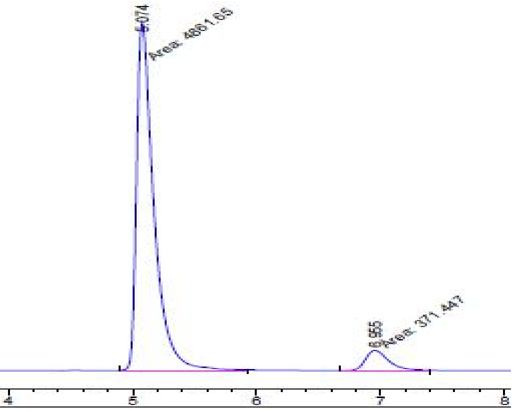


| Peak # | RetTime [min] | Type | Width [min] | Area [mAU*s] | Height [mAU] | Area % |
|---|---|---|---|---|---|---|
| 1 | 5.074 | MF | 0.1640 | 4861.65430 | 494.00443 | 92.9020 |
| 2 | 6.955 | MF | 0.2152 | 371.44678 | 28.76166 | 7.0980 |

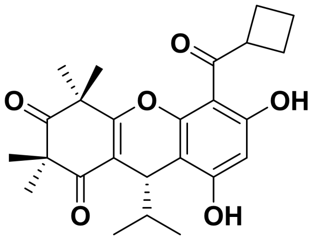


**recrystallization 13q**

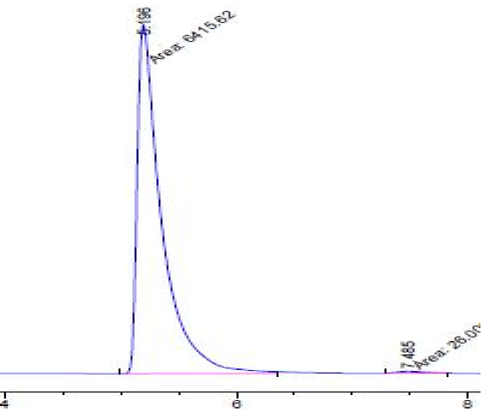

| Peak # | RetTime [min] | Type | Width [min] | Area [mAU*s] | Height [mAU] | Area % |
|---|---|---|---|---|---|---|
| 1 | 5.196 | MF | 0.2448 | 6415.61963 | 436.71884 | 99.5962 |
| 2 | 7.485 | MM | 0.2296 | 26.00991 | 1.88834 | 0.4038 |

**13r:** HPLC analysis: Daicel Chiralpak AD-H column; *n*-hexane/*i*-propanol = 95:5, 1 mL/min, $\lambda$ = 280 nm; major enantiomer: $t_R$ = 6.3 min, minor enantiomer: $t_R$ = 4.7 min. 92:8 *er*.

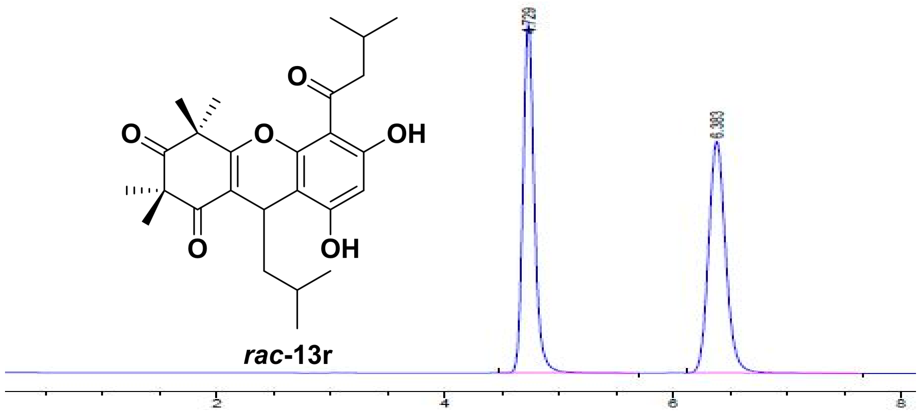


| Peak # | RetTime [min] | Type | Width [min] | Area [mAU*s] | Height [mAU] | Area % |
|---|---|---|---|---|---|---|
| 1 | 4.729 | BB | 0.1069 | 907.80579 | 130.85040 | 50.0705 |
| 2 | 6.383 | BB | 0.1593 | 905.24866 | 87.06976 | 49.9295 |

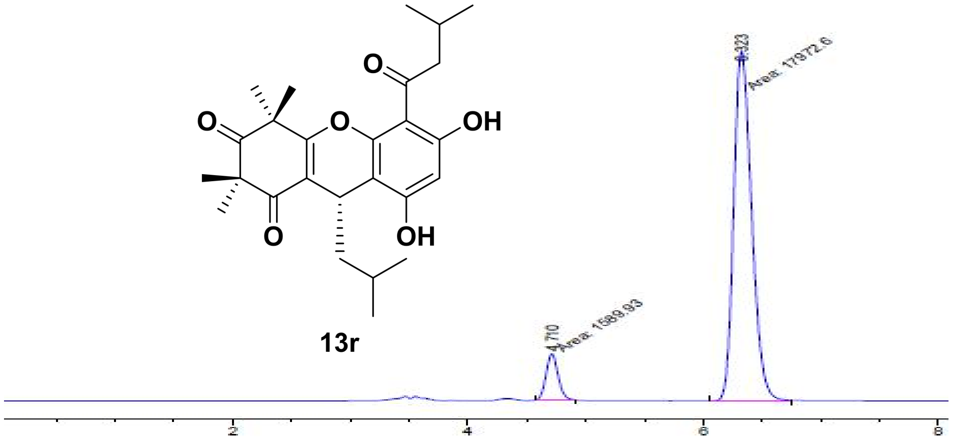


| Peak # | RetTime [min] | Type | Width [min] | Area [mAU*s] | Height [mAU] | Area % |
|---|---|---|---|---|---|---|
| 1 | 4.710 | PP | 0.1182 | 1589.92786 | 224.22241 | 8.1274 |
| 2 | 6.323 | MM | 0.1766 | 1.79726e4 | 1696.13452 | 91.8726 |

**13s:** HPLC analysis: Daicel Chiralpak IE-3 column; *n*-hexane/*i*-propanol = 95:5, 1 mL/min, $\lambda$ = 280 nm; major enantiomer: $t_R$ = 7.0 min, minor enantiomer: $t_R$ = 5.7 min. 93.5:6.5 *er*,

re-crystallised: 99.8:0.2 *er*.

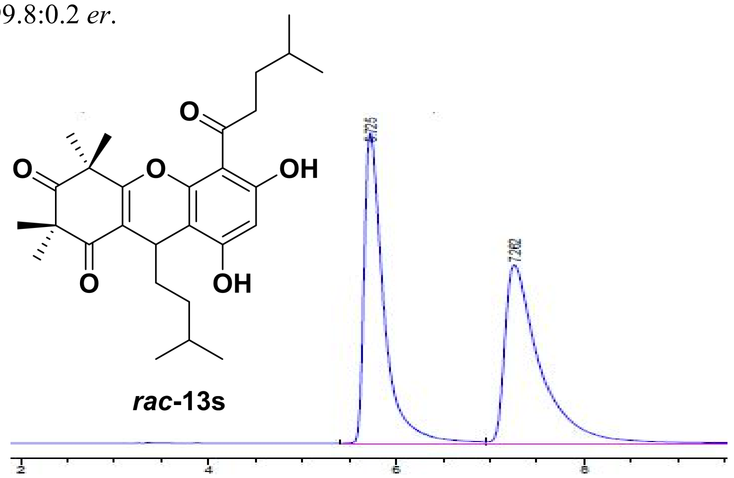


***rac*-13s**

| Peak # | RetTime [min] | Type | Width [min] | Area [mAU*s] | Height [mAU] | Area % |
|---|---|---|---|---|---|---|
| 1 | 5.725 | BV | 0.2161 | 2835.47876 | 194.49390 | 49.3670 |
| 2 | 7.262 | VB | 0.3751 | 2908.19214 | 111.92484 | 50.6330 |

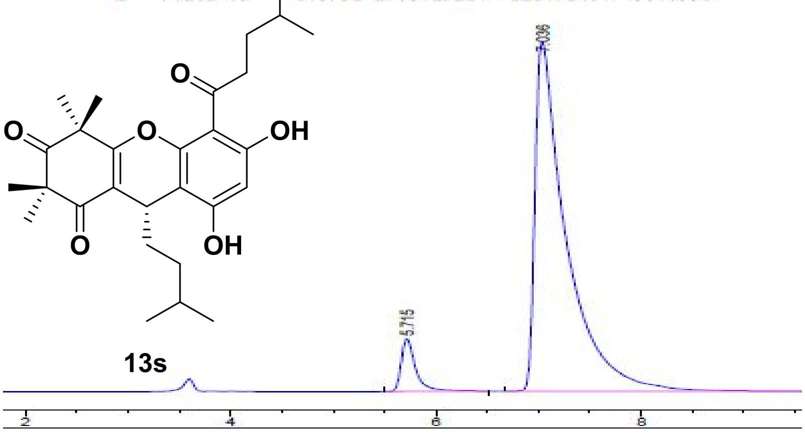


**13s**

| Peak # | RetTime [min] | Type | Width [min] | Area [mAU*s] | Height [mAU] | Area % |
|---|---|---|---|---|---|---|
| 1 | 5.715 | BB | 0.1440 | 817.94958 | 85.21327 | 6.3740 |
| 2 | 7.036 | BB | 0.2946 | 1.20146e4 | 563.84375 | 93.6260 |

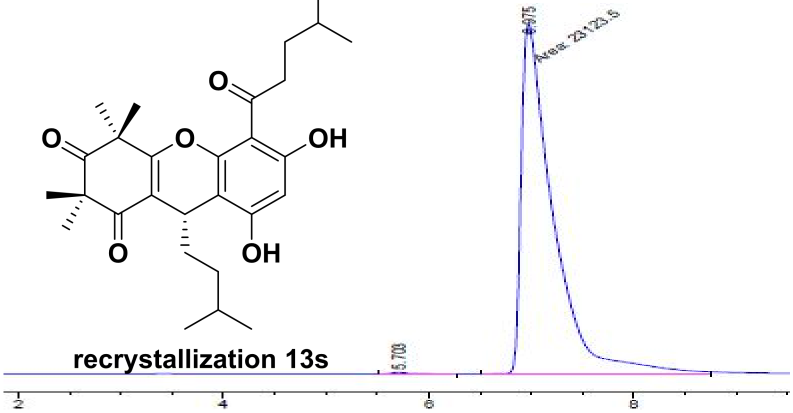


**recrystallization 13s**

| Peak # | RetTime [min] | Type | Width [min] | Area [mAU*s] | Height [mAU] | Area % |
|---|---|---|---|---|---|---|
| 1 | 5.703 | BB | 0.1443 | 36.60844 | 3.73553 | 0.1581 |
| 2 | 6.975 | MF | 0.3612 | 2.31235e4 | 1066.87305 | 99.8419 |

**13t:** HPLC analysis: Daicel Chiralpak IE-3 column; *n*-hexane/*i*-propanol = 95:5, 1 mL/min, $\lambda$ = 280 nm; major enantiomer: $t_R$ = 11.7 min, minor enantiomer: $t_R$ = 9.4 min. 95:5 *er*.

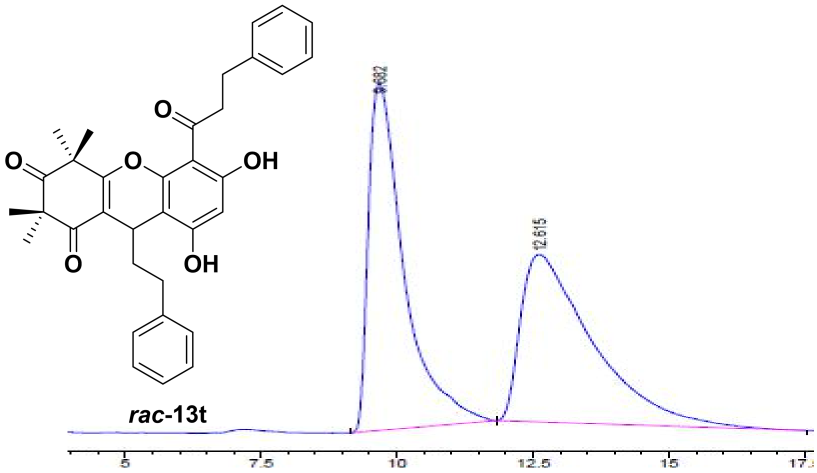


| Peak # | RetTime [min] | Type | Width [min] | Area [mAU*s] | Height [mAU] | Area % |
|---|---|---|---|---|---|---|
| 1 | 9.682 | BB | 0.6942 | 2361.10083 | 51.76268 | 50.5801 |
| 2 | 12.615 | BB | 1.2854 | 2306.94507 | 24.98071 | 49.4199 |

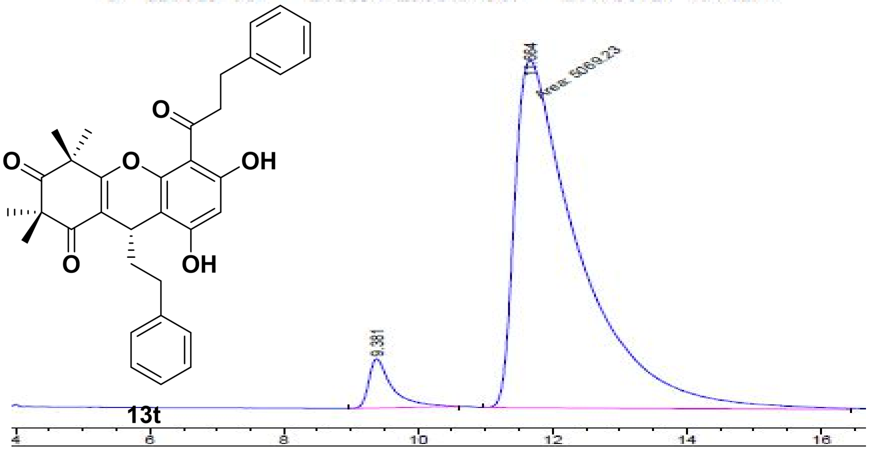


| Peak # | RetTime [min] | Type | Width [min] | Area [mAU*s] | Height [mAU] | Area % |
|---|---|---|---|---|---|---|
| 1 | 9.381 | BB | 0.3494 | 257.88089 | 10.69075 | 4.8409 |
| 2 | 11.664 | MF | 1.1195 | 5069.23096 | 75.46887 | 95.1591 |

**13u:** HPLC analysis: Daicel Chiralpak IE-3 column; *n*-hexane/*i*-propanol = 95:5, 1 mL/min, $\lambda$ = 280 nm; major enantiomer: $t_R$ = 9.5 min, minor enantiomer: $t_R$ = 7.3 min. 92.5:7.5 *er*, re-crystallised: 99.8:0.2 *er*.

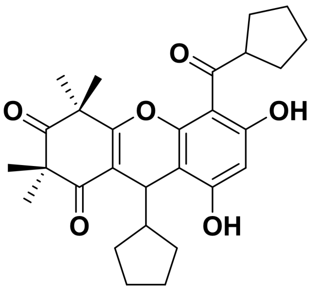


***rac*-13u**

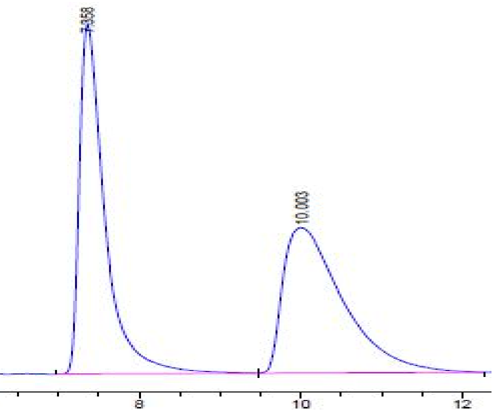


| Peak # | RetTime [min] | Type | Width [min] | Area [mAU*s] | Height [mAU] | Area % |
|---|---|---|---|---|---|---|
| 1 | 7.358 | BB | 0.3291 | 2188.54883 | 99.32778 | 50.6681 |
| 2 | 10.003 | BB | 0.7708 | 2130.83154 | 41.24342 | 49.3319 |

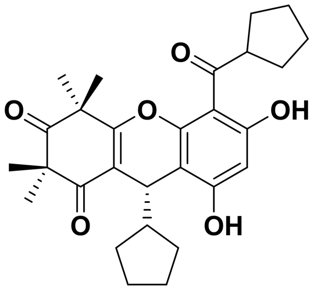


**13u**

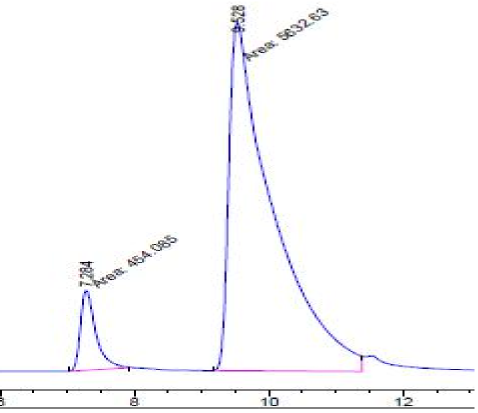


| Peak # | RetTime [min] | Type | Width [min] | Area [mAU*s] | Height [mAU] | Area % |
|---|---|---|---|---|---|---|
| 1 | 7.284 | PM | 0.2664 | 454.08542 | 28.40908 | 7.4603 |
| 2 | 9.528 | MF | 0.7586 | 5632.62500 | 123.74551 | 92.5397 |

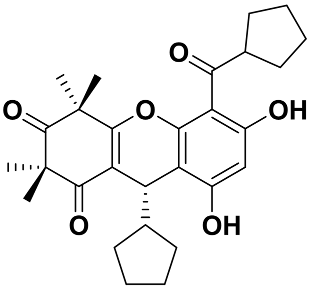


**recrystallization 13u**

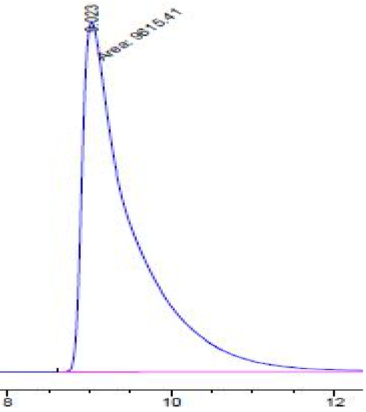


| Peak # | RetTime [min] | Type | Width [min] | Area [mAU*s] | Height [mAU] | Area % |
|---|---|---|---|---|---|---|
| 1 | 6.996 | BB | 0.2008 | 15.66588 | 1.16527 | 0.1627 |
| 2 | 9.023 | MF | 0.7021 | 9615.41211 | 228.25241 | 99.8373 |

**13v:** HPLC analysis: Daicel Chiralpak IE-3 column; *n*-hexane/*i*-propanol = 95:5, 1 mL/min, $\lambda$ = 280 nm; major enantiomer: $t_R$ = 9.7 min, minor enantiomer: $t_R$ = 7.3 min. 94:6 *er*, re-crystallised: 99:1 *er*.

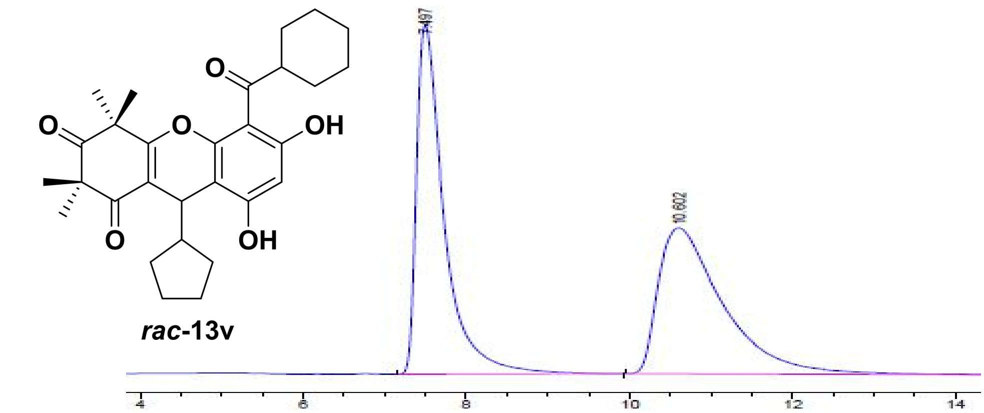


| Peak # | RetTime [min] | Type | Width [min] | Area [mAU*s] | Height [mAU] | Area % |
|---|---|---|---|---|---|---|
| 1 | 7.497 | BB | 0.3510 | 2324.41357 | 98.62396 | 50.4673 |
| 2 | 10.602 | BBA | 0.8214 | 2281.36963 | 41.23809 | 49.5327 |

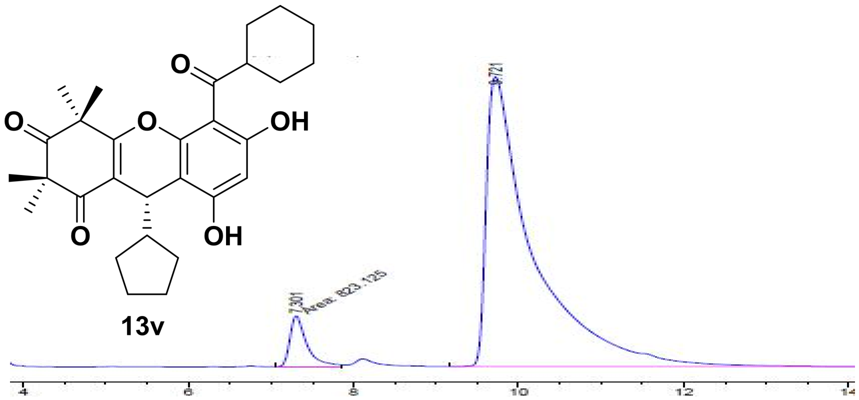


| Peak # | RetTime [min] | Type | Width [min] | Area [mAU*s] | Height [mAU] | Area % |
|---|---|---|---|---|---|---|
| 1 | 7.301 | MF | 0.2434 | 823.12469 | 56.35350 | 6.0970 |
| 2 | 9.721 | BBA | 0.5430 | 1.26773e4 | 322.22156 | 93.9030 |

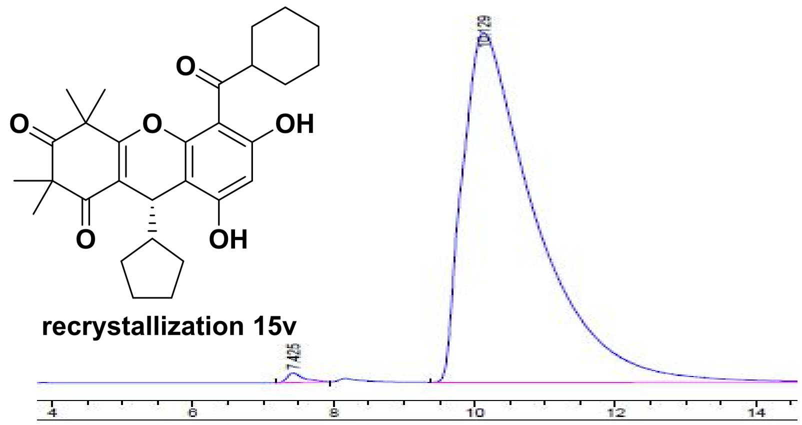

| Peak # | RetTime [min] | Type | Width [min] | Area [mAU*s] | Height [mAU] | Area % |
|---|---|---|---|---|---|---|
| 1 | 7.425 | BB | 0.2325 | 23.09551 | 1.42955 | 0.6084 |
| 2 | 10.129 | BB | 1.0065 | 3773.00244 | 52.12537 | 99.3916 |

**13w:** HPLC analysis: Daicel Chiralpak AD-H column; *n*-hexane/*i*-propanol = 95:5, 1 mL/min, $\lambda$ = 280 nm; major enantiomer: $t_R$ = 7.8 min, minor enantiomer: $t_R$ = 5.7 min. 89:11 *er*.

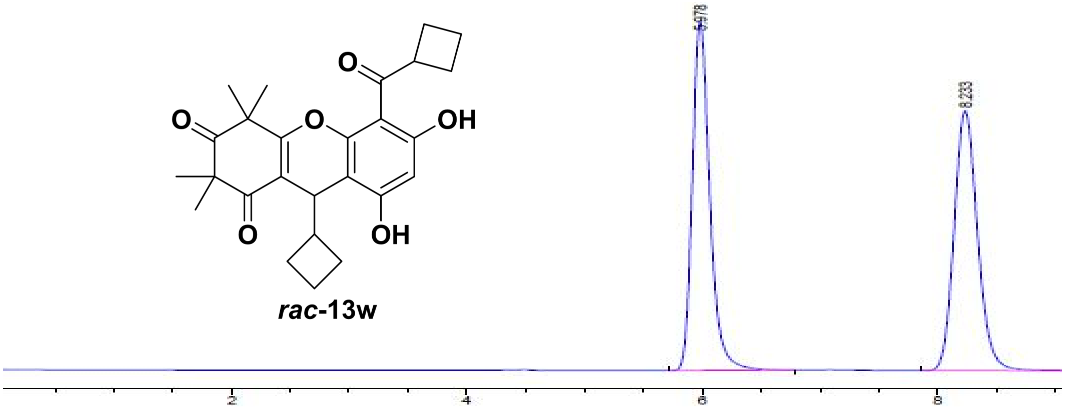


| Peak # | RetTime [min] | Type | Width [min] | Area [mAU*s] | Height [mAU] | Area % |
|---|---|---|---|---|---|---|
| 1 | 5.978 | BB | 0.1567 | 2163.22876 | 209.05998 | 49.9613 |
| 2 | 8.233 | BB | 0.2143 | 2166.57666 | 155.76677 | 50.0387 |

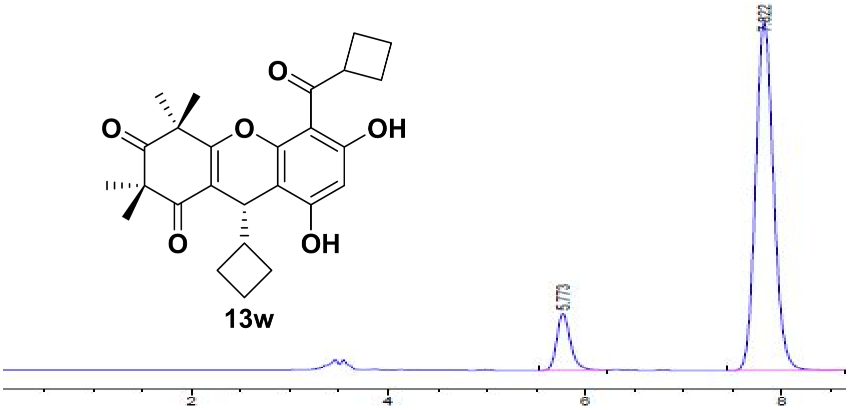


| Peak # | RetTime [min] | Type | Width [min] | Area [mAU*s] | Height [mAU] | Area % |
|---|---|---|---|---|---|---|
| 1 | 5.773 | BB | 0.1487 | 417.24973 | 43.20686 | 11.0122 |
| 2 | 7.822 | BB | 0.1962 | 3371.72388 | 265.44269 | 88.9878 |

## 6. Antibacterial activity assay of 2-7

### 6.1. Microorganisms

*Staphylococcus aureus* ATCC 29213 [methicillin-susceptible *S. aureus* (MSSA)], *Staphylococcus aureus* ATCC 33591 [methicillin-resistant *S. aureus* (MRSA)], *Staphylococcus aureus* ATCC 700699 [vancomycin-intermediate *S. aureus* (VISA)], *Enterococcus faecalis* ATCC 29212, *Enterococcus faecium* ATCC 700221 [vancomycin-resistant *E. faecium* (VRE)], *Staphylococcus epidermidis* ATCC 12228, *Klebsiella pneumoniae* ATCC 700603, *Klebsiella pneumoniae* ATCC BAA-2146 (a NDM-1-producing strain), *Pseudomonas aeruginosa* ATCC 27853, *Acinetobacter baumannii* ATCC 19606, *Escherichia coli* ATCC 25922 were standard isolates from ATCC (American Tissue Culture and Collection, Manassas, VA, USA).

### 6.2 Antimicrobial agents and medium

Five antibacterial agents including daptomycin, vancomycin, oxacillin, levofloxacin, and azithromycin were used in this study. All of them were purchased from National Institutes for Food and Drug Control, People's Republic of China. Cation-adjusted Mueller-Hinton (CAMH) broth was purchased from BD (Cockeysville, MD) and was prepared according to the recommendations of the Clinical and Laboratory Standards Institute (CLSI, formerly NCCLS)[9].

Stock solutions of antibiotics were prepared in solvents and diluents recommended by CLSI based on their actual purity or potency and sterilized through 0.22 μm filters before use. Test solutions with different concentrations of compounds and the antimicrobials were obtained by two-fold serial dilutions with CAMH broth.

CAMH broth was used for all susceptibility testing. Colony counts were determined using tryptic soy agar (TSA; BD, Cockeysville, MD) plates.

### 6.3 MIC determination

The minimum inhibitory concentrations (MICs) of the antibacterial agents for all strains were determined by broth microdilution method according to CLSI guidelines[10]. Wells of 96-well microtiter plates (Nunc, Thermo Fisher Scientific Inc., Roskilde) were inoculated with 100 $\mu$L of CAMH broth containing serial-diluted antimicrobials and a final inoculum of $5\times10^5$ CFU/mL. The

concentration ranges were from 0.06 to 128 $\mu$g/mL for each compounds, daptomycin, vancomycin, levofloxacin, and azithromycin, and from 0.25 to 512 $\mu$g/mL for oxacillin. The microtiter plates were incubated at 35˚C for 24 h. The MIC was defined as the lowest concentration of an antimicrobial agent that prevented turbidity. All MIC determinations were performed in duplicate.

### 6.4 In vitro antibacterial activities of 6 synthetic compounds

The antibacterial activities of 6 synthetic compounds against six Gram-positive and five Gram-negative strains were evaluated with MIC (Table S6-1). Among them, compound **7** exhibited significant antibacterial activity against all Gram-positive bacteria including three multiresistant strains (MRSA, VISA and VRE) with MIC values ranging from 0.25 to 2 $\mu$g/mL. Compounds (+)-**2**, (-)-**3**, and **4** exhibited moderate antibacterial activity against the Gram-positive bacteria with MIC values ranging from 8 to 64 $\mu$g/mL. The MICs of the antibiotics for the ATCC strains were within the expected ranges.

--------------------------------------------

[9] Clinical and Laboratory Standards Institute (**2015**) Performance standards for antimicrobial susceptibility testing; twenty-fifth informational supplement. CLSI document M100-S25. Wayne, Pennsylvania, USA.

[10] Clinical and Laboratory Standards Institute (**2012**) Methods for Dilution Antimicrobial Susceptibility Tests for Bacteria That Grow Aerobically; Approved Standard. CLSI document M07-A9. 9 ed. Wayne, Pennsylvania, USA.

Table S6-1. MICs of 6 synthetic synthetic compounds against six Gram-positive and five Gram-negative strains ($\mu$g/ml)

| compounds | Staphylococcus aureus subsp. aureus ATCC 29213 | Staphylococcus aureus subsp. aureus ATCC 33591 | Staphylococcus aureus subsp. aureus ATCC 700699 | Enterococcus faecalis ATCC 29212 | Enterococcus faecium ATCC 700221 | Staphylococcus epidermidis ATCC 12228 | Klebsiella subsp. pneumoniae ATCC 700603 | Klebsiella pneumoniae ATCC BAA-2146 | Pseudomonas aeruginosa ATCC 27853 | Acinetobacter baumannii ATCC 19606 | Escherichia coli ATCC 25922 |
|---|---|---|---|---|---|---|---|---|---|---|---|
| (+)-2 | 8 | 8 | 8 | 64 | 64 | 16 | ＞ 128 | ＞ 128 | ＞ 128 | ＞ 128 | ＞ 128 |
| (-)-3 | 16 | 16 | 16 | ＞ 128 | ＞ 128 | ＞ 128 | ＞ 128 | ＞ 128 | ＞ 128 | ＞ 128 | ＞ 128 |
| 4 | 16 | 16 | 32 | 64 | - | - | ＞ 128 | ＞ 128 | ＞ 128 | ＞ 128 | ＞ 128 |
| 5 | ＞ 128 | ＞ 128 | ＞ 128 | ＞ 128 | ＞ 128 | ＞ 128 | ＞ 128 | ＞ 128 | ＞ 128 | ＞ 128 | ＞ 128 |
| 6 | ＞ 128 | ＞ 128 | ＞ 128 | ＞ 128 | ＞ 128 | ＞ 128 | ＞ 128 | ＞ 128 | ＞ 128 | ＞ 128 | ＞ 128 |
| 7 | 0.25 | 0.25 | 0.25 | 2 | 0.5 | 2 | ＞ 128 | ＞ 128 | ＞ 128 | ＞ 128 | ＞ 128 |
| Daptomycin | 1 | 4 | 8 | 8 | 32 | 2 | - | - | - | - | - |
| Vancomycin | 1 | 2 | 8 | 4 | ＞ 128 | 2 | - | - | - | - | - |
| Oxacillin | 0.25 | 256 | 512 | 32 | ＞ 512 | 0.25 | - | - | - | - | - |
| levofloxacin | 0.125 | 0.25 | 128 | 1 | 64 | 0.25 | 1 | ＞ 128 | 1 | 0.25 | 0.06 |
| Azithromycin | 1 | ＞ 128 | ＞ 128 | 2 | ＞ 128 | 32 | 32 | 64 | 32 | 32 | 4 |
